# Efficacy and safety of 8-week regimens for the treatment of rifampicin-susceptible pulmonary tuberculosis (TRUNCATE-TB): a prespecified exploratory analysis of a multi-arm, multi-stage, open-label, randomised controlled trial

**DOI:** 10.1016/S1473-3099(25)00151-3

**Published:** 2025-10

**Authors:** Nicholas I Paton, Christopher Cousins, Intan P Sari, Erlina Burhan, Nan-Kai Ng, Victoria B Dalay, Celina Suresh, Tutik Kusmiati, Ka Lip Chew, Vincent M Balanag, Qingshu Lu, Rovina Ruslami, Irawaty Djaharuddin, Jani J R Sugiri, Rholine S Veto, Christine Sekaggya-Wiltshire, Anchalee Avihingsanon, Jitendra Kumar Saini, Padmasayee Papineni, Andrew J Nunn, Angela M Crook, TRUNCATE-TB Trial Team, Erlina Burhan, Fathiyah Isbaniah, Ibrahim N.I.P. Dharmawan, Assica P.A. Hakiman, Hera Afidjati, Diadikma Belarosa, Aga Krisnanda, Nadia U.A. Hadi, Jihaan Hafirain, Dedy Aditia, Tutik Kusmiati, Soedarsono Soedarsono, Deby Kusumaningrum, Ridwan Yasin, Nur S.R. Panenggak, Randy D. Kurniawan, Sri Rejeki, Novi Aryanti, Rovina Ruslami, Prayudi Santoso, Alamanda Larasmanah, Naufal Ihsan, Yuanita Gunawan, Sheila Sumargo, Vycke Yunivita, Irawaty Djaharuddin, Eliana Muis, Nurjannah Lihawa, Nasrum Massi, Yusfiana Majid, A. Siti Kahfiah Mukhlis, Imam Nurjaya, Siti Arifah Lacante, Jani J.R. Sugiri, Gede Sasmika Suwandi, Kristo Kurniawan, Santony Santony, Herman Liem, Tiar Oktavian Effendi, Maria Kristiani, Ni Made Rini, Jatu Aphridasari, Sandy Kurniawan, Dewi Astarini, Linda Soebroto, Titiek Sulistyowati, Yoeke Rasita, Andriansjah Rukmana, Piamlarp Sangsayunh, Thanyanuch Sanchat, Phornchai Pingsusaen, Krisana Cheewakul, Sasithorn Bureechai, Waraporn Thuansuwan, Jirakan Boonyasopun, Karntheera Sangkaew, Sirijit Rattanawai, Anchalee Avihingsanon, Sivaporn Gatechompol, Hay Mar Su Lwin, Win Min Han, Thornthun Ureaphongsukkit, Prachya Chaiyahong, Pornmalai Suriya, Sasiwimol Ubolyam, Anuntaya Uanithirat, Apicha Mahanontharit, Plengsri Lertarrom, Supunnee Jirajariyavej, Stanrat Kanokdeeseerat, Kanyapat Wongwutcharajirakul, Victoria Basa Dalay, Maria Marissa I. Golla, Emmanuel A. Gutierrez, Marietto L. Partosa, Genevieve V. Bayas, Cynthia G. Wagayen, Darecil B. Gelina, Eleonor S. Garcia, Angelita G. Pabruada, Laarean R. Perlas, Vincent M. Balanag, Nerissa A. Donato, Krizia Chloe R. Rivera, Paula Cindy M Villajuan, Zyra Zafe Del Rosario, Thelma E. Tupasi, Rholine Gem Martin Sindingan Veto, Maria Begonia Rejaba Baliwagan, Glenn Ibana Balane, Anthony A. Geronimo, Elsie Marie B. Dela Cruz, Anabella M. Guardiario, Maria Philina P. Villamor, Ma. Bernardita Sarcauga Chua, Peter Dela Torre Blanco, Rose Marie L. Cagwin, Karenza Antipuesto Camus, Jubert P. Benedicto, Ma. Kriselda Karlene G. Tan, Michelle B. Recana-Nieva, Rose Ann A. Espiritu-Villasfer, Rohit Sarin, Jitendra Kumar Saini, Prabhpreet Sethi, Mohit Tomar, Manpreet Bhalla, Shivani, Arti, Shyam Singh Bisht, Christine Sekaggya-Wiltshire, Ruth Mirembe Nabisere, Brian Otaalo, Jesca Asienzo, Letisha Najjemba, Juliet Nampala, Lucy Alinaitwe, Eunice Kaguiri, Cissy Kityo, Henry Mugerwa, Timothy Arthur Serumaga, Timothy Masaba, Theresa Najjuuko, Joseph Akol, Caroline Kayiza, Abbas Lugemwa, Sharif Musumba, Ibrahim Yawe, Assumpta Katusiime, Beatrice Tumusiime, Mariam Kasozi, Myalo Sula, Rogers Ankunda, Nicholas Iain Paton, Christopher Cousins, Celina Suresh, Nan Kai Ng, Elena Wan Yi Lur, Shariba Munawara, Felic Fanusi, Gail Cross, Anushia Panchalingham, Gianna Yau, Padmasayee Papineni, Kristina Rutkute, Meera Gurumurthy, Pauline Yoong, Ka Lip Chew, Intan Permata Sari, QingShu Lu, Shu Ling Lee, Mihir Gandhi, Yogesh Pokharkar, Rajesh Babu Moorakonda, Yin Bun Cheung, Angela M. Crook, Karen Sanders, Patrick Phillips, Andrew J. Nunn, Jody Phelan, Martin Hibberd, Catharina Aprillia, Amalia Rachmawati, Larra Minnellie M. Esconde, Bianca Austria, Kanitta Pussadee, Hathairat Prushyapornsri, Pornkhaun Mungklang, Chanapha Janpanich, Suzan Wilmott, Taran Bedi, Nicholas Iain Paton, Christopher Cousins, Celina Suresh, Padmasayee Papineni, Ibrahim Abubakar, Karen Sanders, Angela M. Crook, Andrew J. Nuun, Geraint Davies, Charles Gilks, Sushil Pandey, Abdul Razak Bin Abdul Muttalif, Basanta Kumar Parajuli, Kaewta Sangsuk, Nicholas Iain Paton, Erlina Burhan, Vincent M. Balanag, Anchalee Avihingsanon, Christine Sekaggya-Wiltshire, Rohit Sarin, Angela M. Crook, Andrew J. Nuun, Guy Thwaites, Matthew Law, Janice Caoili, Reinout Van Crevel

**Affiliations:** a Infectious Diseases Translational Research Programme and Yong Loo Lin School of Medicine, National University of Singapore, Singapore; b Department of Clinical Research, London School of Hygiene & Tropical Medicine, London, UK; c National University Hospital, Singapore; d Persahabatan Hospital, Jakarta, Indonesia; e De La Salle Medical and Health Sciences Institute, Cavite, Philippines; f Dr. Soetomo Hospital, Surabaya, Indonesia; g Lung Centre of the Philippines, Quezon City, Philippines; h Singapore Clinical Research Institute, Singapore; i Fortrea Singapore, Singapore; j Universitas Padjadjaran, Bandung, Indonesia; k Dr Wahidin Sudirohusodo Hospital, Makassar, Indonesia; l Saiful Anwar Hospital, Malang, Indonesia; m Tropical Disease Foundation, Makati, Philippines; n Infectious Diseases Institute, Makerere University, Kampala, Uganda; o HIV-NAT, Thai Red Cross AIDS Research Centre and Centre of Excellence in Tuberculosis, Faculty of Medicine, Chulalongkorn University, Bangkok, Thailand; p National Institute of Tuberculosis and Respiratory Diseases, New Delhi, India; q Medical Research Council Clinical Trials Unit, University College London, London, UK

## Abstract

**Background:**

WHO recommends a 2-month optimal duration for new drug regimens for rifampicin-susceptible tuberculosis. We aimed to investigate the efficacy and safety of the 8-week regimens that were assessed as part of the TRUNCATE management strategy of the TRUNCATE-TB trial.

**Methods:**

TRUNCATE-TB was a multi-arm, multi-stage, open-label, randomised controlled trial in which participants aged 18–65 years with rifampicin-susceptible pulmonary tuberculosis were randomly assigned via a web-based system, using permuted blocks, to 24-week standard treatment (rifampicin, isoniazid, pyrazinamide, and ethambutol) or the TRUNCATE management strategy comprising initial 8-week treatment, then post-treatment monitoring and re-treatment where needed. The four 8-week regimens comprised five drugs, modified from standard treatment: high-dose rifampicin and linezolid, or high-dose rifampicin and clofazimine, or bedaquiline and linezolid, all given with isoniazid, pyrazinamide, and ethambutol; and rifapentine, linezolid, and levofloxacin, given with isoniazid and pyrazinamide. Here, we report the efficacy (proportion with unfavourable outcome; and difference from standard treatment, assessed via Bayesian methods) and safety of the 8-week regimens, assessed in the intention-to-treat population. This prespecified exploratory analysis is distinct from the previously reported 96-week outcome of the strategy in which the regimens were deployed. This trial is registered with ClinicalTrials.gov (NCT03474198).

**Findings:**

Between March 21, 2018, and March 26, 2020, 675 participants (674 in the intention-to-treat population) were enrolled and randomly assigned to the standard treatment group or one of the four 8-week regimen groups. Two 8-week regimens progressed to full enrolment. An unfavourable outcome (mainly relapse) occurred in seven (4%) of 181 participants on standard treatment; 46 (25%) of 184 on the high-dose rifampicin and linezolid-containing regimen (adjusted difference 21·0%, 95% Bayesian credible interval [BCI] 14·3–28·1); and 26 (14%) of 189 on the bedaquiline and linezolid-containing regimen (adjusted difference 9·3% [4·3–14·9]). Grade 3–4 adverse events occurred in 24 (14%) of 181 participants on standard treatment, 20 (11%) of 184 on the rifampicin-linezolid regimen, and 22 (12%) of 189 on the bedaquiline-linezolid regimen.

**Interpretation:**

Efficacy was worse with 8-week regimens, although the difference from standard treatment varied between regimens. Even the best 8-week regimen (bedaquiline-linezolid) should only be used as part of a management strategy involving post-treatment monitoring and re-treatment if necessary.

**Funding:**

Singapore National Medical Research Council; UK Department of Health and Social Care; UK Foreign, Commonwealth, and Development Office; UK Medical Research Council; Wellcome Trust; and UK Research and Innovation Medical Research Council.

## Introduction

The current 6-month standard-of-care treatment for rifampicin-susceptible tuberculosis cures almost all participants in clinical trials but performs less well in treatment programmes, largely due to difficulties in sustaining adherence. Shortening the treatment duration might enhance motivation and adherence and thereby improve outcomes. The WHO Target Regimen Profile recommends an optimal target duration of 2 months for new regimens developed for rifampicin-susceptible tuberculosis.^1^

Only one trial has published efficacy data on treatment at this duration, finding a 16% relapse rate by 1 year in a group of smear-negative participants.^2^ Trials assessing higher doses of rifampicin or substitution with rifapentine did not show non-inferior efficacy of 4-month regimens based on these modifications alone.^3^, ^4^ However, a 4-month regimen in which rifampicin was substituted with rifapentine and ethambutol was substituted with moxifloxacin did show non-inferiority to standard treatment.^3^

**Research in context**

**Evidence before this study**

We searched PubMed, with no start date or language restrictions, using the terms “tuberculosis” and “randomised trial” and “2 months duration” for articles published from database inception until Aug 31, 2024. We identified just one randomised trial, done in 1976, in which 73 smear-negative, culture-positive participants received a 2-month regimen (rifampicin, isoniazid, pyrazinamide, and streptomycin); of these, 12 (16%) relapsed within 12 months.

**Added value of this study**

TRUNCATE-TB is the first substantive trial to assess treatment with novel 8-week, five-drug regimens, based on modifications to standard treatment. In this prespecified exploratory analysis, all four 8-week regimens had efficacy lower than the standard 24-week regimen. The five-drug regimen containing bedaquiline and linezolid had a high probability of non-inferior efficacy in participants with a low baseline bacillary burden. Regimens containing high-dose rifampicin and rifapentine had a high frequency of low-grade gastrointestinal adverse events, not noted in previous trials.

**Implications of all the evidence**

None of the 8-week regimens based on modified standard treatment would be expected to achieve non-inferiority in an unselected population in a definitive phase 3 efficacy trial. Even the best 8-week bedaquiline-linezolid regimen should only be used as part of the TRUNCATE management strategy within which it was previously found to be effective. Low-grade adverse events, such as gastrointestinal events, are common and might impair the tolerability and effectiveness of shorter regimens.

The TRUNCATE-TB trial was based on the premise that an 8-week regimen, comprising modifications to standard treatment, would have more relapses than the 24-week standard treatment regimen, but that the excess of relapses would be modest. The trial tested the hypothesis that, despite the excess relapses, the TRUNCATE management strategy—comprising initial 8-week treatment, prolonged follow-up and re-treatment for relapse (with standard treatment) when needed (in the minority of cases)—would achieve a long-term clinical outcome that was non-inferior to standard treatment.^5^

In keeping with this hypothesis, the TRUNCATE management strategy was analysed not by conventional outcomes for assessing the efficacy of a drug regimen, but instead by outcomes appropriate for assessing the whole strategy. Thus, efficacy was not evaluated by relapses following the initial 8-week treatment but instead by longer-term clinical outcomes, defined as being alive, free from active tuberculosis and not on tuberculosis treatment at week 96. Similarly, safety was not evaluated by adverse events during the initial 8-week treatment but instead by all adverse events up to week 96, including those associated with relapse and occurring during re-treatment with standard treatment. Benefits were not assumed as a given from the initial short treatment duration but instead evaluated by a range of outcomes including overall time on treatment to week 96, quality of life, and acceptability of the strategy. The trial showed non-inferior efficacy of the TRUNCATE management strategy based on long-term clinical outcomes, without safety concerns and with benefits including reduced average total time on treatment. This was achieved when the strategy used initial treatment with a regimen containing bedaquiline and linezolid. The outcome of the strategy analysis has been previously reported.^5^

The trial deployed four 8-week regimens (comprising five drugs) as the initial treatment within this strategy. This approach provided a unique opportunity to explore the efficacy and safety of 8-week regimens that incorporated modifications to the standard regimen, including drugs of contemporary interest (high-dose rifampicin, rifapentine, clofazimine, linezolid, and bedaquiline). It also provided an opportunity to explore how far we are from attaining an 8-week regimen that might show non-inferior efficacy to standard treatment. Here, we present a prespecified analysis of the 8-week regimens using conventional relapse-based efficacy outcomes and on-treatment safety outcomes, none of which were reported in the previous analysis of the TRUNCATE management strategy (because they were not relevant to that question).^5^ This analysis is exploratory and intended to inform future regimen development.

## Methods

### Study design and participants

The TRUNCATE-TB trial was a randomised, open-label, seamless phase 2–3, multi-arm multi-stage, 96-week trial conducted at 18 clinical sites in India, Indonesia, the Philippines, Thailand, and Uganda, coordinated by the National University of Singapore. The trial was designed to evaluate the non-inferiority of the TRUNCATE management strategy, reported elsewhere.^5^ Here, we report the prespecified exploratory analysis of the safety and efficacy of the four novel 8-week drug regimens used for initial treatment, distinct from the analysis of the strategy in which they were deployed. The trial was approved by national and local ethics committees and regulatory agencies in all participating countries, and all participants provided written informed consent.

Eligible participants were aged 18–65 years, with symptoms or evidence of tuberculosis on a chest radiograph and a positive Xpert MTB/RIF test or Xpert MTB/RIF Ultra test (Cepheid) without rifampicin resistance. Those with sputum smear grade 3+, chest radiograph cavity exceeding 4 cm, or a positive HIV antibody test were initially not eligible; these exclusion criteria were later removed, following implementation of protocol version 2 (dated Oct 18, 2019). Complete eligibility criteria and a description of changes are provided in the appendix (pp 7–8).

### Randomisation and masking

Participants were randomly assigned in equal proportions to groups open to enrolment. Randomisation was done via a web-based system, pre-programmed with a computer-generated randomisation list; with random permuted blocks (sizes of five and ten); and was stratified by trial site and relapse risk (appendix p 9). The study coordinator at each site entered participant information on the system and was notified of the assigned treatment group.

The trial management team did not have access to aggregate unmasked data except for serious adverse events and pregnancy reports. The trial statistician accessed unmasked data through formal request to the data management group when required for analyses. Site staff and participants were not masked to treatment allocation.

### Procedures

The standard regimen comprised a daily four-drug regimen containing standard-dose rifampicin (10 mg/kg) and isoniazid (5 mg/kg) for 24 weeks, with pyrazinamide (25 mg/kg) and ethambutol (15 mg/kg) for the first 8 weeks; given as fixed-dose combination pills. The four novel 8-week regimens comprised a daily five-drug regimen containing high-dose rifampicin (35 mg/kg) and linezolid (600 mg) with isoniazid, pyrazinamide and ethambutol (standard doses); high-dose rifampicin (35 mg/kg) and clofazimine (200 mg) with isoniazid, pyrazinamide, and ethambutol (standard doses); rifapentine (1200 mg, in place of rifampicin), linezolid (600 mg), and levofloxacin (1000 mg, in place of ethambutol) with isoniazid and pyrazinamide (standard doses); or bedaquiline (400 mg daily for two weeks then 200 mg three times a week, in place of rifampicin) and linezolid (600 mg) with isoniazid, pyrazinamide, and ethambutol (standard doses). Further details of regimens and the rationale for their selection are provided in the appendix (pp 9–11). The assigned 8-week regimen could be extended until week 12 to make up missed doses or for persistent clinical disease (symptoms and positive sputum smear) at weeks 8 or 10; or switched to the standard regimen (to complete 24 weeks) for persistent clinical disease at week 12 or earlier if not tolerated (appendix p 58).

Treatment was supervised daily at least until completion of the four-drug phase of the standard regimen or the assigned 8-week regimen, through an approach tailored to the participant and local setting.

Participants were monitored by symptom assessment and sputum smear; sputum culture results were returned to clinicians for information; and additional tests were done for suspected relapse. Participants meeting prespecified relapse criteria (appendix p 12) were re-treated for at least 24 weeks with the standard regimen, adjusted by resistance profile.

The trial schedule is provided in the appendix (pp 13–14). Clinic visits were scheduled every 1–4 weeks up to week 24; then every 12 weeks up to week 96; monthly telephone calls to participants were interspersed between clinic visits from week 30. At all visits, the presence of tuberculosis symptoms and adverse events were ascertained with a standard checklist and spontaneous reports. Adverse events were graded with Division of AIDS toxicity criteria^6^ and adherence assessed from treatment records and participant interviews. A chest radiograph was done at screening, weeks 8 and 96, end of treatment, and when relapse was suspected. An electrocardiogram (ECG) was done at screening, baseline, and at weeks 1, 4, and 8.

Sputum was collected for smear (local method, graded with WHO scale) and liquid culture (Mycobacteria Growth Indicator Tube [MGIT] system, Becton Dickinson, Franklin Lakes, NJ, USA) at all scheduled clinic visits and when relapse was suspected. Drug resistance was assessed by phenotypic susceptibility testing at baseline (standard regimen drugs and drugs to which the participant was subsequently exposed) and repeated at relapse (appendix p 15). Whole-genome sequencing was done on all baseline and relapse isolates.

### Outcomes

The primary outcome for analysis of regimen efficacy was an unfavourable outcome by week 96, assessed centrally by the trial statistician following a prespecified algorithm, on unmasked data at the end of the trial (appendix pp 16–19). An unfavourable outcome was assigned for reasons including treatment failure, relapse, or death. An unassessable outcome was assigned for reasons including non-adherence to or non-completion of the initial assigned regimen or switches or restart of treatment without positive culture. Participants were classified by the first event that defined an unfavourable or unassessable outcome, or as having a favourable outcome if there was no such event by week 96.

The major difference between an unfavourable outcome (used for the current analysis of drug regimen efficacy) and an unsatisfactory outcome (used for the previous analysis of the TRUNCATE management strategy) is that treatment failure and relapse count as an unfavourable outcome but not as an unsatisfactory outcome if re-treatment had been completed by week 96 and participants no longer had active tuberculosis (appendix pp 16–19, 36, 59). Additional, prespecified secondary efficacy outcomes were time to unfavourable outcome and time to treatment failure or relapse, both reported here; and the change in the proportion of lung involvement on chest radiograph (to be reported separately).

The main safety outcome was the occurrence of an adverse event of grade 3–4 severity, with onset or increase in grade between the baseline day and within 30 days after cessation of treatment with the assigned regimen. Other prespecified safety outcomes were adverse events of grade 3–4 severity related to the assigned regimen, serious adverse events, treatment-limiting adverse events (leading to permanent dose reduction or treatment cessation), and adverse events of special interest (known important toxicities associated with these study drugs). We also report adverse events occurring in more than 10% of participants in one or more treatment groups. A summary of all efficacy, safety, and other outcomes is given in the appendix (pp 20–22).

Further details on trial conduct are in the protocol (appendix pp 70–239).

### Statistical analysis

The TRUNCATE management strategy was developed on the premise that 8-week regimens would have more relapses than 24-week standard treatment. Therefore, for this drug regimen efficacy analysis, we did not seek to pose and refute a hypothesis of non-inferiority with a conventional (frequentist) approach because such a hypothesis was not considered plausible. Instead, we used a Bayesian approach to estimate risk difference (with 95% Bayesian credible intervals [BCIs]) and the probability that the true risk difference lies above or below a specified threshold (appendix pp 23–27).

An unfavourable outcome was analysed in the intention-to-treat population, which excluded participants randomly assigned in error and withdrawn before receiving trial medication. Participants with an unassessable outcome were included in the main analysis (counted as not unfavourable) and excluded in a sensitivity analysis (described below). The pairwise difference in the proportion of participants with an unfavourable outcome between each 8-week regimen group and the standard treatment group was estimated from regression models with Bayesian methods. The models used the number of participants with an unfavourable outcome as the dependent variable (assumed binomial distribution), and treatment group, country, and baseline relapse risk (defined by sputum smear status and chest X-ray cavitation) as independent variables, with a logit link function. An uninformative (normal) prior was assumed for the intercept and regression parameter for each independent variable.^7^ The precision of estimates for groups of 180 participants (sample size set for the strategy analysis, previously reported)^5^ and underlying assumptions are described in the appendix (pp 28–29).

Bayesian probability was determined for the difference in the proportion of participants with an unfavourable outcome between 8-week regimens and standard treatment being no greater than 12% (the threshold previously used to evaluate non-inferiority in trials of 4-month regimens; the justification is stronger for an 8-week regimen).^8^, ^9^ As a framework for interpretation (not a hypothesis to be tested), we specified a probability exceeding 0·95 as being sufficient to regard a regimen as non-inferior (similar to the way a one-sided 95% CI might be used to assess whether a non-inferiority hypothesis has been met in a frequentist analysis; appendix pp 23–27).^10^, ^11^ Sensitivity analyses of the primary outcome were done in assessable, culture-positive, and fully susceptible populations, and by classifying participants as unassessable if they missed more than 3 days or more than 14 days of assigned treatment within the first 56 trial days (versus the 7-day threshold for unassessable classification in the primary analysis) or if they took more than 58 days of assigned treatment (ignored in the primary analysis); or if they had only a single positive culture at treatment switch, failure, or relapse (defined as unfavourable in the primary analysis). Additional prespecified sensitivity analyses relating to definitions of disease activity did not change outcome assignment and were not performed. The primary outcome was analysed in prespecified subgroups for standard treatment and the fully enrolled 8-week regimen groups.

Time to unfavourable outcome and time to treatment failure or relapse (from randomisation to the earliest of the first event or week 96 visit, whichever occurred first) were compared between treatment groups with Kaplan-Meier methodology and by estimation of hazard ratios (HRs) with stratified Cox regression models, with country and baseline relapse risk as the stratification factors.

The proportion of participants with at least one grade 3–4 adverse event or at least one serious adverse event was compared between standard treatment and the fully enrolled 8-week regimen groups with the χ^2^ test. All other safety comparisons were descriptive. An absolute excess of at least ten percentage points in the frequency of a specific event or category of events in an 8-week regimen group compared to the standard treatment group was regarded as substantive.

An independent data monitoring committee (IDMC) reviewed two interim analyses, after 30 and 70 participants in the standard treatment group had reached week 24, but did not recommend discontinuation of enrolment to any group. The trial steering committee discontinued enrolment to two 8-week regimens, one after each IDMC review meeting, to ensure that sample size requirements could be met in remaining groups. The selection of groups for discontinuation was made on pragmatic grounds, masked to outcome data (appendix p 30).

Details of analyses are in the statistical analysis plan (appendix pp 369–81). All analyses were done in SAS (version 9.4).

This trial is registered with ClinicalTrials.gov (NCT03474198) and is now completed.

### Role of the funding source

The funders of the study had no role in study design, data collection, data analysis, data interpretation or writing of the report.

## Results

**Figure 1:**
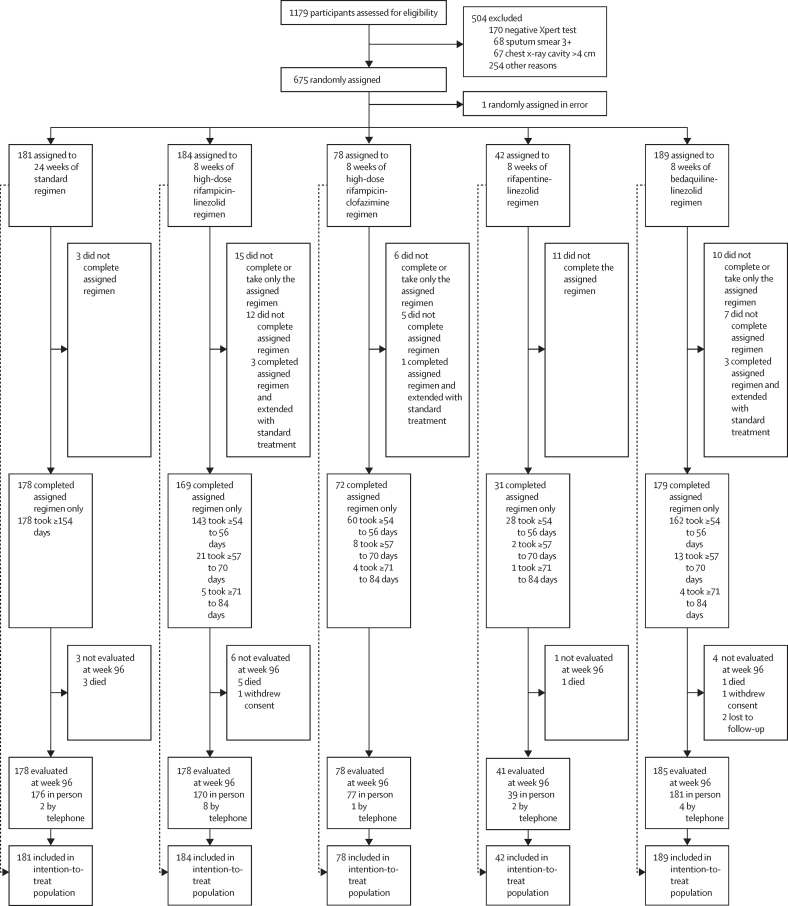
Trial profile

Between March 21, 2018, and March 26, 2020, 1179 potential participants were screened and 675 were randomly assigned to the standard treatment group or one of four 8-week regimen groups (as part of the TRUNCATE management strategy; figure 1). One of these participants was randomly assigned in error and withdrawn before taking the assigned treatment. Reasons for non-eligibility are shown in the appendix (p 31). All groups met the criteria to continue at the interim analysis but enrolment to the rifampicin-clofazimine group (78 participants) and the rifapentine-linezolid group (42 participants) was stopped early for pragmatic reasons (appendix p 30). The dose of rifampicin was reduced from 35 mg/kg to 20 mg/kg in the rifampicin-linezolid group (after 88 participants were enrolled), as a precaution following a death from acute hepatic failure. Four (1%) participants were lost to follow-up or withdrew before week 96.

Baseline characteristics of the 674 participants in the intention-to-treat population were similar across groups; overall, 61% participants had medium or high bacillary burden on Xpert MTB/RIF test (table 1). All participants took at least one dose of the assigned regimen. In the rifampicin-linezolid, rifampicin-clofazimine and bedaquiline-linezolid groups, at least 92% completed the assigned treatment, with mean adherence of at least 98%; and at least 77% stopped at exactly 56 treatment days. In the rifapentine-linezolid group, treatment completion was lower (74%), mainly due to regimen switches associated with adverse events (details below).

**Table 1:** Baseline characteristics of the intention-to-treat population

			**Standard 24-week treatment regimen (n=181)**	**Rifampicin-linezolid 8-week regimen (n=184)**	**Rifampicin-clofazimine 8-week regimen (n=78)**	**Rifapentine-linezolid 8-week regimen (n=42)**	**Bedaquiline-linezolid 8-week regimen (n=189)**	**Overall (N=674)**
Sex*								
	Male		119 (66%)	113 (61%)	48 (62%)	25 (60%)	116 (61%)	421 (62%)
	Female		62 (34%)	71 (39%)	30 (38%)	17 (40%)	73 (39%)	253 (38%)
Age								
	18–34 years		104 (57%)	109 (59%)	51 (65%)	26 (62%)	95 (50%)	385 (57%)
	35–49 years		59 (33%)	57 (31%)	21 (27%)	11 (26%)	70 (37%)	218 (32%)
	50–65 years		18 (10%)	18 (10%)	6 (8%)	5 (12%)	24 (13%)	71 (11%)
Country								
	Indonesia		78 (43%)	73 (40%)	38 (49%)	23 (55%)	82 (43%)	294 (44%)
	Philippines		61 (34%)	66 (36%)	32 (41%)	15 (36%)	63 (33%)	237 (35%)
	Thailand		10 (6%)	15 (8%)	8 (10%)	4 (10%)	12 (6%)	49 (7%)
	Uganda†		28 (15%)	25 (14%)	0	0	27 (14%)	80 (12%)
	India†		4 (2%)	5 (3%)	0	0	5 (3%)	14 (2%)
Bodyweight, kg			49·6 (43·0–58·0)	49·9 (44·3–57·0)	48·1 (44·2–57·2)	49·7 (45·1–57·3)	49·5 (44·2–57·1)	49·5 (44·0–57·2)
BMI, kg/m^2^			18·9 (17·3–21·1)	18·9 (17·2–21·1)	18·8 (16·8–21·5)	18·2 (16·4–22·4)	19·3 (17·1–22·3)	19·0 (17·1–21·5)
BMI								
	<17 kg/m^2^		39 (21%)	42 (23%)	21 (27%)	13 (31%)	47 (25%)	162 (24%)
	17 kg/m^2^ to <18·5 kg/m^2^		40 (22%)	38 (21%)	14 (18%)	9 (21%)	29 (15%)	130 (19%)
	≥18·5 kg/m^2^		102 (56%)	104 (57%)	43 (55%)	20 (48%)	113 (60%)	382 (57%)
Smoking status								
	Current smoker		34 (19%)	33 (18%)	15 (19%)	8 (19%)	31 (16%)	121 (18%)
	Former smoker		58 (32%)	63 (34%)	24 (31%)	13 (31%)	51 (27%)	209 (31%)
	Never-smoker		89 (49%)	88 (48%)	39 (50%)	21 (50%)	107 (57%)	344 (51%)
CXR lung area affected								
	<25%		46 (25%)	62 (34%)	28 (36%)	12 (29%)	53 (28%)	201 (30%)
	25–50%		94 (52%)	87 (47%)	36 (46%)	24 (57%)	98 (52%)	339 (50%)
	>50%		41 (23%)	35 (19%)	14 (18%)	6 (14%)	38 (20%)	134 (20%)
CXR cavitation								
	Absent		87 (48%)	83 (45%)	41 (53%)	19 (45%)	81 (43%)	311 (46%)
	Largest cavity ≤4 cm		90 (50%)	96 (52%)	37 (47%)	23 (55%)	106 (56%)	352 (52%)
	Largest cavity >4 cm		4 (2%)	5 (3%)	0	0	2 (1%)	11 (2%)
WHO smear grade‡								
	Negative		46/180 (26%)	57/184 (31%)	26/78 (33%)	12/41 (29%)	50/189 (26%)	191/672 (28%)
		Scanty	27/180 (15%)	28/184 (15%)	12/78 (15%)	7/41 (17%)	24/189 (13%)	98/672 (15%)
		1+	38/180 (21%)	48/184 (26%)	25/78 (32%)	13/41 (32%)	53/189 (28%)	177/672 (26%)
		2+	44/180 (24%)	37/184 (20%)	8/78 (10%)	7/41 (17%)	38/189 (20%)	134/672 (20%)
		3+	25/180 (14%)	14/184 (8%)	7/78 (9%)	2/41 (5%)	24/189 (13%)	72/672 (11%)
Xpert MTB/RIF bacillary burden§								
	Very low		27/175 (15%)	24/174 (14%)	8/74 (11%)	3/37 (8%)	17/186 (9%)	79/646 (12%)
	Low		40/175 (23%)	48/174 (28%)	22/74 (30%)	11/37 (30%)	52/186 (28%)	173/646 (27%)
	Medium		72/175 (41%)	80/174 (46%)	31/74 (42%)	15/37 (41%)	73/186 (39%)	271/646 (42%)
	High		36/175 (21%)	22/174 (13%)	13/74 (18%)	8/37 (22%)	44/186 (24%)	123/646 (19%)
Positive sputum culture			166 (92%)	168 (91%)	68 (87%)	39 (93%)	172 (91%)	613 (91%)
Drug resistance¶								
	Isoniazid		12/163 (7%)	14/170 (8%)	5/67 (7%)	2/39 (5%)	10/171 (6%)	43/610 (7%)
	Pyrazinamide		5/146 (3%)	2/154 (1%)	3/60 (5%)	1/34 (3%)	3/155 (2%)	14/549 (3%)
	Ethambutol		1/163 (1%)	0	2/67 (3%)	0	2/171 (1%)	5/610 (1%)
Relapse risk‖								
	Low		47 (26%)	57 (31%)	26 (33%)	13 (31%)	50 (26%)	193 (29%)
	Intermediate		105 (58%)	111 (60%)	45 (58%)	27 (64%)	113 (60%)	401 (59%)
	High		29 (16%)	16 (9%)	7 (9%)	2 (5%)	26 (14%)	80 (12%)

Data are n (%), n/N (%), or median (IQR). The table shows baseline characteristics of the intention-to-treat population, which included all randomly assigned participants except for one who was randomly assigned in error and immediately withdrawn. Percentages might not total 100 because of rounding. Data previously published;^5^ updated with additional Xpert MTB/RIF and drug resistance test results. CXR=chest radiograph.

* Sex was as assigned at birth. Information on gender identity was not collected.

† Enrolment into the rifampicin-clofazimine and rifapentine-linezolid groups was discontinued before the full sample size was attained, following the trial design; this was before opening sites in Uganda and India.

‡ Sputum smears were not available for two participants; sputum smear graded with WHO scale; the result is the highest of all smears performed between screening and baseline.

§ Cycle threshold result was not available for 28 participants; Xpert MTB/RIF was done in 527 participants and Xpert MTB/RIF Ultra in 119 participants; conversion of cycle threshold to estimate of bacillary burden used consensus published thresholds; five participants with result of “trace” on Ultra were allocated to the category of very low bacillary burden.

¶ Phenotypic testing result from first available positive culture. No participant had phenotypic resistance at baseline to rifampicin (610 tested), linezolid (202 tested), clofazimine (58 tested), bedaquiline (106 tested), and levofloxacin (504 tested).

‖ Relapse risk categories are defined as low (smear negative and no CXR cavity >4 cm), intermediate (smear positive at grade ≤2+ and no CXR cavity >4 cm) and high (smear positive grade 3+ or CXR cavity >4 cm, or both), based on the maximum sputum smear grade and largest cavity size obtained on any test done between screening and baseline; two participants who attempted but were unable to produce sputum at these study visits were regarded as smear negative for classifying relapse risk (neither had cavitation on CXR).

**Figure 2:**
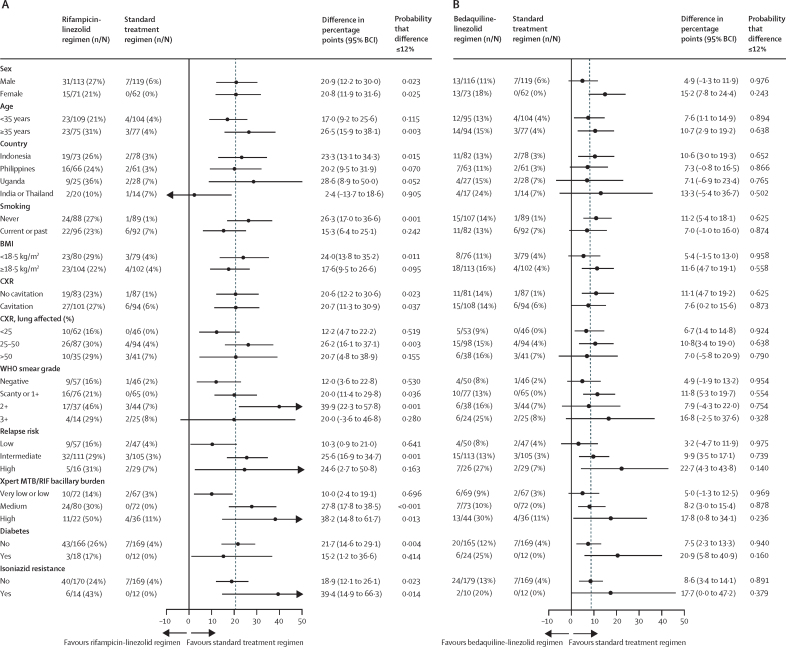
Subgroup analyses of unfavourable outcome The figure shows the subgroups and the proportion of participants with an unfavourable outcome in the rifampicin-linezolid group (A) and bedaquiline-linezolid group (B) compared with the standard treatment group. The point estimate of the difference in proportions between the treatment groups is shown, as well as the probability that the risk difference is no greater than 12%, both estimated from a regression model with Bayesian methods, including country and baseline relapse risk as independent variables. BCI=Bayesian credible interval. CXR=chest radiograph.

Across all groups, 96 participants met the criteria for an unfavourable outcome, 85 (89%) due to treatment failure or relapse (table 2); differences in outcome classification between this regimen efficacy analysis and the previous TRUNCATE management strategy analysis are shown in the appendix (pp 36, 59). In the standard treatment group, an unfavourable outcome in the intention-to-treat population was observed in seven (4%) of 181 participants. In the rifampicin-linezolid group, an unfavourable outcome was observed in 46 (25%) of 184 participants (adjusted risk difference *vs* standard treatment 21·0% [95% BCI 14·3–28·1]; table 2). The probability of the risk difference being no greater than 12% was 0·004 (table 2) and similarly low in sensitivity (appendix pp 37–38) and subgroup analyses (figure 2A) and in participants assigned 35 mg/kg rifampicin (appendix pp 39–40).

**Table 2:** Primary outcome efficacy analysis

		**Standard 24-week treatment regimen (n=181)**	**Rifampicin-linezolid 8-week regimen (n=184)**	**Rifampicin-clofazimine 8-week regimen (n=78)**	**Rifapentine-linezolid 8-week regimen (n=42)**	**Bedaquiline-linezolid 8-week regimen (n=189)**
Primary outcome						
	Unfavourable outcome	7 (4%)	46 (25%)	10 (13%)	7 (17%)	26 (14%)
	Estimated absolute risk (%, 95% BCI)	3·5% (1·5–6·6)	24·5% (18·4–31·0)	11·2% (5·4–18·9)	14·9% (6·2–26·8)	12·9% (8·6–18·0)
	Probability that absolute risk ≤20%	1·000	0·076	0·984	0·831	0·996
	Risk difference (%, 95% BCI)*	..	21·0% (14·3–28·1)	8·9% (2·0–17·2)	10·8% (0·1–23·7)	9·3% (4·3–14·9)
	Probability that risk difference ≤12%	..	0·004	0·800	0·606	0·837
Outcome classification						
	Unfavourable outcome (total)	7 (4%)	46 (25%)	10 (13%)	7 (17%)	26 (14%)
	Switched treatment with positive culture	0	0	0	0	1
	Failure at end of treatment	0	1	0	0	0
	Relapse confirmed†	4	40	10	6	21
	Relapse unconfirmed‡	0	0	0	0	3
	Death, except unrelated§	2	3	0	1	0
	Not seen at week 96, unfavourable¶	1	2	0	0	1
Unassessable outcome (total)		6 (3%)	29 (16%)	12 (15%)	13 (31%)	16 (8%)
	Incomplete initial treatment‖	1	9	4	10	6
	Missed >7 days' treatment in first 56 days	3	14	4	1	6
	Switched treatment without positive culture	0	3	1	0	2
	Restarted treatment without failure or relapse	1	3	3	2	0
	Death, unrelated**	1	0	0	0	0
	Not seen at week 96, unassessable††	0	0	0	0	2
Favourable outcome (total)		168 (93%)	109 (59%)	56 (72%)	22 (52%)	147 (78%)

Data are n or n (%) unless otherwise stated. Primary outcome classification as unfavourable or unassessable was determined by the first outcome event to meet criteria; classification as favourable was determined by the absence of any event by week 96. Analyses were done in the intention-to-treat population. Absolute risk and 95% Bayesian credible interval (95% BCI), risk difference (95% BCI), probability of absolute risk being 20% or lower, and probability of risk difference being 12% or lower were estimated from regression models with Bayesian methods, with a normal prior (mean 0, variance 100) applied to the intercept and independent variables of treatment group, country (India and Thailand, combined; Indonesia; Philippines; and Uganda) and baseline relapse risk (low; intermediate and high combined), weighted in proportion to the number of participants in each category. Three models were done: one for all participants in the rifampicin-linezolid, bedaquiline-linezolid, and standard treatment groups combined; one for all participants in the rifampicin-clofazimine group with those undergoing contemporary randomisation to the rifampicin-linezolid, bedaquiline-linezolid, and standard treatment groups; and one for all participants in the rifapentine-linezolid group with those undergoing contemporary randomisation to all other groups. Comparisons with the standard treatment group were planned for fully enrolled 8-week regimen groups; these comparisons were performed post-hoc for the two groups in which enrolment was discontinued early.

* Risk difference between the 8-week regimen groups and the standard treatment group (participants undergoing contemporary randomisation) expressed in percentage points.

† Classification as relapse confirmed required at least two positive cultures. Whole-genome sequencing was available in paired baseline and relapse isolates for four of four participants in the standard treatment group; 36 of 40 in the rifampicin-linezolid group; nine of ten in the rifampicin-clofazimine group; six of six in the rifapentine-linezolid group; and 20 of 21 in the bedaquiline-linezolid groups. Strains were related, consistent with relapse in all but two of these cases (one in the rifampicin-clofazimine group, one in the bedaquiline-linezolid group) where the findings were inconclusive (no evidence of re-infection).

‡ Classification as relapse unconfirmed was made when there was only one positive culture before restarting treatment. Whole-genome sequencing was available in paired baseline and relapse isolates for all three participants and in all cases the strains were related, consistent with relapse.

§ Deaths that were not considered unrelated to tuberculosis (or anti-tuberculosis medication) were due to cardiac arrest and possible cerebrovascular accident in the standard treatment group; drug-induced liver injury, cirrhosis, and COVID-19 (each in one participant) in the rifampicin-linezolid group; and possible cerebrovascular accident in the rifapentine-linezolid group.

¶ Participants who did not attend at week 96 were classified as unfavourable if there was no evidence of good treatment outcome at the last attended visit or if they were contacted and had evidence of disease activity at week 96.

‖ Incomplete initial treatment defined as taking less than 154 days total of standard treatment in the standard regimen group or less than 54 days total of the assigned regimen in the 8-week regimen groups.

** Death unrelated to tuberculosis in the standard treatment group was due to cervical carcinoma.

†† Participants who did not attend at week 96 were classified as unassessable if there was evidence of good treatment outcome at the last attended visit but they could not be contacted to assess disease activity at week 96.

In the rifampicin-clofazimine group, an unfavourable outcome was observed in ten (13%) of 78 participants (adjusted risk difference *vs* standard treatment 8·9% [95% BCI 2·0–17·2]); the probability of the risk difference being no greater than 12% was 0·800 (table 2; appendix p 41). In the rifapentine-linezolid group, an unfavourable outcome was observed in seven (17%) of 42 participants (adjusted risk difference *vs* standard treatment 10·8% [95% BCI 0·1–23·7]); the probability of the risk difference being no greater than 12% was 0·606 (table 2; appendix p 41).

In the bedaquiline-linezolid group, an unfavourable outcome was observed in 26 (14%) of 189 participants (adjusted risk difference *vs* standard treatment 9·3% [95% BCI 4·3–14·9]; table 2). The probability of the risk difference being no greater than 12% was 0·837 and exceeded 0·95 in a sensitivity analysis in which treatment switch, failure, or relapse with a single positive culture—counted as unfavourable in the main analysis—was classified as unassessable (appendix pp 37–38), and in several subgroup analyses including those with negative sputum smear, low relapse risk, or very low or low Xpert MTB/RIF bacillary burden (figure 2B).

Time to unfavourable outcome in the 8-week regimen groups was fastest in the rifampicin-linezolid group (HR 9·3 [95% CI 4·2–20·7] *vs* standard treatment) and slowest in bedaquiline-linezolid group (HR 4·2 [95% CI 1·8–9·7] *vs* standard treatment); the rifampicin-clofazimine and rifapentine-linezolid groups had intermediate risk (appendix pp 60–61). Findings were broadly similar for time to treatment failure and relapse (appendix pp 62–63).

Two (1%) participants in the bedaquiline-linezolid group had acquired bacterial phenotypic resistance to bedaquiline (one had baseline isoniazid resistance and poor treatment adherence); cases of unconfirmed resistance (to pyrazinamide or isoniazid) are listed in the appendix (p 42).

In the standard treatment group, 25 (14%) of 181 participants had one or more grade 3–4 adverse events; seven (4%) had a serious adverse event, and four (2%) had a treatment-limiting event (table 3; appendix pp 43–49).

**Table 3:** Grade 3–4, serious, and treatment-limiting adverse events

	**Standard 24-week treatment regimen (n=181)**	**Rifampicin-linezolid 8-week regimen (n=184)**	**Rifampicin-clofazimine 8-week regimen (n=78)**	**Rifapentine-linezolid 8-week regimen (n=42)**	**Bedaquiline-linezolid 8-week regimen (n=189)**
**Participants with at least one adverse event grade of grade 3–4 severity**					
Any	25 (14%)	20 (11%)	8 (10%)	9 (21%)	22 (12%)
Related to anti-tuberculosis medication*	13 (7%)	11 (6%)	6 (8%)	7 (17%)	18 (10%)
**Participants with at least one serious adverse event**					
Any	7 (4%)	8 (4%)	5 (6%)	2 (5%)	5 (3%)
Related to anti-tuberculosis medication*	3 (2%)	5 (3%)	4 (5%)	2 (5%)	3 (2%)
**Participants with at least one adverse event of any grade**					
Any	145 (80%)	158 (86%)	69 (88%)	40 (95%)	161 (85%)
Treatment-limiting†	4 (2%)	9 (5%)	8 (10%)	10 (24%)	9 (5%)

Data are n (%). The table shows the number of participants with at least one incident adverse event in each event category. Incident adverse events are events with a start date or date of increase in grade occurring in the period between the day of the baseline visit and 30 days following the day of cessation (last qualifying day) of the assigned regimen, inclusive. A qualifying day is defined as a day on which treatment was taken with at least 50% of the protocol-mandated dose of all drugs in the assigned regimen (exceptions and detailed definitions are given in the protocol and statistical analysis plan). Events were graded with Division of AIDS Toxicity Criteria. Statistical comparisons were prespecified to be limited to comparison of grade 3–4 adverse events (any) and serious adverse events (any) in fully enrolled treatment groups (rifampicin-linezolid and bedaquiline-linezolid groups) pairwise with the standard treatment group. Comparisons were done with the χ^2^ test. Difference in grade 3–4 adverse events in rifampicin-linezolid versus standard treatment group: 3% (95% CI −3·9 to 9·9), p=0·387. Difference in grade 3–4 adverse events in bedaquiline-linezolid versus standard treatment group: 2% (95% CI −4·9 to 9·0), p=0·568. Difference in serious adverse events in rifampicin-linezolid versus standard treatment group: 0% (95% CI −4·4 to 4·4), p=1·000. Difference in serious adverse events in bedaquiline-linezolid versus standard treatment group: 1% (95% CI −3·1 to 5·3), p=0·601.

* Indicated by the local investigator as being at least possibly related to one or more of the anti-tuberculosis drugs in the assigned regimen.

† Adverse event causing permanent dose reduction of one or more drugs in the assigned regimen or permanent cessation of the assigned regimen before completion of at least 54 qualifying days (for the 8-week regimens) or 154 qualifying days (for the standard 24-week regimen).

Among 184 participants in the rifampicin-linezolid group, the frequency of grade 3–4, serious, and treatment-limiting adverse events was similar to that observed with standard treatment (table 3; appendix pp 43–49). Adverse events of special interest that were more common than standard treatment (by at least 10 percentage points, all grades) were limited to hepatic events (hepatic disorders [n=44, 24%], biliary disorders [n=27, 15%], high ALT [n=48, 26%], and high bilirubin [n=43, 23%]; Hy's law biochemical criteria were met in two [1%] participants, of whom one died; table 4; appendix pp 50, 64–67). Other adverse events (not prespecified as being of special interest) that were more common than standard treatment were nausea (n=43, 23%) and vomiting (n=60, 33%; treatment-limiting in three [2%] participants; table 4; appendix pp 48–49, 68). In participants assigned 35 mg/kg rifampicin, the excess of hepatic events, nausea, and vomiting compared to standard treatment was greater than the excess observed in those assigned 20 mg/kg rifampicin (appendix pp 52–56, 69).

**Table 4:** Adverse events of special interest and common adverse events

		**Standard 24-week treatment regimen (n=181)**		**Rifampicin-linezolid 8-week regimen (n=184)**		**Rifampicin-clofazimine 8-week regimen (n=78)**		**Rifapentine-linezolid 8-week regimen (n=42)**		**Bedaquiline-linezolid 8-week regimen (n=189)**	
		Any grade	Grade 3–4	Any grade	Grade 3–4	Any grade	Grade 3–4	Any grade	Grade 3–4	Any grade	Grade 3–4
**Adverse events of special interest (prespecified)**											
Hepatic events											
	Hepatic disorders (SMQ)	23 (13%)	9 (5%)	44 (24%)	6 (3%)	22 (28%)	4 (5%)	15 (36%)	4 (10%)	14 (7%)	2 (1%)
	Biliary disorders (SMQ)	6 (3%)	1 (1%)	27 (15%)	3 (2%)	20 (26%)	2 (3%)	14 (33%)	3 (7%)	1 (1%)	0
	High ALT*	28 (15%)	6 (3%)	48 (26%)	2 (1%)	16 (21%)	3 (4%)	2 (5%)	0	37 (20%)	2 (1%)
	High bilirubin*	17 (9%)	4 (2%)	43 (23%)	5 (3%)	42 (54%)	3 (4%)	27 (64%)	4 (10%)	5 (3%)	0
	Hy's law*	2 (1%)	..	2 (1%)	..	2 (3%)	..	0	..	0	..
Haematological events											
	Anaemia	7 (4%)	5 (3%)	7 (4%)	2 (1%)	4 (5%)	0	5 (12%)	2 (5%)	22 (12%)	14 (7%)
	Haematopoietic cytopenias (SMQ)	8 (4%)	3 (2%)	7 (4%)	1 (1%)	2 (3%)	0	2 (5%)	0	2 (1%)	0
	Low haemoglobin†	30 (17%)	5 (3%)	29 (16%)	2 (1%)	8 (10%)	0	12 (29%)	2 (5%)	56 (30%)	14 (7%)
	Low neutrophil count†	15 (8%)	3 (2%)	12 (7%)	1 (1%)	4 (5%)	0	2 (5%)	0	8 (4%)	0
Other											
	Peripheral neuropathy (SMQ)	14 (8%)	0	15 (8%)	0	1 (1%)	0	2 (5%)	0	10 (5%)	0
	Optic nerve disorders (SMQ)	0	0	0	0	0	0	0	0	0	0
	Skin hyperpigmentation or discolouration‡	0	0	3 (2%)	0	15 (19%)	0	0	0	1 (1%)	0
	Electrocardiogram QTc prolongation§	2 (1%)	0	2 (1%)	0	10 (13%)	0	1 (2%)	0	7 (4%)	1 (1%)
**Additional adverse events occurring in ≥10% of participants in one or more groups**											
Nasopharyngitis		7 (4%)	0	7 (4%)	0	4 (5%)	0	0	0	20 (11%)	0
Hyponatraemia		11 (6%)	0	12 (7%)	1 (1%)	10 (13%)	0	1 (2%)	0	9 (5%)	0
Dizziness		5 (3%)	0	20 (11%)	1 (1%)	12 (15%)	1 (1%)	7 (17%)	0	16 (8%)	0
Headache		5 (3%)	0	22 (12%)	0	4 (5%)	0	6 (14%)	1 (2%)	19 (10%)	1 (1%)
Abdominal pain		21 (12%)	0	33 (18%)	0	13 (17%)	0	6 (14%)	0	19 (10%)	0
Nausea		17 (9%)	0	43 (23%)	0	18 (23%)	0	19 (45%)	2 (5%)	27 (14%)	0
Vomiting		23 (13%)	1 (1%)	60 (33%)	2 (1%)	27 (35%)	1 (1%)	25 (60%)	2 (5%)	25 (13%)	1 (1%)
Rash		14 (8%)	0	15 (8%)	0	10 (13%)	0	8 (19%)	0	8 (4%)	0
Pruritus		23 (13%)	0	16 (9%)	0	12 (15%)	0	1 (2%)	0	18 (10%)	0
Arthralgia		36 (20%)	0	20 (11%)	0	5 (6%)	0	10 (24%)	0	36 (19%)	0

Data are n (%). The table shows the number of participants with at least one incident adverse event in each event category. Incident adverse events are events with a start date or date of increase in grade occurring in the period between the day of the baseline visit and 30 days following the day of cessation (last qualifying day) of the assigned regimen, inclusive. A qualifying day is defined as a day on which treatment was taken with at least 50% of the protocol-mandated dose of all drugs in the assigned regimen (exceptions and detailed definitions are given in the protocol and statistical analysis plan). Adverse events of special interest were prespecified. Standard tuberculosis-related symptoms (cough, haemoptysis, pleuritic chest pain, fever, night sweats, and bodyweight loss) were not considered as incident adverse events in this analysis. Events were graded with Division of AIDS Toxicity Criteria and participants classified by the highest grade observed. Events were coded with the Medical Dictionary for Regulatory Activities (MedDRA), listed by single MedDRA Preferred Term, as a prespecified combination of terms (skin hyperpigmentation or discoloration), or as a composite of terms obtained with a Standardised MedDRA Query (SMQ).

* High alanine aminotransferase (ALT), high bilirubin, and Hy's law assessed centrally from protocol-mandated laboratory tests done at scheduled visits, irrespective of clinical reporting as an adverse event; and from tests done at other visits that were noted on clinical reports for grade 3 or worse adverse events or serious adverse events (limited to events reported with a preferred term under the MedDRA hepatic disorders SMQ). Hy's law criteria (ALT >3 and bilirubin >2 times the upper limit of normal) are not classified by grade in Division of AIDS Toxicity Criteria.

† Low haemoglobin and low neutrophil count assessed centrally from protocol-mandated laboratory tests done at scheduled visits, irrespective of clinical reporting as an adverse event; or from tests done at other visits that were noted on clinical reports for grade 3 or worse adverse events or serious adverse events (limited to events reported with a MedDRA preferred term of anaemia, neutropenia or thrombocytopenia, or corresponding laboratory term).

‡ For the 15 participants in the rifampicin-clofazimine group who reported skin hyperpigmentation (n=11) or discolouration (n=4), median onset from start of treatment was 18 (range 1–43) days and median time to resolution from end of treatment was 43 (range 0–196) days; all participants completed at least 56 qualifying days of treatment.

§ The participant who had grade 3 QTc prolongation had a single reading greater than 500 ms (unconfirmed) that resolved within 4 days of stopping bedaquiline.

Among 78 participants in the rifampicin-clofazimine group, the frequency of grade 3–4, serious, and treatment-limiting adverse events was similar to that observed with standard treatment (table 3; appendix pp 43–49). Adverse events of special interest that were more common than standard treatment were hepatic events (hepatic disorders, biliary disorders, and high bilirubin; Hy's law biochemical criteria were met in two [3%] participants, one with hepatitis C virus infection), skin hyperpigmentation or discoloration (all grade 1–2; none treatment-limiting), and QTc prolongation (all grade 1–2; none treatment-limiting; table 4; appendix pp 48–51, 57, 64–67). Other adverse events that were more common than standard treatment were nausea and vomiting (treatment-limiting in three [4%] participants); but arthralgia was less common (table 4; appendix pp 48–49, 68).

Among the 42 participants in the rifapentine-linezolid group, grade 3–4 adverse events and treatment-limiting adverse events occurred at a higher frequency than with standard treatment; serious adverse events occurred at similar frequency (table 3; appendix pp 43–49). Adverse events of special interest that were more common than standard treatment were hepatic events (hepatic disorders, biliary disorders, and high bilirubin) and haematological events (low haemoglobin); table 4; appendix pp 48–51, 64). Other events that were more common than with standard treatment were nausea and vomiting, which alone or together were treatment-limiting in eight (19%) participants (table 4; appendix pp 48–49, 68).

Among 189 participants in the bedaquiline-linezolid group, the frequency of grade 3–4, serious, and treatment-limiting adverse events was similar to that observed with standard treatment (table 3; appendix pp 43–49). The only adverse event of special interest that was more common than with standard treatment was low haemoglobin (grade 3–4 in 14 [7%] participants and treatment-limiting in four [2%]; table 4; appendix p 51); no other adverse events were more common than with standard treatment.

## Discussion

We found, as expected, that four novel 8-week, five-drug regimens, modified from standard treatment, all had lower efficacy than the standard 24-week treatment regimen. These findings are consistent with the finding of non-inferior efficacy for the analysis of the TRUNCATE management strategy (with the initial 8-week bedaquiline-linezolid regimen), because the strategy included monitoring for and re-treatment of relapses, and evaluated the primary clinical outcome at week 96.^5^

The poor efficacy of the 8-week rifampicin-linezolid regimen (21·0% risk difference *vs* standard treatment) might partly be due to reduced linezolid exposure from a known drug–drug interaction with rifampicin^12^ and reduced pyrazinamide exposure from a possible interaction with rifampicin.^13^, ^14^ Rifampicin dose reduction to 20 mg/kg might also have blunted efficacy, although it was still relatively poor in those taking the 35 mg/kg dose. Ongoing pharmacokinetic–pharmacodynamic analysis could add further insights. Although 8-week regimens were not directly compared, the lower risk difference versus standard treatment (9·3%) observed with the bedaquiline-linezolid regimen compared with the rifampicin-linezolid regimen is remarkable, given that these regimens are identical apart from the bedaquiline-rifampicin substitution. This might suggest greater potency of bedaquiline (enhanced by synergy with pyrazinamide),^15^, ^16^ but could also reflect a possible reduction in linezolid and pyrazinamide exposure in the rifampicin-linezolid group, as noted above. The long half-life of bedaquiline might enhance the efficacy of a short regimen by extending activity beyond the end of treatment,^17^, ^18^ or by sustaining efficacy during short periods of treatment interruption (similarly for clofazimine). Acquired bedaquiline resistance in 1% of participants is an important consideration, but it is less frequent than what is generally observed for drug-resistant tuberculosis treatment in programmes,^19^, ^20^ and within tolerable limits for a new shorter regimen for rifampicin-susceptible tuberculosis.^1^ Alternative companion drugs or bedaquiline dosing strategies might further mitigate this risk.

As this trial presents the only available contemporary trial data for 8-week regimens, it is important to explore—to the extent possible—future prospects for achieving a successful regimen of this duration. We prespecified a probability exceeding 0·95 for a difference from standard treatment of less than 12% as being a target indicating non-inferiority. In the context of this exploratory analysis this was not intended to be definitive, but rather a guide as to whether a regimen might have potential for success in a future phase 3 trial seeking to demonstrate definitive non-inferiority. The probability of 0·84 observed with the bedaquiline-linezolid regimen is substantially higher than the negligible probability observed with the rifampicin-linezolid regimen (which differs by only one drug). This suggests that the additional probability increment required to reach the 0·95 target might ultimately be achievable with an 8-week regimen that includes new drugs of transformative sterilising potential, as envisaged in the WHO-recommended 2-month optimal target duration for new drug regimens.^1^ Probabilities will depend on the threshold for maximum difference (but 12% has good justification, as described above)^8^, ^9^ and population disease severity, but alternative parameters can be considered in regimen development deliberations. Additional probability data from future early-phase trials of 8-week regimens could strengthen this framework for predicting success. The probability of 0·97 observed in participants with a very low or low bacillary burden on Xpert MTB/RIF test—demonstrated with the bedaquiline-linezolid regimen but possibly achievable by other modified standard regimens—provides a rationale for conducting definitive trials of stratified treatment with 8-week regimens in such low-risk groups. Use of such regimens within the TRUNCATE management strategy would provide additional reliability of this approach and could allow extension to those with a medium bacillary burden.^5^

Safety and tolerability are also essential considerations for evaluating novel drug regimens.^1^ The two high-dose rifampicin regimens had similar overall rates of severe and serious adverse events to standard treatment, consistent with previous high-dose rifampicin trials.^4^, ^21^, ^22^, ^23^ We did not find evidence of the excess severe hepatotoxicity observed (albeit rarely) in some trials,^4^, ^22^ although we did not enrol participants with known alcohol or drug abuse, or known hepatitis B or C infection, thus minimising background risk. Nausea and vomiting were common, especially with 35 mg/kg rifampicin, which has not been specifically reported previously, although poor tolerability to doses of 30–35 mg/kg rifampicin, with more frequent interruptions or discontinuations, was noted in trials enrolling a high proportion of participants from Asia.^22^, ^24^ Poor tolerability, with a high prevalence of gastrointestinal symptoms, was found in one study in Africa, but only with a rifampicin dose of 50 mg/kg.^25^ Genetic polymorphisms affecting rifamycin exposure might underlie potential differences in dose tolerance between people of different ethnic backgrounds.^26^, ^27^ Although gastrointestinal symptoms were rarely treatment-limiting in our trial, they could drive non-adherence and loss to follow-up in programme settings where the levels of adherence support, monitoring, and individualised management of side-effects possible within a trial are not always feasible.^28^ Overall, our findings suggest that 20 mg/kg rifampicin is better tolerated than 35 mg/kg; additional pharmacokinetic–pharmacodynamic analyses are ongoing. The high frequency of gastrointestinal adverse events and treatment discontinuation on the rifapentine-linezolid regimen contrasts with findings from the definitive trial of a similar rifapentine-moxifloxacin regimen,^3^ but are consistent with the high discontinuation rates, often due to vomiting, observed in a programme setting.^29^

The bedaquiline-linezolid regimen had the best safety profile, with few hepatic and gastrointestinal events that might decrease tolerability. Severe anaemia was uncommon and can be detected by monitoring and managed with dose reduction or cessation of linezolid, the main drug causing this side-effect. Oxazolidinones with reduced haematological toxicity might eventually replace linezolid and could further increase the feasibility of using this drug class in regimens for drug-susceptible tuberculosis.^30^ Although many factors are relevant, our efficacy and safety findings support consideration of replacing rifampicin with bedaquiline as the anchor drug for drug-susceptible tuberculosis regimens.

This trial has several limitations. The treatment was open label, but this was the only feasible approach given the number of drugs and regimens tested. The risk of bias in ascertainment of regimen efficacy outcomes is small because endpoints were bacteriologically confirmed and isolates sequenced to confirm relapse; the high retention and relatively long follow-up also provides assurance of complete outcome ascertainment. The use of standard symptom checklists, protocol-mandated laboratory safety monitoring, and adverse event grading systems mitigate the risk of bias in safety evaluations. The sample size was relatively small, limiting precision on some estimates, especially in the two groups that discontinued enrolment early. However, the differences seen in pairwise comparisons with standard treatment were mostly substantial and the interpretations clear. Participants were enrolled across diverse clinical sites, mainly in Asia; the minority representation of participants from Africa could affect the generalisability of findings, although subgroup estimates of efficacy were broadly similar by country. Participants with stable, treated HIV infection were eligible to participate in the later stages of the trial, but none were enrolled.

In summary, all 8-week regimens tested had reduced efficacy compared with the 24-week standard regimen. Even the best 8-week bedaquiline-linezolid regimen should only be used as part of the TRUNCATE management strategy within which it was previously shown to be effective in this trial. This 8-week regimen might be successful in people with low bacillary burden and could be tested in a definitive trial. Future trials should report data on low-grade adverse events, such as gastrointestinal toxicity, that are common and could impair the effectiveness of short regimens when ultimately deployed.

### Contributors

### Data sharing

Anonymised individual participant data and study documents can be requested from the corresponding author and will be made available from 6 months after publication of this paper, subject to approval of the trial steering committee.

## Declaration of interests

NIP reports grants paid to their institution, donation of drugs to their institution for the work reported in this manuscript and for work outside that reported in this manuscript, and personal fees for speaking at symposia from Janssen. AA reports grants paid to their institution outside the work reported in this manuscript and travel support from Gilead Sciences. AJN reports grants paid to their institution outside the work reported in this manuscript from Janssen. All other authors declare no competing interests.
