# Correction to *Lancet Infect Dis* 2025; 25: 1084–96

**DOI:** 10.1016/S1473-3099(25)00719-4

**Published:** 2026-01

*Paton NI, Cousins C, Sari IP, et al. Efficacy and safety of 8-week regimens for the treatment of rifampicin-susceptible pulmonary tuberculosis (TRUNCATE-TB): a prespecified exploratory analysis of a multi-arm, multi-stage, open-label, randomised controlled trial.* Lancet Infect Dis *2025;* 25: *1084–96*—In this Article, Professor Erlina Burhan's affiliation should have been as follows: “Faculty of Medicine, Universitas Indonesia and Persahabatan Hospital, Jakarta, Indonesia ”. This correction has been made to the online version as of Nov 17, 2025.

