# Supplementary material for: Efficacy and safety of 8-week regimens for the treatment of rifampicin-susceptible pulmonary tuberculosis (TRUNCATE-TB): a prespecified exploratory analysis of a multi-arm, multi-stage, open-label, randomised controlled trial
Source: Lancet Infect Dis. 2025 Oct;25(10):1084–96. doi: 10.1016/S1473-3099(25)00151-3 (PMC12460212; doi:10.1016/S1473-3099(25)00151-3)
Supplement: Supplementary appendix [file mmc1.pdf]

THE LANCET
Infectious Diseases

Supplementary appendix

This appendix formed part of the original submission and has been peer reviewed. We post it as supplied by the authors.

Supplement to: Paton NI, Cousins C, Sari IP, et al. Efficacy and safety of 8-week regimens for the treatment of rifampicin-susceptible pulmonary tuberculosis (TRUNCATE-TB): a prespecified exploratory analysis of a multi-arm, multi-stage, open-label, randomised controlled trial. *Lancet Infect Dis* 2025; published online May 22. https://doi.org/10.1016/S1473-3099(25)00151-3.

Supplementary Appendix

Supplement to: Paton NI, Cousins C, Sari IP, et al. Efficacy and safety of eight-week regimens for treatment of rifampicin-susceptible pulmonary tuberculosis: a multi-arm, multi-stage, open-label, randomised controlled trial

This appendix has been provided by the authors to give readers additional information about the work.

TABLE OF CONTENTS

Members of the TRUNCATE-TB trial team 4-6

Supplementary methods

Methods S1 Complete eligibility criteria 7-8

Methods S2 Randomisation strata 9

Methods S3 Treatment regimens 9-10

Methods S4 Principles used in constructing the 8-week treatment regimens 11

Methods S5 Disease activity clinical management criteria 12

Methods S6 Trial schedule 13-14

Methods S7 Definition of drug resistance 15

Methods S8 Comparison of main efficacy outcome definitions for analysis of drug regimen efficacy and analysis of TRUNCATE management strategy efficacy 16-19

Methods S9 Comparison of aims and all outcomes for analysis of drug regimens and analysis of TRUNCATE management strategy....................20-22

Methods S10 Approach to the analysis of drug regimen efficacy and rationale 23-27

Methods S11 Precision of estimates for analysis of drug regimen efficacy....................28-29

Methods S12 Interim analyses and selection of arms for discontinuation of enrollment 30

Supplementary tables

Table S1 Reasons for non-eligibility....................31

Table S2 Treatment duration and reasons for extension or switch 32-33

Table S3 Treatment adherence 34-35

Table S4 Comparison of classification of outcome events for drug regimen and TRUNCATE management strategy efficacy analyses 36

Table S5 Primary outcome sensitivity analyses in rifampicin-linezolid and bedaquiline-linezolid groups 37-38

Table S6 Primary outcome efficacy analysis by assigned rifampicin dose in the rifampicin-linezolid group 39-40

Table S7 Primary outcome efficacy analysis in models based on participants undergoing contemporary randomisation....................41

Table S8 Unconfirmed acquired drug resistance....................42

Table S9 Grade 3-4 adverse events....................43-45

Table S10 Serious adverse events 46-47

Table S11 Treatment-limiting adverse events 48-49

Table S12 High ALT and high bilirubin (laboratory reports)………………………………………………………..50

Table S13 Low haemoglobin, neutrophil count and platelet count (laboratory reports)……………..51

Table S14 Grade 3-4, serious and treatment-limiting adverse events
by assigned rifampicin dose in the rifampicin-linezolid group…………………………………….52

Table S15 Adverse events of special interest and common adverse events
by assigned rifampicin dose in the rifampicin-linezolid group………………………………..53-54

Table S16 High ALT and high bilirubin (laboratory reports)
by assigned rifampicin dose in the rifampicin-linezolid group……………………………………..55

Table S17 Low haemoglobin, neutrophil count and platelet count (laboratory reports)
by assigned rifampicin dose in the rifampicin-linezolid group……………………………………..56

Table S18 QTcF prolongation……………………………………………………………………………..……………………..57

Supplementary figures

Figure S1 Trial design ………. 58

Figure S2 Relationship between main outcome classification in drug regimen analysis and
TRUNCATE management strategy analysis …………………………………………………………………….59

Figure S3 Time to unfavourable outcome ………………………………………………………………………….. 60-61

Figure S4 Time to treatment failure or relapse 62-63

Figure S5 Evaluation of drug-induced serious hepatotoxicity (eDISH) …………………………………………..64

Figure S6 Participants meeting Hy's law biochemical criteria ………………………………………………65-67

Figure S7 Prevalence of nausea and vomiting on treatment ………………………………………………………68

Figure S8 Prevalence of nausea and vomiting on treatment
by assigned rifampicin dose in the rifampicin-linezolid group………………………………………69

Protocol ……70-239

Statistical analysis plan ……………………………………………………………………………………………240-385

MEMBERS OF THE TRUNCATE-TB TRIAL TEAM

Participating Sites and central laboratories

Indonesia:
Rumah Sakit Umum Pusat (RSUP) Persahabatan, Jakarta (72): Erlina Burhan, Fathiyah Isbaniah, Ibrahim N.I.P. Dharmawan, Assica P.A. Hakiman, Hera Afidjati, Diadikma Belarosa, Aga Krisnanda, Nadia U.A. Hadi, Jihaan Hafirain, Dedy Aditia

Rumah Sakit Umum Daerah Dr. Soetomo, Surabaya (62): Tutik Kusmiati, Soedarsono Soedarsono, Deby Kusumaningrum, Ridwan Yasin, Nur S.R. Panenggak, Randy D. Kurniawan, Sri Rejeki, Novi Aryanti

Universitas Padjadjaran, Bandung (53): Rovina Ruslami, Prayudi Santoso, Alamanda Larasmanah, Naufal Ihsan, Yuanita Gunawan, Sheila Sumargo, Vycke Yunivita

RSUP Dr *Wahidin* Sudirohusodo, Makassar (52)*:* Irawaty Djaharuddin, Eliana Muis, Nurjannah Lihawa, Nasrum Massi, Yusfiana Majid, A. Siti Kahfiah Mukhlis, Imam Nurjaya, Siti Arifah Lacante

RSUD Saiful Anwar, Malang (50): Jani J.R. Sugiri**,** Gede Sasmika Suwandi**,** Kristo Kurniawan**,** Santony Santony**,** Herman Liem**,** Tiar Oktavian Effendi**,** Maria Kristiani, **,** Ni Made Rini

Rumah Sakit Umum Daerah (RSUD) dr. Moewardi, Solo (5): Jatu Aphridasari, Sandy Kurniawan, Dewi Astarini, Linda Soebroto

Balai Besar Laboratorium Kesehatan (BBLK) Surabaya (central laboratory): Titiek Sulistyowati;
BBLK Makassar (central laboratory): Yoeke Rasita;
FKUI Jakarta (central laboratory): Andriansjah Rukmana

Thailand:
Central Chest Institute of Thailand (28): Nonthaburi: Piamlarp Sangsayunh, Thanyanuch Sanchat, Phornchai Pingsusaen, Krisana Cheewakul, Sasithorn Bureechai, Waraporn Thuansuwan, Jirakan Boonyasopun, Karntheera Sangkaew, Sirijit Rattanawai

Chulalongkorn University, Bangkok (17): Anchalee Avihingsanon, Sivaporn Gatechompol, Hay Mar Su Lwin, Win Min Han, Thornthun Ureaphongsukkit, Prachya Chaiyahong, Pornmalai Suriya, Sasiwimol Ubolyam, Anuntaya Uanithirat, Apicha Mahanontharit, Plengsri Lertarrom

Taksin Hospital, Bangkok (4)*:* Supunnee Jirajariyavej, Stanrat Kanokdeeseerat, Kanyapat Wongwutcharajirakul

Philippines:
De La Salle Medical and Health Sciences Institute, Cavite (72): Victoria B. Dalay, Maria Marissa I. Golla, Emmanuel A. Gutierrez, Marietto L. Partosa, Genevieve V. Bayas (deceased), Cynthia G. Wagayen, Darecil B. Gelina, Eleonor S. Garcia, Angelita G. Pabruada, Laarean R. Perlas

Lung Center of the Philippines, Quezon City (58): Vincent M. Balanag, Jr., Nerissa A. Donato, Krizia Chloe R. Rivera, Paula Cindy M Villajuan, Zyra Zafe Del Rosario

Tropical Disease Foundation, Inc., Makati (42): Thelma E. Tupasi (deceased); Rholine Gem Martin Sindingan Veto, Maria Begonia Rejaba Baliwagan, Glenn Ibana Balane, Anthony A. Geronimo, Elsie Marie B. Dela Cruz

Perpetual Succour Hospital, Cebu (39): Anabella M. Guardiario, Maria Philina P. Villamor, Ma. Bernardita Sarcauga Chua, Peter Dela Torre Blanco, Rose Marie L. Cagwin, Karenza Antipuesto Camus

Philippine Tuberculosis Society Inc. - Quezon Institute, Quezon City (27): Jubert P. Benedicto, Ma. Kriselda Karlene G. Tan, Michelle B. Recana-Nieva, Rose Ann A. Espiritu-Villasfer

India:
National Institute of TB and Respiratory Diseases, New Delhi (14): Rohit Sarin, Jitendra Kumar Saini, Prabhpreet Sethi, Mohit Tomar, Manpreet Bhalla, Shivani, Arti, Shyam Singh Bisht

Uganda:
Infectious Diseases Institute, Kampala (41): Christine Sekaggya-Wiltshire, Ruth Mirembe Nabisere, Brian Otaalo, Jesca Asienzo, Letisha Najjemba, Juliet Nampala, Lucy Alinaitwe, Eunice Kaguiri

Joint Clinical Research Centre, Kampala (29): Cissy Kityo, Henry Mugerwa, Timothy Arthur Serumaga, Timothy Masaba, Theresa Najjuuko, Joseph Akol, Caroline Kayiza

Joint Clinical Research Centre, Mbarara (10): Abbas Lugemwa, Sharif Musumba, Ibrahim Yawe, Assumpta Katusiime, Beatrice Tumusiime, Mariam Kasozi, Myalo Sula, Rogers Ankunda

Trial coordination:
Yong Loo Lin School of Medicine, National University of Singapore: Nicholas Paton, Christopher Cousins, Celina Suresh, Nan Kai Ng, Elena Wan Yi Lur, Shariba Munawara, Felic Fanusi, Gail Cross, Anushia Panchalingham, Gianna Yau, Padmasayee Papineni, Kristina Rutkute, Meera Gurumurthy, Pauline Yoong

National University Hospital: Ka Lip Chew , Intan Permata Sari

Singapore Clinical Research Institute (SCRI): Qing Shu Lu, Shu Ling Lee, Mihir Gandhi, Yogesh Pokharkar, Rajesh Babu Moorakonda, Yin Bun Cheung

MRC Clinical Trials Unit at UCL: Angela Crook, Karen Sanders, Patrick Phillips, Andrew Nunn

London School of Hygiene and Tropical Medicine: Jody Phelan, Martin Hibberd

PRODIA, Indonesia: Catharina Aprillia

Syneos *Health* Indonesia: Amalia Rachmawati

Syneos Health Philippines: Larra Minnellie M. Esconde, Bianca Austria

HIV-NAT, Thailand: Kanitta Pussadee, Hathairat Prushyapornsri, Pornkhaun Mungklang, Chanapha Janpanich, Suzan Wilmott

JSS Medical Research, India: Taran Bedi

Trial Governance:
Trial Management Group: Nicholas Paton, Christopher Cousins, Celina Suresh, Padmasayee Papineni, Ibrahim Abubakar, Karen Sanders, Angela Crook, Andrew Nunn

Trial Steering Committee (TSC): Independent members: Geraint Davies (Chair), Charles Gilks, Sushil Pandey, Abdul Razak Bin Abdul Muttalif, Basanta Kumar Parajuli, Kaewta Sangsuk; Trial related members: Nicholas Paton, Erlina Burhan, Vincent Balanag, Anchalee Avihingsanon, Christine Sekaggya-Wiltshire, Rohit Sarin; Non-voting members: Angela Crook, Andrew Nunn

Independent Data Monitoring Committee (IDMC): Guy Thwaites (Chair), Matthew Law, Janice Caoili, Reinout Van Crevel

SUPPLEMENTARY METHODS

Methods S1 COMPLETE ELIGIBILITY CRITERIA

Inclusion criteria

1. Age 18 to 65 years
2. Clinical symptoms consistent with pulmonary TB and/or evidence of pulmonary TB on CXR
3. Sputum GeneXpert test positive ¶
4. Willing to comply with the study visits and procedures
5. Resident at a fixed address that is readily accessible for visiting, within feasible travelling distance to the site and likely to remain resident there for the duration of trial follow-up
6. Willing to have directly observed therapy
7. Willing and able to provide written informed consent

Exclusion criteria

1. Taken more than 10 daily doses of standard anti-TB medication or fluoroquinolones during the 3 months prior to randomisation
2. Previous active TB disease for which treatment was given prior to the current episode (patients who have received isoniazid prophylaxis are eligible)
3. Known or suspected extra-pulmonary TB (in the opinion of the investigator; no specific screening tests required; if symptoms suggest extra-pulmonary involvement, patient can still be enrolled if relevant tests-performed for clinical management-exclude extra-pulmonary disease at the suspected site; pleural effusion occupying <50% of hemithorax or concomitant intra- or extra-thoracic lymphadenopathy are not exclusions)
4. Severe clinical pulmonary TB e.g. respiratory failure or complications likely to require hospital admission
5. Sputum smear 3+ on sample taken at screening (using WHO/IUATLD grading system)*
6. Cavity size > 4cm on screening CXR*
7. Presence of rifampicin resistance on GeneXpert test
8. Poorly-controlled diabetes that, in the opinion of the investigator, is unlikely to be controlled with available management strategies
9. Active malignancy requiring systemic chemotherapy or radiotherapy
10. Known Hepatitis B surface antigen positive and/or HCV antibody positive, unless liver function tests consistently within normal range for at least 2 years
11. History of myocardial infarction, congestive cardiac failure, cardiac arrhythmias or any known congenital cardiac problems
12. History of severe chronic lung disease (e.g. chronic obstructive pulmonary disease) with symptom score of ≥3 on MRC breathlessness scale
13. History of seizures*
14. Current tendinitis or history of tendinopathy associated with fluoroquinolone use*
15. Symptomatic peripheral neuropathy causing greater than minimal interference with usual social and functional activities.
16. Current alcohol or drug abuse
17. Women who are currently pregnant or breast-feeding
18. Women of childbearing potential unwilling or unable to use appropriate effective contraception (i.e. barrier methods such as condoms, hormonal contraception, intra-uterine device) for the first 6 months of the trial
19. Known allergy to one or more of the study drugs
20. Taking a concomitant medication that has a known or predicted interaction with any of the study drugs to which the patient might be randomised, or is known to prolong the QTc interval

(if the concomitant medication can be stopped after randomisation, or there is a feasible alternative medication available, the patient need not be excluded)

21. Taking any immunosuppressive drugs or use of systemic corticosteroids for more than 2 weeks prior to screening
22. Colour blindness detected by Ishihara test
23. 12-lead ECG at screening shows QTc greater than 450ms and/or any other clinically-significant abnormality such as arrhythmia or ischaemia
24. Any of the following laboratory parameters at screening:
    - Absolute neutrophil <1000 cells/µL, haemoglobin <7.0 g/dL, OR platelet count <50,000 cells/mm^3
    - Creatinine clearance of <60ml/min (calculated using Cockcroft-Gault equation,
    - ALT greater than 3 times the upper limit of normal
    - Uncorrected serum potassium <3.5 mmol/L
25. HIV antibody positive at screening*
26. Any other significant condition (e.g. psychiatric illness, chronic diarrhoeal disease), that would, in the opinion of the investigator, compromise the patient's safety or outcome in the trial or lead to poor compliance with study visits and protocol requirements
27. Participation in other clinical intervention trial or research protocol (participation in other studies that do not involve an intervention may be allowed, but this must be discussed and approved by the Chief Investigator)

*Criteria marked with an asterisk (5,6,13, 14) were removed in version 2 of the protocol.

Criterion 25 (HIV antibody positive), was modified in version 2 of the protocol as follows:
HIV antibody positive, unless **all** the following additional criteria are met:

(i) Current CD4 T-cell count >200cells/mm^3
(ii) Not currently taking ART
(iii) Patient and doctor willing to defer starting ART until 8 weeks after enrolment (up to 12 weeks, if randomised to an 8-week regimen the duration of which is extended)
(iv) have access to ART and are willing to start when recommended by local treatment guidelines, at any point from 8 weeks (or 12 weeks, as above) after enrolment

Version 2 of the protocol was implemented, with removal of eligibility restrictions above, following approval of ethics committees and national regulatory agencies at 11 sites in Indonesia and Philippines. Approvals were received between December 2019 and February 2020, a mean of 9 weeks prior to end of trial enrollment.

¶ GeneXpert test: results of either Xpert MTB/RIF or Xpert Ultra were acceptable.

Methods S2   RANDOMISATION STRATA

Randomisation was stratified by the following factors:

- Site
- Relapse risk
    - Lower: sputum screening smear negative and screening CXR cavities ≤4 cm and HIV negative
    - Intermediate: screening sputum smear positive and screening CXR cavities ≤4 cm and HIV negative
    - Higher: screening sputum smear grade 3+ or screening CXR cavities >4 cm or HIV positive

The threshold of 4cm for cavitation on chest radiograph was based on studies of exposure-response relationships in rifapentine Phase II trials – Savic, Clin Pharmacol Ther 2017; 102(2):321-331.

Participants unable to produce sputum at screening were regarded as smear negative for the purpose of classifying relapse risk.

Methods S3 TREATMENT REGIMENS

A. Standard treatment regimen

For first 8 weeks

DRUG	<40KG	40-54 KG	55-70KG	≥71KG
Rifampicin	300mg	450mg	600mg	750mg
Isoniazid	150mg	225mg	300mg	375mg
Pyrazinamide	800mg	1200mg	1600mg	2000mg
Ethambutol	550mg	825mg	1100mg	1375mg

For subsequent weeks to week 24

DRUG	<40KG	40-54 KG	55-70KG	≥71KG
Rifampicin	300mg	450mg	600mg	750mg
Isoniazid	150mg	225mg	300mg	375mg

B. Rifampicin-linezolid regimen
For 8 weeks

DRUG	<40KG	40KG- 54KG	55KG - 70KG	≥71KG
Rifampicin	35mg/kg (rounded to nearest 150mg, maximum 2100mg)*			
Isoniazid	150mg	225mg	300mg	375mg
Pyrazinamide	800mg	1200mg	1600mg	2000mg
Ethambutol	550mg	825mg	1100mg	1375mg
Linezolid	600mg			

*The dose of rifampicin was decreased from 35mg/kg to 20mg/kg for all participants from 1st November 2019 after 88 participants had been enrolled in this arm (recommendation of the Trial Steering Committee as a precaution following a death from acute hepatic failure in this group). The

dose of 20mg/kg was chosen because there was evidence from phase 2B trials (in particular the HIRIF trial, Velasquez et al, Am J Resp Crit Care Med 2018) that this dose had higher efficacy than 10mg/kg or 15mg/kg doses; and there was a reasonable body of clinical data indicating safety of this dose.

C. Rifampicin-clofazimine regimen

For 8 weeks

DRUG	<40KG	40KG- 54KG	55KG - 70KG	≥71KG
Rifampicin	35mg/kg (rounded to nearest 150mg, maximum 2100mg)			
Isoniazid	150mg	225mg	300mg	375mg
Pyrazinamide	800mg	1200mg	1600mg	2000mg
Ethambutol	550mg	825mg	1100mg	1375mg
Clofazimine	200mg			

D. Rifapentine-linezolid regimen

For 8 weeks

DRUG	<40KG	40KG- 54KG	55KG - 70KG	≥71KG
Isoniazid	5mg/kg rounded to nearest 100mg		300mg	
Pyrazinamide	25mg/kg rounded to nearest 500mg	1000mg	1500mg	2000mg
Rifapentine	1200mg			
Linezolid	600mg			
Levofloxacin	1000mg			

E. Bedaquiline-linezolid regimen

For 8 weeks

DRUG	<40KG	40KG- 54KG	55KG - 70KG	≥71KG
Bedaquiline	400 mg once daily for 2 weeks then 200mg three times a week			
Isoniazid	5mg/kg rounded to nearest 100mg		300mg	
Pyrazinamide	25mg/kg rounded to nearest 500mg	1000mg	1500mg	2000mg
Ethambutol	15mg/kg (rounded to nearest 100mg, maximum 1600mg)			
Linezolid	600mg			

Methods S4 PRINCIPLES USED IN CONSTRUCTING THE 8-WEEK TREATMENT REGIMENS

The treatment regimens used for the initial 8-week treatment were selected based on consideration of potential maximal sterilising efficacy, absence of known pharmacokinetic drug interactions, as well as likely tolerability and safety.

In general, we started from the standard treatment regimen, made 2-3 modifications predicted to enhance sterilising activity and kept 5 drugs in each boosted regimen (in contrast to the 4 drugs in the standard regimen). The experimental arms retain, wherever possible, the two key sterilising drugs of the standard-of-care regimen: rifampicin and pyrazinamide. We also kept isoniazid which, although it is not traditionally considered to have much sterilising activity, does have potent early bactericidal activity that may help to diminish the chances of early selection of resistance. The benefit of ethambutol in these regimens is uncertain, but we retained it where it allowed the use of fixed-dose combinations and there is a net decrease in the pill burden. We have also retained it in the bedaquiline arm so that all the 8-week regimens have 5 drugs (instead of the usual 4-drug therapy).

For the modifications intended to increase sterilising activity, we considered adding drugs for which there was human randomised controlled trial data suggesting they possess potent sterilising activity. We examined each of the potential modifications from safety, tolerability and PK perspectives to ensure that there would be unlikely to be major overlapping toxicity or clinically-significant PK interactions. We also examined data from animal models and clinical studies where individual drugs had been combined to ensure that there was no evidence of antagonism, and preferably evidence of synergy to support a particular choice of combination. We also solicited the opinions of independent experts with particular clinical and non-clinical knowledge of individual drugs and took this expert opinion into account in the final choice of boosted regimens.

Further details are provided on the rationale for selecting an 8-week regimen duration and for the selection of drugs and doses for the regimens is provided in the trial protocol sections 2.4 and 2.5.

Methods S5 DISEASE ACTIVITY CLINICAL MANAGEMENT CRITERIA

These criteria were used for real-time diagnosis of clinical relapse and re-treatment decisions

Participants were considered to have met criteria for clinical disease activity (clinical relapse) if at least 2 out of criteria A or B or C are satisfied, or if criterion D is satisfied. The criteria needed to be satisfied simultaneously and be based on recent imaging and microbiological investigations.

A. Clinical Disease Progression

New, recurrent or increased severity of one or more standard TB-related symptoms (cough, one or more episodes of haemoptysis, fever, pleuritic chest pain, weight loss, night sweats) or physical signs compared with the end of the last course of TB treatment (or period after the end of treatment if further improvement occurred subsequently) without alternative explanation(s) considered more likely in the opinion of the managing clinician.

B. CXR Progression

Presence of abnormalities that were compatible with active TB disease (cavitation, infiltrates, consolidation) with clear evidence of progression compared with CXR at end of the last course of treatment (or period after the end of treatment if further improvement occurred subsequently) without alternative explanation(s) considered more likely in the opinion of the managing clinician.

C. Microbiological persistence / progression

Sputum sample taken at or after the end of treatment that was:
Smear positive OR
GeneXpert positive (if >24w after end of treatment; or if end of treatment test was negative) OR
Culture positive

D. Confirmed positive sputum culture

Sputum culture positive on two consecutive samples taken on separate days with at least one taken ≥ 4 weeks after end of treatment**.**

<u>Extra-pulmonary TB disease activity</u>

The above criteria applied to relapse from pulmonary TB. The definition of relapse could also be met if the patient fulfilled equivalent criteria that indicate disease relapse at another site i.e. non-pulmonary symptoms or signs, evidence of abnormalities on another imaging test that were compatible with active TB disease with evidence of progression from an earlier comparable imaging test (if no previous test available for determining progression, the abnormalities should be of sufficient severity to explain the symptoms) and microbiological evidence (culture evidence, preferred). Histological evidence considered to show characteristic changes of TB would also be acceptable under criterion C. However, the evidence from A, B and C (or the two cultures for D) should relate to the same disease site.

Methods S6 TRIAL SCHEDULE

VISIT TIMING[1]	SCREENING	D0	W1	W2	W4	W6	W8	W10	W12	W16	W20	W24	W36	W48	W60	W72	W84	W96
Informed Consent	X																	
Eligibility criteria	X	X																
Randomisation		X																
CLINICAL EVALUATION																		
Medical history & demographics	X	X																
Symptoms	X	X	X	X	X	X	X	X	X	X	X	X	X	X	X	X	X	X
Physical examination	X	X	X	X	X	X	X	X	X	X	X	X	X	X	X	X	X	X
Medication review and adherence	X	X	X	X	X	X	X	X	X	X	X	X	X	X	X	X	X	X
HEALTHCARE UTILISATION & QOL																		
Healthcare utilisation		X	X	X	X	X	X	X	X	X	X	X	X	X	X	X	X	X
EQ-5D		X	X	X	X	X	X	X	X	X	X	X	X	X	X	X	X	X
MOS-HIV		X																X
Patient acceptability questionnaire														X				X
Socioeconomic evaluation		X																X
INVESTIGATIONS																		
ECG[2]	X	X	X		X		X											
CXR[3]	X	X					X											X
Spirometry							X							X				X
URINE																		
Pregnancy test	X				X		X											
Urine for storage[4]		X			X		X					X						
SPUTUM																		
Smear[5]	X	X	X	X	X	X	X	X	X	X	X	X	X	X	X	X	X	X
Liquid culture[6]		X	X	X	X	X	X	X	X	X	X	X	X	X	X	X	X	X
GeneXpert test[7]	X						X											
Drug susceptibility tests[8]		X					X											
BLOOD																		
Standard safety monitoring[9]	X	X	X	X[10]	X	X[10]	X	X[10]										
HIV test[11] (and CD4 count)[12]	X																	
Drug levels (PK)[13]		X			X		X					X						
Plasma and RNA storage[14]		X			X		X					X						

1. Additional telephone visits at weeks 30, 40, 44, 52, 56, 64, 68, 76, 80, 88, 92 (omitted if attended scheduled visit within previous 7 days) to evaluate symptoms, medication and adherence. A telephone visit will be performed at the time of trial closure. Visit windows: ±3 days for W1 to W4; ± 5 days for W6 and W8; ± 7 days for W10 to W24; ± 14 days for W30 onwards and ± 28 days for W96.
2. Screening ECG may be deferred to Day 0, but result must be available prior to performing randomisation. Additional ECG required at end of treatment for participants who stop assigned regimen between week 8 and 12; additional ECG required at week 12 for participants who switch assigned regimen to standard treatment between week 8 and 12. If QTc >450 ms, ECG should be repeated to confirm.
3. CXR at any visit need not be repeated if done in previous 7 days and film is available for evaluation. Additional CXR done at end of treatment and at the first visit of any suspected episode of relapse.
4. Urine (10ml) for storage. Additional sample taken for storage and for pregnancy test at end of treatment and at first suspicion of relapse (one for each episode)
5. 2 sputum smears on separate days at week 96.
6. Spot sputum sample at each visit (if available), cultured on liquid media (MGIT); 2 sputum samples on separate days at week 96.
7. GeneXpert test at screening need not be repeated if a positive result is available from a test performed earlier during this illness episode, done in study-approved laboratory and results are/will be available to research team. GeneXpert at week 8 (and end of treatment, if treatment continues after 8 weeks), and on the initial sputum sample obtained during any suspected episode of relapse.
8. Drug susceptibility at baseline, and on positive cultures at or after week 8 (monthly during any retreatment episode).
9. Blood for standard safety monitoring: FBC, electrolytes (sodium, potassium, creatinine), LFTs (ALT, alkaline phosphatase, bilirubin) done at scheduled visits and at end-of-treatment visit. Additional bloods at screening only: amylase/lipase, magnesium, calcium and glucose. Blood tests at any visit need not be repeated if results available from previous 2 days.
10. Additional FBC at weeks 2, 6, and 10 for participants receiving linezolid (Arms B, D, E only).
11. HIV antibody test need not be repeated if done in previous 30 days and results available to trial team.
12. CD4 count only if patient HIV positive and trial is open to enrolment of HIV-positive participants at that site.
13. Blood for drug levels to be taken at Day 0 (1 hour post-dose), week 4 (pre-dose and 2 hours post dose) and week 8 (pre-dose and 4 hours post-dose), and week 24 (participants randomised to bedaquiline-linezolid or rifampicin-clofazimine arms only, single sample, no timing requirements). Samples may be omitted for patient convenience or logistical reasons.
14. Blood for plasma (10ml EDTA tube) and host RNA (5ml RNA preservation tube) storage. Additional samples taken at end of treatment and at first suspicion of relapse (one for each episode).

Methods S7 DEFINITION OF DRUG RESISTANCE

The primary definition of drug resistance is based on phenotypic drug resistance testing, with whole genome sequencing (and other relevant molecular tests that were done) considered as supporting data.

Phenotypic resistance on a single isolate was ignored if the same isolate was shown to be susceptible on repeat testing; or if the isolate was followed by one or more isolates that were shown to be susceptible.

<u>Baseline drug resistance</u>

Participants were classified as having baseline drug resistance to a specific drug if there was demonstration of phenotypic resistance to that drug on the first available isolate, obtained at or before week 2 of the trial. If the first available isolate was after week two, drugs to which the isolate was shown to be susceptible were inferred as susceptible at baseline; drugs that were shown to be resistant after week two were designated as unknown susceptibility at baseline.

<u>Acquired drug resistance</u>

Participants were classified as having acquired drug resistance (confirmed) to a specific drug if there was demonstration of phenotypic resistance to that drug on at least two separate isolates during follow-up; and demonstration of phenotypic susceptibility to that drug on two separate samples collected prior to the first of the samples showing resistance; and exposure to the relevant drug (or another drug known to cause cross-resistance to that drug) prior to the collection of the first sample showing resistance.

Participants were classified as having acquired drug resistance (unconfirmed) if there was demonstration of resistance on one follow-up isolate only (without subsequent susceptible isolates); or susceptibility on one previous isolate only; or where there was no exposure to the relevant drug.

Cases of acquired drug resistance were rejected if results from molecular tests or whole genome sequencing showed resistance mutations consistent with the phenotypic drug resistance were present at baseline; or where they show that the follow-up resistant strain was not related to the earlier susceptible strain.

Additional details are provided in the statistical analysis plan.

Methods S8 COMPARISON OF MAIN EFFICACY OUTCOME DEFINITIONS FOR ANALYSIS OF DRUG REGIMEN EFFICACY AND ANALYSIS OF TRUNCATE MANAGEMENT STRATEGY EFFICACY

The primary outcome for the analysis of drug regimen efficacy is unfavourable outcome. The details of the definition of unfavourable outcome are provided in the statistical analysis plan (sections 4.11 and 4.13).

The major difference between *unfavourable outcome* (used for this regimen analysis) and *unsatisfactory outcome* (the primary outcome used in the analysis of the TRUNCATE strategy) is that treatment failure and relapse count as unfavourable outcome at any time during follow-up in the analysis of regimen efficacy (and account for most of the events that are classified as unfavourable outcome), whereas these events did not count as unsatisfactory outcome in the analysis of the TRUNCATE strategy efficacy if re-treatment was successful (as it was in most; defined as participants off treatment and free from active TB at week 96). Furthermore, failure to complete initial treatment or missing more than 7 days' initial treatment count as unassessable in the analysis of regimen efficacy but were ignored in the main analysis of the TRUNCATE strategy.

The table below summarises the algorithm for classification of unfavourable and unassessable outcome for analysis of regimen efficacy and TRUNCATE strategy efficacy.

The impact on the classification on the actual outcomes for participants in the trial is shown in Table S4 and Figure S2 in this Supplementary Appendix.

**EVENT**	**Unfavourable outcome (regimen analysis)** (time outcome assigned)	**Unsatisfactory outcome (strategy analysis)**
Initial treatment insufficient / non-adherent		
<u>8-week regimen groups only:</u><br>Took less than 54 qualifying days of assigned regimen in total *<br><br>Exception 1:<br>Death on initial treatment	Unassessable (at last qualifying day of assigned regimen)<br><br>Exception 1:<br>Unfavourable (at day of death)	Ignore
<u>Standard regimen group only:</u><br>Took less than 154 qualifying days of assigned regimen in total †<br><br>Exception 1:<br>Death on initial treatment	Unassessable (at last qualifying day of assigned regimen)<br><br>Exception 1:<br>Unfavourable (at day of death)	Ignore
<u>All groups:</u><br>Took less than 49 qualifying days of assigned regimen (as allocated by randomisation) during the first 56 days of the trial ‡	Unassessable (at 7th missed dose)	Ignore

Initial treatment switch		
<u>8-week regimen groups only:</u><br>Switched to standard regimen<br><br>Exception 1: ≤ 7 days of standard treatment followed by reintroduction of assigned regimen<br><br>Exception 2: Treatment failure at switch<br>Took ≥ 54 qualifying days of assigned regimen; latest culture positive (taken at ≥ 54 qualifying days of assigned regimen; AND at or before the last day of the assigned regimen; AND at ≤5 days before switch (single positive culture adequate; confirmation not required); AND WGS same strain, inconclusive or not done (if WGS shows different strain this exception does not apply, so classified as unassessable)	Unassessable (at last qualifying day of assigned regimen)<br><br>Exception 1: Ignore (do not censor)<br><br>Exception 2: Unfavourable (at switch) §	Ignore<br><br>Ignore<br><br>Ignore
<u>All groups:</u><br>Added new TB drugs to the assigned initial regimen<br><br>Exception 1:<br>Temporary addition (≤ 7 days of new drug)<br><br>Exception 2:<br>Addition of fluoroquinolone in the standard treatment group for documented baseline drug resistance.	Unassessable (at start of new drug)<br><br>Exception 1: Ignore (do not censor)<br><br>Exception 2: Ignore (do not censor)	Ignore<br><br>Ignore<br><br>Ignore
Treatment failure on stopping initial treatment		
Treatment failure *confirmed* or *unconfirmed* by *treatment failure analysis criteria ¶*<br>WGS same strain as baseline, inconclusive or not done<br><br>Exception 1:<br>Treatment failure *confirmed*<br>WGS shows different strain from baseline<br><br>Exception 2:<br>Treatment failure *unconfirmed*<br>WGS shows different strain from baseline	Unfavourable (at end-of-treatment culture) §<br><br>Exception 1: Unassessable (at end-of-treatment culture)<br><br>Exception 2: Ignore (do not censor)	Ignore<br><br>Ignore<br><br>Ignore
Relapse after initial treatment		
Relapse *confirmed* or *unconfirmed* by *relapse analysis criteria ***<br>WGS same strain as baseline, inconclusive or not done<br><br>Exception 1:<br>Relapse *confirmed*<br>WGS shows different strain from baseline<br><br>Exception 2:<br>Relapse *unconfirmed*<br>WGS shows different strain from baseline	Unfavourable (at first positive culture in relapse episode) §<br><br>Exception 1: Unassessable (at first positive culture in relapse episode)<br><br>Exception 2: Ignore (do not censor)	Ignore<br><br>Ignore<br><br>Ignore

Re-treatment		
Re-started treatment before week 96 (any reason apart from treatment failure/relapse/reinfection meeting criteria above)	Unassessable (at restart of treatment)	Ignore
Ongoing requirement for TB treatment at week 96		
Started treatment at or after week 72 but stopped treatment before week 96<br><br>Exception:<br>Did not meet *disease activity clinical management criteria* when started treatment	Ignore<br>[covered by criteria above]<br><br>Exception :<br>Ignore<br>[covered by criteria above]	Unsatisfactory<br><br>Exception:<br>Unassessable
On treatment at week 96	Ignore<br>[covered by criteria above]	Unsatisfactory
Ongoing TB disease activity at week 96		
Assessed by *disease activity research criteria* at week 96: †† *Definitive* (D) or *Presumed* (C + A and/or B) disease activity [WGS same strain, inconclusive or not done]<br><br>Exception:<br>WGS shows different strain from baseline	Unfavourable (at week 96)<br><br>Exception:<br>Unassessable (at week 96)	Unsatisfactory<br><br>Exception:<br>Unassessable
Assessed by *disease activity research criteria* at week 96: †† *Possible* (A+B only) disease activity<br><br>Exception:<br>At least one sputum culture (observed) negative at week 96	Unfavourable (at week 96) ‡‡<br><br>Exception:<br>Unassessable	Unsatisfactory<br><br>Exception:<br>Unassessable
Single positive culture at week 96		
Assessed by *disease activity research criteria* at week 96: ††<br>Criterion C met (A, B and D all not met)<br>WGS same strain; or WGS inconclusive or not done<br><br>Exception 1:<br>WGS shows different strain from baseline<br><br>Exception 2:<br>WGS inconclusive or not done and at least one other sputum culture (observed) negative at week 96	Unassessable (at week 96) §§<br><br>Exception 1: Ignore (do not censor)<br><br>Exception 2: Ignore (do not censor)	Unassessable<br><br>Exception 1:<br>Ignore<br><br>Exception 2:<br>Ignore
Death at or prior to week 96		
Death before close of week 96 analysis window<br><br>Exception<br>Last study visit was after week 48<br>AND<br>well, not on treatment, no evidence ongoing TB activity at last study visit [assessed by *disease activity clinical management criteria*]<br>AND<br>cause of death known to be unrelated to TB or TB drugs	Unfavourable (at day of death)<br><br>Exception<br>Unassessable (at day of death)	Unsatisfactory<br><br>Exception<br>Unassessable

Did not attend week 96 visit (and not known to have died)		
Withdrawal, lost to follow up, missed week 96 visit<br><br>Exception 1:<br>(a) Completed last prescribed course of treatment (minimum 54 days of assigned regimen, or 154 qualifying treatment days if on or switched to standard regimen)<br>AND<br>(b) Had at least 1 study visit at or after completing last course of treatment<br>AND<br>(c) Did not meet the criteria for ongoing requirement for treatment [addressed by (a), above] or ongoing TB disease activity when last seen [assessed by *disease activity clinical management criteria*; evidence of CXR progression omitted unless CXR available at the visit when last seen]<br>AND<br>(d) Last 2 visits (at or following end of treatment) had negative sputum cultures (observed or imputed based on clinical recovery)<br>AND<br>(e) EITHER patient can be contacted at week 96 and confirms not on treatment and no TB symptoms OR friend/relative can be contacted at week 96 confirming seen alive in previous 2 months, did not report TB treatment nor symptoms (or observed to have symptoms)<br>(a) to (e) all true<br>Exception 2:<br>(a) to (d) all true, no information available to judge (e)	Unfavourable (at last study visit)<br><br>Exception 1: Ignore (do not censor)<br><br>Exception 2: Unassessable (at last study visit)	Unsatisfactory<br><br>Exception 1: Ignore<br><br>Exception 2: Ignore

WGS = whole-genome sequencing

* A sensitivity analysis of the primary outcome classifies participants taking more than 56 days of the assigned regimen as unassessable (versus ignored in the main analysis).
† Duration of standard regimen includes period of standard treatment taken before randomisation
‡ Sensitivity analyses of the primary outcome classify different durations of non-adherence (>3 days missed; > 14 days missed) as unassessable.
§ A sensitivity analysis of the primary outcome classifies participants with treatment failure at switch; treatment failure at end of treatment *unconfirmed*; and relapse *unconfirmed* – all with WGS same strain, inconclusive or not done–as unassessable (versus unfavourable in the main analysis).
¶ Treatment failure analysis criteria are described in the statistical analysis plan (section 4.9.10)
** Relapse analysis criteria are described in the statistical analysis plan (section 4.9.11)
†† Disease activity research criteria are described in the statistical analysis plan (4.11.2)
‡‡ A sensitivity analysis of the primary outcome classifies participants with possible disease activity at week 96 as unassessable (versus unfavourable in the main analysis). No participants met this criterion so the sensitivity analysis was not performed.
§§ A sensitivity analysis of the primary outcome classifies participants with isolated culture positive at week 96 as unassessable (versus unfavourable in the main analysis). No participants met this criterion so the sensitivity analysis was not performed.

Methods S9 COMPARISON OF AIMS AND COMPARISON OF ALL OUTCOMES FOR ANALYSIS OF DRUG REGIMENS AND ANALYSIS OF TRUNCATE MANAGEMENT STRATEGY

Aims of analysis

	**Regimen analysis**	**Strategy analysis**
**Aims of analysis**		
Efficacy aim	Describe efficacy of novel 8-week regimens	Determine non-inferiority of the TRUNCATE strategy *
Safety aim	Describe safety of novel 8-week regimens	Determine safety of the TRUNCATE strategy *
Other aims	Nil	Determine advantages and disadvantages of the TRUNCATE strategy from the patient and programme perspective *

* TRUNCATE strategy comprises 8 weeks of initial treatment, monitoring to 96 weeks and re-treatment of relapses

Efficacy outcomes

	**Regimen analysis**	**Strategy analysis**
**Main efficacy outcome**		
Main efficacy outcome	Unfavourable outcome in ITT population, baseline to week 96 †	Unsatisfactory outcome in ITT population at week 96 † (=trial primary outcome)
**Secondary efficacy outcomes**		
Treatment failure or relapse	Baseline to week 96	Not an outcome
CXR % lung affected	Baseline to week 8 ‡	Not an outcome

† Unfavourable outcome (regimen analysis) and unsatisfactory outcome (strategy analysis) differ substantially. The two outcomes are compared in detail in the previous section

‡ CXR change in proportion lung affected to week 8 was specified as an additional efficacy outcome but is not presented in this manuscript (will be reported separately).

Safety outcomes

	Regimen analysis	Strategy analysis
**Safety outcomes**		
Grade 3-4 adverse events (reported)	Baseline to end of treatment +30 days	Baseline to week 96
Serious adverse events (reported)	Baseline to end of treatment +30 days	Baseline to week 96
Treatment-limiting adverse events (reported)	Baseline to end of treatment +30 days	Not an outcome
Adverse events of special interest:	Baseline to end of treatment + 30 days; all grades	Baseline to week 96<br>Grade 3-4 only
Hepatic	Hepatic disorders (reported events, combined by SMQ)<br>Biliary disorders (reported events, combined by SMQ)<br>Liver function tests (by central lab. test review)<br>LFT patterns (eDISH plot)<br>Hy's law (narrative reviews)	Only individual reported adverse events, limited to grade 3 and 4 and not aggregated by SMQ<br>No direct analysis of laboratory reports (only reported hepatic and haematology events)<br>No direct analysis of QTc data (only reported QTc prolongation events)
Haematological	Anaemia (individual reported events)<br>Haematopoeitic cytopenias (reported events, as SMQ)<br>Haematology tests (by central lab. test review)	
Peripheral neuropathy	Peripheral neuropathy (reported events, as SMQ)	
Optic nerve disorders	Optic nerve disorders (reported events, as SMQ)	
Skin hyperpigmentation	Skin hyperpigmentation or discoloration (reported events, combined)	
Electrocardiogram QTc prolongation	QTc prolongation (reported events, as SMQ)<br>QTc prolongation (by central QTc review)	
Adverse events in ≥10% participants	Baseline to end of treatment + 30 days ¶	Not an outcome
Self-reported nausea and vomiting	From symptom checklist to week 8 (or week 24 for standard treatment) ¶	Not an outcome
Respiratory disability (spirometry or MRC scale)	Not an outcome	At week 96

Other outcomes (participant-centred and programme-centred)

	Regimen analysis	Strategy analysis
**Participant-centred outcomes**		
Total treatment time	Not an outcome	Baseline to week 96
Acceptability of the strategy: Difficulty, Anxiety Motivation, Recommendation to others	Not an outcome	At week 48 and 96
Quality of life scores	Not an outcome	At week 96
Health status score	Not an outcome	Baseline to week 96
Illness-related missed work/study days	Not an outcome	Baseline to week 96
Body weight and BMI change from baseline	Not an outcome	Baseline to week 96
**Programme-centred outcomes**		
Treatment adherence: Over first 56 days Missing ≥ 14 days in first 56d Discontinue in first 56 days	Not an outcome	Baseline to day 56
Treatment adherence: over all treatment courses	Not an outcome	Baseline to week 96
Acquired drug resistance	Not an outcome ‖	Baseline to week 96
Relapse-associated transmission risk	Not an outcome	Baseline to week 96

¶ Adverse events occurring in ≥10% participants and self-reported nausea and vomiting (from symptom checklist) were added as a post-hoc safety outcomes for the regimen analysis

‖ Acquired drug resistance was not specified as an outcome for the regimen analysis but is reported in this manuscript for descriptive purposes.

Outcomes and analyses are pre-specified as described for the regimen and strategy analyses in the statistical analysis plan (Chapter 11 and Chapter 8 respectively).

Methods S10 APPROACH TO THE ANALYSIS OF DRUG REGIMEN EFFICACY AND RATIONALE

Aims of the drug regimen efficacy analysis

The TRUNCATE-TB trial was designed primarily to evaluate efficacy of the TRUNCATE management strategy. However, the follow-up data collected after cessation of the initial 8-week course of treatment provides a unique opportunity to assess the efficacy and safety of novel regimens of 8 weeks' duration.

The analysis is intended to be exploratory, seeking to inform future regimen development decisions, not intended as a substitute for a phase 3 trial. Prior to receiving a license, any new regimen, whether based on modified standard treatment or on regimens with completely new drugs would (based on the current model of drug development), need to undergo conventional efficacy testing in a definitive phase 3 trial enrolling large numbers of participants across all degrees of disease severity and with a representative spectrum of disease comorbidities.

The information from this analysis that may be of relevance for drug development includes:

(i) Identification of novel 8-week regimens have the best potential for achieving efficacy comparable to that of 24-week standard treatment.

(ii) Identification of which modifications to standard treatment appear to have the most impact on efficacy. The composition of several regimens differs by a single drug, which may allow inferences to be made.

(iii) Assessing the future chance of being able to achieve a regimen of 8 weeks' duration that would have comparable efficacy to 24-week standard treatment.

In addition to informing future regimen development decisions, findings from this analysis may also be useful for informing the use of 8-week regimens in treatment strategies. The information from this analysis that may be of relevance to treatment strategy applications includes:

(iv) Identification of subgroups where the excess of relapses with an 8-week regimen would be sufficiently low that the regimen could be used in that selected subgroup, either as a stratified treatment approach with expectation of relapse-free cure, or by deploying with the additional safeguards provided by the TRUNCATE strategy.

(v) Selection or revision of regimens for use as the initial 8-week treatment in the TRUNCATE management strategy (to achieve the target total relapse rate below 20%).

Explanation and justification of Bayesian approach for the regimen analysis

In this analysis of drug regimen efficacy, the main outcome parameter is unfavourable outcome. Treatment failure and relapse count as unfavourable outcome at any time during follow-up. This is the standard outcome for assessing drug regimen efficacy. In contrast, as the monitoring, detection and management of relapses are an integral part of the TRUNCATE management strategy, these events cannot logically be counted towards the primary outcome for assessing the efficacy of the strategy. For the analysis of the TRUNCATE management strategy, a different outcome was required – unsatisfactory outcome – in which treatment failure and relapse did not count as unsatisfactory outcome if re-treatment was successful (defined as participants alive, off treatment and free from active TB at week 96). Thus, these two outcomes are substantially different, appropriately so for assessing interventions that are also substantially different (8-week treatment regimen versus 96-

week TRUNCATE management strategy). A detailed comparison of unfavourable outcome with unsatisfactory outcome is provided in Methods S8, above.

For the analysis of drug regimen efficacy, it was not possible to propose a plausible non-inferiority hypothesis that could be tested with a sample size of 180 for each group (which had been set for the primary analysis of the TRUNCATE management strategy, as detailed in Methods S11 below). To do so would require an assumption that the rate of unfavourable outcome would be the same or only slightly elevated in an 8-week regimen group compared to the 24-week standard treatment group. This assumption would be inconsistent with the underlying premise of the TRUNCATE-TB trial – namely that there would be a moderate excess of unfavourable outcomes (due to treatment failure or relapse with the 8-week regimen), but that any adverse consequences of that excess could be mitigated by the monitoring and re-treatment strategy. In contrast, this sample size was considered adequate for testing a formal hypothesis of non-inferiority of the TRUNCATE strategy versus standard management on unsatisfactory outcome (the primary outcome of the trial), because it was plausible to make an assumption that the rate of unsatisfactory outcome in a TRUNCATE strategy group would be the same as the rate of unsatisfactory outcome in the standard management group (because the excess treatment failure or relapse events occurring after an 8-week treatment regimen mostly did not count towards unsatisfactory outcome).

Therefore, instead of using a frequentist approach to formally test a hypothesis of non-inferiority that was considered to be very unlikely to be met, the approach taken for this exploratory analysis of regimen efficacy – and that was pre-specified in the statistical analysis plan – was to use a Bayesian approach to estimate the probability that the difference in the proportion of unfavourable outcome in an 8-week regimen compared to the 24-week standard treatment regimen is no greater than 12%. The choice of a 12% threshold was based on that used for evaluating non-inferiority of novel 4-month regimens in the STAND trial (and was used, similarly, for formal evaluation of non-inferiority of the TRUNCATE management strategy, as detailed in Methods S11). It could be argued that the justification for a 12% threshold might be even stronger for this analysis, given the more radical reduction in treatment duration being tested here (8-weeks, versus 4-month regimens in the STAND trial).[Tweed et al 2021]

To provide a framework for reference, it was pre-specified that in the (unlikely) event that Bayesian probability exceeds 0.95 that the difference between an 8-week regimen and the 24-week standard regimen is no greater than 12%, then the 8-week regimen could be considered as non-inferior to standard treatment. The probability of 0.95 is similar to the way a one-sided 95% confidence interval might be used to assess whether a non-inferiority hypothesis has been met in traditional frequentist analysis (and which is often interpreted, wrongly, to indicate that there is a probability of 0.95 that the non-inferiority hypothesis has been met).(Bland and Altman, 1998)

This presentation of the Bayesian probability of the difference being no greater than 12% is more aligned with the aims of these exploratory regimen analyses (as described above). This approach was considered likely to be more informative (and easier to interpret) for addressing these aims than would be an analysis that set out to test an (implausible) hypothesis that would have a high likelihood of rejection under conventional frequentist analysis.

The probability estimate allows evaluation and identification of potential regimens that might have an acceptable chance of success in a future, conventional phase 3 non-inferiority registrational trial. It also gives, by considering the probabilities across all regimens against the 12% benchmark, an overall indication of how near (high probability) or far (low probability) we are from being able to

achieve a regimen of 8 weeks duration based on modified standard treatment that has comparable efficacy to 24-week standard treatment (as described above).

The analysis also explored Bayesian probabilities in subgroups to identify those in which there was a high probability that the excess of relapses with an 8-week regimen would be sufficiently low (with reference to the same 12% threshold) that the regimen could be considered for use in that selected population - either alone as a stratified treatment approach with expectation of cure, or with additional safeguards within the TRUNCATE strategy.

In addition to the comparison of 8-week regimens with the standard 24-week treatment regimen, the analysis also evaluated the probability that the absolute rate of unfavourable outcome with an 8-week regimen was no greater than 20%. This probability estimate was considered to be of use for evaluating regimens for their suitability for the initial 8-week treatment phase in the TRUNCATE strategy. The threshold was based on a premise that that 20% would be the maximum threshold for treatment failure or relapse above which the TRUNCATE strategy would be unlikely to be feasible for programme settings (based on practical implementation constraints). This threshold was the same as the threshold of treatment failure or relapse (at the second interim) above which the IDMC was asked to consider making a recommendation to discontinue enrollment to a group (with the same rationale - lack of feasibility of implementation).

Bayesian methods are well established but uncommonly used in clinical trials. Investigators, the pharmaceutical industry and regulators routinely prefer frequentist statistical methods (null hypothesis with significance testing and P values) with which they are familiar. However, there is increasing recognition that Bayesian approaches may be well-suited to the drug development process.(Ruberg et al, 2023) Aside from the advantage of allowing incorporation of prior information into posterior estimates of probability (not used in our analysis), Bayesian analysis also has the major advantage of presenting results in terms of (posterior) probability that a hypothesis is true. This simplifies the interpretation in comparison to the usual frequentist approach. Conventional frequentist confidence intervals and P values are often misinterpreted as hypothesis probabilities, suggesting that probability estimates are what researchers really want. (Bland and Altman,1998)

Further details of the approach to the analysis of drug regimen efficacy are given in the Statistical Analysis Plan, Chapter 11

Tweed CD, Wills GH, Crook AM et al. A partially randomised trial of pretomanid, moxifloxacin and pyrazinamide for pulmonary TB. Int J TB Lung Dis 2021: 25(4):305–314.

Ruberg SJ, Beckers F, Hemmings R et al. Application of Bayesian approaches in drug development: starting a virtuous cycle. Nature Reviews Drug Discovery 2023; 22: 235-250.

Bland and Altman. Bayesians and Frequentists. BMJ 1998; 317: 1151

Summary of efficacy analyses

The analyses conducted for the primary outcome (unfavourable outcome) used to assess regimen efficacy are summarised in the table below (with those used for analysis of the TRUNCATE management strategy shown for comparison).

The main regimen analysis presents the outcome as the proportion experiencing unfavourable outcome rather than time to the outcome. The former is easier to interpret as a measure of the overall treatment efficacy relative to standard treatment for comparison with other published

studies. A secondary analysis using a time-to-event approach for unfavourable outcome is also provided. Additional secondary efficacy outcomes are listed in Methods S9.

	**Regimen analysis**	**Strategy analysis**
**Aims of analysis**		
Efficacy aim	Describe efficacy of novel 8-week regimens	Determine non-inferiority of the TRUNCATE strategy *
**Main efficacy outcome and analyses**		
Main efficacy outcome	Unfavourable outcome in ITT population, baseline to week 96 †	Unsatisfactory outcome in ITT population at week 96 †<br>(=trial primary outcome)
Main analysis of main efficacy outcome	Bayesian regression model to estimate risk difference (95% BCI) in unfavourable outcome versus standard treatment and estimate probability of risk difference ≤ 12%	Generalised linear model to estimate risk difference (97.5% CI) in unsatisfactory outcome versus standard treatment and evaluate hypothesis of non-inferiority (risk difference <12%)
Supplementary presentation of analysis of main efficacy outcome	No additional presentation	Bayesian regression model to estimate probability of risk difference ≤ 12%
Sensitivity analyses of main efficacy outcome	1.ITT assessable<br>2.Culture-positive<br>3.Fully drug-susceptible<br>4.Missed >3 days' treatment in first 56 days<br>5.Missed >14 days' treatment in first 56 days<br>6.Regimen duration >8 weeks classified as unassessable	1.ITT assessable<br>2.Per protocol ‡
Subgroup analyses of main efficacy outcome	Sex, age, country, smoking, BMI, CXR cavitation, CXR % lung affected, smear grade, relapse risk, Xpert bacillary burden, diabetes, isoniazid resistance	Sex, age, country, education, smoking, BMI, CXR cavitation, CXR % lung affected, smear grade, breathlessness grade, relapse risk, diabetes, isoniazid resistance
Analysis of main efficacy outcome by drug dose change	Time before and after systematic dose change (rifampicin dose reduction in rifampicin-linezolid group)	Analysis not specified
Time to event analysis of main efficacy outcome	Cox regression of time to unfavourable outcome	Analysis not specified

* TRUNCATE strategy comprises 8 weeks of initial treatment, monitoring to 96 weeks and re-treatment of relapses

† Unfavourable outcome (regimen analysis) and unsatisfactory outcome (strategy analysis) differ substantially. The two outcomes are compared in the following section, Methods S8

‡ Per-protocol population for the strategy analysis excluded those who did not complete the protocol-specified initial treatment or had inadequate treatment in the first 56 days (equivalent to participants who were classified as unassessable in the regimen analysis)

The outcomes and analyses of efficacy outcomes are described for the regimen and strategy analyses in the statistical analysis plan (Chapter 11 and Chapter 8 respectively).

Methods S11 PRECISION OF ESTIMATES FOR ANALYSIS OF DRUG REGIMEN EFFICACY

Precision of estimates for the analysis of drug regimen efficacy

The sample size available for the analysis of regimen efficacy was determined by the sample size set for the overall trial, based on the primary outcome and assumptions for analysis of the TRUNCATE management strategy. That sample size calculation for the overall trial is set out below.

Here we provide the precision of the estimates that can be obtained for analysis of regimen efficacy with the sample size that had been set for the main trial. For this we used a precision-based approach, suitable for the Bayesian analysis used for the analysis of regimen efficacy (Turner et al, 2023). With the set sample size of 180 per treatment group, the width of the credible interval of the estimated absolute risk of unfavourable outcome is 0.088 for the standard treatment group and 0.104 for an 8-week regimen group; the width of the credible interval for the estimated risk difference for unfavourable outcome between the standard treatment group and an 8-week treatment group is calculated to be 0.137; and the posterior probability for the estimated risk difference being no greater than 12% is 0.978.

These estimates are based on the assumption that unfavourable outcome would occur in 10% of the participants in the standard treatment group (based on the 16% rate in the standard treatment group in a trial of 4-month regimens for drug-susceptible tuberculosis [Gillespie et al. 2014], reduced to adjust for a lower proportion of participants enrolled with severe disease, expected from the initial eligibility criteria) and an estimated 5% excess rate of unfavourable outcome in an 8-week treatment group compared with the standard treatment group. The pre-specified threshold of 12% for the maximum between-group difference is arbitrary and is used as a reference for interpreting posterior probability. For ease of interpretation this is set at 12%, consistent with other trials that have investigated regimens of 4 months' duration (Tweed et al., 2021; Cevik et al. 2024), with stronger justification based on the more radical reduction in treatment duration of the regimens being tested here.

Sample size for the overall trial (for the anlaysis of TRUNCATE management strategy efficacy)

The target sample size of each treatment group in the trial was based on evaluating the trial strategy – unsatisfactory outcome – a composite of death, ongoing treatment, or active disease at week 96. We estimated that a sample of 180 participants in each trial group with complete enrollment (i.e., groups in which enrollment was not discontinued before the sample-size requirement was met) would provide the trial with 85% power to show the noninferiority of the treatment strategy to standard treatment with respect to unsatisfactory outcome. This estimation was based on a noninferiority margin of 12 percentage points; an assumption that a primary-outcome event would occur in 10% of the participants in each trial group; a one-sided significance level of 0.0125 (an adjustment for multiplicity, with the assumption of complete enrollment in two strategy groups); and the exclusion of 10% of participants from the analysis population.

A non-inferiority margin of 12% was considered to be an acceptable difference to clinical collaborators, given the expected large reduction in total treatment duration. This margin is also consistent with FDA guidance. It is estimated that the case-fatality rate for untreated smear positive pulmonary tuberculosis is 70%, ie a cure rate of 30% (Tiemersma et al, 2011). If we assume 90%

satisfactory outcomes after two years on standard treatment, this gives a standard treatment effect (denoted as M1 in FDA guidance document) of 60%. Selecting a non-inferiority margin of 12% (denoted as M2 in FDA guidance document) means that a relative proportion of 80% of the standard treatment effect is preserved in treatment arms that are declared non-inferior on the primary endpoint. This exceeds the general recommendation in the FDA guidance document of preserving at least 50% of the treatment effect. A 12% margin was used for a phase 3 regimen trial (the STAND trial) evaluating a novel regimen for the treatment of drug susceptible-TB that was initiated around the same time as this trial (Tweed et al, 2021).

The projected proportion of participants with unsatisfactory outcome of 10% was based on the folowing assumptions:

(i) the proportion with ongoing requirement for TB drug treatment or with ongoing TB disease activity at week 96 who will be classified as unsatisfactory outcome is estimated as 2%. This is based on data from REMoxTB trial (Gillespie et al, 2014) that shows that <1% of participants on the control arm initiated treatment in the last 26 weeks of follow-up (and were therefore on treatment at the end of follow-up) and <1% had positive cultures at the end of follow-up.

(ii) The proportion who will have died by week 96 (minus those who died after week 48 and for reasons clearly unrelated to TB or treatment – classified as un-assessable) is estimated as 3% based on 2.5% of deaths in REMoxTB in the control arm.

(iii) The proportion who fail to attend at week 96 and who cannot be contacted and confirmed to be clinically well is assumed to be 5%. This is difficult to estimate precisely from previous trials which were not designed as treatment strategy trials and so participants were not often followed for the full duration if retreatment was started, but it was predicted to be unlikely to exceed 5% in this trial.

References:

Tiemersma EW, van der Werf MJ, Borgdorff MW, Williams BG, Nagelkerke NJ. Natural history of tuberculosis: duration and fatality of untreated pulmonary tuberculosis in HIV negative patients: a systematic review. *PloS one* 2011; **6**(4): e17601

Non-Inferiority Clinical Trials to Establish Effectiveness; Guidance for Industry; U.S. Department of Health and Human Services, Food and Drug Administration; Center for Drug Evaluation and Research (CDER); Center for Biologics Evaluation and Research (CBER); November 2016. https://www.fda.gov/media/78504/download

Tweed CD, Wills GH, Crook AM et al. A partially randomised trial of pretomanid, moxifloxacin and pyrazinamide for pulmonary TB. Int J TB Lung Dis 2021: 25(4):305–314.

Gillespie SH, Crook AM, McHugh TD et al. Four-month moxifloxacin-based regimens for drug-sensitive tuberculosis. NEJM 2014; 371: 1588-98.

Cevik M, Thompson, LC, Upton C et al. Bedaquiline-pretomanid-moxifloxacin-pyrazinamide for drug-sensitive and drug-resistant pulmonary tuberculosis treatment: a phase 2c, open-label, multicentre, partially randomised controlled trial. Lancet Infect Dis 2024: 24 (9): 1003-1014

Turner RM, Clements MN, Quartagno M et al. Practical approaches to Bayesian sample size determination in non-inferiority trials with binary outcomes. Statistics in Medicine. 2023;42:1127–1138.

Methods S12 INTERIM ANALYSES AND SELECTION OF GROUPS FOR DISCONTINUATION OF ENROLMENT

Interim analysis

The planned interim analyses, which represent the phase 2 component of the seamless phase 2 / 3 trial design, are described in detail in the Protocol, section 10.4.

In summary, the Independent Data Monitoring Committee (IDMC) performed two pre-specified interim analyses at which they determined whether groups should discontinue enrollment based on the following thresholds:

> First interim analysis (after 30 participants in the standard treatment group reached 6 months post-randomisation): combined treatment failure/relapse rate above 25%, or hazard ratio of time to stable culture conversion versus control not exceeding 0.8
>
> Second interim analysis (after 70 participants in the standard treatment group reached 6 months post-randomisation): combined treatment failure/relapse rate above 20%, or hazard ratio of time to stable culture conversion versus control not exceeding 0.9.

The thresholds for these interim analyses were chosen to ensure high power for identifying groups that correspond to desirable treatment strategies but stopping recruitment to groups that are likely to have high rates of treatment failure or relapse. Simulation studies showed that the maximum family-wise error rate (the type I error under the assumption that none of the groups are non-inferior but no groups are dropped at interim analysis) is maintained at 4%.

The simulation studies are described in Protocol section 10.3 and the approach is explained in the following reference:

Bratton et al, Trials (2016) 17: 309. Type I error rates of muti-arm multi-stage clinical trials: strong control and impact of intermediate outcomes.

Discontinuation of treatment groups

Based on the results at interim analyses, the IDMC did not recommend discontinuation of enrollment to any group. However, the Trial Steering Committee discontinued enrollment to two groups, one after each IDMC review meeting, to ensure that sample size requirements could be met in the remaining groups. Once the decision to discontinue one group had been made, the selection of which group to discontinue was made on pragmatic grounds, blinded to outcome data.

Following the first decision to stop a group had been made, the TRUNCATE strategy rifapentine-linezolid group was selected to discontinue enrollment (after 42 participants had been enrolled) mainly due to the relatively high pill burden compared to other regimens. For participants in the 40-54kg weight band (the majority), 15 pills per day were required for this regimen, in contrast to 9-10 pills per day for the other regimens (12 per day for the first 14 days in the bedaquiline-linezolid regimen). The decision was also influenced by new regulatory guidance on quinolone toxicity.

Following the second decision to stop a group had been made, the TRUNCATE strategy rifampicin-clofazimine group was selected to discontinue enrollment (after 78 participants had been enrolled) mainly due to refusal by one national regulator to give approval for continued clofazimine importation following a switch in drug manufacturer (from generic to originator brand) arising from production issues.

SUPPLEMENTARY TABLES

Table S1: REASONS FOR NON-ELIGIBILITY

Inclusion criterion not met	Participants
Absence of symptoms and/or CXR changes consistent with tuberculosis	11 (2%)
Xpert test negative	170 (34%)
Unwilling to comply with visits	32 (6%)
Not living at fixed, accessible address	2 (<1%)
Not willing to have directly observed therapy	3 (1%)
Withdrew informed consent prior to randomisation	24(5%)
Exclusion criterion met	
Taken more than 10 doses of tuberculosis medication	5 (1%)
Past tuberculosis	4 (1%)
Known/suspected extra-pulmonary tuberculosis	13 (3%)
Severe clinical pulmonary tuberculosis	4 (1%)
Sputum smear 3+ (prior to removal of criterion)	68 (13%)
Cavity size > 4cm on CXR (prior to removal of criterion)	67 (13%)
Rifampicin resistance on Xpert test	10 (2%)
Poorly-controlled diabetes	21 (4%)
Active malignancy requiring chemo/ radiotherapy	1 (<1%)
Known HBV or HCV infection	4 (1%)
Cardiac disease	5 (1%)
Seizures	1 (<1%)
Current alcohol or drug abuse	4 (1%)
Pregnancy / breast-feeding	1 (<1%)
Allergy to study drugs	1 (<1%)
Immunosuppressive drugs / corticosteroids	3 (1%)
Colour blindness	26 (5%)
QTc >450ms at screening	10 (2%)
Abnormal laboratory results at screening	72 (14%)
HIV positive	7 (1%)
Other diseases compromise safety / protocol compliance	28 (6%)

Data are n (%). Table shows the number of participants for which each criterion was indicated as a reason for non-eligibility, and the proportion of the total of 504 participants who were not enrolled due to that criterion. Participants may have more than one reason for non-eligibility.

Table S2 TREATMENT DURATION AND REASONS FOR EXTENSION OR SWITCH

	Standard treatment 24-week regimen (n=181)	Rifampicin-linezolid 8-week regimen (n=184)	Rifampicin-clofazimine 8-week regimen (n=78)	Rifapentine-linezolid 8-week regimen (n=42)	Bedaquiline-linezolid 8-week regimen (n=189)
**Completed initial treatment with assigned regimen only**					
Participants in category	178 (98)	169 (92)	72 (92)	31 (74)	179 (95)
≥ 54 to 56 qualifying days	-	143 (78)	60 (77)	28 (67)	162 (86)
≥ 57 to 70 qualifying days *	-	21 (11)	8 (10)	2 (5)	13 (7)
≥ 71 to 84 qualifying days †	-	5 (3)	4 (5)	1 (2)	4 (2)
≥ 154 qualifying days	178 (98)	-	-	-	-
Total qualifying days of assigned regimen	173.1 ± 25.4	58.4 ± 6.1	58.6 ± 6.7	57.5 ± 5.6	57.7 ± 5.3
**Completed initial treatment with assigned regimen for 84 qualifying days; extended with standard treatment regimen**					
Participants in category ‡	-	3 (2)	1 (1)	0	3 (2)
Total qualifying days of assigned regimen	-	84 ± 0	84	-	84.0 ± 0
**Did not complete initial treatment with assigned regimen**					
Participants in category	3 (2)	12 (7)	5 (6)	11 (26)	7 (4)
Reason(s) for non-completion					
Died before completion of assigned treatment	2	1	0	0	0
Treatment interruption > 8 weeks before completion of assigned treatment	1	1	0	1	2
Switched to standard treatment §	-	10	5	10	5
Reasons for switch					
Missed doses ¶	-	3	1	2	0
Adverse event(s)	-	6	5	8	3
Pill burden/tolerability	-	2	0	3	1
Decision by participant (other reason)	-	3	0	2	1
Logistical reason (drug supply interruption)	-	0	0	0	1
Total qualifying days of assigned regimen	107.3 ± 62.6	14.3 ± 12.0	17.2 ± 16.7	11. 1 ± 10.6	17.6 ± 19.4

Data are n; n (%); and mean ± SD.

Data previously published in Paton, Cousins, Suresh et al; N Engl J Med; 2023; 388: 873-887; adapted with categorical classifications based on completion of assigned regimen (irrespective of completion of assigned TRUNCATE strategy).

Completed assigned regimen is defined as taking at least 54 qualifying days of the assigned regimen in the 8-week regimen groups; and at least 154 qualifying days of the standard treatment regimen in the 24-week standard regimen group. A qualifying day is defined as a day on which treatment was taken with at least 50% of the protocol-mandated dose of all drugs in the assigned regimen (exceptions and detailed definition are given in the protocol and statistical analysis plan).

* Extension with the assigned regimen in 44 participants to complete treatment after a total of ≥57 to 70 qualifying days was for persistent clinical disease (symptoms and positive smear) in 12; symptoms with either negative smear or no sputum available in 25; positive smear without symptoms in 2; miscalculation of days in 4; and clinical decision in 1 participant. Later review established that 35 of the 44 had met the criteria for culture conversion by the 56th qualifying day of treatment; for the remaining 9, the last qualifying day of treatment on which a positive culture was observed was day 0, 1, 14, 26, 28, 40, 53 and 57 and one had no positive culture at any time.

† Extension with the assigned regimen in 14 participants to complete treatment after a total of ≥71 to 84 qualifying days was for persistent clinical disease (symptoms and positive smear) in 9; and symptoms with either negative smear or no sputum available in 5 participants. One participant classified in this category took 85 qualifying days of the assigned regimen in error. Later review established that 13 of the 14 had met the criteria for culture conversion by the 56th qualifying day of treatment; for the remaining participant, the last qualifying day of treatment on which a positive culture was observed was day 53.

‡ Extension with standard treatment in 7 participants after completing a total of 84 qualifying days of the assigned regimen was for persistent clinical disease (symptoms and positive smear) in 6, and symptoms with negative smear in 1 participant. Later review established that 2 of the 7 had met the criteria for culture conversion by the 56th qualifying day of treatment; for the remaining 5 participants, the last qualifying day of treatment on which a positive culture was observed was day 38, 42, 54, 55 and 79.

§ Participants may have more than 1 reason for switch to standard treatment

¶ Switch to standard treatment was mandated for treatment interruption lasting more than 14 consecutive days or if the required number of days of the assigned initial regimen could not be completed by week 12.

Table S3 TREATMENT ADHERENCE

	Standard treatment 24-week regimen (n=181)	Rifampicin-linezolid 8-week regimen (n=184)	Rifampicin-clofazimine 8-week regimen (n=78)	Rifapentine-linezolid 8-week regimen (n=42)	Bedaquiline-linezolid 8-week regimen (n=189)
**Completing initial treatment with assigned regimen only**					
Participants in category	178 (98)	169 (92)	72 (92)	31 (74)	179 (95)
Adherence over the treatment course (assigned regimen), %	98.6 ± 4.9	97.8 ± 5.1	98.5 ± 4.1	98.9 ± 2.4	99.0 ± 3.0
Missed > 3 and ≤ 7 days	8 (4)	6 (4)	3 (4)	0	5 (3)
Missed > 7 and ≤ 14 days	3 (2)	12 (7)	4 (6)	1 (3)	4 (2)
Missed > 14 days	10 (6)	2 (1)	0	0	0
Classified as unassessable (missed > 7 days by trial day 56) *	3	12	3	0	5
**Completed initial treatment with assigned regimen for 84 qualifying days; extended with standard treatment regimen**					
Participants in category †	-	3 (2)	1 (1)	0	3 (2)
Adherence over treatment course (assigned regimen), %	-	100.0 ± 0	100	-	100.0 ± 0
Missed > 3 and ≤ 7 days	-	0	0	0	0
Missed > 7 and ≤ 14 days	-	0	0	0	0
Missed > 14 days	-	0	0	0	0
Classified as unassessable (missed > 7 days by trial day 56)	-	0	0	0	0
**Did not complete initial treatment with assigned regimen**					
Participants in category ‡	3 (2)	12 (7)	5 (6)	11 (26)	7 (4)
Adherence over treatment course (assigned regimen), %	100 ± 0	61.6 ± 28.0	41.2 ± 17.9	61.8 ± 30.4	86.6 ± 19.2
Missed > 3 and ≤ 7 days	0	2	0	3	0
Missed > 7 and ≤ 14 days	0	1	2	2	2
Missed > 14 days	0	4	3	2	0
Classified as unassessable (missed > 7 days by trial day 56)	0	2	1	1	1

Data are n; n (%); and mean ± SD.

Completed assigned regimen is defined as taking at least 54 qualifying days of the assigned regimen in the 8-week regimen groups; and at least 154 qualifying days of the standard treatment regimen in the 24-week standard regimen group. A qualifying day is defined as a day on which treatment was taken with at least 50% of the protocol-mandated dose of all drugs in the assigned regimen (exceptions and detailed definition are given in the protocol and statistical analysis plan). Adherence over treatment course (assigned regimen) is the proportion of the days that were classified as a qualifying day during the treatment course with the assigned regimen (period between the

first and last qualifying day of the assigned regimen). A missed day is a day that was not classified as a qualifying day during the treatment course with the assigned regimen.

* In the standard treatment, rifampicin-linezolid, rifampicin-clofazimine, and rifapentine-linezolid groups, the total number of participants classified as missed > 7 days during the treatment course with the assigned regimen is greater than the number of participants classified as unassessable (missed > 7 days by trial day 56) on the primary outcome because the latter does not count days of non-adherence to the assigned regimen beyond trial day 56 in those participants whose treatment with the assigned regimen extends beyond day 56. In the bedaquiline-linezolid group, one participant was not classified as missed > 7 days during the treatment course with the assigned regimen but was classified as unassessable (missed > 7 days by trial day 56) on the primary outcome. This is because the participant started the assigned regimen 8 days after baseline to allow correction of electrolyte abnormalities (maintaining standard treatment during this period); then completed 56 days' treatment with the assigned regimen with no missed days; the initial period counts does not count as non-adherence prior to the first day of treatment with the assigned regimen, but is counted as days of non-adherence to the assigned treatment during in the first 56 days of the trial.

† In the bedaquiline-linezolid group, one of the 3 participants classified as completed 84 days of assigned treatment followed by extension with standard treatment was not classified as unassessable (completed 84 days of assigned treatment then extended with standard treatment) because the participant had a positive sputum culture at the time of switch and thus met criteria for classification as unfavourable outcome.

‡ The number of participants that did not complete treatment shown here differs from the number classified as unassessable (incomplete treatment [<154 days assigned standard treatment group, < 54 days other treatment groups]) because the efficacy analysis classifies participants according to the first event that meets criteria for unassessable (which may include death; and > 7 days missed treatment by trial day 56 that may occur prior to the last qualifying day of treatment). In the standard treatment group, of the 3 participants that did not complete treatment, 1 was classified as unassessable for this reason and 2 were classified as unfavourable (and therefore included in the assessable population) due to death (not considered unrelated to TB) during treatment. In the rifampicin-linezolid group, of the 12 participants that did not complete treatment, 9 were classified as unassessable for this reason, 2 were classified as unassessable due to missing > 7 days and 1 was classified as unfavourable (and included in the assessable population) due to death (not considered unrelated to TB) during treatment. In the rifampicin-clofazimine group, of the 5 participants that did not complete treatment, 4 were classified as unassessable for this reason and 1 was classified as unassessable due to missing > 7 days. In the rifampicin-linezolid group, of the 11 that did not complete treatment, 10 were classified as unassessable for this reason and 1 was classified as unassessable due to missing > 7 days. In the bedaquiline-linezolid group, of the 7 participants that did not complete initial treatment, 6 were classified as unassessable for this reason, and 1 was classified as unassessable due to missing > 7 days.

Table S4 COMPARISON OF CLASSIFICATION OF OUTCOME EVENTS FOR DRUG REGIMEN AND TRUNCATE MANAGEMENT STRATEGY EFFICACY ANALYSIS

		Outcome classification in the strategy analysis			
**Outcome classification and subcategories in the regimen analysis**		**Unsatisfactory outcome (total 49)**	**Unassessable outcome (total 4)**	**Satisfactory outcome * (total 621)**	**Subcategories and reason for differences in the strategy analysis ***
**Unfavourable outcome (total 96)**		**40**	**1**	**55**	
Switched treatment with positive culture	1	0	0	1	Switch ignored in the strategy analysis
Failure at end of treatment	1	1	0	0	Failure at end of treatment ignored in the strategy analysis<br>Unsatisfactory (1): Death before W96
Relapse confirmed	81	29	1	51	Relapse ignored in the strategy analysis<br>Unsatisfactory (29): On TB treatment at week 96 (14); TB disease activity at W96 (13); death before W96 (1); W96 telephone evaluation, insufficient evidence of TB clearance when last seen (1)<br>Unassessable (1): single positive culture at W96, no other evidence of TB activity
Relapse unconfirmed	3	0	0	3	Relapse ignored in the strategy analysis
Death, except unrelated	6	6	0	0	Unsatisfactory (6): death before W96
Not seen at W96, unfavourable	4	4	0	0	Unsatisfactory (4): W96 telephone evaluation (3) or no evaluation (1), insufficient evidence of TB clearance when last seen
**Unassessable outcome (total 76)**		**9**	**3**	**64**	
Incomplete initial treatment	30	5	0	25	Incomplete initial treatment ignored in the strategy analysis<br>Unsatisfactory (5): tuberculosis disease activity at W96 (1): death before week 96 (1); W96 telephone evaluation (2) or no evaluation (1), insufficient evidence of TB clearance when last seen
Missed >7 days' treatment in first 56 days	28	1	0	27	Missed treatment ignored in the strategy analysis<br>Unsatisfactory (1): On TB treatment at W96
Switch treatment without positive culture	6	0	0	6	Switch ignored in the strategy analysis
Restarted treatment without failure/relapse	9	3	0	6	Restarted treatment ignored in the strategy analysis<br>Unsatisfactory (3): On TB treatment at W96
Death, unrelated	1	0	1	0	Unassessable (1): death, unrelated
Not seen at W96, unassessable	2	0	2	0	Unassessable (2): W96 no evaluation, evidence of TB clearance when last seen
**Favourable outcome (total 502)**		**0**	**0**	**502**	

* Classified as satisfactory outcome in the strategy analysis if there is no event that meets the criteria for unsatisfactory or unassessable outcome. Strategy outcomes as previously published in Paton, Cousins, Suresh et al; N Engl J Med; 2023; 388: 873-887

Table S5 PRIMARY OUTCOME SENSITIVITY ANALYSES IN RIFAMPICIN-LINEZOLID AND BEDAQUILINE-LINEZOLID GROUPS

	Standard treatment 24-week regimen (n=181)	Rifampicin-linezolid 8-week regimen (n=184)	Risk difference (95% BCI)*	P Diff. ≤ 12%	Bedaquiline-linezolid 8-week regimen (n=189)	Risk difference (95% BCI)*	P Diff. ≤ 12%
**Intention-to-treat population (primary analysis population)**							
Unfavourable outcome	7 / 181 (4%)	46 /184 (25%)	-	-	26 /189 (14%)	-	-
Estimated absolute risk (%, 95%BCI)	3.5 (1.5 to 6.6)	24.5 (18.4 to 31.0)	21.0 (14.3 to 28.1)	0.004	12.9 (8.6 to 18.0)	9.3 (4.3 to 14.9)	0.837
Probability that absolute risk ≤ 20%	1.0	0.076	-	-	0.996	-	-
**Assessable population †**							
Unfavourable outcome	7 / 175 (4%)	46/ 155 (30%)	-	-	26/ 173 (15%)	-	-
Estimated absolute risk (%, 95%BCI)	3.6 (1.4 to 6.7)	29.3 (22.2 to 37.0)	25.7 (18.0 to 33.8)	<0.001	14.1 (9.3 to 19.6)	10.5 (5.0 to 16.5)	0.698
Probability that absolute risk ≤ 20%	1.000	0.004	-	-	0.983	-	-
**Culture-positive population ‡**							
Unfavourable outcome	6 / 166 (4%)	44 / 168 (26%)	-	-	26 / 172 (15%)	-	-
Estimated absolute risk (%, 95%BCI)	3.4 (1.3 to 6.6)	25.9 (19.5 to 32.9)	22.5 (15.4 to 29.9)	0.001	14.5 (9.7 to 20.1)	11.1 (5.4 to 17.1)	0.628
Probability that absolute risk ≤ 20%	1.000	0.038	-	-	0.973	-	-
**Fully drug-susceptible population §**							
Unfavourable outcome	6 / 148 (4%)	39 /155 (25%)	-	-	23 / 159 (14%)	-	-
Estimated absolute risk (%, 95%BCI)	3.7 (1.3 to 7.1)	24.6 (18.0 to 31.8)	21.0 (13.8 to 28.6)	0.008	13.5 (8.6 to 19.1)	9.8 (4.2 to 16.0)	0.770
Probability that absolute risk ≤ 20%	1.000	0.096	-	-	0.987	-	-
**Missed > 3 days treatment in first 56 days (as first event) classified as unassessable ¶**							
Unfavourable outcome	7 /181 (4%)	44/184 (24%)	-	-	24/189 (13%)	-	-
Estimated absolute risk (%, 95%BCI)	3.4 (1.4 to 6.3)	23.1 (17.3 to 29.8)	19.7 (13.3 to 26.6)	0.008	11.6 (7.4 to 16.5)	8.1 (3.3 to 13.5)	0.929
Probability that absolute risk ≤ 20%	1.000	0.163	-	-	0.999	-	-
**Missed > 14 days treatment in first 56 days (as first event) classified as unassessable ‖**							
Unfavourable outcome	7/181 (4%)	46 / 184 (25%)	-	-	27 / 189 (14%)	-	-
Estimated absolute risk (%, 95%BCI)	3.6 (1.4 to 6.6)	24.6 (18.5 to 30.9)	21.0 (14.3 to 27.9)	0.003	13.4 (9.0 to 18.6)	9.9 (4.5 to 15.4)	0.782
Probability that absolute risk ≤ 20%	1.000	0.074	-	-	0.993	-	-
**Regimen duration > 8 weeks (as first event) classified as unassessable ****							
Unfavourable outcome	7 / 181 (4%)	41/184 (22%)	-	-	23/189 (12%)	-	-
Estimated absolute risk (%, 95%BCI)	3.6 (1.4 to 6.6)	21.8 (16.1 to 28.1)	18.2 (11.8 to 25.0)	0.029	11.5 (7.5 to 16.5)	7.9 (3.0 to 13.4)	0.934
Probability that absolute risk ≤ 20%	1.000	0.291	-	-	1.000	-	-

Treatment switch, failure or relapse with single positive culture (as first event) classified as unassessable ††							
Unfavourable outcome	7 / 181 (4%)	46 /184 (25%)	-	-	22/189 (12%)	-	-
Estimated absolute risk (%, 95%BCI)	3.5 (1.4 to 6.5)	24.6 (18.7 to 31.0)	21.1 (14.6 to 28.0)	0.003	10.9 (6.8 to 15.7)	7.4 (2.4 to 12.8)	0.958
Probability that absolute risk ≤ 20%	1.000	0.073	-	-	1.000	-	-

Data are n/N (%) unless otherwise stated. Absolute risk and 95% Bayesian Credible Interval (95% BCI), risk difference (95% BCI) and probability that absolute risk ≤ 20% and risk difference ≤ 12% were estimated from regression models using Bayesian methods, with a normal prior (mean 1, variance 100) applied to the intercept and independent variables of treatment group, country (India and Thailand, combined; Indonesia; Philippines; and Uganda) and for baseline relapse risk (lower; intermediate and higher combined), weighted in proportion to number of participants in each category.

* Risk difference between the 8-week regimen groups and the standard treatment group is expressed in percentage points.

† Population excludes those with unassessable outcome

‡ Population excludes those that did not have at least one positive culture up to the time of the week 2 visit.

§ Population excludes those with phenotypic drug resistance to one or more of the drugs in the randomly-assigned regimen on an isolate obtained at baseline or the first available isolate if no isolate was obtained at baseline.

¶ Outcome classified as unassessable at the third missed dose within the first 56 days if this was the first outcome event (modified from seventh missed dose in the primary analysis)

‖ Outcome classified as unassessable at the 14th missed dose within the first 56 days if this was the first outcome event (modified from seventh missed dose in the primary analysis)

** Outcome classified as unassessable at the 58th qualifying day of treatment in those who took more than 58 qualifying days of the assigned regimen in the 8-week regimen groups if this was the first outcome event (modified from primary analysis in which such participants are classified as unassessable only if they later switch to or extend with standard treatment [prior to the occurrence of another outcome event]).

†† Outcome classified as unassessable at the point of treatment switch, treatment failure or relapse with a single positive culture if this was the first outcome event (modified from the primary analysis in which such participants are classified as unfavourable)

TABLE S6 PRIMARY OUTCOME EFFICACY ANALYSIS BY ASSIGNED RIFAMPICIN DOSE IN THE RIFAMPICIN-LINEZOLID GROUP

	**Period 1**		**Period 2**	
	**Standard treatment 24-week regimen (n=91)**	**Rifampicin (35mg/kg)-linezolid 8-week regimen (n=88)**	**Standard treatment 24-week regimen (n=90)**	**Rifampicin (20mg/kg)-linezolid 8-week regimen (n=96)**
**Primary outcome**				
Unfavourable outcome	3 (3%)	17 (19%)	4 (4%)	29 (30%)
Estimated absolute risk (%, 95% BCI)	2.9 (0.6 to 6.7)	17.6 (10.0 to 26.4)	3.6 (1.0 to 8.0)	28.2 (18.8 to 38.3)
Probability that absolute risk ≤ 20%	1.000	0.731	1.000	0.043
Risk difference (%, 95% BCI) *	-	14.7 (6.5 to 24.0)	-	24.6 (14.9 to 35.2)
Probability that risk difference ≤12%	-	0.280	-	0.007
**Outcome classification**				
**Unfavourable outcome – total**	3 (3%)	17 (19%)	4 (4%)	29 (30%)
Switch treatment with positive culture	0	0	0	0
Failure at end of treatment	0	0	0	1 (1%)
Relapse confirmed †	2 (2%)	16 (18%)	2 (2%)	24 (25%)
Relapse unconfirmed	0	0	0	0
Death, except unrelated ‡	0	1 (1%)	2 (2%)	2 (2%)
Not seen at W96, unfavourable §	1 (1%)	0	0	2 (2%)
**Unassessable outcome -total**	3 (3%)	22 (25%)	3 (3%)	7 (7%)
Incomplete initial treatment ¶	1 (1%)	7 (8%)	0	2 (2%)
Miss >7 days' treatment in first 56 days	1 (1%)	13 (15%)	2 (2%)	1 (1%)
Switch treatment without positive culture	0	1 (1%)	0	2 (2%)
Restart treatment without failure/relapse	1 (1%)	1 (1%)	0	2 (2%)
Death, unrelated ‖	0	0	1 (1%)	0
Not seen at W96, unassessable **	0	0	0	0
**Favourable outcome – total**	85 (93%)	49 (56%)	83 (92%)	60 (63%)

Data are n or n (%) unless otherwise stated. The standard treatment regimen comprised rifampicin (10mg/kg) and isoniazid for 24 weeks, with pyrazinamide and ethambutol for the first 8 weeks; the rifampicin-linezolid regimen comprised rifampicin (35mg/kg or 20mg/kg), isoniazid, pyrazinamide, ethambutol and linezolid. Table shows outcomes for participants 1-88 (period 1, assigned 35mg/kg rifampicin) and 89-184 (period 2, assigned 20mg/kg rifampicin) enrolled in the rifampicin-linezolid group and for participants who underwent contemporary randomisation to the standard treatment group during these periods. Primary outcome classification as unfavourable or unassessable outcome is determined by the first outcome event to meet either of the criteria; classification as favourable outcome is determined by the absence of any event by week 96. Analyses are done in the intention-to-treat population. Absolute risk and 95% Bayesian Credible Interval (95% BCI), risk difference (95% BCI), probability that absolute risk ≤ 20% and probability that risk difference ≤ 12% were estimated from regression models using Bayesian methods, with a normal prior (mean 0, variance

100) applied to the intercept and independent variables of treatment group, country (India and Thailand combined; Indonesia; Philippines; and Uganda) and baseline relapse risk (lower; medium and high combined; based on sputum smear and chest X-ray cavitation) weighted in proportion to number of participants in each category.

* Risk difference between the rifampicin-linezolid group and the standard treatment group (participants undergoing contemporary randomisation) is expressed in percentage points.

† Whole genome sequencing was available in paired baseline and relapse isolates for 2/2 in the standard treatment group and 12/16 in the rifampicin-linezolid group in period 1; and 2/2 in the standard treatment group and 24/24 in the rifampicin-linezolid group in period 2. In all cases the strains were related, consistent with relapse.

‡ Deaths that were not considered unrelated to tuberculosis (or anti-tuberculosis drugs) were due to cardiac arrest and possible cerebrovascular accident in the standard treatment group (both in period 2); and drug-induced liver injury (period 1), cirrhosis and COVID-19 disease (period 2) in the rifampicin-linezolid group.

§ Participants that did not attend at week 96 were classified as unfavourable if there was no evidence of good treatment outcome at the last attended visit or if they were contacted and had evidence of disease activity at week 96.

¶ Incomplete initial treatment defined as taking <154 days total of standard treatment in the standard regimen group or <54 days total of the assigned regimen in the 8-week regimen groups.

‖ Death unrelated to tuberculosis in the standard treatment group was due to cervical carcinoma.

** Participants that did not attend at week 96 were classified as unassessable if there was evidence of good treatment outcome at the last attended visit but they could not be contacted to assess disease activity at week 96.

Table S7 PRIMARY OUTCOME EFFICACY ANALYSIS IN MODELS BASED ON PARTICIPANTS UNDERGOING CONTEMPORARY RANDOMISATION

	Standard treatment 24-week regimen	Rifampicin-linezolid 8-week regimen	Rifampicin-clofazimine 8-week regimen	Rifapentine-linezolid 8-week regimen	Bedaquiline- linezolid 8-week regimen
**Model 1: all participants in the three fully-enrolled treatment groups**					
Number in model	181	184	-	-	189
Unfavourable outcome	7 (4%)	46 (25%)	-	-	26 (14%)
Estimated absolute risk (%, 95%BCI)	3.5 (1.5 to 6.6)	24.5 (18.4 to 31.0)	-	-	12.9 (8.6 to 18.0)
Probability that absolute risk ≤ 20%	1.000	0.076	-	-	0.996
Risk difference (%, 95% BCI) *	-	21.0 (14.3 to 28.1)	-	-	9.3 (4.3 to 14.9)
Probability that risk difference ≤12%	-	0.004	-	-	0.837
**Model 2: all participants in the rifampicin-clofazimine group and those with contemporary randomisation to the three fully-enrolled treatment groups**					
Number in model	76	78	78	-	75
Unfavourable outcome	2 (3%)	17 (22%)	10 (13%)	-	8 (11%)
Estimated absolute risk (%, 95%BCI)	2.3 (0.3 to 6.2)	19.9 (11.6 to 30.0)	11.2 (5.4 to 18.9)	-	8.7 (3.5 to 15.8)
Probability that absolute risk ≤ 20%	1.000	0.540	0.984	-	0.997
Risk difference (%, 95% BCI) *	-	17.6 (8.8 to 27.8)	8.9 (2.0 to 17.2)	-	6.4 (0.2 to 13.8)
Probability that risk difference ≤12%	-	0.126	0.800	-	0.937
**Model 3: all participants in the rifapentine-linezolid group and those with contemporary randomisation to other treatment groups**					
Number in model	44	47	41	42	46
Unfavourable outcome	2 (5%)	11 (23%)	7 (17%)	7 (17%)	5 (11%)
Estimated absolute risk (%, 95%BCI)	4.1 (0.5 to 11.1)	21.5 (11.1 to 34.7)	14.9 (6.0 to 27.2)	14.9 (6.2 to 26.8)	9.9 (3.2 to 20.0)
Probability that absolute risk ≤ 20%	1.000	0.424	0.828	0.831	0.975
Risk difference (%, 95% BCI) *	-	17.4 (5.2 to 31.0)	10.8 (-0.1 to 23.8)	10.8 (0.1 to 23.7)	5.8 (-3.6 to 16.6)
Probability that risk difference ≤12%	-	0.205	0.594	0.606	0.895

Data are n or n (%) unless otherwise stated. Analyses are done in the intention-to-treat population. Absolute risk and 95% Bayesian Credible Interval (95% BCI), risk difference (95% BCI), probability that absolute risk ≤ 20% and probability that risk difference ≤ 12% were estimated from regression models using Bayesian methods, with a normal prior (mean 0, variance 100) applied to the intercept and independent variables of treatment group, country (India and Thailand, combined; Indonesia; Philippines; and Uganda) and baseline relapse risk (lower; intermediate and higher combined), weighted in proportion to number of participants in each category. Three models were done: one for all participants in the rifampicin-linezolid, bedaquiline-linezolid and standard treatment groups combined; one for all participants in the rifampicin-clofazimine group with those undergoing contemporary randomisation to the rifampicin-linezolid, bedaquiline-linezolid and standard treatment groups; and one for all participants in the rifapentine-linezolid group with those undergoing contemporary randomisation to all other groups. Models and comparisons with standard treatment group were pre-specified as limited to the fully-enrolled 8-week groups; these comparisons were also performed as post-hoc analyses for the two groups in which enrollment was discontinued early. * Risk difference between the 8-week regimen groups and the standard treatment group (participants undergoing contemporary randomisation) is expressed in percentage points.

Table S8 UNCONFIRMED ACQUIRED DRUG RESISTANCE

Treatment group	Drug resistance (week first detected)	Details
Rifampicin-linezolid	Isoniazid (Week 16)	Isoniazid heteroresistance, alternating susceptible and resistant isolates on phenotypic testing detected at week 16 and relapse at week 48 (inhA mutation detected in resistant isolates on whole genome sequencing; absent in susceptible isolates); only one isolate at baseline (susceptible, no inhA mutation) prior to week 16 is insufficient to establish absence of heterogeneity at trial entry
Rifampicin-linezolid	Pyrazinamide (Week 96)	Pyrazinamide resistance on phenotypic testing of the single isolate available at week 96 (no mutation detected on whole genome sequencing), no subsequent isolates available to confirm
Rifampicin-linezolid	Pyrazinamide (Week 84)	Pyrazinamide resistance on phenotypic testing on isolates at week 84 and 86 (sequencing unsuccessful); pyrazinamide susceptible isolates on phenotypic testing at week 83 (no mutation detected on whole genome sequencing) and at week 88

Table S9 GRADE 3-4 ADVERSE EVENTS

System Organ Class<br>Preferred Term	Standard treatment 24-week regimen (n=181)	Rifampicin-linezolid 8-week regimen (n=184)	Rifampicin-clofazimine 8-week regimen (n=78)	Rifapentine-linezolid 8-week regimen (n=42)	Bedaquiline-linezolid 8-week regimen (n=189)
**All system organ classes**					
Any	25 (14%)	20 (11%)	8 (10%)	9 (21%)	22 (12%)
Related to TB medication	13 (7%)	11 (6%)	6 (8%)	7 (17%)	18 (10%)
**Infections & infestations**					
Any	0	1 (1%)	0	0	2 (1%)
Lung abscess	0	1 (1%)	0	0	0
Pneumonia	0	0	0	0	2 (1%)
**Blood and lymphatic system disorders**					
Any	8 (4%)	3 (2%)	0	3 (7%)	14 (7%)
Anaemia	5 (3%)	2 (1%)	0	2 (5%)	14 (7%)
Lymphadenopathy	0	0	0	1 (2%)	0
Neutropenia	3 (2%)	1 (1%)	0	0	0
**Immune system disorders**					
Anaphylactic reaction	0	1 (1%)	0	0	0
**Metabolism and nutrition disorders**					
Any	1 (1%)	6 (3%)	1 (1%)	0	1 (1%)
Decreased appetite	0	3 (2%)	0	0	0
Diabetes mellitus	1 (1%)	1 (1%)	0	0	0
Gout	1 (1%)	0	0	0	0
Hyperglycaemia	0	1 (1%)	1 (1%)	0	0
Hyperuricaemia	0	0	0	0	1 (1%)
Hypokalaemia	1 (1%)	0	0	0	0
Hyponatraemia	0	1 (1%)	0	0	0
**Psychiatric disorders**					
Insomnia	0	0	0	0	1 (1%)
**Nervous system disorders**					
Any	1 (1%)	1 (1%)	2 (3%)	1 (2%)	1 (1%)
Cerebrovascular accident	1 (1%)	0	1 (1%)	0	0
Dizziness	0	1 (1%)	1 (1%)	0	0

Headache	0	0	0	1 (2%)	1 (1%)
**Cardiac disorders**					
Cardiac arrest	1 (1%)	0	0	0	0
**Vascular disorders**					
Any	1 (1%)	2 (1%)	0	0	0
Hypertension	0	1 (1%)	0	0	0
Hypotension	1 (1%)	1 (1%)	0	0	0
**Respiratory, thoracic and mediastinal disorders**					
Any	1 (1%)	3 (2%)	0	0	1 (1%)
Asthma	0	1 (1%)	0	0	0
Bronchospasm	1 (1%)	0	0	0	0
Cough	0	1 (1%)	0	0	0
Pneumothorax	0	1 (1%)	0	0	1 (1%)
**Gastrointestinal disorders**					
Any	1 (1%)	2 (1%)	1 (1%)	4 (10%)	2 (1%)
Diarrhoea	0	0	0	0	1 (1%)
Nausea	0	0	0	2 (5%)	0
Vomiting	1 (1%)	2 (1%)	1 (1%)	2 (5%)	1 (1%)
**Hepatobiliary disorders**					
Any	6 (3%)	6 (3%)	4 (5%)	4 (10%)	1 (1%)
Acute hepatic failure	0	1 (1%)	0	0	0
Drug-induced liver injury	3 (2%)	1 (1%)	1 (1%)	0	0
Hepatitis	1 (1%)	0	2 (3%)	0	0
Hepatotoxicity	1 (1%)	1 (1%)	0	1 (2%)	1 (1%)
Hyperbilirubinaemia	1 (1%)	3 (2%)	1 (1%)	3 (7%)	0
Jaundice	0	0	1 (1%)	0	0
**Musculoskeletal and connective tissue disorders**					
Musculoskeletal pain	1 (1%)	0	0	0	0
**General disorders and administration site conditions**					
Asthenia	1 (1%)	0	0	0	0
**Investigations**					
Any	4 (2%)	0	1 (1%)	0	3 (2%)
Alanine aminotransferase increased	3 (2%)	0	0	0	1 (1%)
Blood glucose increased	1 (1%)	0	0	0	0
Blood potassium increased	0	0	0	0	1 (1%)

Electrocardiogram QT prolonged	0	0	0	0	1 (1%)
Weight decreased	0	0	1 (1%)	0	0

Data are n (%). Table shows number of participants with at least one incident grade 3 or 4 adverse event in each event category. Incident adverse events are events with start date (if started at grade 3 or 4) or date of increase in grade (to grade 3 or 4) occurring in the period between the day of the baseline visit and 30 days following the day of cessation (last qualifying day) of the assigned regimen, inclusive. A qualifying day is defined as a day on which treatment was taken with at least 50% of the protocol-mandated dose of all drugs in the assigned regimen (exceptions and detailed definition are given in the protocol and statistical analysis plan). Events were graded using Division of AIDS Toxicity Criteria; coded using the Medical Dictionary for Regulatory Activities (MedDRA) and listed by single MedDRA Preferred Term.

Table S10. SERIOUS ADVERSE EVENTS

System Organ Class<br>Preferred Term	Standard treatment 24-week regimen (n=181)	Rifampicin-linezolid 8-week regimen (n=184)	Rifampicin-clofazimine 8-week regimen (n=78)	Rifapentine-linezolid 8-week regimen (n=42)	Bedaquiline-linezolid 8-week regimen (n=189)
**Infections and infestations**					
Dengue fever	1 (1%)	0	0	0	0
Lung abscess	0	1 (1%)	0	0	0
Pneumonia	0	0	0	0	1 (1%)
**Blood and lymphatic system disorders**					
Anaemia	0	0	0	0	2 (1%)
**Immune system disorders**					
Anaphylactic reaction	0	1 (1%)	0	0	0
**Metabolism and nutrition disorders**					
Gout	1 (1%)	0	0	0	0
**Nervous system disorders**					
Cerebrovascular accident	1 (1%)	0	1 (1%)	0	0
**Cardiac disorders**					
Cardiac arrest	1 (1%)	0	0	0	0
**Respiratory, thoracic and mediastinal disorders**					
Asthma	0	1 (1%)	0	0	0
Pneumothorax	0	1 (1%)	0	0	1 (1%)
**Gastrointestinal disorders**					
Vomiting	1 (1%)	2 (1%)	1 (1%)	2 (5%)	1 (1%)
**Hepatobiliary disorders**					
Acute hepatic failure	0	1 (1%)	0	0	0
Drug-induced liver injury	1 (1%)	1 (1%)	1 (1%)	0	0
Hepatitis	0	0	1 (1%)	0	0
Hepatotoxicity	0	0	0	1 (2%)	0
Jaundice	0	0	1 (1%)	0	0
**Investigations**					
Electrocardiogram repolarisation abnormality	1 (1%)	0	0	0	0

Data are n (%). Table shows number of participants with at least one incident serious adverse event in each event category. Incident serious adverse events are events with start date (date met criteria for classification as serious) occurring in the period between the day of the baseline visit and 30 days following the day of cessation (last qualifying day) of the assigned regimen, inclusive. A qualifying day is defined as a day on which treatment was taken with at least 50% of the protocol-mandated dose of all drugs in the assigned regimen (exceptions and detailed definition are given in the protocol and statistical analysis plan).

† Serious adverse events considered at least possibly related to anti-tuberculosis medication in the standard treatment group were vomiting, drug-induced liver injury, and gout (each in one participant); in the rifampicin-linezolid group were vomiting (two participants) and drug-induced liver injury, acute hepatic failure (fatal) and anaphylactic reaction (each in one participant); in the rifampicin-clofazimine group were vomiting, jaundice, drug-induced liver injury and hepatitis (each in one participant); in the rifapentine-linezolid group were vomiting and hepatotoxicity (as two separate events in one participant) and vomiting (in one participant); and in the bedaquiline-linezolid group were anemia (two participants) and vomiting (in one participant).

Table S11 TREATMENT-LIMITING ADVERSE EVENTS

Adverse event (maximum grade)	Treatment received before adverse event onset (days) *	Treatment received before dose reduced or regimen ceased (days) *	Impact
**Standard regimen**			
Cardiac arrest (4, fatal)	144	144	Regimen cessation
Stroke (4, fatal)	142	143	Regimen cessation
Increased ALT (2)	34	34	Regimen cessation
Gout (4)	37	41	Regimen cessation
**Rifampicin-linezolid regimen**			
Vomiting (3) †	3	3	Regimen cessation
Vomiting (2)	11	11	Regimen cessation
Vomiting (3) and increased bilirubin (3) †	6	6	Regimen cessation
Acute hepatic failure (4, fatal) †	41	41	Regimen cessation
Decreased appetite (3) †	11	12	Regimen cessation
Dizziness (3) †	6	7	Regimen cessation
Headache (2) †	1	5	Regimen cessation
Rash (2) with ocular hyperaemia (1)	26	30	Regimen cessation
Increased ALT (3) †	6	43	Rifampicin reduction (to 450mg, 10mg/kg)
**Rifampicin-clofazimine regimen**			
Rash (2)	1	7	Regimen cessation
Rash (1)	42	42	Regimen cessation
Dizziness (3), Hepatitis (4)	27	27	Regimen cessation
Jaundice (4)	8	8	Regimen cessation
Vomiting (2)	2	3	Regimen cessation
Nausea (1), dizziness (1), increased bilirubin (3)	2	23	Rifampicin reduction (to 600mg, 10mg/kg)
Increased bilirubin (2)	7	7	Rifampicin reduction (to 1050mg, 25mg/kg)
Vomiting (2)	8	11	Rifampicin reduction (to 1500mg, 30mg/kg)
**Rifapentine-linezolid regimen**			
Vomiting (2)	1	1	Regimen cessation
Vomiting (2)	1	1	Regimen cessation
Vomiting (2) and nausea (3)	1	8	Regimen cessation

Vomiting (2) and nausea (2)	2	4	Regimen cessation
Vomiting (3) and headache (3)	5	11	Regimen cessation
Nausea (1) and arthralgia (2)	3	10	Regimen cessation
Hypersensitivity (2)	20	20	Regimen cessation
Hypersensitivity (1)	25	25	Regimen cessation
Vomiting (1)	1	40	Rifapentine reduction (to 900mg)
Nausea (3)	1	8	Rifapentine reduction (to 900mg)
**Bedaquiline-linezolid regimen**			
Tachycardia (1), ECG repolarisation abnormality (1)	8	10	Regimen cessation
QTc prolongation (3)	41	41	Regimen cessation
Palpitations (2), insomnia (2)	1	1	Regimen cessation
Anaemia (4)	42	42	Regimen cessation
Anaemia (3)	43	44	Linezolid reduction (to 0mg) ‡
Anaemia (3)	32	32	Linezolid reduction (to 300mg)
Arthralgia (2)	15	28	Pyrazinamide reduction (to 500mg, 10mg/kg)
Vomiting (2)	6	23	Linezolid reduction (to 300mg)
Anaemia (3)	42	43	Linezolid reduction (to 300mg)

Table lists individual participants that had an incident adverse event that was treatment-limiting. Incident adverse events are events with start date or date of increase in grade occurring in the period between the day of the baseline visit and 30 days following the day of discontinuation of the assigned regimen, inclusive. A treatment-limiting adverse event is an event that leads to permanent dose reduction or cessation of the assigned regimen prior to the completion of at least 154 qualifying days of standard treatment (for participants assigned to the standard treatment group) or at least 54 qualifying days of the assigned regimen (for participants in the 8-week regimen groups). A qualifying day is defined as a day on which the participant received at least 50% of the protocol-specified dose of all drugs in the assigned regimen (exceptions and detailed definition in protocol and statistical analysis plan). An additional 7 participants in the standard treatment group, 4 participants in the rifampicin-linezolid group, 1 participant in the rifampicin-clofazimine group, 1 participant in the rifapentine-linezolid group and 3 participants in the bedaquiline-linezolid group who were not otherwise counted as having a treatment-limiting adverse event had an adverse event leading to temporary interruption of one or more drugs in the assigned regimen.

* Number of days of treatment taken before adverse event onset and before permanent dose reduction or cessation refers to qualifying days of treatment, and includes treatment taken on the day of adverse event onset (if that meets the definition of a qualifying day of treatment). Events were graded using Division of AIDS Toxicity Criteria. Adverse event onset is the first day when the adverse event was reported at any grade; the maximum grade may have occurred later.
† Participant assigned a starting dose of rifampicin 35mg/kg (versus assigned a starting dose of rifampicin 20mg/kg)
‡ Cessation of linezolid for toxicity after receiving 42 days of linezolid was permitted by the protocol, with continuation of the remaining four drugs in the regimen to complete the required number of qualifying days. This participant continued other drugs to complete 56 qualifying days of the assigned regimen after cessation of linezolid, so this is regarded as dose reduction of linezolid (to 0mg) rather than regimen cessation.

Table S12: HIGH ALT AND HIGH BILIRUBIN (LABORATORY REPORTS)

	Standard treatment 24-week regimen (n=181)	Rifampicin-linezolid 8-week regimen (n=184)	Rifampicin-clofazimine 8-week regimen (n=78)	Rifapentine-linezolid 8-week regimen (n=42)	Bedaquiline-linezolid 8-week regimen (n=189)
**High ALT**					
Any grade	28 (15%)	48 (26%)	16 (21%)	2 (5%)	37 (20%)
Grade 1 (1.25 - 2.5 x ULN)	13 (7%)	35 (19%)	10 (13%)	2 (5%)	31 (16%)
Grade 2 (2.6 - 5 x ULN)	9 (5%)	11 (6%)	3 (4%)	0	4 (2%)
Grade 3 (5.1 - 10 x ULN)	5 (3%)	1 (1%)	2 (3%)	0	2 (1%)
Grade 4 (> 10 x ULN)	1 (1%)	1 (1%)	1 (1%)	0	0
**High bilirubin**					
Any grade	17 (9%)	43 (23%)	42 (54%)	27 (64%)	5 (3%)
Grade 1 (1.1-1.5 x ULN)	11 (6%)	25 (14%)	27 (35%)	14 (33%)	4 (2%)
Grade 2 (1.6-2.5 x ULN)	2 (1%)	13 (7%)	12 (15%)	9 (21%)	1 (1%)
Grade 3 (2.6-5 x ULN)	4 (2%)	4 (2%)	1 (1%)	4 (10%)	0
Grade 4 (>5 x ULN)	0	1 (1%)	2 (3%)	0	0
**Hy's Law**					
ALT > 3 x ULN and Bilirubin > 2 x ULN	2 (1%)	2 (1%)	2 (3%)	0	0

Data are n (%). Table shows the number of participants in each treatment group with incident high ALT or high bilirubin or both. Incident events are those with start date or increase in grade occurring in the period between the day of the baseline visit and 30 days following the day of cessation (last qualifying day) of the assigned regimen, inclusive. A qualifying day is defined as a day on which treatment was taken with at least 50% of the protocol-mandated dose of all drugs in the assigned regimen (exceptions and detailed definition are given in the protocol and statistical analysis plan).

High ALT, high bilirubin and Hy's law determined centrally from protocol-mandated laboratory tests done at scheduled visits, irrespective of clinical reporting as an adverse event; and from tests done at other visits that were noted on clinical reports for grade 3 or higher adverse events or serious adverse events (limited to events reported with a preferred term under the MedDRA hepatic disorders SMQ). Each participant is classified by the highest grade of incident ALT and bilirubin observed, based on Division of AIDS Toxicity Criteria; these values could be from tests done on different days. Hy's law criteria (ALT > 3 and bilirubin > 2 times upper limit of normal) are not classified by grade in Division of AIDS Toxicity Criteria; to meet Hy's law criteria, the ALT and bilirubin values were required to be from a test done on the same day.

Table S13 LOW HAEMOGLOBIN, NEUTROPHIL COUNT AND PLATELET COUNT (LABORATORY REPORTS)

	Standard treatment 24-week regimen (n=181)	Rifampicin-linezolid 8-week regimen (n=184)	Rifampicin-clofazimine 8-week regimen (n=78)	Rifapentine-linezolid 8-week regimen (n=42)	Bedaquiline-linezolid 8-week regimen (n=189)
**Low haemoglobin level**					
Any grade	30 (17%)	29 (16%)	8 (10%)	12 (29%)	56 (30%)
Grade 1 (10-10.9 g/dL)	21 (12%)	22 (12%)	5 (6%)	8 (19%)	29 (15%)
Grade 2 (9-9.9 g/dL)	4 (2%)	5 (3%)	3 (4%)	2 (5%)	13 (7%)
Grade 3 (7-8.9 g/dL)	5 (3%)	2 (1%)	0	2 (5%)	10 (5%)
Grade 4 (<7 g/dL)	0	0	0	0	4 (2%)
**Low neutrophil count**					
Any grade	15 (8%)	12 (7%)	4 (5%)	2 (5%)	8 (4%)
Grade 1 (1-1.3 x 10^9/L)	6 (3%)	11 (6%)	3 (4%)	2 (5%)	5 (3%)
Grade 2 (0.75-0.999 x 10^9/L)	6 (3%)	0	1 (1%)	0	3 (2%)
Grade 3 (0.5-0.749 x 10^9/L)	2 (1%)	1 (1%)	0	0	0
Grade 4 (<0.5 x 10^9/L)	1 (1%)	0	0	0	0
**Low platelet count**					
Any grade	4 (2%)	2 (1%)	0	0	4 (2%)
Grade 1 (100-124.999 x 10^9/L)	4 (2%)	0	0	0	3 (2%)
Grade 2 (50-99.999 x 10^9/L)	0	2 (1%)	0	0	1 (1%)
Grade 3 (25-49.999 x 10^9/L)	0	0	0	0	0
Grade 4 (<25 x 10^9/L)	0	0	0	0	0

Data are n (%). Table shows number of participants in each treatment group with incident low haemoglobin, low neutrophil count or low platelet count. Incident events are those with start date or increase in grade occurring in the period between the day of the baseline visit and 30 days following the day of cessation (last qualifying day) of the assigned regimen, inclusive. A qualifying day is defined as a day on which treatment was taken with at least 50% of the protocol-mandated dose of all drugs in the assigned regimen (exceptions and detailed definition are given in the protocol and statistical analysis plan). Low haemoglobin, neutrophil count or platelet count determined centrally from protocol-mandated full blood count tests done at scheduled visits, irrespective of clinical reporting as an adverse event; or from tests done at other visits that were noted on clinical reports for grade 3 or higher adverse events or serious adverse events (limited to events reported with a MedDRA preferred term of anaemia, neutropenia or thrombocytopenia or corresponding laboratory term). Each participant is classified by the highest grade of incident low haemoglobin, neutrophil count or platelet count observed, based on Division of AIDS Toxicity Criteria; these values could be from tests done on different days.

Table S14 GRADE 3-4, SERIOUS AND TREATMENT-LIMITING ADVERSE EVENTS BY ASSIGNED RIFAMPICIN DOSE IN THE RIFAMPICIN-LINEZOLID GROUP

	**Period 1**		**Period 2**	
	**Standard treatment 24-week regimen (n=91)**	**Rifampicin (35mg/kg)-linezolid 8-week regimen (n=88)**	**Standard treatment 24-week regimen (n=90)**	**Rifampicin (20mg/kg)-linezolid 8-week regimen (n=96)**
**Participants with at least one adverse event of grade 3-4 severity**				
Any	11 (12%)	15 (17%)	14 (16%)	5 (5%)
Related to anti-tuberculosis medication*	7 (8%)	10 (11%)	6 (7%)	1 (1%)
**Participants with at least one serious adverse event**				
Any	4 (4%)	6 (7%)	3 (3%)	2 (2%)
Related to anti-tuberculosis medication †	3 (3%)	5 (6%)	0	0
**Participants with at least one adverse event of any grade**				
Any	72 (79%)	81 (92%)	73 (81%)	77 (80%)
Treatment-limiting ‡	1 (1%)	7 (8%)	3 (3%)	2 (2%)

Data are n (%). Table shows number of participants with at least one incident adverse event in each event category for participants 1-88 (period 1, assigned 35mg/kg rifampicin) and 89-184 (period 2, assigned 20mg/kg rifampicin) enrolled in the rifampicin-linezolid group and for participants who underwent contemporary randomisation to the standard treatment group during these periods.

Incident adverse events are events with start date or date of increase in grade occurring in the period between the day of the baseline visit and 30 days following the day of cessation (last qualifying day) of the assigned regimen, inclusive. A qualifying day is defined as a day on which treatment was taken with at least 50% of the protocol-mandated dose of all drugs in the assigned regimen (exceptions and detailed definition are given in the protocol and statistical analysis plan). Events were graded using Division of AIDS Toxicity Criteria.

* Indicated by the local investigator as at least possibly related to one of more of the anti-tuberculosis drugs in the assigned regimen.

† Serious adverse events considered at least possibly related to anti-tuberculosis medication in the standard treatment group were vomiting, drug-induced liver injury, and gout (each in one participant), all in period 1; and in the rifampicin-linezolid group were vomiting (two participants) and drug-induced liver injury, acute hepatic failure (fatal) and anaphylactic reaction (each in one participant), all in period 1.

‡ Adverse event causing permanent dose reduction of one or more drugs in the assigned regimen or permanent cessation of the assigned regimen before completion of at least 54 qualifying days (for the 8-week rifamicin-linezolid regimen) or 154 qualifying days (for the standard 24-week regimen).

Table S15 ADVERSE EVENTS OF SPECIAL INTEREST AND COMMON ADVERSE EVENTS, BY ASSIGNED RIFAMPICIN DOSE IN THE RIFAMPICIN-LINEZOLID GROUP

	**Period 1**				**Period 2**			
	**Standard treatment 24-week regimen (n=91)**		**Rifampicin(35mg/kg)-linezolid 8-week regimen (n=88)**		**Standard treatment 24-week regimen (n=90)**		**Rifampicin (20mg/kg)-linezolid 8-week regimen (n=96)**	
	Any grade	G3-4	Any grade	G3-4	Any grade	G3-4	Any grade	G3-4
**Adverse events of special interest (pre-specified)**								
Hepatic events								
Hepatic disorders (SMQ)	11 (12%)	5 (5%)	27 (31%)	6 (7%)	12 (13%)	4 (4%)	17 (18%)	0
Biliary disorders (SMQ)	4 (4%)	1 (1%)	21 (24%)	3 (3%)	2 (2%)	0	6 (6%)	0
High ALT *	11 (12%)	3 (3%)	25 (28%)	2 (2%)	17 (19%)	3 (3%)	23 (24%)	0
High bilirubin *	13 (14%)	4 (4%)	32 (36%)	5 (6%)	4 (4%)	0	11 (11%)	0
Hy's law *	2 (2%)	-	2 (2%)	-	0	-	0	-
Haematological events								
Anaemia	4 (4%)	2 (2%)	5 (6%)	2 (2%)	3 (3%)	3 (3%)	2 (2%)	0
Haematopoietic cytopenias (SMQ)	4 (4%)	0	5 (6%)	0	4 (4%)	3 (3%)	2 (2%)	1 (1%)
Low haemoglobin †	14 (15%)	2 (2%)	18 (20%)	2 (2%)	16 (18%)	3 (3%)	11 (11%)	0
Low neutrophil count †	3 (3%)	0	4 (5%)	0	12 (13%)	3 (3%)	8 (8%)	1 (1%)
Other								
Peripheral neuropathy (SMQ)	4 (4%)	0	3 (3%)	0	10 (11%)	0	12 (12%)	0
Optic nerve disorders (SMQ)	0	0	0	0	0	0	0	0
Skin hyperpigmentation/discoloration	0	0	1 (1%)	0	0	0	2 (2%)	0
Electrocardiogram QTc prolongation	0	0	2 (2%)	0	2 (2%)	0	0	0
**Additional adverse events occurring in ≥ 10% of participants in one or more groups**								
Dizziness	3 (3%)	0	12 (14%)	1 (1%)	2 (2%)	0	8 (8%)	0
Headache	2 (2%)	0	14 (16%)	0	3 (3%)	0	8 (8%)	0
Abdominal pain	11 (12%)	0	25 (28%)	0	10 (11%)	0	8 (8%)	0
Nausea	13 (14%)	0	29 (33%)	0	4 (4%)	0	14 (15%)	0
Vomiting	15 (16%)	1 (1%)	40 (45%)	2 (2%)	8 (9%)	0	20 (21%)	0
Pruritus	12 (13%)	0	11 (12%)	0	11 (12%)	0	5 (5%)	0
Arthralgia	16 (18%)	0	5 (6%)	0	20 (22%)	0	15 (16%)	0

Data are n (%). Table shows number of participants with at least one incident adverse event in each event category for participants 1-88 (period 1, assigned 35mg/kg rifampicin) and 89-184 (period 2, assigned 20mg/kg rifampicin) enrolled in the rifampicin-linezolid group and for participants who underwent contemporary randomisation to the standard treatment group during these periods.

Incident adverse events are events with start date or date of increase in grade occurring in the period between the day of the baseline visit and 30 days following the day of cessation (last qualifying day) of the assigned regimen, inclusive. A qualifying day is defined as a day on which treatment was taken with at least 50% of the protocol-mandated dose of all drugs in the assigned regimen (exceptions and detailed definition are given in the protocol and statistical analysis plan). Adverse events of special interest were pre-specified. Standard TB-related symptoms (cough, haemoptysis, pleuritic chest pain, fever, night sweats, weight loss) were not considered as incident adverse events in this analysis.

Events were graded using Division of AIDS Toxicity Criteria, and participants classified by the highest grade observed. Events were coded using the Medical Dictionary for Regulatory Activities (MedDRA); listed by single MedDRA Preferred Term, or as a prespecified combination of terms (skin hyperpigmentation or discoloration), or as a composite of terms obtained using a Standardised MedDRA Query (SMQ).

* High ALT, high bilirubin and Hy's law determined centrally from protocol-mandated laboratory tests done at scheduled visits, irrespective of clinical reporting as an adverse event; and from tests done at other visits that were noted on clinical reports for grade 3 or higher adverse events or serious adverse events (limited to events reported with a preferred term under the MedDRA hepatic disorders SMQ). Hy's law criteria (ALT > 3 and bilirubin > 2 times upper limit of normal) are not classified by grade in Division of AIDS Toxicity Criteria.

† Low haemoglobin and low neutrophil count determined centrally from protocol-mandated laboratory tests done at scheduled visits, irrespective of clinical reporting as an adverse event; or from tests done at other visits that were noted on clinical reports for grade 3 or higher adverse events or serious adverse events (limited to events reported with a MedDRA preferred term of anaemia, neutropenia or thrombocytopenia or corresponding laboratory term).

Table S16 HIGH ALT AND HIGH BILIRUBIN (LABORATORY REPORTS), BY ASSIGNED RIFAMPICIN DOSE IN THE RIFAMPICIN-LINEZOLID GROUP

	Period 1		Period 2	
	**Standard treatment 24-week regimen (n=91)**	**Rifampicin(35mg/kg) -linezolid 8-week regimen (n=88)**	**Standard treatment 24-week regimen (n=90)**	**Rifampicin (20mg/kg)-linezolid 8-week regimen (n=96)**
**High ALT**				
Any grade	11 (12%)	25 (28%)	17 (19%)	23 (24%)
Grade 1 (1.25 - 2.5 x ULN)	3 (3%)	20 (23%)	10 (11%)	15 (16%)
Grade 2 (2.6 - 5 x ULN)	5 (5%)	3 (3%)	4 (4%)	8 (8%)
Grade 3 (5.1 - 10 x ULN)	2 (2%)	1 (1%)	3 (3%)	0
Grade 4 (> 10 x ULN)	1 (1%)	1 (1%)	0	0
**High bilirubin**				
Any grade	13 (14%)	32 (36%)	4 (4%)	11 (11%)
Grade 1 (1.1-1.5 x ULN)	7 (8%)	18 (20%)	4 (4%)	7 (7%)
Grade 2 (1.6-2.5 x ULN)	2 (2%)	9 (10%)	0	4 (4%)
Grade 3 (2.6-5 x ULN)	4 (4%)	4 (5%)	0	0
Grade 4 (>5 x ULN)	0	1 (1%)	0	0
**Hy's Law**				
ALT > 3 x ULN and Bilirubin > 2 x ULN	2 (2%)	2 (2%)	0	0

Data are n (%). Table shows number of participants with incident high ALT or high bilirubin or both for participants 1-88 (period 1, assigned 35mg/kg rifampicin) and 89-184 (period 2, assigned 20mg/kg rifampicin) enrolled in the rifampicin-linezolid group and for participants who underwent contemporary randomisation to the standard treatment group during these periods. Incident high ALT or high bilirubin are events with start date or date of increase in grade occurring in the period between the day of the baseline visit and 30 days following the day of cessation (last qualifying day) of the assigned regimen, inclusive. A qualifying day is defined as a day on which treatment was taken with at least 50% of the protocol-mandated dose of all drugs in the assigned regimen (exceptions and detailed definition are given in the protocol and statistical analysis plan). High ALT, high bilirubin and Hy's law determined centrally from protocol-mandated laboratory tests done at scheduled visits, irrespective of clinical reporting as an adverse event; and from tests done at other visits that were noted on clinical reports for grade 3 or higher adverse events or serious adverse events (limited to events reported with a preferred term under the MedDRA hepatic disorders SMQ). Each participant is classified by the highest grade of incident ALT and bilirubin observed, based on Division of AIDS Toxicity Criteria; these values could be from tests done on different days. Hy's law criteria (ALT > 3 and bilirubin > 2 times upper limit of normal) are not classified by grade in Division of AIDS Toxicity Criteria; to meet Hy's law criteria, the ALT and bilirubin values were required to be from a test done on the same day.

S17 LOW HAEMOGLOBIN, NEUTROPHIL COUNT AND PLATELET COUNT (LABORATORY REPORTS) BY ASSIGNED RIFAMPICIN DOSE IN THE RIFAMPICIN-LINEZOLID GROUP

	Period 1		Period 2	
	**Standard treatment 24-week regimen (n=91)**	**Rifampicin (35mg/kg)-linezolid 8-week regimen (n=88)**	**Standard treatment 24-week regimen (n=90)**	**Rifampicin (20mg/kg)-linezolid 8-week regimen (n=96)**
**Low haemoglobin level**				
Any grade	14 (15%)	18 (20%)	16 (18%)	11 (11%)
Grade 1 (10-10.9 g/dL)	11 (12%)	14 (16%)	10 (11%)	8 (8%)
Grade 2 (9-9.9 g/dL)	1 (1%)	2 (2%)	3 (3%)	3 (3%)
Grade 3 (7-8.9 g/dL)	2 (2%)	2 (2%)	3 (3%)	0
Grade 4 (<7 g/dL)	0	0	0	0
**Low neutrophil count**				
Any grade	3 (3%)	4 (5%)	12 (13%)	8 (8%)
Grade 1 (1-1.3 x 10^9/L)	2 (2%)	4 (5%)	4 (4%)	7 (7%)
Grade 2 (0.75-0.999 x 10^9/L)	1 (1%)	0	5 (6%)	0
Grade 3 (0.5-0.749 x 10^9/L)	0	0	2 (2%)	1 (1%)
Grade 4 (<0.5 x 10^9/L)	0	0	1 (1%)	0
**Low platelet count**				
Any grade	1 (1%)	1 (1%)	3 (3%)	1 (1%)
Grade 1 (100-124.999 x 10^9/L)	1 (1%)	0	3 (3%)	0
Grade 2 (50-99.999 x 10^9/L)	0	1 (1%)	0	1 (1%)
Grade 3 (25-49.999 x 10^9/L)	0	0	0	0
Grade 4 (<25 x 10^9/L)	0	0	0	0

Data are n (%). Table shows number of participants in each treatment group with incident low haemoglobin, low neutrophil count or low platelet count with onset or increased grade occurring on or after the baseline day until 30 days following the day of cessation (last qualifying day) of the assigned regimen, inclusive. Low haemoglobin, neutrophil count or platelet count determined centrally from protocol-mandated full blood count tests done at scheduled visits, irrespective of clinical reporting as an adverse event; or from tests done at other visits that were noted on clinical reports for grade 3 or higher adverse events or serious adverse events (limited to events reported with a MedDRA preferred term of anaemia, neutropenia or thrombocytopenia or corresponding laboratory term). Each participant is classified by the highest grade of incident low haemoglobin, neutrophil count or platelet count observed, based on Division of AIDS Toxicity Criteria.

Table S18. QTcF PROLONGATION

	Standard treatment 24-week regimen (n=181)	Rifampicin-linezolid 8-week regimen (n=184)	Rifampicin-clofazimine 8-week regimen (n=78)	Rifapentine-linezolid 8-week regimen (n=42)	Bedaquiline-linezolid 8-week regimen (n=189)
Maximum QTcF, ms *	413.0 ± 19.0	414.4 ± 16.9	436.3 ± 22.1	415.8 ± 20.1	421.1 ± 19.7
Maximum QTcF, increase, ms †	19.1 ± 19.8	17.1 ± 18.8	33.3 ± 22.4	17.0 ± 24.0	23.8 ± 20.6
Maximum QTcF, increase ≥ 60ms ‡	7/181 (4%)	4/184 (2%)	11/78 (14%)	1/42 (2%)	10/188 (5%)
QTcF ≥ 500 ms (confirmed) §	0	0	0	0	0

Data are mean ± SD and n (%). QTcF is from machine readings at screening, and from scheduled and unscheduled visits from baseline day until 30 days following the day of cessation (last qualifying day) of the assigned regimen, inclusive. A qualifying day is defined as a day on which treatment was taken with at least 50% of the protocol-mandated dose of all drugs in the assigned regimen (exceptions and detailed definition are given in the protocol and statistical analysis plan). When two or more ECG readings were performed on the same day, the mean value was used for that day. One participant in the bedaquiline-linezolid group did not have any post-baseline QTcF values (participant switched to standard treatment after the first dose of the assigned regimen and completed treatment at a local clinic).

* Maximum QTcF was the highest QTcF value obtained in the period following the baseline day until 30 days after discontinuation of the assigned regimen.

† Maximum QTcF increase was the difference between the maximum QTcF and the QTcF value at screening (value from baseline used if QTcF not measured at screening).

‡ Maximum QTcF increases above stipulated (DAIDS) thresholds were not counted as QTcF prolongation in the adverse event tables (adverse events were based only on absolute QTcF values above thresholds).

§ QTcF ≥500 (confirmed) is defined as ≥ 2 machine readings of ≥ 500ms on the same day. No participants met this criterion. Three participants had a single reading ≥500ms. One participant in the standard treatment group had QTcF ≥ 500ms at week 4; repeat on the same day showed QTcF < 500ms, with no further occurrence on treatment. One participant in the rifampicin-clofazimine group had QTcF ≥ 500ms at week 8; this was not repeated on the same day; treatment was stopped at week 8 (planned). One participant in the bedaquiline-linezolid group had QTcF ≥ 500ms at week 6; repeat on same day showed QTcF < 500ms; this resolved after interruption of the assigned regimen for 4 days but the participant was switched to standard treatment (event was reported as an adverse event of Grade 3 severity).

SUPPLEMENTARY FIGURES

Figure S1 TRIAL DESIGN

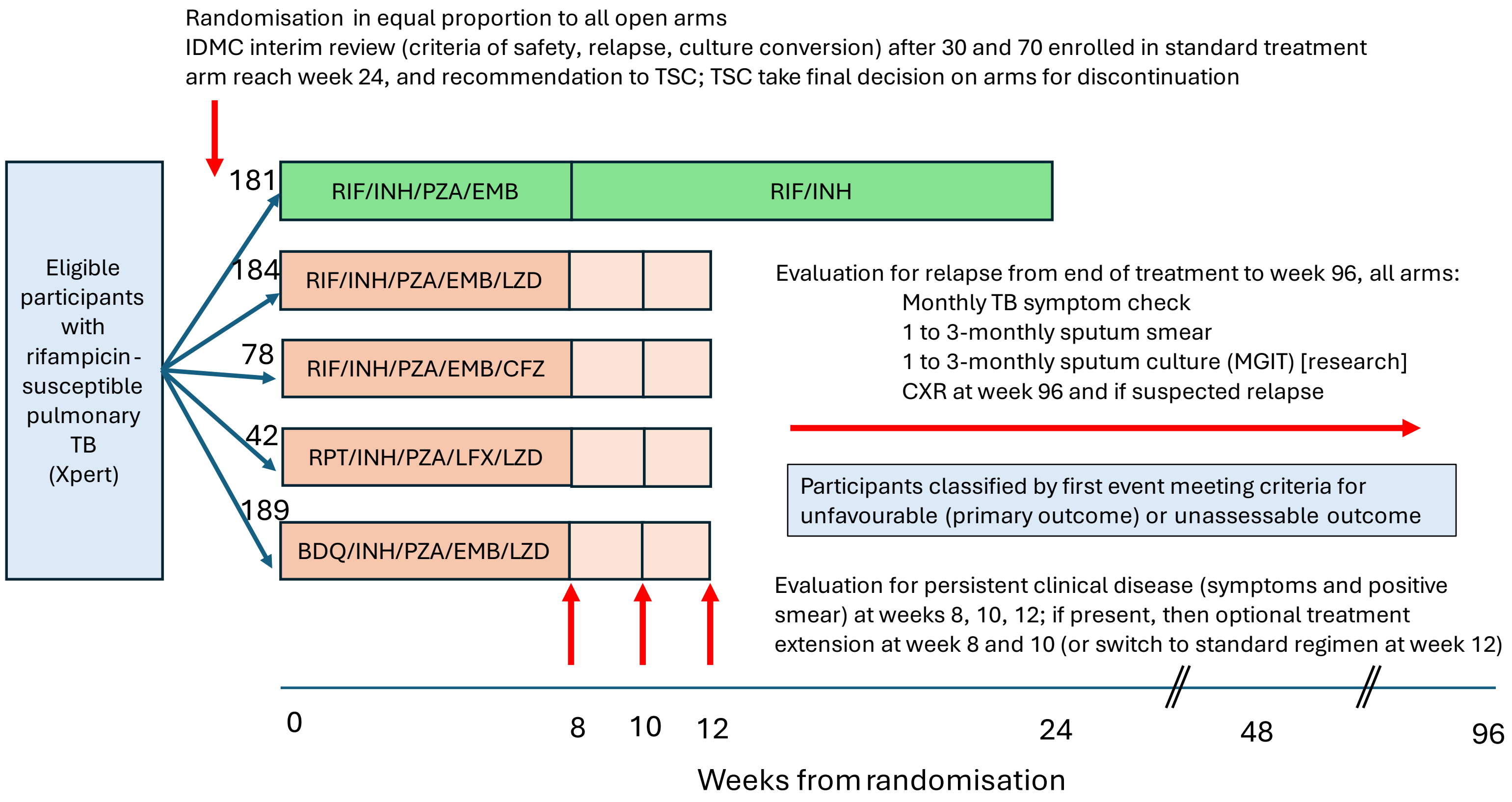

IDMC= independent data monitoring committee; TSC=trial steering committee; RIF=rifampicin; INH=isoniazid; PZA=pyrazinamide; EMB=ethambutol; LZD=linezolid; CFZ=clofazimine; RPT=rifapentine; LFX=levofloxacin; BDQ=bedaquiline

Figure S2 RELATIONSHIP BETWEEN MAIN OUTCOME CLASSIFICATION IN DRUG REGIMEN ANALYSIS AND TRUNCATE MANAGEMENT STRATEGY ANALYSES

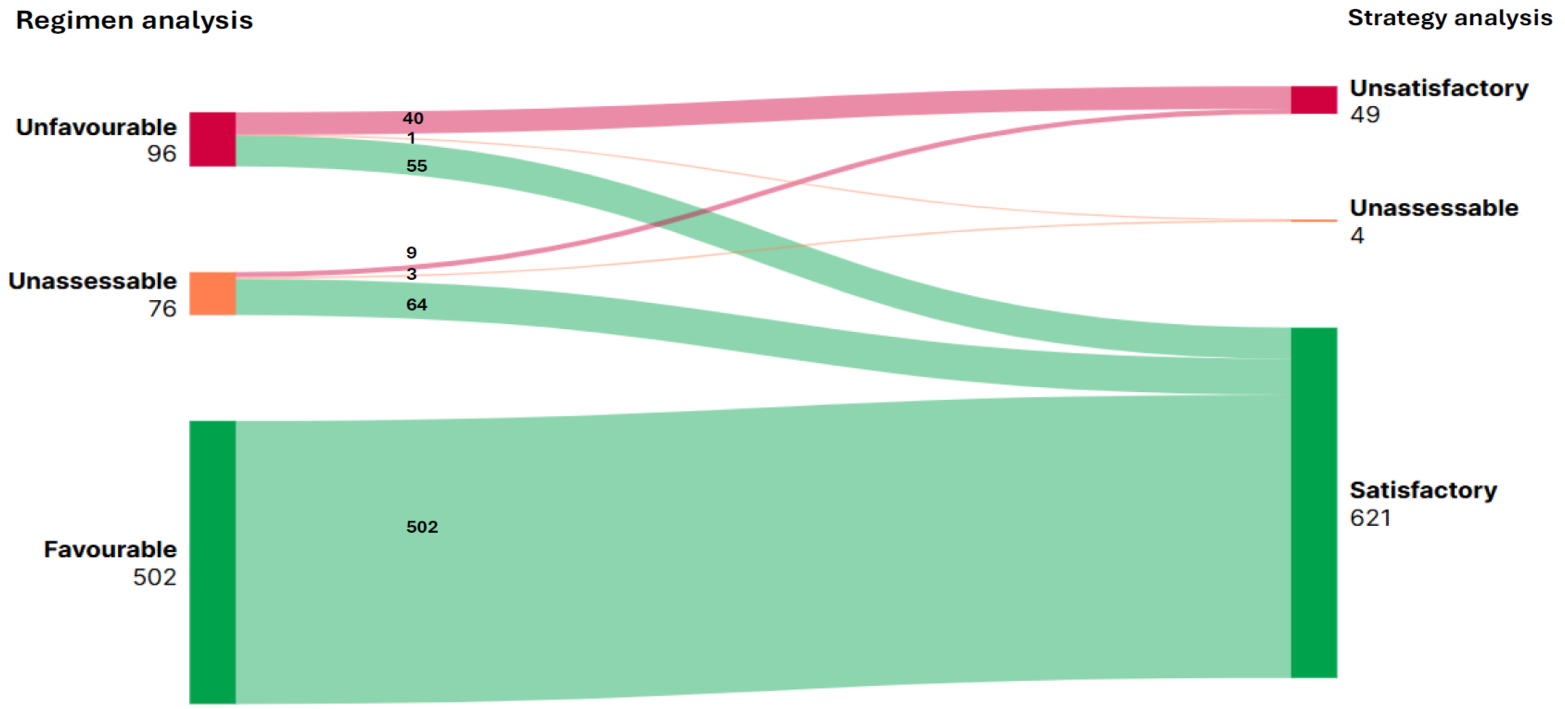

Sankey diagram showing the relationship between the classificaton of outcomes for the drug regimen efficacy analysis and TRUNCATE management strategy efficacy analysis (outcomes previously published). The primary outcome for the drug regimen efficacy analysis was unfavourable outcome which could occur at any time during follow-up from baseline to week 96. The primary outcome for the TRUNCATE management strategy anlaysis was unsatisfactory outcome, defined by clinical disease status at week 96. The majority (55/96) of the participants classified as unfavourable outcome on the drug regimen analysis were classified as satisfactory outcome on the TRUNCATE management strategy analysis because relapses (which accounted for most of the unfavourable outcomes on the regimen analysis but did not count as outcomes *per se* in the strategy analysis) had mostly been successfully re-treated by week 96 such that participants were no longer taking treatment and were free from active tuberculosis disease at week 96. The majority (64/76) of the participants classified as unassessable on the drug regimen analysis were classified as satisfactory outcome on the TRUNCATE management strategy analysis because initial treatment non-completion, non-adherence or treatment switches (which accounted for most of the unasessable outcomes on the regimen analysis) did not count as outcomes *per se* in the strategy analysis; and with subsequent adjustment to the treatment regimen most participants were no longer taking treatment and were free from active tuberculosis disease at week 96. A detailed comparison of outcome classifications between the drug regimen and TRUNCATE mangement strategy analyses is shown in Table S4.

Figure S3 TIME TO UNFAVOURABLE OUTCOME

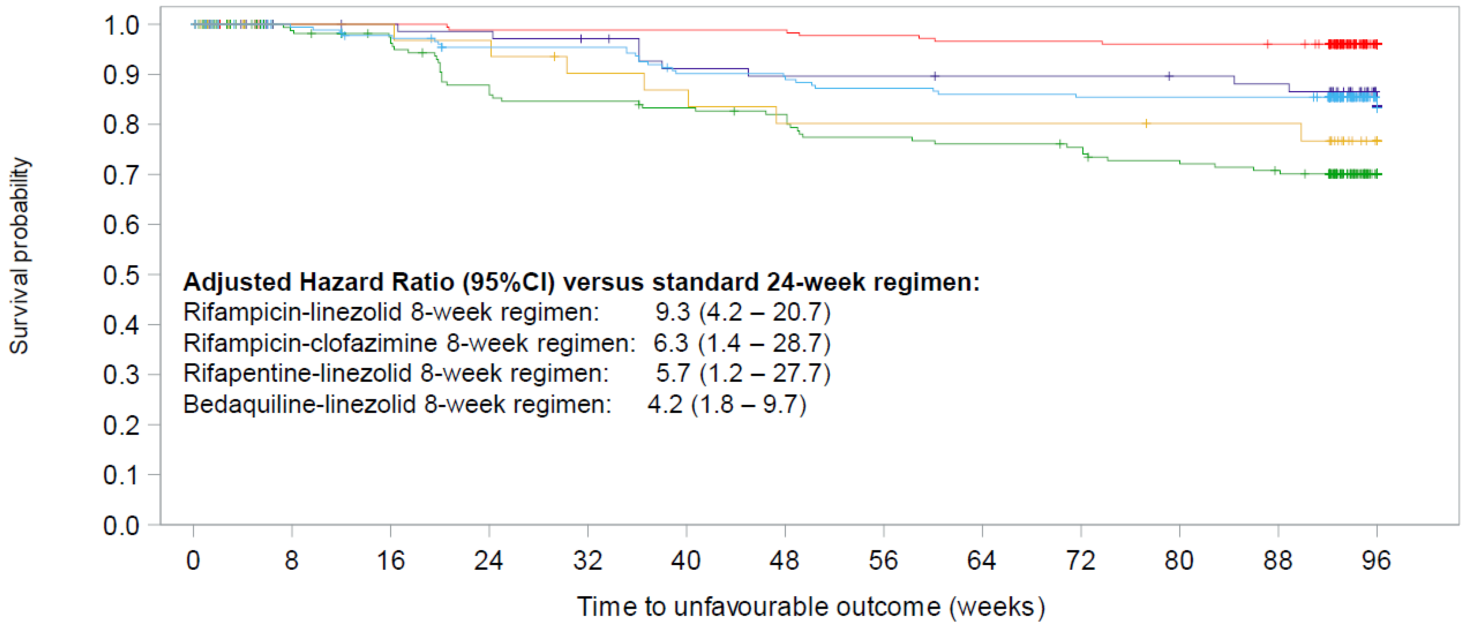

Participants at risk

	0	8	16	24	32	40	48	56	64	72	80	88	96
Standard treatment group	181	177	177	175	175	175	175	173	171	171	170	169	44
Rifampicin-linezolid group	184	159	152	136	131	128	125	118	116	114	109	105	29
Rifampicin-clofazimine group	78	70	69	68	66	61	60	60	59	59	58	57	30
Rifapentine-linezolid group	42	31	31	30	27	26	24	24	24	24	23	23	10
Bedaquiline-linezolid group	189	176	170	163	163	153	152	148	146	145	145	145	40

Standard treatment group (events: 7/181)
Rifampicin-linezolid group (events: 46/184)
Rifampicin-clofazimine group (events: 10/78)
Rifapentine-linezolid group (events: 7/42)
Bedaquiline-linezolid group (events: 26/189)

Kaplan-Meier curves of time to unfavourable outcome in the 5 treatment groups based on all participants enrolled in each group. Hazard ratios are for comparison of the 8-week regimens against the standard 24-week treatment regimen, estimated using stratified Cox regression models (starting at randomisation and ending at the earliest of the first event or the week 96 visit), with country and baseline relapse risk as the stratification factors. Hazard ratios for the rifampicin-linezolid and bedaquiline-linezolid groups were obtained from a model including all participants enrolled to these groups and the standard treatment group; the hazard ratio for the rifampicin-clofazimine group was obtained from a model including all participants in this group and those participants undergoing contemporary randomisation to the rifampicin-linezolid, bedaquiline-linezolid and standard treatment groups; and hazard ratio for the rifapentine-linezolid group was obtained from a model including all participants in this group and those participants undergoing contemporary randomisation to all other groups. Models and comparisons with standard treatment group were pre-specified as limited to the fully-enrolled 8-week groups; these comparisons were also performed as post-hoc analyses for the two groups in which enrollment was discontinued early.

Figure S4 TIME TO TREATMENT FAILURE OR RELAPSE

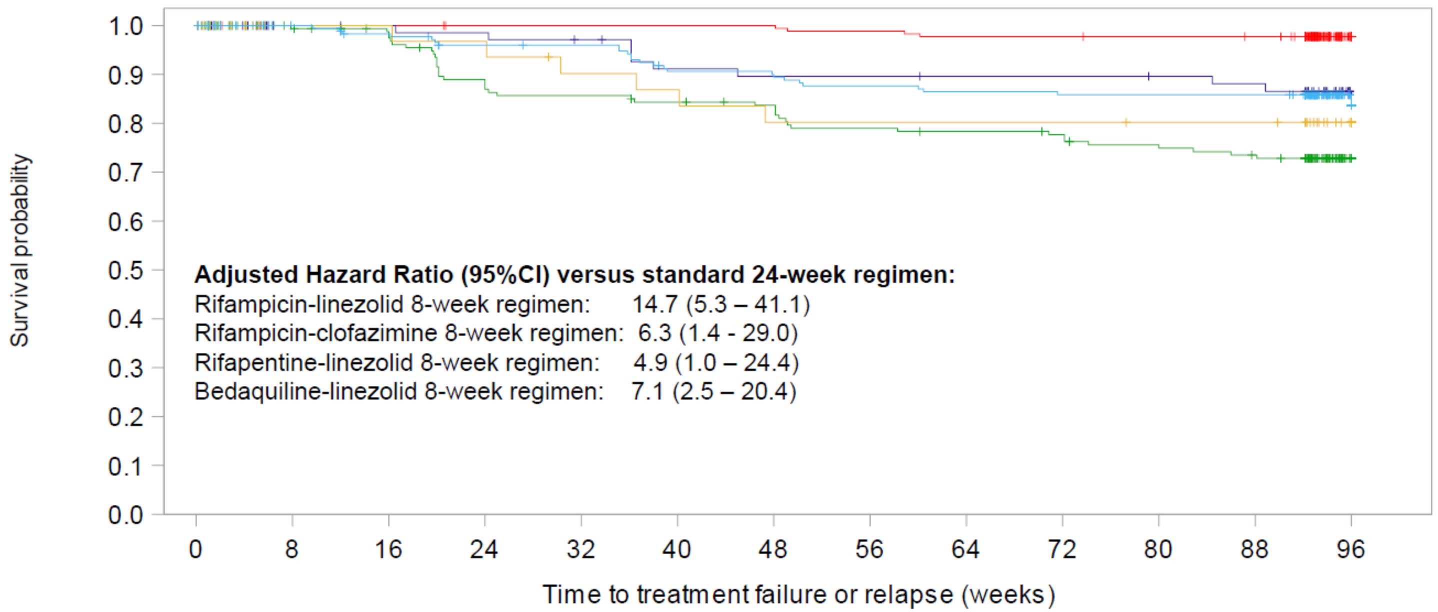

Participants at risk

Standard treatment group	181	177	177	175	175	175	175	173	171	171	170	169	44
Rifampicin-linezolid group	184	159	152	136	131	128	125	118	116	114	109	105	29
Rifampicin-clofazimine group	78	70	69	68	66	61	60	60	59	59	58	57	30
Rifapentine-linezolid group	42	31	31	30	27	26	24	24	24	24	23	23	10
Bedaquiline-linezolid group	189	176	170	163	162	152	151	147	145	144	144	144	39

Standard treatment group (events: 4/181)
Rifampicin-linezolid group (events: 41/184)
Rifampicin-clofazimine group (events: 10/78)
Rifapentine-linezolid group (events: 6/42)
Bedaquiline-linezolid group (events: 25/189)

Kaplan-Meier curves of time to treatment failure or relapse in the 5 treatment groups, based on all participants enrolled in each group. Hazard ratios are for comparison of the 8-week regimens against the standard 24-week treatment regimen, estimated using stratified Cox regression models (starting at randomisation and ending at the earliest of the first event or the week 96 visit), with country and baseline relapse risk as the stratification factors. Hazard ratios for the rifampicin-linezolid and bedaquiline-linezolid groups were obtained from a model including all participants enrolled to these groups and the standard treatment group; the hazard ratio for the rifampicin-clofazimine group was obtained from a model including all participants in this group and those participants undergoing contemporary randomisation to the rifampicin-linezolid, bedaquiline-linezolid and standard treatment groups; and hazard ratio for the rifapentine-linezolid group was obtained from a model including all participants in this group and those participants undergoing contemporary randomisation to all other groups. Models and comparisons with standard treatment group were pre-specified as limited to the fully-enrolled 8-week groups; these comparisons were also performed as post-hoc analyses for the two groups in which enrollment was discontinued early.

Figure S5 EVALUATION OF DRUG-INDUCED SERIOUS HEPATOTOXICITY (eDISH)

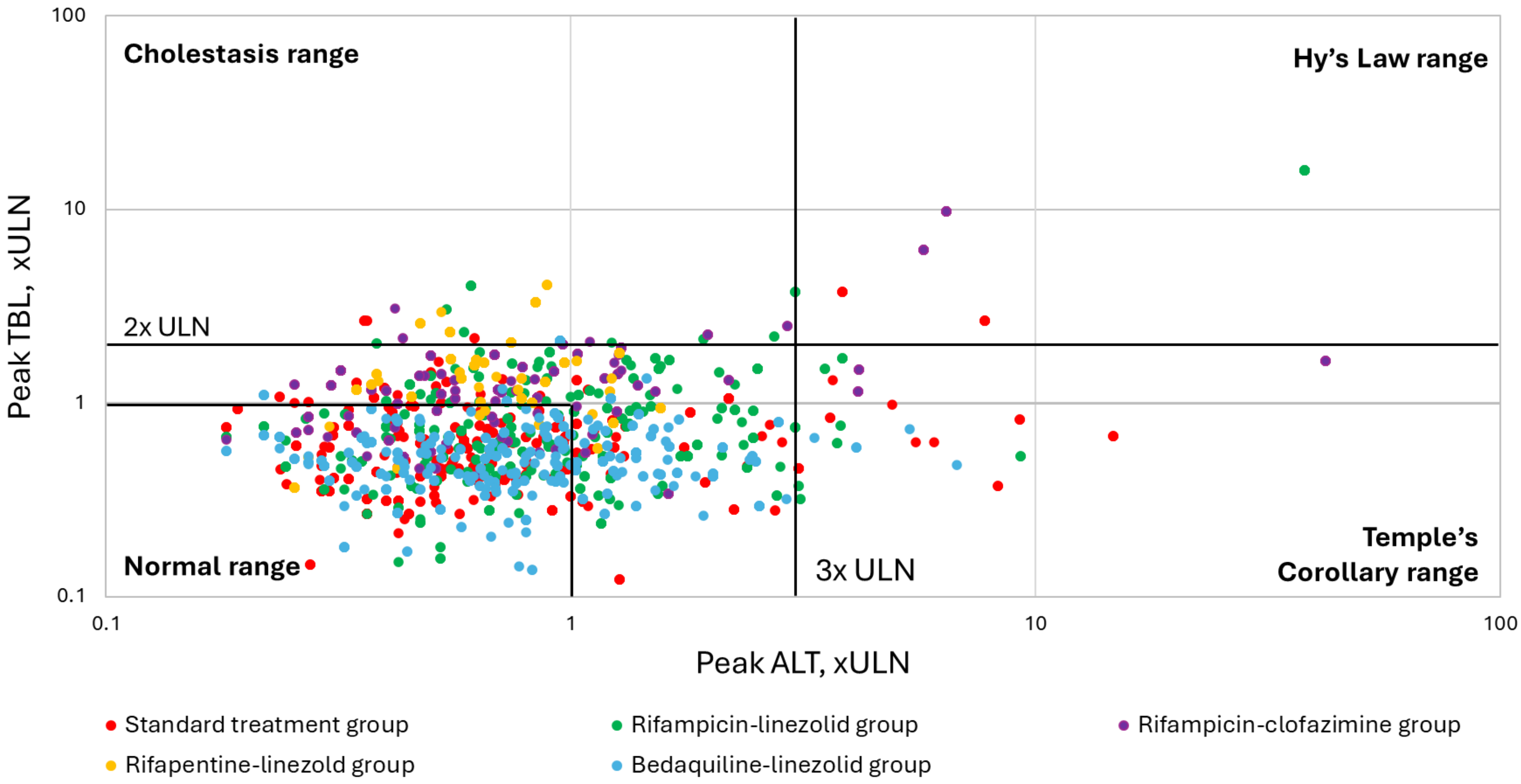

TBL = total bilirubin. ALT = alanine transaminase, ULN = upper limit of normal. One participant in the standard treatment group had a maximum value of ALT of 0.07 x ULN and bilirubin of 0.61 x ULN and is not shown on the figure.

Figure S6 PARTICIPANTS MEETING HY'S LAW BIOCHEMICAL CRITERIA

Participants in standard treatment group

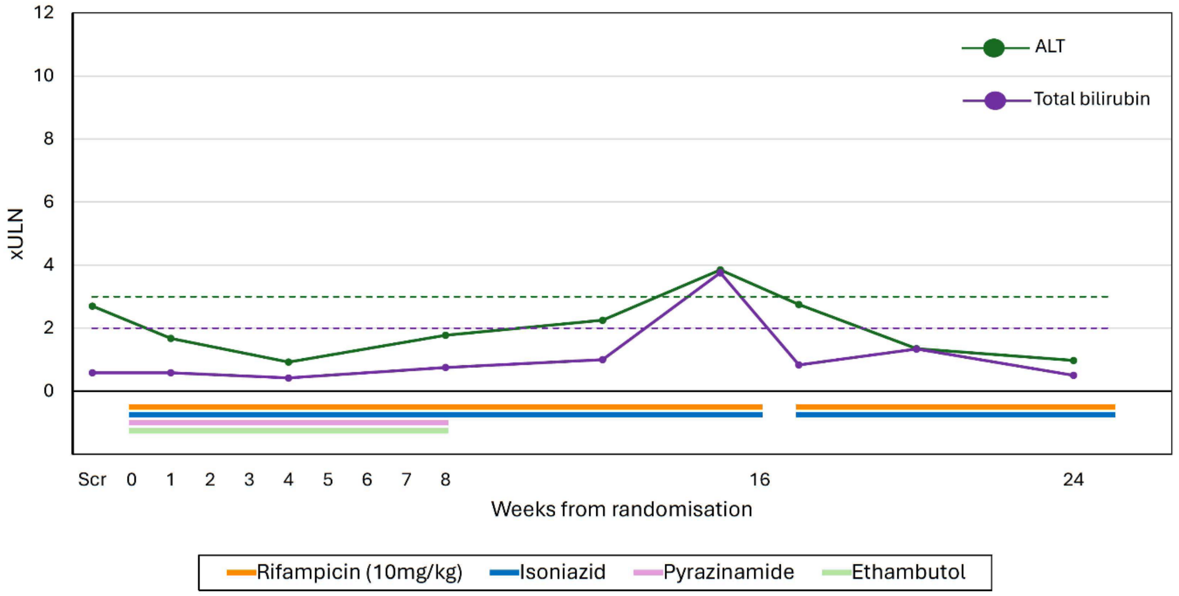

A) Participant in standard treatment group. Male, 40-50 years of age, with Type 2 diabetes on oral hypoglycaemic agents; alcohol consumption of 8-14 units per week. Rash and dyspepsia, onset week 16. Elevated ALT and total bilirubin meeting Hy's Law criteria at week 16; alkaline phosphatase not elevated. Hepatitis serology not done. Possible contributing causes: alcohol consumption. Hepatitis resolved with treatment interruption; treatment restarted after 7 days without recurrence.

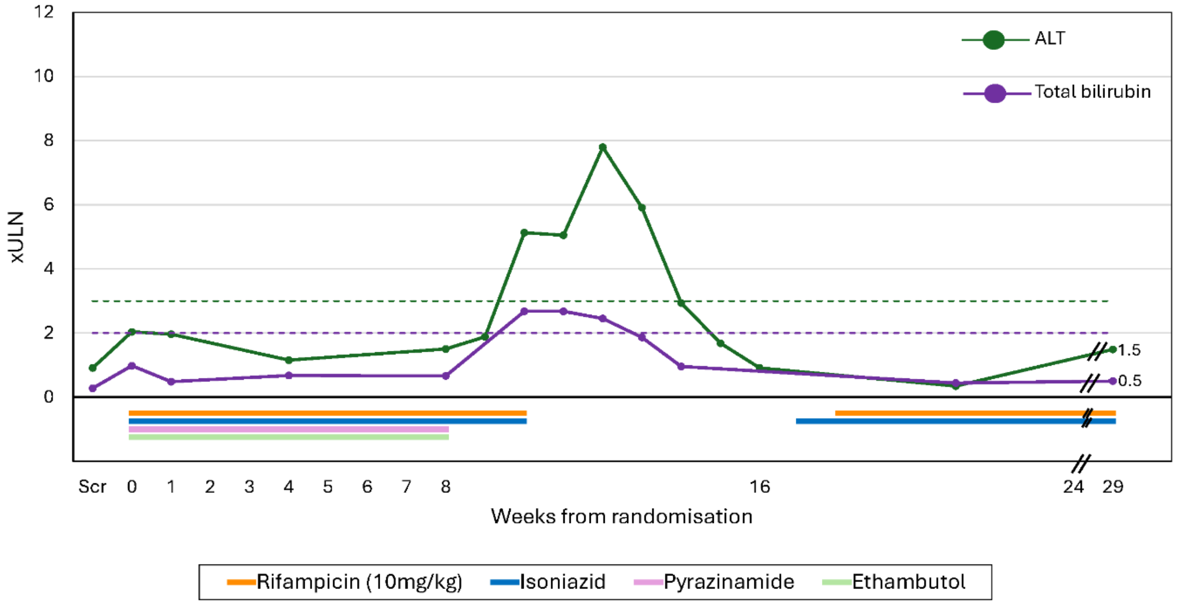

B) Participant in standard treatment group. Male, below 20 years of age, with no past medical history of note; alcohol consumption 2-12 units per week. Anorexia from baseline to week 10; no other liver-related symptoms. Elevated ALT and total bilirubin meeting Hy's Law criteria at week 12; alkaline phosphatase elevated < 2 times upper limit of normal. Hepatitis B surface antigen positive; core antibody IgG positive and IgM negative; A and C negative. Possible contributing causes: alcohol consumption and chronic hepatitis B infection. Hepatitis resolved with treatment interruption; treatment restarted after 36 days without recurrence.

Participants in rifampicin-linezolid group

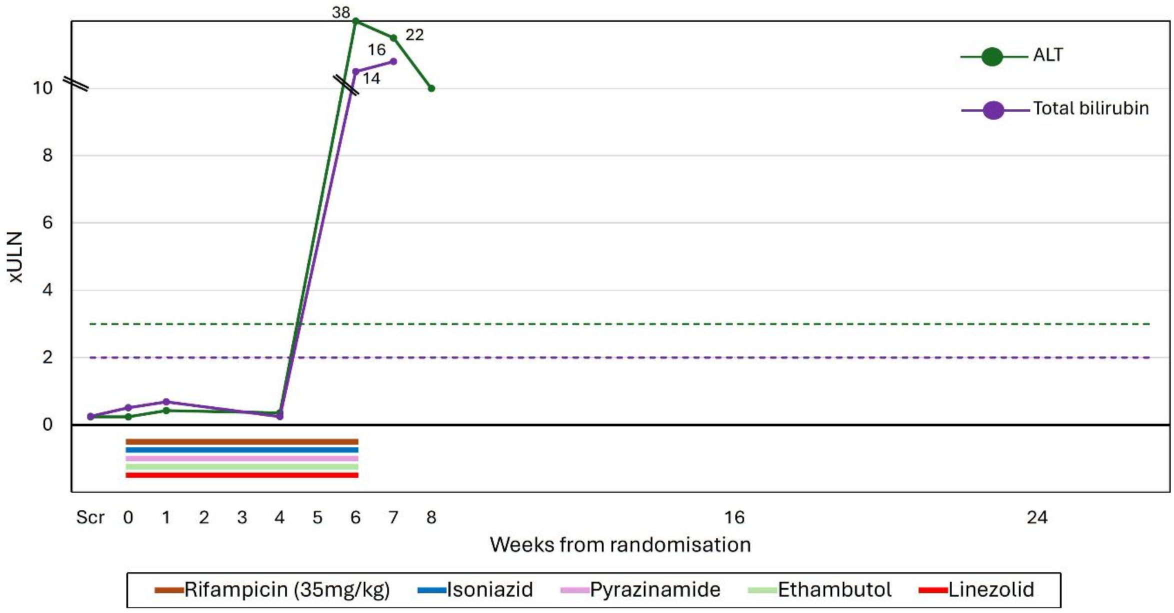

C) Participant in rifampicin-linezolid group. Female, 20-30 years of age; no past medical history of note. Abdominal pain, vomiting, nausea from week 1; jaundice at week 6. Elevated ALT and total bilirubin meeting Hy's Law criteria at week 6; alkaline phosphatase not elevated. Hepatitis A immune; B (surface antigen), C (antibody), D, E all negative. Possible contributing causes: lansoprazole started week 5, 6 days prior to LFT elevation. Progressive liver failure resulting in death.

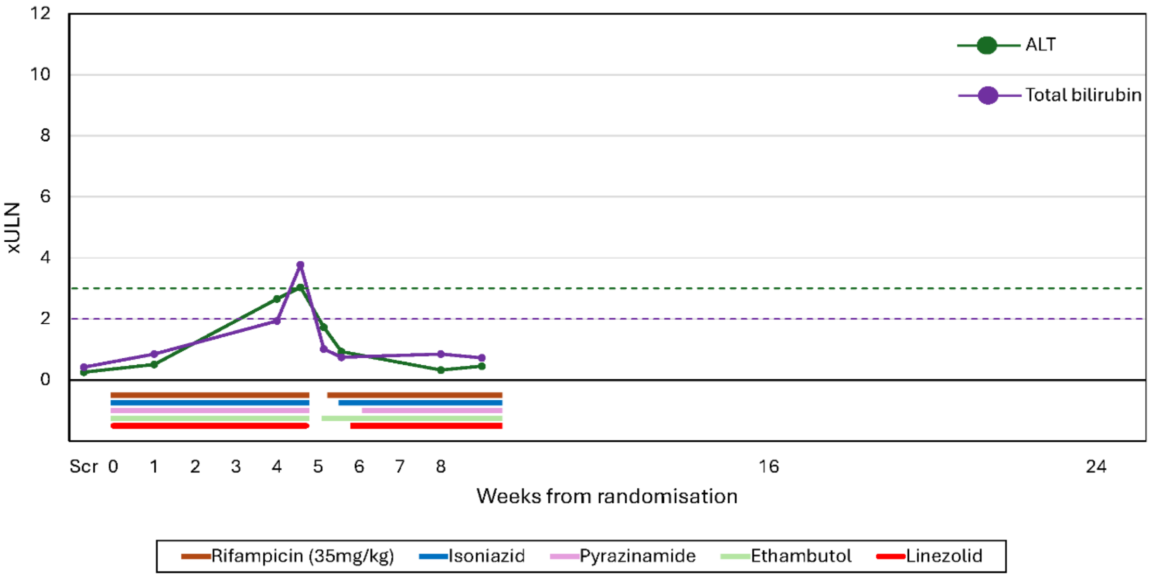

D) Participant in rifampicin-linezolid group. Male, 50-60 years of age, with Type 2 diabetes managed with insulin. Nausea and vomiting from week 4; followed by jaundice 4 days later. Elevated ALT and total bilirubin meeting Hy's Law criteria at week 5; alkaline phosphatase not elevated. Hepatitis A, B (surface antigen), C (antibody) and E negative. Possible contributing causes: none known. Hepatitis resolved with treatment interruption; assigned treatment restarted without recurrence.

Participants in rifampicin-clofazimine group

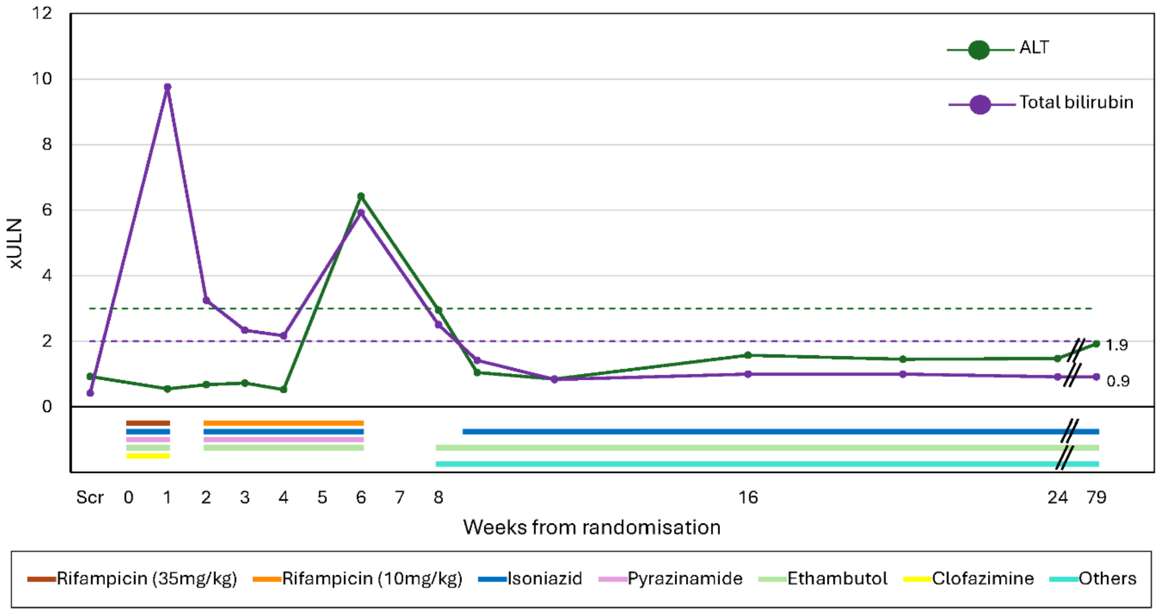

E) Participant in rifampicin-clofazimine group. Male, above 60 years of age; no past medical history of note. Vomiting and jaundice from week 1 to 2. Initial episode of isolated hyperbilirubinaemia; resolved with 9-day treatment interruption. Switched to standard treatment (participant request). Elevated ALT and total bilirubin meeting Hy's Law criteria at week 6; alkaline phosphatase not elevated. HCV antibody positive and HCV PCR 900,000 copies/ml; A, B, E serology negative. Possible contributing causes: HCV infection. Hepatitis resolved with treatment interruption; treatment restarted with isoniazid, ethambutol, levofloxacin without recurrence.

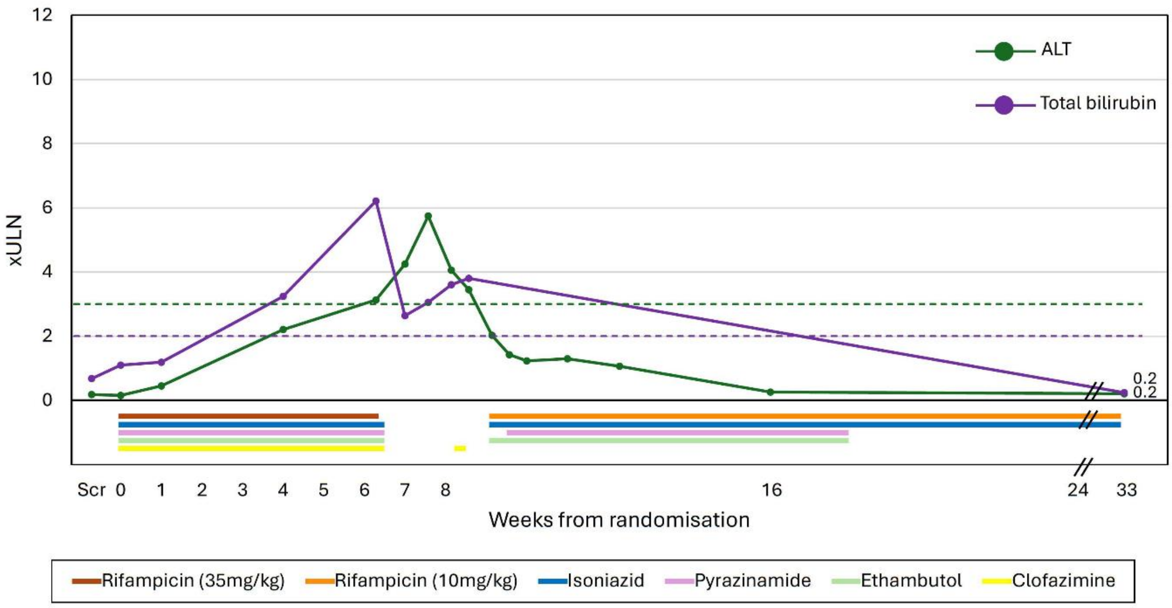

F) Participant in rifampicin-clofazimine group. Male, 20-30 years of age; no past medical history of note. Vomiting, intermittent, from baseline to week 3; jaundice from week 6. Elevated ALT and total bilirubin meeting Hy's Law criteria at week 6; alkaline phosphatase not elevated. Hepatitis A, B, C serology negative. Possible contributing causes: none known. Hepatitis resolved with treatment interruption; rash after restarting clofazimine; switched to standard treatment without recurrence of hepatitis.

Figure S7 PREVALENCE OF NAUSEA AND VOMITING ON TREATMENT

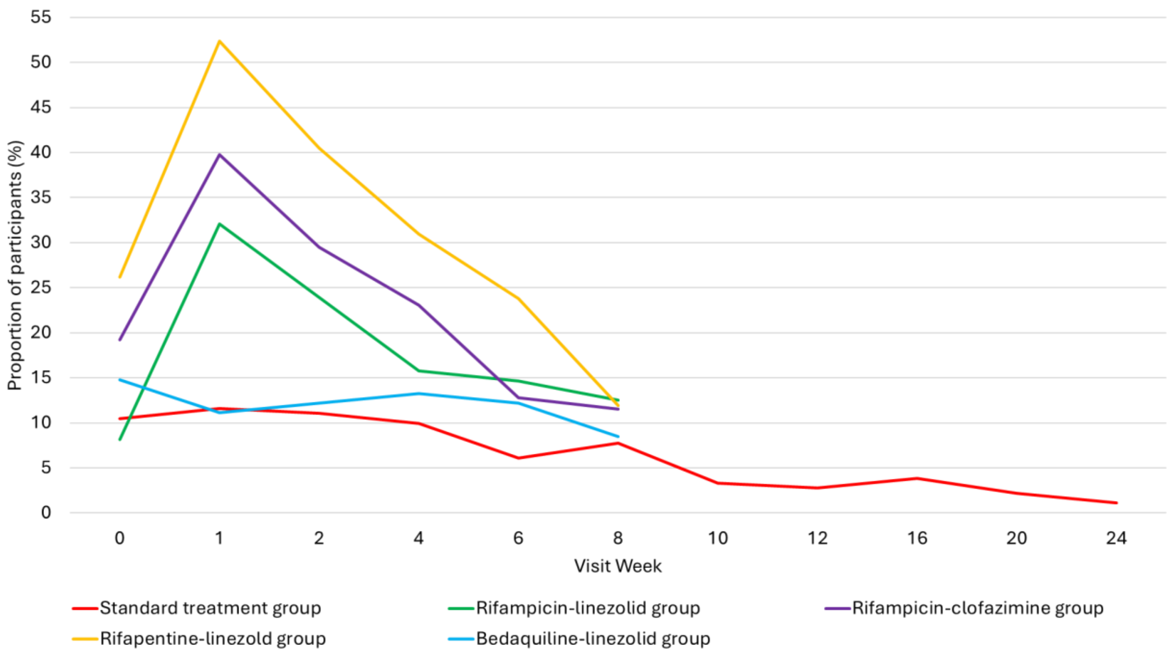

Proportion of participants in each group that reported presence of either nausea or vomiting or both with the previous 7 days, as recorded on the symptom checklist at each scheduled visit

Figure S8 PREVALENCE OF NAUSEA AND VOMITING ON TREATMENT BY ASSIGNED RIFAMPICIN DOSE IN THE RIFAMPICIN-LINEZOLID GROUP

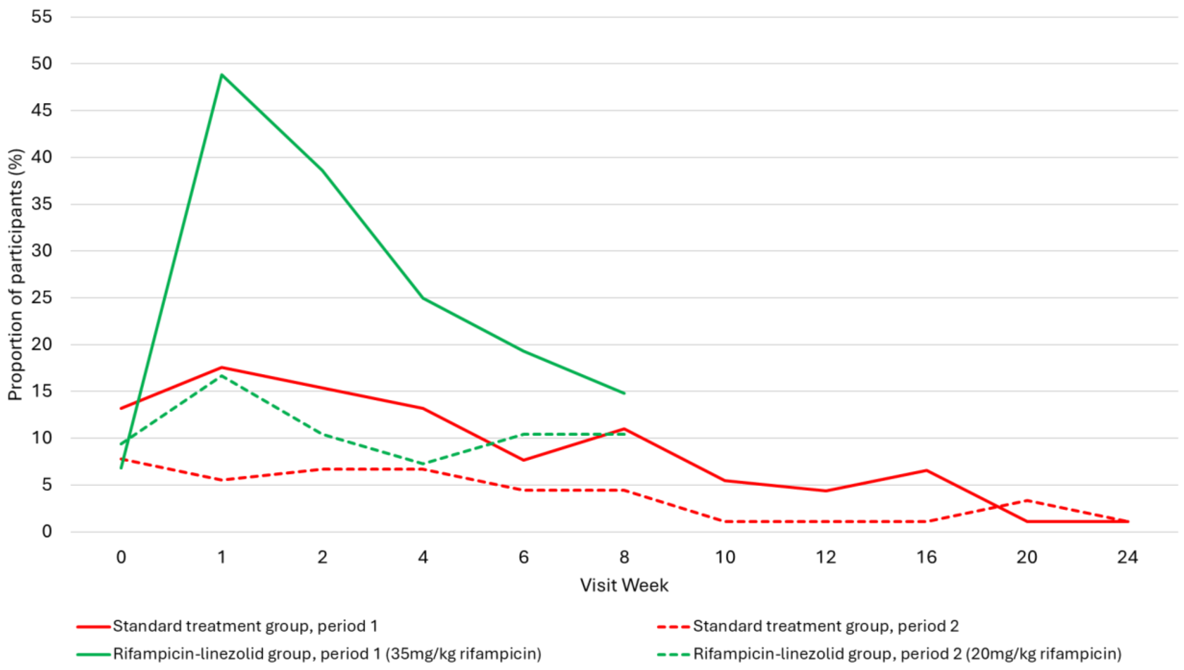

Proportion of participants that reported presence of either nausea or vomiting or both within the previous 7 days, as recorded on the symptom checklist at each scheduled visit; shown for participants 1-88 (period 1, assigned 35mg/kg rifampicin) and 89-184 (period 2, assigned 20mg/kg rifampicin) enrolled in the rifampicin-linezolid group and for participants who underwent contemporary randomisation to the standard treatment group during these periods.

TRUNCATE-TB

Two-month Regimens Using Novel Combinations to Augment Treatment Effectiveness for drug-sensitive Tuberculosis

TRIAL PROTOCOL
CONFIDENTIAL

**Version:**	**2.0**
**Date:**	**18 October 2019**
**SHORT TITLE**	**TRUNCATE-TB**
**NCT #:**	**03474198**

Authorised by:
Name: Nicholas Paton
Role: Chief Investigator
Signature:
Date:

Name: Angela Crook
Role: Trial Statistician (UK)
Signature:
Date:

GENERAL INFORMATION

This document describes the TRUNCATE-TB trial, coordinated by National University Hospital (Singapore), and provides information about procedures for entering patients into it. The protocol should not be used as an aide-memoire or guide for the treatment of other patients. Every care has been taken in drafting this protocol, but corrections or amendments may be necessary. These will be circulated to the registered investigators in the trial, but sites entering patients for the first time are advised to contact National University Hospital (Singapore) to confirm they have the most up-to-date version.

COMPLIANCE

The trial will be conducted in compliance with the approved protocol, the principles of Good Clinical Practice (GCP) and the UK Data Protection Act (DPA number: Z5886415). All participating sites will comply with the principles of GCP as laid down by the ICH topic E6 (Note for Guidance on GCP) and applicable national regulations.

SPONSOR

UCL is the trial legal sponsor and has delegated responsibility for the management of the trial to the National University Hospital (Singapore).

FUNDING

Direct funding for the trial: MRC/Wellcome Trust/DfID (UK); NMRC (Singapore)

MAIN CONTACTS

CHIEF INVESTIGATOR

Professor Nicholas Paton, MD FRCP
Department of Medicine,
Yong Loo Lin School Of Medicine,
NUHS Tower Block Level 10,
1E Kent Ridge Road,
Singapore 119228
Tel: +65 6772 6988
Email: nick_paton@nus.edu.sg

TRIAL STATISTICIAN (UK)

Angela Crook, PhD
MRC Clinical Trials Unit at UCL
90 High Holborn, 2nd floor
London, WC1V 6LJ
Tel: +44 207 670 4751
E-mail: angela.crook@ucl.ac.uk

TRIAL STATISTICIAN (SINGAPORE)

Qingshu Lu, PhD
Singapore Clinical Research Institute
31 Biopolis Way, Nanos #02-01
Singapore 138669
Tel: +65 6508 6753
E-Mail: Qingshu.lu@scri.edu.sg

PROJECT LEADER

Christopher Cousins, MBChB MRCP
Department of Medicine,
Yong Loo Lin School of Medicine,
National University of Singapore,
14 Medical Drive,
Singapore 117599
Tel: +65 6601 5371
Email: mdccdc@nus.edu.sg

INVESTIGATORS

A list of sites and investigators is provided in Appendix 7.

1 SUMMARY OF TRIAL

1.1 BACKGROUND AND AIMS

The standard management strategy for drug-sensitive (DS) pulmonary tuberculosis (TB) is to treat with multiple drugs for 6 months, although the majority of patients can be cured with much shorter treatment. Patients often fail to adhere to the long treatment, leading to poor clinical outcomes including drug resistance, which is expensive and difficult to treat.

This trial evaluates an alternative strategy of treating patients with DS-TB for 2 months with combinations including new drugs or optimised doses of currently available drugs, chosen for their sterilising efficacy. After treatment completion, patients are monitored closely and those who relapse are re-treated with a 6 month standard regimen.

The primary aim is to test the hypothesis that the TRUNCATE-TB management strategy is non-inferior to the standard TB management strategy assessed by the proportion of patients with unsatisfactory outcome at 2 years (96 weeks) after randomisation. The secondary aim is to assess the possible advantages of the TRUNCATE-TB management strategy compared to the standard management strategy from the patient perspective, the programme perspective and to assess cost-effectiveness. Other aims are to evaluate the pharmacokinetics, microbiological efficacy and toxicity of a number of boosted 8-week regimens in comparison to the standard treatment regimen; and to explore the relationship between various biomarkers and sterilisation/cure.

If this trial shows TRUNCATE-TB management strategy to be non-inferior and of potential benefit to patients and programmes this can be further tested in a large scale trial done at a programme level based on the optimal regimen, patient group and monitoring strategy identified by this trial.

1.1.1 TRIAL DESIGN

A randomised, open-label, multi-arm, multi-stage (MAMS), parallel group strategy trial.

1.1.2 PATIENTS

Adult patients with newly diagnosed DS-TB (confirmed by GeneXpert) enrolled within 10 days of starting TB therapy [for detailed criteria see Chapter 4].

Up to 900 patients (180 patients in each arm) will be studied.

1.1.3 STRATEGIES TESTED

The interventions to be studied are shown in Figure 1, page 6. Patients will be randomised to receive:

1) **The standard TB management strategy** using an initial 6 month (24 week) standard treatment regimen and treatment of relapses with a 6 to 8 month re-treatment regimen.

The standard treatment regimen comprises 4 drugs:
Arm A: 8 weeks rifampicin (10mg/kg), isoniazid, pyrazinamide, ethambutol, then 16 weeks rifampicin, isoniazid only

OR

2) **The TRUNCATE-TB management strategy** using an initial 2 month (8 week) boosted treatment regimen, close monitoring after treatment, and treatment of relapses with a 6-month standard treatment regimen.

The boosted treatment regimens comprise 5 drugs, including modifications to the standard regimen intended to increase sterilising activity:
Arm B: 8 weeks rifampicin (35mg/kg), isoniazid, pyrazinamide, ethambutol, linezolid
Arm C: 8 weeks rifampicin (35mg/kg), isoniazid, pyrazinamide, ethambutol, clofazimine
Arm D: 8 weeks rifapentine, isoniazid, pyrazinamide, linezolid, levofloxacin
Arm E: 8 weeks isoniazid, pyrazinamide, ethambutol, linezolid, bedaquiline

The duration of the boosted regimen will be extended to 12 weeks if patients are symptomatic and have a positive sputum smear at week 8 (linezolid will only be given for 8 weeks). Treatment will be supervised by directly observed therapy (DOT).

Randomisation will be in equal proportion to each of the arms (A to E).

1.1.4 Trial follow up and outcomes

Trial follow-up and outcome assessments are shown in Figure 2, page 7 and Table 1, page 9. Patient participation will last for 96 weeks from randomisation (extended in individual cases to allow 24 weeks follow-up after the completion of any period of re-treatment).

For comparison of TRUNCATE-TB management strategy with the standard TB management strategy, the primary outcome measure is unsatisfactory clinical outcome at week 96 after randomisation, as defined by:

- Ongoing requirement for TB treatment at week 96
  OR
- Ongoing TB disease activity at week 96 (clinical, microbiological and/or imaging evidence)
  OR
- Death before week 96

The main secondary outcome measures include:

From the patient perspective:
- Acceptability
- Total time on treatment
- Quality of Life
- Clinical adverse events
- Serious adverse events

From the programme perspective:
- Treatment adherence
- Treatment default
- Drug resistance
- Community transmission
- Health economics

For comparison of boosted 8-week treatment regimens with standard treatment regimen, other outcomes include:

- Pharmacokinetics
- Time to stable culture conversion
- Treatment failure
- Relapse
- Grade 3 or 4 adverse events on treatment
- QTc prolongation

1.1.5 TRIAL MONITORING

The trial is arranged in a number of stages (see Figure 3, page 8):

Stage 1: Pilot safety
Stage 2. Early efficacy
Stage 3. Qualifying efficacy
Stage 4: Definitive efficacy and safety

An independent data monitoring committee (IDMC) will review ongoing safety and efficacy data and at the end of each stage will make recommendations on which of the multiple arms should progress to the next stage.

1.1.6 TRIAL LOGISTICS AND OPERATIONAL ISSUES

The trial will be conducted at approximately 15-20 clinical sites in 4-6 countries.

The trial duration will be approximately 5 years, with 2 years for recruitment and a further 3 years for the completion of follow-up assessments.

1.1.7 TRIAL ORGANISATION

Sponsor: UCL
Main funding organisations: Medical Research Council, Wellcome Trust, Department for International Development (UK) and National Medical Research Council (Singapore)

1.1.8 TRIAL STATUS AT DATE OF CURRENT PROTOCOL VERSION

Arms open for new enrolment: A, B and E
The list of arms open for new enrolment is correct at the date of the current protocol version. This will be updated in the protocol at the time of any subsequent amendments to the main body of the protocol. This information may change in the intervening period between protocol versions as enrolment to arms is discontinued, following the MAMS trial design. An up-to-date list of arms open for enrolment is available from the trial coordinating centre at any time.

TRIAL PROCESSES

Figure 1: Patient Management Strategy

TRUNCATE-TB MANAGEMENT STRATEGY

INITIAL TREATMENT
8w boosted regimen

↓

Extended to:

- **≤12w** boosted regimen for missed doses, treatment interruption/treatment default
- **≤12w** boosted regimen if symptomatic and smear positive at week 8 (i.e. persistent clinical disease)
- **24w (≤32w)** boosted regimen + standard regimen (boosted ≤12w), if: toxicity; treatment interruption >14d; persistent symptoms and smear positive at week 12; pregnancy; other reasons

STANDARD MANAGEMENT STRATEGY

INITIAL TREATMENT
24w standard regimen

↓

Extended to:

≤32w standard regimen if long interruption (>14d during intensive phase)

↓

STOP TREATMENT AND MONITOR
Symptom checklist (every 4w) **+ Smear** (every 4-12w)
Cultures every 4-12w (research data collection)
GeneXpert if symptoms increase
CXR if symptoms increase but smear negative; or no symptoms but smear becomes positive (when previously negative)

↓

RELAPSE DIAGNOSED IF ≥ 2 of A, B or C are satisfied; OR if D is satisfied

A. Clinical Disease Progression
New, recurrent or increased severity of TB symptoms or signs compared with end of treatment without alternative more likely explanation

B. CXR Progression
Abnormalities that are compatible with active TB disease (cavitation, infiltrates, consolidation) with clear evidence of progression compared with end of treatment without alternative more likely explanation

C. Microbiological persistence / progression
Sputum sample taken at or after end of treatment is smear positive OR GeneXpert positive (if >24w after end of treatment; or if end of treatment was negative) OR culture positive

D. Confirmed positive sputum culture
Sputum culture positive on two consecutive samples with at least one taken ≥ 4w after end of treatment

↓

Collect **additional sputum samples** to ensure ≥ 2 consecutive cultures with results positive / awaited
Request **DST** on last 2 positive cultures taken at or prior to diagnosis of relapse
GeneXpert if not done as part of relapse evaluation (for rifampicin susceptibility)

↓

RE-TREATMENT (TRUNCATE-TB management strategy)
Empirical 24w standard regimen
Or
Empirical 24-32w re-treatment regimen based on national treatment guidelines if 24w initial treatment

Regimen modified according to DST results and drug tolerability
May stop if cultures do not confirm relapse and alternative diagnosis emerges (clinician discretion)

RE-TREATMENT (standard management strategy)
Empirical 24-32w re-treatment regimen based on national treatment guidelines

Regimen modified according to DST results and drug tolerability
May stop if cultures do not confirm relapse and alternative diagnosis emerges (clinician discretion)

Figure 2: Trial treatment Arms and Follow-up

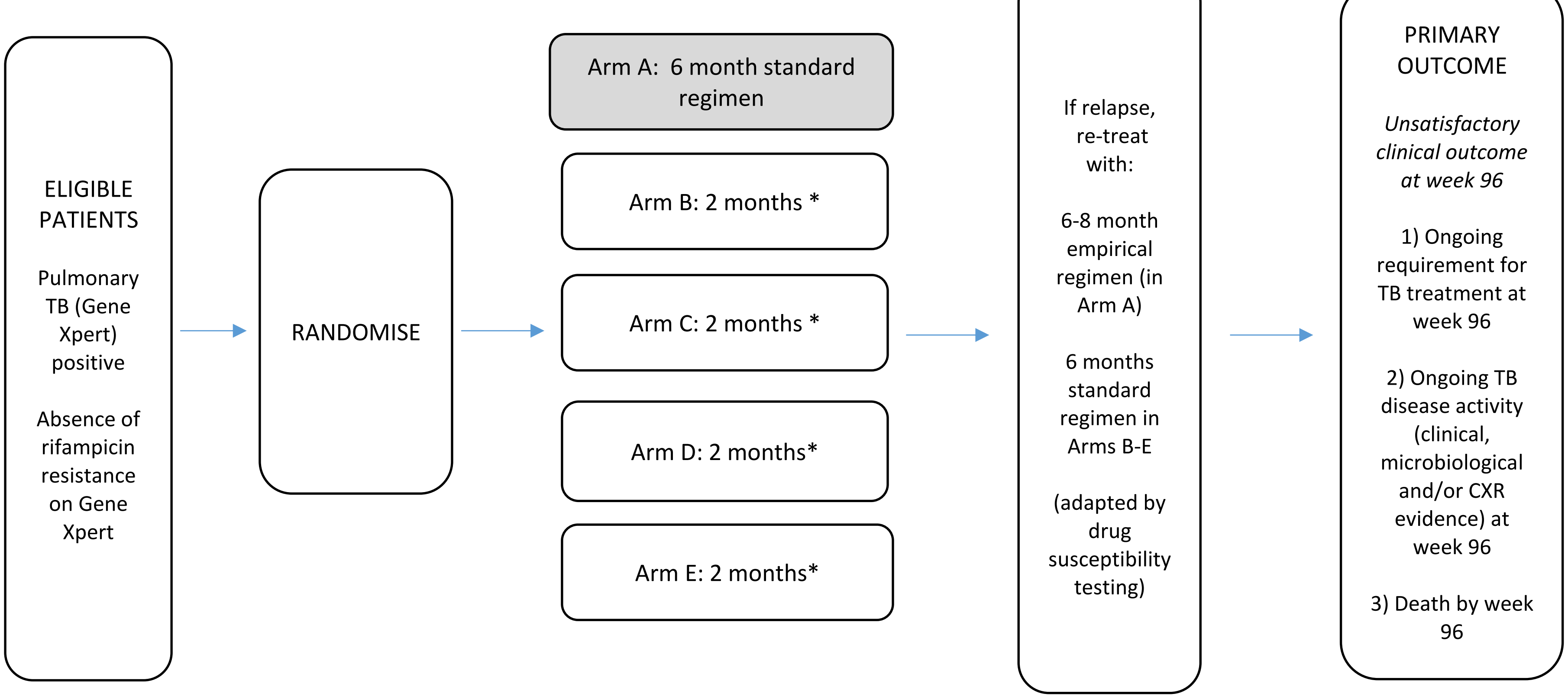

*Extend up to 3 months for missed doses or if symptomatic and positive smear at 2 months

Figure 3: Trial stages and safety/efficacy monitoring

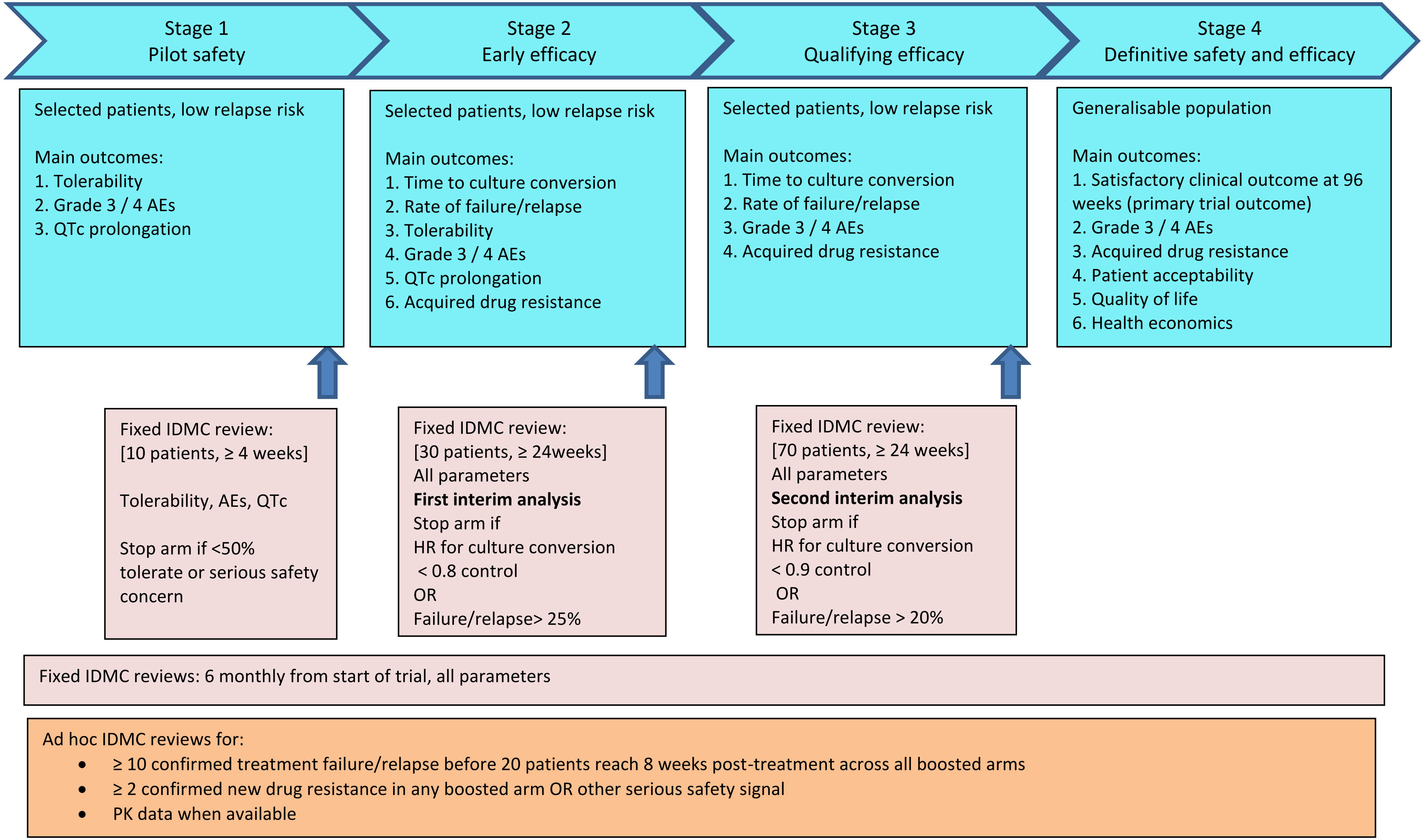

Table 1: Trial visit and assessment schedule

VISIT TIMING[1]	SCREENING	D0	W1	W2	W4	W6	W8	W10	W12	W16	W20	W24	W36	W48	W60	W72	W84	W96
Informed Consent	X																	
Eligibility criteria	X	X																
Randomisation		X																
CLINICAL EVALUATION																		
Medical history & demographics	X	X																
Symptoms	X	X	X	X	X	X	X	X	X	X	X	X	X	X	X	X	X	X
Physical examination	X	X	X	X	X	X	X	X	X	X	X	X	X	X	X	X	X	X
Medication review and adherence	X	X	X	X	X	X	X	X	X	X	X	X	X	X	X	X	X	X
HEALTHCARE UTILISATION & QOL																		
Healthcare utilisation		X	X	X	X	X	X	X	X	X	X	X	X	X	X	X	X	X
EQ-5D		X	X	X	X	X	X	X	X	X	X	X	X	X	X	X	X	X
MOS-HIV		X																X
Patient acceptability questionnaire														X				X
Socioeconomic evaluation		X																X
INVESTIGATIONS																		
ECG[2]	X	X	X		X		X											
CXR[3]	X	X					X											X
Spirometry							X							X				X
URINE																		
Pregnancy test	X				X		X											
Urine for storage[4]		X			X		X					X						
SPUTUM																		
Smear[5]	X	X	X	X	X	X	X	X	X	X	X	X	X	X	X	X	X	X
Liquid culture[6]		X	X	X	X	X	X	X	X	X	X	X	X	X	X	X	X	X
GeneXpert test[7]	X						X											
Drug susceptibility tests[8]		X					X											
BLOOD																		
Standard safety monitoring[9]	X	X	X	X[10]	X	X[10]	X	X[10]										
HIV test[11] (and CD4 count)[12]	X																	
Drug levels (PK)[13]		X			X		X					X						
Plasma and RNA storage[14]		X			X		X					X						

1. Additional telephone visits at weeks 30, 40, 44, 52, 56, 64, 68, 76, 80, 88, 92 (omitted if attended scheduled visit within previous 7 days) to evaluate symptoms, medication and adherence. A telephone visit will be performed at the time of trial closure. Visit windows: ±3 days for W1 to W4; ± 5 days for W6 and W8; ± 7 days for W10 to W24; ± 14 days for W30 onwards and ± 28 days for W96.
2. Screening ECG may be deferred to Day 0, but result must be available prior to performing randomisation. Additional ECG required at end of treatment for patients who stop boosted regimen between week 8 and 12; additional ECG required at week 12 for patients who switch boosted regimen to standard treatment between week 8 and 12. If QTc >450 ms, ECG should be repeated to confirm.
3. CXR at any visit need not be repeated if done in previous 7 days and film is available for evaluation. Additional CXR done at end of treatment and at the first visit of any suspected episode of relapse.
4. Urine (10ml) for storage. Additional sample taken for storage and for pregnancy test at end of treatment and at first suspicion of relapse (one for each episode)
5. 2 sputum smears on separate days at week 96.
6. Spot sputum sample at each visit (if available), cultured on liquid media (MGIT); 2 sputum samples on separate days at week 96.
7. GeneXpert test at screening need not be repeated if a positive result is available from a test performed earlier during this illness episode, done in study-approved laboratory and results are/will be available to research team. GeneXpert at week 8 (and end of treatment, if treatment continues after 8 weeks), and on the initial sputum sample obtained during any suspected episode of relapse.
8. Drug susceptibility at baseline, and on positive cultures at or after week 8 (monthly during any retreatment episode).
9. Blood for standard safety monitoring: FBC, electrolytes (sodium, potassium, creatinine), LFTs (ALT, alkaline phosphatase, bilirubin) done at scheduled visits and at end-of-treatment visit. Additional bloods at screening only: amylase/lipase, magnesium, calcium and glucose. Blood tests at any visit need not be repeated if results available from previous 2 days.
10. Additional FBC at weeks 2, 6, and 10 for patients receiving linezolid (Arms B, D, E only).
11. HIV antibody test need not be repeated if done in previous 30 days and results available to trial team.
12. CD4 count only if patient HIV positive and trial is open to enrolment of HIV-positive participants at that site.
13. Blood for drug levels to be taken at Day 0 (1 hour post-dose), week 4 (pre-dose and 2 hours post dose) and week 8 (pre-dose and 4 hours post-dose), and week 24 (patients randomised to bedaquiline or clofazimine arms only, single sample, no timing requirements). Samples may be omitted for patient convenience or logistical reasons.
14. Blood for plasma (10ml EDTA tube) and host RNA (5ml RNA preservation tube) storage. Additional samples taken at end of treatment and at first suspicion of relapse (one for each episode).

CONTENTS

1 SUMMARY OF TRIAL 3
1.1 BACKGROUND AND AIMS 3
1.1.1 Trial design 3
1.1.2 Patients 3
1.1.3 Strategies tested 3
1.1.4 Trial follow up and outcomes 4
1.1.5 Trial monitoring 5
1.1.6 Trial logistics and operational issues 5
1.1.7 Trial organisation 5
1.1.8 Trial status at date of current protocol version 5
TRIAL PROCESSES 6
Figure 1: Patient Management Strategy 6
Figure 2: Trial treatment Arms and Follow-up 7
Figure 3: Trial stages and safety/efficacy monitoring 8
Table 1: Trial visit and assessment schedule 9
CONTENTS 11
ABBREVIATIONS 15
2 BACKGROUND 18
2.1 TB GLOBAL DISEASE BURDEN AND THE STANDARD TB MANAGEMENT STRATEGY 18
2.2 A NEW APPROACH: THE TRUNCATE-TB MANAGEMENT STRATEGY 19
2.3 ADVANTAGES AND DISADVANTAGES OF THE TRUNCATE-TB MANAGEMENT STRATEGY 19
2.4 THE OPTIMAL LENGTH OF INITIAL TREATMENT FOR THE TRUNCATE-TB MANAGEMENT STRATEGY 21
2.5 BOOSTED TREATMENT REGIMENS IN THE TRUNCATE-TB MANAGEMENT STRATEGY 23
2.6 TRIAL DESIGN: THE MULTI-ARM MULTI-STAGE APPROACH 26
2.7 AIMS OF THE TRIAL 28
3 SELECTION OF SITES AND PRINCIPAL INVESTIGATORS 29
3.1 SITE/INVESTIGATOR INCLUSION CRITERIA 29
4 SELECTION OF PATIENTS 31
4.1 PATIENT INCLUSION CRITERIA 31
4.2 PATIENT EXCLUSION CRITERIA 31
4.3 SCREENING PROCEDURES AND PRE-RANDOMISATION INVESTIGATIONS 33
5 RANDOMISATION AND ENROLMENT 35
5.1 RANDOMISATION VISIT (BASELINE, DAY 0) 35
5.2 PROCEDURE FOR RANDOMISATION 35

5.3 POST-RANDOMISATION PROCEDURES AND FOLLOW-UP ... 36
5.4 CO-ENROLMENT GUIDELINES ... 36
6 TREATMENT OF PATIENTS ... 37
6.1 INTRODUCTION ... 37
6.2 STANDARD TB TREATMENT ARM ... 38
6.3 BOOSTED REGIMENS FOR USE IN THE TRUNCATE-TB MANAGEMENT STRATEGY ... 39
6.4 TREATMENT SUPPLY AND SUPERVISION ... 42
6.5 GENERAL MANAGEMENT AND CONCOMITANT MEDICATIONS ... 42
6.6 MANAGEMENT OF HIV ... 43
6.7 MANAGEMENT OF PREGNANCY ... 43
6.8 MODIFICATION OF INITIAL TREATMENT REGIMEN IN THE TRUNCATE-TB MANAGEMENT STRATEGY ... 44
6.9 MODIFICATION OF INITIAL TREATMENT IN THE STANDARD MANAGEMENT STRATEGY ... 46
6.10 MONITORING AND DIAGNOSIS OF RELAPSE ... 47
6.11 RE-TREATMENT REGIMENS ... 49
7 ASSESSMENTS & FOLLOW-UP ... 51
7.1 VISIT TIMING ... 51
7.2 VISIT ASSESSMENTS ... 52
7.3 ASSESSMENT PROCEDURES ... 56
7.4 MAINTAINING ADHERENCE TO FOLLOW-UP ... 62
8 SAFETY REPORTING ... 64
8.1 DEFINITIONS ... 64
8.2 INVESTIGATOR RESPONSIBILITIES ... 66
8.3 SPONSOR RESPONSIBILITIES ... 68
9 QUALITY ASSURANCE & CONTROL ... 69
9.1 RISK ASSESSMENT ... 69
9.2 CENTRAL MONITORING AT THE TRIAL COORDINATING CENTRE ... 69
9.3 MONITORING AT THE CLINICAL SITES ... 69
9.4 CONFIDENTIALITY ... 69
10 STATISTICAL CONSIDERATIONS ... 70
10.1 METHOD OF RANDOMISATION ... 70
10.2 OUTCOME MEASURES ... 70
10.3 SAMPLE SIZE ... 75
10.4 INTERIM MONITORING & ANALYSES ... 77
10.5 OUTLINE ANALYSIS PLAN ... 80
11 PHARMACOKINETIC SUB-STUDY ... 84
11.1 BACKGROUND ... 84
11.2 SELECTION OF PATIENTS ... 84

11.3 SELECTION OF SITES 85
11.4 NUMBER OF PARTICIPANTS 85
11.5 ANALYTICAL METHODS 86
11.6 PK DATA ANALYSIS 87
11.7 SUB-STUDY DATA MONITORING 87
12 ETHICAL CONSIDERATIONS AND APPROVALS 88
12.1 OVERALL ETHICAL DESIGN PRINCIPLES 88
12.2 RESPECT FOR PERSONS 88
12.3 JUSTICE 88
12.4 BENEFICENCE 89
12.5 RISKS OF THE TRIAL FOR THE PATIENT IN THE TRUNCATE-TB MANAGEMENT STRATEGY ARMS 89
12.6 RISKS FOR PATIENTS IN THE STANDARD TB MANAGEMENT ARM 96
12.7 BENEFITS TO SOCIETY FROM CONDUCTING THIS TRIAL 97
12.8 WEIGHING THE RISKS AGAINST THE BENEFITS TO THE PATIENT AND SOCIETY 97
12.9 ETHICAL APPROVALS 98
13 REGULATORY APPROVALS AND COMPLIANCE 99
13.1 REGULATORY AUTHORITY APPROVALS 99
13.2 OTHER APPROVALS 99
13.3 REGULATORY COMPLIANCE 99
16.1 TRIAL MANAGEMENT GROUP (TMG) 102
16.2 TRIAL STEERING COMMITTEE (TSC) 102
16.3 INDEPENDENT DATA MONITORING COMMITTEE (IDMC) 102
17 PUBLICATION 103
REFERENCES 104
APPENDIX 1: QUANTIFICATION OF SIZE OF PLEURAL EFFUSIONS 110
APPENDIX 2: IUATLD SMEAR MICROSCOPY GRADING SCALE 111
APPENDIX 3: MRC BREATHLESSNESS SCALE 112
APPENDIX 4: MEASUREMENT OF QT INTERVAL 113
APPENDIX 5: CREATININE CLEARANCE CALCULATION 115
APPENDIX 6: PATIENT ACCEPTABILITY QUESTIONNAIRE 116
APPENDIX 7: LIST OF SITES AND INVESTIGATORS 117
APPENDIX 8: TEMPLATE FOR CONSENT FORM 120
APPENDIX 9: SAFETY PROFILE OF TRIAL DRUGS IN PREGNANCY 138
APPENDIX 10: DIVISION OF AIDS TABLE 140
APPENDIX 11: MEDICAL OUTCOMES STUDY-HIV HEALTH SURVEY 149
APPENDIX 12: EQ-5D 153

APPENDIX 13: PHARMACOKINETICS OF TRIAL DRUGS AND REGIMENS................................154
APPENDIX 14: TEMPLATE FOR PHARMACOKINETIC SUB-STUDY CONSENT FORM 157
APPENDIX 15: SUMMARY OF AMENDMENTS IN VERSION 1.2 162
APPENDIX 16: SUMMARY OF AMENDMENTS IN VERSION 2.0 163

ABBREVIATIONS

Abbreviation	Expansion
AE	Adverse event
AIDS	Acquired immune deficiency syndrome
ALT	Alanine transferase
AR	Adverse reaction
ART	Antiretroviral therapy
AUC	Area under the curve
CD4	Cluster of differentiation-4 T-cell lymphocyte
CRF	Case report form
CXR	Chest X-ray
D	Day
DAIDS	Division of AIDS
DfID	Department for International Development (UK)
DNA	Deoxyribonucleic acid
DOT	Directly observed therapy
DST	Drug susceptibility testing
DS-TB	Drug-sensitive tuberculosis
EBA	Early bactericidal activity
ECG	Electrocardiogram
EDTA	Ethylenediaminetetraacetic acid
EQ-5D	EuroQOL five dimensions questionnaire
ERC	Endpoint Review Committee
FBC	Full blood count
FDA	Food and Drug Administration
FDC	Fixed dose combination
GCP	Good Clinical Practice

Abbreviation	Expansion
HIV	Human immunodeficiency virus
ICH	International Conference on Harmonisation of Technical Requirements for Registration of Pharmaceuticals for Human Use
IDMC	Independent Data Monitoring Committee
IRB	Institutional Review Board
IMP	Investigational medicinal product
ISRCTN	International Standard Randomised Controlled Trial Number
ITT	Intention-to-treat
LFT	Liver function test
MAMS	Multi-arm, multi-stage
MDR-TB	Multi-drug resistant tuberculosis
MGIT	Mycobacterial growth indicator tube system
MIC	Minimum inhibitory concentration
mITT	Modified intention-to-treat
MOS-HIV	Medical outcomes study HIV questionnaire
MRC	Medical Research Council
MRC CTU at UCL	Medical Research Council Clinical Trials Unit at University College London
ms	Milliseconds
NMRC	National Medical Research Council (Singapore)
NUS	National University of Singapore
PK	Pharmacokinetics
PP	Per-protocol
QoL	Quality of life
QT	QT interval
QTc	QT interval (corrected)
RNA	Ribonucleic acid

Abbreviation	Expansion
SAE	Serious adverse event
SAP	Statistical analysis plan
SAR	Serious adverse reaction
SOP	Standard operating procedure
SPC	Summary of product characteristics
STR	Standard therapy re-introduction
SUSAR	Suspected Unexpected Serious Adverse Reaction
TB	Tuberculosis
TMG	Trial Management Group
TSC	Trial Steering Committee
UCL	University College London
W	Week
WGS	Whole genome sequencing
WHO	World Health Organization

2 BACKGROUND

2.1 TB GLOBAL DISEASE BURDEN AND THE STANDARD TB MANAGEMENT STRATEGY

TB remains a major global disease, with an estimated 9 million new TB cases and 1.5 million TB deaths per year.[1] Although multi-drug resistant TB (MDR-TB), defined as TB with resistance to rifampicin and isoniazid, is an increasing problem and is complicated and expensive to treat,[2] the large majority of new cases of TB are drug-sensitive (DS-TB). Improving the treatment of DS-TB is likely to bring important global health benefits both by directly improving outcomes of patients and by preventing the generation of new cases of MDR-TB.

The standard TB management strategy for drug-sensitive pulmonary disease comprises 6 months combination therapy, preferably with the intake of each dose of medication observed by a healthcare worker (called directly observed therapy, DOT).[3] Following completion of the course of treatment, patients are usually discharged without follow-up. The regimen used in the standard TB management strategy consists of 2 months of rifampicin, isoniazid, pyrazinamide and ethambutol (the intensive phase) followed by 4 months of rifampicin and isoniazid alone (the continuation phase). Although generally well tolerated, common side-effects include gastrointestinal intolerance (from rifampicin or pyrazinamide), hepatitis (from rifampicin, isoniazid, or pyrazinamide), skin reactions (from rifampicin), and arthralgia/gout (from pyrazinamide).

If healthcare systems were able to deliver the standard TB management strategy with appropriate supervision to all patients globally, then the incidence of DS-TB disease would likely decline rapidly and the drug selection pressure driving the generation of MDR-TB would diminish, reducing its incidence too.[4] However, once clinical symptoms improve, as they usually do within a few weeks after starting treatment, patients often find it challenging to adhere to the long course of treatment, especially if there are ongoing treatment side-effects. As few programmes have sufficient resources to maintain supervision of treatment throughout the full course, poor adherence may go undetected, leading to treatment failure which in turn drives the emergence of drug resistance. Furthermore, even if a course of treatment is completed satisfactorily, the rates of post-treatment recurrence are substantial (ranging from 2% in low incidence settings to 8% in high incidence settings in the first year following the completion of treatment, and likely much higher in sub-optimal programme settings.[5,6] With no post-treatment follow-up, episodes of recurrence, whether of MDR-TB or DS-TB, are detected relatively late allowing unnecessary spread within the community.

Thus, although the standard TB management strategy is theoretically sound, in reality resource limitations combined with a challenging patient population create conditions that promote low cure rates, generation of MDR-TB and spread (of DS- and MDR-TB) within the community. With healthcare systems overstretched and experiencing large funding gaps, it is clear that simply continuing to emphasise the same treatment and delivery approach is unlikely to provide a solution to the problem in any reasonable time frame.[7] Although the development of new drugs and novel regimens targeted at MDR-TB is important, this is a downstream solution that will benefit only a minority of patients. Furthermore, the benefits of new drugs for MDR-TB is likely to be short-lived as resistance to new drugs develops rapidly when they are combined with relatively weak companion drugs.[8,9]

The solution clearly must be found in addressing the limitations of the current approach to the treatment of DS-TB in programmes. New approaches are needed that allow healthcare systems to use limited resources more effectively and economically to treat and cure more patients with DS-TB and that minimise the conditions that promote the generation and spread of MDR-TB.

2.2 A NEW APPROACH: THE TRUNCATE-TB MANAGEMENT STRATEGY

The current 6 month standard of care was adopted when clinical trials in the 1980s found that cure rates in trial settings were consistently above 95% with this duration of treatment.[10] However, it was clear at that time that the majority of patients can be cured with much shorter courses of treatment.[10] A review of the British MRC trials in patients with sputum smear positive disease found average relapse rates of 12% and 13% in patients with smear positive disease treated for just 4 months and 3 months respectively.[11] A more recent Cochrane review of trials with shorter duration of standard drugs showed relatively low absolute rates of relapse for regimens of 3-4 months' duration in smear-positive disease (usually below 10%, with the exception of 1 trial where it was 18%).[12] This has also been corroborated by the recent findings in large trials of 4-month fluoroquinolone containing regimens, that found overall rates of culture confirmed relapse of 9% - 15%.[13-15]

The relapse rate with short regimens may be even lower in patients with less severe disease or lower bacterial burden. A 4-month regimen with standard drugs in patients without cavitary disease and with culture conversion at 8 weeks gave a relapse rate of 7%.[16] An early trial of 3 month and 2 month regimens with standard drugs in smear-negative (culture positive) patients in Hong Kong found relapse rates of 7% and 14% respectively.[17]

Thus it is clear that with current standard drugs, the large majority of patients are cured after a few months and receive many months of additional treatment that is unnecessary. For patients, this unnecessary treatment causes inconvenience, increases the risk of drug toxicity and also sets a target for treatment that may feel unattainable and thereby contribute to poor motivation and default. For programmes, resources are squandered on distributing and supervising this unnecessary treatment that in turn dilutes the capacity to provide more intensive support at periods and to persons where it matters most.

An alternative strategy can therefore be proposed which may better match treatment to need:

> The TRUNCATE-TB management strategy:
> Instead of treating everyone with a long course of treatment (thereby giving unnecessary treatment to the majority for the sake of the minority who need it, but cannot be identified), an alternative approach is to treat everyone with a much shorter course of treatment (which is adequate for the majority) and to follow closely and retreat the minority of those who relapse (and are thereby identified as needing longer treatment) with a standard treatment regimen.

2.3 ADVANTAGES AND DISADVANTAGES OF THE TRUNCATE-TB MANAGEMENT STRATEGY

There are a number of potential advantages and disadvantages of this new strategic approach and these are best examined separately from the perspective of the patient and from the perspective of the programme:

Potential advantages for patients include:

- reduced time on initial treatment
- reduced duration of side-effects from medication
- reduced potential for adverse drug interactions (e.g. with HIV medication)
- reduced socioeconomic burden from lengthy supervised therapy
- increased treatment adherence (more modest goals may increase motivation)
- reduced risk of drug resistance (shorter drug treatment with increased adherence and focused supervision). This strategy should not increase the risk of drug resistance. In previous short course trials, relapse strains were nearly all drug sensitive,[11] and could be retreated with standard drugs. Drug resistance arises when treatment is taken erratically, and the strategy should help to protect against this

Potential disadvantages for patients include:

- the possible need for repeat treatment for 6 months (although the total duration of TB treatment would be only 2-3 months longer)
- the additional morbidity associated with a relapse episode – this is likely to be minimal because patients will be followed closely post-treatment in this strategy and hence relapses will be detected early; furthermore relapses are usually mild[18]
- the possibility of additional toxicity arising from use of a boosted treatment regimen (see below)

Potential advantages for programmes include:

- Ability to direct resources for adherence support to focus on critical periods when people can be cured
- Resource savings by reducing drugs and the manpower needed to supervise treatment (simply maintaining contact post-treatment is considerably easier than trying to supervise treatment)
- Reduced potential for drug resistance generation and spread

Potential disadvantages for programmes include:

- The need for longer-term supervision for a minority of patients who need retreatment
- The need to maintain closer contact with all patients post-treatment (but the resources required for the programme to remain in contact with the patient to ensure recurrent symptoms are reported and to evaluate the patient when needed are likely to be much less than the resources needed for the alternative of distributing medication, supervise treatment ingestion and monitor side effects)
- Increased episodes of transmission in the community and the additional resources needed for contact tracing (although relapses should be detected early with the post-treatment follow-up, in contrast to the potential for transmission from relapses occurring after the normal standard treatment which are likely to be detected later in the absence of post-treatment follow up. Furthermore, close household contacts may be taking isoniazid prophylaxis during the period when patients have completed the initial course of treatment and so will be protected against early relapse).

The TRUNCATE-TB management strategy will present patients with more modest and achievable goals that may optimise adherence to treatment. This strategy will allow programmes to target treatment-support to the critical periods when it is needed. Thus, in a programme setting, by focusing the effort of patients and correcting the mal-distribution of programme resources, this new strategy may achieve better overall cure rates than the standard TB management strategy as well as minimise the opportunity for unsupervised and erratic drug consumption that leads to the selection of drug resistance.

Weighing up the numerous advantages and disadvantages of the new strategy from a patient perspective and from a programme perspective is complicated. For example, from the programme perspective, some of the advantages of shorter initial treatment may be offset by the resources needed to follow and retreat people who relapse later.

It is uncertain what the proportion needing re-treatment would be, nor how low this proportion would need to be for this strategy to prove cost-effective and to be adopted by programmes. It is possible that a potent 2 month regimen with one or more new TB drugs can cure nearly all and follow up post-treatment could be of minimal intensity. It is also likely that the response to a 2-month treatment strategy seen in a clinical trial will be better sustained in a programme setting, in contrast to the attrition of cure rates seen when a 6 month regimen is translated from clinical trial to programme setting (see above).

In weighing these advantages and disadvantages, it is important to note two key points:

1. That the advantages will be experienced by all patients, whereas the disadvantages will affect only a minority.

2. That there are very limited data currently that can be used to judge this new strategy. These data, crucial for evaluating the relative merits of this approach, will be generated by this trial.

2.4 THE OPTIMAL LENGTH OF INITIAL TREATMENT FOR THE TRUNCATE-TB MANAGEMENT STRATEGY

For this strategy, we have chosen a **2 month initial treatment** using a boosted treatment regimen (extended to 3 months for inadequate response, as below). The reasons for this are:

(i) A 2-month regimen is likely to be sufficiently attractive to most patients to be perceived as outweighing the potential increased risk of relapse

(ii) A 2-month regimen is likely to be attractive to programmes both in terms of cost-effectiveness and in terms of optimising constrained resources (can treat more people and deliver a better quality of care to those people)

(iii) There is a reasonable likelihood, based on the existing data and the conditions listed below, that a 2-month regimen could yield relapse rates that were sufficiently low to not offset the various advantages to programmes and patients

(iv) A 2-month regimen will reduce the risk of toxicity (known and unknown) from the newer drugs where this is exposure-related

(v) A 2-month regimen will fit well with the recommended time for initiation of antiretroviral therapy (ART) in HIV-infected patients reducing the need for TB and HIV medication to be taken concurrently

The optimal length of treatment for use in this new strategy is closely related to the risk of relapse that will be tolerated – the shorter the initial regimen, the higher the relapse rate is likely to be. However, the dynamics of this inverse relationship between treatment duration and relapse rate are not well understood, even with varying the duration of standard treatment, and are not known at all for regimens that include non-standard sterilising drugs such as we propose to use in this trial. There may also be factors unrelated to the duration of treatment – in particular the human immune response (which is known to be able to clear TB even in the absence of drug treatment)[19] – that contribute a greater proportion to cure with shorter treatment durations.

The following steps are intended to minimise the treatment failure/ relapse rate in the TRUNCATE-TB management strategy:

1. The use of boosted regimens with predicted enhanced sterilising activity (see Section 2.5)
2. The extension of initial treatment from 8 to 12 weeks in patients who remain symptomatic with a positive smear result at week 8 (see Section 6.8.2)
3. The use of a dose-based (rather than fixed) duration of treatment – i.e. extension of the initial treatment beyond 8 weeks in patients with missed doses, until completion of the required total number of doses
4. The exclusion of patients with risk factors for higher rate of relapse such as uncontrolled diabetes, concomitant use of steroids or immunosupressive therapy, alcohol or drug abuse (risk of poor adherence) and initially, until completion of early interim efficacy analyses, patients with HIV infection, sputum smear 3+ or large cavities on chest X-ray (CXR) (see Section 4.2).

Based on the data from previous trials of 2-4 month regimens with standard drugs outlined in Section 2.2 above and the 4 measures intended to ameliorate the relapse rate listed above, we believe that the combined treatment failure/relapse rates for the selected boosted regimens will lie within the range of 5-15% and this should allow the TRUNCATE-TB management strategy to be viable in principle. However, the only reliable evidence on which to judge this will come from this trial.

For the purposes of the trial interim efficacy analyses (see Section 10.4) we have taken a position that if the proportion of patients randomised to the TRUNCATE-TB management strategy that require introduction of the standard treatment regimen for treatment failure/relapse is above 20% then this approach is unlikely to be of value for programmes or acceptable to patients (whether lower levels will be acceptable will depend on a full consideration of the risks and benefits of this approach which need to await the comprehensive evaluation at the end of the trial when the necessary trial data are available to make that decision). Ultimately the trial may yield valuable information on biomarkers that may enable the selection of an optimal treatment population that would most benefit from the strategy, but again this will not be known until the end of the trial.

2.5 BOOSTED TREATMENT REGIMENS IN THE TRUNCATE-TB MANAGEMENT STRATEGY

There are a number of potential additions to or substitutions for drugs in the standard treatment regimen that may be predicted to boost sterilising efficacy at 2 months. Allowing several modifications leads to a large number of potential boosted regimens. There are no data upon which to make a definitive prediction of which of these changes and the resulting boosted regimens would achieve the lowest relapse rate after a 2-month course. Therefore in this trial we will initially test the TRUNCATE-TB management strategy with several candidate 2 month boosted regimens and recruitment to those that do not achieve promising early results will be discontinued (see trial design, below).

2.5.1 PRINCIPLES USED IN CONSTRUCTING THE BOOSTED REGIMENS

The boosted treatment regimens used for the initial 2 month treatment have been selected based on consideration of potential maximal sterilising efficacy, absence of known pharmacokinetic drug interactions, as well as likely tolerability and safety. In general, we have started from the standard treatment regimen, made 2-3 modifications predicted to enhance sterilising activity and kept 5 drugs in each boosted regimen (in contrast to the 4 drugs in the standard regimen). The experimental arms retain, wherever possible, the two key sterilising drugs of the standard-of-care regimen: rifampicin and pyrazinamide. We also keep isoniazid which, although it is not traditionally considered to have much sterilising activity, does have potent early bactericidal activity that may help to diminish the chances of early selection of resistance. The benefit of ethambutol in these regimens is uncertain, but we have retained it where it allows the use of fixed-dose combinations and there is a net decrease in the pill burden. We have also retained it in the bedaquiline arm so that all boosted regimens have 5 drugs (instead of the usual 4-drug therapy).

For the modifications intended to increase sterilising activity, we considered adding drugs for which there was human randomised controlled trial data suggesting they possess potent sterilising activity. We examined each of the potential modifications from safety, tolerability and PK perspectives to ensure that there would be unlikely to be major overlapping toxicity or clinically-significant PK interactions. We also examined data from animal models and clinical studies where individual drugs had been combined to ensure that there was no evidence of antagonism, and preferably evidence of synergy to support a particular choice of combination. We also solicited the opinions of independent experts with particular clinical and non-clinical knowledge of individual drugs and took this expert opinion into account in the final choice of boosted regimens.

In summary, the modifications to the standard regimen (Arm A in the trial) are:

- In Arms B and C we have increased the dose of rifampicin to 35mg/kg and added either linezolid or clofazimine respectively
- In Arm D we have substituted rifapentine for rifampicin and added levofloxacin and linezolid (ethambutol removed to limit the pill burden)
- In Arm E we have substituted bedaquiline for rifampicin (interactions prevent the use of the two together) and added linezolid (levofloxacin avoided because of potential combined QTc prolongation with bedaquiline)

In the following sections we outline the evidence for the predicted sterilising efficacy and safety of each of the drug modifications and the resulting boosted regimens. The PK of the individual drugs is well established and there are no known or predicted drug interactions within the regimens that are likely to affect drug levels to an extent where clinical efficacy might be impaired or where toxicity

might be increased. Information on the PK of the individual drugs and potential interactions within the regimens is summarised in Appendix 13.

The drugs used are licensed drugs (for TB or other indications) and further information is available from the summary of product characteristics (SPC) for each drug. A comprehensive summary of all side effects of the drugs and interactions is included in the TRUNCATE-TB Clinical Management Guide that will be provided to site investigators and updated periodically during the trial (Section 6.1.2).

These boosted regimens will be given for 8 weeks, with the duration extended to up to 12 weeks for missed doses or if persistent clinical disease at or after 8 weeks (Section 6.8). No more than 8 weeks (56 doses) of linezolid will be given (Section 6.8).

2.5.2 ARM B: RIFAMPICIN (35MG/KG), ISONIAZID, PYRAZINAMIDE, ETHAMBUTOL, LINEZOLID

Evidence for predicted sterilising effects of regimen modifications:
In this regimen the rifampicin dose is increased to 35mg/kg which has been shown to increase rate of culture conversion.[20-22] Linezolid has also been added, which has been shown to have sterilising activity in an *in vitro* and *in vivo* model of latent TB.[23] A randomised controlled trial in XDR-TB showed additional ≈30% culture conversion with linezolid above optimised background alone at 60 days.[24]

Evidence for predicted efficacy of the combination:
High dose rifampicin (35mg/kg) has been given with the other standard TB drugs used in this regimen (isoniazid, pyrazinamide, ethambutol) in a 12-week trial.[22] Linezolid shows additive activity with rifampicin in an *in vivo* model,[23] and is currently being assessed with rifampicin, isoniazid and pyrazinamide in a DS-TB trial.[25]

Evidence for predicted safety and tolerability of the combination:
Clinical trials of rifampicin 20mg/kg and 35mg/kg used with standard drugs for 8-12 weeks have shown the combination to be safe and well tolerated.[21,22,26] In particular there does not appear to be a higher incidence of drug-induced hepatitis.[21] If gastrointestinal intolerance develops, the dose may be reduced to 20mg/kg (and still retain higher sterilising efficacy).[22] Linezolid may cause myelosuppression (18% of patients in a meta-analysis),[27] but there is no reason to expect this to be enhanced by the other drugs in the regimen (rifampicin is often given with linezolid for Gram-positive infections).[28] As the haematological toxicity of linezolid is dose-dependent this will be addressed by dose reduction (300mg once daily will maintain levels above the MIC in most patients).[24] Nervous system toxicity, the other main side effect of linezolid, is unlikely to occur with only 8 weeks of treatment (peripheral and optic neuropathy are duration-dependent and usually seen after 5 months of treatment).[27] The mechanism is different to that of isoniazid neuropathy , and there is no reason to expect synergistic neuropathic effects (prophylaxis for isoniazid neuropathy with pyridoxine will be given to further mitigate the risk).

2.5.3 ARM C: RIFAMPICIN (35MG/KG), ISONIAZID, PYRAZINAMIDE, ETHAMBUTOL, CLOFAZIMINE

Evidence for predicted sterilising effects of regimen modifications:
The evidence for increased sterilising activity of rifampicin given in Arm B. Clofazimine has been used for decades in treatment of leprosy (a mycobacterial infection), and *in vitro* and *in vivo* evidence indicates it has sterilising activity against persistent *Mycobacterium tuberculosis.*[29] Observational cohort data and a randomised controlled trial have demonstrated sterilising activity in clinical TB.[30,31] A further potential benefit is that clofazimine has a long terminal half-life (70 days) and may therefore have prolonged sterilising activity after the end of treatment.[32]

Evidence for predicted efficacy of the combination:
The evidence for the efficacy of high dose rifampicin in combination with standard drugs is described above (Arm B). Isoniazid and clofazimine are synergistic *in vitro*.[33] Clofazimine with standard TB drugs shortens treatment duration in a mouse model, [34] and has been given with pyrazinamide and ethambutol in MDR regimens.[35]

Evidence for predicted safety and tolerability of the combination:
For evidence of safety and tolerability of high-dose rifampicin with standard TB drugs, see above (Arm B). Clofazimine has been given in regimens with pyrazinamide, ethambutol and isoniazid for MDR-TB with no added toxicity.[36] The safety of clofazimine in the dose we will use is known from extensive use in leprosy.[37] In TRUNCATE-TB we will use approximately half the total dose of clofazimine that is used for WHO–recommended treatment of multibacillary leprosy.[38] The common side effect of clofazimine used in patients with MDR-TB is vomiting and reversible skin discolouration.[39] The dose of clofazimine will be reduced or stopped in the event of severe skin pigmentation.

2.5.4 ARM D: RIFAPENTINE, ISONIAZID, PYRAZINAMIDE, LINEZOLID, LEVOFLOXACIN

Evidence for predicted sterilising effects of regimen modifications:
In this regimen we have substituted rifapentine for rifampicin. Rifapentine is more active than rifampicin in TB mouse models.[40,41] Comparison of rifapentine (10mg/kg) with rifampicin in a clinical trial showed no advantage in overall culture conversion,[42] but subsequent modelling indicated a reduction in time to culture conversion by 4 weeks in patients receiving a higher rifapentine dose (1200mg) compared to rifampicin.[43] A dose of 1200mg is considered to be optimal for achieving sterilisation.[43,44] The second modification in this regimen is the substitition of levofloxacin for ethambutol. Ethambutol is considered a weak drug with little sterilising activity.[45] Levofloxacin has been shown to have sterilising activity *in vitro*[46-48] and in animal models.[49] Several trials that substituted other fluroquinolones (gatifloxacin, moxifloxacin) for ethambutol[13,15] showed enhanced culture conversion at 8 weeks compared to the standard regimen (although relapse rates were relatively high with a 4 month regimen,[13,15] suggesting that additional longer-term sterilising efficacy was limited). A third modification to increase sterilisation is the addition of linezolid. The evidence for increased sterilising activity of linezolid is provided under Arm B, above.

Evidence for predicted activity of the combination:
There have been no trials using levofloxacin with either the standard regimen or with rifapentine. However, a regimen containing moxifloxacin, rifapentine (low dose, 7.5mg/kg) pyrazinamide and isoniazid increased the rate of stable culture conversion by ≈15-20% at 8 weeks, compared to the standard regimen in TB patients.[50] Levofloxacin has similar activity to moxifloxacin[51] and appears to have synergy with pyrazinamide,[52] isoniazid and rifampicin.[53] Linezolid has not been given with rifapentine, but is being tested with the other standard drugs (as above).[25]

Evidence for predicted safety and tolerability of the combination:
Rifapentine has been safely administered with isoniazid, pyrazinamide and moxifloxacin.[14,42,54] There is no reason to expect additional toxicity from levofloxacin in the regimen compared to moxifloxacin (we selected levofloxacin as it has less QTc- prolongation than moxifloxacin).[55] The main concerns with linezolid (see Arm B, above) can be predicted and managed as in Arm B above.

2.5.5 ARM E: ISONIAZID, PYRAZINAMIDE, ETHAMBUTOL, LINEZOLID, BEDAQUILINE

Evidence for predicted sterilising effects of regimen modifications:
In this regimen we substitute bedaquiline for rifampicin. As rifampicin increases bedaquiline clearance by 4.78-fold, we cannot administer both together.[56] Bedaquiline is a new drug that inhibits mycobacterial ATP synthase, which is likely a critical pathway even in dormant organisms.[57-59]

Bedaquiline has shown greater sterilising activity than rifampicin in animal models.[60] Bedaquiline increases sputum culture sterilisation at 2 months by approximately 40% in MDR-TB,[61] and adds 26% to overall cure rates[62] (equivalent or greater than the effects of rifampicin added to early treatment regimens).[63,64] A further potential advantage of bedaquiline over rifampicin is its long terminal half-life (164 days) so it may have prolonged sterilising activity after the end of treatment.[65] The second modification is the addition of linezolid, evidence described under Arm B, above.

Evidence for predicted activity of the combination:
In animal models, the combination of bedaquiline and pyrazinamide has shown high sterilising efficacy.[60,66] Bedaquiline has shown potent sterilising efficacy in MDR-TB regimens that contain pyrazinamide and ethambutol.[62] The drugs in this regimen have not formally been tested together in combination in a clinical trial, but have been given in a clinical cohort.[67]

Evidence for predicted safety and tolerability of the combination:
Both bedaquiline and linezolid have been given in regimens with pyrazinamide and isoniazid for the treatment of MDR-TB and all the drugs have been administered safely together in at least one clinical cohort of patients.[67] This regimen uses the licensed dose of bedaquiline that has been given to 335 patients with MDR-TB for a duration of 6 months and was well tolerated. Bedaquiline is known to increase QTc but the risk of QTc prolongation is low when administered, as in our regimen, without other drugs with QTc-prolonging effects (no case of absolute QTc above 480ms).[68] There were more deaths in bedaquiline-treated patients in one phase 2 study (10 versus 2) but the timing and diverse aetiology strongly suggest that they are unrelated to bedaquiline use, and subsequent reports have failed to confirm this finding.[67,69] Of note, treatment will be limited to a 2-month (maximum 3-month) course, which should mitigate any risk related to dose/duration (if a risk exists). The main concerns with linezolid (see Arm B, above) can be predicted and managed as in Arm B above, and there is no reason to expect these to be exacerbated by the other drugs in the regimen.

2.6 TRIAL DESIGN: THE MULTI-ARM MULTI-STAGE APPROACH

The trial is a non-inferiority trial comparing the TRUNCATE-TB management strategy with the standard TB management strategy on the primary outcome of the proportion of patients who have unsatisfactory clinical outcome at week 96. If the TRUNCATE-TB management strategy is found to be non-inferior on this outcome in any of the arms, then the various advantages and disadvantages (to patients and programmes) can be evaluated by comparison of the arm(s) with the standard TB management strategy arm on secondary outcomes.

The trial will test the TRUNCATE-TB management strategy using different boosted regimens as separate arms, using a multi-arm multi-stage (MAMS) design that has been applied successfully in a trial of multiple regimens for prostate cancer,[70] and a TB trial.[22,71] The trial starts with multiple arms and, as it progresses, recruitment to those arms that do not show sufficient promise on an early intermediate outcome measure is discontinued whilst recruitment to the control arm and remaining boosted arms continues until sufficient numbers of patients have been enrolled to assess the outcome on the defined primary outcome measure. Recommendations about stopping or continuing arms are made by an Independent Data Monitoring Committee (IDMC).

The trial will have 4 stages at which these arms will be evaluated, termed the pilot safety, early efficacy, qualifying efficacy, and definitive efficacy and safety stage (see Figure 3, page 8). These stages are similar in objective and approach to typical phase II to III clinical trials but in the MAMS design they are combined seamlessly into a single trial with the phase transition decisions made by the IDMC as the trial progresses – a more efficient approach than the separate phases of conventional trials.[72] The stages and IDMC meetings are summarised below (see also Figure 3, page 8).

2.6.1 Stage 1: Pilot safety stage

The main purpose of this stage is to gather data on the tolerability and safety of the boosted treatment regimens. The IDMC will review tolerability and safety data when the first 10 patients enrolled in the control arm have reached 4 weeks post-randomisation.

2.6.2 Stage 2: Early efficacy stage

The main purpose of this stage is to gather data on the early efficacy of the boosted treatment regimens and their potential utility in the TRUNCATE-TB management strategy. The IDMC will conduct the first interim efficacy analysis when the first 30 patients enrolled in the control arm have reached 24 weeks post-randomisation (see Section 10.4.1). This review will make a preliminary evaluation of the sterilising efficacy of the boosted regimens to determine whether one or more arms are performing so badly as to need discontinuation, based on a markedly slower rate of culture conversion than control patients or a high treatment failure/relapse rate (Section 10.4.2). In addition the IDMC will review the safety data and any pharmacokinetic data available at that time.

2.6.3 Stage 3: Qualifying efficacy stage

The main purpose of this stage is to perform a more precise evaluation of the sterilising efficacy of the boosted regimens and their utility in the TRUNCATE-TB management strategy to be tested in the definitive phase of the trial. This stage will also continue to gather safety data on larger numbers of patients through recording of adverse events and will continue to assess for acquired drug resistance. The IDMC will conduct the second interim efficacy analysis at the end of this stage when 70 patients enrolled in the control arm have reached 24 weeks post-randomisation (see Section 10.4.1). In addition to the culture conversion and treatment failure/relapse data, all the safety data will be included together with all the pharmacokinetic data available at the time of the review.

In this stage, the eligibility criteria may be relaxed to allow entry of patients with factors considered predictive of potential higher risk of relapse (see Section 4.2.1) to ensure the generalisability of the trial results (unless instructed otherwise by the local ethics committee, or by any regulatory body that is required to approve amendments to the protocol).

2.6.4 Stage 4: Definitive safety and efficacy stage

The main purpose of this stage is to evaluate the non-inferiority of the TRUNCATE-TB management strategy in comparison to standard TB management strategy, as well as the various advantages and disadvantages of the strategy to patients and programmes. This will be the final analysis conducted at the end of the trial.

2.6.5 IDMC meetings and decisions

In addition to the fixed reviews described in these stages, the IDMC will meet every 6 months during the trial and conduct additional *ad hoc* reviews in the event of an observed high rate of early treatment failure/relapse in an arm, or if there are more than 2 cases of confirmed new drug resistance in an arm or if there are any other serious safety signals detected (Section 10.4.1). After the start of enrolment from Stage 3 of patients with factors considered predictive of potential higher risk of treatment failure/relapse, the IDMC will monitor this group as a separate subgroup (with an ad hoc meeting called if there are ≥10 confirmed treatment failure/relapses before 20 patients reach 8 weeks post-treatment across all of the boosted treatment regimens combined).

At any of their meetings, the IDMC may recommend that recruitment to all arms continues, recruitment to one or more arms stops or the recruitment to all arms stops (Section 10.4.5). From

Stage 3, the IDMC may also recommend the cessation of recruitment of patients with any or all of the factors considered predictive of potential higher risk of relapse if there is concern.

2.7 AIMS OF THE TRIAL

Primary aim: To test the hypothesis that the TRUNCATE-TB management strategy is non-inferior to the standard management strategy assessed by the proportion of patients with unsatisfactory clinical outcome at 2 years (96 weeks) after randomisation.

Secondary aim: To assess the possible advantages of the TRUNCATE-TB management strategy compared to the standard management strategy from the patient perspective (including acceptability, quality of life and clinical adverse events) and the programme perspective (including treatment adherence, default, new drug resistance and community transmission) as well as costs (to patients and programmes) and cost-effectiveness.

Other aims: To evaluate the pharmacokinetics, microbiological efficacy and toxicity of a number of boosted 8-week regimens in comparison to the standard treatment regimen. To explore the relationship between various biomarkers (clinical and laboratory parameters as well as drug exposure and possible novel laboratory biomarkers) on outcomes related to sterilisation and cure.

NOTE: this is not intended to be the definitive trial that will lead to immediate change in the way TB is treated in programmes. Rather, this trial aims to explore for the first time (and with the maximum gain in knowledge), the risks and benefits, feasiblity, acceptability and cost-effectiveness of the TRUNCATE-TB management strategy to determine its potential suitability for wider use and to identify the optimal regimen, patient group, and monitoring strategy for a subsequent definitive large-scale trial of this approach done at a programme level.

3 SELECTION OF SITES AND PRINCIPAL INVESTIGATORS

3.1 SITE/INVESTIGATOR INCLUSION CRITERIA

To participate in the trial, clinical trial sites and investigators must fulfil the criteria and be able to provide the commitments listed below.

3.1.1 Site Principal Investigator's Qualifications and Agreements

1. The principal investigator should be qualified by education, training, and experience to assume responsibility for the proper conduct of the trial at their site and should provide evidence of such qualifications through an up-to-date curriculum vitae and/or other relevant documentation requested by the Sponsor and/or the regulatory authority (ies).

2. The principal investigator should have sufficient time to properly conduct and complete the trial within the agreed trial period.

3. The principal investigator should commit to ensuring that there are adequate personnel, appropriately-trained, to perform the trial management and data management at the site (utilising the site budget to hire additional resources where needed).

4. The principal investigator should commit to ensuring that the study clinical personnel are thoroughly familiar with the appropriate use of the investigational products as described in the protocol, in the SPCs, and in other information provided by the Sponsor.

5. The principal investigator should be aware of, and should commit to comply with, the principles of ICH GCP and the applicable regulatory requirements.

6. The principal investigator/site should commit to permitting monitoring and auditing by the Sponsor, and inspection by the appropriate regulatory authority(ies)

7. The principal investigator should commit to developing a plan for community engagement that ensures appropriate communication, feedback and collaboration between site staff and the community they are working with.

3.1.2 Site Resources

1. The site should have the potential for recruiting the required number of suitable patients (directly at the site, or by referral from other centres) within the agreed recruitment period.

2. The site should have available an adequate number of qualified staff and adequate facilities for the foreseen duration of the trial to conduct the trial properly and safely. The site must have access to adequate senior clinical expertise to be able to deal with any serious complications of TB disease or of the TB medication (standard and non-standard) used in the trial.

3. The site should have a laboratory on site, or have access to a designated laboratory or laboratories that have sufficient capacity to be able to provide sputum smear, GeneXpert testing, TB liquid culture (by MGIT) and first line drug susceptibility testing (DST) (with second

line available at the site or a reference centre). Evidence of participation in appropriate quality assurance programmes should be provided by the laboratory performing each test.

4. Sites must be able to implement directly observed therapy reliably and in a manner that is likely to achieve good adherence rates.

4 SELECTION OF PATIENTS

Patients will be considered eligible for enrolment in this trial if they fulfil all the inclusion criteria and none of the exclusion criteria as defined below.

For the safety of the patients, as well as to ensure that the results of this study can be useful for making treatment decisions regarding future patients, **no exceptions** can be made to these criteria for admission to the study. Questions about eligibility criteria should be addressed prior to attempting to randomise the participant.

The criteria below apply to the initial 2 stages of the trial. These may be modified formally from stage 3 of the trial (Section 4.2.1).

4.1 PATIENT INCLUSION CRITERIA

1. Age 18 to 65 years
2. Clinical symptoms consistent with pulmonary TB and/or evidence of pulmonary TB on CXR
3. Sputum GeneXpert test positive
4. Willing to comply with the study visits and procedures
5. Resident at a fixed address that is readily accessible for visiting, within feasible travelling distance to the site and likely to remain resident there for the duration of trial follow-up
6. Willing to have directly observed therapy
7. Willing and able to provide written informed consent

4.2 PATIENT EXCLUSION CRITERIA

1. Taken more than 10 daily doses of standard anti-TB medication or fluoroquinolones during the 3 months prior to randomisation
2. Previous active TB disease for which treatment was given prior to the current episode (patients who have received isoniazid prophylaxis are eligible)
3. Known or suspected extra-pulmonary TB (in the opinion of the investigator; no specific screening tests required; if symptoms suggest extra-pulmonary involvement, patient can still be enrolled if relevant tests-performed for clinical management-exclude extra-pulmonary disease at the suspected site; pleural effusion occupying <50% of hemithorax or concomitant intra- or extra-thoracic lymphadenopathy are not exclusions)
4. Severe clinical pulmonary TB e.g. respiratory failure or complications likely to require hospital admission
5. Sputum smear 3+ on sample taken at screening (using IUATLD grading system, see Appendix 2)* [removed at time of approval of V2 protocol, depending on site-specific approval by ethics and regulatory bodies]
6. Cavity size > 4cm on screening CXR* [removed at time of approval of V2 protocol, depending on site-specific approval by ethics and regulatory bodies]
7. Presence of rifampicin resistance on GeneXpert test
8. Poorly-controlled diabetes that, in the opinion of the investigator, is unlikely to be controlled with available management strategies
9. Active malignancy requiring systemic chemotherapy or radiotherapy

10. Known Hepatitis B surface antigen positive and/or HCV antibody positive, unless liver function tests consistently within normal range for at least 2 years
11. History of myocardial infarction, congestive cardiac failure, cardiac arrhythmias or any known congenital cardiac problems
12. History of severe chronic lung disease (e.g. chronic obstructive pulmonary disease) with symptom score of ≥3 on MRC breathlessness scale (see Appendix 3)
13. History of seizures [removed at time of approval of V2 protocol]
14. Current tendinitis or history of tendinopathy associated with fluoroquinolone use [removed at time of approval of V2 protocol]
15. Symptomatic peripheral neuropathy causing greater than minimal interference with usual social and functional activities.
16. Current alcohol or drug abuse
17. Women who are currently pregnant or breast-feeding
18. Women of childbearing potential unwilling or unable to use appropriate effective contraception (i.e. barrier methods such as condoms, hormonal contraception, intra-uterine device) for the first 6 months of the trial
19. Known allergy to one or more of the study drugs
20. Taking a concomitant medication that has a known or predicted interaction with any of the study drugs to which the patient might be randomised, or is known to prolong the QTc interval (if the concomitant medication can be stopped after randomisation, or there is a feasible alternative medication available, the patient need not be excluded)
21. Taking any immunosuppressive drugs or use of systemic corticosteroids for more than 2 weeks prior to screening
22. Colour blindness detected by Ishihara test
23. 12-lead ECG at screening shows QTc greater than 450ms (see Appendix 4) and/or any other clinically-significant abnormality such as arrhythmia or ischaemia
24. Any of the following laboratory parameters at screening:
    - Absolute neutrophil <1000 cells/µL, haemoglobin <7.0 g/dL, OR platelet count <50,000 cells/mm^3
    - Creatinine clearance of <60ml/min (calculated using Cockcroft-Gault equation, (Appendix 5)
    - ALT greater than 3 times the upper limit of normal
    - Uncorrected serum potassium <3.5 mmol/L
25. HIV antibody positive at screening* [modified to the text in 4.2.1 below, at time of approval of V2 protocol, depending on site-specific approval by ethics and regulatory bodies]
26. Any other significant condition (e.g. psychiatric illness, chronic diarrhoeal disease), that would, in the opinion of the investigator, compromise the patient's safety or outcome in the trial or lead to poor compliance with study visits and protocol requirements
27. Participation in other clinical intervention trial or research protocol (participation in other studies that do not involve an intervention may be allowed, but this must be discussed and approved by the Chief Investigator)

* Criteria may be modified from stage 3 of the trial, see below

4.2.1 Modifications for exclusion criteria from Stage 3

In order to maximise generalisability, the exclusion criteria marked (*) that are considered to be factors predictive of potential higher risk of relapse, will be modified as follows from stage 3 (qualifying efficacy stage) of the trial (unless instructed otherwise by the oversight local ethics committee, or by any regulatory body that is required to approve amendments to the protocol, see below):

Criterion 5 (smear positive 3+) and criterion 6 (more than 4cm cavitation) will be removed.

Criterion 25 (HIV antibody positive), will be modified as follows:

HIV antibody positive, unless **all** the following additional criteria are met:

(i) Current CD4 T-cell count >200cells/mm^3
(ii) Not currently taking ART
(iii) Patient and doctor willing to defer starting ART until 8 weeks after enrolment (up to 12 weeks, if randomised to a boosted regimen the duration of which is extended)
(iv) have access to ART and are willing to start when recommended by local treatment guidelines, at any point from 8 weeks (or 12 weeks, as above) after enrolment

The changes in these criteria will be implemented on a site-specific basis. The criteria will not be changed at sites where either the ethics committee or regulatory agency responsible for trial oversight has stated in writing (at the time of reviewing the original protocol, or a protocol amendment) that they do not agree to the criteria being changed. The objection to change may be specific for one, two or all three of the criteria; only the criteria for which no objection has been raised will be changed.

To implement the change, the eligibility checklist in use at that site will be modified (one, two or all three criteria changed, as appropriate). From that time, the site will be permitted to randomise patients into the higher-risk stratum (see section 5.2). The PI would retain discretion not to enrol individual patients considered to be at high risk of relapse if they did not consider them suitable for the trial, even if they met all the eligibility criteria.

4.3 SCREENING PROCEDURES AND PRE-RANDOMISATION INVESTIGATIONS

4.3.1 PRE-SCREENING CHECK AND INFORMED CONSENT

Prior to taking consent for screening or performing any screening related procedures, a check of medical and drug history should be performed to ensure that the patient meets the basic eligibility criteria.

Patients who appear suitable on this initial pre-screening will be given adequate information about the trial together with a Consent Form and given an opportunity to ask questions about the trial. It will be made clear to the participant that they are free to refuse to participate in the trial or to later withdraw from the trial without incurring any penalty or affecting their access to standard clinical care. The investigator will ensure that the patient understands the known risks and possible benefits of participating in the study.

After reading the Consent Form and after receiving answers to any further questions that they may have about the trial, if the patient is willing to proceed then the patient and Investigator must both sign and date two copies of the Consent Form. One copy of the consent form will be given to the patient and one copy will be retained in the trial file. It will be documented in the patient's medical record that the patient is in a clinical trial and that consent has been taken. This must be done before any trial-specific screening procedures are carried out.

All individuals who have signed the Consent Form will have their name, date of birth and clinic number recorded in a screening log, together with their Trial Number (when allocated), or the reason the

screened individual was not enrolled (e.g. ineligible based on screening test results). The screening log must be stored by the site investigator in a secure place only accessible to appropriate clinic staff.

4.3.2 SCREENING VISIT

Screening assessments will be performed as listed below and as summarised in the Trial Schedule (page 9) and in the assessments (Section 7.3).

- Review of medical history, demographics, standard TB symptoms (cough, one or more episodes of haemoptysis, fever, pleuritic chest pain, weight loss, night sweats), medication
- Physical examination-vital signs and comprehensive systems examination, including Ishihara test
- 12 lead ECG (if the QTc reading is >450 ms the ECG may be repeated once; if QTc ≤450 ms this result can be used for screening purposes)
- CXR (need not be repeated for screening purposes if done in previous 7 days and the film is available for evaluation by the research team)
- Urine pregnancy test for women of child bearing potential
- Sputum collection for smear and GeneXpert (GeneXpert need not be repeated if a positive result is available from a test performed earlier during this illness episode, done in study-approved laboratory and results are available to research team)
- Blood tests
    - for standard safety tests (FBC, electrolytes (sodium, potassium, creatinine), LFTs (ALT, alkaline phosphatase, bilirubin), glucose, amylase/lipase, magnesium and calcium (need not be repeated at screening if done in previous 2 days)
    - HIV antibody test (need not be repeated if done in the previous 30 days)
    - CD4 count (only if patient HIV positive and trial is open to enrolment of HIV-infected participants at that site)

Repetition of individual components of screening or complete re-screening is permitted for clinical or logistical reasons.

5 RANDOMISATION AND ENROLMENT

5.1 RANDOMISATION VISIT (BASELINE, DAY 0)

The randomisation visit should take place as soon as possible after screening and at the latest before the patient has taken more than 10 daily doses of TB medication (in order to meet eligibility criteria). The randomisation visit may be performed on the same day as screening provided the patient is confirmed to meet all eligibility criteria (see Chapter 4).

Randomisation should be performed while the patient is present in the clinic. Prior to performing randomisation, patients should be given a further opportunity to ask questions about the trial and to confirm that they wish to proceed.

Prior to randomisation the investigator should review the eligibility criteria to ensure that all have been fulfilled. The investigator should also re-assess the patient to ensure that there has been no significant change in the patient's clinical condition since the time of screening that results in violation of eligibility criteria. Any concomitant medications that the patient is taking will be reviewed.

The results of all the evaluations carried out for screening must be reviewed prior to randomisation. If the screening ECG has been deferred to the day of randomisation, the result of this ECG must be reviewed prior to randomisation. If the screening CXR was done more than 7 days prior to randomisation the CXR should be repeated and reviewed prior to randomisation. The blood tests scheduled for the randomisation visit do not need to be performed (or the results reviewed) prior to randomisation.

The following will be performed at the randomisation visit (day 0). Details of the assessments are described in Section 7.3.

- Review of eligibility criteria, medical history, demographics, symptoms, medication
- Physical examination including test for visual acuity
- Healthcare utilisation
- EQ-5D and MOS-HIV questionnaire
- Socioeconomic evaluation
- 12 lead ECG
- CXR need not be repeated if performed in previous 7 days and if available for evaluation by the research team. The same X-ray film may be used for both screening and Day 0 if within the specified time frame for each visit.
- Urine (10ml) for storage
- Sputum collection for smear, liquid culture (MGIT) and DST
- Blood tests
    - for standard safety monitoring tests
    - for drug levels at 1-hour post-dose (if split dose, times from first drug ingestion; window 1-2 hours post-dose)
    - for plasma storage (10ml EDTA) and for host RNA storage (5ml RNA preservation tube)

5.2 PROCEDURE FOR RANDOMISATION

Randomisation will be performed using a centralised computer-generated randomisation list that has been pre-programmed by the trial statistician. A manual randomisation process will be set up to cover

any instances when the main electronic system is not working. The details of the patient's treatment allocation and trial number will be notified to the site immediately online.

An enrolment log that contains the patient's trial number, allocated drug arm, and the date of randomisation will be completed for all these patients.

Randomisation will be web-based and stratified by site and potential relapse risk (Lower risk: sputum screening smear negative and screening CXR cavities ≤ 4 cm and HIV negative; Intermediate risk: screening sputum smear positive and screening CXR cavities ≤ 4 cm and HIV negative; Higher risk: screening sputum smear grade 3+ or screening CXR cavities > 4 cm or HIV positive). Enrolment of patients with potential higher relapse risk will only be done from stage 3 (subject to the conditions described in sections 4.2.1).

Where patients are unable to produce sputum at screening, the smear result will be regarded as negative for the purposes of relapse risk stratification.

Patients will be allocated in equal proportions to each of the regimens open for randomisation. Randomised permuted blocks with variable block size will be used to balance the allocation of regimens.

5.3 POST-RANDOMISATION PROCEDURES AND FOLLOW-UP

The details of post-randomisation procedures and assessments are outlined in the Trial Schedule (page 9). Trial visit schedules will be prepared for each patient at randomisation. The target dates for trial visits are determined by the date of randomisation and are not affected by subsequent events. The schedule defines visit dates (with windows) necessary for data collection, but the patient may be seen more frequently for clinical care as needed.

Patients will also be given a card with the contact details of the trial research team for use in case of emergency.

5.4 CO-ENROLMENT GUIDELINES

Patients will not ordinarily be permitted to participate in any other clinical intervention trial or research protocol while in the trial. Participation in other studies that do not involve an intervention may be acceptable. Any possible co-enrolment should be discussed with the Chief Investigator.

6 TREATMENT OF PATIENTS

6.1 INTRODUCTION

This chapter outlines the approach to management of patients in the trial. It is essential that patients are managed as closely as possible to the strategy and regimens to which they have been allocated by randomisation. However, in view of the considerable heterogeneity of patients it is recognised that there will be some variation from the precise management specified in the protocol. The protocol allows a degree of clinician discretion in adapting therapy to individual patient circumstances. In particular there is flexibility for the drug and doses in the standard regimen and boosted regimens to be modified (for intolerance or drug toxicity; principles described under the individual regimens below), or for the duration of treatment to be extended (for missed doses, treatment interruption, persistent clinical disease at week 8, or other reasons; principles described in Section 6.8.1). Additional management advice will be set out in the Clinical Management Guide. Any variation from the management set out in the protocol and the options in the Clinical Management Guide should be discussed with the Chief Investigator, preferably prior to implementation, and be notified to the coordinating centre.

The patients randomised to the standard TB management strategy will receive initial treatment for 6 months (24 weeks, 168 daily doses) with standard combination therapy.

Patients randomised to the TRUNCATE-TB management strategy will receive an initial period of treatment for 2 months (8 weeks, 56 daily doses) with one of the boosted regimens. Treatment with the boosted regimen may be extended for up to 12 weeks (maximum of 84 daily doses) for missed doses or for those with persistent symptoms and positive smear at week 8.

Patients will be observed closely after end of treatment and for those that relapse (anticipated to be the minority in both treatment arms), retreatment will be given using a 6 month course of standard combination therapy (if the initial treatment comprised a boosted regimen only) or an empirical 6 to 8-month regimen if standard therapy was used in whole or in part for the initial treatment.

The boosted regimens each use a different combination of drugs for the initial treatment period. The number of boosted arms being tested at any time will depend on the stage of the trial and the outcomes of interim analyses, as arms may be stopped early.

All drugs used in the control and boosted combinations listed below will be given orally. A fixed dose combination (FDC) will be used in place of the individual drugs wherever possible, in order to minimise pill burden. Treatment will be administered daily and using DOT.[3]

6.1.1 ADVICE TO PATIENTS

Following randomisation, patients will be supplied with a regimen-specific trial guide. This will detail the common side effects of the medications, any regimen-specific food requirements, and advice of what actions to take in the event of a delay in taking the prescribed dose. Patients will be strongly advised to restrict intake of alcohol (this may increase the risk of hepatotoxicity). Female patients will be advised to use effective contraceptive measures during treatment (and for 6 months after enrolment).

6.1.2 TRUNCATE-TB CLINICAL MANAGEMENT GUIDE

The TRUNCATE-TB Clinical Management Guide will be provided to site investigators and updated periodically during the trial. The Guide will include information on food requirements and the action

to take for delayed doses or treatment interruptions. The guide will also include management plans for drug intolerance and toxicity, as well as recommendations on regimen modifications in these situations. A comprehensive list of main side effects of the drugs, interactions and contraindications will be included and sites will also be provided with updated SPCs for each of the study drugs, as and when these are changed by the manufacturers.

6.1.3 FOOD REQUIREMENTS AND TIMING OF DOSES

The food recommendations differ for the individual medications, with most of the study regimens containing some medications that are recommended to be taken with food and some that are recommended to be taken without. Food recommendations are standardised for each regimen to simplify ease of administration (timing prioritised to optimise absorption of the drug considered likely to have the greatest sterilising activity, i.e. rifampicin, rifapentine, or bedaquiline). The food recommendations for the individual drugs will be provided in the Clinical Management Guide, in case specific clinical management issues arise, that require separation of dosing.

6.2 STANDARD TB TREATMENT ARM

6.2.1 ARM A: 24 WEEKS RIFAMPICIN, ISONIAZID, PYRAZINAMIDE, ETHAMBUTOL

TABLE 2A: DOSES OF DRUGS IN ARM A IN INTENSIVE PHASE

DRUG	<40KG	40-54 KG	55-70KG	≥71KG
Rifampicin	300mg	450mg	600mg	750mg
Isoniazid	150mg	225mg	300mg	375mg
Pyrazinamide	800mg	1200mg	1600mg	2000mg
Ethambutol	550mg	825mg	1100mg	1375mg

Treatment will be taken once daily on empty stomach.

For the first 8 weeks of this Arm, the 4-drug FDC tablets recommended by WHO[3] (containing rifampicin 150mg, isoniazid 75mg, pyrazinamide 400mg and ethambutol 275mg) will be used as per weight by the above schedule.

At 8 weeks, the treatment will change to the 2-drug FDC tablets recommended by WHO (rifampicin 150mg and isoniazid 75mg) for the remaining 16 weeks of treatment, with dosing as follows (once daily, on empty stomach).

TABLE 2B: DOSES OF DRUGS IN ARM A CONTINUATION PHASE

DRUG	<40KG	40-54 KG	55-70KG	≥71KG
Rifampicin	300mg	450mg	600mg	750mg
Isoniazid	150mg	225mg	300mg	375mg

Treatment may by modified for missed doses or treatment interruption (Section 6.8.1). Where the national or local TB treatment guidelines differ from the above, the investigator may be permitted to modify the treatment approach. However this should be discussed with the Chief Investigator first.

<u>Regimen modification for side-effects:</u>

Arthralgia and swelling of one or more joints may occur in patients who are receiving pyrazinamide. Treatment with non-steroidal anti-inflammatory drugs will be attempted initially, followed by dose reduction of pyrazinamide if necessary to control symptoms.

Gastrointestinal intolerance, usually associated with rifampicin or pyrazinamide, will be initially treated with anti-emetics and/or alteration of dose schedule and food ingestion.

Hepatitis and other drug intolerances such as rash will be managed by cessation or dose reduction of individual drugs or temporary cessation of the entire regimen.

Individual drugs may be used instead of FDC if required, with daily doses adjusted according to weight as recommended by WHO (maximum doses for pyrazinamide and ethambutol are not specified by WHO; these are taken from respective SPCs): rifampicin 10mg/kg (rounded to nearest 150mg, maximum 600mg); isoniazid 5mg/kg (rounded to nearest 100mg, maximum 300mg); pyrazinamide 25mg/kg (rounded to nearest 500mg, maximum 2000mg); ethambutol 15mg/kg (rounded to nearest 100mg, maximum 1600mg).[3]

Detailed management guidance for these and other toxicities will be provided to the clinical sites in the TRUNCATE-TB Clinical Management Guide.

6.3 BOOSTED REGIMENS FOR USE IN THE TRUNCATE-TB MANAGEMENT STRATEGY

The following is a list of the planned boosted 8-week regimens that will be tested in this trial. Randomisation to the control arm will continue throughout the duration of the trial, but randomisation to some of the experimental regimens may be discontinued following interim evaluations of the trial data.

The TRUNCATE-TB management strategy involves re-treatment with a standard combination regimen in the event of relapse or treatment failure.

6.3.1 ARM B: 8 WEEKS RIFAMPICIN (HIGH-DOSE), ISONIAZID, PYRAZINAMIDE, ETHAMBUTOL AND LINEZOLID

TABLE 3: DOSES OF DRUGS IN ARM B

DRUG	<40KG	40KG- 54KG	55KG - 70KG	≥71KG
Rifampicin	35mg/kg (rounded to nearest 150mg, maximum 2100mg)			
Isoniazid	150mg	225mg	300mg	375mg
Pyrazinamide	800mg	1200mg	1600mg	2000mg
Ethambutol	550mg	825mg	1100mg	1375mg
Linezolid	600mg			

Treatment will be taken once daily on empty stomach.

<u>Regimen modification for side-effects:</u>

The doses given in the table above will be delivered using a FDC of rifampicin, isoniazid, pyrazinamide and ethambutol with supplementary rifampicin (150mg, 300mg, 450mg or 600mg capsules) to reach the total rifampicin dose and separate linezolid. If the FDC is not used, then the daily doses of the individual drugs will be as follows: rifampicin 35mg/kg (rounded to nearest 150mg, maximum 2100mg); isoniazid 5mg/kg (rounded to nearest 100mg, maximum 300mg); pyrazinamide 25mg/kg

(rounded to nearest 500mg, maximum 2000mg); ethambutol 15mg/kg (rounded to nearest 100mg, maximum 1600mg); linezolid 600mg.

Gastrointestinal intolerance usually associated with rifampicin, pyrazinamide, or linezolid, will be initially treated with anti-emetics and/or alteration of dose schedule and food ingestion, and subsequently by dose reduction if needed.

For anaemia or low white cell count, the dose of linezolid will initially be reduced to 300mg. Low platelet count may be caused by linezolid or rifampicin. It will be managed by cessation or dose reduction of linezolid initially and then rifampicin.

Detailed management guidance for these and other toxicities will be provided to the clinical sites in the TRUNCATE-TB Clinical Management Guide.

6.3.2 Arm C: 8 weeks rifampicin (high-dose), isoniazid, pyrazinamide, ethambutol and clofazimine

TABLE 4: DRUG DOSES IN ARM C

DRUG	<40KG	40KG- 54KG	55KG - 70KG	≥71KG
Rifampicin	35mg/kg (rounded to nearest 150mg, maximum 2100mg)			
Isoniazid	150mg	225mg	300mg	375mg
Pyrazinamide	800mg	1200mg	1600mg	2000mg
Ethambutol	550mg	825mg	1100mg	1375mg
Clofazimine	200mg			

Treatment will be taken once daily on empty stomach.

<u>Regimen modification for side-effects:</u>

The doses given in the table above will be delivered using a FDC of rifampicin, isoniazid, pyrazinamide and ethambutol with supplementary rifampicin (150mg, 300mg, 450mg or 600mg capsules) to reach the total rifampicin dose and separate clofazimine. If the FDC is not used, then the daily doses of the individual drugs will be as follows: rifampicin 35mg/kg (rounded to nearest 150mg, maximum 2100mg); isoniazid 5mg/kg (rounded to nearest 100mg, maximum 300mg); pyrazinamide 25mg/kg (rounded to nearest 500mg, maximum 2000mg); ethambutol 15mg/kg (rounded to nearest 100mg, maximum 1600mg); clofazimine 200mg.

Gastrointestinal intolerance, usually associated with clofazimine, rifampicin or pyrazinamide will be initially treated with anti-emetics and/or alteration of dose schedule and food ingestion, and subsequently by dose reduction if needed. If there is skin pigmentation as a result of clofazimine therapy that is of concern to the patient, the dose will initially be reduced to 100mg once daily. If the patient is unable to tolerate the pigmentation, clofazimine will be stopped and the remaining drugs in the regimen will continue (see Clinical Management Guide).

Detailed management guidance for these and other toxicities will be provided to the clinical sites in the TRUNCATE-TB Clinical Management Guide.

6.3.3 ARM D: 8 WEEKS RIFAPENTINE, ISONIAZID, PYRAZINAMIDE, LINEZOLID AND LEVOFLOXACIN

TABLE 5: DRUG DOSES IN ARM D

DRUG	<40KG	40KG- 54KG	55KG - 70KG	≥71KG
Isoniazid	5mg/kg rounded to nearest 100mg		300mg	
Pyrazinamide	25mg/kg rounded to nearest 500mg	1000mg	1500mg	2000mg
Rifapentine	1200mg			
Linezolid	600mg			
Levofloxacin	1000mg			

Treatment will be taken once daily with food.

<u>Regimen modification for side-effects:</u>

Gastrointestinal intolerance, usually associated with pyrazinamide, linezolid or levofloxacin will be initially treated with anti-emetics and/or alteration of dose schedule and food ingestion, and subsequently by dose reduction if needed.

Alteration in the blood count is a side effect of linezolid. If anaemia, low white cell count or thrombocytopenia are noted, this will be managed initially by dose reduction to 300mg, followed by cessation of the drug if necessary.

QTc prolongation may be caused by levofloxacin. If the QTc (confirmed) is more than 450ms, other contributing factors will be sought and removed/corrected where possible. If QTc is greater than 500ms, levofloxacin will be stopped.

Detailed management guidance for these and other toxicities will be provided to the clinical sites in the TRUNCATE-TB Clinical Management Guide.

6.3.4 ARM E: 8 WEEKS BEDAQUILINE, ISONIAZID, PYRAZINAMIDE, ETHAMBUTOL AND LINEZOLID

TABLE 6: DRUG DOSES IN ARM E

DRUG	<40KG	40KG- 54KG	55KG - 70KG	≥71KG
Bedaquiline	400 mg once daily for 2 weeks then 200mg three times a week			
Isoniazid	5mg/kg rounded to nearest 100mg		300mg	
Pyrazinamide	25mg/kg rounded to nearest 500mg	1000mg	1500mg	2000mg
Ethambutol	15mg/kg (rounded to nearest 100mg, maximum 1600mg)			
Linezolid	600mg			

Treatment will be taken once daily with food.

<u>Regimen modification for side-effects:</u>

If abnormalities of magnesium, calcium or potassium levels are detected at screening, these will be corrected prior to starting the boosted regimen (patient will be treated with standard treatment until this has been achieved).

Gastrointestinal intolerance, usually associated with bedaquiline, pyrazinamide or linezolid will be initially treated with anti-emetics and/or alteration of dose schedule and food ingestion, and subsequently by dose reduction if needed.

QTc prolongation may be caused by bedaquiline. If the QTc (confirmed) is more than 450ms, other contributing factors will be sought and removed/corrected where possible. If the corrected QTc interval is greater than 500ms, bedaquiline will be stopped.

Alteration in the blood count is a side effect of linezolid. If anaemia, low white cell count or thrombocytopenia are noted, this will be managed initially by dose reduction to 300mg, followed by cessation of the drug if necessary.

Detailed management guidance for these and other toxicities will be provided to the clinical sites in the TRUNCATE-TB Clinical Management Guide.

6.4 TREATMENT SUPPLY AND SUPERVISION

All study drugs will be supplied to the trial sites for dedicated use in the trial and will be stored separately from routine clinic drug supplies in a designated area. WHO pre-qualified drugs will be used where possible. Procedures will be described in detail in the TRUNCATE-TB pharmacy manual.

Supervised treatment refers to helping patients to take their TB medications regularly and to complete TB treatment. Treatment will be given by DOT. For the purposes of TRUNCATE-TB, treatment will be administered on a supervised basis for the entire duration of the boosted treatment regimens and the intensive phase of the standard TB treatment arm. Depending on national guideline recommendations, the supervision may be longer for patients receiving the 6-month standard treatment regimen. Treatment will be supervised by a named Treatment Supporter who must check that the drugs have been swallowed, and record this on the treatment card. The treatment supporter will usually be a clinic staff member, but if it is unfeasible for the patient to attend to receive their medications daily, then other options may be considered such as a community health worker or a trained and supervised local community member.

6.5 GENERAL MANAGEMENT AND CONCOMITANT MEDICATIONS

The sites will be provided with a Trial Clinical Management Guide that will detail the clinical management procedures for managing adverse events that will include provision for dose reduction or interruption where needed and for a switch to alternative treatment (the standard treatment) where dose modification is not successful in ameliorating the side-effects. Staff will be trained in these clinical management protocols at the site initiation visit with periodic retraining at monitoring visits and investigator meetings. The Guide will be updated and circulated regularly, along with updates to the SPCs as available, by the coordinating centre. A clinical management core will be formed for the trial comprising experts in TB management and the drugs used in the trial and site staff will be able to consult this group (through the coordinating centre) for advice on difficult clinical management problems.

Concomitant medication will be reviewed at the time of randomisation and drugs with significant interactions with study medications will be stopped or switched as clinically appropriate.

All patients will receive pyridoxine 10mg once daily for the duration of their TB treatment as supplementation to prevent isoniazid-induced peripheral neuropathy. The dose of pyridoxine may be increased at clinician discretion if other risk factors for neuropathy are present.

Patients will be advised of the risks of medication interactions, and will be advised to consult with the trial team before taking any new medication prescribed for other conditions.

The managing clinicians may prescribe additional medications that they feel are appropriate for managing the patient. However, care must be taken to avoid prescribing any medication that has a known or predicted interaction with one or more of the trial drugs. The complete list of drug interactions and contraindications are listed in the Clinical Management Guide and under individual drug SPCs.

If prescription of a concomitant medication is unavoidable, then consideration should be given to changing the TB treatment regimen, for the shortest duration possible, to avoid the offending drug. This is regarded as a period of treatment interruption and TB treatment will be managed as described in Section 6.8.1. Switches from a boosted treatment permanently to the standard of care should be discussed, where possible, with the Chief Investigator prior to switching, and such switches will need to be reported to the coordinating centre.

All non-trial treatment taken by the patient will be recorded.

6.6 MANAGEMENT OF HIV

HIV-infected patients may be enrolled in the trial (only from stage 3, subject to conditions described in section 4.2.1), but must have a CD4 count ≥200 cells/mm^3 and not be on ART at trial entry.

They will initiate an ART regimen (recommended by WHO guidelines and provided by national programmes) at the CD4 count threshold recommended in local treatment guidelines. ART initiation should be deferred until after they have completed the boosted regimen (8 weeks) or intensive phase of standard regimen (8 weeks). Trials show no benefit to starting ART before week 8 of TB treatment if CD4 count is above 50 cells/mm^3.[73] In the event that the boosted regimen requires extension beyond 8 weeks (this is limited to 12 weeks), the treating clinician may start ART at 8 weeks if it is considered that the risks of further deferral are too great, but in such cases the boosted regimen may need to be converted to standard TB treatment to avoid potential interactions or drug toxicity. Further information on treatment options will be provided in the Clinical Management Guide.

Patients who require re-treatment for TB during the trial will be managed as described in Section 6.11. The TB and the ART regimens will be reviewed and adjusted as necessary to minimize treatment interactions. Further information will be provided in the Clinical Management Guide.

6.7 MANAGEMENT OF PREGNANCY

Pregnant women will not be enrolled in the trial and all women of childbearing potential will be advised to use effective contraception during the first 6 months of the trial (to cover the period of standard treatment and to allow a washout period following treatment with the boosted regimens).

If a pregnancy occurs in a woman whilst taking the standard TB treatment regimen, no treatment changes are required. All 4 first line drugs (isoniazid, rifampicin, ethambutol and pyrazinamide) have an excellent safety record in pregnancy and are not associated with human foetal malformations.[74]

If pregnancy occurs in a woman whilst taking a boosted regimen, the regimen will be immediately switched to the standard TB treatment regimen to complete a course of 6 months' treatment (with the intensive phase or continuation phase shortened by the number of doses of the boosted regimen taken prior to treatment switch, as described in Section 6.8.4). Switch is required because at present there are inadequate data for some of these drugs in pregnant women (summary of current safety information provided in Appendix 9).

All pregnancies occurring in TRUNCATE-TB should be reported to the coordinating centre immediately (see Section 8.2.3) and will be followed until their outcome is known.

6.8 MODIFICATION OF INITIAL TREATMENT REGIMEN IN THE TRUNCATE-TB MANAGEMENT STRATEGY

Initial treatment will be for 8 weeks with a boosted treatment regimen as per the randomised allocation. However, this regimen may need to be modified in a number of situations, either by extension of the boosted regimen or by switch to the standard treatment regimen. Detailed guidance will be provided in the Clinical Management Guide. The principles are described below and illustrated in Figure 1 (page 6).

6.8.1 MODIFICATION FOR MISSED DOSES, TREATMENT INTERRUPTION OR TREATMENT DEFAULT

A missed dose of the treatment regimen is defined as taking less than 50% of the protocol-mandated starting dose of one or more of the component drugs in a regimen on a particular day with the following exceptions:

- (i) rifampicin dose reduction to the standard dose for toxicity or intolerance, will not count as a missed dose
- (ii) linezolid cessation for toxicity would not count as missed dose once 42 daily doses of linezolid have been taken
- (iii) clofazimine dose reduction below 100mg or cessation would not count as a missed dose once 42 daily doses have been taken
- (iv) ethambutol dose reduction or cessation will not count as a missed dose

Treatment interruption is defined as a period of consecutive missed doses.
Treatment default is defined as treatment interruption (all drugs) lasting more than 8 weeks.

Treatment with the boosted regimen should be extended beyond week 8 to make up for missed doses until the required number of daily doses of the regimen have been taken. In cases where individual drugs have been missed or interrupted, this may mean that companion drugs are required to be given for more than 56 doses to complete the regimen. However, for regimens containing linezolid that need to continue after week 8, linezolid should be stopped as soon as the required number of daily doses (56) have been taken. Linezolid may be stopped after 42 daily doses have been taken, at clinician discretion if there are compelling clinical reasons to do so (principally toxicity).

The boosted treatment regimen should not continue beyond 12 weeks from randomisation.

If patients have a treatment interruption lasting more than 14 consecutive days, or if it is considered unlikely that the required number of daily doses can be completed within 12 weeks from randomisation then patients will be switched to standard treatment to complete a 6-month treatment course.

Patients who default treatment will restart treatment using the standard treatment regimen.

Following treatment interruption or default, additional measures will be put in place to encourage adherence to the treatment regimen.

6.8.2 MODIFICATION FOR PERSISTENT CLINICAL DISEASE

Treatment with the boosted treatment regimen may be extended to a maximum of 12 weeks (84 daily doses) if the following conditions are met at week 8 (or after completion of 56 daily doses if treatment has been extended for missed doses, see Section 6.8.1; or at week 10 if treatment is extended until that time):

(a) Persistent symptoms consistent with ongoing active tuberculosis without alternative, more likely explanation for those symptoms in the opinion of the managing clinician AND

(b) Smear result is positive

For regimens containing linezolid that need to continue after week 8, linezolid should be stopped as soon as the required number of daily doses (56) have been taken. Linezolid may be stopped at clinician discretion after 42 daily doses have been taken if there are clinical reasons to do so (principally toxicity). Clofazimine may also be stopped at clinical discretion after 42 daily doses have been taken if there are clinical reasons to do so (in particular, patient concern about pigmentation).

The boosted regimen should not continue beyond 12 weeks from randomisation.

If the patient has persistent clinical disease at week 12 (as defined above) then treatment may be further extended by switching to the standard treatment regimen.

6.8.3 MODIFICATION FOR OTHER REASONS

In addition to missed doses, treatment interruption, treatment default and requirement for treatment beyond week 12, patients taking one of the boosted regimens may also need to modify treatment in other situations including:

1. Severe and intolerable adverse events not remediable by dose or schedule modification
2. Inability to tolerate the dose schedule or pill burden or other side effects, not remediable by dose or schedule modification
3. Persistent inability to comply with treatment regimen or study protocol safety monitoring requirements
4. Pregnancy
5. Decision by the patient (for reasons other than the above) to discontinue the boosted regimen
6. Decision by the patient to withdraw from trial follow-up (even if they wanted to continue the boosted regimen, they will need to be switched to standard-of-care)
7. Decision by the treating clinician that switch would be in the patient’s best interests (e.g. for TB disease progression)

8. A decision from the Trial Steering Committee (TSC) to stop treatment with a boosted regimen which the patient is currently receiving (usually following IDMC review, see Section 10.4.5)

In these cases, patients will need to switch treatment to the standard treatment regimen.

Where possible, the treating clinician or site PI should discuss with the Chief Investigator prior to switching patients on a boosted regimen to the standard treatment. All cases switching from a boosted regimen to the standard of care regimen during the initial course of treatment will be notified to the trial coordinating centre with reason(s) indicated.

6.8.4 Management of patients switching from a boosted regimen to the standard treatment regimen

The standard treatment regimen as described in Section 6.2.1 will be used. Alternative drugs may be substituted for the drugs in the standard regimen if toxicity, drug resistance or other concerns suggest that the standard regimen is unsuitable in the opinion of the treating doctor.

The standard treatment regimen will be given for 24 weeks, but the duration of the intensive or continuation phase may be modified as follows, at the discretion of the treating clinician:

For patients taking a boosted regimen containing rifampicin (or rifapentine), isoniazid and pyrazinamide who have not stopped or decreased the dose of these drugs below standard treatment doses for a period of more than 14 consecutive days, the duration of the intensive phase may be shortened by the number of doses of these drugs taken prior to treatment switch For patients who have stopped or decreased the doses of these drugs below standard doses for more than 14 consecutive days, the full duration of the intensive and continuation phase should be given.

For patients taking a boosted regimen that does not contain rifampicin or rifapentine (i.e. arm E) who have not stopped or decreased the dose of any of the drugs in the regimen below the prescribed dose for more than 14 consecutive days, the intensive phase of the standard regimen should be given in full, but the duration of the continuation phase may be shortened by the number of doses of the full boosted regimen that were completed prior to the treatment switch.
Further prolongation of the period of standard treatment may be done at the discretion of the treating clinician if alternative (non-standard) drugs are used, or if there are substantive periods of treatment interruption or poor adherence during the period of standard treatment (management will follow the approach for interruptions to the standard regimen as described in Section 6.9 below).

NB: Switch to the standard regimen does not require or constitute trial withdrawal. Such a switch forms part of the management strategy. Patients should not be withdrawn from the trial if the clinician decides a change in treatment is required. If patients choose to discontinue the boosted regimen they are free to do. Although they are not required to give a reason for this, a reasonable effort should be made to establish this reason while fully respecting the patient's rights. Patients deciding to discontinue the boosted regimen should be encouraged to continue treatment with the standard regimen and to remain on trial follow up (see Section 7.4.3).

6.9 MODIFICATION OF INITIAL TREATMENT IN THE STANDARD MANAGEMENT STRATEGY

This will follow the principles outlined for the boosted regimens above. Details will be provided in the Clinical Management Guide. The principles are outlined below.

A missed dose of the treatment regimen is defined as taking less than 50% of the prescribed dose of one or more of the component drugs in a regimen on a particular day.

Treatment interruption is defined as a period of consecutive missed doses.
Treatment default is defined as treatment interruption (all drugs) lasting more than 8 weeks.

Missed doses will be managed by extending the duration of the intensive phase and/or continuation phase until the required number of doses have been taken.

If patients have a treatment interruption lasting more than 14 consecutive days in the intensive phase, treatment should re-start from the beginning. If patients have a treatment interruption lasting more than 14 consecutive days (but less than 8 weeks) in the continuation phase, then treatment should be extended to complete the required number of daily doses.

If patients default treatment in either intensive or continuation phase, treatment should start again from the beginning of the intensive phase.

6.10 MONITORING AND DIAGNOSIS OF RELAPSE

The TRUNCATE-TB monitoring approach is summarised in the Patient Management Strategy (Figure 1, page 6).

Note: the monitoring, diagnosis and clinical management approach does not differentiate between treatment failure and relapse (or re-infection) because this is difficult to do in real-time and is largely academic as the management will be the same. However, treatment failure and relapse will be differentiated based on culture results as defined in Section 10.2.6) for the purposes of the IDMC monitoring and the final trial analysis.

6.10.1 MONITORING STRATEGY

After completion of the initial treatment period, patients in all arms will be monitored closely for the detection of relapse using a pragmatic approach based around close symptom surveillance and regular sputum smears considered likely to be feasible for programmes that might adopt the TRUNCATE-TB management strategy if it is ultimately shown to be effective (and advantageous). This monitoring forms an integral part of the TRUNCATE-TB management strategy. Although post-treatment monitoring is not normally part of the standard management strategy, we include the same monitoring for this group in the trial for the purposes of research outcome data collection and to avoid ascertainment bias that may confound comparison of outcomes between the arms.

The monitoring strategy for the trial comprises the following:

- Monitoring for symptoms every 4 weeks using a standard checklist of symptoms (Section 7.3.1) done at clinic visits and by telephone assessments between scheduled clinic follow-up visits (see Section 7.1.1)
- Sputum smear at all clinic visits (every 4-12 weeks)
- Sputum TB cultures at all clinic visits (every 4-12 weeks), predominantly for the purposes of research data collection, but the results may prove helpful for diagnosis or clinical management in some cases
- Sputum GeneXpert testing if symptoms increase
- CXR if symptoms increase but smear negative; or if no symptoms but smear becomes positive when previously negative

6.10.2 Criteria for diagnosing relapse

Patients will be considered to have relapsed if at least 2 out of criteria A or B or C are satisfied, or if criterion D is satisfied. The criteria should be satisfied simultaneously and be based on recent imaging and microbiological investigations.

A. Clinical Disease Progression
New, recurrent or increased severity of one or more standard TB-related symptoms (cough, one or more episodes of haemoptysis, fever, pleuritic chest pain, weight loss, night sweats) or physical signs compared with the end of the last course of TB treatment (or period after the end of treatment if further improvement occurred subsequently) without alternative explanation(s) considered more likely in the opinion of the managing clinician.

B. CXR Progression
Presence of abnormalities that are compatible with active TB disease (cavitation, infiltrates, consolidation) with clear evidence of progression compared with CXR at end of the last course of treatment (or period after the end of treatment if further improvement occurred subsequently) without alternative explanation(s) considered more likely in the opinion of the managing clinician.

C. Microbiological persistence / progression
Sputum sample taken at or after the end of treatment is:
Smear positive OR
GeneXpert positive (if >24w after end of treatment; or if end of treatment test was negative) OR
Culture positive

D. Confirmed positive sputum culture
Sputum culture positive on two consecutive samples taken on separate days with at least one taken ≥ 4 weeks after end of treatment.

Extra-pulmonary TB disease activity
The above criteria apply to relapse from pulmonary TB. The definition of relapse may also be met if the patient fulfils equivalent criteria that indicate disease relapse at another site i.e. non-pulmonary symptoms or signs, evidence of abnormalities on another imaging test that are compatible with active TB disease with evidence of progression from an earlier comparable imaging test (if no previous test available for determining progression, the abnormalities should be of sufficient severity to explain the symptoms) and microbiological evidence (culture evidence, preferred). Histological evidence considered to show characteristic changes of TB would also be acceptable under criterion C. However, the evidence from A, B and C (or the two cultures for D) should relate to the same disease site.

6.10.3 Clinical management of relapse

For clinical management purposes and prior to commencing a course of re-treatment, the criteria in Section 6.10.2 above should be met. In cases where the relapse diagnosis is based on evidence of disease at a non-pulmonary site, or where the criteria are not met fully, the clinician should discuss with the Chief investigator. Where the criteria are met, treatment should be restarted promptly (within 1 week wherever possible). However, the managing clinician may choose to delay treatment whilst awaiting further clinical or laboratory confirmation of TB relapse or alternative diagnosis if the patient is clinically stable and there are reasons to believe that an alternative explanation is more likely than TB relapse.

Irrespective of the diagnostic criteria met, prior to re-starting treatment the following should be done:

- Additional sputum samples (or other sample, if extra-pulmonary disease suspected) should be collected to ensure that there are at least two sputum (or other) culture samples that are positive or that have results awaited in the period prior to restarting therapy.
- A GeneXpert test should be performed, if not already done as part of the relapse evaluation (the aim here is to check for rifampicin susceptibility)
- DST should be requested on the last 2 positive cultures taken at or prior to the time of relapse.

6.11 RE-TREATMENT REGIMENS

The retreatment regimens are described below. Other aspects of clinical management will follow those described for the initial treatment (Sections 6.4 and 6.5).

6.11.1 EMPIRICAL RE-TREATMENT REGIMEN FOR THE STANDARD MANAGEMENT STRATEGY

Patients randomised to Arm A (standard TB treatment):

Treatment will be with an empirical 24 to 32-week re-treatment regimen based on national treatment guidelines, modified according to drug susceptibility testing (see 6.11.3 below) and drug tolerability.

Some national treatment guidelines recommend empirical treatment with the WHO standard (Category II) 8-month re-treatment regimen.[3] This consists of 3 months of streptomycin (first 2 months only), isoniazid, rifampicin, ethambutol and pyrazinamide administered daily , followed by 5 months of rifampicin, isoniazid, and ethambutol administered daily, as shown in the table below.

Table 7: Drug doses in 32-week (8-month) empirical re-treatment regimen

DRUG	<40KG	40KG- 54KG	55KG - 70KG	>71KG	FREQUENCY
**2 MONTHS TREATMENT WITH**					
Rifampicin<br>Isoniazid<br>Pyrazinamide<br>Ethambutol	Dosing as in Section 6.2.1				Once daily
Streptomycin	15mg/kg (maximum dose 1000mg)				
**FOLLOWED BY 1 MONTH OF**					
Rifampicin<br>Isoniazid<br>Pyrazinamide<br>Ethambutol	Dosing as in Section 6.2.1				Once daily
**FOLLOWED BY 5 MONTHS OF**					
Rifampicin	Dosing as in Section 6.2.1				Once daily
Isoniazid	Dosing as in Section 6.2.1				
Ethambutol	15mg/kg (rounded to nearest 100mg, maximum 1600mg)				

6.11.2 EMPIRICAL RE-TREATMENT FOR THE TRUNCATE-TB MANAGEMENT STRATEGY

The empirical re-treatment regimen will depend on the initial treatment regimen.

For patients whose initial treatment consisted only of treatment with a boosted regimen, empirical re-treatment will be with the standard 6-month 4-drugs regimen as in Arm A (see Section 6.2.1), modified according to drug susceptibility testing (see 6.11.3 below) and drug tolerability.

For patients who switched from a boosted regimen to the standard treatment regimen during their initial treatment, the empirical retreatment regimen will be a 24 to 32-week re-treatment regimen based on national treatment guidelines (as above in Section 6.11.1), modified according to drug susceptibility testing (see 6.11.3 below) and drug tolerability.

6.11.3 DRUG SUSCEPTIBILITY TESTING AND MODIFICATION OF THE EMPIRICAL REGIMENS

It is anticipated that all patients who stop therapy in this trial will have fully susceptible TB at the time of retreatment. However, all patients with relapse will have DST done prior to or at the time of starting a re-treatment regimen: rapid molecular DST results (GeneXpert and others as available), conventional DST (standard first- and second-line drugs by MGIT) and supplemented by identification of mutations on whole genome sequencing (WGS) (where available). The results of cultures done on baseline or any within-study tests will also be taken into consideration in selecting the best re-treatment regimen. If resistance is observed, the empirical regimens will be modified to take this into account.[3] If rapid DST indicates rifampicin resistance, other DST will be accelerated as far as possible and an interim regimen selected by the treating clinician based on local MDR-TB treatment guidelines, which can be modified as appropriate once the full susceptibility results are known.

If drug resistance is detected on any one of the assays (rapid molecular testing, culture or WGS), this will be notified by the sites immediately to the central coordinating team (direct to the Chief Investigator or project leader) and the re-treatment regimen will be discussed with the managing clinician.

6.11.4 STOPPING THE RETREATMENT REGIMEN

In the event that treatment has been started empirically for relapse (in the absence of culture results) and the results of subsequent investigations indicate that the event was unlikely to be a true relapse (e.g. the cultures are negative and an alternative diagnosis emerges to explain the patient's clinical condition) the treatment may be ceased at the discretion of the managing clinician.

7 ASSESSMENTS & FOLLOW-UP

The schedule of visits and assessments is summarised in the Trial Schedule (page 9).

This chapter describes the timing of study visits and the assessments to be performed at study visits. Visits are categorised as standard scheduled visits (those shown in the Trial Schedule, page 9), re-treatment scheduled visits (additional scheduled visits in patients who require re-treatment of TB), and unscheduled visits (additional visits required for evaluation of adverse events or TB disease progression). Procedures to ensure adherence to visits and approaches to handle early withdrawal from follow-up are also described.

7.1 VISIT TIMING

7.1.1 STANDARD SCHEDULED VISITS

Patients will be required to attend protocol-mandated follow-up visits at weeks 1, 2, 4, 6, 8, 10, 12, 16, 20, 24, 36, 48, 60, 72, 84 and 96.

In addition to the scheduled clinic visits, brief telephone assessments will be conducted at the following time points (timed approximately every 4 weeks, between study visits): weeks 30, 40, 44, 52, 56, 64, 68, 76, 80, 88, 92 and at the close of the trial. The telephone assessment will be omitted if it coincides with a visit in person.

Windows around visits up to and including week 4 will be ±3 days; for week 6 to week 8 ±5 days; week 10 to week 24 ±7 days; for week 30 to week 84 ± 14 days; for week 96 ± 28 days.

7.1.2 RE-TREATMENT SCHEDULED VISITS

If a patient is being re-treated for relapse or following a long period of treatment interruption (Section 6.8.1) a revised schedule of study visits will be required to ensure adequate follow-up during and after treatment.

Re-treatment scheduled visits will be required at 2, 4, 8, 12, and 24 and 48 weeks after the start of the re-treatment episode (with the same windows as for the corresponding weeks for standard protocol visits listed above). The standard scheduled visits and re-treatment scheduled visits will be combined and the timing adjusted in order to minimise the additional visit burden for patients (where possible within the respective visit windows, and with priority given to most closely approximate the target date for the re-treatment scheduled visits).

In some cases of retreatment (e.g. for relapse that occurs after week 48 of the trial) this will mean that one or more of the follow-up visits (including the final trial visit-see Section 7.1.4) will occur after week 96 of the trial.

At the point of initiating re-treatment, the trial visit schedule (page 9) will be revised to present the standard and re-treatment scheduled visits together in a single table identifying all visits by the time from randomisation (i.e. in the same format as the trial assessment schedule, page 9).

7.1.3 END OF TREATMENT VISITS

End of treatment visits will be performed at the point when treatment is stopped at the end of the first and any subsequent courses of re-treatment. This refers to the time when treatment is considered

completed and is stopped with the agreement of the clinician (i.e. following extensions of treatment duration, for example for missed doses or switches of treatment for toxicity, Sections 6.8.1 and 6.8.3).

The visit should be done on the same day as treatment cessation (window ± 5 days from the last dose of study medication).

Where possible this visit will coincide with the time of a scheduled treatment visit (with priority in timing given to the target date for the end-of-treatment visit), but where this is not possible an additional visit will be required.

7.1.4 FINAL TRIAL VISIT

The final trial visit will occur at week 96 (or later if additional follow-up is required to complete 48 weeks' observation after retreatment as described in Section 7.1.2). At the final trial visit the patient will be informed that they do not need to come for further trial visits. They will be informed that they will be contacted by telephone again once at the close of the trial and that in the meantime they should notify the study team if they experience any symptoms that suggest possible TB recurrence.

In the event that symptoms reported at a visit after the final trial visit suggest a possible new TB recurrence, patients will be asked to return for an unscheduled visit (see 7.1.6 below).

7.1.5 CLOSE OF TRIAL VISIT

In the month prior to trial closure (approximately 48 weeks from the time when the last patient who required re-treatment prior to week 96 commenced the retreatment) all patients will be contacted by telephone for a close of trial visit.

If symptoms reported at the close of trial visit suggest a possible new TB recurrence, patients will be asked to return for an unscheduled visit (see 7.1.6 below).

7.1.6 UNSCHEDULED VISITS

Patients will be encouraged to report to the study team by telephone or in person if they experience any new or progressive symptoms. If the patient reports by telephone, the information will be documented as for a scheduled telephone assessment and an in-person visit encouraged where necessary.

Unscheduled visits can be performed between the scheduled visits if required for reasons such as evaluation of possible adverse events, or investigation of possible TB relapse. The timing of such visits will depend on the patient and the clinician. Where such a visit falls within the window of a scheduled visit, the visit may replace the scheduled visit (and be recorded as such), provided that all the tests appropriate for the scheduled visit are performed.

Clinic attendances for routine care where the patient is not evaluated by a member of the trial team (e.g. patient attends to have blood tests or a pre-arranged investigation) or attends for reasons that are unrelated to the trial or trial medication need not be recorded as a visit (but any relevant information will be recorded at the following scheduled or unscheduled visit).

7.2 VISIT ASSESSMENTS

Tests listed in this section (sputum smear and culture) and other tests, are not required to be done on the same day as the study visit. They may be performed, for convenience, at any time during the specific visit window (Section 7.1.1). Results from tests performed for routine clinical management

may also be used if they fall within the visit window, are from the same laboratory or facility used in the trial, and the results are available for review by the study team.

7.2.1 ASSESSMENTS AT ALL SCHEDULED VISITS

The following will be performed at all scheduled visits (i.e. all standard scheduled visits and re-treatment scheduled visits):

- Review of symptoms
- Physical examination (vital signs and targeted examination)
- Review of concomitant medication
- Review of TB treatment adherence (where relevant)
- Assessment of healthcare utilisation
- EQ-5D and time off work/education
- Sputum (if available) for smear and liquid culture (MGIT)

Scheduled telephone assessments (Section 7.1.1) will be limited to review of symptoms, concomitant medication and TB treatment adherence (where relevant).

7.2.2 ADDITIONAL ASSESSMENTS AT SPECIFIC SCHEDULED VISITS

The following will be performed at specific scheduled visits in addition to the assessments listed in Section 7.2.1:

Week 1
- 12 lead ECG
- Blood for standard safety monitoring tests

Week 2
- Blood for FBC (only for patients taking linezolid)

Week 4
- Physical examination to include Ishihara and visual acuity test
- 12 lead ECG
- Urine for pregnancy test (if applicable)
- Urine for storage
- Blood for standard safety monitoring tests
- Blood for drug levels (before and 2 hours after taking medication)
- Blood for plasma and host RNA storage

Week 6
- Blood for FBC (only for patients taking linezolid)

Week 8
- Physical examination to include Ishihara and visual acuity test
- 12 lead ECG
- CXR
- Spirometry
- Urine for pregnancy test (if applicable)
- Urine for storage
- Sputum (if available) for GeneXpert test

- Blood for standard safety monitoring tests
- Blood for drug levels (before and 4 hours after taking medication)
- Blood for plasma and host RNA storage

Week 10

- Blood for FBC (only for patients who continue linezolid after week 8)

Week 12

- 12 lead ECG (only for patients who continue a boosted regimen after week 8 and who did not have an end of treatment ECG after week 8).

Week 24

- Urine for storage
- Blood for drug levels (only for patients who took bedaquiline or clofazimine)
- Blood for plasma and host RNA storage

Week 48

- Patient acceptability questionnaire
- Spirometry

Week 96

- MOS-HIV questionnaire
- Patient acceptability questionnaire
- Socioeconomic evaluation
- CXR
- Spirometry
- Sputum (if available) for smear and liquid culture – two samples should be taken on separate days (within the week 96 visit window, and preferably within the same week)

7.2.3 Assessments at retreatment scheduled visits

Assessments at re-treatment visits will include those listed in Sections 7.2.1 and 7.2.2 for the appropriate scheduled visit, plus the following tests if they are not already included at the scheduled visit:

4 weeks after start of re-treatment

- Blood for standard safety monitoring tests

48 weeks after start of re-treatment (only if this falls after the week 96 scheduled visit):

- MOS-HIV questionnaire
- Patient acceptability questionnaire
- Socioeconomic evaluation
- CXR
- Spirometry
- Sputum (if available) for smear and liquid culture – two samples should be taken on separate days (within the visit window, and preferably within the same the same week)

7.2.4 ASSESSMENTS AT END OF TREATMENT VISITS

Assessments at the end of treatment visits will include those listed in Sections 7.2.1 and 7.2.2 for the appropriate scheduled visits, plus the following tests if they are not already included at the corresponding scheduled or unscheduled visit:

- 12 lead ECG (only for patients who stop boosted treatment between week 8 and 12; need not be repeated if done in the previous 7 days and the result was normal including a QTc <450 ms)
- CXR (need not be repeated if done within the previous 7 days and no changes in clinical condition)
- Urine for pregnancy test (if applicable)
- Urine for storage
- Sputum (if available) for smear and liquid culture (need not be repeated if done within previous 7 days)
- Sputum (if available) for GeneXpert test (need not be repeated if done within the previous 7 days)
- Blood for standard safety monitoring tests
- Blood for plasma and host RNA storage

7.2.5 ASSESSMENTS AT THE FINAL TRIAL VISIT

At the final trial visit assessments will be as listed for week 96.

7.2.6 ASSESSMENTS AT THE CLOSE OF TRIAL VISIT

At the close of trial telephone visit, the assessment will be limited to confirming that the patient is alive and assessing whether has been a relapse of TB after trial follow-up ended (defined as the patient being prescribed TB treatment). If the patient cannot be contacted directly, the designated contacts will be approached for information on whether the patient is known to be alive. If the patient reports possible TB relapse and is seen in person for a clinic visit then assessments will be performed as for an unscheduled visit (see above).

7.2.7 ASSESSMENTS AT UNSCHEDULED VISITS

At these visits the assessments listed for all scheduled visits (Section 7.2.1) will be performed, although the sputum sample may be omitted if not relevant to the purpose of the visit. Where a decision is made to combine the visit with a scheduled visit all the assessments required for that visit should be performed. Additional assessments or investigations may be performed at the discretion of the treating clinician, as relevant for the purpose of the visit.

For visits after the final trial visit assessments will be limited to review of symptoms and reports of presumed or confirmed TB.

7.2.8 ADDITIONAL ASSESSMENTS PERFORMED WHEN SUSPECTED RELAPSE (AT SCHEDULED OR UNSCHEDULED VISITS)

If an increase in symptoms, or reversion of smear from negative to positive after the end of treatment, or presence of physical signs indicate the possibility of TB relapse the following additional tests (if not already mandated at the visit) should be done at the first visit where relapse is suspected:

- Medical history including household contact history (household contact history only required if decision is made to re-start treatment)

- CXR to look for progression of TB radiological findings from an earlier CXR or to look for alternative causes for the symptoms

- Sputum for smear and liquid culture (and DST and WGS if culture positive) Collect additional sample(s) if required, to ensure there are at least two with positive cultures or that have results awaited in the period prior to restarting therapy.

- GeneXpert test. A positive result is of value in detecting the presence of new rifampicin resistance; a negative result may also be reassuring. A positive result obtained after at least 24 weeks from the time of stopping treatment may be taken as confirmation of relapse for clinical management purposes, regardless of the end of treatment result (Section 7.3.10). If the end of treatment result was negative, subsequent positive results will also be regarded as indicative of relapse

- Urine for pregnancy test (if applicable; if the decision is made to re-start TB treatment)

- Urine for storage

- Blood for plasma and host RNA storage

- Blood tests or other investigations to rule out alternative diagnoses (optional, at the discretion of the managing clinician)

7.3 ASSESSMENT PROCEDURES

This Section describes the procedures used for performing the assessments listed in the Trial Schedule and in Sections 4.3, 5.1 and 7.2. The main safety assessments are symptom evaluation, physical examination, ECG, pregnancy testing, sputum DST, and blood tests for safety monitoring with additional investigations as needed. Procedures for the classification and reporting of adverse events are described in Chapter 8 and for analysis and reporting of safety outcomes in Chapter 10. The main efficacy assessments are symptom evaluation, physical examination and sputum liquid culture. Procedures for analysis and reporting of efficacy outcomes are described in Chapter 10.

7.3.1 CLINICAL EVALUATION

Current and past medical history:
At the screening and baseline visits, patients will be asked about the history of TB and other co-morbid diseases relevant for the assessment of eligibility for the trial, with information supplemented by review of the relevant medical records.

Demographics:
At the screening visit relevant demographic information will be recorded (age, gender, ethnicity, country of birth) and patients will be asked about disease risk factors such as smoking and alcohol consumption. At the baseline visit and when there is a suspected relapse, a household contact history will be taken (number of people living in the same household and their extent of daily contact).

Symptoms:
At screening, baseline and every study visit patients will be asked about the presence or absence and duration of common TB symptoms (cough, haemoptysis, fever, pleuritic chest pain, weight loss, night sweats), disturbed vision, and symptoms suggestive of peripheral neuropathy or hepatitis. Patients will also be asked to report any other symptoms.

At screening patients will also be asked about the presence or absence of specific symptoms that might suggest an extra-pulmonary site TB disease.

At baseline, end of treatment, weeks 48, 96 (and the final trial visit, see Section 7.1.4), symptoms of respiratory disability will be assessed using the MRC breathlessness scale (see Appendix 3).

Physical examination:
Patients will be examined at each study visit including, at a minimum, the measurement of vital signs (body temperature by oral or tympanic route, pulse, blood pressure and respiratory rate). Body weight will also be measured at each visit, where possible using the same set of electronic weighing scales throughout trial follow-up. Height will be measured at the baseline visit.

At the screening, baseline and week 8 visits, a comprehensive physical examination (including at a minimum a general inspection, cardiovascular, respiratory and abdominal examination) will be performed. At these and other visits, targeted physical examination will be done as required to evaluate new or worsening symptoms likely related to TB or other causes.

A test for visual acuity and an Ishihara test for colour vision will be performed at screening (Ishihara only), baseline (visual acuity only), week 4, and week 8 for all patients and repeated if a patient experiences visual symptoms while on TB treatment.

Additional investigations and/or specialist referrals may be performed if deemed necessary by the site investigator, either for clinical management purposes or to enable accurate evaluation of a possible adverse drug reaction.

Clinical adverse events will be graded by the site team according to standard criteria of severity (Appendix 10) and will also be evaluated to determine whether they meet the criteria for being considered a serious adverse event (Chapter 8). For serious adverse events, further classification by causality and expectedness will be performed (Sections 8.2.1). All reports of Grade 3 and 4 adverse events and SAEs will be reviewed by the Chief Investigator or medically-qualified delegate to ensure that they meet specified criteria and any discrepancies will be resolved with the site.

7.3.2 MEDICATION AND ADHERENCE ASSESSMENT

Adherence to anti-TB medication will be assessed at each study visit by means of standard questions to the patient and by reviewing the TB treatment or DOT card. Patients will be asked at each visit about any other (non-TB) medication or drugs they are taking and the reason for the medication use.

7.3.3 HEALTH CARE UTILISATION

The trial will measure all the healthcare-related costs of patients in the trial, from the start of TB treatment until the final trial visit. Estimates of costs incurred by the patients (transport and out-of-pocket healthcare expenditures including days of work lost for patient and caregiver) will be obtained by patient self-report. Reported transport costs will be confirmed using local information on distance and cost of transport.

Information on hospitalisations (number, reason, and duration of stay), and data on other healthcare resource utilisation (outpatient visits, medications, and procedures) will be collected by patient interview, supplemented by clinic or hospital records or discharge summaries, where available. Information on contact tracing activity performed during the study as a result of treatment failure or relapse will be collected where available.

7.3.4 QUALITY OF LIFE

Quality of life will be assessed at baseline, and week 96 (and final trial visit, see Section 7.1.4) using a comprehensive assessment tool, the Medical Outcomes Study HIV Health Survey (MOS-HIV) (Appendix 11). This is a 13-item questionnaire based on the SF36 which has been validated for use in patients with HIV infection[75] and used in TB trials.[76] Existing validated translations will be used where possible. Where no existing translation is available, translations of the questionnaire will be made into appropriate local languages and verified using standard forward and back translation procedures. The questionnaire will be administered in the patient's own language by a member of the research team and the language in which the questionnaire was administered will be recorded on the CRF.

Quality of life will also be assessed at each study visit using a very brief tool, the EQ-5D index (Appendix 12). Five domains (mobility, self-care, usual activities, pain/discomfort and anxiety/depression) are included and each item is ranked on a 5-point scale ranging from 'no problems' to 'extreme problems' with lower scores indicating better health status. Existing validated translations will be used where possible. Where no existing translation is available, translations of the questionnaire will be made into appropriate local languages and verified using standard forward and back translation procedures. The questions will be administered in the patient's own language by a member of the research team and the language in which the questionnaires were administered will be recorded on the CRF.

The patient will also be asked at each visit about any time off work/school required by TB illness or the process of treatment (quantified in total hours missed).

Acceptability of the management strategy to the patient will be assessed at week 48, week 96 (and the final trial visit, see Section 7.1.4) with a short series of additional questions about their experience with treatment (Appendix 6).

7.3.5 SOCIOECONOMIC EVALUATION

Socio-economic status of trial participants will be assessed using questions based on the World Bank Living Standards Measurement Survey[77] and the Measure DHS Demographic and Health Survey.[78] Socioeconomic assessment will be conducted at baseline and week 96 (and the final trial visit, see Section 7.1.4). The questions encompass the individuals, and the household they belong to. The questions focus on educational level attained, income proxies (including type of housing, nature of occupation and whether formal or casual employment), household composition (number of adults and children), participation in welfare and insurance schemes and information on asset ownership which can then be aggregated into an index of socioeconomic status.

7.3.6 ECG

A 12-lead ECG will be performed at screening, baseline (before taking randomised treatment), and at weeks 1, 4, 8, end of treatment (only required if patient stops a boosted regimen between weeks 8 and 12) and week 12 (only required if patient switches a boosted regimen to standard treatment between weeks 8 and 12). The time of the ECG and the time of the last dose of study medication will be recorded.

If the QTc reading is above 450ms on any occasion, the ECG should be repeated to confirm this result. At screening, the repeat ECG can be used to determine eligibilty if the result is ≤ 450 ms. In patients with QTc above 500ms, the ECG should be repeated at appropriate intervals (at the discretion of the clinician) until resolution of the QTc prolongation or until changes have stabiliised.

Where possible, the ECG machines will be pre-programmed to display QTc values calculated by Fridericia's formula. The machine-calculated heart rate, QT, and QTc interval will be recorded in the

CRF, together with any clinically-significant ECG abnormalities if present in the opinion of the site doctor. If the machine-calculated QTc is above 450ms and this is using Bazett's formula (or the formula the machine is using is unknown), then the QTc will be calculated manually using Fridericia's formula (Appendix 4).

Interpretation of the ECG tracing must be made by a qualified doctor (minimum 1 full year of licensed clinical practice) whose details must be listed in the study delegation log. QTc prolongation or other ECG abnormalities will be graded by the site team according to standard criteria of severity (Appendix 10). A QTc above 500ms will not be regarded as a serious adverse event unless accompanied by arrhythmias or it results in hospitalisation.

7.3.7 Chest X-ray

A standard posterior to anterior view CXR will be obtained at screening, baseline, week 8, end of treatment, at the time of suspected relapse, week 96 and the final trial visit (if this is not at week 96; see Section 7.1.4). This will be evaluated by the site clinician and the percentage of lung involvement and cavitation classified using a standardised approach.[79] The CXR at any visit need not be repeated if done in the previous 7 days and the film is available for evaluation.

7.3.8 Spirometry

Spirometry to measure FEV1 and FVC will be done at week 8, week 48 and week 96 (and the final trial visit, if this is not at week 96; see Section 7.1.4) using a standardised portable spirometer. If a patient has recently re-started treatment at the time of a scheduled spirometry measurement, the procedure will be deferred until the completion of 8 weeks of treatment. Spirometry may be omitted for clinical or logistical reasons.

7.3.9 Urine

Urine pregnancy test will be performed at baseline, weeks 4, 8 , end of treatment and prior to start of re-treatment for relapse in women of childbearing potential using a standard test procedure at the local site.

Urine (10ml) will be stored at baseline, weeks 4, 8, 24, end of treatment and at first suspicion of any relapse for possible biomarker measurement at end of the trial, as recommended by the CDC/NIH TB biomarker and surrogate endpoint research roadmap.[80]

7.3.10 Sputum analysis

Laboratory methods will be standardised at all the study sites and will be described in detail in the TRUNCATE-TB Laboratory Manual. The general approach is outlined below.

Spot sputum samples will be obtained (where possible) at all protocol-mandated study visits, and at any additional visits following the completion of treatment when relapse is suspected, but has not yet been bacteriologically confirmed. All sputum samples will be obtained using standard approaches for spontaneous expectoration.[3]

Overnight sputum samples may be used for tests where available. Where a patient is unable to produce a sample due to clinical improvement and resolution of symptoms this circumstance will be recorded, but there is no need for further action. Induced sputum is not required by the protocol in any situation. However, it may be performed in cases where the patient is unable to produce sputum spontaneously, induced sputum is a routine procedure at the clinical site, and the treating clinician considers that an induced sputum is essential for clinical management. The results from an induced sputum will be treated for protocol purposes as a sputum obtained by regular methods.

The sputum samples will be processed as described below:

- Smear microscopy

This will be done on sputum samples taken at screening, baseline and on all follow-up time points during the trial, and will be graded using IUATLD grading system (see Appendix 2).

- Liquid Culture

Liquid culture will be performed at baseline and on all sputum samples obtained during trial follow-up. Liquid culture will be standardised to the Becton Dickinson Mycobacterial Growth Indicator Tube system (MGIT). The culture result (positive or negative) and the time to positive culture will be recorded. All isolates obtained during the trial will be stored in case of need for future identification or supplementary resistance testing. The strain type will be identified using standard local laboratory approaches on the first sample and on the first isolate available during an episode of relapse. Additional cultures with resuscitation promoting factors (a potential biomarker for relapse) may be performed on sputum samples collected up to week 8 (only in selected laboratories, where feasible, and on samples where sufficient sputum volume remains after other protocol-mandated tests have been performed).

- GeneXpert

This rapid molecular test will be performed on sputum samples obtained at screening, at week 8 (and end of treatment, if treatment continues after 8 weeks) and on the initial sputum sample obtained during any suspected episode of relapse. The test will be done using the standardised method recommended by the manufacturer, and three results will be recorded: whether the test is positive or negative for TB and, if positive, the cycle threshold and the presence of rifampicin resistance mutations. These results will be used and interpreted in different ways at each time point. At screening the test will be used to confirm the diagnosis of tuberculosis, and to confirm the absence of rifampicin resistance. At the end of treatment the test will be used to document the continued absence of rifampicin resistance (of interest for safety assessment of boosted regimens that include a rifamycin), and the cycle threshold will be recorded for later validation (at the end of the trial) as a possible biomarker of cure, but a positive overall result will not be considered indicative of treatment failure at this time point (as current evidence indicates a considerable lag time between the killing of bacilli and reversion of the GeneXpert result to negative). At subsequent time points, a positive result will be regarded as indicative of relapse if:
- it is taken at more than 24 weeks following the completion of the last course of TB treatment, regardless of the end of treatment result OR
- it is taken at any time point after treatment completion and the end of treatment result was negative (as outlined Figure 1, page 6).

- Drug susceptibility testing (DST)

DST will be performed on:
- all isolates obtained from baseline cultures (if baseline cultures are negative then DST will be performed on the first positive culture obtained, or on the first isolate obtained prior to starting treatment, if available).
- all isolates obtained from cultures at or after 8 weeks on treatment (including retreatment episodes) and every 4 weeks thereafter until cultures become negative.
- the first two isolates cultured during a relapse episode.
- all isolates obtained from positive cultures from the week 96 visits (and final trial visit, see Section 7.1.4)
- additional isolates as required to verify findings obtained from the isolates listed above or to elucidate evolution of drug susceptibility in longitudinal samples

DST will be performed primarily using MGIT methodology, supplemented by GeneXpert, other molecular/typing methods and other rapid testing methods where appropriate/available to optimise the speed and accuracy by which drug resistance is detected. At a minimum, baseline isolates will be tested for susceptibility to standard first-line drugs. At a minimum, isolates obtained at week 8, isolates obtained at relapse, and isolates obtained at baseline in patients who subsequently relapse will be tested for susceptibility to standard first-line drugs, fluoroquinolones and any additional drugs to which the patient has been exposed in their current or past trial regimen and for which reliable testing methods are available. Additional DST may be performed at the discretion of the trial management team to seek (or exclude) evidence of phenotypic resistance patterns that might contribute to understanding trial outcomes. Additional DST may be performed at the discretion of the managing clinician if clinically indicated for regimen modification/optimisation.

- Whole genome sequencing (WGS)

WGS will be performed on all strains from relapse episodes and the corresponding baseline isolate to identify new drug resistance mutations in the relapse isolate and to differentiate relapse from reinfection.[81] Additional WGS may be performed systematically on isolates obtained at baseline or during treatment to examine the relationship between bacterial genome, drug susceptibility and treatment outcomes. If WGS is not available locally, samples will be sent to alternative national or international laboratories where this test is performed (subject to regulatory approvals and granting of export permits, where needed). If WGS is not available locally or nationally and permission for export of samples cannot be obtained, then baseline and relapse strains will be compared using alternative locally or nationally-available typing methods.

7.3.11 Blood tests

- Standard safety tests

Blood will be drawn for standard safety tests (FBC, electrolytes (sodium, potassium, creatinine), LFTs (ALT, alkaline phosphatase, bilirubin)) at screening, baseline, weeks 1, 4, 8 and the end of treatment visit. Additional tests for amylase/lipase, magnesium, calcium and glucose will be performed at screening. Additional FBC will be performed at weeks 2 and 6 (and at week 10 if treatment extended) in patients receiving linezolid (Arms B, D and E). Safety blood tests will be performed in the local laboratory used by the site.

Additional blood tests may be performed if deemed necessary by the site investigator, either for clinical management purposes or to enable accurate evaluation of a possible adverse drug reaction. Any grade 3 or 4 laboratory abnormalities or other results of concern should be followed with further tests to document resolution after the completion of treatment. Any laboratory adverse events will be graded by the study team according to standard criteria of severity (see Appendix 10). The investigators will also assess whether the event meets standard definitions for a serious adverse event (Section 8.1.2).

- HIV test (and CD4 count)

Blood will be taken for HIV antibody testing at screening. If this is positive, and the trial has reached a stage where the site is open to enrolment of HIV patients, the patient still wishes to participate, has access to ART and meets other eligibility criteria, then a CD4 count will be performed to further determine eligibility.

- Drug levels

Blood (5ml) will be obtained from all patients at Day 0 (1 hours post-dose; window 1-2 hours post-dose), week 4 (pre-dose and 2 hours post- dose (if split dose, timed from first drug ingestion; window 1-2 hours post-dose and week 8 (pre-dose and 4 hours post- dose (if split dose, timed from first drug ingestion; window 3-4 hours post-dose) for measurement of levels of all the drugs in the randomised

regimen (plus any additional drugs if switched to standard TB regimen). If the time point for PK sampling is missed, blood should still be taken and the time recorded as this may remain valuable in PK analyses. Samples may be omitted for patient convenience or logistical reasons.

At week 24, patients who were randomised to arms containing clofazimine (Arm C) or bedaquiline (Arm E) will have samples taken for measurement of levels of the respective drug (one sample, no timing requirements). Drug levels will be measured in one or more laboratories using liquid chromatography and mass spectrometry (LC/MS) protocols (Chapter 11).

- Blood for storage

A 10ml EDTA blood sample and 5ml sample in an RNA preservation tube will be drawn at baseline, weeks 4, 8, 24 and end of treatment and at the first suspicion of any relapse episode as recommended by the CDC/NIH TB biomarker and surrogate endpoint research roadmap.[80] These samples may be used for further analyses which may include biomarker measurement at end of the trial (precise tests and methods to be decided at a later stage, subject to approval by the TSC and local ethics committees where required).

7.4 MAINTAINING ADHERENCE TO FOLLOW-UP

7.4.1 MEASURES TO MAINTAIN ADHERENCE TO FOLLOW-UP

Trial visit schedules will be prepared for each patient at randomisation (amended if there is a need for re-treatment, Section 7.1.2) and patients will be encouraged to attend on the exact scheduled days where possible. At each study visit an agreed follow-up date for the subsequent visit will be set according to patient and staff availability, as close as possible to the scheduled day and within the specified visit window. Each site will put in place reliable systems to remind patients of a forthcoming visit (for example using telephone calls and SMS texts). Sites will also take prompt action to trace and re-call patients who do not attend on the expected and pre-arranged day. If the patient cannot be contacted in person, the site will contact named friends/relatives (consent and contacts obtained at trial recruitment) and conduct home visits where necessary. These systems will be described in a site-specific trial implementation manual that will be developed prior to the recruitment of patients at the site (see also Section 7.4.4).

7.4.2 MANAGING PATIENT TRANSFERS

If a patient moves from the area, every effort should be made for the patient to be seen at another participating trial site if available. A copy of the patient's CRFs should be provided to the new site and the patient will need to sign a new consent form. Once this has been done, the new site will take over responsibility for the patient; until this has been done, responsibility for the patient lies with the original site. In some circumstances, such as a patient moving outside the study area, it may no longer be possible for the patient to be followed-up in the trial clinic. In these cases the approach to maintaining follow-up data collection should be discussed as described in the Section below.

7.4.3 MANAGING REQUESTS FOR WITHDRAWAL FROM FOLLOW-UP

If a patient expresses a desire to withdraw from trial follow-up visits, the treating clinician and site staff should explain to the patient the importance of remaining on trial follow-up in order to monitor long-term safety and to gather important long–term outcome data so that the trial can deliver a meaningful answer. The site team should make every attempt to encourage the patient to continue with trial follow-up visits.

If it is clear that the patient is unwilling or unable to attend trial follow-up visits (such as moving out of the area), then their permission will be sought to be contacted periodically by telephone (at scheduled study visit times, but if this is not acceptable then at mutually agreeable frequency) to check on their wellbeing. The importance of the week 96 visit will be emphasised and permission explicitly sought to be contacted at this time point. Their permission will also be sought to make arrangements for routine clinical outcome data to be collected through other channels (either from the routine service clinic at the site, or a new clinic if they are transferring their care). If the patient accepts one or both of these compromise measures, then follow-up will continue as agreed.

If the patient explicitly states their wish that no there should be no further contact and no further follow-up routine data collected for the trial, then the site should inform the Chief Investigator as soon as possible and a Withdrawal CRF should be completed. Patients withdrawing from trial follow-up whilst taking one of the boosted regimens will need to change to standard therapy and be referred to the routine TB care services. If treatment has recently been completed with a boosted treatment regimen, then patients may also be offered re-treatment with a standard course of treatment if so desired (Section 6.11.2). Patients may change their minds and resume follow-up and/or permit data collection at any time.

7.4.4 Managing loss to follow-up

A patient will be regarded as “lost to follow-up” if they have not been seen in clinic for at least 8 months. During this time, periodic attempts should be made to contact the patient via telephone, email and or post, via designated friends/relatives given as contacts, and by a home visit if necessary. Details of those attempts (e.g. dates of telephone calls, registered letters, email correspondence etc.) should be recorded in the source documents. Subsequently, if the patient attends clinic or is contactable by telephone and provided follow-up is rescheduled and a CRF is completed, the “lost to follow-up” status will be reversed.

The measures taken to ensure adherence to clinic visits and the monthly telephone assessments from week 30 will maintain ongoing close contact with patients and this will contribute to minimising loss to follow-up. For patients designated as lost-to-follow-up, a special effort will be made to contact them for the week 96 visit given the particular importance of this visit for the analysis of the primary outcome. At this visit, if contact with the patient has not been established, then a check with the national TB registry and death registry will be made, in countries where this resource is available. Consent will be obtained for this when the patient enters the trial.

8 SAFETY REPORTING

The principles of ICH GCP require that both investigators and sponsors follow specific procedures to record, assess and report adverse events in clinical trials. The procedures required for this trial are described in this chapter. Section 8.1 gives relevant definitions, Section 8.2 gives details of the investigator responsibilities and Section 8.3 provides information on the sponsor responsibilities.

8.1 DEFINITIONS

The definitions for safety reporting in this trial are based on the principles of ICH GCP.

8.1.1 DEFINITION OF ADVERSE EVENT

Any untoward medical occurrence in a patient or clinical trial subject to whom a medicinal product has been administered (including occurrences that are not necessarily caused by or related to that product).

Clarifications:
A medicinal product is defined as any medication administered to the participant.
Adverse Events include:

- An increase in the severity of a pre-existing illness or symptom present at baseline (defined as an increase in the grade, see Appendix 10)
- An increase in frequency or severity of a pre-existing episodic illness or symptom (increase in severity defined as an increase in the grade, see Appendix 10)
- A condition (even though it may have been present prior to the start of the trial) that is first detected after trial drug administration

Adverse Events do not include:

- Undergoing a medical or surgical procedure (the condition that leads to the procedure is the adverse event)
- Hospitalisation where no untoward or unintended response has occurred, e.g. elective cosmetic surgery, social admissions
- Overdose of medication without signs or symptoms
- Pregnancy (but pregnancy must be reported, see Section 8.2.3 below)

8.1.2 DEFINITION OF SERIOUS ADVERSE EVENT

Any adverse event (irrespective of relationship to medicinal product) that:

- Results in death
- Is life-threatening
- Requires hospitalisation or prolongation of existing hospitalisation
- Results in persistent or significant disability or incapacity
- Is a congenital anomaly or birth defect
- Is another important medical condition

Clarifications:

Adverse event is defined as above (Section 8.1.1).

Life-threatening refers to an event in which the patient is at risk of death at the time of the event; it does not refer to an event that hypothetically might cause death if it were more severe, for example, a silent myocardial infarction.

Hospitalisation is defined as an inpatient admission, regardless of length of stay, even if the hospitalisation is a precautionary measure for continued observation. Hospitalisation for a pre-existing condition that has not worsened or hospitalisation for an elective procedure are not SAEs.

Important medical condition is not defined, but instead relies on medical judgement. This should include events that are not immediately life-threatening or do not result in death or hospitalisation but that carry a real (not hypothetical) risk of one of the outcomes listed in the definition above.

8.1.3 Definition of Adverse Reaction

An adverse event that is considered related to an investigational medicinal product.

Clarifications:
Adverse event is defined as above (Section 8.1.1).

Investigational medicinal product (IMP) is defined as the medicinal products and the comparators used in the study (i.e. those medications listed as components of the treatment regimens in Section 2.5; does not include concomitant medications used for supportive or background care).

Related is defined as possibly, probably or definitely related, following the guidance in the following table.

Table 8: Assessment of relationship between investigational medicinal product and the adverse event

Relationship	Description
Unrelated	There is no evidence of any causal relationship
Unlikely	There is little evidence to suggest that there is a causal relationship (for example, the event did not occur within a reasonable time after administration of the trial medication). There is another reasonable explanation for the event (for example, the patient's clinical condition, other concomitant treatment).
Possible	There is some evidence to suggest a causal relationship (for example, because the event occurs within a reasonable time after administration of the IMP). However, the influence of other factors may have contributed to the event (for example, the patient's clinical condition, other concomitant treatments).
Probable	There is evidence to suggest a causal relationship and the influence of other factors is unlikely.
Definitely	There is clear evidence to suggest a causal relationship and other possible contributing factors can be ruled out.

8.1.4 DEFINITION OF AN UNEXPECTED ADVERSE REACTION

An adverse reaction, the nature or severity or frequency of which is not consistent with the information about the investigational medicinal product set out in the current Summary of Product Characteristics (SPC) or Investigator Brochure (IB) for that product.

Clarifications:
Adverse reaction is defined in 8.1.3 above.
IMP is defined in 8.1.3 above.

8.1.5 DEFINITION OF A SUSPECTED UNEXPECTED SERIOUS ADVERSE REACTION

An unexpected adverse reaction that meets the definition for serious.

Clarifications:
Unexpected adverse reaction is defined in 8.1.4 above.
Seriousness is defined in 8.1.2 above.

8.2 INVESTIGATOR RESPONSIBILITIES

All adverse events should be assessed by the site investigator as described below and recorded in the patient's medical notes and the trial CRF (and SAE Form if appropriate). When required, the event should be reported to the trial co-ordinating centre and appropriate agencies within specified timelines, as described in Section 8.2.4 below. Pregnancy (although not an adverse event) also has safety concerns and has specific recording and reporting requirements as described below.

8.2.1 ASSESSMENT OF ADVERSE EVENTS

All events will be evaluated by the investigator as follows:

Assess whether it's an adverse event: assess whether the event meets the definition of an adverse event as in Section 8.1.1 above.

Assess severity: assign a severity grade using the DAIDS toxicity grading scale as described in Section 7.3 and Appendix 10.

Assess seriousness: assess whether the event meets the definition of SAE, as in Section 8.1.2 above.

Assess relatedness: for events that meet the definition of SAE, proceed to assess the relationship between the event and the IMP as in Section 8.1.3 above.

Assess expectedness: for events that meet the definition of SAE and that are considered related to the IMP (Section 8.1.4 above), proceed to assess whether the event is unexpected.

8.2.2 RECORDING OF ADVERSE EVENTS

From screening to the final trial visit (at week 96, or the end of scheduled follow up, if extended for re-treatment), all adverse events will be recorded. After the final trial visit (and up to the close of trial visit), only deaths and recurrent TB will be recorded.

Events will be recorded in the patient's medical notes and on standard CRFs and a severity grade assigned. For events of Grade 3 and 4 severity, a short justification will be provided for the classification based on key words in the toxicity criteria (Appendix 10).

Events that are assessed to be SAEs will also be recorded on a SAE Form (completed by the investigator, see below). Patients with a SAE must be followed closely until clinical recovery is complete and laboratory results have returned to normal or baseline, or until the event has stabilised. Follow-up should continue after completion of protocol treatment if necessary. Follow-up information will be recorded on additional SAE forms. Completion of a SAE form will not be required for events occurring between screening and randomisation or occurring after the final trial visit.

Recording of adverse events is not required for patients who are not eventually randomised.

8.2.3 RECORDING OF PREGNANCY

Although pregnancy does not constitute an adverse event, the investigator should record any pregnancy occurring in a patient or in a partner of a patient in the trial up to the time of the final trial visit. This should be done in the patient's medical notes and on a specific Pregnancy CRF. The pregnancy should be followed up to determine outcome, including spontaneous or voluntary termination, details of the birth, and the presence or absence of any birth defects, congenital abnormalities, or maternal and/or newborn complications. The Pregnancy form should be updated when such information becomes available.

8.2.4 REPORTING TIMELINES AND PROCEDURES

The investigator should report all Serious Adverse Events and pregnancies occurring between randomisation and the final trial visit to the coordinating centre as soon as possible (latest **within 24 hours)** of the investigator becoming aware of the event.

To report an SAE, the SAE Form must be completed by the investigator (the clinician named on the Signature List and Delegation of Responsibilities Log who is responsible for the patient's care). In the absence of the responsible investigator, the form should be completed and signed by a member of the site trial team. The responsible investigator should subsequently check the SAE Form, make changes as appropriate, sign and then re-send the form to the co-ordinating centre as soon as possible. The initial report must be followed by detailed, written reports as appropriate.

The minimum criteria required for reporting an SAE are the trial number, name of investigator reporting, the event, and why it is considered serious.

Extra, annotated information and/or copies of test results may be provided separately. The patient must be identified by trial number and initials only. The patient's name should not be used on any correspondence and should be deleted from any test results.

Follow-up SAE Forms should be completed and sent to the coordinating centre as further information becomes available. When the event has resolved a final follow-up report should be sent indicating that the event has resolved.

A similar procedure is followed for initial and follow-up reporting of pregnancies.

The investigator is also responsible for reporting of SAEs or pregnancies to their Ethics committee and / or regulatory agency as required by local regulations.

Data on all other adverse events that do not require specific reporting within 24 hours will be provided to the coordinating centre through routine CRF completion processes and within agreed timelines.

8.3 SPONSOR RESPONSIBILITIES

The Chief Investigator (or a medically-qualified delegate) will review all SAE reports received at the co-ordinating centre, as well as all Grade 3 and 4 AEs recorded on the standard CRFs. If there is disagreement on the nature or grading of the event, this will be discussed with the site investigator and the SAE report and CRF record amended as needed. If agreement cannot be reached, the causality assessment given by the local investigator at the site cannot be overruled and both opinions will be provided in any subsequent reports. For the assessment of expectedness, the Chief Investigator (on behalf of the sponsor) has the final say.

The responsibility for safety reporting to the regulatory authorities as required by national regulations will be delegated by the sponsor to the coordinating centre or site by agreement. The coordinating centre will be responsible for reporting to any pharmaceutical companies according to contractual agreements. The coordinating centre will submit Annual Safety Reports in the required format to Competent Authorities (Regulatory Authority and Ethics Committee) and will also keep all investigators informed of any safety issues that arise during the course of the trial.

9 QUALITY ASSURANCE & CONTROL

9.1 RISK ASSESSMENT

The Quality Assurance (QA) and Quality Control (QC) considerations for this trial have been based on a formal trial-specific Risk Assessment, which acknowledges the risks associated with the conduct of the trial and how to address them with QA and QC processes.

QA includes all the planned and systematic actions established to ensure the trial is performed and data generated, documented and/or recorded and reported in compliance with the principles of ICH GCP and applicable regulatory requirements. QC includes the operational techniques and activities done within the QA system to verify that the requirements for quality of the trial-related activities are fulfilled.

9.2 CENTRAL MONITORING AT THE TRIAL COORDINATING CENTRE

Central monitoring measures are described in a trial-specific Monitoring Plan that is based on the trial-specific Risk Assessment.

Staff at the coordinating centre will review any CRFs received for errors and missing data points. Data stored on the central database will be checked for missing or unusual values (range checks) and checked for consistency within participants over time. Sites will be contacted with data queries according to procedures in the Monitoring Plan.

9.3 MONITORING AT THE CLINICAL SITES

The frequency, type and intensity for routine monitoring and the requirements for triggered monitoring will be detailed in the Monitoring Plan. This plan will also detail the procedures for review and sign-off.

Participating investigators will be obliged by contract to allow trial-related monitoring, including audits, ethics committee review and regulatory inspections by providing direct access to source data and documents as required. The consent form will seek explicit patient consent for this.

9.4 CONFIDENTIALITY

Patients' confidentiality will be maintained throughout the trial. Data submitted to the coordinating centre and samples sent to central testing facilities will be identified only by trial number and patient initials.

10 STATISTICAL CONSIDERATIONS

10.1 METHOD OF RANDOMISATION

Patients will be randomised using a web-based system with randomisation lists based on random permuted blocks stratified by site and potential relapse risk (Lower risk: screening sputum smear negative and screening CXR cavities ≤ 4 cm and HIV negative; Intermediate risk: screening sputum smear positive and screening CXR cavities ≤ 4 cm and HIV negative; Higher risk: screening sputum smear grade 3+ or screening CXR cavities > 4 cm or HIV positive). Enrolment of patients with potential higher relapse risk will only be done from stage 3 (subject to the conditions described in sections 4.2.1). Patients will be randomised in equal proportion to all the treatment arms open for randomisation.

10.2 OUTCOME MEASURES

The primary endpoint for the trial is unsatisfactory clinical outcome at week 96. This is a composite endpoint chosen to represent a pragmatic view of the outcome of treatment from a patient and programme perspective. A patient with an unsatisfactory clinical outcome can be viewed holistically as one for whom ongoing programme resources are required to manage TB drug dispensing and monitoring, who has symptomatic active TB disease, or who has died from their disease. Based on the information available at week 96, or other considerations if the patient is not seen at week 96, the primary outcome for each patient will be classified as unsatisfactory, satisfactory or un-assessable.

If non-inferiority is demonstrated on this primary outcome, then the various advantages and disadvantages of the TRUNCATE-TB management strategy can be explored in analyses of secondary outcomes. The important secondary outcomes are those that reflect the possible advantages and disadvantages of the TRUNCATE-TB management strategy from the **patient perspective** (including acceptability of the strategy, total days on treatment, time off work/study due to TB disease or its treatment, quality of life, health status, respiratory disability, clinical grade 3 or 4 adverse events, all serious adverse events and death; see Section 10.2.3) and from the **programme perspective** (including treatment adherence, treatment default, new drug resistance and community transmission risk; see Section 10.2.4).

Analysis of costs (from patient and programme perspective) and cost-effectiveness are also critical for evaluation of the TRUNCATE-TB management strategy (health economics, Section 10.5.4).

Outcomes for the evaluation of the individual drugs and the boosted regimens (Section 10.2.6) include pharmacokinetics, microbiological ouctomes (time to stable culture conversion, treatment failure, relapse, STR) and toxicty outcomes (grade 3/4 clinical and laboratory adverse events during the initial treatment period, QTc prolongation and serious adverse events considered related to study medication).

10.2.1 PRIMARY OUTCOME DEFINITION FOR PATIENTS WHO ATTEND DURING THE WEEK 96 ANALYSIS WINDOW OR ARE KNOWN TO HAVE DIED

Patients who have an ongoing requirement for TB treatment, or who have ongoing TB disease activity at week 96 or who have died before week 96 (unless definitely unrelated to TB), all defined below,

will be classified as unsatisfactory outcome. All other patients seen at week 96 will be classified as satisfactory outcome.

For the purposes of the analysis of the primary outcome, the week 96 analysis window is 4 weeks either side of week 96 (i.e. from 92 weeks to 100 weeks after randomisation).

Definitions

1) Ongoing requirement for TB treatment at week 96
Taking any combination of TB drugs when seen during the week 96 analysis window; or been prescribed a course of treatment for a new episode of TB relapse (meeting criteria in 6.10.2) starting in the 6 months prior to the scheduled week 96 visit date but has stopped the drugs or never started.

2) Ongoing TB disease activity at week 96
This definition will be met if at least 2 out of criteria A or B or C are satisfied, or if criterion D is satisfied.

- A. Clinical evidence:

New, recurrent, or increased severity of one of more standard TB-related symptoms (cough, one or more episodes of haemoptysis, fever, pleuritic chest pain, weight loss, night sweats) for a duration of at least 7 days or of physical signs, compared with the end of the last course of TB treatment (or period after the end of treatment if further improvement occurred subsequently) without alternative explanation(s) considered more likely in the opinion of the managing clinician.

- B. CXR evidence

CXR taken within week 96 analysis window that has abnormalities that are compatible with active TB disease (cavitation, infiltrates, consolidation) and clear evidence of progression compared with CXR at the end of the last course of TB treatment (or period after the end of treatment if further improvement occurred subsequently) without alternative explanation(s) considered more likely in the opinion of the managing clinician.

- C. Microbiological evidence (presumptive)

Sputum culture positive for *Mycobacterium tuberculosis* on 1 sample taken within the week 96 analysis window

- D. Microbiological evidence (definitive)

Sputum culture positive for *Mycobacterium tuberculosis* on 2 samples taken on separate days with at least one taken within the week 96 analysis window OR
Sputum culture positive on 1 sample taken within the week 96 analysis window plus either sputum smear positive (≥ 1 positive sample taken within week 96 window, but only if 12 weeks after completing last course of treatment), and/or GeneXpert positive (≥ 1 positive sample taken within week 96 window, if taken > 24 weeks after the end of treatment or if end of treatment result was negative).

Extra-pulmonary TB disease activity
The above criteria apply to evidence of ongoing pulmonary TB disease activity. The definition of ongoing disease activity at week 96 may also be met if the patient fulfils equivalent criteria that indicate ongoing TB disease activity at another site i.e. non-pulmonary symptoms or signs, evidence of abnormalities on another imaging test that are compatible with active TB disease with evidence of progression from an earlier comparable imaging test (if no previous test available for determining progression, the abnormalities should be of sufficient severity to explain the symptoms), and microbiological evidence (presumptive or definitive) based on samples obtained from another site.

However, the evidence from A, B and C (or the two cultures for D) should relate to the same disease site.

3) Death during or prior to week 96 window from cause possibly related to TB
All deaths where the patient had a study visit at or after week 48, was not on treatment and had no evidence of ongoing TB activity when last seen, and where the cause of death is known to be unrelated to TB or the drugs used to treat it (e.g. road traffic accident) will be classified as un-assessable. All other deaths during or prior to the week 96 window will be classified as an unsatisfactory outcome.

10.2.2 PRIMARY OUTCOME DEFINITION FOR PATIENTS NOT ATTENDING DURING THE WEEK 96 ANALYSIS WINDOW

If the patient
(a) Completed their last prescribed course of treatment AND
(b) Had at least one study visit at or after completing the course of treatment AND
(c) Did not meet the criteria for ongoing requirement for treatment or ongoing TB disease activity (as defined above) when last seen AND
(d) The last two sputum cultures obtained at study visits were negative at and following the completion of treatment (for the purposes of this definition, if patients are unable to produce a sputum sample at any designated time point at or after the completion of treatment, and this is because they have improved clinically, then the sputum will be viewed as a negative culture at that time point) AND
(e) The patient can be contacted at week 96 and confirms that they are not currently taking TB treatment (or have been prescribed TB treatment that they should be taking – information on intent to prescribe at last attended visit takes precedence over information provided by the patient), and has no current pulmonary symptoms meeting the criteria of clinical evidence of TB (see above, and discounting symptoms clearly attributable to other causes), OR the patient cannot be contacted but a designated friend / relative verifies that they have personally seen the patient within the previous 2 months (prior to week 96) and that the patient was alive, neither reported taking TB treatment nor that they had symptoms meeting the criteria of clinical evidence of pulmonary TB (above) nor were observed to have symptoms meeting such criteria

If (a) to (e) all true, classify as satisfactory.
If (a) to (d) all true, but for (e) there is evidence of ongoing need for treatment or symptoms from patient or friend/relative (if patient cannot be contacted) or the friend/relative reports that the patient is known to have died, classify as unsatisfactory.

If (a) to (d) all true, but there is no information available from patient of friend/relative to make a judgement on (e), classify as un-assessable.

If (a) to (d) are not all true, classify as unsatisfactory.

Alternative classifications will be specified in the SAP for the purposes of sensitivity analyses on the primary endpoint. One of these sensitivity analyses will include an assessment by an endpoint review committee of the components of the composite endpoint in all cases where patients have TB-related symptoms at week 96, have one or more positive cultures at week 96, or who do not attend the visit at week 96. The committee will consist entirely of independent experts with no other involvement in the trial and will review all relevant data on the clinical course of the patient (including treatment course, clinical symptoms and culture results).

10.2.3 SECONDARY OUTCOMES FOR EVALUATION OF THE MANAGEMENT STRATEGY FROM THE PATIENT PERSPECTIVE

- Acceptability of the strategy
  For patient acceptability, a total score will be calculated for each patient from the sum of the responses on the first two questions of the patient acceptability questionnaire (see Appendix 6). The response to question 3 (motivation) will be scored separately. The response to question 4 will be converted to a binary variable (recommend vs unsure/do not recommend).

- Total days on TB drug treatment
  Defined as including all retreatment episodes up to the final trial visit

- Time off work or study due to illness/treatment
  The sum of all reported time taken off work or education attributable to TB or its treatment from randomisation up to the final trial visit.

- Quality of life
  Patient responses on the MOS-HIV quality of life questionnaire will be converted to scores on 11 subscales, ranging from 0 to 100, with higher scores indicating better health. The scores are then synthesised into a physical health summary score (PHS) and a mental health summary score (MHS). The scores on the 5 levels of the EQ5D will be converted to an overall index score.

- Respiratory disability at week 96
  Respiratory disability will be defined as a score of ≥ Grade 3 on the MRC dyspnoea scale. An additional definition based on spirometry values will also be used (FEV1 < 50% predicted).[82]

- Total Grade 3 or 4 clinical adverse events
  From randomisation to the final trial visit

- Total serious adverse events
  From randomisation to the final trial visit

- Death
  All-cause mortality between randomisation and the scheduled date for the final trial visit. An additional definition will include extended follow-up to the close of trial visit

10.2.4 SECONDARY OUTCOMES FOR EVALUATION OF THE MANAGEMENT STRATEGY FROM THE PROGRAMME PERSPECTIVE

- Adherence to TB medication
  Adherence will be calculated as the proportion of prescribed daily doses missed (missed any of the prescribed pills, but excluding clinician-recommended interruptions or dose reductions for toxicity) over the initial 8 weeks of treatment and as the proportion missed over the entire time for which TB medication was prescribed (including any retreatment episodes) up to the final trial visit.

- Treatment default
  Defined as treatment interruption for more than 8 weeks, occurring during the initial 8 weeks of treatment.

A second definition will include default occurring at any time during the period when TB medication is prescribed (including retreatment episodes).

- Acquired drug resistance by week 96
  Any new resistance (phenotypic or genotypic, not present at baseline) indicating significant reduction in susceptibility to a drug to which the patient was exposed during the trial (with the exposure occurring prior to detection of the resistance mutation). Typing will be used to differentiate relapse from re-infection and new resistance occurring in a re-infection strain will not count towards this outcome.

- Acquired resistance by completion of extended follow-up
  Defined as above, but including resistance detected during the extended period of follow-up required to have 48 weeks' post-treatment follow up in those patients who were re-started on treatment later in the trial.

- Community transmission risk
  This will be estimated in two ways. Firstly, an estimate of household transmission risk will be obtained, based on the number of close household contacts exposed to the patient when smear positive. Secondly, an overall measure of the community infectiousness will be obtained, based on the duration of cough and the grade of smear positivity of the patient.

10.2.5 Health economics outcomes

- utilisation of healthcare resources
- direct medical costs
- total (direct and indirect) costs

10.2.6 Other outcomes for evaluation of boosted regimens

- Pharmacokinetic parameters
  Standard PK parameters will be obtained for each drug in the boosted regimens from the sparse sampling and the full PK assays as described in the PK sub-study (Chapter 11).

Note: For the definitions below that are based on sputum culture results, a culture will be regarded as negative if it shows no growth of mycobacteria within a period of 6 weeks or if the patient is unable to expectorate due to clinical improvement at a visit where a culture is mandated.

- Time to stable culture conversion
  Defined as the time from randomisation to the first of two negative cultures, without an intervening positive culture. Patients on boosted regimens who switch to standard treatment regimens (for toxicity or other reasons) will be censored at the time of switch. Patients who do not achieve culture conversion by 16 weeks will be censored at 16 weeks.

- Sputum culture status at 8 weeks from randomisation

- Treatment failure
  For the purposes of comparison of the efficacy of the boosted regimens with the standard regimen, treatment failure will be defined as a positive sputum culture at the end of the first period of treatment if this is confirmed by another culture-positive sample taken after the end of treatment with no more than 1 intervening negative culture between the positive end-of-treatment sample and the subsequent positive sample. The end of treatment will be considered as the time when the boosted regimen is completed (i.e. at 8 weeks, or up to 12

weeks if extended for missed dose or persistent clinical disease) or when the standard regimen is stopped in Arm A (i.e. at 24 weeks, or later if treatment extended). When the boosted regimen is not continued for at least 8 weeks (e.g. because of switch to standard treatment), the patient will be censored (removed from numerator and denominator). When treatment for at least 8 weeks with the boosted regimen has been completed, but the patient switches to standard treatment instead of stopping treatment, then the last culture taken on boosted treatment (provided taken at or after 8 weeks) will be regarded as the end of treatment culture. In these cases a confirmatory culture will not be required to meet the definition of treatment failure. The WGS (or alternative sequencing method if WGS not performed) from the latest positive culture and the baseline isolate will be compared and if the sequences differ sufficiently (thresholds will differ by sequencing method and will be defined in detail in the SAP), this will be reclassified as reinfection rather than treatment failure.

- Relapse to week 96
  For the purposes of comparison of the efficacy of the boosted regimens with the standard regimen, relapse will be defined as two consecutive positive sputum cultures obtained after the end of the first period of treatment, unless classified as treatment failure (see above). To meet this definition the second positive culture should be obtained on a separate day and within a 12-week period from the first positive sample. When the boosted regimen is not continued for at least 8 weeks (e.g. because of switch to standard treatment), or when the patient switches to standard treatment instead of stopping treatment, the patient will be censored (removed from numerator and denominator). As for treatment failure, above, sequencing will be used to exclude re-infections.

- Combined treatment failure or relapse to week 96
  The sum of treatment failure and relapse defined as above

- Standard treatment re-introduction by week 96
  Defined as re-introduction of standard treatment for any cause

- Grade 3 or 4 adverse events
  All grade 3 or 4 adverse events (clinical and laboratory) occurring during the initial period of TB treatment

- QTc prolongation
  Defined as one or more episodes of QTc prolongation (QTc > 500ms) during follow-up.
  Episodes of QTc prolongation from baseline of >60ms during follow-up.

- Serious adverse events occurring during the initial course of TB treatment considered related to the study medication to which the patient was randomised

- Change in extent of CXR disease from baseline to week 8

10.3 SAMPLE SIZE

The sample size estimate is based on a non-inferiority margin of 12% for the difference in response between a boosted regimen and the control in the primary endpoint of unsatisfactory outcome at 96 weeks. Previous trials evaluating shorter regimens for the treatment of drug-sensitive TB used a margin of non-inferiority of 6%[13,15] but this was based on the addition of only a single drug. The

justification for the margin of 12% for TRUNCATE evaluating completely new regimens is as follows. It is estimated that the case fatality rate for untreated smear positive pulmonary tuberculosis is 70%, ie a cure rate of 30%.[83] If we assume 90% satisfactory outcomes after two years on standard treatment, this gives a standard treatment effect (denoted as M1 in the FDA guidance document)[84] of 60%. Selecting a non-inferiority margin of 12% (denoted as M2 in the FDA guidance document) means that a relative proportion of 80% of the standard treatment effect is preserved in treatment arms that are declared non-inferior on the primary endpoint. This exceeds the general recommendation in the FDA document of preserving at least 50% of the treatment effect. The same argument (and non-inferiority margin) has been used for another ongoing clinical trial evaluated novel regimens for the treatment of DS-TB.[85]

We estimate that the proportion of patients meeting the primary endpoint of unsatisfactory outcome in the control arm will be 10% based on the following assumptions:[13]

(i) the proportion with ongoing requirement for TB drug treatment or with ongoing TB disease activity at week 96 who will be classified as unsatisfactory outcome is estimated as 2%. This is based on data from REMoxTB trial that shows that <1% of patients on the control arm initiated treatment in the last 26 weeks of follow-up (and were therefore on treatment at the end of follow-up) and <1% had positive cultures at the end of follow-up.

(ii) The proportion who will have died by week 96 (minus those who died after week 48 and for reasons clearly unrelated to TB or treatment – classified as un-assessable, see below) is estimated as 3% based on 2.5% of deaths in REMoxTB in the control arm.

(iii) The proportion who fail to attend at week 96 and who cannot be contacted and confirmed to be clinically well is assumed to be 5%. This is difficult to estimate precisely from previous trials which were not designed as treatment strategy trials and so patients were not often followed for the full duration if retreatment was started, but it is unlikely to exceed 5% in this trial.

We also estimate that 10% will be classified as un-assessable (and therefore will be excluded from the primary analysis) based on estimates from the REMoxTB, RIFAQUIN and OFLOTUB trials (although these trials used a broader definition of un-assessable).

We assume that:
(i) 2 boosted arms will be included in the final analysis
(ii) the proportion of patients with an unsatisfactory outcome in each of these arms will also be 10% (i.e. the same as the standard-of-care arm).

With these estimates and assumptions, with 87% power to conclude non-inferiority at a 1.25% one-sided significance level (reduced from the standard 2.5% to allow for the two final comparisons) the required sample size for the control arm and each of the boosted arms at the final analysis will be 180 patients per arm. With four intervention arms and one control arm, the maximum sample size will be 900.

The actual power for determining non-inferiority may be lower than 87% because of the potential for discontinuing arms at the interim analyses that might ultimately prove to be non-inferior. Based on simulations, for a non-inferior arm with a true probability of treatment failure/relapse of 10%, the power for concluding non-inferiority would be 83%.

This sample size of 900 is a maximum sample size which may be lower if recruitment to arms is discontinued at interim analyses (thereby reducing the number of patients randomised to those arms).

Even in arms that are non-inferior on the primary endpoint, if the true probability of treatment failure/relapse exceeds 20%, this would not be a desirable regimen and a high probability of discontinuing recruitment to such an arm at an interim efficacy analysis is preferred. Conversely, the probability of discontinuing recruitment to an arm for which the true probability of treatment failure/relapse does not exceed 10% should be low. Simulations were performed to test the performance characteristics, based on the sample size and stopping rules and timings of interim analyses described in this section and below. These simulations show that an arm with a hazard ratio of time to stable culture conversion of 1.3 compared to the control arm and a true probability of treatment failure/relapse of 10% would have a 5% chance of being recommended for discontinuation at either first or second interim analysis. Conversely an arm with a true probability of treatment failure/relapse of 25% would have a 77% chance of being recommended for discontinuation.

Simulations show that the maximum family-wise error rate (the type I error under the assumption that none of the arms are non-inferior but no arms are dropped at interim analyses) is maintained at 4.0%.

10.4 INTERIM MONITORING & ANALYSES

An IDMC will be established to monitor the trial. The IDMC reviews will include data on recruitment, adherence to randomised strategies, culture conversion, rate of relapse and treatment failure, toxicity, switches to standard treatment regimens and other key safety and efficacy outcome parameters. The IDMC will also consider findings emerging from any other studies that provide relevant context to assessment of the safety of the drugs and regimens used in this trial. An IDMC Charter will be drawn up that describes the membership of the IDMC, relationships with other committees, terms of reference, decision-making processes, and the timing and frequency of interim analyses (with a description of stopping rules and/or guidelines, if any). The IDMC is advisory to the TSC and any decisions regarding stopping or continuing recruitment in certain arms lies with the TSC.

10.4.1 IDMC MEETING SCHEDULE

The IDMC will meet every 6 months from the start of trial recruitment and at the following fixed time points related to the trial stages:

1. After Stage 1 (pilot safety stage). As soon as possible after the first 10 patients enrolled in the control arm of the trial have reached 4 weeks of follow-up. This meeting will be for the purpose of reviewing initial safety data to ascertain whether there are any safety concerns that might require additional safety monitoring or the cessation of one or more of the treatment arms on safety grounds.
2. After Stage 2 (early efficacy stage). As soon as possible after the first 30 patients enrolled in the control arm of the trial have reached 6 months post-randomisation. The first interim efficacy analysis will be done at this meeting for potential recommendations on discontinuing recruitment to arms.
3. After Stage 3 (qualifying efficacy stage). As soon as possible after the first 70 patients enrolled in the control arm of the trial have reached 6 months post-randomisation. The second interim efficacy analysis will be done at this meeting for potential recommendations on discontinuing recruitment to arms.

Additional ad hoc IDMC meetings will be held in the following circumstances:

1. As soon as possible following the identification of a very high rate of early treatment failure / relapse in one or more of the boosted arms, defined as 10 or more confirmed cases before 20 patients

have completed more than 2 months of post-treatment follow-up in any arm. This meeting, if required, will be triggered by the un-blinded statistician.

2. As soon as possible if 2 or more cases of new drug resistance are identified at any time point in any one of the boosted arms. Confirmed cases of new drug resistance will be as defined in the trial laboratory manual. Rapid typing (preferably by WGS) will be performed where possible to confirm similarity with baseline strains and to confirm the presence of new mutations. This meeting, if required, will be triggered by the un-blinded statistician.

3. As soon as possible following the identification of a very high rate of early treatment failure / relapse in the subgroup of patients considered to be at higher risk of such outcomes (HIV positive, smear 3+, cavitation > 4cm, see Section 4.2) if a decision is made to enrol these patients from Stage 3 of the trial. A very high rate is defined as more than 10 confirmed treatment failure/ relapse outcomes before 20 patients reach 8 weeks post-treatment across all of the boosted treatment regimens combined. The IDMC may recommend the cessation of recruitment to any or all of these higher risk groups at any stage if there is concern.

4. At the discretion of the IDMC or at the request of the trial management team or the TSC to review any safety or efficacy concerns identified by one or more of those groups.

10.4.2 First interim efficacy analysis

The first interim efficacy analysis will occur after data are available for 30 patients enrolled in the control arm who have reached 6 months post-randomisation (details of timing given above). The interim efficacy analysis will be based on the proportion of patients who have met the criteria for treatment failure/relapse in which the boosted arms are compared against a pre-determined threshold (see below) and the time to stable culture conversion in which boosted regimens are compared to the control arm.

Treatment failure / relapse

This will be based on the definitions in Section 10.2.6 above. It is considered that if the treatment failure/relapse rate with a boosted regimen is above 20% then the strategy using this regimen is unlikely to be of value for programmes or acceptable to patients (whether lower levels will be acceptable will depend on a full consideration of the risks and benefits of this approach which can only be done at the end when the trial has provided the necessary data for a comprehensive evaluation of this strategy). As the point estimate will not be precise, at the first interim analysis the IDMC will be asked to consider recommending stopping recruitment in any arm where the combined treatment failure/relapse rate on a boosted regimen exceeds 25%.

Time to stable culture conversion

This is defined in Section 10.2.6 above. The IDMC will be asked to consider recommending stopping recruitment in any arm where the hazard ratio of the time to stable conversion on a boosted regimen as compared to the control does not exceed a threshold of 0.8. Note that evidence for a statistically significant decrease in the time to culture conversion is not a requirement for an arm to continue, see below.

Toxicity

The IDMC will monitor toxicity of the boosted regimens in a number of ways including:

(i) The rate of standard treatment re-introduction for toxicity, defined as switch to standard treatment (or modified standard treatment if toxicity to standard drugs) for intolerance or adverse events.

(ii) The overall adverse event profile of a regimen and whether this would seriously limit its acceptability to patients/programmes or whether there are specific adverse events that need

excessive medical intervention to manage the toxicity that would not be feasible in programme settings. The IDMC will give more weight to events graded as 3 or greater.

(iii) Death and the causes of death in all arms (with a low threshold for termination in the event of an unexplained death in the arm containing bedaquiline).

Overall assessment

The IDMC will be able to recommend discontinuation of recruitment to an arm based on a combination of efficacy and toxicity factors above, or taking into account other considerations.

In considering the treatment failure/relapse rate the IDMC will also review data to evaluate the impact of those outcomes in terms of clinical morbidity, drug resistance and community transmission risk.

10.4.3 Second interim efficacy analysis

This will occur after 70 patients have been enrolled (details of timing given above). The second interim analysis will be based on the same factors as the first interim analysis, but with different thresholds:

(i) treatment failure/ relapse with a threshold of 20%

(ii) Time to stable culture conversion with a threshold of 0.9

If the combined treatment/relapse rate exceeds 20% in a boosted arm or if the hazard ratio of the time to stable conversion as compared to the control does not exceed a threshold of 0.9 then the IDMC will be asked to consider recommending stopping recruitment in that arm.

Safety and combinations of these factors will again be taken into account in the recommendations.

10.4.4 Rationale for choice of thresholds for interim analyses

The thresholds for the interim analyses are chosen to ensure high power for identifying arms that correspond to desirable treatment strategies, but stopping recruitment to arms early that are likely to have high rates of treatment failure or relapse. The precise numbers are based on a simulation study undertaken in preparation for the trial, see Section 10.3.

10.4.5 IDMC recommendations

The IDMC will be asked to make recommendations on the following:

(i) Whether the trial should continue in its present form, or recruitment be stopped entirely, or be modified by discontinuing recruitment to one or more boosted arms.

(ii) For a boosted arm for which it is recommended that recruitment be discontinued, the IDMC will also advise on the following:

- whether patients in that boosted arm should continue to complete their course of treatment as randomised, or whether they should switch therapy. The default regimen, if a recommendation is made to switch therapy, will be the standard TB treatment with the total duration of treatment shortened by the number of doses of the boosted regimen completed prior to treatment switch (Section 6.8). The IDMC may recommend an alternative regimen if appropriate.
- whether patients who have completed treatment in that boosted arm should be observed as specified in the protocol, or with a higher frequency of monitoring, or whether they should be re-treated regardless of relapse. If the patients should be retreated the default will be the standard regimen as described above, but the IDMC may recommend an alternative if appropriate.
- whether additional safety monitoring or other interventions are needed for patients in that arm

(iii) whether additional safety monitoring is needed for the trial overall, or in one particular arm
These recommendations could be made at the first and/or second interim analysis stage, or at any of the scheduled or ad hoc meetings based on safety, efficacy or drug resistance.

(iv) whether the protocol-mandated dose of any trial medications should be reduced in order to minimise toxicity

(v) at the time of the first interim efficacy analysis and upon completion of Stage 2, whether the IDMC would recommend that the planned expansion of enrolment to include higher-risk patients (HIV positive, cavitation >4cm, smear positivity 3+ as defined in Section 4.2, subject to the criteria specified in section 4.2.1), should proceed or whether this expansion should be deferred.

10.4.6 Reporting and implementation of decisions

If the IDMC makes a recommendation to the TSC to discontinue recruitment to a treatment arm, the essential reasons for that decision will be shared in confidence with the TSC if so requested.

The TSC will meet by teleconference as soon as possible and at the latest within 1 week of a recommendation from the IDMC to discontinue recruitment to an arm.

The TSC will make the appropriate recommendations to the Trial Management Group for implementation of discontinuation of recruitment to an arm. The TMG will immediately notify the statistician in charge of randomisation, and for each boosted arm identified, randomisation will cease within 12 hours of notification.

10.5 OUTLINE ANALYSIS PLAN

This Section summarises the main approaches to the analysis of data. The analyses will be described in detail in a full Statistical Analysis Plan (SAP). Where details differ from the outline below, the SAP will take precedence.

10.5.1 Analysis populations and overall approach

Modified intention-to-treat (mITT) population
The mITT population is defined as all randomised patients, with the exclusion of patients with isolates taken before or on the day of randomisation that are subsequently found to be resistant to rifampicin on phenotypic DST.

Per protocol (PP) population
The PP population will be the same as the mITT population but with the additional exclusion of patients who do not complete a full 8 weeks of treatment with the randomised combination (those patients who interrupt treatment but complete a full course, who reduce doses due to toxicity or tolerability but do not stop the drug entirely, and those who stop a single drug after completing at least 6 weeks' treatment at full or reduced dose will be included in the per protocol population; those who add new drugs to the assigned regimen will be excluded from the per protocol population).

Overall approach
The primary analysis for this trial will be done on the mITT population. Although it is traditional for equal importance to be given to analyses based on mITT and PP populations in non-inferiority trials, in a strategy trial such as this, where treatment switches and re-treatment form an essential part of the strategy, a PP analysis is of less relevance. The PP population will be used for a secondary efficacy

analysis and also for comparing the early laboratory marker results between the various boosted regimens.

The main analysis will include all arms, even those for which randomisation was terminated at an interim analysis, although the results of such arms will be interpreted with caution due to the wider confidence intervals with smaller number of patients and also the potential for bias introduced by early termination.

Any arms which continue through to the final analysis will be compared individually with the standard TB management strategy arm. Where there is more than one TRUNCATE-TB management strategy arm that continues through to the final analysis and these have similar efficacy on the primary outcome measure (point estimates differ by less than 5%), a supplementary analysis will be performed in which these arms are combined and compared with the standard TB management strategy arm on primary and secondary outcomes.

Data analysis of the primary outcome and secondary outcomes will control for site and potential relapse risk. Strata of a potential relapse risk may be grouped across multiple sites in the analyses according to their similarity (e.g., by country or hospital size/level), depending on the numbers of patients recruited into the strata. Full details of the analysis will be given in the SAP.

10.5.2 PRIMARY ANALYSIS

For the primary endpoint comparison, the difference in proportions with an unsatisfactory outcome and a two-sided 97.5% confidence interval for that difference will be calculated, comparing each TRUNCATE-TB management strategy arm with the standard TB management strategy arm using the mITT population (see 10.5.1).

A TRUNCATE-TB management strategy arm will be declared to be non-inferior to the standard TB management strategy arm if the upper limit of the two-sided 97.5% confidence interval for the difference between the two arms in the proportion of patients with a satisfactory outcome (standard TB management strategy minus TRUNCATE-TB management strategy arm) is less than 12% (for justification of non-inferiority margin, see Section 10.3).

It is important to note that the decision on the appropriateness and utility of the TRUNCATE-TB management strategy will depend as much on consideration of key secondary outcomes as it will on meeting a pre-specified non-inferiority threshold for the primary outcome.

10.5.3 SECONDARY ANALYSES EVALUATING THE TRUNCATE-TB MANAGEMENT STRATEGY

Secondary analyses will compare TRUNCATE-TB management strategy arms to the standard TB management strategy on a range of secondary outcomes relevant to the patient perspective (Section 10.2.3) and the programme perspective (Section 10.2.4).

For secondary outcomes that are expressed as proportions , $\chi 2$ test will be used to compare arms with a P value less than 0.05 taken to indicate a significant difference. Fisher's exact test will be used for comparison of proportions with few events. Secondary outcomes expressed as continuous variables will be compared between arms by linear regression. For time-to-event ouctomes, arms will be compared by Kaplan-Meier plots and Cox proportional hazards regression, if the proportion hazards assumption is reasonable.

A general linear mixed model will be used to compare the treatment arms in terms of changes from baseline in the summary PHS and MHS quality of life indexes over the follow-up period. A similar approach will be used for the EQ-5D data over the duration of follow-up. Harms will be reported as recommended by Ioannidis et al.[86] Mortality will be tabulated by cause for each randomised arm. The frequency of grade 3 and 4 adverse events will be tabulated by body systems and by randomised arms and the arms will be compared using Fisher's exact test. Serious adverse events will be tabulated by category and by randomised arms and compared in a similar way.

10.5.4 Health economic and socioeconomic analyses

The health economic analysis for the study will consider the patient, the programme, the health system (direct medical costs) as well as the overall societal cost (inclusive of indirect medical costs) over the period of the trial. Costs will cover the use of TB medication, laboratory tests, hospital, primary care and community health services. Routinely available estimates of unit costs that best reflect long run marginal opportunity cost will be attached to resource use, to obtain a cost per patient over the period of follow-up. For the within-trial analysis, the differential cost of the treatment interventions will be related to their differential outcomes in terms of the primary outcome. The relative cost-effectiveness of the alternative management strategies will then be assessed using standard decision rules and a full stochastic analysis will be undertaken. A cost-utility analysis will also be conducted using the EQ-5D data collected at each visit. Further scenario analysis will be conducted, including the analysis of a maximum "break-even" rate of relapse which offsets the net present value of the intervention benefits.

Depending on trial findings, the within-trial analysis will be augmented by extrapolation beyond the trial follow-up to predict the implications of any difference in clinical endpoints in the trial for subsequent quality-adjusted survival duration and long-term resource costs. This will inform the question of whether any differences in management costs between the treatment arms are offset by reduction in other treatment costs or health improvements in the long-term.

The data collected from the socioeconomic status battery will be transformed into both continuous and binary classifications of socioeconomic status with living standards being dichotomised into high, medium and low outcomes. The data will be used to characterise the patient population for assessment of trial generalisability. The change in socioeconomic status from baseline to week 96 will also be compared between treatment arms to determine whether there is any advantage or disadvantage of the TRUNCATE-TB management strategy on socioeconomic status.

10.5.5 Other analyses evaluating the boosted treatment regimens

Other analyses will compare the boosted treatment regimens with the standard TB treatment regimen on a range of outcomes including pharmacokinetics (Section 11.6), microbiological efficacy and toxicity.

Pharmacokinetic anlayses will be perfomed as described in Section 11.5. The approach to other analyses will depend on the nature of the data and will follow those described in 10.5.3 above.

10.5.6 Exploratory analyses of biomarkers predicting outcomes with boosted regimens

Although relapse and re-treatment form an integral part of the TRUNCATE-TB management strategy, there may nevertheless be value in refining the strategy to diminish the risk of relapse when implemented in practice. Approaches might include optimizing patient selection at the time of starting treatment or adjusting treatment duration based on initial response to treatment.

At the end of the trial, exploratory multivariable analyses will be performed examining the relationship between potential biomarkers and relapse following the boosted two month regimens. In addition to the main analysis of the relationship between biomarkers and relapse (which is the gold standard for sterilisation), analyses may be performed looking at associations between biomarkers and other traditional surrogate markers of response (such as sputum culture status at week 8).

Some of these biomarkers for analysis will be available from data collected as part of the main trial, including demographic variables (e.g. age, gender), clinical variables (e.g. duration and severity of symptoms; body mass index), imaging variables (e.g. CXR cavitation), routine laboratory test variables (e.g. haemoglobin, white cell count), and specific TB laboratory test variables (e.g. sputum smear status, GeneXpert cycle threshold, time to positivity on liquid culture).

Analyses will also include estimates of drug exposure obtained from the population PK model.

The analyses may also include promising novel biomarkers that have been identified (by other research) during the course of the trial. Sputum (at trial entry only), blood and urine samples are stored at key time points in the trial to enable analyses of such biomarkers (as yet unidentified), should any become available.

Once the main trial analyses have been completed, the dataset and biological samples may be used for validation of biomarkers in development, with proposals approved by the TSC.

11 PHARMACOKINETIC SUB-STUDY

11.1 BACKGROUND

This Section describes the pharmacokinetic sub-study that is performed in addition to the sparse drug levels measured in all patients as part of the main protocol, as described in Section 7.3.11.

The regimens used in the TRUNCATE-TB trial have been selected on the basis of combining drugs with likely sterilising efficacy as well as on the basis of careful consideration of safety, toxicity and predicted drug-drug interactions. The pharmacokinetics of the individual drugs has been in most cases well described and the PK interactions within the regimens can be predicted. The relevant existing PK data for the regimens is summarised in Appendix 13.

However, there are no studies that have described the full PK for these particular combination regimens and such data are needed to support the clinical efficacy and safety data that will be obtained from the trial and to develop a population PK model in conjunction with the sparse PK values obtained in the main trial in order to be able to relate drug exposure to outcomes.

The main aims of this sub-study are:
1. To describe the PK of the drugs used in combination in the study regimens
2. To build a population PK model to examine exposure-response relationships (with sparse PK data from the main study)

11.2 SELECTION OF PATIENTS

Participants will be considered eligible for enrollment in this sub-study if they fulfil all of the inclusion criteria and none of the exclusion criteria listed below.

11.2.1 PATIENT INCLUSION CRITERIA

1. Taking all study medications in their randomised treatment arm at least at 50% of the protocol-recommended starting dose on the day of PK study
2. Willing and able to provide written informed consent to participate in the PK sub-study

11.2.2 PATIENT EXCLUSION CRITERIA

1. Taking concomitant medication with known or potential interactions with the study medications
2. Has a condition that may cause malabsorption of drugs
3. Creatinine clearance of <60ml/min (calculated using Cockcroft-Gault equation-see Appendix 5) on the last test done prior to the sub-study
4. ALT greater than 3 times the upper limit of normal on the last test done prior to the sub-study
5. Other reasons why the patient may not be suitable for the sub-study in the opinion of the treating clinician

11.3 SELECTION OF SITES

Sites will be eligible for recruiting patients for the PK sub-study if they have access to the appropriate local facilities for the timely processing and storage of blood samples, and have access either nationally or by international shipment (if permitted by national regulations) to one of the designated central study laboratories where the PK analyses will be performed.

11.4 NUMBER OF PARTICIPANTS

A total of 12 to 16 patients will be recruited from each of the treatment arms (80 patients in total). If an arm is stopped early by the IDMC it may not be possible to enroll 12 patients, and PK analysis for that arm will be performed with all data that are available when recruitment to the other arms of the sub-study is complete.

11.4.1 ENROLLMENT PROCEDURES

All eligible patients at the selected sites will be invited to participate and enrolled consecutively, with sites endeavoring to recruit equally across all the treatment arms. Patients may be approached about the sub-study at any point from first contact about the main TRUNCATE-TB trial until the close of the window for the PK visit (i.e. week 8 plus 7 days). A consent form that is distinct from the main study forms will be used for this sub-study (see Appendix 14).

Patients will be informed that participation is voluntary and that they may refuse without affecting their participation in the main trial, or their medical care. Patients taking part in the sub-study will be compensated separately from the main trial for their travel expenses and the additional time and inconvenience involved.

A screening log will be kept as a record of all eligible patients and the reasons for non-participation if given. Eligibility may change between the time of providing consent and the time the sub-study is performed. Patients may also decide to withdraw consent prior to or during the sub-study day for their own reasons and without necessarily having to justify their decision.

When 16 patients on a particular treatment arm have completed a full PK study, the trial management team will inform all participating sites to cease recruitment of patients on that study arm.

11.4.2 INDIVIDUAL PARTICIPANT VISIT SCHEDULING

The PK study day will be at 8 weeks (window 42 days to 63 days) from randomisation. The PK study may be done on the same day as the week 8 main TRUNCATE-TB trial study visit, but may also be performed on a different day for patient convenience or logistical reasons.

11.4.3 PK PROCEDURES

Patients will be requested to avoid strenuous exercise and the use of alcohol, grapefruit juice, over-the-counter medications (including antacids), vitamins or mineral supplements, herbal medications, recreational drugs or other medicinal products for 48 hours prior to the PK study day. They will be requested to fast from midnight on the evening prior to the PK study day (water allowed freely) and to omit their dose of medication on the morning of the PK study day.

Patients will be asked to present in the early morning at the study site, and will stay throughout the study if the facilities allow overnight accommodation. Alternatively (or if the facilities do not permit overnight stay) the patient will go home after the 12-hour sample has been taken and will return the following morning for the 24 hours sample to be taken.

Upon arrival at the study site, eligibility for sub-study participation will be confirmed. An intravenous cannula will be inserted through which the PK samples can be drawn to maximize patient convenience.

The study medications will be taken at the same time (over a maximum period of 15 minutes). Patients randomised to Arms A, B and C will take the study medications with water on an empty stomach, and 2 hours after will receive a full breakfast. Patients randomised to Arms D and E will take the study medications with a full breakfast. The time of study medication ingestion will be recorded.

Patients will then be given a full lunch, and subsequent snacks and drinks as required. Water may be consumed freely throughout the study day.

11.4.4 PK SAMPLING SCHEDULE

Blood sampling will be performed at the following times:

Sample 0:	Prior to treatment administration
Sample 1:	1 h after treatment administration
Sample 2:	2 h after treatment administration
Sample 3:	3 h after treatment administration
Sample 4:	4 h after treatment administration
Sample 5:	5 h after treatment administration
Sample 6:	6 h after treatment administration
Sample 7:	8 h after treatment administration
Sample 8:	12 h after treatment administration
Sample 9:	24 h after treatment administration

The sample should be taken as close as possible to the specified time after treatment administration (window for sampling – 5 minutes before to 15 minutes after the specified time). The exact times the sample is collected (i.e. when the blood collection tube is filled) will be recorded. The 24 hour time-point may be omitted for logistical reasons if necessary. 5ml of blood will be drawn at all time-points. Blood samples will be centrifuged within 30 minutes of collection and the plasma divided into at least 3 aliquots (4 aliquots at 0, 1, 2, and 8 hour time points) of approximately 0.5ml each.

A 12-lead ECG will be performed pre-dose, and at 2 hours and 5 hours post-dose.

If the PK study is not done on the same day as the week 8 study visit then a record of symptoms, concomitant medications, vital signs and the results of targeted physical examination will be made. However no additional tests will be performed (the results of tests done on the week 8 study visit will be regarded as valid for the PK study day).

11.5 ANALYTICAL METHODS

Levels of all study drugs will be measured using liquid chromatography-tandem mass spectrometry in up to 4 designated study laboratories using common protocols.

Participating laboratories will undergo cross-validation by testing approximately 5% of the samples tested in each of the other participating labs. Analysis will be done batched when all the sub-study samples have been collected, although the cross-validation may be performed earlier on a smaller number of samples.

11.6 PK DATA ANALYSIS

Data from all laboratories will be pooled centrally and analyzed using a non-compartmental analysis (NCA) approach to give the following PK parameters:

- Area under the curve for plasma drug concentrations vs. time, until 24 hours after dosing (AUC_{24})
- Peak plasma concentration (C_{max})
- Plasma concentration at 24 hours after dosing (C_{24})
- Time to peak plasma concentration (T_{max})
- Half-life

11.6.1 SAMPLE SIZE AND STATISTICAL ANALYSIS

The sample size of 12-16 per arm is typical of such PK studies[87] and should allow the description of the PK parameters of interest with adequate precision and provide robust estimates that can be used to support the population PK modeling. Summary statistics (median and standard deviation) for the PK measures of individual drugs in each regimen will be presented. Drug levels will be compared with historical data from other studies and against putative thresholds considered to provide adequate efficacy against TB. Where appropriate, levels of drugs will be compared across arms to provide information on the potential magnitude of the effect of drug interactions, and statistical significance will be assessed using parametric or non-parametric approaches depending on the distribution of the data.

The data from this study, together with the sparse PK levels obtained in the main study, will be used to generate a population PK model, which can be used to predict the PK parameters from sparse sampling in the main study protocol.

11.7 SUB-STUDY DATA MONITORING

The PK sub-study will be monitored (source data verification and data review) using the mechanisms established for the main TRUNCATE-TB trial. Sub-study data will be made available to the IDMC initially and (upon their approval) to the TSC. The TSC will make a decision on whether the results should be released to the investigators and whether the data should be published in advance of the completion of the main trial.

12 ETHICAL CONSIDERATIONS AND APPROVALS

12.1 OVERALL ETHICAL DESIGN PRINCIPLES

This is an innovative trial that tests a new strategy for the management of TB and the design therefore differs in a number of important ways from standard TB treatment trials. Ethical issues have been considered carefully during the development of the trial. The trial design and proposed implementation adhere to the core principles of ethics articulated by the Belmont Report,[88] namely respect for persons, beneficence and justice. The trial is discussed in the context of these principles below.

12.2 RESPECT FOR PERSONS

The autonomy of patients is assured by the process of informed consent (described in Section 4.3.1). Written site procedures will ensure that the consent process is conducted in an appropriate way (see Section 4.3.1) and by appropriately trained individuals. At the site initiation visit, specific training will be provided in the presentation of the trial to patients. Consent forms will be translated into the local language(s) at the trial site and approved by local ethics committees (Section 12.9). Sites will be encouraged to evaluate critically the success of communicating the key elements of the trial to the patients who decide to participate. If there are significant changes in risk during the trial, re-consent will be obtained. The trial will not recruit patients from vulnerable groups such as prisoners or those with mental illness.

12.3 JUSTICE

Justice is assured in the following ways.

The inclusion criteria are kept as broad as possible, with exclusion criteria limited to those essential for minimising risks to patients.

The research will be conducted in the populations and settings that it is intended to benefit. The clinical teams at the trial sites will gain experience with using new or repurposed drugs that are likely to become more widely used in the treatment of TB. Site investigators benefit from the experience of being involved in a rigorous TB clinical trial protocol and specifically through their involvement in the trial management team, participation in the interpretation of the data and academic outputs of the trial.

The drugs used in the trial are mostly licensed drugs that are no longer subject to patent protection. Most are available at relatively low cost from generic manufacturers, and costs would be expected to fall further if the evidence from this trial led to increased use of one or more of these repurposed drugs in treatment of TB. The most recently approved drug-bedaquiline-is still protected by a patent although the company involved has made a public commitment to make this drug available at reduced pricing to populations in need. If shown by this trial to have major benefit in the treatment of TB, then there is no barrier in principle to the affected populations getting access to these drugs.

This trial has been designed with input from and the support of community representatives and from the Global Tuberculosis Community Advisory Board (TBCAB), an organisation that represents the TB affected community.

12.4 BENEFICENCE

The principle of beneficence involves the need to consider the risks of the trial for the participant and to ensure that these risks are minimised; to consider the benefits of the trial for the participant and for society; and to weigh the risks of the trial against the benefits to ensure that the balance is appropriate.

Prevention and avoidance of harm (nonmaleficence) is complicated as often research patients are put at risk of unknown and unknowable harms in the hope of discovering better ways of treating disease for patients in the future.

This trial tests a strategy of treating patients for a much shorter duration than normal, observing closely, and re-treating the minority who relapse with a longer course of treatment. This strategic approach has not been tested before in a TB trial. However there are precedents from the the field of HIV, where numerous trials have been done to reduce the number of drugs or to stop treatment accepting that decreasing the intensity of treatment from the maximal standard-of-care intensity or duration may have benefits to individuals and the community that outweigh the consequences of periods of unchecked microbial replication.[89-92] To date these approaches have not been shown to be of benefit although there are still trials that continue to challenge this question. Such strategic trials have undergone extensive review by multinational ethics committees and therefore serve as an ethical precedent for the principle of testing a strategy that explicitly offsets the benefits (to the majority) of treatment reduction against a small risk of disease progression/recurrence (to the minority).

For assessing beneficence, the risks and benefits of the trial intervention groups are most appropriately compared against the locally-relevant standard-of-care. This is particularly important in TB trials, because outcomes from standard TB treatment in clinical trials are known to be better than standard-of-care treatment in local programme settings because of the interaction with enhanced ancillary care and monitoring given in a trial (which is critically important in TB, because adherence plays an important part in obtaining good ouctomes).

12.5 RISKS OF THE TRIAL FOR THE PATIENT IN THE TRUNCATE-TB MANAGEMENT STRATEGY ARMS

The risks in the TRUNCATE-TB management strategy arms are discussed below. These differ somewhat for the individual arms, especially concerning drug toxicity. They will be discussed in general with reference to detailed information elsewhere in the protocol where needed.

12.5.1 RISK OF TREATMENT FAILURE OR RELAPSE AND CONSEQUENT HARM

Probability of treatment failure/relapse

The TRUNCATE-TB management strategy involves reducing the treatment duration to a threshold at which it is sufficient to cure the majority of patients with TB whilst avoiding over-treatment of the majority, and providing additional resources to follow-up, identify and re-treat those patients who are not cured with the short treatment. It is therefore possible that patients randomised to the TRUNCATE-TB management strategy arms will experience a higher rate of treatment failure/relapse that would be the case if they were to be treated outside the trial. However, there are a number of mitigating factors that may decrease this risk:

1. The boosted regimens have at least two modifications that are likely to increase sterilisation with a short course of treatment. The evidence for the efficacy of these regimens is given in Section 2.5. The drugs have been tested alone and in combination in animal models, and there are randomised controlled clinical trial data to show that the drug modifications add to sterilising activity. The precise regimens in this trial have not been compared with standard treatment in the mouse model. However, it is increasingly accepted, following the recent experience with fluoroquinolone trials, that neither the mouse model nor phase 2 surrogate endpoint studies can reliably predict relapse rates and many investigators now support an approach of advancing potential regimens early into definitive clinical trials.[13]

2. Treatment duration will be extended to up to 12 weeks in those patients (likely a small proportion) who have evidence of ongoing TB disease activity at 8 weeks or who miss doses (Section 6.8.2). Patients who remain symptomatic after completing the boosted regimen (likely a very small proportion) may switch to standard treatment to complete a 6 month-course (see Section 6.8.4).

3. Treatment will be switched to standard treatment for a total regimen duration of at least 24 weeks in patients who are unable to tolerate or who interrupt the boosted regimen for 14 days or more (see Section 6.8.4).

4. Patients who may be predicted to require longer periods of treatment will either not be enrolled in the trial (e.g. those with uncontrolled diabetes or taking immunosupressive treatment) or will only be enrolled in the final stage of the trial after the IDMC has reviewed safety and efficacy in a substantial number of patients enrolled in the early stages. By careful patient selection we increase the chances of cure with a short regimen.

5. The opportunity to be treated with a much shorter course of treatment as well as the close relationship with the trial team is likely to be motivating for for patients and this is likely to increase adherence. Furthermore, the trial will provide resources to support supervised treatment through DOT. The increased patient motivation and support provided is likely to produce high rates of treatment adherence which will in turn minimise the treatment failure/relapse rate.

Based on previous experience with short-course regimens of 3 months duration with standard drugs (7%-13% relapse)[11] in clinical trials together with the mitigating factors listed above, we estimate that the combined treatment failure/relapse rate will be under 15%. An important protection offered for the patients in the trial design is that of rigorous oversight by the IDMC that will review interim data and recommend stopping recruitment to any arm where the risk of combined treatment failure/relapse exceeds 20%.

The magnitude of the increase in probability of treatment failure/relapse for patients choosing to take part in this trial is difficult to estimate because it also depends on the probability of treatment failure/relapse that they would have with treatment in the the programme setting, which is variable. Typically, based on WHO and cohort data, the non-cure rate in programmes is around 14% with a subsequent relapse rate of around 8%.[1,5,6] With the protections offered by the trial and by the IDMC oversight, the probability may not be substantially higher in the trial than the patients would experience if they were to receive treatment outside of the trial.

Impact of treatment failure/relapse

In addition to evaluating the probability of the occurrence of these outcomes, an assessment of risk also requires an estimate of the impact of these outcomes. These are discussed in turn below, with mitigating factors:

1. Clinical morbidity

The extent of clinical morbidity associated with treatment failure/relapse in this trial is likely to be modest. There is a general perception that relapses tend to be clinically milder than the original TB episode which may in part be explained by faster diagnosis and initiation of treatment in a second episode, but may also be due to better immunological control of the disease. In a mouse model, both the bacterial burden and the inflammatory responses are attenuated in relapse[18] compared to the original infection.

The degree of morbidity associated with a relapse episode occurring in the trial is likely to be reduced compared to a relapse in the normal programme setting. This is because the relapse will be detected earlier - the trial will provide much closer post-treatment monitoring (monthly in person visits or phone calls for 2 years) than done in programmes (which usually discharge patients upon completion of treatment). Treatment failure/relapse is likely to be confirmed faster (because the trial provides for serial microbiology (smear and culture) testing as well as GeneXpert testing, and therefore treatment started faster thereby minimising morbidity compared to those who relapse in the programme setting. Relapses after short-course treatment in clinical trials generally happen early, within the first few months,[14,93] and are therefore likely to be picked up early when this post-treatment follow-up is at its most intense. Data that permit the comparison of the severity of relapses detected in a clinical trial with the severity of the relapses in a normal programme setting are very limited, even for the outcome of death. In the Rifaquin trial there was 1 TB death during the follow up period (out of 27 relapses, 4%), and in the ReMox trial there were 2 deaths (out of 122 relapses, 2%). In comparison, in one of the few reports of outcomes data from relapses in a programme setting, the death rate was reported as 9%.[94]

As a further protection of patients, we exclude patients from the trial if they have serious underlying chronic lung disease, as treatment failure or relapse in such patients may carry more harmful consequences, even if mild.

2. Drug resistance

A major determinant of the impact of treatment failure/relapse is whether patients can be successfully retreated (which in turn depends on the presence and detection of drug resistance and the availability of appropriate re-treatment regimens – with MDR-TB being the most important concern). The trial strategy is predicated on the assumption that treatment failure and relapses will remain drug sensitive, and all data from short course trials to date support this assumption. Patients who take treatment regularly and then stop treatment relapse with drug sensitive disease. This is consistent with the known biology of TB–organisms enter a dormant non-replicative state in the face of drug pressure and cannot develop new resistance in this state. The mechanism for developing resistance is based on the selection of pre-existing resistance mutations that may be present when there is a large untreated bacterial burden at the start of treatment, or when treatment adherence is erratic, allowing replenishment of the bacterial population.

Although there is a theoretical concern that bedaquiline and clofazimine, having long half-lives, may exert selection pressure for drug resistance during the tail period after treatment cessation when they remain effectively as monotherapy, this is not consistent with the known biology of resistance selection in TB. After 8 weeks' treatment the residual bacterial population will be very unlikely to

contain bedaquiline-resistant mutants (frequency of naturally-occurring resistance mutations to bedaquiline is between one in 10^7 to one in 10^8 organisms,[57,95] comparable to that of rifampicin and less frequent than resistance mutations to isoniazid).[96] Furthermore, the risk is also extremely low that *de novo* bedaquiline resistance will develop as levels of drug are likely to remain above the MIC for several months and this will prevent and residual bacterial growth. Resistance has not been seen with isoniazid monotherapy used during the continuation phase,[97] and it is no easier to generate new resistance mutations to bedaquiline than it is to generate them to isoniazid.[98] As further reassurance, monotherapy with bedaquiline has been given in several early bactericidal activity (EBA) studies in patients with very high bacterial burden without the development of resistance.[99,100] A similar argument can be made for clofazimine. Resistance to clofazimine is rare,[101,102-104] levels of clofazimine are likely to remain above the MIC for several weeks after the end of treatment, and it is very unlikely that resistance will arise during treatment or during the tail period.[105,106] This is further supported by an EBA study that found no resistance of organisms to clofazimine when administered alone for two weeks in TB patients.[107]

The trial includes additional measures to mitigate the harm arising from drug resistance. Firstly, the thorough resistance testing performed as part of the trial (including baseline and end of treatment strains, tested by conventional methods, as well as GeneXpert testing for rifampicin resistance and WGS sequencing where available) will ensure that drug susceptibilities are determined promptly and accurately, and an optimised re-treatment regimen can be rapidly selected for the patient. Secondly, such a re-treatment regimen, including any necessary second-line drugs and ancillary treatment and monitoring, including further DST for monitoring where needed, will be provided without cost to the patients in the trial to ensure optimal outcomes. Lastly, to reduce the overall risk, resistance data will be closely monitored by the IDMC, and recruitment will be discontinued to any arm where there is evidence that the strategy is promoting drug resistance.

In contrast, for patients managed in typical programme settings, the probability of treatment failure/resistance being accompanied by drug resistance is much greater. Firstly, because there is more limited adherence support available and people are taking drugs (partially or incompletely supervised) for longer, treatment failure/relapse episodes are commonly accompanied by drug resistance (prevalence of MDR-TB in re-treatment patients is typically in the range of 12% - 26% for high burden countries in Asia).[108] For patients who develop MDR-TB in the programme setting (either at the time of failing the initial treatment or when relapse occurs) then outcomes are particularly poor. Firstly because susceptibility testing may not be performed comprehensively, drug resistance may be detected late (if at all) with delay in instituting appropriate treatment. Secondly because, even if MDR-TB is detected, the appropriate treatment regimens may not be available given that they are complex and expensive (only a minority of MDR-TB patients receive treatment in resource-constrained programmes).

Thus the risk of drug-resistance-associated harm accompanying an episode of treatment failure/relapse for a patient on a TRUNCATE-TB management arm should be considerably less than the risk of such harm for a patient with treatment failure/relapse in a programme setting.

3. Risk of transmission to others
This risk depends on the infectiousness of the person with TB (usually judged by the smear status - positive or negative,[109] although there is probably a gradation of risk with higher levels of smear positivity)[110] and the proximity of their contacts. Although patients who relapse in the trial would potentially expose their household contacts to TB again, given that the relapse in trial patients is likely to be detected early (close follow up and use of GeneXpert for early diagnosis) and re-treatment started promptly before they become smear positive and more infectious, the risk to household contacts is likely to be small.

Nevertheless, in the event of treatment failure/relapse with smear positive disease, the trial team will arrange for contact tracing of the household contacts, appropriate testing, and provision of prophylaxis where appropriate. As part of ancillary care the trial will provide the necessary resources to re-screen contacts (using IGRA testing if locally available) and provide isoniazid prophylaxis (for 6 months) if indicated.

In contrast, for patients who have treatment failure/relapse in a typical programme setting, this is more often detected late, after the patient has become smear positive and after there has been considerable time for community transmission to occur (compounded by the higher risk of drug resistance being present and being transmitted).

Thus the risk of community transmission accompanying an episode of treatment failure/relapse for a patient on a TRUNCATE-TB management arm should be considerably less than the risk of such harm for a patient with treatment failure/relapse in a programme setting.

Overall risk arising from treatment failure/relapse

In summary, although patients in the TRUNCATE-TB management strategy in this trial may have a higher probability of experiencing episodes of treatment failure/relapse than patients managed outside of the trial, the impact of these episodes is mitigated by strong protections within the trial, such that the overall added risk to patients (and their contacts) is small. The overall risk compared to treatment outside the trial, although very difficult to estimate, is likely neutral. The trial will provide the necessary data to answer these important questions. The close monitoring by the IDMC and plans to stop treatment arms if these risks are outside the range anticipated allow these data to be gathered whilst protecting the patients.

12.5.2 Risk of drug toxicity and consequential harm in the TRUNCATE-TB management arms

Patients in the TRUNCATE-TB management arms will take boosted regimens which include modifications to the standard TB treatment regimen intended to increase sterilising potency (Section 2.5). These regimens were also constructed with the aim of avoiding combining drugs with known or predicted adverse pharmacokinetic interactions or likely to have overlapping toxicity. The toxicity profile of the drugs in these regimens is described in detail in Sections 2.5.

The main potential serious toxicities that are relevant to one or more of these boosted regimens and are important for the assessment of risk are as follows:

(i) Risk of significant cardiac arrhythmias (estimated < 1 in 100,000) arising from QTc prolongation with bedaquiline, levofloxacin or clofazimine. QTc prolongation in itself is not harmful, but carries a risk of Torsade de pointes, followed by arrhythmias. QTc prolongation with bedaquiline has not been associated with clinically significant arrhythmias.[111] The risk is mitigated in this trial by excluding patients with pre-existing cardiac disease, by not administering these drugs together by avoiding administration of concomitant medication with QTc-prolonging effects, and by performing ECG monitoring (with treatment modification if significant QTc prolongation occurs, see Section 6.3).

(ii) Possible risk of death associated with bedaquiline (unknown). An excess of deaths was observed in one trial of bedaquiline in MDR-TB (10 versus 2 deaths in placebo). There was no association between the deaths and bedaquiline plasma concentrations or a QTc of 500 or more during the trial.[62] The causes were diverse, there was no temporal relationship with bedaquiline, and the finding has not been borne out in subsequent studies. It is uncertain whether this risk is real, and the consensus in the field is that this

should not preclude further study of this drug for DS-TB or DR-TB. If there is a risk, it may be mitigated in the present trial by using a shorter duration of bedaquiline than used in MDR-TB treatment (2-3 months versus 6 months), and the IDMC will pay special attention to any deaths in the bedaquiline arm with a low threshold for terminating the arm if any unexplained deaths occur that are considered possibly related to bedaquiline.

(iii) Risk of severe haematological side effects from linezolid requiring cessation of drug (estimated 2.5% from clinical trial data).[24] The risk is mitigated by excluding patients with severe haematological suppression at baseline, frequent monitoring of the full blood count (FBC), and plans for dose reduction or discontinuation of therapy and the fact it is reversible within 2-4 weeks.

(iv) Risk of severe hepatitis from high dose rifampicin, estimated < 5 in 100 for grade 4 hepatotoxicity, based on trials that indicate no excess of severe hepatitis compared with standard dose rifampicin.[21,22,26] The risk is mitigated by excluding patients with preexisting chronic liver disease, alcohol abuse, raised liver function tests at baseline and by protocol-mandated dose-reduction or interruption if indicated.

In general, the trial is designed to minimise the impact of side-effects by close symptom monitoring, dose reduction or cessation of trial medication where needed, enhanced laboratory monitoring (consistent with safety recommendations for each of the study drugs) and regular ECG monitoring. The trial will provide the necessary resources to do additional investigations or follow-up tests for monitoring toxicities where these are required to minimise harms to the patient and will also provide the resources for the clinical management of drug-related toxicity. Sites will be provided with a Clinical Management Guide, trained in the clinical management protocols and will be able to consult a clinical management core comprising experts in TB management and the drugs used in the trial for advice on difficult clinical management problems (Section 6.5).

A further feature of the trial protocol to mitigate risks is the ongoing safety oversight provided by the IDMC (as described in Section 2.6 and 10.4). At interim analyses, the IDMC will consider inability to tolerate a regimen or identification of a serious side effect profile of a regimen (that jeopardises patient wellbeing, or that would represent a toxicity burden that would be impracticable for programmes to manage) as grounds for recommending termination of a boosted regimen (Section 10.4.2).

With all the measures taken to decrease the impact of drug toxicity, the overall risk of serious harm arising from drug toxicity from the boosted combinations is expected to be low.

Comparison of the risk of harm arising from drug toxicity for patients in the TRUNCATE-TB management arms with the risk of drug toxicity for patients receiving standard TB treatment in a programme setting is complex because of competing factors. Overall, because of use of additional drugs in the boosted regimens, each with the potential to add side effects and toxicity, the overall probability of drug-related toxicity is likely to be higher than that of standard treatment. However, this may be partially offset by the fact that the standard regimen is given for much longer, thus exposing the patient to more chronic side effects and risks of medication interactions than they would be exposed to in the TRUNCATE-TB managements arms. Furthermore, there is usually very limited clinical and laboratory monitoring of toxicity in programme settings and hence the impact in that setting may be greater with delay in detection and the implementation of prompt corrective action for toxicity that may result in more serious morbidity for the patients. Although hard to assess, overall risk of serious harm associated with the TRUNCATE-TB management arms is likely to be only modestly

greater than standard treatment in programme settings. The IDMC will monitor this aspect and provides a further safeguard if the overall risk is greater than predicted.

12.5.3 Burden of study visits and procedures in the TRUNCATE-TB management arms

Patients in the TRUNCATE-TB management arms will be required to receive treatment by directly observed therapy, as described in Section 6.4. This will be for the full duration of the initial treatment period (i.e. for 8 weeks in most, but longer in those who extend treatment for persistent symptoms, for treatment interruption or who switch to the standard treatment for toxicity and tolerability reasons).

DOT will be done by a healthcare worker in the treatment facility or the community. The trial will also attempt wherever possible, to tailor the supervision to the convenience of the patient – for example by allowing a responsible community member to perform the supervision if such a person can be identified. The burden of attending supervised therapy will be reduced by reimbursing patients for transport costs, time and inconvenience of attending these visits as appropriate for the transport costs and time involved, which is rarely possible in the programme setting. The overall burden of provision of DOT for the patient is also minimised because the majority of patients in the TRUNCATE-TB management arm will require DOT for 8 weeks only (as opposed to the usual 24 weeks).

The trial follow-up clinic visits are approximately every 2 weeks for the first 12 weeks, then monthly up to 6 months, then every 3 months thereafter. For patients who require retreatment, additional visits will be required for follow-up during and after treatment (although these additional visits will be consolidated, as far as possible, with the mandatory scheduled visits, as described in Section 7.2.3). Although the initial burden is relatively frequent and may require the patient to take time off work, the visits will generally be brief (lasting less than 30 minutes) and dedicated trial staff will ensure that the patients are seen rapidly and efficiently. Furthermore, patients will be reimbursed for all clinic visits in order to compensate for inconvenience and time off work. The TRUNCATE-TB management strategy requires close patient follow-up after treatment completion, but this is mainly achieved by telephone calls (lasting a few minutes) to keep the burden of visits to a minimum. Thus the burden of clinic visits for the trial is greater than that in the programme setting during the course of treatment, but steps are taken to minimise the impact of this on the patient.

The burden of investigations is substantial but these mainly involve sputum collection which requires minimal effort from the patient, and blood collection (mostly for safety monitoring while on treatment). Volumes of blood collection are modest, with tests kept to a minimum. These tests (and others required by the protocol, such as ECG and CXR) are provided free of charge by the trial. The burden of tests are also greater than required by the normal programme treatment, but whereas patients may be required to pay for routine tests in the programme, all the tests will be provided free of charge in the trial.

12.5.4 Benefits to patients in the TRUNCATE-TB management arms

The main benefit to patients in the TRUNCATE-TB management strategy is that they will have access to drugs and regimens that may have higher rates of sterilisation than the standard regimen, and thereby enable most patients to be cured after just 2 months of therapy, with associated socioeconomic and quality of life benefits.

Patients will also benefit generally from the additional resources devoted to their clinical care, which would be in excess of those if they received standard TB treatment outside the trial. The increased care and attention may improve motivation and adherence (critical for good outcomes). The close trial

follow up should decrease the risk of harms associated with treatment failure / relapse (as described above). The costs of the treatment of TB, the laboratory monitoring and ancillary care for the management of TB required by the protocol will be provided free of charge to the patient. This may also extend, on a discretionary basis, to provision of medical care for management of any complications of TB or managing the side effects of the drugs. Patients taking part in the trial will also receive drugs needed for the treatment of drug resistant TB, if this were to be detected in relapse / treatment failure.

12.6 RISKS FOR PATIENTS IN THE STANDARD TB MANAGEMENT ARM

The main risks to patients in the standard management strategy arm are those of inadequate treatment efficacy (manifesting as treatment failure or relapse) or toxicity of the drugs used for treatment. These risks are of a similar nature to those arising from standard therapy delivered outside the trial, but there are trial specific measures to minimise these risks for patients in the trial.

12.6.1 RISK OF TREATMENT FAILURE OR RELAPSE AND CONSEQUENT HARM

In the standard TB management strategy arm patients will take the standard regimen for the same duration as they would in the routine programme setting. The increased resources provided by the trial are likey to result in lower rates of treatment failure / relapse than if patients were to receive treatment in the programme setting. In typical programme settings, the cure rate is typically around 86% with a subsequent relapse rate of around 8%,[1,112] whereas in the standard treatment arm of contemporary clinical trials, the cure rate is 93-95%, with 2-7% relapse.[13-15] Thus we estimate that patients in this trial randomised to the standard TB management arm may have an 8-12% reduction in combined rate of treatment failure/relapse than patients receiving treatment in the local programme setting as a result of the added resources for treatment adherence and support.

Not only is the probability of treatment failure/relapse likely to be lower in the trial, but the impact of these episodes arising from clinical morbidity, from drug resistance and from transmission in the community is likely to be reduced compared to treatment failure / relapse occurring in the programme setting (see arguments in the risks of the TRUNCATE-TB management strategy above).

12.6.2 RISK OF DRUG TOXICITY AND CONSEQUENT HARM

The patients in the standard management arm will receive the same standard drugs as they would in the normal programme setting. Closer symptom and laboratory monitoring are likely to result in earlier detection of adverse drug reactions and therapy can be modified appropriately avoiding more serious toxicity. Thus the overall risk of serious harm arising from the TB drugs is also likely to be reduced in patients receiving standard treatment in the trial than it would be in the normal programme setting.

12.6.3 BURDEN OF STUDY VISITS AND PROCEDURES

Patients in the standard management arm will be required to receive treatment by DOT, as described in Section 6.4. This will be for at least the duration of the intensive phase (8 weeks) and may extend through the continuation phase (16 weeks) if this is the norm for treatment in the national programme. The burden of DOT, clinic visits for trial follow up and of tests required are all greater than that of standard management, however the trial will reimburse patients for their costs and for the inconvenience.

12.6.4 BENEFITS TO PATIENTS IN THE STANDARD TB MANAGEMENT ARM

Patients likely will have more resources devoted to their clinical care than they would if they were to received standard TB treatment outside the trial and this should result in overall better outcomes. It

should decrease the risk of harms as outlined above (reduce the risk of treatment failure / relapse and the associated morbidity, reduce the risk of drug resistance, reduce the risk of severe drug toxicity, reduce the risk of transmission to contacts) and reduce the burden of receiving such care (both in terms of the inconvenience of receiving DOT and the financial costs of treatment and ancillary care).

12.7 BENEFITS TO SOCIETY FROM CONDUCTING THIS TRIAL

Although the current TB management approach, unchanged in 40 years, works for the majority of patients, it is failing at a population level with only very slow decline in TB incidence and the spread of drug resistance. Persisting with the current approach will not solve the problem in any reasonable time frame. This trial may identify an innovative new strategy that could allow programmes to use their finite resources both to treat a greater number of patients and to treat those patients more effectively. By reducing the use of antibiotics and ensuring that resources are focused on supporting shorter courses of treatment, the strategy may also decrease the selection and spread of drug resistance in the community and thus mitigate another growing problem considered a public health emergency. The trial has the potential to decrease morbidity and mortality from TB with huge benefits to global health.

The trial will provide a wealth of new knowledge about the efficacy and safety of licensed drugs that have been re-purposed for use in TB (such as clofazimine, levofloxacin and linezolid) as well as make a major contribution to the existing body of knowledge from relatively small clinical trials that have supported the license of a new drug for TB (bedaquiline). The trial will provide valuable pharmacokinetic interaction data as well as experience with combining these drugs with standard TB drugs in novel combinations.

The trial will provide a wealth of ancillary scientific knowledge that may benefit society. In particular it will be the first trial to use a full MAMS design in TB (it has previously been applied only to cancer) and may thus change the way that TB clinical trials are done in the future, paving the way for faster development of new treatments for TB as well as more effective use of public research funds to increase new knowledge.

In summary, the benefit to society from conducting this research is enormous. If the trial shows the strategy to succeed it will have the potential to transform the way TB is treated globally as well as transform the way that TB trials are done in the future.

12.8 WEIGHING THE RISKS AGAINST THE BENEFITS TO THE PATIENT AND SOCIETY

In summary, the main risks to patients in the trial that have been described above – the excess of harm associated with treatment failure and relapse (clinical morbidity arising from TB, from drug resistance, and the risk of transmission to contacts) and the risk of drug toxicity. These are mitigated substantially by the trial design and additional resources provided by the trial to optimise monitoring and treatment. It is difficult to estimate the additional net risk to participants on the TRUNCATE-TB management strategy compared to those receiving standard treatment in the programme setting, but all factors considered it is unlikely that any excess risk will be very large. The magnitude of any excess risk is further limited by the close oversight of the trial by the IDMC that will recommend discontinuing recruitment to any arm where there appears to be a substantial excess risk. For patients in the trial on the standard TB management strategy, the risks are likely to be lower than those for patients treated in the programme setting.

In summary, the main benefits to the patients in the trial are the additional resources devoted to their clinical care and to support treatment adherence, which would be in excess of those if they received

standard TB treatment outside the trial. This is likely to improve overall outcomes. The most important benefit to patients in the TRUNCATE-TB management strategy (and the rationale for testing this approach) is that they will have access to drug regimens that may enable most to be cured after just 2 months of therapy, with major socioeconomic and quality of life benefits.

Overall, the trial has taken all possible measures to reduce the risks to patients and the resulting risks and benefits to patients of participating in this trial are well balanced. Taken together with the added potentially huge benefit to society of conducting this ground-breaking trial, the balance is strongly in favour of benefit. Thus the trial fulfils the ethical principle of beneficence.

12.9 ETHICAL APPROVALS

The protocol and the consent form will be reviewed and approved by an Ethics Committee designated by the UK sponsor, and an Independent Ethics Committee responsible for each study site. A signed and dated statement that the protocol and informed consent have been approved by the Ethics Committee must be given to the Chief Investigator and the coordinating centre before study initiation.

The investigator must sign a protocol signature page confirming his/her agreement to conduct the study in accordance with these documents (study instructions and procedures found in this protocol) and to provide access to all relevant data and records to field monitors, Quality Assurance representatives and designees from the coordinating centre, auditors, ethics committees and regulatory authorities as required. In the event where an inspection of the clinical site is requested by a regulatory authority, the investigator must inform coordinating centre immediately that this request has been made.

Investigators must adhere to the protocol and its procedures to avoid protocol deviations. The investigator shall not contact the Chief Investigator and/or the coordinating centre to request approval of a protocol deviation because there are no allowable authorized deviations. If the investigator feels a protocol deviation would improve the conduct of the study, this must be considered as a protocol amendment. Such an amendment must be agreed upon by the Chief Investigator and shall be approved by the Ethics Committee before implementation.

Any change or addition to the protocol can only be made in a written protocol amendment that must be approved by the Chief Investigator, regulatory authorities where required, and the Ethics Committees. Only amendments that are required for patient safety may be implemented prior to ethics committee approval. Although there is a need for approval of formal protocol amendment, the investigator is expected to take any immediate action required for the safety of any patient included in the study, even if this action represents a deviation from the protocol. In such cases, the Chief Investigator and the coordinating centre should be notified of this action and the Ethics Committee at the study site should be informed in accordance to the requirements stated in the Ethics Committee SOP, if applicable, to be reported as per local regulatory requirements.

13 REGULATORY APPROVALS AND COMPLIANCE

13.1 REGULATORY AUTHORITY APPROVALS

This protocol will be reviewed by/submitted to the national regulatory authority as appropriate in each country where the trial will be run.

The progress of the trial and safety issues will be reported to the regulatory authorities in accordance with local requirements and practices in a timely manner.

13.2 OTHER APPROVALS

The protocol will be submitted by those delegated to do so to the relevant department of each participating site or to other local departments for approval as required in each country. A copy of the local approval (as required) and Consent Form (CF) on local headed paper should be forwarded to the coordinating centre before patients are entered.

13.3 REGULATORY COMPLIANCE

The trial complies with the principles that are consistent with ICH Harmonised Tripartite Guidelines for Good Clinical Practice and the applicable laws and regulations.

13.3.1 SITE COMPLIANCE

The site will comply with the above. An agreement will be in place between the site and National University Hospital (Singapore), setting out respective roles and responsibilities (see Section 15-Finance).

The site will inform the coordinating centre as soon as they are aware of a possible serious breach of compliance, so that the coordinating centre can report this breach if necessary to regulatory authorities, ethics committees and the trial Sponsor. For the purposes of this regulation, a 'serious breach' is one that is likely to affect to a significant degree:

- The safety or physical or mental integrity of the patients in the trial, or
- The scientific value of the trial

13.3.2 SOURCE DATA AGREEMENT

The location of the information regarded as source data at each site will be described in a source-data agreement between the site and the sponsor. The CRF may be used as the source document for some or all of the data to be collected, provided that the specific arrangements are noted in the source data agreement and that this does not contravene applicable local regulations.

13.3.3 DATA COLLECTION & RETENTION

CRFs, clinical notes and administrative documentation should be kept in a secure location (for example, locked filing cabinets in a room with restricted access) and will be held for up to 15 years after the end of the trial, depending on sponsor and national regulatory requirements. During this period, all data should be accessible to the regulatory authorities and the Sponsor with suitable notice.

14 INDEMNITY

University College London holds insurance against claims from patients for injury caused by their participation in this clinical trial.

The local hospital has a duty of care to the participant of the clinical trial. University College London does not accept liability for any breach in the hospital's duty of care, or any negligence on the part of hospital employees. Sites participating in the trial should ensure that all staff working directly or indirectly on the trial have appropriate insurance to cover harm caused by negligence.

Patients who sustain injury and wish to make a claim for compensation should do so in writing in the first instance to the Chief Investigator.

15 FINANCE

The funders of the trial are the Wellcome Trust, UK Medical Research Council (MRC) and the UK Department for International Development (DFID) under the MRC/DFID Concordat agreement and the National Medical Research Council, Singapore.

This trial will be managed and coordinated by the National University Hospital (Singapore) in collaboration with Medical Research Council Clinical Trials Unit at UCL (MRC CTU at UCL).

Each participating site will be supported according to the submissions of their budgetary requirements.

Reimbursements will be made according to sub-agreements signed between the National University Hospital (Singapore) and the participating sites.

16 OVERSIGHT & TRIAL COMMITTEES

There are a number of committees involved with the oversight of the trial. These committees are detailed below.

The trial will be managed as a partnership between National University Hospital (Singapore) and the MRC Clinical Trials Unit at UCL (MRC CTU at UCL), supported by The International Union Against TB and Lung Disease (“The Union”) and a coordinating centre. The day to day activities will be managed from Singapore and the MRC CTU at UCL will maintain oversight. Data entry will be done at the sites on a web-based data entry system developed at the coordinating centre.

16.1 TRIAL MANAGEMENT GROUP (TMG)

A Trial Management Group (TMG) will be formed comprising the Chief Investigator, other lead investigators (clinical and non-clinical) and members of the MRC CTU at UCL. The TMG will be responsible for overseeing the day-to-day running and management of the trial, and will meet by teleconference at least monthly and face-to-face annually. There will be four operational cores comprising staff from the coordinating centre and relevant site personnel that will report to the TMG: a Project Management Core, a Laboratory Core, a Data Management Core and a Clinical Management Core.

16.2 TRIAL STEERING COMMITTEE (TSC)

The TSC has membership from the TMG plus independent members, including the Chair and representation from the community. The role of the TSC is to provide overall supervision for the trial and provide advice through its independent Chair. The ultimate decision for the continuation of the trial lies with the TSC. Further details of TSC functioning are presented in the TSC Charter.

16.3 INDEPENDENT DATA MONITORING COMMITTEE (IDMC)

An Independent Data Monitoring Committee (IDMC) will be formed of clinical trials research-experienced clinicians, and a statistician, none of whom have direct involvement with the study. The IDMC will report to the TSC.

The primary responsibility of the IDMC will be to oversee the safety of the trial patients. The IDMC will be the only group who sees the confidential, accumulating data for the trial. Reports to the IDMC will be produced by the trial statisticians. The IDMC will meet within 6 months of the trial opening; the IDMC will meet every 6 months during the course of the trial and at other specified fixed and *ad hoc* meetings as required (Section 10.4.1). The IDMC will consider data using the SAP (see Section 10.5) and consider evidence emerging from other studies, and will advise the TSC. The IDMC will be able to recommend discontinuing recruitment to an arm or other modifications (see Section 10.4.5)

Further details of IDMC functioning, and the procedures for interim analysis and monitoring are provided in the IDMC Charter.

17 PUBLICATION

The key study design elements of this protocol will be posted in a publicly accessible database such as clinicaltrials.gov.

It is anticipated that a number of opportunities will arise for publication during the course of, and following completion of the TRUNCATE-TB trial. In order to avoid disputes regarding authorship, it is important to establish a consensus approach that will provide a framework for all publications derived in full or in part from this clinical trial. The details of this approach are described in a publication policy for the trial. The main elements are outlined here.

No analyses by randomised group of any outcome (primary, secondary or other) will be conducted before the end of the trial, other than those for interim review by the IDMC. Outcome data by treatment arm will not be revealed to the participating investigators until the data collection phase and primary full analysis of the trial has been completed. This policy safeguards against possible bias affecting the data collection.

All abstracts and manuscripts must be approved by the TMG and TSC before submission for presentation or publication. Any presentation or publication arising before the end of the trial (not by randomised groups) must also be approved by the IDMC in order to ensure that the primary objective of the trial (the randomised comparison) is not compromised. This is true whether the publication or presentation is concerned directly with the results of the trial or is associated with the trial in some other way. However, although individual participating investigators will not have any inherent right to perform analyses or interpretations or to make public presentations or seek publication of any of the data other than under the auspices of and with the approval of the TMG and TSC (and the IDMC before the end of the trial), they will be encouraged to develop sub-studies or propose analyses subject to the approval by the TMG and TSC (and the IDMC before the end of the trial).

A writing committee will be formed to develop each approved abstract or manuscript and the members of the writing group will usually be named as authors on the publication. All headline authors in any publication arising must have a made a significant academic or project management contribution to the work that is being presented. In addition to fulfilling the criteria based on contribution, additional features that will be considered in selecting an authorship group will include the recruitment of patients who contributed data to any set of analyses contained in the manuscript, and /or the conduct of analyses (laboratory and statistical), leadership and coordination of the project in the absence of a clear academic contribution.

To allow recognition of a larger number of contributors than is possible by individual names on a manuscript, all publications will include, as an author, “The TRUNCATE-TB Trial Team” and the list of all those who have contributed to the Team will be included as an appendix to the publication.

REFERENCES

1. WHO. Global tuberculosis report 2014. Geneva: World Health Organization; 2014
2. Laurence YV, Griffiths UK, Vassall A. Costs to Health Services and the Patient of Treating Tuberculosis: A Systematic Literature Review. *PharmacoEconomics* 2015.
3. Treatment of Tuberculosis: Guidelines, 4th edition. Geneva: World Health Organization; 2010. 4th ed.
4. Reichman LB. Unsexy tuberculosis. *Lancet* 2009; **373**(9657): 28.
5. Panjabi R, Comstock GW, Golub JE. Recurrent tuberculosis and its risk factors: adequately treated patients are still at high risk. *The international journal of tuberculosis and lung disease : the official journal of the International Union against Tuberculosis and Lung Disease* 2007; **11**(8): 828-37.
6. Hung CL, Chien JY, Ou CY. Associated factors for tuberculosis recurrence in taiwan: a nationwide nested case-control study from 1998 to 2010. *PloS one* 2015; **10**(5): e0124822.
7. The Barcelona Declaration. 2014. http://www.globaltbcaucus.org/.
8. Shrestha S, Knight GM, Fofana M, et al. Drivers and trajectories of resistance to new first-line drug regimens for tuberculosis. *Open forum infectious diseases* 2014; **1**(2): ofu073.
9. Udwadia ZF. MDR, XDR, TDR tuberculosis: ominous progression. *Thorax* 2012; **67**(4): 286-8.
10. Fox W. Whither short-course chemotherapy? *British journal of diseases of the chest* 1981; **75**(4): 331-57.
11. Fox W, Ellard GA, Mitchison DA. Studies on the treatment of tuberculosis undertaken by the British Medical Research Council tuberculosis units, 1946-1986, with relevant subsequent publications. *The international journal of tuberculosis and lung disease : the official journal of the International Union against Tuberculosis and Lung Disease* 1999; **3**(10 Suppl 2): S231-79.
12. Gelband H. Regimens of less than six months for treating tuberculosis. *The Cochrane database of systematic reviews* 2000; (2): Cd001362.
13. Gillespie SH, Crook AM, McHugh TD, et al. Four-month moxifloxacin-based regimens for drug-sensitive tuberculosis. *The New England journal of medicine* 2014; **371**(17): 1577-87.
14. Jindani A, Harrison TS, Nunn AJ, et al. High-dose rifapentine with moxifloxacin for pulmonary tuberculosis. *The New England journal of medicine* 2014; **371**(17): 1599-608.
15. Merle CS, Fielding K, Sow OB, et al. A four-month gatifloxacin-containing regimen for treating tuberculosis. *The New England journal of medicine* 2014; **371**(17): 1588-98.
16. Johnson JL, Hadad DJ, Dietze R, et al. Shortening treatment in adults with noncavitary tuberculosis and 2-month culture conversion. *American journal of respiratory and critical care medicine* 2009; **180**(6): 558-63.
17. Sputum-smear-negative pulmonary tuberculosis: controlled trial of 3-month and 2-month regimens of chemotherapy. *Lancet* 1979; **1**(8131): 1361-3.
18. de Steenwinkel JE, de Knegt GJ, ten Kate MT, et al. Relapse of tuberculosis versus primary tuberculosis; course, pathogenesis and therapy in mice. *Tuberculosis* 2013; **93**(2): 213-21.
19. Verrall AJ, Netea MG, Alisjahbana B, Hill PC, van Crevel R. Early clearance of Mycobacterium tuberculosis: a new frontier in prevention. *Immunology* 2014; **141**(4): 506-13.
20. Steingart KR, Jotblad S, Robsky K, et al. Higher-dose rifampin for the treatment of pulmonary tuberculosis: a systematic review. *The international journal of tuberculosis and lung disease : the official journal of the International Union against Tuberculosis and Lung Disease* 2011; **15**(3): 305-16.
21. Boeree MJ, Diacon AH, Dawson R, et al. A dose-ranging trial to optimize the dose of rifampin in the treatment of tuberculosis. *American journal of respiratory and critical care medicine* 2015; **191**(9): 1058-65.
22. Boeree MJ, Hoelscher M (on behalf of the PanACEA Consortium). High-Dose Rifampin, SQ109 and Moxifloxacin for Treating TB: The PanACEA MAMS-TB Trial. Conference on Retroviruses and Opportunistic Infections. . Seattle; 2015.

23. Zhang M, Sala C, Dhar N, et al. In vitro and in vivo activities of three oxazolidinones against nonreplicating Mycobacterium tuberculosis. *Antimicrobial agents and chemotherapy* 2014; **58**(6): 3217-23.
24. Lee M, Lee J, Carroll MW, et al. Linezolid for treatment of chronic extensively drug-resistant tuberculosis. *The New England journal of medicine* 2012; **367**(16): 1508-18.
25. https://www.clinicaltrials.gov/ct2/show/NCT01994460.
26. Boeree M. High-dose rifampicin: a phase II trial comparing 10, 15 and 20mg/kg rifampicin for two months. 45th Union World Conference 2014; Barcelona, Spain; 2014.
27. Zhang X, Falagas ME, Vardakas KZ, et al. Systematic review and meta-analysis of the efficacy and safety of therapy with linezolid containing regimens in the treatment of multidrug-resistant and extensively drug-resistant tuberculosis. *Journal of thoracic disease* 2015; **7**(4): 603-15.
28. Nguyen S, Pasquet A, Legout L, et al. Efficacy and tolerance of rifampicin-linezolid compared with rifampicin-cotrimoxazole combinations in prolonged oral therapy for bone and joint infections. *Clinical microbiology and infection : the official publication of the European Society of Clinical Microbiology and Infectious Diseases* 2009; **15**(12): 1163-9.
29. Xu J, Lu Y, Fu L, et al. In vitro and in vivo activity of clofazimine against Mycobacterium tuberculosis persisters. *The international journal of tuberculosis and lung disease : the official journal of the International Union against Tuberculosis and Lung Disease* 2012; **16**(8): 1119-25.
30. Aung KJ, Van Deun A, Declercq E, et al. Successful '9-month Bangladesh regimen' for multidrug-resistant tuberculosis among over 500 consecutive patients. *The international journal of tuberculosis and lung disease : the official journal of the International Union against Tuberculosis and Lung Disease* 2014; **18**(10): 1180-7.
31. Tang S, Yao L, Hao X, et al. Clofazimine for the treatment of multidrug-resistant tuberculosis: prospective, multicenter, randomized controlled study in China. *Clinical Infectious Diseases* 2015.
32. Holdiness MR. Clinical pharmacokinetics of clofazimine. A review. *Clinical pharmacokinetics* 1989; **16**(2): 74-85.
33. Bulatovic VM, Wengenack NL, Uhl JR, et al. Oxidative Stress Increases Susceptibility of Mycobacterium tuberculosis to Isoniazid. *Antimicrobial agents and chemotherapy* 2002; **46**(9): 2765-71.
34. Tyagi S, Ammerman NC, Li SY, et al. Clofazimine shortens the duration of the first-line treatment regimen for experimental chemotherapy of tuberculosis. *Proceedings of the National Academy of Sciences of the United States of America* 2015; **112**(3): 869-74.
35. Van Deun A, Maug AK, Salim MA, et al. Short, highly effective, and inexpensive standardized treatment of multidrug-resistant tuberculosis. *American journal of respiratory and critical care medicine* 2010; **182**(5): 684-92.
36. Dey T, Brigden G, Cox H, Shubber Z, Cooke G, Ford N. Outcomes of clofazimine for the treatment of drug-resistant tuberculosis: a systematic review and meta-analysis. *The Journal of antimicrobial chemotherapy* 2013; **68**(2): 284-93.
37. World Health Organization. Multidrug Therapy Against Leprosy: Development and Implementation Over the Past 25 Years Geneva 2004.
38. McNeeley D. Elucidating the Role of Clofazimine in the Treatment of MDR-TB. MDR-TB Landscape Meeting. 2014.
39. Hwang TJ, Dotsenko S, Jafarov A, et al. Safety and availability of clofazimine in the treatment of multidrug and extensively drug-resistant tuberculosis: analysis of published guidance and meta-analysis of cohort studies. *BMJ open* 2014; **4**(1): e004143.
40. Rosenthal IM, Zhang M, Williams KN, et al. Daily dosing of rifapentine cures tuberculosis in three months or less in the murine model. *PLoS medicine* 2007; **4**(12): e344.
41. Rosenthal IM, Tasneen R, Peloquin CA, et al. Dose-ranging comparison of rifampin and rifapentine in two pathologically distinct murine models of tuberculosis. *Antimicrobial agents and chemotherapy* 2012; **56**(8): 4331-40.

42. Dorman SE, Goldberg S, Stout JE, et al. Substitution of rifapentine for rifampin during intensive phase treatment of pulmonary tuberculosis: study 29 of the tuberculosis trials consortium. *The Journal of infectious diseases* 2012; **206**(7): 1030-40.
43. Savic R. PK-PD analysis of rifapentine in patients during intensive phase treatment for tuberculosis from Tuberculosis Trial Consortium Studies 29 and 29X. 6th International Workshop on Clinical Pharmacology of TB Drugs; 2013; 2013.
44. Savic RM, Lu Y, Bliven-Sizemore E, et al. Population pharmacokinetics of rifapentine and desacetyl rifapentine in healthy volunteers: nonlinearities in clearance and bioavailability. *Antimicrobial agents and chemotherapy* 2014; **58**(6): 3035-42.
45. Mitchison DA. The action of antituberculosis drugs in short-course chemotherapy. *Tubercle* 1985; **66**(3): 219-25.
46. Hu Y, Coates AR, Mitchison DA. Sterilizing activities of fluoroquinolones against rifampin-tolerant populations of Mycobacterium tuberculosis. *Antimicrobial agents and chemotherapy* 2003; **47**(2): 653-7.
47. Rodriguez JC, Ruiz M, Lopez M, Royo G. In vitro activity of moxifloxacin, levofloxacin, gatifloxacin and linezolid against Mycobacterium tuberculosis. *International journal of antimicrobial agents* 2002; **20**(6): 464-7.
48. Cremades R, Rodriguez JC, Garcia-Pachon E, et al. Comparison of the bactericidal activity of various fluoroquinolones against Mycobacterium tuberculosis in an in vitro experimental model. *The Journal of antimicrobial chemotherapy* 2011; **66**(10): 2281-3.
49. Ji B, Lounis N, Truffot-Pernot C, Grosset J. In vitro and in vivo activities of levofloxacin against Mycobacterium tuberculosis. *Antimicrobial agents and chemotherapy* 1995; **39**(6): 1341-4.
50. Conde MB, C CS, M D, Dorman SE. A phase II trial of a rifapentine plus moxifloxacin-based regimen for pulmonary TB treatment. . 21st Conference on Retroviruses and Opportunistic Infections Boston 2014.
51. Koh WJ, Lee SH, Kang YA, et al. Comparison of levofloxacin versus moxifloxacin for multidrug-resistant tuberculosis. *American journal of respiratory and critical care medicine* 2013; **188**(7): 858-64.
52. Chang KC, Leung CC, Yew WW, et al. Pyrazinamide may improve fluoroquinolone-based treatment of multidrug-resistant tuberculosis. *Antimicrobial agents and chemotherapy* 2012; **56**(11): 5465-75.
53. Rodriguez Diaz JC, Ruiz M, Lopez M, Royo G. Synergic activity of fluoroquinolones and linezolid against Mycobacterium tuberculosis. *International journal of antimicrobial agents* 2003; **21**(4): 354-6.
54. Dawson R, Narunsky K, Carman D, et al. Two-stage activity-safety study of daily rifapentine during intensive phase treatment of pulmonary tuberculosis. *The international journal of tuberculosis and lung disease : the official journal of the International Union against Tuberculosis and Lung Disease* 2015; **19**(7): 780-6.
55. Stahlmann R, Lode HM. Risks associated with the therapeutic use of fluoroquinolones. *Expert opinion on drug safety* 2013; **12**(4): 497-505.
56. Svensson EM, Murray S, Karlsson MO, Dooley KE. Rifampicin and rifapentine significantly reduce concentrations of bedaquiline, a new anti-TB drug. *The Journal of antimicrobial chemotherapy* 2015; **70**(4): 1106-14.
57. Andries K, Verhasselt P, Guillemont J, et al. A diarylquinoline drug active on the ATP synthase of Mycobacterium tuberculosis. *Science* 2005; **307**(5707): 223-7.
58. Shi R, Sugawara I. Development of New Anti-tuberculosis Drug Candidates. *The Tohoku Journal of Experimental Medicine* 2010; **221**(2): 97-106.
59. Koul A, Vranckx L, Dendouga N, et al. Diarylquinolines are bactericidal for dormant mycobacteria as a result of disturbed ATP homeostasis. *The Journal of biological chemistry* 2008; **283**(37): 25273-80.

60. Tasneen R, Li SY, Peloquin CA, et al. Sterilizing activity of novel TMC207- and PA-824-containing regimens in a murine model of tuberculosis. *Antimicrobial agents and chemotherapy* 2011; **55**(12): 5485-92.
61. Diacon AH PA, Grobusch M, Patientia R, et al. . . The diarylquinoline TMC207 for multidrug resistant tuberculosis. *The New England journal of medicine* 2009 **Jun 4;360**(23): 2397-405.
62. Diacon AH, Pym A, Grobusch MP, et al. Multidrug-resistant tuberculosis and culture conversion with bedaquiline. *The New England journal of medicine* 2014; **371**(8): 723-32.
63. Mitchison DA. Role of individual drugs in the chemotherapy of tuberculosis. *The international journal of tuberculosis and lung disease : the official journal of the International Union against Tuberculosis and Lung Disease* 2000; **4**(9): 796-806.
64. Controlled clinical trial of four short-course (6-month) regimens of chemotherapy for treatment of pulmonary tuberculosis. Third report. East African-British Medical Research Councils. *Lancet* 1974; **2**(7875): 237-40.
65. van Heeswijk RP, Dannemann B, Hoetelmans RM. Bedaquiline: a review of human pharmacokinetics and drug-drug interactions. *The Journal of antimicrobial chemotherapy* 2014; **69**(9): 2310-8.
66. Ibrahim M, Andries K, Lounis N, et al. Synergistic activity of R207910 combined with pyrazinamide against murine tuberculosis. *Antimicrobial agents and chemotherapy* 2007; **51**(3): 1011-5.
67. Guglielmetti L, Le Du D, Jachym M, et al. Compassionate use of bedaquiline for the treatment of multidrug-resistant and extensively drug-resistant tuberculosis: interim analysis of a French cohort. *Clinical infectious diseases : an official publication of the Infectious Diseases Society of America* 2015; **60**(2): 188-94.
68. http://www.who.int/tb/challenges/mdr/Package_insert_bedaquiline.pdf.
69. Ndjeka N, Conradie F, Schnippel K, et al. Treatment of drug-resistant tuberculosis with bedaquiline in a high HIV prevalence setting: an interim cohort analysis. *The international journal of tuberculosis and lung disease : the official journal of the International Union against Tuberculosis and Lung Disease* 2015; **19**(8): 979-85.
70. Sydes MR, Parmar MK, James ND, et al. Issues in applying multi-arm multi-stage methodology to a clinical trial in prostate cancer: the MRC STAMPEDE trial. *Trials* 2009; **10**: 39.
71. Phillips PP, Gillespie SH, Boeree M, et al. Innovative trial designs are practical solutions for improving the treatment of tuberculosis. *The Journal of infectious diseases* 2012; **205 Suppl 2**: S250-7.
72. Royston P, Parmar MK, Qian W. Novel designs for multi-arm clinical trials with survival outcomes with an application in ovarian cancer. *Statistics in medicine* 2003; **22**(14): 2239-56.
73. Naidoo K, Baxter C, Abdool Karim SS. When to start antiretroviral therapy during tuberculosis treatment? *Current opinion in infectious diseases* 2013; **26**(1): 35-42.
74. Bothamley G. Drug treatment for tuberculosis during pregnancy: safety considerations. *Drug safety : an international journal of medical toxicology and drug experience* 2001; **24**(7): 553-65.
75. Wu AW, Revicki DA, Jacobson D, Malitz FE. Evidence for reliability, validity and usefulness of the Medical Outcomes Study HIV Health Survey (MOS-HIV). *Quality of life research : an international journal of quality of life aspects of treatment, care and rehabilitation* 1997; **6**(6): 481-93.
76. Babikako HM, Neuhauser D, Katamba A, Mupere E. Feasibility, reliability and validity of health-related quality of life questionnaire among adult pulmonary tuberculosis patients in urban Uganda: cross-sectional study. *Health and quality of life outcomes* 2010; **8**: 93.
77. http://go.worldbank.org/WKOXNZV3X0.
78. http://www.dhsprogram.com/.
79. Ralph AP, Ardian M, Wiguna A, et al. A simple, valid, numerical score for grading chest x-ray severity in adult smear-positive pulmonary tuberculosis. *Thorax* 2010; **65**(10): 863-9.
80. Nahid P, Saukkonen J, Mac Kenzie WR, et al. CDC/NIH Workshop. Tuberculosis biomarker and surrogate endpoint research roadmap. *American journal of respiratory and critical care medicine* 2011; **184**(8): 972-9.

81. Bryant JM, Harris SR, Parkhill J, et al. Whole-genome sequencing to establish relapse or re-infection with Mycobacterium tuberculosis: a retrospective observational study. *The Lancet Respiratory medicine* 2013; **1**(10): 786-92.
82. Vestbo J, Hurd SS, Agusti AG, et al. Global strategy for the diagnosis, management, and prevention of chronic obstructive pulmonary disease: GOLD executive summary. *American journal of respiratory and critical care medicine* 2013; **187**(4): 347-65.
83. Tiemersma EW, van der Werf MJ, Borgdorff MW, Williams BG, Nagelkerke NJ. Natural history of tuberculosis: duration and fatality of untreated pulmonary tuberculosis in HIV negative patients: a systematic review. *PloS one* 2011; **6**(4): e17601.
84. FDA. http://www.fda.gov/downloads/Drugs/Guidances/UCM202140.pdf.
85. https://clinicaltrials.gov/ct2/show/NCT02342886.
86. Ioannidis JP, Evans SJ, Gotzsche PC, et al. Better reporting of harms in randomized trials: an extension of the CONSORT statement. *Ann Intern Med* 2004; **141**(10): 781-8.
87. Aarons L, Ogungbenro K. Optimal design of pharmacokinetic studies. *Basic & clinical pharmacology & toxicology* 2010; **106**(3): 250-5.
88. Protection of human subjects; Belmont Report: notice of report for public comment. *Federal register* 1979; **44**(76): 23191-7.
89. Ananworanich J, Gayet-Ageron A, Le Braz M, et al. CD4-guided scheduled treatment interruptions compared with continuous therapy for patients infected with HIV-1: results of the Staccato randomised trial. *Lancet* 2006; **368**(9534): 459-65.
90. El-Sadr WM, Lundgren J, Neaton JD, et al. CD4+ count-guided interruption of antiretroviral treatment. *The New England journal of medicine* 2006; **355**(22): 2283-96.
91. Shi J, Pabon K, Scotto KW. Methylxanthines Increase Expression of the Splicing Factor SRSF2 by Regulating Multiple Post-transcriptional Mechanisms. *The Journal of biological chemistry* 2015; **290**(24): 14986-5003.
92. Paton NI, Stöhr W, Arenas-Pinto A, et al. Protease inhibitor monotherapy for long-term management of HIV infection: a randomised, controlled, open-label, non-inferiority trial. *The Lancet HIV*.
93. Nunn AJ, Phillips PP, Mitchison DA. Timing of relapse in short-course chemotherapy trials for tuberculosis. *The international journal of tuberculosis and lung disease : the official journal of the International Union against Tuberculosis and Lung Disease* 2010; **14**(2): 241-2.
94. Dolma KG, Adhikari L, Dadul P, Laden T, Singhi L, Mahanta J. A study on the assessment of retreatment tuberculosis patients attending the DOTS centre in Sikkim, India from 2002-2010. 2013.
95. Leibert E, Danckers M, Rom WN. New drugs to treat multidrug-resistant tuberculosis: the case for bedaquiline. *Therapeutics and clinical risk management* 2014; **10**: 597-602.
96. David HL. Probability distribution of drug-resistant mutants in unselected populations of Mycobacterium tuberculosis. *Applied microbiology* 1970; **20**(5): 810-4.
97. Controlled clinical trial of two 6-month regimens of chemotherapy in the treatment of pulmonary tuberculosis. Tanzania/British Medical Research Council Study. *The American review of respiratory disease* 1985; **131**(5): 727-31.
98. McGrath M, Gey van Pittius NC, van Helden PD, Warren RM, Warner DF. Mutation rate and the emergence of drug resistance in Mycobacterium tuberculosis. *The Journal of antimicrobial chemotherapy* 2014; **69**(2): 292-302.
99. Rustomjee R, Diacon AH, Allen J, et al. Early bactericidal activity and pharmacokinetics of the diarylquinoline TMC207 in treatment of pulmonary tuberculosis. *Antimicrobial agents and chemotherapy* 2008; **52**(8): 2831-5.
100. Diacon AH, Dawson R, Von Groote-Bidlingmaier F, et al. Randomized dose-ranging study of the 14-day early bactericidal activity of bedaquiline (TMC207) in patients with sputum microscopy smear-positive pulmonary tuberculosis. *Antimicrobial agents and chemotherapy* 2013; **57**(5): 2199-203.

101. Gopal M, Padayatchi N, Metcalfe JZ, O'Donnell MR. Systematic review of clofazimine for the treatment of drug-resistant tuberculosis. *The international journal of tuberculosis and lung disease : the official journal of the International Union against Tuberculosis and Lung Disease* 2013; **17**(8): 1001-7.
102. David HL, Rastogi N, Clavel-Seres S, Clement F. Studies on clofazimine-resistance in mycobacteria: is the inability to isolate drug-resistance mutants related to its mode of action? *Zentralblatt fur Bakteriologie, Mikrobiologie, und Hygiene Series A, Medical microbiology, infectious diseases, virology, parasitology* 1987; **266**(1-2): 292-304.
103. Schon T, Jureen P, Chryssanthou E, et al. Wild-type distributions of seven oral second-line drugs against Mycobacterium tuberculosis. *The international journal of tuberculosis and lung disease : the official journal of the International Union against Tuberculosis and Lung Disease* 2011; **15**(4): 502-9.
104. van Ingen J, Simons S, de Zwaan R, et al. Comparative study on genotypic and phenotypic second-line drug resistance testing of Mycobacterium tuberculosis complex isolates. *Journal of clinical microbiology* 2010; **48**(8): 2749-53.
105. Rastogi N, Ross BC, Dwyer B, et al. Emergence during unsuccessful chemotherapy of multiple drug resistance in a strain of Mycobacterium tuberculosis. *European journal of clinical microbiology & infectious diseases : official publication of the European Society of Clinical Microbiology* 1992; **11**(10): 901-7.
106. Reddy VM, O'Sullivan JF, Gangadharam PR. Antimycobacterial activities of riminophenazines. *The Journal of antimicrobial chemotherapy* 1999; **43**(5): 615-23.
107. Diacon AH, Dawson R, von Groote-Bidlingmaier F, et al. Bactericidal activity of pyrazinamide and clofazimine alone and in combinations with pretomanid and bedaquiline. *American journal of respiratory and critical care medicine* 2015; **191**(8): 943-53.
108. World Health Organisation. Global tuberculosis report 2015.
109. World Health Organization. Recommendations for investigating contacts of persons with infectious tuberculosis in low-and middle-income countries: World Health Organization; 2012.
110. Lohmann EM, Koster BF, le Cessie S, Kamst-van Agterveld MP, van Soolingen D, Arend SM. Grading of a positive sputum smear and the risk of Mycobacterium tuberculosis transmission. *The international journal of tuberculosis and lung disease : the official journal of the International Union against Tuberculosis and Lung Disease* 2012; **16**(11): 1477-84.
111. Harausz E, Cox H, Rich M, Mitnick CD, Zimetbaum P, Furin J. QTc prolongation and treatment of multidrug-resistant tuberculosis. *The international journal of tuberculosis and lung disease : the official journal of the International Union against Tuberculosis and Lung Disease* 2015; **19**(4): 385-91.
112. Azhar GS. DOTS for TB relapse in India: A systematic review. *Lung India : official organ of Indian Chest Society* 2012; **29**(2): 147-53.

APPENDIX 1: QUANTIFICATION OF SIZE OF PLEURAL EFFUSIONS

Grade of effusion	Characteristic of chest X-ray
0	No pleural fluid present
1	Blunting of the costophrenic angle
2	More than blunting of the costophrenic angle but less than 25% of hemithorax occupied by pleural fluid
3	Pleural fluid occupying 25% to 50% of hemithorax
4	Pleural fluid occupying 50% to 75% of hemithorax
5	Pleural fluid occupying more than 75% of hemithorax

Reference: Light RW, Rogers JT, Cheng D-s, Rodriguez RM. Large Pleural Effusions Occurring after Coronary Artery Bypass Grafting. Annals of Internal Medicine. 1999; 130(11): 891-6.

APPENDIX 2: IUATLD SMEAR MICROSCOPY GRADING SCALE

Finding	Recording
No AFB in at least 100 fields	Negative
1 to 9 AFB in 100 fields	Exact no./100 fields
10 to 99 AFB in 100 fields	1+
1 to 10 AFB per field in at least 50 fields	2+
>10 AFB per field in at least 20 fields	3+

Reference: International Union Against Tuberculosis and Lung Disease. Technical Guide: Sputum Examination for Tuberculosis by Direct Microscopy in Low-Income Countries. 2000.

APPENDIX 3: MRC BREATHLESSNESS SCALE

Grade	Degree of breathlessness related to activities
1	Not troubled by breathlessness except on strenuous exercise
2	Short of breath when hurrying on the level or walking up a slight hill
3	Walks slower than most people on the level, stops after a mile or so, or stops after 15 minutes walking at own pace
4	Stops for breath after walking about 100 yds or after a few minutes on level ground
5	Too breathless to leave the house, or breathless when undressing

Reference: Stenton C. The MRC breathlessness scale. Occupational medicine (Oxford, England). 2008; 58(3): 226-7.

APPENDIX 4: MEASUREMENT OF QT INTERVAL

The QT interval should be measured in lead II of the ECG since it is typically the longest QT interval.

For the purposes of the trial, we will use Fridericia's formulae for calculation of the corrected QT.

$$QTc = \frac{QT}{(RR)^{0.33}}$$

QTc = corrected QT interval

QT = Q wave to end of T wave

RR = time from two consecutive R waves

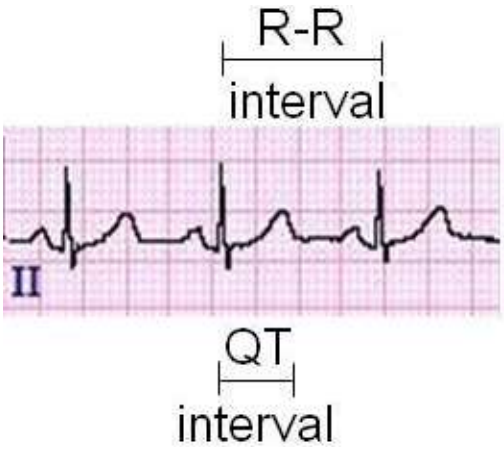

Reference: Postema PG, Wilde AAM. The Measurement of the QT Interval. *Current Cardiology Reviews* 2014; 10(3): 287-94.

APPENDIX 5: CREATININE CLEARANCE CALCULATION

1) Ensure that the creatinine is expressed in µmol/L

-if your lab reports the creatinine in mg/dL: multiple by 88.4

-if your lab reports the creatinine value in µmol/L: use this value

2) Calculate the creatinine clearance using the Cockcroft-Gault formula

$$\text{Creatinine Clearance} = \left[\frac{(140\text{-age}) \times \text{weight (kg)} \times \text{Constant}}{\text{Creatinine } (\mu\text{mol/L})} \right]$$

Where Constant is 1.23 for men and 1.04 for women

Reference: Cockcroft DW, Gault MH. Prediction of creatinine clearance from serum creatinine. *Nephron* 1976; 16(1): 31-41.

APPENDIX 6: PATIENT ACCEPTABILITY QUESTIONNAIRE

1. How much **difficulty** did the following cause you?

1a. Swallowing a large number of pills each day

1 none ☐ 2 a little ☐ 3 some ☐ 4 a lot ☐

1b. Coming for visits after the treatment was finished

1 none ☐ 2 a little ☐ 3 some ☐ 4 a lot ☐

2. How much **anxiety** did the following cause you?

2a. The risk that you might experience serious treatment side effects (whether or not you actually experienced them)

1 none ☐ 2 a little ☐ 3 some ☐ 4 a lot ☐

2b. The risk that the TB might come back again (whether or not it did so)

1 none ☐ 2 a little ☐ 3 some ☐ 4 a lot ☐

2c. The risk that you might infect others (whether or not you did so)

1 none ☐ 2 a little ☐ 3 some ☐ 4 a lot ☐

3. How much did it increase your motivation to take treatment when we told you at the beginning of the trial which treatment length you would be given (2 months or 6 months)?

1 none ☐ 2 a little ☐ 3 some ☐ 4 a lot ☐

4. If a friend had TB and was given a choice between starting with the short treatment (2 months) or the standard 6 month treatment, based on your experience which would you recommend they choose?

- 2m treatment ☐
- 6 month treatment ☐
- No preference/unsure ☐

APPENDIX 7: LIST OF SITES AND INVESTIGATORS

The following list of sites and investigators contains information that is correct at the date of this version of the protocol was finalised and will be updated in the protocol at the time of any subsequent amendments to the main body of the protocol. This information is likely to change in the intervening period as new sites are added and new investigators identified or investigators change contact details. An up-to-date list of sites and investigators is available from the trial coordinating centre during this time.

BANDUNG, INDONESIA UNIVERSITAS PADJADJARAN	Prof Rovina Ruslami (PI)<br>Jl. Prof. Eyckman No. 38, Bandung 40161 Indonesia	Email: n.ruslami@gmail.com<br>Tel: +62 222 038218<br>Fax: +62 222 033915
JAKARTA, INDONESIA, PERSAHABATAN GENERAL HOSPITAL	Dr Erlina Burhan (PI)<br>Jl. Persahabatan Raya No. 1 Jakarta Timur 13230	Email: erlina_burhan@yahoo.com<br>Tel: +62 816 1628 471<br>Fax:
MAKASSAR, INDONESIA, DR WAHIDIN SUDIROHUSODO HOSPITAL	Dr Irawaty Djaharuddin (PI)<br>Jalan Perintis Kemerdekaan Kampus Tamalanrea Km. 10, Makassar 90245 Sulawesi Selatan, Indonesia	Email: irawatymuzakkir@gmail.com<br>Tel: + 62 811 447082<br>Fax:
SURABAYA, INDONESIA, DR SOETOMO GENERAL HOSPITAL	Dr Tutik Kusmiati (PI)<br>Jl. Mayjend Prof. Dr. Moestopo No. 6-8, Surabaya, Jawa Timur 60285, Indonesia	Email: tutik_kusmiati@yahoo.com<br>Tel: +62 818 0300 4561<br>Fax:
MALANG, INDONESIA, DR SAIFUL ANWAR HOSPITAL	Dr Yani Jane R. Sugiri (PI)<br>Jalan Jaksa Agung Suprapto No. 2, Malang 65111, Indonesia	Email: yani.sugiri@yahoo.com<br>Tel: +62 811 366847<br>Fax:
SOLO, INDONESIA, DR MOEWARDI GENERAL HOSPITAL	Dr Jatu Aphridasari (PI)<br>Jl. Kolonel Sutarto No.132, Jebres, Kec. Jebres, Kota Surakarta, Jawa Tengah 57126	Email: jatuparu@gmail.com<br>Tel: +62 817 9450255<br>Fax:
QUEZON CITY, PHILIPPINES LUNG CENTER OF THE PHILIPPINES	Dr Vince Balanag Jr. (PI)<br>Lung Centre of the Philippines, Quezon Avenue, Quezon City	Email: vmbalanag@gmail.com<br>Tel: 924-6101 local 221/300<br>Fax:
QUEZON CITY, PHILIPPINES QUEZON INSTITUTE	Dr Jubert P. Benedicto (PI)<br>Philippine Tuberculosis Society, Inc.<br>Quezon Institute Compound<br>E. Rodriguez Sr. Ave., 1101<br>Quezon City, Philippines	Email: jubertpb@yahoo.com<br>Tel: (632) 781-3755<br>Fax:
CAVITE, PHILIPPINES	Dr Victoria Basa-Dalay (PI)<br>De La Salle Health Sciences Institute, Dasmarinas City, Cavite, Philippines	Email: vbasa.dalay@gmail.com

DE LA SALLE HEALTH SCIENCES INSTITUTE		Tel: (632) 988 3100 Extn: 1374/1373<br>Fax: 02 988 3100 loc. 1374
CEBU CITY, PHILIPPINES PERPETUAL SUCCOUR HOSPITAL	Dr Ma. Bernadita Sarcauga-Chua (PI)<br>Perpetual Succour Hospital, Gorordo Avenue, Cebu City, Philippines	Email: bebotmd888@gmail.com<br>Tel: +63 917 8800399<br>Fax:
MAKATI, MANILA, PHILIPPINES, TROPICAL DISEASE FOUNDATION	Dr Rholine Gem Martin Veto (PI)<br>1 Amorsolo St, Cor Urban Ave, Pio Del Pilar, Makati 1230 Metro Manila, Philippines	Email: rsveto@tdf.org.ph<br>Tel: +632-3598681,894-0741/43<br>locals 112,113<br>Fax:
SINGAPORE, NATIONAL UNIVERSITY HOSPITAL	Prof Nicholas Paton (PI)<br>1E Kent Ridge Road, #13-00, Singapore 119228	Email: nick_paton@nuhs.edu.sg<br>Tel:<br>Fax:
BANGKOK, THAILAND, CHULALONGKORN HOSPITAL	Dr Anchalee Avihingsanon (PI)<br>1873 Rama 4 Road, Bangkok, Pathumwan 10330, Thailand	Email: anchalee.a@hivnat.org<br>Tel: (+66) 2-652-3040 ext. 107,<br>(+66) 86-812-8889<br>Fax: (+66) 2 2508890
NONTHA BURI, THAILAND CENTRAL CHEST INSTITUTE OF THAILAND	Dr Piamlarp Sangsayunh (PI)<br>Bang Kraso, Mueang Nonthaburi District, Nonthaburi, Thailand	Email: piamlarp@yahoo.com<br>Tel: (+66)-818-454-950<br>Fax: (+66) 2 591 9252
BANGKOK, THAILAND, TAKSIN HOSPITAL	Dr Supunnee Jirajariyavej (PI)<br>543 Somdejjoaphraya Rd, Khlongsarn, Bangkok, Thailand 10600	Email: jsupunee@yahoo.com<br>Tel:<br>Fax:
KAMPALA, UGANDA, INFECTIOUS DISEASES INSTITUTE	Dr Christine Sekaggya-Wiltshire (PI)<br>P.O. Box 22418<br>Kampala, Uganda	Email: csekaggya@idi.co.ug<br>Tel:<br>Fax:
KAMPALA, UGANDA, JOINT CLINICAL RESEARCH CENTRE	Dr Cissy Kityo Mutuluuza (PI)<br>Plot 101, Entebbe Road, Lubowa Estates, P.O Box 10005, Kampala, Uganda	Email: ckityo@jcrc.org.ug<br>Tel: (+256) 417723000<br>Fax:
MBARARA, UGANDA, JOINT CLINICAL RESEARCH CENTRE	Dr Abbas Lugemwa (PI)<br>Mbarara Regional Referral Hospital<br>P.O. Box 1410 Mbarara District, Uganda	Email: alugemwa@jcrc.org.ug<br>Tel: +256 485 433545<br>Fax:

**GULU, UGANDA, JOINT CLINICAL RESEARCH CENTRE**	Dr Robert Kidega (PI)<br>Koro Sub County -<br>Kampala - Gulu Road<br>P.O. Box 160, 7 Gulu District, Uganda	Email: rkidega@jcrc.org.ug<br>Tel: (+256) 481 660318<br>Fax:
**CHENNAI, INDIA, NATIONAL INSTITUTE FOR RESEARCH IN TUBERCULOSIS**	Dr Syed Hissar (PI)<br>No.1, Mayor Sathiyamoorthy Road, Chetpet, Chennai, Tamil Nadu 600031, India	Email:<br>syed.hissar@nirt.res.in<br>Tel:<br>Fax:
**DELHI, INDIA, NATIONAL INSTITUTE OF TUBERCULOSIS AND RESPIRATORY DISEASES**	Prof (Dr) Rohit Sarin (PI)<br>Sri Aurobindo Marg, Seth Sarai, Mehrauli, New Delhi, Delhi 110030, India	Email: r.sarin@nitrd.nic.in<br>Tel: (+91) 9111 26963335<br>Fax:
**AHMEDABAD, INDIA, B J MEDICAL COLLEGE & CIVIL HOSPITAL**	Dr Rajesh Solanki (PI)<br>New Civil Hospital, Civil Hospital Rd, Haripura, Asarwa, Ahmedabad, Gujarat 380016, India	Email:<br>rns04sec@yahoo.co.in<br>Tel: +91 98253 19344,<br>+91 97277 22885<br>Fax:

APPENDIX 8: TEMPLATE FOR CONSENT FORM

This form is the English language template for the trial consent form, provided for background information. A version adapted for the local setting at each site and compatible with national ethics and regulatory requirements and translated into the local language(s) will be provided as a separate document for review alongside this protocol.

Consent Form for the TRUNCATE-TB Trial

[Each site to insert version number and date of consent form here]

Two-month Regimens Using Novel Combinations to Augment Treatment Effectiveness for drug-sensitive Tuberculosis (TB): a randomised controlled non-inferiority trial (TRUNCATE-TB)

[Each site to use its own consent form, translated into local language(s) and presented on local headed paper with any additional cover sheet information required by local IRB/Ethics Committee; any modifications to the text must be discussed with the trial coordinating centre prior to submission to the IRB/Ethics Committee]

Invitation

You are being invited to take part in a research study called TRUNCATE-TB. This study aims to enrol approximately 900 patients in 10-15 treatment centres in 4-6 countries over 2 years.

Having people like you join such a study is the only way we can find out for sure if there are better ways to treat TB. Your participation in this study may be of help to you and many other people with TB in the future.

Before you decide to participate, it is important that you understand why the research is being done and what it will involve. Please read the following information carefully and discuss it with others if you wish. You will be given a copy of this form to keep. Please ask us if anything is not clear or if you have any other questions.

Participation is entirely voluntary. If you decide not to take part, it will not affect your medical care, and you will receive the normal standard TB treatment. If you take part, you may decide later to withdraw from the study at any time without giving a reason and without affecting your future care. If you withdraw from the study you will receive the normal standard TB treatment or alternative as advised by your doctor.

Why have I been invited to take part in this study?

You have been invited because you have TB of the lungs that is being treated for the first time.

What is the background to the study?

TB is caused by a germ (bacterium) and commonly affects the lungs. Standard TB treatment is usually taken for 6 months, but some patients find this difficult and do not take the medicines regularly. This increases the risk that the treatment will not clear the TB infection completely, so it remains at the end of treatment (what we call

"treatment failure") or it comes back again later (what we call "relapse"). If there is treatment failure or relapse, the TB has to be treated again for at least another 6 months. Sometimes, when there is treatment failure or relapse, the standard TB medicines may not work as well (what we call "resistance") and so we have to change to medicines that may be more difficult to take and require treatment for longer than 6 months.

What is the purpose of the study?

In this study we are testing a new approach to TB treatment that we think may be better for most people. This approach involves taking a much shorter course of treatment (2 months) using combinations of new medicines and standard medicines in normal and/or higher doses. We believe these combinations may be stronger than the standard treatment – we call these new combinations "boosted treatment". We expect that the boosted treatment will kill the TB in most people within 2 months. After the boosted treatment is completed we will maintain close contact with you and do regular checks for TB so that we can detect early treatment failure or relapse in the expected small number of people who have not cleared the infection. For these few people the TB should be successfully cleared by a course of standard treatment for 6 months. As most people should receive a much shorter course of treatment, with just a minority needing longer treatment for around 6 months, we think this may be better for patients overall. This new approach will also allow the TB clinics to spend more time supporting those who really need longer treatment.

The purpose of this study is to find out whether this new treatment approach is as good as the standard 6 months treatment.

What will be the expected duration of my participation?

Your participation will last for 96 weeks (just less than 2 years).

How can I join the TRUNCATE-TB study?

If after reading this information sheet and asking any questions you decide you would like to join the study, you will first sign a copy of this consent form. Then we will see if you are suitable for the study by asking about your medical history, doing a physical examination, performing a chest X-ray and an electrical recording of your heart beat, and collecting a sample of your sputum (the material that you cough up). We will also do some standard blood tests to check the overall function of your body organs (such as your kidneys and liver) as well as an HIV test. The blood tests will require 15ml (3 teaspoons) of blood. If you are female of child-bearing age we will do a urine pregnancy test.

Based on the results of these tests, your doctor will tell you whether you are able to enter the study.

If your HIV test is positive, we will refer you to see specialist doctors for your HIV care. If you are HIV positive most likely you will not be able to participate in the study, but your doctor will advise whether it may be allowed. If it might be possible then we would need to do an extra test called a CD4 count that assesses the strength of your immune

system. This will require an extra 5ml (1 teaspoon) of blood, but this would be needed for your routine care anyway. If you are found to be HIV-positive, the study team will be obliged by law to inform the Ministry of Health and relevant authorities [site to delete/amend as necessary].

What will happen if I take part in the study?

Treatment

Allocation to a treatment group

After you enter the study, you will either start or continue on the standard combination treatment to complete 6 months or start or change to one of the boosted treatments for 2 months.

Neither you nor your doctor will be able to choose to which treatment group you will be allocated because the selection will be made by a computer, completely by chance – like rolling a dice. There is an equal chance that you will be allocated to each treatment group. We need to allocate treatment in this way so that the study is able to compare the different groups.

At the time you enter the study the allocation to some of the treatment groups may already have been completed. The remaining ones to which you might be allocated are indicated by a check in the box below. You will only be allocated to **ONE** of the possible treatment groups:

[investigator to indicate with a check which arms are open for randomisation]

- ☐ **Group A:** 24 weeks standard treatment of rifampicin, isoniazid, pyrazinamide, ethambutol (pyrazinamide and ethambutol stopped after the first 8 weeks)

- ☐ **Group B**: 8 weeks rifampicin (higher-dose than usual), isoniazid, pyrazinamide, ethambutol and linezolid

- ☐ **Group C**: 8 weeks rifampicin (higher-dose than usual), isoniazid, pyrazinamide, ethambutol and clofazimine

- ☐ **Group D:** 8 weeks rifapentine, isoniazid, pyrazinamide, linezolid, levofloxacin

- ☐ **Group E**: 8 weeks isoniazid, pyrazinamide, ethambutol, linezolid, bedaquiline

Standard treatment

If you are taking standard treatment (either because you were allocated to Group A or because you switched from one of the boosted treatments) and experience side effects then we will adjust the medication to minimise these. If you have treatment failure or relapse after completing this standard treatment then you will be re-treated according to standard international recommendations. This usually requires 8 months treatment using the standard combination plus another drug (streptomycin) that is

given by injection for the first 2 months. Drug resistance tests will be performed to make sure you are on the best re-treatment combination.

Boosted treatments

The medications used for boosted treatments have all been approved for the treatment of TB or other infections and have already been taken by millions of people (hundreds in the case of bedaquiline). The combination of medicines in the boosted treatments have been chosen, based on the latest available scientific evidence, to have the best chance of working well together to cure TB in the shortest time whilst keeping side effects to the minimum.

The boosted treatments will require you to take more pills each day than the standard treatment but for a much shorter duration. The exact number of pills per day will depend on the particular treatment group and your body weight. Details are provided under each treatment description (below) and your doctor can give you more information about the number of pills for your specific body weight. All the treatments can be taken once a day

If you experience side effects on a boosted treatment that are not controlled by reducing the medication dose, then we will switch you to standard treatment (as in Group A, above) to complete a total course of approximately 6 months.

If you still have TB symptoms and TB in your sputum after 8 weeks on a boosted treatment, we will extend the boosted treatment to 12 weeks with some or all of the same medicines. In the very unlikely event that you still have TB symptoms and TB in your sputum at 12 weeks we will switch you to the standard treatment (as in Group A, above) to complete a total course of approximately 6 months.

If you have treatment failure or relapse after the course of boosted treatment has ended then you will be re-treated with a standard course of treatment (as in Group A, above) for 6 months. Drug resistance tests will be performed in the laboratory to make sure you are on the best re-treatment combination.

Supporting your treatment

It is very important that you do not miss doses of treatment because this increases the chances of treatment failure, relapse and resistance. To help you take your medicines regularly, we will arrange to supervise your treatment for at least the first 2 months. If you are allocated to the 6 months standard treatment, the supervision may need to be longer, depending on standard practice in your country. Supervision means that a nurse or community worker (or sometimes a responsible family member or friend) will need to watch you swallow your medication each day. Where possible, we will arrange for this to be done in a way that is convenient to you, such as having it done at home or at your workplace. Even if you do not take part in the study, it is likely that there you will need some treatment supervision according to the policy in your country. With your agreement, we may also try to find additional ways of helping you take your medicine such as mobile phone messages.

If you miss doses, the period of treatment may need to be extended to allow you to complete the treatment. If you miss too many doses or stop treatment for a period of more than 2 weeks you may need to start a new course of standard treatment

You can take other, non-TB medicines but should inform the study doctor so they can check there is no problem taking them with TB medicines.

Timing of study visits

You will need to attend the study clinic on the day you enter the study, then 1 week and 2 weeks later, then every 2 weeks for the first 3 months (12 weeks), then every 4 weeks until 6 months (24 weeks), and then every 12 weeks until you have reached week 96 (almost 2 years). In total you will need to attend the study clinic for approximately 18 visits over 2 years.

After the first 3 months and up to week 96, we will also contact you by telephone approximately once a month. If you experience any TB symptoms in-between the monthly contact it is important that you inform the study team and not wait for the next call or your next clinic visit.

If you need re-treatment, you will need to attend additional clinic visits to monitor this treatment, with similar frequency to those during your first course of treatment. Where possible, these additional visits will be combined with the other scheduled study visits to minimise the burden on you. If relapse occurs later in the study this may require a few extra visits after week 96.

We will contact you again by telephone when the study ends to ask if you have had any further episodes of TB since your last study visit.

It is essential that you attend all the scheduled study visits. If you do not attend a visit, we will contact you to rearrange the visit as soon as possible. At the beginning of the study we will also ask you for contact details of several people whom you wish to be supporters of your treatment, such as friends, family and work colleagues who see you frequently. If you miss a scheduled clinic visit and we are unable to contact you directly after several attempts then we will contact your named treatment supporters to find out about you. One of the clinic or research staff may also visit your home to try and find you. We may also look at your routine clinical care records or national health records to check your progress. In agreeing to participate in this study, you are giving permission for us to take these steps to find out about your health if you do not attend a scheduled clinic visit.

What will happen at the study visits?

Most of these clinic visits will be brief (taking no more than 30 minutes); you will need to stay longer (1-4 hours) on the day you enter the study and at weeks 4 and 8. At each visit we will ask you about your symptoms, medication, general health and use of health services and perform a brief physical examination. If you are able to produce a sputum sample we will check whether this still contains TB.

We will take a blood sample (approximately 10ml, or 2 teaspoons) on the day you enter the study, at 1, 4 and 8 weeks later and at the end of your treatment to check the general functioning of your body organs. Some of these tests are routine blood tests that would be done as part of your normal care, but we are doing them more often than normal to increase the safety for you of participating in the study. If your

treatment combination includes linezolid we will take an additional blood sample (5ml, or 1 teaspoon) at week 2 and week 6 (and at week 10 if your treatment is extended). On the day you enter the study, and at weeks 4 and 8 we will take additional blood samples before and after you take your pills (one or two samples, each of 5ml or 1 teaspoon) to measure the concentrations of medications in your body. If your treatment combination includes bedaquiline or clofazimine we will take a blood sample (5ml, or 1 teaspoon) to measure medication concentration at week 24. On the day you enter the study, at weeks 4, 8 and 24 and at the end of your treatment, we will take a blood sample (10ml, 2 teaspoons) to store for future testing. In total we will collect approximately 165mls of blood (approximately 11 tablespoons) over the entire study period.

If you need re-treatment, one additional blood test will be needed to check the general functioning of your body organs at 4 weeks after starting re-treatment (10ml, 2 teaspoons), and one additional blood sample (10ml, 2 teaspoons) for storage at 8 weeks after starting re-treatment.

You will need to provide a urine specimen (approximately 10 to 20ml, or 2 to 4 teaspoons) on the day you enter the study, at weeks 4, 8 and 24 and at the end of your treatment for storage. For women of child-bearing age, we will do a pregnancy test on the urine samples on the day you enter the study, at weeks 4 and 8 and at the end of treatment.

We will do a chest X-ray on the day you enter the study and at week 8 and at the end of your course of treatment and at week 96. We will do an electrical recording of your heart beat on the day you enter the study and at weeks 1, 4 and 8 and, if you have been taking a boosted treatment, at the end of your course of treatment. We will ask you some questions about your quality of life on the day you enter the study and at week 96, and ask your overall opinion about your TB treatment at weeks 48 and 96. We will ask you to blow into a small tube (like blowing out candles) to measure the strength of your lungs at weeks 8, 48 and 96.

If you experience treatment failure or relapse you may need an additional chest X-ray, additional sputum tests, and additional blood and urine sample for storage.

The blood and urine samples we collect for storage will be kept for a period of up to 5 years after the study ends for the future analyses. The samples may be used for tests measuring the levels of biological substances in the body that may help assess the activity of TB disease or predict TB treatment response (what we call “biomarkers”). There are no tests that can do this accurately now, but there may be some that become available at the end or after the study. Future analyses on samples will only be related to TB or medicines used to treat TB. The decision of what tests to perform (if any) will be made near the end of the study and the decision will be reviewed by the ethics committee that oversees research in your study centre/country. If permitted by the ethics committee and national regulations in your country, some of these samples may be sent outside your country for these tests. We will not inform you of the results of these tests as they will not be of relevance for decisions about your clinical care.

If any new information becomes available that may be relevant to your taking part in the study, you will be informed as soon as possible by the study investigator.

What are the possible side effects of the medication used in the study?

Below we list the main side effects for each of the possible treatments to be tested in this study. Allocation to some of the treatment groups may already have been completed when you enter the study, so for each treatment we have indicated whether it is possible you might receive it or whether you will definitely not receive it (in which case you can ignore the possible side effects).

A common side effect means that that between 1% and 10% of people had that side effect (or, out of 100 people, it affected more than one, but fewer than 10 people). The list also gives information on known severe side effects, however common or rare they are.

For the combination that you do receive in the study, you may or may not have any of these side effects listed or you may have other side effects not included in the lists. Experience with the use of these drugs in new combinations or higher doses is limited and it is possible that unknown or more severe side effects will occur when the drugs are used together.

We will monitor you closely for side effects during this study and also perform regular blood tests to check your health. This monitoring is more detailed than in normal clinical care to give you the maximum protection.

If you experience side effects that you cannot tolerate or that your doctor considers are more serious, then we will either reduce the dose of the likely causative medication or stop the medication entirely, depending on the nature and severity of the side effects. If the side effects are not controlled in this way, then we will switch you to alternative treatment (for the boosted treatment this will mean switching to standard treatment (as in Group A, above).

Group A (24 weeks, standard treatment): rifampicin, isoniazid, pyrazinamide, ethambutol (pyrazinamide and ethambutol stopped after the first 8 weeks)

☑ It is possible you may receive this treatment
☐ You will not receive this treatment

Rifampicin:
Common side effects include: allergic reaction, diarrhoea, abdominal pain, nausea, loss of appetite, vomiting, disturbance of your liver function (usually without symptoms, detected on blood tests), reddish discolouration of body fluids (such as urine), low platelets (a type of blood cell) and itchy skin.
Serious side effects include: liver damage (rare), kidney damage (rare), allergic reaction

Isoniazid:
Common side effects include: allergic reaction; pain or discomfort in the hands and feet (we will give you an extra vitamin tablet to prevent this).
Serious side effects include: liver damage (rare).

Pyrazinamide:
Common side effects include: nausea, vomiting, disturbance of your liver function (usually without symptoms, detected on blood tests), and joint pains.
Serious side effects include: liver damage (rare).

Ethambutol:
Common side effects: vision disturbance like blurred vision (reversible upon stopping ethambutol)
Serious side effects include: damage to the eye (uncommon), causing symptoms including decreased vision and colour blindness. The eye symptoms usually go away after stopping treatment.

For this treatment combination you will need to take approximately 2-5 pills each day (depending on your body weight) over the treatment period of 8 weeks, and then 2-5 pills each day for the following 16 weeks (so total of approximately 336-840 pills for the whole treatment course).

Group B (8 weeks): rifampicin (given in a higher-dose than usual), isoniazid, pyrazinamide, ethambutol and linezolid.

- ☐ It is possible you may receive this treatment [investigator to check one box]
- ☐ You will not receive this treatment

Rifampicin:
Common side effects include: allergic reaction, diarrhoea, abdominal pain, nausea, loss of appetite, vomiting, disturbance of your liver function (usually without symptoms, detected on blood tests), reddish discolouration of body fluids (such as urine), low platelets (a type of blood cell) and itchy skin.
Serious side effects include: liver damage (rare), kidney damage (rare), allergic reaction.

There is no evidence that these side effects are more common with high dose rifampicin.

Isoniazid:
Common side effects include: allergic reaction; pain or discomfort in the hands and feet (we will give you an extra vitamin tablet to prevent this).
Serious side effects include: liver damage (rare).

Pyrazinamide:
Common side effects include: nausea, vomiting, disturbance of your liver function (usually without symptoms, detected on blood tests), and joint pains.
Serious side effects include: liver damage (rare).

Ethambutol:
Common side effects: vision disturbance like blurred vision (reversible upon stopping ethambutol)
Serious side effects include: damage to the eye (uncommon), causing symptoms including decreased vision and colour blindness. The eye symptoms usually go away after stopping treatment.

Linezolid:

Common side effects include: low blood count, difficulty sleeping, metallic taste, dizziness, diarrhoea, nausea, vomiting, headache, oral or vaginal thrush (yeast infection), high blood pressure, itchy skin, rash, and disturbance of liver function (usually without symptoms, detected on blood tests).

Serious side effects include: severe skin rash with blistering and peeling (unknown frequency), nerve damage (unknown frequency), damage to the eye causing symptoms including decreased vision and colour blindness (unknown frequency; the eye symptoms usually go away after stopping treatment), severe low blood count (uncommon), severe diarrhoea (unknown frequency) and a syndrome (combination of symptoms) of increased heart rate, shivering and high temperature (unknown frequency).

For this treatment combination you will need to take approximately 6-11 pills each day (depending on your body weight) over the treatment period of 8 weeks (so total of approximately 336-616 pills for the whole treatment course).

Group C (8 weeks): rifampicin (given in a higher-dose than usual), isoniazid, pyrazinamide, ethambutol and clofazimine

- ☐ It is possible you may receive this treatment [investigator to check one box]
- ☐ You will not receive this treatment

Rifampicin:
Common side effects include: allergic reaction, diarrhoea, abdominal pain, nausea, loss of appetite, vomiting, disturbance of your liver function (usually without symptoms, detected on blood tests), reddish discolouration of body fluids (such as urine), low platelets (a type of blood cell) and itchy skin.
Serious side effects include: liver damage (rare), kidney damage (rare), allergic reaction

There is no evidence that these side effects are more common with high dose rifampicin.

Isoniazid:
Common side effects include: allergic reaction; pain or discomfort in the hands and feet (we will give you an extra vitamin tablet to prevent this).
Serious side effects include: liver damage (rare).

Pyrazinamide:
Common side effects include: nausea, vomiting, disturbance of your liver function (usually without symptoms, detected on blood tests), and joint pains.
Serious side effects include: liver damage (rare).

Ethambutol:
Common side effects: vision disturbance like blurred vision (reversible upon stopping ethambutol)
Serious side effects include: damage to the eye (uncommon), causing symptoms including decreased vision and colour blindness. The eye symptoms usually go away after stopping treatment.

Clofazimine:

Common side effects include: dryness of the skin and eyes, vomiting, diarrhoea, nausea, and abdominal pain and darkening of the skin (the skin darkening goes away after stopping treatment, usually within a few weeks but it may take months to years).

Serious side effects include: bowel obstruction, bleeding, and blockage of the blood supply of the spleen (all very rare).

For this treatment combination you will need to take approximately 7-12 pills each day (depending on your body weight) over the treatment period of 8 weeks (total of approximately 392-672 pills for the whole treatment course).

Group D (8 weeks): rifapentine, isoniazid, pyrazinamide, linezolid and levofloxacin

☐ It is possible you may receive this treatment [investigator to check one box]
☐ You will not receive this treatment

Rifapentine:

Common side effects include: reddish discolouration of urine, sweat, sputum, tears, nausea, vomiting, heartburn, disturbance of liver function (usually without symptoms and detected on blood tests), low platelets (a type of blood cell) and allergic reactions.

Serious side effects include: liver damage (rare), severe diarrhoea (rare), severe allergy (rare)

Isoniazid:

Common side effects include: allergic reaction; pain or discomfort in the hands and feet (we will give you an extra vitamin tablet to prevent this).

Serious side effects include: liver damage (rare).

Pyrazinamide:

Common side effects include: nausea, vomiting, disturbance of your liver function (usually without symptoms, detected on blood tests), and joint pains.

Serious side effects include: liver damage (rare).

Linezolid:

Common side effects include: low blood count, difficulty sleeping, metallic taste, dizziness, diarrhoea, nausea, vomiting, headache, oral or vaginal thrush (yeast infection), high blood pressure, itchy skin, rash, and disturbance of liver function (usually without symptoms, detected on blood tests).

Serious side effects include: severe skin rash with blistering and peeling (unknown frequency), nerve damage (unknown frequency), damage to the eye causing symptoms including decreased vision and colour blindness (unknown frequency; the eye symptoms usually go away after stopping treatment), severe low blood count (uncommon), severe diarrhoea (unknown frequency) and a syndrome (combination of symptoms) of increased heart rate, shivering and high temperature (unknown frequency).

Levofloxacin:

Common side effects include: difficulty sleeping, nausea, diarrhoea, vomiting, headache, dizziness, and disturbance of liver function (usually without symptoms, detected on blood tests).

Serious side effects include: changes to the heart beat (rare), inflammation of the gut (intestine) (rare), allergic reactions (rare), tearing of a tendon (the tissue that connects muscle to bone) (very rare), seizures (fits) (rare), kidney failure (very rare), severe skin rash with blisters (unknown frequency).

For this treatment combination you will need to take approximately 14-16 pills each day (depending on your body weight) over the treatment period of 8 weeks (so total of approximately 784-896 pills for the whole treatment course).

Group E (8 weeks): isoniazid, pyrazinamide, ethambutol, linezolid and bedaquiline

- ☐ It is possible you may receive this treatment [investigator to check one box]
- ☐ You will not receive this treatment

Isoniazid:
Common side effects include: allergic reaction; pain or discomfort in the hands and feet (we will give you an extra vitamin tablet to prevent this).
Serious side effects include: liver damage (rare).

Pyrazinamide:
Common side effects include: nausea, vomiting, disturbance of your liver function (usually without symptoms, detected on blood tests), and joint pains.
Serious side effects include: liver damage (rare).

Ethambutol:
Common side effects: vision disturbance like blurred vision (reversible upon stopping ethambutol)
Serious side effects include: damage to the eye (uncommon), causing symptoms including decreased vision and colour blindness. The eye symptoms usually go away after stopping treatment.

Linezolid:

Common side effects include: low blood count, difficulty sleeping, metallic taste, dizziness, diarrhoea, nausea, vomiting, headache, oral or vaginal thrush (yeast infection), high blood pressure, itchy skin, rash, and disturbance of liver function (usually without symptoms, detected on blood tests).

Serious side effects include: severe skin rash with blistering and peeling (unknown frequency), nerve damage (unknown frequency), damage to the eye causing symptoms including decreased vision and colour blindness (unknown frequency; the eye symptoms usually go away after stopping treatment), severe low blood count (uncommon), severe diarrhoea (unknown frequency) and a syndrome (combination of symptoms) of increased heart rate, shivering and high temperature (unknown frequency).

Bedaquiline:

Common side effects include: joint and muscle pains, nausea, vomiting, diarrhoea, headache, dizziness, and disturbance of liver function (usually without symptoms, detected on blood tests). Serious side effects include: changes to the heartbeat (rare).

Note: In one clinical trial there were more deaths in patients who took bedaquiline compared to those who did not. These deaths did not appear to be caused by bedaquiline and other studies have not found a difference. This is most likely to be chance finding (in other words, not a true risk to you), but if any new information becomes available that confirms this risk then this study treatment will be stopped.

For this treatment combination you will need to take approximately 9-14 pills each day (depending on your body weight) for the first 2 weeks of treatment and 7-12 pills each day for weeks 3-8 of treatment (so total of approximately 372-652 pills for the whole treatment course)

What are the possible risks to me if I join the study?

There may be a higher risk of side effects if you take one of the boosted treatments compared to receiving standard TB treatment in your local clinic. If you have side effects we will adjust your treatment to minimise them.

If you join the study and take one of the boosted treatments, you may have a higher chance of treatment failure or relapse than if you were to receive standard TB treatment in your local clinic. Although we cannot be certain how much higher this chance is, we think it is likely to be a relatively small difference overall (because the boosted treatments may be stronger, and because of all of the extra help you will have with taking your medicines regularly). If you join the study and receive the standard 6 month treatment, the chance that you will have treatment failure or relapse is likely to be lower than if you receive standard TB treatment in your local clinic (because of the help with taking medicines regularly). The number of people with treatment failure or relapse will be monitored closely in the study and if this occurs in more than approximately 20% (or 1 in 5) people taking a particular boosted treatment then this treatment will be stopped.

If you have treatment failure or relapse (whether on the standard or boosted treatments) there is a risk that this may be harmful as described below:

Firstly, there is a risk that the TB may damage your lungs. We will follow you closely after completion of your TB treatment, so that we can detect symptoms of treatment failure or relapse early, perform the right diagnostic tests early, and restart treatment promptly. The risk that you will have any serious harm from treatment failure or relapse in this situation is very low.

Secondly, there is a risk of developing resistance to one or more TB medicines. If there is resistance the new treatment may need to be much longer and include other drugs that have additional side effects. Drug resistance is very rare in people in a clinical trial (because of the extra help with taking medication regularly) and the risk should be very low in this study too.

Thirdly, if the TB comes back you may become infectious to people around you, especially to those who live with you. These people may need to be tested for TB and possibly take preventive medication. However, because we will stay in close contact with you in this study, it is most likely that you will be able to re-start treatment before you become an infection risk to the people living with you, so this risk is also low.

Overall, although there may be a higher chance of treatment failure or relapse if you are in the study as opposed to receiving standard treatment in your local clinic, the risk of harm to you or others arising from an episode of treatment failure or relapse is likely to be lower in the study (because of the help with taking medication regularly, and the earlier detection, diagnosis and start of re-treatment) than it is for patients receiving treatment in the normal clinic. In addition, the study will provide both you and your contacts rapidly and free-of-charge with the tests and treatment needed to minimise or prevent harm after an episode of treatment failure or relapse. This may not be so easily available for people receiving standard TB treatment in the local clinic (see benefits of the study, below).

You should also consider the additional burden of study visits and tests required by taking part in the study (although we will try to make these easy for you and the study will pay for your transport costs and time as described below). There are minor risks of taking blood including bleeding, pain, infection, and bruising.

Risks in pregnancy

The standard TB drugs are safe in pregnancy.

At present there is not enough known about the effects of the other study drugs in pregnant women. Animal studies indicate a possible risk to the unborn child with clofazimine, rifapentine, linezolid and levofloxacin. Animal studies of bedaquiline do not suggest a risk to the unborn child.

Pregnant and breastfeeding women are not allowed to join the study.

Rifampicin and rifapentine can decrease the effectiveness of hormonal contraceptives. You must use an effective method of barrier contraception, such as a condom for 6 months from when you enter the study, or have a coil in place, or be surgically sterilised (i.e. have your tubes tied).

If you become pregnant during the study and are taking one of the boosted treatments, then we will change your treatment to the standard TB treatment. We will continue to follow you up in the study until the outcome of the pregnancy is known.

If you are male, and your female partner become pregnant during the study, you can continue to participate in the study, but we would like to collect information about the outcome of the pregnancy by asking you questions at clinic visits.

What are the possible benefits to me if I join the study?

You will receive all the TB medical care, tests and drugs required for the study free of charge including treatment for any side effects and alternative drugs in the unlikely event that you develop resistance.
You may benefit from receiving one of the boosted treatment combinations, if this is effective in curing your TB in a shorter time than usual.
You may benefit from more resources devoted to your clinical care, including extra monitoring by clinical staff and extra tests such as detailed drug resistance testing.

The study will also provide free testing and TB treatment to any household contacts in the unlikely event that they have been put at risk of TB by your participation in the study.

Will I be reimbursed for my participation in this study?

The study will be performed at no charge to you. You will receive reimbursement for your time and transport costs associated with travelling to the clinic at the following rates:

[Currency X] [amount AAA] for travel less than [1]km, [amount BBB] for travel more than [1]km for visits for treatment supervision if this requires additional travel to the clinic or approved treatment supervisor

[Currency X] [amount CCC] for all planned study visits

[Currency X] [amount DDD] for the week 96 visit

If you withdraw from the study before the end, you will be reimbursed for the visits you have completed up to that point.

What if something goes wrong?

The study will provide free medical treatment for any conditions arising from your participation in this study (see above).

If something goes wrong and you are harmed as a result of participating in the study, even if no one involved in the study was at fault, then you may be eligible to apply for compensation. The study Sponsor, University College London (UCL), who represents the researchers organising the study, has obtained trial-related insurance for this purpose. If you think you have a case for compensation you should first discuss this possibility with your study doctor. The hospital(s) you are treated in continue to have a duty of care to you, whether or not you agree to participate in this study, and UCL does not accept liability for fault on the part of employees of the hospital.

By signing the Informed Consent Form attached, you do not waive any of your legal rights or release the parties involved in this study from liability for negligence.

What will happen if I want to leave the study?

You will be free to leave the study at any time. You do not have to give a reason, although it is helpful for us if you do. If you leave, we will still keep records and samples relating to the treatment given to you, as this is important for the study. If you withdraw, we will also still collect information on your progress from the routine healthcare system unless you specifically request this to stop. A decision to leave the study at any time will not affect the quality of care you receive. If you do not inform us about wishing to leave the study, and we have not been able to get in contact with you for more than 6 months, we wish to check any available national health records to see whether you are OK.

What happens to the information collected in the study?

The study clinical staff will collect information at each visit. This information will be stored both on paper and in a computer and will be checked and analysed by study staff in [**insert name of country**], Singapore and the UK. The information will be regularly reviewed by an independent group of international experts to ensure both your safety and that the study is being properly conducted. Information from TRUNCATE-TB will be analysed and be presented and published. We will make such publications publicly available, including to yourself and your community. Your name or identity will remain confidential within the study team.

Samples (blood, urine, sputum) collected during the study may be exported to overseas centres for analysis if permitted by national regulations and subject to ethics committee approvals where required. If we need to transfer some of your samples for analysis overseas, they will be coded and will not contain your name or any information that could identify you.

Will my taking part in the study be kept confidential?

All information about you will be kept confidential and anonymous and will not be made available to anyone who is not connected with the study without your consent. If you agree to join the study, persons authorised by University College London (the study Sponsor), as well as ethics and regulatory authorities [site to insert names of relevant local authorities] will be granted direct access to your original medical records to check study procedures and data without making any of your information public. Study records may also be inspected by independent auditors or government regulators to ensure that the study is being carried out correctly and safely.

Further information

After reading this information sheet, if you feel you do not fully understand everything, please ask for further information from the doctors, nurses or other members of the study team. If you later need more information, please call:

[Insert name / telephone number appropriate for study site]

Name: …………………………………

Telephone Number: ……………………………………………

CONSENT FORM FOR TRUNCATE-TB TRIAL: SIGNATURE PAGE

Please check each box.

- ☐ I confirm that I have read the Consent Form Version [site to insert version number before printing], dated [site to insert version date before printing], for the above study. I have had the opportunity to consider the information, ask questions and have had these answered satisfactorily.

- ☐ I understand that my participation is voluntary and that I am free to withdraw at any time without giving any reason, without my medical care or legal rights being affected.

- ☐ I understand that information collected about me will be anonymised and may be shared with other researchers.

- ☐ I understand that some of my blood and urine samples will be stored and may be used for future tests of the activity of TB disease or prediction of TB treatment response. These tests may be sent overseas for testing if approved by the local ethics committee and other regulations.

- ☐ I consent voluntarily to participate in this study.

Name	Signature	Date
Name of researcher taking consent	Signature	Date
Name of witness	Signature	Date

APPENDIX 9: SAFETY PROFILE OF TRIAL DRUGS IN PREGNANCY

The following summarises the information on the safety profile of the drugs in pregnancy based on the SPCs for the individual drugs supplemented by available published data that is correct at the date that this version of the protocol was finalised. This information will be updated in the protocol at the time of any subsequent amendments to the main body of the protocol. This information is likely to change in the intervening period as new information becomes available. Investigators will be provided with revised SPCs for individual drugs as and when these are updated by the manufacturers. Any important changes to the safety profile of the drugs in pregnancy will be notified separately to the investigators based on the changes to the SPCs as reported to the FDA.

All 4 first line drugs (isoniazid, rifampicin, ethambutol and pyrazinamide) have an excellent safety record in pregnancy and are not associated with human foetal malformations.[74]

Bedaquiline is in FDA pregnancy category B, that is animal reproduction studies have failed to demonstrate a risk to the foetus and there are no adequate and well-controlled studies in pregnant women. The manufacturer's SPC states that at clinically relevant exposures, animal studies do not indicate direct or indirect harmful effects with respect to reproductive toxicity. In animal studies, bedaquiline had no effects on fertility when evaluated in female rats. Three out of 24 male rats treated with high bedaquiline doses failed to produce offspring in a fertility study, but normal spermatogenesis was observed in these rats. The corresponding plasma exposure (AUC) was 2-fold higher in rats compared to humans. Bedaquiline is secreted in the breast milk of lactating rats, concentrations of which 6-12-fold higher than the maximum concentration observed in maternal plasma.

Clofazimine is in FDA category C. Clofazimine crosses the human placenta. Adequate and well-controlled studies in humans have not been done. Although the skin of infants born to mothers who received clofazimine during pregnancy was deeply pigmented at birth, clofazimine has not been shown to be teratogenic in humans; this pigmentation faded with time. Clofazimine is excreted in breast milk and therefore it is not recommended in nursing mothers. The skin and fatty tissues of animal offspring become discolored approximately 3 days after birth. A gradual fading of pigmentation followed discontinuation of breast-feeding. There may be an effect on fertility- in one study rats given 25-times the usual human dose of clofazimine resulted in reduced number of offspring.

There is no adequate data from the use of **linezolid** in pregnant women. Linezolid decreased fertility and reproductive performance of male rats at exposure levels approximately equal to those expected in humans. Studies in mice and rats showed no evidence of a teratogenic effect at exposure levels 4 times or equivalent, respectively, to those expected in humans. The same linezolid concentrations caused maternal toxicity in mice and were related to increased embryo death including total litter loss and decreased fetal body weight. In rats, slight maternal toxicity was noted at exposures lower than expected clinical exposures. Animal data suggest that linezolid and its metabolites may pass into breast milk and, accordingly, breastfeeding should be discontinued prior to and throughout administration.

Levofloxacin is in FDA category C. Animal studies do not indicate direct or indirect harmful effects with respect to reproductive toxicity. However in the absence of human data and due to that novel data suggest a risk of damage by fluoroquinolones to the weight-bearing cartilage of the growing organism, levofloxacin must not be used in pregnant women. As experimental data suggest a risk of damage by fluoroquinolones to the weight-bearing cartilage of the growing organism, levofloxacin must not be used in breast-feeding women.

Rifapentine has been assigned to FDA pregnancy category C. Animal studies using doses similar to or less than the human dose (based on body surface area) have revealed evidence of teratogenicity. There are no controlled studies in human pregnancy. In one clinical study, six patients randomised to rifapentine became pregnant during treatment. There were 2 normal deliveries, 2 first trimester spontaneous abortions, one elective abortion, and one lost to follow-up. Of the two patients with spontaneous abortions, one had a history of ethanol abuse and the other had HIV infection.

APPENDIX 10: DIVISION OF AIDS TABLE

General Instructions:
If the need arises to grade a clinical adverse event (AE) that is not identified in the DAIDS AE grading table, us the category "Estimating Severity Grade" located at the top of the table.

If the severity of an AE could fall under either one of two grades (e.g. the severity of an AE could be either Grade 2 or Grade 3) select the higher of the two grades for the AE.

Definitions:

**Basic Self-care Functions**	Adult: Activities such as bathing, dressing, toileting, transfer/movement, continence, and feeding.
**Usual Social & Functional Activities**	Adult: Adaptive tasks and desirable activities, such as going to work, shopping, cooking, use of transportation, pursuing a hobby etc.

CLINICAL				
**PARAMETER**	**GRADE 1 MILD**	**GRADE 2 MODERATE**	**GRADE 3 SEVERE**	**GRADE 4 POTENTIALLY LIFE-THREATENING**
**ESTIMATING SEVERITY GRADE**				
Clinical adverse event NOT identified elsewhere in this DAIDS AE grading table	Symptoms causing no or minimal interference with usual social & functional activities	Symptoms causing greater than minimal interference with usual social & functional activities	Symptoms causing inability to perform usual social & functional activities	Symptoms causing inability to perform basic self-care functions OR medical or operative intervention indicated to prevent permanent impairment, persistent disability, or death
**SYSTEMIC**				
Acute systemic allergic reaction	Localized urticaria (wheals) with no medical intervention indicated	Localized urticaria with medical intervention indicated OR mild angioedema with no medical intervention indicated	Generalized urticaria OR angioedema with medical intervention indicated OR symptomatic mild bronchospasm	Acute anaphylaxis OR Life-threatening bronchospasm OR laryngeal edema
Chills	Symptoms causing no or minimal interference with usual social & functional activities	Symptoms causing greater than minimal interference with usual social & functional activities	Symptoms causing inability to perform usual social & functional activities	NA
Fatigue<br><br>Malaise	Symptoms causing no or minimal interference with usual social & functional activities	Symptoms causing greater than minimal interference with usual social & functional activities	Symptoms causing inability to perform usual social & functional activities	Incapacitating fatigue/ malaise symptoms causing inability to perform basic self-care functions
Fever (nonaxillary)	37.7 – 38.6°C	38.7 – 39.3°C	39.4 – 40.5°C	> 40.5°C
Pain (indicate body site)<br><br>DO NOT use for pain due to injection (See Injection Site Reactions: Injection site pain) See also Headache, Arthralgia, and Myalgia	Pain causing no or minimal interference with usual social & functional activities	Pain causing greater than minimal interference with usual social & functional activities	Pain causing inability to perform usual social & functional activities	Disabling pain causing inability to perform basic self-care functions OR hospitalization (other than emergency room visit) indicated
Unintentional weight Loss	NA	5 – 9% loss in body weight from baseline	10 – 19% loss in body weight from baseline	≥ 20% loss in body weight from baseline OR aggressive intervention indicated [e.g., tube feeding or total parenteral nutrition (TPN)
**INFECTION**				
Infection (any other than HIV infection)	Localized, no systemic antimicrobial treatment indicated AND Symptoms causing no or minimal interference with	Systemic antimicrobial treatment indicated OR Symptoms causing greater than minimal interference with usual social &	Systemic anti-microbial treatment indicated AND Symptoms causing inability to perform usual social & functional activities OR Operative	Life-threatening consequences (e.g., septic shock)

CLINICAL				
**PARAMETER**	**GRADE 1 MILD**	**GRADE 2 MODERATE**	**GRADE 3 SEVERE**	**GRADE 4 POTENTIALLY LIFE-THREATENING**
	usual social & functional activities	functional activities	intervention (other than simple incision and drainage) indicated	
**INJECTION SITE REACTIONS**				
Injection site pain (pain without touching)<br><br>Or<br><br>Tenderness (pain when area is touched)	Pain/tenderness causing no or minimal limitation of use of limb	Pain/tenderness limiting use of limb OR Pain/tenderness causing greater than minimal interference with usual social & functional activities	Pain/tenderness causing inability to perform usual social & functional activities	Pain/tenderness causing inability to perform basic self-care function OR Hospitalization (other than emergency room visit) indicated for management of pain/tenderness
Injection site reaction (localized)				
**Adult > 15 years**	Erythema OR Induration of 5x5 cm – 9x9 cm (or 25 cm2 – 81cm2)	Erythema OR Induration OR Edema > 9 cm any diameter (or > 81 cm2)	Ulceration OR Secondary infection OR Phlebitis OR Sterile abscess OR Drainage	Necrosis (involving dermis and deeper tissue)
Pruritis associated with injection<br><br>See also Skin: Pruritis (itching - no skin lesions)	Itching localized to injection site AND Relieved spontaneously or with < 48 hours treatment	Itching beyond the injection site but not generalized OR Itching localized to injection site requiring ≥ 48 hours treatment	Generalized itching causing inability to perform usual social & functional activities	NA
**SKIN – DERMATOLOGICAL**				
Alopecia	Thinning detectable by study participant (or by caregiver for young children and disabled adults)	Thinning or patchy hair loss detectable by health care provider	Complete hair loss	NA
Cutaneous reaction – rash	Localized macular Rash	Diffuse macular, maculopapular, or morbilliform rash OR Target lesions	Diffuse macular, maculopapular, or morbilliform rash with vesicles or limited number of bullae OR Superficial ulcerations of mucous membrane limited to one site	Extensive or generalized bullous lesions OR Stevens-Johnson syndrome OR Ulceration of mucous membrane involving two or more distinct mucosal sites OR Toxic epidermal necrolysis (TEN)
Hyperpigmentation	Slight or localized	Marked or generalized	NA	NA
Hypopigmentation	Slight or localized	Marked or generalized	NA	NA
Pruritis (itching – no skin lesions)<br><br>(See also Injection Site Reactions: Pruritis associated with injection)	Itching causing no or minimal interference with usual social & functional activities	Itching causing greater than minimal interference with usual social & functional activities	Itching causing inability to perform usual social & functional activities	NA
**CARDIOVASCULAR**				
Cardiac arrhythmia (general) (By ECG or physical exam)	Asymptomatic AND No intervention Indicated	Asymptomatic AND Non-urgent medical intervention indicated	Symptomatic, non-lifethreatening AND Non-urgent Medical intervention indicated	Life-threatening arrhythmia OR Urgent intervention indicated
Cardiacischemia/ infarction	NA	NA	Symptomatic ischemia (stable angina) OR Testing consistent with ischemia	Unstable angina OR Acute myocardial infarction
Hemorrhage (significant acute blood loss)	NA	Symptomatic AND No transfusion indicated	Symptomatic AND Transfusion of ≤ 2 units packed RBCs indicated	Life-threatening hypotension OR Transfusion of > 2 units packed RBCs indicated
Hypertension				
**Adult > 17 years** (with repeat testing at same visit)	> 140 – 159 mmHg systolic OR > 90 – 99 mmHg Diastolic	> 160 – 179 mmHg systolic OR > 100 – 109 mmHg diastolic	> 180 mmHg systolic OR > 110 mmHg diastolic	Life-threatening consequences (e.g., malignant hypertension) OR Hospitalization indicated (other than emergency room visit)
Hypotension	NA	Symptomatic, corrected with oral	Symptomatic, IV fluids indicated	Shock requiring use of vasopressors or

CLINICAL				
**PARAMETER**	**GRADE 1 MILD**	**GRADE 2 MODERATE**	**GRADE 3 SEVERE**	**GRADE 4 POTENTIALLY LIFE-THREATENING**
		fluid replacement		mechanical assistance to maintain blood pressure
Pericardial effusion	Asymptomatic, small effusion requiring no Intervention	Asymptomatic, moderate or larger effusion requiring no intervention	Effusion with non-life threatening physiologic consequences OR Effusion with non-urgent intervention indicated	Life-threatening consequences (e.g., tamponade) OR Urgent intervention indicated
Prolonged PR interval				
**Adult > 16 years**	PR interval 0.21 – 0.25 sec	PR interval > 0.25 sec	Type II 2nd degree AV block OR Ventricular pause > 3.0 sec	Complete AV block
Prolonged QTc				
**Adult > 16 years**	Asymptomatic, QTc interval 0.45 – 0.47 sec OR Increase interval < 0.03 sec above baseline	Asymptomatic, QTc interval 0.48 – 0.49 sec OR Increase in interval 0.03 – 0.05 sec above baseline	Asymptomatic, QTc interval ≥ 0.50 sec OR Increase in interval ≥ 0.06 sec above baseline	Life-threatening consequences, e.g. Torsade de pointes or other associated serious ventricular dysrhythmia
Thrombosis/embolism	NA	Deep vein thrombosis AND No intervention indicated (e.g., anticoagulation, lysis filter, invasive procedure)	Deep vein thrombosis AND intervention indicated (e.g., anticoagulation, lysis filter, invasive procedure)	Embolic event (e.g., pulmonary embolism, life-threatening thrombus)
Vasovagal episode (associated with a procedure of any kind)	Present without loss of consciousness	Present with transient loss of consciousness	NA	NA
Ventricular dysfunction (congestive heart failure)	NA	Asymptomatic diagnostic finding AND intervention indicated	New onset with symptoms OR Worsening symptomatic congestive heart failure	Life-threatening congestive heart failure
**GASTROINTESTINAL**				
Anorexia	Loss of appetite without decreased oral intake	Loss of appetite associated with decreased oral intake without significant weight loss	Loss of appetite associated with significant weight loss	Life-threatening consequences OR Aggressive intervention indicated [e.g., tube feeding or total parenteral nutrition TPN)]
Ascites	Asymptomatic	Symptomatic AND Intervention indicated (e.g., diuretics or Therapeutic paracentesis)	Symptomatic despite intervention	Life-threatening consequences
Cholecystitis	NA	Symptomatic AND Medical intervention indicated	Radiologic, endoscopic or operative intervention indicated	Life-threatening consequences (e.g., sepsis or perforation)
Constipation	NA	Persistent constipation requiring regular use of dietary modifications, laxatives, or enemas	Obstipation with manual evacuation indicated	Life-threatening consequences (e.g., obstruction)
Diarrhoea	Transient or intermittent episodes of unformed stools OR Increase of ≤ 3 stools over baseline per 24-hour period	Persistent episodes of unformed to watery stools OR Increase of 4 – 6 stools over baseline per 24-hour period	Bloody diarrhea OR Increase of ≥ 7 stools per 24-hour period OR IV fluid replacement indicated	Life-threatening consequences (e.g., hypotensive shock)
Dysphagia-Odynophagia	Symptomatic but able to eat usual diet	Symptoms causing altered dietary intake without medical intervention indicated	Symptoms causing severely altered dietary intake with medical intervention indicated	Life-threatening reduction in oral intake
Mucositis/stomatitis (clinical exam)<br><br>Indicate site (e.g., larynx, oral)<br>See Genitourinary for Vulvovaginitis<br>See also Dysphagia-	Erythema of the Mucosa	Patchy pseudomembranes or ulcerations	Confluent pseudomembranes or ulcerations OR Mucosal bleeding with minor trauma	Tissue necrosis OR Diffuse spontaneous mucosal bleeding OR Life-threatening consequences (e.g., aspiration, choking)

CLINICAL				
**PARAMETER**	**GRADE 1 MILD**	**GRADE 2 MODERATE**	**GRADE 3 SEVERE**	**GRADE 4 POTENTIALLY LIFE-THREATENING**
Odynophagia and Proctitis				
Nausea	Transient (< 24 hours) or intermittent nausea with no or minimal interference with oral intake	Persistent nausea resulting in decreased oral intake for 24 – 48 hours	Persistent nausea resulting in minimal oral intake for > 48 hours OR Aggressive rehydration indicated (e.g., IV fluids)	Life-threatening consequences (e.g., hypotensive shock)
Pancreatitis	NA	Symptomatic AND Hospitalization not indicated (other than emergency room visit)	Symptomatic AND Hospitalization indicated (other than emergency room visit)	Life-threatening consequences (e.g., circulatory failure, hemorrhage, sepsis)
Proctitis (functionalsymptomatic)<br><br>Also see Mucositis/stomatitis for clinical exam	Rectal discomfort AND No intervention Indicated	Symptoms causing greater than minimal interference with usual social & functional activities OR Medical intervention indicated	Symptoms causing inability to perform usual social & functional activities OR Operative intervention indicated	Life-threatening consequences (e.g., perforation)
Vomiting	Transient or intermittent vomiting with no or minimal interference with oral intake	Frequent episodes of vomiting with no or mild dehydration	Persistent vomiting resulting in orthostatic hypotension OR Aggressive rehydration indicated (e.g., IV fluids)	Life-threatening consequences (e.g., hypotensive shock)
**NEUROLOGIC**				
Alteration in personality-behavior or in mood (e.g., agitation, anxiety, depression, mania, psychosis)	Alteration causing no or minimal interference with usual social & functional activities	Alteration causing greater than minimal interference with usual social & functional activities	Alteration causing inability to perform usual social & functional activities	Behavior potentially harmful to self or others (e.g., suicidal and homicidal ideation or attempt, acute psychosis) OR Causing inability to perform basic self-care functions
Altered Mental Status<br><br>For Dementia, see Cognitive and behavioral/attentional disturbance (including dementia and attention deficit disorder)	Changes causing no or minimal interference with usual social & functional activities	Mild lethargy or somnolence causing greater than minimal interference with usual social & functional activities	Confusion, memory impairment, lethargy, or somnolence causing inability to perform usual social & functional activities	Delirium OR obtundation, OR coma
Ataxia	Asymptomatic ataxia detectable on exam OR Minimal ataxia causing no or minimal interference with usual social & functional activities	Symptomatic ataxia causing greater than minimal interference with usual social & functional activities	Symptomatic ataxia causing inability to perform usual social & functional activities	Disabling ataxia causing inability to perform basic self-care functions
Cognitive and behavioral/attentional disturbance (including dementia and attention deficit disorder)	Disability causing no or minimal interference with usual social & functional activities OR Specialized resources not indicated	Disability causing greater than minimal interference with usual social & functional activities OR Specialized resources on part-time basis indicated	Disability causing inability to perform usual social & functional activities OR Specialized resources on a full-time basis indicated	Disability causing inability to perform basic self-care functions OR Institutionalization indicated
CNS ischemia (acute)	NA	NA	Transient ischemic attack	Cerebral vascular accident (CVA, stroke) with neurological deficit
Headache	Symptoms causing no or minimal interference with usual social & functional activities	Symptoms causing greater than minimal interference with usual social & functional activities	Symptoms causing inability to perform usual social & functional activities	Symptoms causing inability to perform basic self-care functions OR Hospitalization indicated (other than emergency room visit) OR Headache with significant impairment of alertness or other neurologic function
Insomnia	NA	Difficulty sleeping causing greater than	Difficulty sleeping causing inability to	Disabling insomnia causing inability to

CLINICAL				
PARAMETER	GRADE 1<br>MILD	GRADE 2<br>MODERATE	GRADE 3<br>SEVERE	GRADE 4<br>POTENTIALLY<br>LIFE-THREATENING
		minimal interference with usual social & functional activities	perform usual social & functional activities	perform basic self-care functions
Neuromuscular weakness (including myopathy & neuropathy)	Asymptomatic with decreased strength on exam OR Minimal muscle weakness causing no or minimal interference with usual social & functional activities	Muscle weakness causing greater than minimal interference with usual social & functional activities	Muscle weakness causing inability to perform usual social & functional activities	Disabling muscle weakness causing inability to perform basic self-care functions OR Respiratory muscle weakness impairing ventilation
Neurosensory alteration (including paresthesia and painful neuropathy)	Asymptomatic with sensory alteration on exam or minimal paresthesia causing no or minimal interference with usual social & functional activities	Sensory alteration or paresthesia causing greater than minimal interference with usual social & functional activities	Sensory alteration or paresthesia causing inability to perform usual social & functional activities	Disabling sensory alteration or paresthesia causing inability to perform basic self-care functions
Seizure: (new onset)<br>**– Adult ≥ 18 years**<br><br>See also Seizure: (known pre-existing Seizure disorder)	NA	1 seizure	2 – 4 seizures	Seizures of any kind which are prolonged, repetitive (e.g., status epilepticus), or difficult to control (e.g., refractory epilepsy)
Seizure: (known pre-existing seizure disorder)<br>**– Adult ≥ 18 years**<br><br>For worsening of existing epilepsy the grades should be based on an increase from previous level of control to any of these levels.	NA	Increased frequency of pre-existing seizures (non-repetitive) without change in seizure character OR Infrequent breakthrough seizures while on stable medication in a previously controlled seizure disorder	Change in seizure character from baseline either in duration or quality (e.g., severity or focality)	Seizures of any kind which are prolonged, repetitive (e.g., status epilepticus), or difficult to control (e.g., refractory epilepsy)
Syncope (not associated with a procedure)	NA	Present	NA	NA
Vertigo	Vertigo causing no or minimal interference with usual social & functional activities	Vertigo causing greater than minimal interference with usual social & functional activities	Vertigo causing inability to perform usual social & functional activities	Disabling vertigo causing inability to perform basic self-care functions
**RESPIRATORY**				
Bronchospasm (acute)	FEV1 or peak flow reduced to 70 – 80%	FEV1 or peak flow 50 – 69%	FEV1 or peak flow 25 – 49%	Cyanosis OR FEV1 or peak flow < 25% OR Intubation
Dyspnea or respiratory distress				
**Adult ≥ 14 years**	Dyspnea on exertion with no or minimal interference with usual social & functional activities	Dyspnea on exertion causing greater than minimal interference with usual social & functional activities	Dyspnea at rest causing inability to perform usual social & functional activities	Respiratory failure with ventilatory support indicated
**MUSCULOSKELETAL**				
Arthralgia<br><br>See also Arthritis	Joint pain causing no or minimal interference with usual social & functional activities	Joint pain causing greater than minimal interference with usual social & functional activities	Joint pain causing inability to perform usual social & functional activities	Disabling joint pain causing inability to perform basic self-care functions
Arthritis<br><br>See also Arthralgia	Stiffness or joint swelling causing no or minimal interference with usual social & functional activities	Stiffness or joint swelling causing greater than minimal interference with usual social & functional activities	Stiffness or joint swelling causing inability to perform usual social & functional activities	Disabling joint stiffness or swelling causing inability to perform basic self-care functions
Bone Mineral Loss				

CLINICAL				
**PARAMETER**	**GRADE 1 MILD**	**GRADE 2 MODERATE**	**GRADE 3 SEVERE**	**GRADE 4 POTENTIALLY LIFE-THREATENING**
**Adult ≥ 21 years**	BMD t-score -2.5 to -1.0	BMD t-score < -2.5	Pathological fracture (including loss of vertebral height)	Pathologic fracture causing life-threatening consequences
**Pediatric < 21 years**	BMD z-score -2.5 to -1.0	BMD z-score < -2.5	Pathological fracture (including loss of vertebral height)	Pathologic fracture causing life-threatening consequences
Myalgia (non-injection site)	Muscle pain causing no or minimal interference with usual social & functional activities	Muscle pain causing greater than minimal interference with usual social & functional activities	Muscle pain causing inability to perform usual social & functional activities	Disabling muscle pain causing inability to perform basic self-care functions
Osteonecrosis	NA	Asymptomatic with radiographic findings AND No operative intervention indicated	Symptomatic bone pain with radiographic findings OR Operative intervention indicated	Disabling bone pain with radiographic findings causing inability to perform basic self-care functions
**GENITOURINARY**				
Cervicitis (symptoms)<br><br>(For use in studies evaluating topical study agents)<br><br>For other cervicitis see Infection: Infection (any other than HIV infection)	Symptoms causing no or minimal interference with usual social & functional activities	Symptoms causing greater than minimal interference with usual social & functional activities	Symptoms causing inability to perform usual social & functional activities	Symptoms causing inability to perform basic self-care functions
Cervicitis (clinical exam)<br><br>(For use in studies evaluating topical study agents)<br><br>For other cervicitis see Infection: Infection (any other than HIV infection)	Minimal cervical abnormalities on examination (erythema, mucopurulent discharge, or friability) OR Epithelial disruption < 25% of total surface	Moderate cervical abnormalities on examination (erythema, mucopurulent discharge, or friability) OR Epithelial disruption of 25 – 49% total surface	Severe cervical abnormalities on examination (erythema, mucopurulent discharge, or friability) OR Epithelial disruption 50 – 75% total surface	Epithelial disruption > 75% total surface
Inter-menstrual bleeding (IMB)	Spotting observed by participant OR Minimal blood observed during clinical or colposcopic examination	Inter-menstrual bleeding not greater in duration or amount than usual menstrual cycle	Inter-menstrual bleeding greater in duration or amount than usual menstrual cycle	Hemorrhage with lifethreatening hypotension OR Operative intervention indicated
Urinary tract obstruction (e.g., stone)	NA	Signs or symptoms of urinary tract obstruction without hydronephrosis or renal dysfunction	Signs or symptoms of urinary tract obstruction with hydronephrosis or renal dysfunction	Obstruction causing life-threatening consequences
Vulvovaginitis (symptoms)<br><br>(Use in studies evaluating topical study agents)<br><br>For other vulvovaginitis see Infection: Infection (any other than HIV infection)	Symptoms causing no or minimal interference with usual social & functional activities	Symptoms causing greater than minimal interference with usual social & functional activities	Symptoms causing inability to perform usual social & functional activities	Symptoms causing inability to perform basic self-care functions
Vulvovaginitis (clinical exam)<br><br>(Use in studies evaluating topical study agents)<br><br>For other vulvovaginitis see Infection: Infection (any other than HIV infection)	Minimal vaginal abnormalities on examination OR Epithelial disruption < 25% of total surface	Moderate vaginal abnormalities on examination OR Epithelial disruption of 25 - 49% total surface	Severe vaginal abnormalities on examination OR Epithelial disruption 50 - 75% total surface	Vaginal perforation OR Epithelial disruption > 75% total surface

CLINICAL				
**PARAMETER**	**GRADE 1 MILD**	**GRADE 2 MODERATE**	**GRADE 3 SEVERE**	**GRADE 4 POTENTIALLY LIFE-THREATENING**
**OCULAR/VISUAL**				
Uveitis	Asymptomatic but detectable on exam	Symptomatic anterior uveitis OR Medical intervention indicated	Posterior or pan-uveitis OR Operative intervention indicated	Disabling visual loss in affected eye(s)
Visual changes (from baseline)	Visual changes causing no or minimal interference with usual social & functional activities	Visual changes causing greater than minimal interference with usual social & functional activities	Visual changes causing inability to perform usual social & functional activities	Disabling visual loss in affected eye(s)
**ENDOCRINE/METABOLIC**				
Abnormal fat accumulation (e.g., back of neck, Breasts, abdomen)	Detectable by study participant (or by caregiver for disabled adults)	Detectable on physical exam by health care provider	Disfiguring OR Obvious changes on casual visual inspection	NA
Diabetes mellitus	NA	New onset without need to initiate medication OR Modification of current medications to regain glucose control	New onset with initiation of medication indicated OR Diabetes uncontrolled despite treatment modification	Life-threatening consequences (e.g., ketoacidosis, hyperosmolar nonketotic coma)
Gynecomastia	Detectable by study participant or caregiver (for disabled adults)	Detectable on physical exam by health care provider	Disfiguring OR Obvious on casual visual inspection	NA
Hyperthyroidism	Asymptomatic	Symptomatic causing greater than minimal interference with usual social & functional activities OR Thyroid suppression therapy indicated	Symptoms causing inability to perform usual social & functional activities OR Uncontrolled despite treatment modification	Life-threatening consequences (e.g., thyroid storm)
Hypothyroidism	Asymptomatic	Symptomatic causing greater than minimal interference with usual social & functional activities OR Thyroid replacement therapy indicated	Symptoms causing inability to perform usual social & functional activities OR Uncontrolled despite treatment modification	Life-threatening consequences (e.g., myxedema coma)
Lipoatrophy (e.g., fat loss from the face, extremities, buttocks)	Detectable by study participant (or by caregiver for disabled adults)	Detectable on physical exam by health care provider	Disfiguring OR Obvious on casual visual inspection	NA

LABORATORY				
**PARAMETER**	**GRADE 1 MILD**	**GRADE 2 MODERATE**	**GRADE 3 SEVERE**	**GRADE 4 POTENTIALLY LIFE-THREATENING**
**HEMATOLOGY**	***Standard International Units are listed in italics***			
Absolute neutrophil count (ANC)	1,000 –< 1,3000/mm^3<br>*1.00 x 10^9–1.30. x 10^9/L*	750 – 999/mm^3<br>*0.750 x 10^9 – 0.999 x 10^9/L*	500 – 749/mm^3<br>*0.50 x 10^9 – 0.749 x 10^9/L*	< 500/mm^3<br>*< 0.500 x 10^9/L*
Fibrinogen, decreased	100 – 200 mg/dL<br>*1.00 – 2.00 g/L*<br>OR<br>0.75 – 0.99 x LLN	75 – 99 mg/dL<br>*0.75 – 0.99 g/L*<br>OR<br>0.50 – 0.74 x LLN	50 – 74 mg/dL<br>*0.50 – 0.74 g/L*<br>OR<br>0.25 – 0.49 x LLN	< 50 mg/dL<br>*< 0.50 g/L*<br>OR<br>< 0.25 x LLN<br>OR<br>Associated with gross Bleeding
Haemoglobin (Hb)	10.0 – 10.9 g/dL<br>*1.55 – 1.69 mmol/L*	9.0 – 9.9 g/dL<br>*1.40 – 1.54 mmol/L*	7.0 – 8.9 g/dL<br>*1.09 – 1.39 mmol/L*	< 7.0 g/dL<br>*< 1.09 mmol/L*
International Normalized Ratio of prothrombin time (INR)	1.1 – 1.5 x ULN	1.6 – 2.0 x ULN	2.1 – 3.0 x ULN	> 3.0 x ULN
Methemoglobin	5.0 – 10.0%	10.1 – 15.0%	15.1 – 20.0%	> 20.0%
Prothrombin Time (PT)	1.1 – 1.25 x ULN	1.26 – 1.50 x ULN	1.51 – 3.00 x ULN	> 3.00 x ULN
Partial Thromboplastin Time (PTT)	1.1 – 1.66 x ULN	1.67 – 2.33 x ULN	2.34 – 3.00 x ULN	> 3.00 x ULN
Platelets, decreased	100,000 –	50,000 –	25,000 –	< 25,000/mm^3

LABORATORY				
**PARAMETER**	**GRADE 1 MILD**	**GRADE 2 MODERATE**	**GRADE 3 SEVERE**	**GRADE 4 POTENTIALLY LIFE-THREATENING**
	124,999/mm^3<br>*100.000 x 10^9 – 124.999 x 10^9/L*	99,999/mm^3<br>*50.000 x 10^9 – 99.999 x 10^9/L*	49,999/mm^3<br>*25.000 x 10^9 – 49.999 x 10^9/L*	*< 25.000 x 10^9/L*
WBC, decreased	2,000 – 2,500/mm^3<br>*2.000 x 10^9 – 2.500 x 10^9/L*	1,500 – 1,999/mm^3<br>*1.500 x 10^9 – 1.999 x 10^9/L*	1,000 – 1,499/mm^3<br>*1.000 x 10^9 – 1.499 x 10^9/L*	< 1,000/mm^3<br>*< 1.000 x 10^9/L*
**CHEMISTRIES**	***Standard International Units are listed in italics***			
Acidosis	NA	pH < normal, but ≥ 7.3	pH < 7.3 without life-threatening consequences	pH < 7.3 with life-threatening consequences
Albumin, serum, low	3.0 g/dL – < LLN<br>*30 g/L – < LLN*	2.0 – 2.9 g/dL<br>*20 – 29 g/L*	< 2.0 g/dL<br>*< 20 g/L*	NA
Alkaline Phosphatase	1.25 – 2.5 x ULN†	2.6 – 5.0 x ULN†	5.1 – 10.0 x ULN†	> 10.0 x ULN†
Alkalosis	NA	pH > normal, but ≤ 7.5	pH > 7.5 without life-threatening consequences	pH > 7.5 with life-threatening consequences
ALT (SGPT)	1.25 – 2.5 x ULN	2.6 – 5.0 x ULN	5.1 – 10.0 x ULN	> 10.0 x ULN
AST (SGOT)	1.25 – 2.5 x ULN	2.6 – 5.0 x ULN	5.1 – 10.0 x ULN	> 10.0 x ULN
Bicarbonate, serum, low	16.0 mEq/L – < LLN<br>*16.0 mmol/L – < LLN*	11.0 – 15.9 mEq/L<br>*11.0 – 15.9 mmol/L*	8.0 – 10.9 mEq/L<br>*8.0 – 10.9 mmol/L*	< 8.0 mEq/L<br>*< 8.0 mmol/L*
Bilirubin	1.1 – 1.5 x ULN	1.6 – 2.5 x ULN	2.6 – 5.0 x ULN	> 5.0 x ULN
Calcium, serum, high (corrected for albumin)	10.6 – 11.5 mg/dL<br>*2.65 – 2.88 mmol/L*	11.6 – 12.5 mg/dL<br>*2.89 – 3.13 mmol/L*	12.6 – 13.5 mg/dL<br>*3.14 – 3.38 mmol/L*	> 13.5 mg/dL<br>*> 3.38 mmol/L*
Calcium, serum, low (corrected for albumin)	7.8 – 8.4 mg/dL<br>*1.95 – 2.10 mmol/L*	7.0 – 7.7 mg/dL<br>*1.75 – 1.94 mmol/L*	6.1 – 6.9 mg/dL<br>*1.53 – 1.74 mmol/L*	< 6.1 mg/dL<br>*< 1.53 mmol/L*
Cardiac troponin I (cTnI)	NA	NA	NA	Levels consistent with myocardial infarction or unstable angina as defined by the manufacturer
Cardiac troponin T (cTnT)	NA	NA	NA	≥ 0.20 ng/mL<br>OR<br>Levels consistent with myocardial infarction or unstable angina as defined by the manufacturer
Cholesterol (fasting)				
**Adult ≥ 18 years**	200 – 239 mg/dL<br>*5.18 – 6.19 mmol/L*	240 – 300 mg/dL<br>*6.20 – 7.77 mmol/L*	> 300 mg/dL<br>*> 7.77 mmol/L*	NA
Creatine Kinase	3.0 – 5.9 x ULN†	6.0 – 9.9 x ULN†	10.0 – 19.9 x ULN†	≥ 20.0 x ULN†
Creatinine	1.1 – 1.3 x ULN†	1.4 – 1.8 x ULN†	1.9 – 3.4 x ULN†	≥ 3.5 x ULN†
Glucose, serum, high				
Nonfasting	116 – 160 mg/dL<br>*6.44 – 8.88 mmol/L*	161 – 250 mg/dL<br>*8.89 – 13.88 mmol/L*	251 – 500 mg/dL<br>*13.89 – 27.75 mmol/L*	> 500 mg/dL<br>*> 27.75 mmol/L*
Fasting	110 – 125 mg/dL<br>*6.11 – 6.94 mmol/L*	126 – 250 mg/dL<br>*6.95 – 13.88 mmol/L*	251 – 500 mg/dL<br>*13.89 – 27.75 mmol/L*	> 500 mg/dL<br>*> 27.75 mmol/L*
Glucose, serum, low	55 – 64 mg/dL<br>*3.05 – 3.55 mmol/L*	40 – 54 mg/dL<br>*2.22 – 3.06 mmol/L*	30 – 39 mg/dL<br>*1.67 – 2.23 mmol/L*	< 30 mg/dL<br>*< 1.67 mmol/L*
Lactate	< 2.0 x ULN without Acidosis	≥ 2.0 x ULN without acidosis	Increased lactate with pH < 7.3 without life-threatening consequences	Increased lactate with pH < 7.3 with life-threatening consequences
LDL cholesterol (fasting)				
**Adult ≥ 18 years**	130 – 159 mg/dL<br>*3.37 – 4.12 mmol/L*	160 – 190 mg/dL<br>*4.13 – 4.90 mmol/L*	≥ 190 mg/dL<br>*≥ 4.91 mmol/L*	NA
Lipase	1.1 – 1.5 x ULN	1.6 – 3.0 x ULN	3.1 – 5.0 x ULN	> 5.0 x ULN
Magnesium, serum, low	1.2 – 1.4 mEq/L<br>*0.60 – 0.70 mmol/L*	0.9 – 1.1 mEq/L<br>*0.45 – 0.59 mmol/L*	0.6 – 0.8 mEq/L<br>*0.30 – 0.44 mmol/L*	< 0.60 mEq/L<br>*< 0.30 mmol/L*
Pancreatic amylase	1.1 – 1.5 x ULN	1.6 – 2.0 x ULN	2.1 – 5.0 x ULN	> 5.0 x ULN
Phosphate, serum, low				
**Adult and Pediatric > 14 years**	2.5 mg/dL – < LLN<br>*0.81 mmol/L – < LLN*	2.0 – 2.4 mg/dL<br>*0.65 – 0.80 mmol/L*	1.0 – 1.9 mg/dL<br>*0.32 – 0.64 mmol/L*	< 1.00 mg/dL<br>*< 0.32 mmol/L*
Potassium, serum, high	5.6 – 6.0 mEq/L<br>*5.6 – 6.0 mmol/L*	6.1 – 6.5 mEq/L<br>*6.1 – 6.5 mmol/L*	6.6 – 7.0 mEq/L<br>*6.6 – 7.0 mmol/L*	> 7.0 mEq/L<br>*> 7.0 mmol/L*
Potassium, serum, low	3.0 – 3.4 mEq/L<br>*3.0 – 3.4 mmol/L*	2.5 – 2.9 mEq/L<br>*2.5 – 2.9 mmol/L*	2.0 – 2.4 mEq/L<br>*2.0 – 2.4 mmol/L*	< 2.0 mEq/L<br>*< 2.0 mmol/L*
Sodium, serum, high	146 – 150 mEq/L	151 – 154 mEq/L	155 – 159 mEq/L	≥ 160 mEq/L

LABORATORY				
**PARAMETER**	**GRADE 1 MILD**	**GRADE 2 MODERATE**	**GRADE 3 SEVERE**	**GRADE 4 POTENTIALLY LIFE-THREATENING**
	146 – 150 mmol/L	*151 – 154 mmol/L*	*155 – 159 mmol/L*	*≥ 160 mmol/L*
Sodium, serum, low	130 – 135 mEq/L<br>*130 – 135 mmol/L*	125 – 129 mEq/L<br>*125 – 129 mmol/L*	121 – 124 mEq/L<br>*121 – 124 mmol/L*	≤ 120 mEq/L<br>*≤ 120 mmol/L*
Triglycerides (fasting)	NA	500 – 750 mg/dL<br>*5.65 – 8.48 mmol/L*	751 – 1,200 mg/dL<br>*8.49 – 13.56 mmol/L*	> 1,200 mg/dL<br>*> 13.56 mmol/L*
Uric acid	7.5 – 10.0 mg/dL<br>*0.45 – 0.59 mmol/L*	10.1 – 12.0 mg/dL<br>*0.60 – 0.71 mmol/L*	12.1 – 15.0 mg/dL<br>*0.72 – 0.89 mmol/L*	> 15.0 mg/dL<br>*> 0.89 mmol/L*
**URINALYSIS**	***Standard International Units are listed in italics***			
Hematuria (microscopic)	6 – 10 RBC/HPF	> 10 RBC/HPF	Gross, with or without clots OR with RBC casts	Transfusion indicated
Proteinuria, random collection	1 +	2 – 3 +	4 +	NA
Proteinuria, 24 hour collection	200 – 999 mg/24 h<br>*0.200 – 0.999 g/d*	1,000 – 1,999 mg/24 h<br>*1.000 – 1.999 g/d*	2,000 – 3,500 mg/24 h<br>*2.000 – 3.500 g/d*	> 3,500 mg/24 h<br>*> 3.500 g/d*

Reference: Division of AIDS table for grading the severity of adult adverse events, December 2004.

APPENDIX 11: MEDICAL OUTCOMES STUDY-HIV HEALTH SURVEY

This is the English language template for the MOS-HIV Health Survey, provided for background information. A version translated into the local language(s) will be provided as a separate document for review alongside this protocol for national ethics and regulatory authorities.

MOS – HIV QUALITY OF LIFE QUESTIONNAIRE

Date :	Clinic no:	Initials:	Date of birth:	Trial no:

INSTRUCTIONS

1. Please answer every question even though some questions may seem very similar to others.
2. Answer by placing a tick in the appropriate box. If you feel that the answer lies in between one possible response and another, please mark whichever box comes closest to the way you feel.
3. If you don't understand what a question means, please ask the Trial Nurse to explain or clarify it.

THANK YOU FOR COMPLETING THIS QUESTIONNAIRE YOUR COOPERATION WILL BENEFIT YOURSELF, OTHER PEOPLE AND RESEARCH INTO HIV DISEASE.

1. In general, would you say your health is: (Please tick ONE box)

☐ Excellent ☐ Very Good ☐ Good ☐ Fair ☐ Poor

2. How much **bodily** pain have you generally had during **the past 4 weeks?** (Please tick ONE box)

☐ None ☐ Very Mild ☐ Mild ☐ Moderate ☐ Severe ☐ Very Severe

3. During **the past 4 weeks**, how much did **pain** interfere with your normal work (including both work outside the home and housework)? (Please tick ONE box)

☐ Not at all ☐ A little bit ☐ Moderately ☐ Quite a bit ☐ Extremely

4. The following questions are about activities you might do during a typical day. Does your <u>health now limit you</u> in these activities? If so, how much? (Please tick ONE box on each line)

	YES, Limited A Lot	YES, Limited A Little	NO, Not Limited At All
a. The kinds or amounts of **vigorous** activities you can do, like lifting heavy objects, running or participating in strenuous sports.	☐	☐	☐
b. The kinds or amounts of **moderate** activities you can do, like moving a table, carrying groceries or bowling.	☐	☐	☐
c. Walking uphill or climbing a few flights of stairs.	☐	☐	☐
d. Bending, lifting or stooping.	☐	☐	☐
e. Walking one hundred yards.	☐	☐	☐
f. Easting, dressing, bathing, or using the toilet.	☐	☐	☐

5. Does your health **keep** you from working at a job, doing work around the house or going to school? (Please tick ONE box)

☐ Yes ☐ No

6. Have you been unable to do **certain kinds or amounts** of work, housework, or schoolwork because of your health? (Please tick ONE box)

☐ Yes ☐ No

For each of the following questions, please tick the box for the one answer that comes closest to the way you have been feeling during the past 4 weeks. (Please tick ONE box on each line)

	All of the Time	Most of the Time	A Good Bit of the Time	Some of the Time	A Little of the Time	None of the Time
7. How much of the time, during the past 4 weeks, has your **health limited your social activities** (like visiting with friends or close relatives)?	☐	☐	☐	☐	☐	☐
8. How much of the time, during the past 4 weeks:	☐	☐	☐	☐	☐	☐
a. Have you been a very nervous person?	☐	☐	☐	☐	☐	☐
b. Have you felt calm and peaceful?	☐	☐	☐	☐	☐	☐
c. Have you felt downhearted and low?	☐	☐	☐	☐	☐	☐
d. Have you been a happy person?	☐	☐	☐	☐	☐	☐
e. Have you felt so down in the dumps that nothing could cheer you up?	☐	☐	☐	☐	☐	☐
**9. How often during the past four weeks:**						
a. Did you feel full of life?	☐	☐	☐	☐	☐	☐
b. Did you feel worn out?	☐	☐	☐	☐	☐	☐
c. Did you feel tired?	☐	☐	☐	☐	☐	☐
d. Did you have enough energy to do the things you wanted to do?	☐	☐	☐	☐	☐	☐
e. Did you feel weighed down by your health problems?	☐	☐	☐	☐	☐	☐
f. Were you discouraged by your health problems?	☐	☐	☐	☐	☐	☐
g. Did you feel despair over your health problems?	☐	☐	☐	☐	☐	☐
h. Were you afraid because of your health?	☐	☐	☐	☐	☐	☐
10. How much of the time, during the **past 4 weeks:**						
a. Did you have difficulty reasoning and solving problems, for example, making plans, making decisions, learning new things?	☐	☐	☐	☐	☐	☐
b. Did you forget things that happened recently, for example, where you put things and when you had appointments?	☐	☐	☐	☐	☐	☐
c. Did you have trouble keeping your attention on any activity for long?	☐	☐	☐	☐	☐	☐
d. Did you have difficulty doing activities involving concentration and thinking?	☐	☐	☐	☐	☐	☐

(Please tick ONE box on each line)

	Definitely True	Mostly True	Don't Know	Mostly False	Definitely False
11. Please tick the box that best describes whether each of the following statements is true or false for you.	☐	☐	☐	☐	☐
a. I am somewhat ill.	☐	☐	☐	☐	☐
b. I am as healthy as anybody I know.	☐	☐	☐	☐	☐
c. My health is excellent.	☐	☐	☐	☐	☐
d. I have been feeling bad lately.	☐	☐	☐	☐	☐

12. How has the quality of your life been during the **past 4 weeks**? That is, how have things been going for you?
(Please tick ONE box)

Very well; could hardly be better	☐
Pretty good	☐
Good and bad parts about equal	☐
Pretty bad	☐
Very bad; could hardly be worse	☐

13. How would you rate your physical health and emotional condition now compared to **4 weeks ago**?
(Please tick ONE box)

Much better	☐
A little better	☐
About the same	☐
A little worse	☐
Much worse	☐

APPENDIX 12: EQ-5D

This is the English language template for the EQ--5D, provided for background information. A version translated into the local language(s) will be provided as a separate document for review alongside this protocol for national ethics and regulatory authorities.

Under each heading, please tick the ONE box that best describes your health TODAY.

MOBILITY
I have no problems in walking about ☐
I have slight problems in walking about ☐
I have moderate problems in walking about ☐
I have severe problems in walking about ☐
I am unable to walk about ☐

SELF-CARE
I have no problems washing or dressing myself ☐
I have slight problems washing or dressing myself ☐
I have moderate problems washing or dressing myself ☐
I have severe problems washing or dressing myself ☐
I am unable to wash or dress myself ☐

USUAL ACTIVITIES *(e.g. work, study, housework, family or leisure activities)*
I have no problems doing my usual activities ☐
I have slight problems doing my usual activities ☐
I have moderate problems doing my usual activities ☐
I have severe problems doing my usual activities ☐
I am unable to do my usual activities ☐

PAIN / DISCOMFORT
I have no pain or discomfort ☐
I have slight pain or discomfort ☐
I have moderate pain or discomfort ☐
I have severe pain or discomfort ☐
I have extreme pain or discomfort ☐

ANXIETY / DEPRESSION
I am not anxious or depressed ☐
I am slightly anxious or depressed ☐
I am moderately anxious or depressed ☐
I am severely anxious or depressed ☐
I am extremely anxious or depressed ☐

Sample

2

UK (English) © 2009 EuroQol Group EQ-5D™ is a trade mark of the EuroQol Group

APPENDIX 13: PHARMACOKINETICS OF TRIAL DRUGS AND REGIMENS

Arm A: rifampicin (standard dose), isoniazid, pyrazinamide, ethambutol
Rifampicin is an inducer as well as substrate of several metabolic mechanisms including CYP3A4 and P-glycoprotein. Rifampicin auto induces its metabolism, resulting in a 20–40% fall in exposure over the first 2 weeks of therapy (Davies, 2008). Isoniazid is metabolised in the liver, with the rate of elimination determined by the individual's genetically determined acetylation phenotype (Iseman, 2000). Both pyrazinamide and ethambutol are metabolised in the liver, but are unaffected by cytochrome enzymes (Arbex, 2010). The pharmacokinetics of the first-line anti-tuberculosis drugs, rifampicin, isoniazid, pyrazinamide and ethambutol have been delineated in a number of patient population groups (Tostmann, 2013; Babalik, 2013), and has also been studied in relation to treatment response (Burhan, 2013).

Arm B: rifampicin (35mg/kg), isoniazid, pyrazinamide, ethambutol, linezolid
Increases in rifampicin dose result in concentrations that are more than dose proportional; specifically, a 3-fold increase in dose from 10 to 30 mg/kg daily results in a 7-fold increase in the $AUC_{0–24}$ (Ruslami, 2007). Linezolid is metabolised by oxidation in liver (independent of cytochrome P_{450} enzymes), and therefore is not expected to have PK effects on the first-line drugs. A PK study demonstrated that the serum concentrations of linezolid obtained following each 300mg administration twice daily are well above the MIC, i.e. 0.125-0.5 mgL^{-1} against M.tb and that the serum concentration-time curve over 24h/MIC ratios were sufficiently high (>100) to predict efficacy (in 7 out of 8 patients studied) (Alffenaar, 2010).

Possible intra-regimen drug interactions:
The effect of rifampicin on the pharmacokinetics of linezolid was studied in sixteen healthy adult male volunteers administered linezolid 600mg twice daily for 2.5 days with and without rifampicin 600 mg once daily for 8 days. Rifampicin decreased the linezolid C_{max} and AUC by a mean 21% and a mean 32%, respectively, possibly by P-glycoprotein induction (Gandelman, 2011; Egle, 2005). The linezolid dose we use (600mg once daily) gives levels that are approximately 20 times the MIC (McGee, 2009; Dietze, 2008) and this rifampicin effect is therefore unlikely to impair linezolid sterilising efficacy. Using a higher dose of rifampicin may paradoxically increase activity of linezolid at the 600mg dose (Drusano, 2014).

Arm C: rifampicin (35mg/kg), isoniazid, pyrazinamide, ethambutol, clofazimine
PK studies of clofazimine demonstrate a prolonged lag time for absorption, high variability in bioavailability and clearance, and a terminal half-life of 70 days. Pharmacokinetic studies of clofazimine demonstrate a prolonged lag time for absorption, high variability in bioavailability and clearance, and a terminal half-life of 70 days (Holdiness, 1989).

Possible intra-regimen drug interactions:
Clofazimine is a weak inhibitor of CYP3A4 (Cholo, 2012) but to date PK studies have shown no impact of clofazimine on rifampicin (600mg dose) levels (Venkatesan, 1986). Clofazimine delayed the time to reach its peak plasma concentration but this had no effect on the AUC (O'Connor, 1995).

Arm D: rifapentine, isoniazid, pyrazinamide, linezolid, levofloxacin
Rifapentine induces metabolism by the cytochrome P_{450} system and P-glycoprotein transport system, although to a lesser extent than rifampicin (Baciewicz, 2013). Population PK modelling of rifapentine has found bioavailability decreased linearly with increasing dose, clearance was time but not concentration-dependent, and steady state may not yet have been achieved after 2 weeks of daily dosing because auto induction of clearance was increasing up to that point (Savic 2014; Dooley, 2012).

Fluoroquinolones are P-glycoprotein substrates. Levofloxacin PK (Peloquin, 2008) has been studied in patients with pulmonary tuberculosis.

Possible intra-regimen drug interactions:
Moxifloxacin exposure is not affected by rifapentine (Zvada, 2012) and it is therefore unlikely that levofloxacin would be affected although this has not been tested directly. Although rifampicin decreases linezolid C_{max} and AUC by 21% and 32% respectively, possibly by P-glycoprotein induction (Gandelman, 2011; Egle, 2005) the effect is likely to be less marked with rifapentine given its more moderate effects on P-glycoprotein. Furthermore, as with the regimen containing rifampicin and linezolid above, the linezolid dose we use (600mg once daily) gives levels that are approximately 20 times the MIC and so this interaction is unlikely to impair sterilising activity (McGee, 2009; Dietze, 2014).

Arm E: isoniazid, pyrazinamide, ethambutol, linezolid, bedaquiline
Bedaquiline is metabolized primarily by the cytochrome P_{450} isoenzyme 3A4 (CYP3A4) to a less-active N-monodesmethyl metabolite. Bedaquiline is well absorbed following oral administration of single and multiple doses, reaching its maximum plasma concentration (Cmax) 4–6 h after administration, irrespective of the dose. Bedaquiline has a long terminal elimination half-life in plasma of 164 days (van Heeswijk, 2014).

Possible intra-regimen drug interactions:
PK studies of bedaquiline with isoniazid/pyrazinamide and ethambutol found no significant changes in exposure of any of the drugs (van Heeswijk, 2014). There have been no drug interaction studies between linezolid and bedaquiline, but there is no reason to expect there to be an issue (bedaquiline is metabolized primarily by cytochrome P_{450} isoenzyme 3A4 which is not affected by linezolid; linezolid is metabolised mainly by oxidation in the liver and to some extent by P-glycoprotein, neither of which are affected by bedaquiline) (van Heeswijk, 2014).

References:
Alffenaar JW, van Altena R, Harmelink IM, Filguera P, Molenaar E, Wessels AM, et al. Comparison of the pharmacokinetics of two dosage regimens of linezolid in multidrug-resistant and extensively drug-resistant tuberculosis patients. *Clinical pharm* 2010; **49**(8): 559-65.
Arbex MA, Varella Mde C, Siqueira HR, Mello FA. Antituberculosis drugs: drug interactions, adverse effects, and use in special situations. Part 1: first-line drugs*. J Bras Pneumol*. 2010 Sep-Oct;36(5):626-40.
Babalik A, Ulus IH, Bakirci N, Kuyucu T, Arpag H, Dagyildiz L, et al. Pharmacokinetics and serum concentrations of antimycobacterial drugs in adult Turkish patients. *The international journal of tuberculosis and lung disease : the official journal of the International Union against Tuberculosis and Lung Disease* 2013; **17**(11): 1442-7.
Baciewicz AM, Chrisman CR, Finch CK, Self TH. Update on rifampin, rifabutin, and rifapentine drug interactions. *Current medical research and opinion* 2013; **29**(1): 1-12.
Burhan E, Ruesen C, Ruslami R, Ginanjar A, Mangunnegoro H, Ascobat P, et al. Isoniazid, rifampin, and pyrazinamide plasma concentrations in relation to treatment response in Indonesian pulmonary tuberculosis patients. *AAC* 2013; **57**(8): 3614-9.
Cholo MC, Steel HC, Fourie PB, Germishuizen WA, Anderson R. Clofazimine: current status and future prospects. *The Journal of Antimicrobial Chemotherapy* 2012; 67(2): 290-8.
Davies GR, Nuermberger EL. Pharmacokinetics and pharmacodynamics in the development of anti-tuberculosis drugs. *Tuberculosis* (Edinb). 2008 Aug;88 Suppl 1:S65-74.
Dietze R, Hadad DJ, McGee B, Molino LP, Maciel EL, Peloquin CA, et al. Early and extended early bactericidal activity of linezolid in pulmonary tuberculosis. *AJRCCM* 2008; **178**(11): 1180-5.

Dooley KE, Bliven-Sizemore EE, Weiner M, Lu Y, Nuermberger EL, Hubbard WC, et al. Safety and pharmacokinetics of escalating daily doses of the antituberculosis drug rifapentine in healthy volunteers. *Clinical pharmacology and therapeutics* 2012; **91**(5): 881-8.
Drusano GL, Neely M, Van Guilder M, Schumitzky A, Brown D, Fikes S, et al. Analysis of combination drug therapy to develop regimens with shortened duration of treatment for tuberculosis. *PloS one* 2014; **9**(7): e101311.
Egle H, Trittler R, Kummerer K, Lemmen SW. Linezolid and rifampin: Drug interaction contrary to expectations? *Clinical pharmacology and therapeutics* 2005; **77**(5): 451-3.
Gandelman K, Zhu T, Fahmi OA, Glue P, Lian K, Obach RS, et al. Unexpected effect of rifampin on the pharmacokinetics of linezolid: in silico and in vitro approaches to explain its mechanism. *Journal of clinical pharmacology* 2011; **51**(2): 229-36.
Holdiness MR. Clinical pharmacokinetics of clofazimine. A review. *Clinvpharm* 1989; **16**(2): 74-85.
Iseman MD, editor. A clinician's guide to tuberculosis. Philadelphia, Pa: Lippincott Williams and Wilkins; 2000
McGee B, Dietze R, Hadad DJ, Molino LP, Maciel EL, Boom WH, et al. Population pharmacokinetics of linezolid in adults with pulmonary tuberculosis. *AAC* 2009; **53**(9): 3981-4.
O'Connor R, O'Sullivan JF, O'Kennedy R. The pharmacology, metabolism, and chemistry of clofazimine. *Drug metabolism reviews* 1995; 27(4): 591-614.
Peloquin CA, Hadad DJ, Molino LP, Palaci M, Boom WH, Dietze R, et al. Population pharmacokinetics of levofloxacin, gatifloxacin, and moxifloxacin in adults with pulmonary tuberculosis. *AAC* 2008; **52**(3): 852-7.
Ruslami R, Nijland HM, Alisjahbana B, Parwati I, van Crevel R, Aarnoutse RE. Pharmacokinetics and tolerability of a higher rifampin dose versus the standard dose in pulmonary tuberculosis patients. *AAC* 2007; **51**(7): 2546-51.
Tostmann A, Mtabho CM, Semvua HH, van den Boogaard J, Kibiki GS, Boeree MJ, et al. Pharmacokinetics of first-line tuberculosis drugs in Tanzanian patients. *Antimicrobial agents and chemotherapy* 2013; **57**(7): 3208-13.
van Heeswijk RP, Dannemann B, Hoetelmans RM. Bedaquiline: a review of human pharmacokinetics and drug-drug interactions. *The Journal of antimicrobial chemotherapy* 2014; **69**(9): 2310-8.
Venkatesan K, Mathur A, Girdhar BK, Bharadwaj VP. The effect of clofazimine on the pharmacokinetics of rifampicin and dapsone in leprosy. *The Journal of Antimicrobial Chemotherapy* 1986; 18(6): 715-8.
Zvada SP, Denti P, Geldenhuys H, Meredith S, van As D, Hatherill M, et al. Moxifloxacin population pharmacokinetics in patients with pulmonary tuberculosis and the effect of intermittent high-dose rifapentine. *Antimicrobial agents and chemotherapy* 2012; **56**(8): 4471-3.

APPENDIX 14: TEMPLATE FOR PHARMACOKINETIC SUB-STUDY CONSENT FORM

Consent Form for Pharmacokinetic sub-study of the TRUNCATE-TB Trial
[Each site to insert version number and date of consent form here]

Two-month Regimens Using Novel Combinations to Augment Treatment Effectiveness for drug-sensitive Tuberculosis (TB): a randomised controlled non-inferiority trial (TRUNCATE-TB)-Pharmacokinetic sub-study

[Each site to use its own consent form, translated into local language(s) and presented on local headed paper with any additional cover sheet information required by local IRB/Ethics Committee; any modifications to the text must be discussed with the trial coordinating centre prior to submission to the IRB/Ethics Committee]

Invitation

You are being invited to take part in the Pharmacokinetic (PK) sub-study of the TRUNCATE-TB trial. Only people taking part in the TRUNCATE-TB trial will be invited to take part in this sub-study. Participation is entirely voluntary. Your decision to take part, or not, in this additional research, will not affect your participation in the main TRUNCATE-TB trial. Refusal to take part will not affect your access to treatment in any way. If you take part, you may decide later to withdraw from the study at any time without giving a reason and without affecting your future care. If you withdraw from the study you will continue to receive your TB medications and care as part of the TRUNCATE-TB trial.

Before you decide to participate, it is important that you understand why the research is being done and what it will involve. Please read the following information carefully and discuss it with others if you wish. Please ask us if anything is not clear or if you have any other questions. If you decide to take part in this sub-study, you will be asked to sign and date this document to confirm that you understand the procedures and agree to take part. You will be given a copy of this form to keep.

What is the purpose of the study?

Pharmacokinetics (PK) is the study of what the body does to a drug. The purpose of this sub-study is to gather information about the amount of the medicines in the bodies of TB patients taking the drugs in the TRUNCATE-TB treatment groups and also how these drugs affect each other in combination. This information will help to improve TB drug combinations and

treatment, thereby benefiting future patients with TB. This additional research will add information to the TRUNCATE-TB study, in which you are already taking part.

Who can take part?

If you are taking all the study medicines assigned in your treatment group (or at least at half of the doses you started on) and are willing to participate in the PK sub-study, you may take part. If you are taking any other medicines that may interact with the study medicines in your group, or if your bloods tests show that your kidney or liver tests are abnormal, or if you have a condition that may interfere with how your medicines are absorbed, you cannot join the PK sub-study.

How many patients will take part?

The PK sub-study aims to enroll between 12 and 16 patients from each of the treatment groups (up to 80 patients in total).

What will happen if I take part in the PK sub-study?

The duration of the PK sub-study is one day. This will take place at 8 weeks from when you started the medicines in your TRUNCATE-TB group. The PK study tests may be done on the same day as the week 8 TRUNCATE-TB trial study visit, but may also be done on a different day if more convenient. If the PK study is not done on the same day as the week 8 TRUNCATE-TB trial study visit then you will also be asked some questions on your symptoms, if you are taking other medications, and a brief physical examination will be performed.

What will happen at the PK sub-study visit?

You will be requested to avoid strenuous exercise and the use of alcohol, grapefruit juice, over-the-counter medicines (including antacids), vitamins or mineral supplements, herbal medications, recreational drugs or other medicinal products for 48 hours prior to the PK study day. You will be requested to fast from midnight on the evening prior to the PK study day (water allowed freely) and to not take your dose of medicines on the morning of the PK study day.

You will need to arrive at the study centre early in the morning and may need to stay overnight until the following morning / stay until the evening (12 hours) and return for a brief visit the following morning [site to modify according to local arrangements]. It is important NOT to take any medicines in the morning of the day when you have your appointment at the study centre. You will be given your TB treatment at the study centre. If you are receiving either Arms A, B or C you will take the study medications with water on an empty stomach, and 2 hours after will

receive a full breakfast. If you are receiving either Arms D or E you will take the study medications with a full breakfast. You will then be given a full lunch, and subsequent snacks and drinks as required. Water may be consumed freely throughout the study day.

At the beginning of the day a cannula (a flexible plastic needle) will be inserted into a vein in your arm, through which the blood samples can be drawn. A blood sample will be drawn before you are given your TB medicine, and then every few hours until the end of the day. The final sample will be drawn the following morning. If you do not stay overnight, then the cannula will be removed and final sample the following morning will need to be drawn using a needle in the usual way [site to delete previous phrase depending on local arrangements]. A total of 9 blood samples, each of 5ml (1 teaspoonful), will be collected (total 45ml, 9 teaspoons for the entire sub-study). The blood samples will be used for the purpose of measuring drug concentrations only.

A tracing of your heart rate will be performed before you take your medicine, and at 2 hours and 5 hours after taking your medicines.

What are the possible risks to me if I join the study?
The risks of taking part in this study are minimal. The blood samples will be drawn through a cannula, although you may also need to have a separate final blood sample drawn if you go home overnight [site to amend as per local arrangements]. There are minor risks of taking blood or having a cannula including bleeding, pain, infection, and bruising.

What are the possible benefits to me if I join the study?
There will be no direct medical benefits to you from taking part in this sub-study. Information that will be obtained from this procedure may be useful scientifically and may benefit future TB patients.

Will I be reimbursed for my participation in this study?
The sub-study will be performed at no charge to you. You will be compensated [Currency X] [amount AAA] for your time, inconvenience and transport costs associated with participation in this sub-study.

What happens to the information collected in the study?
The study clinical staff will collect information at the PK study visit. This information will be stored both on paper and in a computer and will be checked and analysed by study staff in

[insert name of country] in Singapore and the UK. The information will be regularly reviewed by an independent group of international experts to ensure both your safety and that the study is being properly conducted. Information from the PK sub-study will be analysed and be presented and published. We will make such publications publicly available, including to yourself and your community. Your name or identity will remain confidential within the study team. Blood samples collected during the PK sub study may be exported to overseas centres for analysis if permitted by national regulations and subject to ethics committee approvals. The blood samples we collect for storage will be kept for a period of up to 5 years after the study ends for the future analyses. Future analyses on samples will only be related to TB or medicines used to treat TB. We will not inform you of the results of these tests as they will not be of relevance for decisions about your clinical care.

If any new information becomes available that may be relevant to your taking part in the sub--study, you will be informed as soon as possible by the study investigator.

Will my taking part in the PK sub- study be kept confidential?

All information about you will be kept confidential and anonymous and will not be made available to anyone who is not connected with the study without your consent.

If you agree to join the study, persons authorised by University College London (the study Sponsor), as well as ethics and regulatory authorities [site to insert to names of relevant local authorities] will be granted direct access to your original medical records to check study procedures and data without making any of your information public. Study records may also be inspected by independent auditors or government regulators to ensure that the study is being carried out correctly and safely.

Further information

After reading this information sheet, if you feel you do not fully understand everything, please ask for further information from the doctors, nurses or other members of the study team. If you later need more information, please call:

[Insert name / telephone number appropriate for study site]

Name: …………………………………

Telephone Number: ……………………………………………….

CONSENT FORM FOR PHARMACOKINETIC SUB-STUDY OF TRUNCATE-TB TRIAL: SIGNATURE PAGE

Please check each box

- ☐ I confirm that I have read the Consent Form Version [site to insert version number before printing], dated [site to insert version date before printing], for the above study. I have had the opportunity to consider the information, ask questions and have had these answered satisfactorily.

- ☐ I understand that my participation is voluntary and that I am free to withdraw at any time without giving any reason, without my medical care or legal rights being affected.

- ☐ I understand that information collected about me will be anonymised and may be shared with other researchers.

- ☐ I consent voluntarily to participate in this study.

Name	Signature	Date
Name of researcher taking consent	Signature	Date
Name of witness	Signature	Date

APPENDIX 15: SUMMARY OF AMENDMENTS IN VERSION 1.2

Section (V1.2)	Page (V1.2)	Change from V1.1	Rationale
General			
Title page and header	-	Version updated to 1.2; date amended	Update
All sections	-	Throughout protocol: corrected typographical errors; minor rearrangements of text and added or removed phrases that do not alter meaning	Optimise accuracy and clarity
Chapter 1 Trial Summary			
1.1.6	5	Changed from 15 sites in 6-8 countries to 10-15 sites in 4-6 countries	Update
Table 1	9	Footnote 7-Changed “baseline” to “screening” for GeneXpert test	Correct error
Chapter 6 Treatment of patients			
6.2.1	38	Changed doses to those recommended by WHO using FDCs. Increased from 2 to 4 weight bands for Table 2B<br>Added doses of individual drugs and maximum doses, where FDC cannot be used	Optimise accuracy and clarity, improve safety
6.3.1	39	Changed doses of isoniazid, pyrazinamide, ethambutol to those recommended by WHO for FDCs<br>Added doses of individual drugs and maximum doses, where FDC cannot be used	Optimise accuracy and clarity, improve safety
6.3.2	40	Changed doses of isoniazid, pyrazinamide, ethambutol to those recommended by WHO for FDCs<br>Added doses of individual drugs and maximum doses where FDC cannot be used	Optimise accuracy and clarity, improve safety
6.3.3	41	Changed isoniazid dose to weight-based dosing as recommended by WHO	Optimise accuracy and clarity, improve safety
6.3.4	41	Change isoniazid dose to weight-based dosing as recommended by WHO	Optimise accuracy and clarity, improve safety
6.11.1	49	Changed frequency of continuation phase drugs from two times weekly to daily as recommended by WHO<br>Changed doses of rifampicin and isoniazid to those recommended by WHO for FDC	Optimise accuracy
Appendices			
Appendix 7	116	Updated site contact details	Update
Appendix 8	118	Updated number of treatment centres/countries<br>Amended pill burden for each regimen to be consistent with changes in protocol	Update

APPENDIX 16: SUMMARY OF AMENDMENTS IN VERSION 2.0

Section (V2.0)	Page (V2.0)	Change from V1.2	Rationale
**General changes**			
Through-out	–	Corrected minor typos, grammatical errors; changes to formatting, updates to abbreviations and contents list	Cosmetic improvement
**Cover page**			
NA	cover	Changed Trial Statistician<br>Added NCT #	New Trial Statistician
**General Information**			
NA	2	Changed Trial Statistician (UK) and Project Leader.	New personnel in trial coordinating team.
**Chapter 1: Summary of trial**			
1.1.3	3	Changed "treatment of relapses with an 8 month regimen" to "treatment of relapses with a 6 to 8 month re-treatment regimen"	To allow flexibility in regimen duration to follow national treatment guidelines.
1.1.6	5	Changed "10-15 clinical sites" to "approximately 15-20 clinical sites"	New sites being added to meet recruitment target.
1.1.8	5	Added section on "current trial status"	To describe the current status of the MAMS design with the arms open to recruitment at the time of this version of the protocol.
Figure 1	6	1) [Left side, box 3]: Changed "**12w** boosted regimen if symptomatic and smear positive at week 8..." to "**≤ 12w** boosted regimen if symptomatic and smear positive at week 8..."<br>2) [Centre, "Relapse Diagnosed" Box]: Changed "intensity" to "severity" for TB symptoms<br>3) [Centre, "Relapse Diagnosed" Box]: Changed "Increase in cavitation, infiltrates, consolidation..." to "Abnormalities that are compatible with active TB disease (cavitation, infiltrates, consolidation) with clear evidence of progression..."<br>4) [Left side, last box] Added "Empirical" [24w standard regimen]<br>5) [Right side, last box]: Changed "32w WHO empirical re-treatment regimen" to "Empirical 24-32w re-treatment regimen based on national treatment guidelines"<br>6) [Right and left side, last boxes]: Changed "Regimen modified after DST results available" to "Regimen modified according to DST results and drug tolerability"	1) Correction of typo. The clinician may continue boosted treatment for an additional period, up to a maximum of 12 weeks, but this need not be exactly 12 weeks.<br>2) and 3) For consistency with wording in protocol section 6.10.2.<br>5) and 6) For consistency with revised wording in protocol section 6.11.
Figure 2	7	Changed "8 months empirical regimen (in arm A)" to "6-8 months empirical regimen (in Arm A)"	For consistency with revised wording in protocol section 6.11.
Table 1	9	1) Added 'X' for CXR at w96<br>2) Added 'X' for smear at baseline	1) Correction of omission. CXR required for assessment of primary outcome at week 96.<br>2) Correction of inconsistency with text (Section 7.3.10 states smear will be done at baseline)
Table 1 foot note 2	10	Replaced "Additional ECG done at end of treatment (only required for patients taking boosted regimens)" with "Additional ECG required at end of treatment for patients who stop boosted regimen between week 8 and 12; additional ECG required at week 12 for patients who switch boosted regimen to standard treatment between week 8 and 12"	Need a post-treatment ECG in patients who switch boosted treatment (not just those who stop treatment); rephrased to clarify timing.
Table 1 foot note 3	10	Added "and at the first visit of any suspected episode of relapse"	Consistency with other sections of protocol.
Table 1 foot note 7	10	1) Added: "… if a positive result is available from a test performed earlier during this illness episode"<br>2) Changed: "or end of treatment, if treatment is extended" to "and end of treatment, if treatment continues after 8 weeks"	1) Can only omit GeneXpert if previous test is positive.<br>2) Clarification: week 8 GeneXpert required at week 8 (because may be a

			useful biomarker for predicting success) and not optional in the event of treatment extension
Table 1 foot note 12	10	Changed “in stage 4 of trial” to “and trial is open to enrolment of HIV-infected participants at that site”	To be consistent with change in eligibility criteria that may allow entry of HIV patients in Stage 3
Table 1 foot note 13	10	Added: “Samples may be omitted for patient convenience or logistical reasons.”	It may not be possible to collect drug levels for a number of logistical reasons – for example, in cases where a patient needs to take their tablets at night for management of nausea, or who have had drugs suspended for management of toxicity etc.
**Chapter 2: Background**			
2.6.3	27	Moved the following statement from 2.6.4 to 2.6.3: ”In this stage, the eligibility criteria may be relaxed to allow entry of patients with factors considered predictive of potential higher risk of relapse (see Section 4.2.1) to ensure the generalisability of the trial results…”<br>And changed “(the IDMC may recommend against this automatic relaxation of the eligibility criteria depending on their findings at the second interim analyses).” To “(unless instructed otherwise by the local ethics committee, or by any regulatory body that is required to approve amendments to the protocol)”	Earlier expansion of eligibility criteria to increase generalisability
2.6.5	27 and 28	Changed :<br>1) “If a decision is made to permit the enrolment in Stage 4…..” to “After the start of enrolment from Stage 3 …..” in para 1<br>2) “In Stage 4,…” to “From Stage 3, ….” In para 2	Related to earlier expansion of eligibility criteria
**Chapter 4: Selection of patients**			
4	31	Changed:<br>1) “initial 3 stages” to “initial 2 stages”.<br>2) “may be modified formally in stage 4” to “may be modified formally from stage 3”.	Related to earlier expansion of eligibility criteria
4.2	31	Changed “on microscopy” to “on sample taken at screening”	The smear result is needed for both eligibility and stratification. Patients may come from other clinics/ hospitals where the labs have insufficient QA/QC processes in operation for the smear. Therefore this will be standardised to the sputum smear at screening done at the study site
4.2	32	Added: “[removed at time of approval of V2 protocol, depending on site-specific approval by ethics and regulatory bodies]” next to both exclusion criteria 5 and 6.	It is important to test the strategy on a trial population that includes some patients with risk factors associated with a higher rate of relapse, to ensure that the strategy is generalisable to programme settings.<br><br>The amendment brings forward the time when eligibility criteria are expanded, to ensure that sufficient patients with risk factors are enrolled. This has been approved by the IDMC.<br><br>The implementation on a site specific basis means that other aspects of the protocol amendment can be approved even if the oversight body does not agree to the change in eligibility criteria
4.2	32	Added: “[removed at time of approval of V2 protocol]” to exclusion criteria 13 and 14.	Of the trial drugs, only levofloxacin is contraindicated with history of seizures or tendonitis/tendinopathy. Enrolment to Arm D, the only arm containing levofloxacin, has been discontinued.

4.2	32	Added: "[modified to the text in 4.2.1 below, at time of approval of V2 protocol, depending on site-specific approval by ethics and regulatory bodies]" to exclusion criterion 25.	As per rationale for change to exclusion criteria 5 and 6.
4.2	32	Changed from "criteria may be modified in stage 4 of the trial" to "Criteria may be modified from stage 3 of the trial"	Related to earlier expansion of eligibility criteria
4.2.1	32 and 33	Changed:<br>1) "in stage 4" to "from stage 3" in both the heading and the text.<br>2) "...during stage 4 (definitive efficacy and safety stage)" to "…from stage 3 (Qualifying efficacy stage)"<br>3) "…unless instructed otherwise by the IDMC (See Figure 3, page 8 and Section 10.4)" to "(unless instructed otherwise by the local ethics committee, or by any regulatory body that is required to approve amendments to the protocol, see below)"<br><br>Added the following:<br>"The changes in these criteria will be implemented on a site-specific basis. The criteria will not be changed at sites where either the ethics committee or regulatory agency responsible for trial oversight has stated in writing (at the time of reviewing the original protocol, or a protocol amendment) that they do not agree to the criteria being changed. The objection to change may be specific for one, two or all three of the criteria; only the criteria for which no objection has been raised will be changed."<br><br>"To implement the change, the eligibility checklist in use at that site will be modified (one, two or all three criteria changed, as appropriate). From that time, the site will be permitted to randomise patients into the higher-risk stratum (see section 5.2). The PI would retain discretion not to enrol individual patients considered to be at high risk of relapse if they did not consider them suitable for the trial, even if they met all the eligibility criteria".	As per rationale for changes to exclusion criteria 5 and 6.
4.3.2	34	Changed "child bearing age" to "child bearing potential"	Consistency with exclusion criteria.
4.3.2	34	Added: "(GeneXpert need not be repeated if a positive result is available from a test performed earlier during this illness episode, done in study-approved laboratory and results are available to research team)"	Consistency with footnote in trial schedule (Table 1)
4.3.2	34	Changed "CD4 count (Only in stage 4 of the trial" to "CD4 count (only if patient HIV positive and trial is open to enrolment of HIV-positive participants at that site)"	Consistency with footnote in trial schedule (Table 1).
**Chapter 5: Randomisation and Enrolment**			
5.1	35	1) Added "urine (10ml) for storage"<br>2) Added "Sputum collection for smear, liquid culture (MGIT) and DST"<br>3) Changed "safety tests" to "standard safety monitoring tests"<br>4) Changed "sparse PK" to "drug levels"	1) Correction of error (test listed in trial schedule and elsewhere, but previously omitted here)<br>2) Correction of error (tests listed in trial schedule and elsewhere, but previously omitted here; baseline smear listed only in section 7.3.10)<br>3) and 4) Consistency with wording elsewhere
5.2	36	1) Added "screening" before every mention of "smear" and "CXR cavities".<br>2) Changed "…from stage 4" to "…from stage 3 (subject to the conditions described in section 4.2.1)"	1) Clarification<br>2) Consistency with wording elsewhere

5.2	36	Added: "Where patients are unable to produce sputum at screening, the smear result will be regarded as negative for the purposes of relapse risk stratification."	Clarification on approach to risk stratification - inability to produce sputum (despite appropriate effort and technique) correlates with less severe disease
**Chapter 6: Treatment of Patients**			
6.1	37	Changed the duration for the retreatment regimen after relapse on standard treatment from "8 months" to "6 to 8 months"	Change in national treatment guidelines may allow shorter treatment for relapse (WHO category II 8-month regimen no longer recommended)
6.2.1	38 - 41	Changed threshold for highest weight band from ">71kg" to "≥71kg".	Correction, to align with threshold in WHO guidelines
6.3.1 and 6.3.2	39 and 40	Added rifampicin "450mg or 600mg"	450mg or 600mg rifampicin preferred to reduce pill burden where possible.
6.6	43	Changed "in stage 4, following IDMC recommendation" to "from stage 3, subject to conditions described in section 4.2.1"	Related to earlier expansion of eligibility criteria
6.8.2	45	Added: "or at week 10 if treatment is extended until that time"	Treatment can be extended to week 10 and clinical status re-evaluated then; this is better for patients than mandating extension to 12 weeks in all who have persistent clinical disease at week 8
6.10.2	48	Added "Histological evidence considered to show characteristic changes of TB would also be acceptable under criterion C"	Histological evidence often important for making diagnoses at extra-pulmonary sites
6.10.3	49	Added: "or other sample, if extra-pulmonary disease suspected" and "or other" [culture]"	Relapse may be at a non-pulmonary site and non-sputum samples may be obtained.
6.11.1	49	Added:<br>1) "Treatment will be with an empirical 24 to 32-week re-treatment regimen based on national treatment guidelines, modified according to drug susceptibility testing (see 6.11.3 below) and drug tolerability."<br>2) Some national treatment guidelines recommend …"	Prioritises national treatment guidelines above previous WHO recommended category II 32-week (8 month) regimen. Additional text emphasises the need to adjust for results of susceptibility testing and drug tolerability.
6.11.1	49	Changed: "Empirical re-treatment regimen for the standard management strategy" to "Drug doses in 32w (8-month) empirical re-treatment regimen"	Re-phrased for accuracy.
6.11.2	50	Added: "Empirical" and "modified according to drug susceptibility testing (see 6.11.3 below) and drug tolerability" .	As per justification for 6.11.1 above.
6.11.2	50	Changed: "the empirical retreatment regimen will be with the standard WHO re-treatment regimen (as above in Section 6.11.1)." to "the empirical regimen will be a 24 to 32-week re-treatment regimen based on national treatment guidelines (as above in Section 6.11.1), modified according to drug susceptibility testing (see 6.11.3 below) and drug tolerability.	As per justification for 6.11.1 above.
**Chapter 7: Assessments & Follow-up**			
7.1.2	51	Changed "week 24" to "week 48"	Correction of error.
7.2.2	53	Deleted "(should be deferred to end-of-treatment visit (Section 7.1.3) if treatment extended)"	Clarification: week 8 GeneXpert required at week 8 (because may be a useful biomarker for predicting success) and not optional in the event of treatment extension
7.2.2	54	Added "and who did not have an end of treatment ECG after week 8"	Week 12 ECG not necessary if already done at end of treatment between week 8 and 12.
7.2.2	54	Added CXR to the list of week 96 assessments.	Correction of omission. CXR required for evaluation of the primary outcome at week 96.
7.2.3	54	Added: "and 7.2.2"	To correct omission

7.2.3	54	Added CXR to the list of week assessments required 48 weeks after start of re-treatment (only if this falls after the week 96 scheduled visit)	To permit evaluation of the primary outcome in patients who require final visit after week 96.
7.2.4	55	Added: "…and 7.2.2 for the appropriate scheduled visits"	To correct omission.
7.2.4	55	Changed "…only if patient on boosted regimen"<br>to: "…only for patients who stop boosted treatment between week 8 and 12"	Clarification of which patients require end of treatment ECG.
7.2.8	55	Added: "…or reversion of smear from negative to positive after end of treatment"	Monitoring strategy includes monitoring symptoms and smear. Therefore a change in smear should prompt a relapse assessment.
7.2.8	55 and 56	1) Added: "(household contact history only required if decision is made to re-start treatment)"<br>2) Deleted: "(encouraged, but optional at the discretion of the managing clinician)"<br>3) Added: "Collect additional sample(s) if required to ensure there are at least two with positive cultures or that have results awaited in the period prior to restarting therapy".<br>4) Added: "Urine for pregnancy test (if applicable; if the decision is made to re-start TB treatment)"	1) Some patients may be assessed as not having a relapse so household contact history not needed.<br>2) CXR is essential for evaluation of suspected relapse in order to assess the relapse diagnostic criteria.<br>3) Additional sputum samples are needed to increase changes proving relapse.<br>4) To be consistent with other parts of protocol (footnote of table 1).
7.3.4	58	Changed "higher" to "lower"	Correction of error.
7.3.6	58	Changed: "…and end of treatment (end of treatment only required in those who have taken a boosted regimen)" to:<br>"...end of treatment (only required if patient stops a boosted regimen between weeks 8 and 12) and week 12 (only required if patient switches a boosted regimen to standard treatment between weeks 8 and 12).	Clarification of ECG requirements.
7.3.7	59	Changed "…at the time of suspected relapse (optional at the discretion of the clinician)" to:<br>"…at the time of suspected relapse, week 96 and the final trial visit (if this is not at week 96; see section 7.1.4)"	1) Change from optional to required at time of suspected relapse as per rationale in 7.28 above.<br>2) Addition of week 96 CXR as per rationale in 7.2.2.<br>5) Clarification of requirements for final trial visit.
7.3.8	59	Added :<br>1) "if this is not at week 96"<br>2) "Spirometry may be omitted for clinical or logistical reasons".	1) Clarification of requirement for final trial visit<br>Some patients may be too unwell to perform spirometry; or it may also not be possible for logistical reasons.
7.3.9	59	Urine; added:<br>1) "….and prior to start of re-treatment for relapse"<br>2) "week 24, end of treatment" for urine for storage	Correction of omissions.
7.3.10	60	Changed: "or end of treatment, if treatment is extended" to "and end of treatment, if treatment continues after 8 weeks"	3) Clarification: week 8 GeneXpert required at week 8 (because may be a useful biomarker for predicting success) and not optional in the event of treatment extension
7.3.10	60 and 61	Re DST:<br>1) Changed: "all isolates obtained from cultures after 8 weeks on treatment" to: "all isolates obtained from cultures at or after 8 weeks on treatment"<br>2) Added: "additional isolates as required to verify findings obtained from the isolates listed above or to elucidate evolution of drug susceptibility in longitudinal samples"<br>3) Changed: "Baseline isolates will be tested for susceptibility to standard first-line drugs and fluoroquinolones" to "At a minimum, baseline isolates will be tested for susceptibility to standard first-line drugs"<br>4) Changed "At week 8 and subsequently, isolates will be tested for standard first-line drugs…" to: "At a minimum, isolates obtained at week 8, isolates obtained at relapse, and isolates obtained at	1) Clarification that samples obtained at 8 weeks will be tested.<br>2) It may be necessary to test intermediate samples to fully explore drug susceptibility profiles and changes.<br>3) Not essential to do baseline fluoroquinolone DST for patients who are not exposed to fluoroquinolones. May want to do additional DST on baseline samples from any patients who subsequently relapse to look for baseline resistance to the drugs prescribed.

		baseline in patients who subsequently relapse will be tested for susceptibility to standard first-line drugs..."<br>5) Added "Additional DST may be performed at the discretion of the trial management group to seek (or exclude) evidence of phenotypic resistance patterns that might contribute to understanding trial outcomes".	4) and 5): Expanding the scope of drug resistance measurement will add value for understanding trial results.
7.3.10	61	3) Added "Additional WGS may be performed systematically on isolates obtained at baseline or during treatment to examine the relationship between bacterial genome, drug susceptibility and treatment outcomes".	Added scientific value from performing additional WGS on bacterial isolates.
7.3.11	61	Changed "…has reached stage 4 (where HIV is not an absolute exclusion)" to "…has reached a stage where the site is open to enrolment of HIV patients"	Change related to earlier expansion of eligibility criteria
7.3.11	61 and 62	Added:<br>1) "window 1-2 hours post-dose"<br>2) "Samples may be omitted for patient convenience or logistical reasons."	1) Consistency with section 5.1 of protocol.<br>2) It may not be possible to collect drug levels for a number of logistical reasons – for example, in cases where a patient needs to take their tablets at night for management of nausea, or who have had drugs suspended for management of toxicity etc.
**Chapter 8: Safety Reporting**			
8.1.5	66	Added standard definition of Suspected Unexpected Serious Adverse Reaction (SUSAR)	Correction of omission
8.2.1	66	Delete the words: "describing safety assessments"	Redundant words removed for clarity.
8.2.4	67	Changed "within 24 hours" to "as soon as possible (latest within 24 hours).	Prompt reporting necessary in order to ensure that sponsor can meet obligations for onward reporting
**Chapter 10: Statistical considerations**			
10.1	70	1) Added "screening" before every mention of "smear" and "CXR cavities".<br>2) Changed "…in stage 4" to "….from stage 3 (subject to the conditions described in section 4.2.1)"	1) Consistency with wording in 5.2<br>2) As per justification for changes to sections 2.6.3, 2.6.4 and 4.2.1, above.
10.4.1	78	Changed "in stage 4" to "from stage 3 of the trial"	As per rationale for changes to sections 2.6.3 and 2.6.4.
10.4.5	80	Changed:<br>1) "second" to "first" [interim analysis]<br>2) "Stage 3" to "Stage 2";<br>3) "high-risk" to "higher-risk"<br>4) "subject to the criteria specified in the trial eligibility criteria" to "subject to the criteria specified in section 4.2.1"	1) and 2) As per rationale for changes to sections 2.6.3 and 2.6.4. "<br>3) The factors increase the relative risk of relapse but the absolute risk may be low; "higher risk" is the more appropriate term<br>4) As per rationale for changes to sections 2.6.3 and 2.6.4.
10.5.1	80	Deleted "that have a positive culture for *Mycobacterium tuberculosis* at screening and/or randomisation" from definition.	Correction of error. Eligibility criteria require participants to be GeneXpert positive which is taken as sufficient evidence for TB to be included in the main analysis population (culture positive not required).
**Chapter 13: Regulatory approvals and compliance**			
13.3.2	99	Added the following section:<br>"Source Data Agreement<br>The location of the information regarded as source data at each site will be described in a source-data agreement between the site and the sponsor. The CRF may be used as the source document for some or all of the data to be collected, provided that the specific arrangements are noted in the source data agreement and that this does not contravene applicable local regulations."	As per ICH GCP guidelines, the trial has a site-specific agreements in place to define the location of source data at a particular site.

13.3.3	99	Changed: “and held for 15 years” to “and will be held for up to 15 years”<br>Added “depending on sponsor and national regulatory requirements”	Added for clarity. In some of the trial countries, storage of trial documents is not required for as long as 15 years.
**Appendices**			
Appendix 7	116 to 118	Updated list of sites and principle investigators	Updated list
Appendix 8	124	Added: “and at week 96”	Additional CXR will be performed at the week 96 trial visit, as per rationale for change to 7.2.2.

STATISTICAL ANALYSIS PLAN

Two-month Regimens Using Novel Combinations to Augment Treatment Effectiveness for drug-sensitive Tuberculosis (TRUNCATE-TB)

Protocol Version/Date: Version 2.0 dated 06 November 2019

Indication: Pulmonary Tuberculosis

Phase: seamless II/III

Document Version: 1.0

Date: 25 November 2021

CONFIDENTIAL

Chief Investigator:
Prof Nicholas Paton, MD FRCP
Department of Medicine,
Yong Loo Lin School of Medicine, National University of Singapore
NUHS Tower Block Level 10,
1E Kent Ridge Road, Singapore 119228
Tel: +65 6772 6988
Email: nick_paton@nus.edu.sg

Trial Statisticians:
Angela Crook, PhD
Institute of Clinical Trials and Methodology
MRC Clinical Trials Unit at UCL
90 High Holborn 2nd Floor
London WC1V 6LJ
Direct line: +44 20 7670 4751
E-mail: angela.crook@ucl.ac.uk

Mihir Gandhi
Singapore Clinical Research Institute
31 Biopolis Way, Nanos #02-01
Singapore 138669
Tel: +65 6508 8300
E-Mail: mihir.gandhi@scri.cris.sg

APPROVAL

The undersigned hereby declare that they have prepared/examined the Statistical Analysis Plan and agree to its form and content. In addition, they confirm that to the best of their knowledge the Statistical Analysis Plan contains all information relevant for the conduct of statistical analysis of the study.

Initial draft prepared by: Trial statistician (blinded) [Mihir Gandhi]

25 November 2021

Sign *Date*

Final draft prepared by: Trial statistician (blinded) [Angela Crook]

25 November 2021

Sign *Date*

Final draft reviewed and approved by: Chief Investigator [Nicholas Paton**]**

25 November 2021

Sign *Date*

TABLE OF CONTENTS

APPROVAL **2**
TABLE OF CONTENTS **3**
GLOSSARY AND ABBREVIATIONS **9**
1 INTRODUCTION **11**
1.1 Global Disease Burden 11
1.2 Rationale of This Study 11
2 STUDY OBJECTIVES **12**
3 STUDY DESCRIPTION **12**
3.1 Study Design 12
3.2 Eligibility 13
3.3 Randomisation 13
3.4 Blinding 14
3.5 Interim Analyses 14
3.6 Interim Analysis after each of the first 3 trial stages 14
3.7 Study Assessments 15
4 DERIVATION OF ENDPOINTS AND OTHER DATA TO BE ANALYSED16
4.1 Summary of endpoints used for each analysis 16
4.1.1 Efficacy and safety of the management strategy 16
4.1.2 Implementation of the management strategy 16
4.1.3 Health economics of the management strategy 17
4.1.4 Efficacy and safety of the regimens 17
4.1.5 Bactericial activity of the boosted regimens 18
4.2 Baseline visit and windows for assessments 18
4.3 Other follow-up visits and windows for assessments 19
4.4 Week 96 visit and windows for assessments 20
4.5 Terminology used for randomised arms 22
4.6 Treatment courses, adherence, and observation 23
4.6.1 Treatment days 24
4.6.2 Trial-wide regimen changes, impact on treatment days 25
4.6.3 Number of treatment courses 25
4.6.4 Start and end dates for treatment courses 26
4.6.5 Treatment duration 29
4.6.6 Treatment daily doses taken 29

4.6.7 Categorical classifications for treatment completion and default 30
4.6.8 Categorical classifications for treatment extension 31
4.6.9 Re-treatment after week 96 32
4.6.10 Adherence 32
4.6.11 Treatment observation 33
4.7 Clinical observations and investigations (non-laboratory) 34
4.7.1 TB-related symptom checklist 34
4.7.2 Severity of TB episode 36
4.7.3 Chest X-ray 36
4.7.4 ECG 37
4.7.5 Spirometry 38
4.7.6 Pre-existing diabetes 40
4.8 Classification of adverse events 40
4.8.1 Classification rules used by sites 40
4.8.2 Central review of adverse events 40
4.8.3 Coding of adverse events 41
4.8.4 Definition of incident adverse events 41
4.8.5 Definition of relatedness 42
4.8.6 Counting of adverse events used in the safety analyses 42
4.8.7 Adverse events of special interest 43
4.9 Microbiology variables 44
4.9.1 Sputum sample collection status 44
4.9.2 Allocating sputum samples to visits 45
4.9.3 Interpreting multiple samples at a single visit / critical timepoint 45
4.9.4 Sputum smear status and grade 46
4.9.5 Sputum smear conversion and reversion 47
4.9.6 Sputum GeneXpert status and cycle threshold 47
4.9.7 Sputum GeneXpert conversion and reversion 50
4.9.8 Sputum Culture Status and Time to Positivity 50
4.9.9 Sputum culture conversion 52
4.9.10 Treatment failure analysis criteria 54
4.9.11 Relapse 57
4.9.12 Interpretation of positive culture results by whole genome sequencing 59
4.9.13 Drug resistance by MGIT 60
4.9.14 Drug resistance by MIC plate testing 61
4.9.15 Drug resistance by whole genome sequencing 61
4.10 Baseline Risk Stratum 62
4.11 Disease activity criteria 63

4.11.1 Disease activity clinical management criteria ... 63
4.11.2 Disease activity research criteria ... 66
4.12 Definition of unsatisfactory clinical outcome at week 96 (primary outcome for the strategy analysis ... 67
4.12.1 Classification of clinical outcome for patients who attend during the week 96 analysis window or are known to have died ... 68
4.12.2 Classification of clinical outcome for patients not attending during the week 96 analysis window ... 70
4.12.3 Blinded review of outcomes ... 71
4.13 Definition of unfavourable outcome (primary outcome for the regimen analysis) ... 71
4.14 Patient-centred variables ... 78
4.14.1 Acceptability of the strategy ... 78
4.14.2 Time off work or study due to illness or treatment ... 79
4.14.3 Quality of life (MOS-HIV) ... 79
4.14.4 Health status (EQ-5D) ... 79
4.14.5 Respiratory disability (MRC dyspnoea scale) ... 80
4.15 Definitions related to transmission risk ... 80
4.16 Definitions related to health economics evaluation ... 81
4.17 Derivation of socioeconomic status ... 82

5 SAMPLE SIZE CALCULATION ... 83

6 ANALYSIS POPULATIONS ... 84
6.1 Intention-To-Treat (ITT) Population ... 84
6.2 Intention-To-Treat Exposed (ITT Exposed) Population ... 84
6.3 Modified Intention-To-Treat (mITT) Population ... 84
6.4 Modified Intention-To-Treat Exposed (mITT Exposed) Population ... 84
6.5 Modified Intention-To-Treat, other populations ... 85
6.6 Per-Protocol Population ... 86

7 STATISTICAL ANALYSIS GENERAL PRINCIPLES ... 88
7.1 Sequence of analyses ... 88
7.2 General approach to analysis ... 89

8 ANALYSIS OF THE EFFICACY AND SAFETY OF THE MANAGEMENT STRATEGY ... 90
8.1 General ... 90
8.2 Analysis populations and treatment arms ... 91
8.2.1 Analysis populations ... 91

8.2.2 Analysis treatment arms ... 91
8.3 Enrollment and follow-up ... 92
8.4 Demographics and Baseline Characteristics ... 93
8.5 Exposure and Adherence to Study Medications ... 95
8.5.1 Trial-wide regimen changes ... 95
8.5.2 Number of treatment courses ... 95
8.5.3 Treatment course duration and daily doses taken ... 96
8.5.4 Treatment completion and default ... 96
8.5.5 Adherence ... 97
8.5.6 Treatment observation ... 98
8.6 Analysis of the primary outcome ... 98
8.6.1 Main analysis of the primary outcome ... 98
8.6.2 Sensitivity analyses of the primary outcome ... 99
8.6.3 Exploratory analyses of primary outcome on all arms ... 100
8.6.4 Subgroup analyses of the primary outcome ... 100
8.7 Analysis of Secondary Outcomes from the patient perspective - patient-centred outcomes and measured respiratory disability ... 101
8.7.1 Acceptability of the strategy ... 102
8.7.2 Total days on TB drug treatment ... 103
8.7.3 Time off work or study due to illness/treatment ... 103
8.7.4 Quality of life ... 103
8.7.5 Health Status ... 104
8.7.6 Respiratory disability ... 104
8.8 Analysis of secondary outcomes from the patient perspective – clinical adverse events ... 105
8.9 Analysis of secondary outcomes from the programme perspective ... 107
8.9.1 Adherence to TB medication ... 107
8.9.2 Treatment default ... 107
8.9.3 Acquired drug resistance ... 107
8.9.4 Community transmission risk ... 108
8.10 Analysis of other outcomes from the patient and programme perspective ... 108
8.10.1 Body weight ... 108
8.10.2 BMI ... 109
9 ANALYSIS OF THE IMPLEMENTATION OF THE MANAGEMENT STRATEGY ... 110
9.1 General ... 110
9.2 Analysis population and treatment arms ... 111

9.3 Enrollment and follow-up ... 112
9.4 Demographics and Baseline Characteristics ... 112
9.5 Overall evaluation of strategy implementation ... 112
9.5.1 Overall treatment burden over 96 weeks ... 113
9.5.2 Overall symptom burden over 96 weeks ... 113
9.5.3 Overall microbiological disease activity over 96 weeks ... 113
9.6 Analyses of feasibility and utility of each of the strategy components ... 114
9.6.1 Identification of patients suitable for the strategy ... 114
9.6.2 Initial treatment ... 114
9.6.3 Initial treatment course, stop decision ... 118
9.6.4 Monitoring ... 119
9.6.5 Retreatment, decision to start ... 123
9.6.6 Re-treatment ... 124
9.6.7 Retreatment, decision to stop ... 124
9.7 Relationship between site characteristics, patient factors and implementation parameters ... 125
9.8 Relationship between initial treatment, relapses and strategy outcomes ... 126

10 ANALYSIS OF THE HEALTH ECONOMICS OF THE MANAGEMENT STRATEGY ... 128
10.1 Analysis population and treatment arms ... 128
10.2 Analysis general ... 128

11 ANALYSIS OF EFFICACY AND SAFETY OF BOOSTED REGIMENS ... 130
11.1 General ... 130
11.2 Study population and treatment arms ... 132
11.3 Enrollment and follow-up ... 133
11.4 Demographics and Baseline Characteristics ... 133
11.5 Exposure and Adherence to Study Medications ... 133
11.6 Efficacy of the trial regimens ... 134
11.6.1 Main analysis of the primary outcome ... 134
11.6.2 Sensitivity analyses of the primary outcome ... 135
11.6.3 Possible relapses in post week-96 follow-up period ... 136
11.6.4 Subgroup analyses of the primary outcome ... 137
11.6.5 Analysis of time-to-unfavourable outcome ... 138
11.6.6 Analysis of time to treatment failure or relapse ... 138
11.6.7 Analysis of Chest X-ray change ... 138

11.7 Safety of the trial regimens 139
11.7.1 General overview 139
11.7.2 QTc Prolongation 141
11.7.3 Liver toxicity 142

12 ANALYSIS OF BACTERICIDAL ACTIVITY OF THE REGIMENS 143

12.1 Endpoints for analysis of the bactericidal activity of the regimens 143
12.2 Study population and treatment arms 143
12.3 Enrollment and follow-up 143
12.4 Demographics and Baseline Characteristics 143
12.5 Exposure and Adherence to Study Medications 143
12.6 Time to culture conversion by week 8 144
12.7 Sputum culture conversion status at week 8 145
12.8 Change in Time to Positivity (TTP) 145

GLOSSARY and ABBREVIATIONS

AE	Adverse event
ALT	Alanine transferase
ART	Antiretroviral therapy
AUC_{24}	area under the curve of plasma drug concentration until 24 hours
CD4	Cluster of differentiation-4 T-cell lymphocyte
CI	Confidence interval
C_{max}	Peak plasma concentration
CONSORT	Consolidated Standards of Reporting Trials
CRF	Case report form
CXR	Chest X-ray
DAIDS	Division of AIDS
DOT	Directly observed therapy
DST	Drug susceptibility testing
DS-TB	Drug sensitive tuberculosis
ECG	Electrocardiogram
EQ5D	EuroQOL five dimensions questionnaire
FEV1	Forced expiratory volume in 1 second
GLI	Global Lung Function Initiative
HIV	Human immunodeficiency virus
IDMC	Independent Data Monitoring Committee
MAMS	Multi-arm, multi-stage
MDR-TB	Multi-drug resistant tuberculosis
MedDRA	Medical Dictionary for Regulatory Activities
MGIT	Mycobacterial growth indicator tube system
MHS	mental health summary score
mITT	Modified intention-to-treat
MOS-HIV	Medical outcomes study HIV questionnaire
MRC	Medical Research Council
ms	Milliseconds
PHS	physical health summary score
PK	Pharmacokinetic
PP	Per-protocol
PT	Preferred term
QT	QT interval
QTc	QT interval (corrected)
SAE	Serious adverse event
SAP	Statistical analysis plan
SAS	Statistical Analysis System
SCRI	Singapore Clinical Research Institute
SOC	System organ class
SMS	Standard Management Strategy

STR	Standard therapy re-introduction
$t_{1/2}$	Half-life
TB	Tuberculosis
T_{max}	Time to peak plama concentration
TMS	TRUNCATE-TB Management Strategy
TSC	Trial Steering Committee
WCC	White Cell Count
WGS	Whole genome sequencing

1 INTRODUCTION

1.1 Global Disease Burden

TB remains a major global disease, with an estimated 9 million new TB cases and 1.5 million TB deaths per year. Although multi-drug resistant TB (MDR-TB), defined as TB with resistance to rifampicin and isoniazid, is an increasing problem and is complicated and expensive to treat, the large majority of new cases of TB are drug-sensitive (DS-TB). Improving the treatment of DS-TB is likely to bring important global health benefits both by directly improving outcomes of patients and by preventing the generation of new cases of MDR-TB.

The standard TB management strategy for drug-sensitive pulmonary disease comprises 6 months combination therapy, preferably with the intake of each dose of medication observed by a healthcare worker (called directly observed therapy, DOT). The regimen used in the standard TB management strategy consists of 2 months of rifampicin, isoniazid, pyrazinamide and ethambutol (intensive phase) followed by 4 months of rifampicin and isoniazid alone (continuation phase).

1.2 Rationale of This Study

Although the standard TB management strategy is theoretically sound, with cure rates in trial setting that were consistenly above 95%, in reality resource limitations combined with a challenging patient population create conditions that promote low cure rates, generation of MDR-TB and spread (of DS- and MDR-TB) within the community. With healthcare systems overstretched and experiencing large funding gaps, it is clear that simply continuing to emphasise the same treatment and delivery approach is unlikely to provide a solution to the problem in any reasonable time frame. Although the development of new drugs and novel regimens targeted at MDR-TB is important, this is a downstream solution that will benefit only a minority of patients. Furthermore, the benefits of new drugs for MDR-TB is likely to be short-lived as resistance to new drugs develops rapidly when they are combined with relatively weak companion drugs.proach to the treatment of DS-TB in programmes. New approaches are needed that allow healthcare systems to use limited resources more effectively and economically to treat and cure more patients with DS-TB and that minimise the conditions that promote the generation and spread of MDR-TB.

It has been shown in various clinical trials that majority of patients can be cured with treatment courses of 4 months or shorter, where relapse rates were between 7% to 15%. It is clear that with current standard TB management strategy, the large majority of patients receive many months of additional treatment that is unnecessary. For patients, this unnecessary treatment causes inconvenience, increases the risk of drug toxicity and also sets a target for treatment that may feel unattainable and thereby contribute to poor motivation and default. For programmes, resources are squandered on distributing and supervising this unnecessary treatment that in turn dilutes the capacity to provide more intensive support at periods and to persons where it matters most.

An alternative approach of treating everyone with a long course 6-months of treatment (thereby giving unnecessary treatment to the majority for the sake of the minority who need it, but cannot be identified) is the TRUNCATE-TB management strategy: to treat everyone with a much shorter course of treatment (which is adequate for the majority) and to follow closely and retreat the minority of those who relapse (and are thereby identified as needing longer treatment) with a standard treatment regimen. This trial aims to evaluate the effectiveness of the TRUNCATE-TB Management Strategy using different 8-week boosted regimens as separate treatment arms, compared to the Standard Management Strategy.

2 STUDY OBJECTIVES

Primary Objective

To test the hypothesis that the TRUNCATE-TB Management Strategy is non-inferior to the Standard Management Strategy assessed by the proportion of patients with unsatisfactory clinical outcome at 2 years (96 weeks) after randomisation.

Secondary Objectives

- To assess the possible advantages of the TRUNCATE-TB Management Strategy compared to the SMS from the patient perspective (including acceptability, quality of life and clinical adverse events) and the programme perspective (including treatment adherence, default, new drug resistance and community transmission) as well as costs (to patients and programmes) and cost-effectiveness.
- To evaluate the pharmacokinetics, microbiological efficacy and toxicity of a number of boosted 8-week regimens in comparison to the standard treatment regimen.

Exploratory Objective

To explore the relationship between various biomarkers (clinical and laboratory parameters as well as drug exposure and possible novel laboratory biomarkers) on outcomes related to sterilisation and cure.

3 STUDY DESCRIPTION

3.1 Study Design

The trial is a randomised, open-label, multi-arm, multi-stage (MAMS), parallel group non-inferiority trial comparing the TRUNCATE-TB Management Strategy with the standard TB management strategy on the primary outcome of the proportion of patients who have unsatisfactory clinical outcome at week 96. Patients will be randomised in equal proportions to 5 treatment arms: 4 arms of boosted regimens of the TRUNCATE-TB management strategy, and 1 control arm of the standard TB management strategy (See Section 6.3 of trial protocol for details of boosted regimens).

The regimen used in the standard TB management strategy consists of 2 months of rifampicin, isoniazid, pyrazinamide and ethambutol (the intensive phase) followed by 4 months of rifampicin and isoniazid alone (the continuation phase). The planned treatment duration of 8 weeks with boosted regimens may be extended to a maximum of 12 weeks in patients who have evidence of ongoing TB disease activity at 8 weeks or who miss doses. Patients in all treatment arms are to receive their prescribed TB medications once daily during the treatment period. The standard TB treatment may be introduced/re-introduced to patients if clinically required. Patient participation will last for 96 weeks from randomisation (extended in individual cases to allow 24-weeks follow-up after the completion of any period of re-treatment). All patients will be contacted by telephone for a close of trial visit.

The trial will test the TRUNCATE-TB Management Strategy using a multi-arm multi-stage (MAMS) design that has been applied successfully in a trial of multiple regimens for prostate cancer,[16] and a TB trial. The trial starts with multiple arms and, as it progresses, recruitment to those arms that do not show sufficient promise on an early intermediate outcome measure is discontinued whilst recruitment to the control arm and remaining boosted arms continues until sufficient numbers of patients have been enrolled to assess the outcome on the primary outcome measure. Recommendations about stopping or continuing arms are made by an Independent Data Monitoring Committee (IDMC).

3.2 Eligibility

Patients aged 18 to 65 years with pulmonary TB who took no more than 10 daily doses of standard anti-TB medication or fluroquinolones during the 3 months prior to randomisation may be enrolled in to the trial. The full list of patient inclusion and exclusion criteria is available in Section 4 of the trial protocol (pages 31 and 32). The protocol includes provision to remove the criteria restricting enrolment of patients with factors predictive of potential higher risk of relapse during stage 4 (definitive efficacy and safety stage) of the trial unless instructed otherwise by the IDMC (as described in section 4 of the trial protocol).

3.3 Randomisation

Randomisation of participants will be performed by delegated site personnel with password secured accounts through a web-based randomisation system provided by Singapore Clinical Research Institute (SCRI). Patients will be randomised in equal proportions to the following 5 arms.

- Arm A: 24 weeks rifampicin (standard dose), isoniazid, pyrazinamide, ethambutol (pyrazinamide and ethambutol for 8 weeks)
- Arm B: 8 weeks high dose rifampicin, isoniazid, pyrazinamide, ethambutol, linezolid
- Arm C: 8 weeks high dose rifampicin, isoniazid, pyrazinamide, ethambutol, clofazimine
- Arm D: 8 weeks rifapentine, isoniazid, pyrazinamide, linezolid, levofloxacin
- Arm E: 8 weeks isoniazid, pyrazinamide, ethambutol, linezolid, bedaquiline

Randomisation will be stratified by site and baseline risk stratum (standard definition, as in 4.10; lower risk: sputum smear negative and CXR cavities ≤ 4 cm and HIV negative; intermediate risk: sputum smear positive ≤2+ and CXR cavities ≤ 4 cm and HIV negative; higher risk: sputum smear grade 3+ or CXR cavities > 4 cm or HIV positive). Enrolment of patients with potential higher risk will only be done in stage 4.

Enrolment to treatment arms of boosted regimens may be discontinued during the trial if recommended by IDMC based on review of safety and/or efficacy data. In that case, patients will be randomised in equal proportions to each of the regimens (including the control arms) open for enrolment.

3.4 Blinding

This is an open-label trial: treatment allocations will not be concealed to study team members at sites and patients themselves once their randomisations are performed.

3.5 Interim Analyses

Interim analyses will be performed for IDMC meetings after each of the first 3 trial stages as below. Results may be generated without a formal report for IDMC review at other time points as specified in Section 10.4.1 of the protocol. The IDMC reviews will include data on recruitment, adherence to randomised strategies, culture conversion, rate of relapse and treatment failure, toxicity, switches to standard treatment regimens and other key safety and efficacy outcome parameters.

The IDMC will be able to recommend discontinuation of recruitment to an arm based on a combination of efficacy and toxicity factors, or taking into account other considerations. In considering the treatment failure/relapse rate the IDMC will also review data to evaluate the impact of those outcomes in terms of clinical morbidity, drug resistance and community transmission risk.

3.6 Interim Analysis after each of the first 3 trial stages

1. After Stage 1 (pilot safety stage). As soon as possible after the first 10 patients enrolled in the control arm of the trial have reached 4 weeks of follow-up. This analysis will be for the purpose of reviewing initial safety data to ascertain whether there are any safety concerns that might require additional safety monitoring or the cessation of one or more of the treatment arms on safety grounds.

2. After Stage 2 (early efficacy stage). As soon as possible after the first 30 patients enrolled in the control arm of the trial have reached 6 months post-randomisation, the first interim efficacy analysis will be done for potential recommendations on discontinuing recruitment to arms. The IDMC will be asked to consider recommending stopping recruitment to any boosted regimen arm that meets at least one of the following guidelines:

- The observed combined treatment failure/relapse rate exceeds 25%.
- The hazard ratio of the time to stable culture conversion (as compared to the control arm) does not exceed a threshold of 0.8.

3. After Stage 3 (qualifying efficacy stage). As soon as possible after the first 70 patients enrolled in the control arm of the trial have reached 6 months post-randomisation, the second interim efficacy analysis will be performed. If the observed combined treatment/relapse rate exceeds 20% in a boosted arm or if the hazard ratio of the time to stable culture conversion as compared to the control does not exceed a threshold of 0.9 then the IDMC will be asked to consider recommending stopping recruitment in that arm.

3.7 Study Assessments

Patients will be required to attend protocol-mandated follow-up visit at weeks 1, 2, 4, 6, 8, 10, 12, 16, 20, 24, 36, 48, 60, 72, 84 and 96. In addition to the scheduled clinic visits, brief telephone assessments will be conducted at the following time points (timed approximately every 4 weeks, between study visits): weeks 30, 40, 44, 52, 56, 64, 68, 76, 80, 88, 92 and at the close of the trial. The telephone assessment will be omitted if it coincides with a visit in person.

Windows around visits up to and including week 4 will be ±3 days; for week 6 to week 8 ±5 days; week 10 to week 24 ±7 days; for week 30 to week 84 ± 14 days; for week 96 ± 28 days.

If a patient is being re-treated, his/her re-treatment scheduled visits will be required at 2, 4, 8, 12, and 24 and 48 weeks after the start of the re-treatment episode (with the same windows as for the corresponding weeks for standard protocol visits listed above). The standard scheduled visits and re-treatment scheduled visits will be combined and the timing adjusted in order to minimise the additional visit burden for patients where possible.

Other potential visits, including end of treatment visit, final trial visit, close of trial telephone visit and unscheduled visits, are described in Sections 7.1.3 to 7.1.6 of the trial protocol.

The schedule of assessments is summarized in Table 1 of the trial protocol (page 9). Additional assessments (if not already mandated at the visit) will be done at the first visit where relapse is suspected, which include but not limited to medical history, CXR, sputum for smear and liquid culture (and DST and WGS if culture positive), GeneXpert test.

4 DERIVATION OF ENDPOINTS AND OTHER DATA TO BE ANALYSED

4.1 Summary of endpoints used for each analysis

4.1.1 Efficacy and safety of the management strategy

Primary:
- Unsatisfactory clinical outcome at week 96

Secondary:
(main secondary endpoints marked with *)

Patient perspective: Efficacy
- Acceptability of the strategy
- Total days on TB treatment *
- Time off work or study due to illness/treatment
- Quality of life
- Health status
- Respiratory disability

Patient perspective: Safety
- Grade 3 or 4 adverse event *
- Serious Adverse Event
- Death

Programme perspective
- Adherence to TB medication
- Treatment default
- Acquired drug resistance *
- Community transmission risk

Other
- Body weight
- Body mass index

4.1.2 Implementation of the management strategy

Overall evaluation
- Treatment burden
- Symptom burden
- Microbiological disease burden

Initial treatment
- Initial treatment adherence
- Main method of treatment supervision used in initial treatment

- Symptom prevalence
- Time to symptom clearance
- Smear prevalence
- Time to smear clearance
- Cuture positive prevalence
- Time to culture conversion
- End of treatment clinical status
- New drug resistance during initial treatment
- Reason for switch / extension with standard treatment
- Premature cessation of initial treatment
- Excess initial treatment

Monitoring

- Frequency of symptom assessment
- Frequency of smear assessment
- Time from end of treatment to clinical disease progression

Re-treatment

- Time from relapse (from first positive culture in cuture confirmed treatment failure/relapse) and start of re-treatment
- Disease severity at time of start of re-treatment
- Premature cessation of retreatment
- Time from end of treatment to clinical disease progression
- Number of relapse episodes

4.1.3 Health economics of the management strategy

- Utilisation of healthcare resources
- Direct medical costs
- Total (direct and indirect) costs
- Health status

4.1.4 Efficacy and safety of the regimens

Primary

- Unfavourable outcome by week 96

Secondary endpoints (main endpoints marked with *)

- Time to unfavourable outcome*
- Time to treatment failure/relapse
- Change in CXR % lung involvement from baseline to week 8
- Serious adverse event
- Grade 3 or 4 adverse event *
- Grade 3 or 4 adverse event considered related to TB drug(s)

- Grade 3 or 4 clinical adverse event causing permanent dose reduction or cessation of TB drug(s)
- Drug toxicity of special interest, derived from medDRA.

4.1.5 Bactericial activity of the boosted regimens

- Time to sputum culture conversion within 8 weeks from randomization*
- Sputum culture conversion status at week 8
- Change in time to positivity in sputum culture from baseline to week 8

4.2 Baseline visit and windows for assessments

The baseline day (also abbreviated to "baseline"; and referred to as Day 0 in the trial schedule) is the day when the patient attended for their baseline symptom and event review.

The protocol requires that randomisation be done in the clinic on the baseline day. In the event that randomisation is performed before or after the baseline day (e.g. the baseline symptom and event review were performed but randomisation could not be done at that time due to system connection issues) the baseline day will remain as the definitive start of the patient's participation in the trial and the basis for calculating windows for time-specific follow-up assessments.

Baseline values that are used to define changes in laboratory and other measurements (e.g body weight) over time for each participant are defined as the nearest measurement to the baseline day, within the window of 14 days before to 1 day after baseline, with exceptions listed below.

For laboratory parameters that may show acute changes in response to drugs, any values within the window prior to or at baseline will be used in preference to a value obtained on the day after baseline (which will be used only of nothing else available).

Baseline QTcF will be the absolute QTcF value from the screening ECG; if no ECG was done at screening the QTcF value from the baseline day will be used (section 4.7.4).

Baseline CXR values will be from the CXR performed on the baseline study day; if no CXR was performed on the baseline day then the CXR from screening will be used as the baseline CXR. An additional *expanded baseline CXR* evaluation will be used in which the maximum extent of disease and the maximum cavity size will be determined from all available CXR films taken between screening and baseline + 1 day (section 4.7.3).

Baseline smear will be the result of the test done at baseline. If no test was done at baseline, the test result obtained closest to the baseline day will be used, within the window from screening to baseline + 1 day. A second classification, termed *expanded baseline sputum smear* will also be used, defined as the maximum grade of all smear results obtained between screening and baseline + 1 day.

Baseline GeneXpert will be the result of the test done at screening.
If no test was done at screening, the test result obtained closest to the baseline day will be used (within the window -28 to +1 days from baseline). A second classification, termed *expanded baseline GeneXpert* will also be used, based on the lowest CT from all tests performed within the same window around baseline (see section 4.9.6).

Baseline *MGIT culture status* will be result of the culture done at baseline. Where the baseline culture is missing or contaminated, the first available culture value up to week 2 will be used as the baseline culture status. This will be used for comparison of randomised groups at baseline.

Baseline *MGIT culture positive status* will be defined as any positive culture (positive; or positive and contamined) observed from baseline to the week 2 visit. This will be used for comparison of the randomised groups at baseline and for determining eligibility for inclusion in the mITT exposed culture positive population (section 6.5).

Baseline TTP will be the result obtained from the baseline MGIT culture only (but alternative definitions may be used in biomarker analyses; section 13.7)

Baseline drug susceptibility profile will be that of the isolate from the baseline MGIT culture. Where there is no isolate available from the baseline culture, the first susceptibility result obtained on the MGIT culture up to the week 2 visit will be taken as the drug susceptibility profile. If there are no results for DST available at or before week 2, the results of later tests during the initial treatment episode may be used to infer drug susceptibility to particular drugs at baseline but not to infer drug resistance (baseline status will be marked as unknown for such drugs where later resistance is detected).

4.3 Other follow-up visits and windows for assessments

Follow-up visits between baseline and week 96 visit

Laboratory measurements, and other clinical parameters (e.g. body weight), at any nominal week of a scheduled visit (specified in Trial Schedule, section 1 of the trial protocol) are defined as those taken nearest to the nominal week at which the measurement is scheduled to be done. The midpoint between two scheduled visit weeks at which a measurement is due should be taken as belonging to the latter window (see below).

Where there are two values within one of these windows and the values are equidistant from the nominal visit week, the later value will be used.

Follow-up vists after week 96

For patients that commence re-treatment in the trial at or before the week 96 visit (or after the week 96, if the relapse was present at the week 96 visit; but not for re-treatment commencing after week 96 for relapse occurring after week 96 or for other reasons), protocol-mandated

follow-up continues for 48 weeks after the start date of re-treatment. In some cases, this means that follow-up visits extend beyond week 96 – termed *post week 96 re-treatment follow-up visits*. When the participant completes follow-up to 48 weeks post-re-treatment, a *post week 96 re-treatment follow-up final visit* will be performed at which the assessments scheduled for week 96 will be repeated. Data collected at *post week 96 re-treatment follow-up and final visits* (to the extent they are performed prior to the closure of the trial at each site) will be used in a supplementary analysis of secondary outcomes for the analysis of the management strategy (section 8.7).

4.4 Week 96 visit and windows for assessments

<u>Windows</u>

The week 96 *protocol-mandated visit window* is 96 ± 4 weeks (i.e. from week 92 to week 100; day 644 to day 700 inclusive).

The week 96 *analysis window* is from 6 weeks before week 96 (midway between the week 84 and 96 visits, following the rule in section 4.3) and 12 weeks after week 96 (i.e. from week 90 to week 108; day 630 to day 756 inclusive).

<u>Data from in-person visits to the site at week 96</u>

If there are multiple in-person visits within the window, the visit closest to week 96 (Day 672) will be taken as the definitive week 96 visit date. Where there are two visits within the window that are equidistant from Day 672, the second visit will be taken as the definitive visit (with the results of any assessments not done at the second visit carried forward from the first visit).

If protocol-mandated assessments (including investigations) are omitted on the day of the definitive visit but are performed earlier or later within the analysis visit window, then the closest of these assessments to Day 672 will be counted as the definitive week 96 value for that assessment.

Once a definitive week 96 value has been obtained on an assessment, subsequent values for that assessment, if any, will be ignored. There are two exceptions to this:

- Patient vital status (alive or dead): if the patient attends their week 96 visit in person (within the analysis window) but is known to have died subsequently before the end of the analysis window then they will be counted as a death during study follow-up to week 96.
- Microbiology tests: the results of all microbiology tests done within the week 96 analysis window will be taken into account in the assessment of the primary (composite) outcome, although the definitive result for each protocol-mandated assessment will follow the rules above (i.e. the value closest to the study week will be taken as the result).

If there is no assessment within the week 96 analysis window, then the assessment will be considered missing at week 96.

Data from in-person home visits performed at week 96

Where there is no in-person clinic visit during the week 96 analysis window, or when specific assessments are not done at the in-person visit during the week 96 analysis window, then data on those assessments gathered at a home visit performed during the analysis window will be taken as the definitive data for week 96 (provided that the patient was seen and assessed at home in person by site staff). This may include the result from a CXR performed at an imaging facility (not the usual site imaging facility); and the results of any sputum samples sent for processing at the site laboratory. The results obtained from sputum samples sent for processing at a non-study lab will be considered as supporting evidence but will not be formally used in the study analyses.

Data from in-person (patient) telephone visits performed at week 96

Information gathered at patient telephone visits (including electronic media communication) within the week 96 analysis window may be used in the analysis, but will be considered as definitive only:

- If there is no in-person clinic visit or in-person home visit within the analysis window, or an in-person visit was performed but specific assessments deemed assessable by phone were omitted AND
- For specific variables that are deemed to be suitable for adequate assessment by phone, namely assessment of symptoms and adverse events, use of TB medication, healthcare utilisation, health status (EQ-5D), MOS-HIV, patient acceptability questionnaire, and soeconomic evaluation.

Data from friend/relative telephone visits performed at week 96

Information gathered at friend/relative telephone visits (including electronic media communication) performed during or after the week 96 analysis window will only be considered as definitive in respect to a report of vital status (alive or dead) at week 96. If the friend/relative reports seeing the patient in person on a date within the analysis window (if the date is only known to the nearest month, it will be considered in the analysis if the week 96 analysis window includes one or more days during that month). If there is more than one applicable friend/relative telephone visit, the report where the patient has been seen in person by the friend/relative closest to the scheduled week 96 visit will be used. If the dates are equidistant, or dates are not certain (but considered likely to be within months of the visit window), use both reports in making the judgement of the primary outcome. If either report states that the patient has TB symptoms and/or is on TB treatment, take this to be the result for the visit. Other than vital status, information gathered from a friend/relative telephone visit will be used only indirectly for determination of the primary outcome (unsatisfactory outcome) using the decision algorithm described in section 4.12.2.

4.5 Terminology used for randomised arms

<u>Terminology based on randomised strategy and boosted regimen</u>

The following terminology will be used throughout this SAP and the accompanying trial reports.

Randomised treatment arms will be referred to as “arms” rather than “groups”.

TRUNCATE-TB Management Strategy arms (abbreviated TMS) will be used to refer to the experimental arms. Standard Management Strategy arm (abbreviated SMS) will be used for the control arm.

The general term for the novel drug regimen(s) used in the TMS arms is “boosted regimen(s)”. The general term used for the standard-of-care drug regimen (modified as needed for drug susceptibility or toxicity) used in any of the arms (from randomisation in the SMS arm; after switch or relapse in the TMS arms) is “standard regimen”.

For the analyses of strategy, the terms TMS arm and SMS arm will be preferred to emphasise that the primary comparison is of the treatment strategy with the standard strategy, not a comparison between a boosted regimen and standard regimen. For the analyses comparing boosted treatment regimens with the standard regimen, identifying the arms by the drug regimen abbreviation may be preferred.

For the TMS arms, the boosted regimen is identified by the key sterilizing drugs that differentiate that regimen from the other regimens (isoniazid, pyrazinamide are common to all arms; ethambutol common to all arms except arm D).

SMS [RHZE] arm; or *RHZE* arm
[Protocol Arm A: (Standard treatment)]

TMS [hRIF/LZD] arm; or *hRIF/LZD arm*
[Protocol Arm B: (High-dose Rifampicin/Linezolid, with HZE)]

TMS [hRIF/CFZ] arm or *hRIF/CFZ arm*
[Protocol Arm C: (High-dose Rifampicin/Clofazimine, with HZE)]

TMS [RPT/LZD/LFX] arm or *RPT/LZD/LFX arm*
[Protocol Arm D: (Rifapentine/Linezolid/Levofloxacin, with HZ)]

TMS [BDQ/LZD] arm or *BDQ/LZD arm*
[Protocol Arm E: (Bedaquiline/Linezolid, with HZE)]

Terminology based on whether recruitment was completed or discontinued to a particular arm

The MAMS design plans for cessation of some TMS arms prior to full enrollment. These arms will be treated differently in some analyses and require to be identified as such in this analysis plan. The following terminology will be used to differentiate treatment arms throughout this plan and in study reports.

Complete TMS arm: an arm to which enrolment was continued until reaching the target sample size

Partial TMS arm: an arm to which enrolment was discontinued before reaching the target sample size

4.6 Treatment courses, adherence, and observation

Various definitions to quantify treatment administration are needed for this trial, necessitated by:

- The flexible duration of initial treatment required by the strategy
- The extensions and switches
- The inclusion of repeat treatment courses as part of the strategy
- The need to differentiate between amount of time on a boosted regimen and on a standard regimen within the same patient

Treatment duration, daily doses and adherence parameters variously function as outcome variables (as essential components of composite outcomes, and also as individual outcomes in their own right); as variables used for defining analysis populations; as determinants of the end of follow-up or point of censoring; and as variables used for adjustment in prediction models of other outcomes.

Three main parameters of treatment administration will be used in the analysis. These are summarised below and further elaborated in subsequent sections

- ***Treatment days:*** number of qualifying days on which treatment is taken, defined below
- ***Treatment duration:*** number of calendar days between first and last day of a treatment course, defined below.
- ***Adherence*****:** the proportion of qualifying treatment days over a specified time, including:
    - the first 8 weeks of the trial
    - the duration of a single treatment course
    - the total of all treatment courses

These related parameters each provide important independent information about treatment. The duration of a treatment course likely provides the best overall indicator of the burden placed on a treatment programme and patient to deliver a course of treatment. The qualifying treatment days in a treatment course likely has a stronger association with efficacy than does the total

duration of a treatment course. However, the efficacy arising from the qualifying treatment days may be reduced if they are spread over a longer duration, with interspersed missing days, than if they are taken continuously i.e. the effects may be reduced by poor adherence.

4.6.1 Treatment days

A *qualifying treatment day* is a day, from the start day of the treatment to the end day of treatment in a treatment course (defined in 4.6.4 below), when treatment is taken with the allocated boosted regimen or standard regimen, with drugs and doses that do not meet the definition of a *missed dose* (definition specific for each regimen, following rules given below).

A *missed treatment day* is a day, from the start day of the treatment to the end day of treatment in a treatment course (defined in 4.6.4 below), when the definition of missed dose is met for one or more of the prescribed medications in the allocated regimen. The definition of missed treatment day does not attempt to distinguish between the causes of a missed day; whether due to by patient non-adherence; or caused by temporary clinician-mandated dose changes or treatment interruption with later return to subsequent qualifying dose.

For the purposes of real-time management in the trial – counting the qualifying treatment days until treatment can be stopped – the definition of missed treatment day includes days when some TB treatment was taken (but not meeting the definition of qualifying treatment day) and days when no treatment was taken. This is intended to be conservative so that the reciprocal number of qualifying days is not overestimated.

The main analyses will follow this definition of missed treatment day. Additional analyses may be conducted in which missed treatment days will be sub-classified as :

- *Missed treatment day (complete)*: a day on which no TB treatment was taken
- *Missed treatment day (partial)*: a day on which some TB treatment was taken, but not meeting the definition of qualifying treatment day.

<u>Classification of missed drug doses</u>

A missed dose of an individual drug is defined as follows:

For the boosted regimen:
Ingestion of less than 50% of the protocol-recommended starting dose of a drug in the boosted regimen on a particular day (exceptions below)

For the standard regimen:
Ingestion of less than 50% of the dose of a drug in the <u>prescribed</u> standard regimen on a particular day. The prescribed standard regimen may include a fluoroquinolone in place of one of the standard drugs or in addition to the standard drugs (for resistance or toxicity reasons). The definition applies to standard regimen taken by patients randomised to the SMS arm; or

randomised to TMS arm following the point at which they have made a definitive switch to the standard regimen* (prior to this point the classification of missed doses will be judged against the dosing requirements of the boosted regimen); or when they are taking re-treatment.
The following do not count as missed doses (in either boosted regimen or standard regimen):

- Rifampicin dose reduction to standard dose (weight-based)
- Clofazimine dose reduction or cessation once 42 daily doses taken
- Linezolid cessation once 42 daily doses taken
- Ethambutol dose reduction or cessation at any time

*Definitive switch to standard regimen
This is defined as the first day when any TB drug in the standard regimen is ingested after the last qualifying day of the boosted regimen; first day includes missed dose (partial) but excludes missed dose (complete)). However, see the exception for patients in Arm B and C in section 4.6.4.

4.6.2 Trial-wide regimen changes, impact on treatment days

Systematic, trial-wide, dose changes may be required during the trial to address toxicity or other concerns and will be triggered by a recommendation from the Trial Steering Committee and notification to investigators. In such cases, patients allocated by randomisation to the affected regimen(s) will be classified by the initial regimen that they started following randomisation. In study reports, the numbers of such patients who started on the original dose but switched during the initial treatment period to the TSC-recommended modified regimen will be presented, along with the median duration (and range) of time spent on the original regimen before modification.

The thresholds for definition of missed dose will be modified only if they are incompatible with the TSC-recommended regimen change.

4.6.3 Number of treatment courses

Patients will be categorized by the number of treatment courses given: 1, 2 or ≥ 3 courses

The time to initiation of re-treatment will be calculated as time (days):

- From baseline to the first day of the re-treatment course (days)
- From the end-of-treatment day for the previous course to the first day of the re-treatment course

Re-start of treatment for relapse will always be regarded as a new treatment course. Re-start of treatment for reasons of premature cessation following inadequate initial treatment (without suspected relapse) will be regarded as part of the previous treatment course if the period of interruption is < 56 days; or as a new course of treatment if the interruption is ⩾ 56 days (classified as “default”). See further detail on treatment interruption and default in 4.6.4 below.

4.6.4 Start and end dates for treatment courses

Pre-baseline treatment:

Start day: the day when the first dose of a rifampicin-containing TB treatment regimen was taken, if any, before baseline. Any dose of rifampicin, irrespective of companion drugs, will count for this definition. Fluoroquinolone monotherapy, that may initially have been taken for presumed community acquired pneumonia, is not counted as, when given alone, it is unlikely to contribute substantially to sterilising efficacy of a regimen.

End day: the last day, prior to baseline, on which a dose of rifampicin-containing TB treatment was taken. For avoidance of doubt, if the start of the allocated boosted regimen in the initial treatment course is deferred until after the baseline day (or later), or both the pre- baseline standard regimen and the randomised boosted regimen are taken on the baseline day, the end day of the pre- baseline treatment course will still be on the day prior to baseline and the start day of the initial treatment course will still be on the baseline day (i.e. the division between pre- baseline treatment course and initial treatment course is determined by the baseline day, not by when the standard regimen is stopped and the boosted regimen is started).

Initial treatment course (any regimen)

The initial treatment course (any regimen) refers to the course of TB treatment starting at or after the baseline day, whether boosted regimen or standard regimen, and including any extension of treatment or switches from boosted to standard regimen (for persistent clinical disease, toxicity or other reasons); it is defined separately from the pre-baseline treatment.

The duration of the initial treatment course (any regimen), as well as the subsequent courses are mainly used to quantify the burden of administration of treatment on the patient and the health system as well as adherence.

Start day: the first day that any TB drug treatment was ingested on or after the baseline day. It does not matter whether the ingested drug or drugs are appropriate for the randomised group (e.g. if the patient is randomised to receive a boosted regimen and the standard regimen is started initially, this would still count as the first day); and irrespective of whether the drugs are taken at the full dose or reduced dose. In the (unlikely) event that no TB treatment at all is ingested on the baseline day, this would not count as the start day of treatment and the start day will be deferred until any dose of a TB drug is ingested.

End day: the last day when any TB treatment was ingested that counts as a qualifying day.

Initial treatment course (strict randomised regimen)

The initial treatment course (strict randomised regimen) refers to the course of TB treatment starting at or after the baseline day but with start and end dates limited to qualifying treatment days. The duration of the regimen is used predominantly for descriptive purposes and for calculating adherence to the boosted regimen. The latter purpose necessitates a stricter definition of start and stop dates, to avoid over-inflating the denominator.

Start day:
For the TMS arms, this is the first day on or after the baseline day that all drugs mandated in the allocated randomised boosted regimen are taken at a dose that is above the threshold to count as a qualifying day.
For the SMS arm, this is the first day on or after the baseline day that all the presecribed drugs in the standard regimen - that may include a fluoroquinolone in place of one of the standard drugs or in addition to the standard drugs (for resistance or toxicity reasons) - are taken at a dose that is above the threshold to count as a qualifying day.

In almost all cases, the start day (strict randomised regimen) will be identical to the start day (any regimen). It will differ in a few cases where the start day of the randomised regimen is deferred (e.g. for the bedaquiline regimen, if normalization of electrolytes is required); or if the patient continues on the standard regimen where this has been started before baseline (by mistake or deliberately, if they have already taken a dose of standard treatment on the baseline day), or does not start taking the boosted regimen at or above the required minimum doses.

End day:
For the TMS arms this is the last day on which the drugs in the strict randomised regimen are ingested at doses that count as a qualifying day.

Patients in Arm B (hRIF/LZD) and Arm C (hRIF/CFZ) who complete at least 42 days of linezolid or clofazimine but continue the standard regimen.

A specific definition for end day of the strict randomised regimen is required for such patient because, by the study definition of missed doses, the subsequent days on standard treatment following cessation of linezolid or clofazimine in Arms B and C respectively after 42 doses could be counted as either:

- Qualifying days of boosted treatment OR
- Qualifying days of standard treatment

Counting as additional qualifying days of boosted treatment may result in the patient exceeding the threshold of 54 qualifying days required to be included in the per-protocol analysis population used for the sensitivity analysis of the primary outcome (strategy analysis) or extending the time to censoring (regimen and microbiology outcome analyses). Also, for analyses of TMS arms in general, giving unequal weight across the treatment arms to an explicit decision to switch to

standard treatment or extend treatment on the calculation of the treatment duration (strict-randomised) could introduce a bias between arms.

Thus, for these specific cases (where linezolid or clofazimine end after 42 qualifying days and standard regimen drugs continue) the last day of the boosted regimen will be determined as follows:

If no explicit decision to switch to standard treatment is made prior to or at the week 12 visit, the end day of the strict randomised regimen will be the last day on which the drugs in the strict randomised regimen are ingested at doses that count as a qualifying day

If an explicit decision is made to switch to standard treatment prior to or at the week 12 visit, the end day of the strict randomised regimen will be:
The *latest* of:

- The date at which the first dose of standard treatment was taken following a decision to switch
  OR
- The last day on which the drugs in the strict randomised regimen are ingested at doses that count as a qualifying day <u>and</u> the dose of rifampicin is ingested at the protocol-mandated starting (high) dose

The end day for initial treatment course (strict randomised regimen) in the SMS arm is the last day that the standard regimen is ingested at doses that count as a qualifying day.

<u>Re-treatment courses</u>

Re-treatment for suspected relapse

Re-treatment for suspected relapse is always regarded as a new course of treatment.
Start day: the first day that <u>any</u> TB drug as part of a prescribed TB re-treatment regimen (whether as qualifying dose or missed dose (partial)) was ingested on or after the definitive end day of a previous treatment course.
End day: the last day that <u>any</u> TB drug as part of a prescribed TB re-treatment regimen (whether as qualifying dose or missed dose (partial)) was ingested for that treatment course.

Re-treatment following treatment interruption or default

Treatment interruption is defined as complete cessation of all drugs for less than 8 weeks (56 consecutive days; missed days (partial) do not count as cessation) starting before the patient reached the criteria for completion of the treatment course (section 4.6.7 below). Restarting treatment after interruption will be regarded as part of the previous course of treatment (unless for suspected relapse, in which case it will be regarded as a new course of treatment, as above).

Treatment default is defined as complete cessation of all drugs for more than 8 weeks (56 consecutive days; missed days (partial) do not count as cessation) starting before the patient reached the criteria for completion of the treatment course (section 4.6.7 below). Re-starting treatment after default will be regarded as a new course of treatment, with the start day and end day as for the re-treatment course above.

Start and end day of retreatment course (any regimen)

Start day / end day: The first and last day that all the presecribed drugs in the standard regimen used for re-treatment are taken at a dose that is above the threshold to count as a qualifying day.

4.6.5 Treatment duration

The duration of an individual treatment course is defined as the total number of days from the start day to the end day or a treatment course inclusive (as defined above).

For retreatment courses that continue beyond week 96, the duration will be censored at the date of the week 96 visit (data from *post week 96 re-treatment follow-up visits* may be used in a supplementary analysis; sections 4.3 and 8.7).

The duration of the following courses/ course components will be calculated for each patient:

- Pre-baseline treatment
- Initial treatment course (strict randomised regimen)
- Initial treatment course (any regimen)
- Total initial treatment course (pre-baseline and post-baseline, any regimen)
- Total re-treatment courses (any regimen)
- Total treatment courses (initial and re-treatment, any regimen)*

*Total treatment duration (any regimen) is the duration of all treatment courses (post-baseline) to the week 96 visit, or until follow-up is censored as defined in each analysis. It may also include post week 96 retreatment follow-up (used in a supplementary analysis; sections 4.3 and 8.7). Total treatment duration is a secondary outcome in the strategy analysis.

4.6.6 Treatment daily doses taken

The number of treatment daily doses taken in each treatment course will be the number of qualifying treatment days during that treatment course

The number of qualifying treatment days will be calculated for each of the following dosing periods (as defined above):

- Pre-baseline treatment
- Initial treatment course (strict randomised regimen)
- Initial treatment course (any regimen)
- Total initial treatment course (pre-baseline and post-baseline, any regimen)
- Total re-treatment courses (any regimen)
- Total treatment courses (initial and re-treatment, any regimen)

Qualifying days will be detetmined for the boosted regimen and standard regimen following the rules in 4.6.4 above, that define missed doses. In the TMS arms, qualifying days of the standard regimen will only be counted after the last qualifying day of the boosted regimen; standard treatment alone, taken between the first and last qualifying day of the boosted regimen, will count as missed days; even if there is a later switch to the standard regimen.

4.6.7 Categorical classifications for treatment completion and default

<u>Initial treatment course, treatment completion and default categories</u>

Patients will be classified by qualifying days of boosted or standard treatment taken during the initial treatment course (any regimen):

Qualifying treatment days (boosted)	Qualifying treatment days (standard)	Qualifying treatment days (total)	Description of dose completion
0	0	0	No treatment
<54	0	<54	Incomplete boosted
<54	>0	>0, <154	Incomplete boosted, incomplete standard
<54	>0	≥154	Incomplete boosted, complete standard
≥54	0	≥54	Complete boosted
≥54	>0	>54, <154	Complete boosted, incomplete standard
≥54	>0	≥154	Complete boosted, complete standard
0	<154	>0, <154	Incomplete standard
0	≥154	≥154	Complete standard

Shading in table is to indicate different duration of boosted treatment (no other significance)

The following aggregate categories will be determined for the initial treatment course (any regimen):

Completed treatment:
[Complete boosted] + [Incomplete boosted, complete standard] + [Complete boosted, complete standard] + [Complete standard]

Defaulted treatment:

[Incomplete boosted] + [Incomplete boosted, incomplete standard] + [Complete boosted, incomplete standard] + [Incomplete standard] + [No treatment]

An alternative definition of defaulted treatment will be used for descriptive purposes:

Defaulted treatment (including pre-baseline doses)
As for definitions above but counting any days of treatment taken pre-baseline into the total duration of standard treatment (for categories where at least one dose of post-baseline standard treatment is given).

For these definitions, determination of qualifying treatment days using rules in 4.6.4 above. Minimum of 54 and 154 qualifying days rather than 56 and 168 days for boosted and all treatment respectively, for reasons given above.

<u>All treatment default categories</u>

There are 3 definitions of default, according to the timing of the first day of default:

Treatment default (initial treatment course)
This is defined as above, day of default any time during the initial treatment course

Treatment default (first 8 weeks)
This is defined (for TMS and SMS arms) as cessation within 56 days following baseline, unless the patient ingested at least 54 qualifying days of boosted treatment before cessation and there was no documented decision to extend treatment prior to default.
This is the main definition of default used as a secondary outcome in the strategy analysis

Treatment default (any treatment course)
As for treatment default (initial treatment course), with the addition of the following to the criteria for TMS and SMS arms: cessation before ingestion of at least 154 qualifying days of treatment in the second or subsequent treatment course

Patients will be categorised as treatment default (yes/no) in each of these 3 course definitions.

4.6.8 Categorical classifications for treatment extension

<u>Initial treatment course, reasons for extending boosted treatment</u>
Participants randomised to the TMS arms who extended boosted treatment beyond 56 qualifying days will be classified into:

- Persistent clinical disease
- Other

Classification will follow reasons for extending boosted treatment reported by the sites at the week 8 and 10 visits; answers will be combined; participant can be classified under more than one category. The category of “missed dose” will be ignored (sites provided reasons for extension of boosted treatment beyond the fixed timepoint of the visit rather than beyond 56 doses)

Initial treatment course, reasons for starting standard treatment
Participants randomised to the TMS arms who take one or more doses of standard treatment following the end of the boosted regimen during the initial treatment course (as defined above) will be classified according to the categories reported by the site team

Classification will follow the reasons for starting standard treatment reported by the sites at the weeks 8,10 and 12 visits; answers will be combined; participant can be classified under more than one category.

4.6.9 Re-treatment after week 96

Information on post week-96 re-treatment episodes will be obtained from a close-of-trial telephone assessment, performed in all participants around the time of the last patient last visit and before the closure of the trial site. Patients will be classified by whether the re-started treatment post week 96 (yes or no) and, if yes, the time of restarting treatment. This will be reported as an additional analysis of regimens (section 11.6.3).

4.6.10 Adherence

Adherence during a course of treatment will be obtained from the total qualifying daily treatment doses taken (daily doses defined above, excluding missed doses) divided by the duration of the treatment course (various periods, see below).

Adherence will not differentiate between patient non-adherence to the prescribed regimen and clinician non-adherence to the protocol-mandated regimen (e.g. due to prescribed interruptions or dose-reductions for the management of toxicity) as it is often arbitrary whether interruption is initiated by the patient or the clinician.

Adherence as proportion of missed doses in a treatment course

Adherence will be expressed as percentage of days on which treatment was taken for the following 4 periods:

1) Initial treatment course (any regimen) to week 8 (day 56 inclusive).
Treatment days (numerator) is the number of qualifying days of initial treatment course (any regimen), as defined in section 4.6.4, taken during the period of assessment.

The period of assessment is from the start day of the initial treatment course (any), as defined in section 4.6.4, up to and including day 55 from randomisation (day 56 of treatment, if first dose is taken on baseline day) even if treatment stops before Day 55 (denominator).
This will be a main secondary outcome used for the strategy analysis.

2) Initial treatment course (any regimen) to end of treatment course
Treatment days (numerator) is the number of qualifying days of initial treatment course (any regimen), as defined in section 4.6.4, taken during the period of assessment.
The period of assessment is from the start day to the end day of the initial treatment course (any regimen), as defined in section 4.6.4. If the duration of the initial treatment course is less than 56 days in the TMS arms or 168 days in the SMS arm, the denominator for calculation of adherence will be set to 56 and 168 respectively.
This will be used as a descriptive parameter in the analysis of implementation of the strategy.

3) Initial treatment course (strict randomised regimen).
Treatment days (numerator) is the number of qualifying days of initial treatment course (strict randomised regimen), as defined in section 4.6.4, taken during the period of assessment.
The priod of assessment is from the start day to end day of the initial treatment course (strict randomised regimen). This will be used as a descriptive parameter in the regimen analysis, biomarker analyses and PK analyses.

4) Total treatment courses (any regimen)
Treatment days (numerator) is the sum of the qualifying days of treatment (any regimen) from baseline to week 96, as defined in section 4.6.4, taken during the periods of assessment of all treatment courses.
The period of assessment is the sum of the days from the start day to end day of all the treatment courses (any regimen) to week 96. This will be used as an additional secondary outcome parameter for comparing adherence between TMS arms and SMS arm in the strategy analysis.

<u>Adherence as category of number of missed doses in first 8 weeks</u>

Adherence will also be classified into categories for this initial period (baseline to Day 56) based on number of missed days: 0, < 7, 7-13, >14 days. Missed days are not required to be consecutive for this definition to be met (in contrast to the definition for real-time clinical management decisions).

Patients with > 7 missed days of treatment in the first 8 weeks will be classified as unassessable in the regimen analyses (section 4.13).

4.6.11 Treatment observation

The main method of treatment observation during the *initial treatment course* will be classified for each patient in two ways:

- Staff at site
- Staff at other clinic
- Trained lay provider
- Trained family member
- Video-observed therapy
- None (self-administered)
- Other

Each patient will be classified by method of treatment observation in two ways:
The main method used for observation in the month following initiation of treatment post-baseline.
All methods used as the main method for observation at any time during initial treatment.

These data are used for description of the intervention delivery in the main analysis (8.5.6) and for strategy implementation analyses (section 9.6).

4.7 Clinical observations and investigations (non-laboratory)

4.7.1 TB-related symptom checklist

A standard checklist of 6 TB-related symptoms is performed at 28 scheduled timepoints (baseline, 16 scheduled in person follow-up visits, 11 scheduled telephone visits).

The assessment asks whether 6 specific symptoms have each been present during the previous 7 days (fever, night sweats, cough, coughing up blood, chest pain (worse on breathing) and progressive involuntary weight loss).

A symptom will be considered to be absent if the response on the checklist is either:

- "No"
  OR
- Blank (neither "no" nor "yes") AND at least one other response on the checklist of 6 TB symptoms has been indicated as "no" or "yes"

The symptom checklist data are classified in 2 ways:

1) Presence or absence of each of the 6 individual TB symptoms
2) Total TB symptom score: the total number of the 6 symptoms that are indicated as present

All symptom assessments performed will be allocated to one of the 28 scheduled symptom assessment timepoints, irrespective of whether the mode of symptom assessment performed (in person or telephone) matches the protocol-mandated type of visit (in person or telephone visit) specified for that time point. Specific windows will be created around each scheduled symptom assessment timepoint, with the dividing point equidistant between the scheduled assessment

timepoints. Similar to the approach to allocating windows around the in-person study visits (section 4.3), the midpoint between two scheduled symptom assessment timepoints will be taken as belonging to the latter window (see below).

Symptom profile for analyses reporting symptom prevalence at specific visit timepoints:
Where there are several symptom assessments performed within the same window, these will be handled as follows:

- Count each symptom as present if it is indicated as present at any symptom assessment done within the window
- The total symptom score will be the highest score of any of the evaluations performed (but will not be recalculated based on symptoms present on different evaluations within the window).

Symptom profile for time-to-event analyses

- Time to clearance of TB symptoms is defined as the time from baseline (or the start of a re-treatment course) to the first of 2 consecutive symptom assessments performed (in person or telephone visit, and at least 2 weeks (14 days) apart) that have a symptom score of 0 (section 9.5.2). For the initial treatment course, the timepoints will be fixed according to the scheduled visit weeks (with grouping as above, if multiple assessments within window). For subsequent treatment courses, the analysis will use the timing of individual symptom assessments from the start day of treatment (as these may not follow an identical visit schedule between patients).
- Time to TB symptom recurrence is defined as the time from the end of the first treatment course (or the end of a re-treatment course) to the first of 2 consecutive symptom assessments performed (in person or telephone visit, the first at least 2 weeks after the end of the last course of treatment, the second at least 2 weeks (14 days) and no more than 12 weeks later than the first) that have a symptom score of ≥ 1 (section 9.5.4). A second definition will be used requiring a symptom score of ≥ 1 above the symptom score at end of the last course of treatment. This analysis will use the timing of individual symptom assessments from the start day of treatment (as these may not follow an identical visit schedule).

Symptom profile for imputing sputum culture results
As described in section 4.9.1 imputation will use the symptom assessment recorded at the visit where the sputum sample was due and will therefore be based on an individual result, rather than the aggregate data in windows.

Application of symptom checklist data in analyses
The symptom checklist data are used directly only as descriptive data in the strategy implementation analyses: overall symptom burden to week 96 (section 9.4.2); symptom prevalence and time to symptom clearance during initial treatment and status at end of treatment (section 9.5.2); feasibility and utility of symptom monitoring (section 9.5.4); feasibility and

utility of relapse assessment (section 9.5.4); disease severity at restart of treatment (section 9.5.5); and utility of reatreatment (9.5.6).

The symptom checklist data are also used to impute a sputum result as negative in some analyses (when the score is zero, signifying clinical disease recovery; section 4.9.1): strategy implementation analysis of overall microbiological disease activity (9.4.3), of initial treatment smear resolution and of culture conversion (9.5.2); regimen bactericidal activity analysis of sputum culture conversion and of TTP (4.9.9 and 12.6).

Note that symptom checklist data are not used in the disease activity clinical management criteria, disease activity research criteria, treatment failure criteria or relapse criteria which are derived from separate algorithms and so symptom assessments do not directly affect assessment of the primary outcome for strategy or regimen efficacy.

4.7.2 Severity of TB episode

This is categorised in two ways; based on overall interference with usual social and functional activity; and based on the maximum grade of the component events graded separately (section 4.8.6). Severity of TB relapses is used in analysis of strategy and regimen safety, and in the analyses of the implementation of the treatment strategy.

4.7.3 Chest X-ray

The findings from chest X-ray (CXR) examination are used for the following purposes:

- Eligibility assessment (evidence for TB diagnosis; cavity size; absence of extraplmonary TB)
- Baseline risk stratification for randomisation and adjustment of analyses
- Primary outcome determination for assessment of the strategy
- Subgroup analysis for primary outcome for strategy and regimen analysis
- Secondary analyses of regimen efficacy
- Analyses of routine clinical biomarkers for predicting sterilising efficacy

Baseline CXR: CXR performed on the baseline study day; if no CXR was performed on the baseline day then the CXR from screening will be used as the baseline CXR.

Expanded baseline CXR: all CXRs performed between screening and baseline + 1 day. The maximum extent of disease (bilateral > unilateral disease; extent of pleural effusion; proportion of total lung affected and extent of cavitation) will be determined from all available CXRs performed in this window; the maximum extent of each parameter may be from different CXRs.

Derivation of CXR parameters:

Standard CXR evaluation: parameters recorded by the clinical sites, following a standard approach given in the protocol and the manual of operations and recorded in real-time on CRFs. This will be the primary evaluation used in analyses.

Central CXR evaluation: review of CXR films will be conducted at the coordinating centre to confirm the key measured parameters (extent of lung disease, cavity size). These results may be used in sensitivity analyses of selected outcomes. Analyses using artificial intelligence reading of CXR films may also be performed for supplementary analyses.

4.7.4 ECG

QTc intervals will be values corrected by Fridericia's formula (corrected interval value designated as QTcF).

ECGs will be grouped by calendar day (ECG-day) for analysis (multiple ECGs may be performed on the same ECG-day). ECG days may be scheduled or unscheduled (see below).

Derivation of variables on an ECG-day:

Values, both absolute and categorical, will be assigned to each ECG-day as follows:

(i) Absolute QTcF
If the ECG is repeated on the same day (due to the initial ECG showing a QTcF > 450ms, or for other reasons) the QTcF (in ms) for that ECG-day will be taken as the mean of the QTcF intervals of all ECGs done on that day.

(ii) QTcF ≥ 500 (categorical)

- ≥ 500ms (confirmed): QTcF is ≥500ms on (at least) two ECGs taken on an ECG-day (i.e. will still meet the criteria if a third or more ECG is performed that shows QTcF <500ms; the order of ECGs does not matter).
- ≥ 500ms (unconfirmed): one (and only one) ECG was performed on an ECG-day and this had QTcF ≥500ms. Note if one or more additional ECGs are performed on that day that have QTcF <500ms this does not meet this criterion (in this case the QTcF is regarded as not above threshold)

(iii) QTcF ≥ 60 (categorical)
The QTcF change for each ECG-day (with the exception of the screening day) will be calculated from the difference between the absolute QTcF on that day (mean of all readings on the day, as above) and the screening QTcF (as below), to determine whether there has been an increase of ≥ 60 ms.

Scheduled and unscheduled ECG days

Scheduled ECG days are at screening, baseline, weeks 1, 4, and 8; and at week 12 and at end of treatment (in selected patients). Results are recorded in the CRFs.

Uncheduled ECGs days are days where one or more ECGs are performed for clinical reasons outside of the protocol-mandated visits; and that meet the definition of QTcF ≥500ms

(categorical; confirmed or unconfirmed) or QTcF change ≥ 60 (categorical); these are identified from adverse event reports of QTcF prolongation (reported as Grade 3 or higher) that are not otherwise recorded in a visit CRF. The variables will be derived based on values stated in the report (and limited to information in the report) with QTcF change ≥ 60 (categorical) recalculated using the screening value on the database.

Baseline QTcF

Baseline QTcF will be the absolute QTcF value from the screening ECG; if no ECG was done at screening the QTcF value from the baseline day will be used (section 4.2; section 4.7.4).

Derivation of summary QTcF values used in the analyses

The following values / categories will be allocated for each patient, based on the values of QTcF on ECG-days (scheduled or unscheduled) occurring on the baseline day (if the ECG is taken after the first dose of study medication) and at any time up to 30 days following the end of the initial treatment course (any regimen; last day comprises qualifying day or missed day (partial); as defined in section 4.6.4).

(i) Maximum QTcF on initial treatment:

The highest absolute QTcF for any ECG-day after the baseline day

(ii) QTcF ≥500ms (confirmed)

This category will be allocated if the patient has been categorised as QTcF ≥500ms (confirmed) on one or more ECG-days after the baseline day

(iii) QTcF ≥500ms (unconfirmed)

This category will be allocated if the patient has been categorised as QTcF ≥500ms (unconfirmed) on one or more ECG-days days after the baseline day, but has not at any point been classified as QTcF ≥500ms (confirmed).

(iv) QTcF change ≥ 60

This category will be allocated if the patient has been categorised as QTcF change ≥60ms at one of more ECG-days after the baseline day.

4.7.5 Spirometry

Evaluable spirometry assessment

The forced expiratory volume in one second (FEV1) and forced vital capacity (FVC) are measured at weeks 8, 48 and 96. If spirometry is deferred at week 96 to a later date when the

first 8 weeks of re-treatment is completed, the spirometry values at that later date will be the used in the week 96 analysis.

A spirometry measurement at a study visit will be evaluable if the following criteria are met:

- Measurement made using the standard spirometer (EasyOne Air)
- At least one measurement recorded that meets the criteria of acceptable quality as designated by the spirometer.

<u>Grading</u>

FEV1 and FVC will be graded (separately) for each spirometry assessment based on the number of acceptable measurements and the reproducibility of the two highest acceptable measurements of each parameter, using the ATS/ERS 2019 guidelines:

Table 10. Grading System for FEV_1 and FVC (Graded Separately)

Grade	Number of Measurements	Repeatability: Age >6 yr
A	⩾3 acceptable	Within 0.150 L
B	2 acceptable	Within 0.150 L
C	⩾2 acceptable	Within 0.200 L
D	⩾2 acceptable	Within 0.250 L
E	⩾2 acceptable	>0.250 L
	OR 1 acceptable	N/A
U	0 acceptable AND ⩾1 usable	N/A
F	0 acceptable and 0 usable	N/A

<u>Parameters used in the analysis</u>

The FEV1 and FVC at a visit will be the maximum value of all the technically satisfactory measurements obtained for each parameter (may not come from the same maneouvre).

Maximum FEV1 will be expressed as a percentage of the predicted value for an individual of that age, sex, height, and race (FEV1%) calculated using the Global Lung Function Initiative (GLI) 2012 equations. [Quanjer PH et al; Multi-ethnic reference values for spirometry for the 3-95-yr age range: the global lung function 2012 equations. Eur Respir J. 2012;40(6):1324–43].

FEV1% will be categorized based on GOLD stages for COPD as follows: mild (≥ 80%), moderate (50-79%), severe (30-49%) and very severe (<30%).[https://goldcopd.org/wp-content/uploads/2019/12/GOLD-2020-FINAL-ver1.2-03Dec19_WMV.pdf]

Respiratory disability (spirometry) will be defined as FEV1% < 50% predicted (i.e. severe or very severe on GOLD criteria).[Vestbo J et al. Global strategy for the diagnosis, management, and prevention of chronic obstructive pulmonary disease: GOLD executive summary. American journal of respiratory and critical care medicine 2013; 187(4): 34].

New respiratory disability at week 96 will be defined as FEV1 < 50% predicted at week 96 in participants who had FEV1 ≥ 50% predicted at the week 8 measurement (where no week 8 measurement is available for comparison at week 8, the participant will be non-evaluable for new respiratory disability at week 96).

4.7.6 Pre-existing diabetes

Patients will be classified as having diabetes at study entry if diabetes is recorded in the past medical history or diagnosed within one week following the baseline visit (reported as an adverse event).

4.8 Classification of adverse events

4.8.1 Classification rules used by sites

Severity of adverse events is assessed by the investigator using the DAIDS (Division of AIDS) 2014 toxicity grading scale.

A clinical adverse event is defined as any new, recurrent, or increased severity (on DAIDS grading scale) of a disease; a symptom; a physical sign; or a clinically-significant laboratory abnormality.

A clinically-significant laboratory abnormality is defined as one that suggests underlying disease and/or organ toxicity that is worsening; and/or requires additional active management e.g. change of dose, discontinuation of drug, new concomitant medications, close observation, more frequent follow-up assessments or further diagnostic investigation; and is above/below the threshold required to meet the DAIDS grade 1 threshold for an event (if a DAIDS threshold is given in the table) or is outside of the local laboratory normal range (if there is no DAIDS threshold).
A serious adverse event is defined as any adverse event that results in death; is life-threatening; requires hospitalization or prolongation of existing hospitalization; results in persistent or significant disability or incapacity; is a congenital anomaly or birth defect; or is an important medical condition. SAEs will be regarded as episodes, with all components of the same clinical SAE presented (and analysed) as one episode.

4.8.2 Central review of adverse events

Grade 3 and 4 AEs:
The event name, grade and justification for the grading provided by the trial site are reviewed centrally by the coordinating centre (blinded to participant ID and treatment arm) to verify that the grading appears to be appropriate. Sites are asked to consider alternative classifications where the grading appeared inappropriate; the final grading given by the site is used in the analysis.

SAEs:

All SAEs are reviewed centrally by the coordinating centre in real time to verify that the name, description, relationship of the event to drugs and TB disease reported by the site are accurate. Sites are asked to consider alternative classifications where this appears appropriate after central review but have discretion to make the final determination of what is recorded on the database.

4.8.3 Coding of adverse events

For analysis, all AEs are coded into Preferred Terms and grouped by System Organ Class (SOC), using the Medical Dictionary for Regulatory Activities (MedDRA version 19 or later).

4.8.4 Definition of incident adverse events

Pre-existing (non-incident) adverse events

Adverse events that have a start date prior to baseline will be regarded as pre-existing and will not be included in the analysis of incident adverse events in the trial.

Incident adverse events

Adverse events that have a start date or date of increase in grade from the baseline day and the day of the definitive week 96 visit (defined in section 4.4), inclusive, will be regarded as incident adverse events during the trial. For avoidance of doubt, events that have an onset date after the definitive week 96 visit day (but nevertheless lie within the week 96 analysis window) will not be regarded as incident adverse events during trial follow-up for the purposes of analysis at week 96 (but see exception for deaths below).

Deaths

All deaths that occur to the end of the week 96 analysis window, including any that occur after the patient attended for their definitive week 96 visit assessment (and within the week 96 analysis window), will be included in the analysis. Such deaths will be included in the total deaths in the CONSORT diagram (even though the patient completed follow-up to the week 96 visit); will be evaluated as unfavourable or unassessable according to definitions used for unsatisfactory and unfavourbale outcome; and will be included in the total of deaths (and SAEs) reported in the safety outcome tables.

Deaths that occur after the close of the week 96 analysis window will not be reported in the safety analysis. If an SAE with onset at or prior to the definitive week 96 visit day progresses to cause death after the close of the week 96 analysis window this will still be classified as a non-fatal SAE outcome in the safety table, but the additional death associated with an SAE will be noted in a footnote.

Definition for analysis of regimen safety

Adverse events that have a start date or date of increase in grade that lies between the baseline day and the end date of the initial treatment course (strict randomised regimen) plus 30 days

(inclusive) will be regarded as incident adverse events for the purpose of analysis of regimen safety. End date of the initial treatment course (strict randomised regimen) is defined in section 4.6.4.

Adverse events occurring after the definitive week 96 trial visit and reported at *post week 96 re-treatment follow-up visits* will be used in a supplementary analysis (sections 4.3 and 8.7).

4.8.5 Definition of relatedness

A binary classification of events into related (at least possibly related) and unrelated (equivalent to not related or unlikely related) will be used, as reported by the study site.

4.8.6 Counting of adverse events used in the safety analyses

Consolidation of event grades
In the analysis, any event (relapse or other event) that increases in grade will be consolidated into a single event at the higher grade, but with the onset date as the date the event started at the lower grade.

Site recording and grading of TB relapses
All events that met the clinical management criteria for TB relapse – whether based on symptoms, CXR findings and/or microbiology data, or that the site clinician considered to be a relapse, even though the criteria were not fully met – are reported as TB relapse. Where a relapse diagnosis was made and treatment was restarted outside of the trial site, and the site clinician does not consider the disease activity clinical management criteria have been met or that the episode constitutes a relapse, then the relapse is not reported as an AE.

TB relapse is reported both as single adverse event (graded according to impact on social and functional status by DAIDS criteria) and separately as individual components of the TB relapse event (each graded according to DAIDS criteria).

Analysis of TB relapses
For the analysis, the individually reported component events will be combined with the single reported TB relapse event as a single combined event termed *TB relapse aggregated event*. The criteria for events to be combined as a TB relapse aggregated event are:

- Onset (or increase in grade) within 60 days prior and 3 days after the start date of re-treatment for that episode of relapse AND
- Onset (or increase in grade) after the end of the last course of treatment *AND*
- One of the 6 standard TB symptoms (fever, night sweats, cough, coughing up blood (haemoptysis), chest pain, weight loss AND
- Classification as related (at least possibly related) to TB disease

In the analysis, overall severity of a TB relapse will classified in two ways (section 4.8.6):

- Grade allocated for the single event by the site based on impact on social and functional status (this will be the primary grade for the safety analysis).
- Maximum grade for any of the adverse events included in the *TB relapse aggregated event* category.

Death is classified as Grade 4 in the DAIDS 2004 criteria. Death where the cause is unknown will be counted as a grade 4 event. Where cause of death is known and and is itself a grade 4 event, the death will not be counted as a separate event to avoid double counting. If the cause of death is a grade 3 (or lower) event, it will be upgraded to a grade 4 event because it has led to death and counted as above.

4.8.7 Adverse events of special interest

Standardised medDRA Queries (SMQs) will be used to identify instances of specific toxicities of interest, as listed in the table below.

**Key toxicities of interest**	**SMQs**	**Drug(s) associated**
QTc prolongation	Torsade de pointes/QT prolongation; Cardiac arrhythmias	Bedaquiline, clofazimine, levofloxacin
Hepatic events	Biliary disorders; Hepatic disorders	Isoniazid, rifampicin, pyrazinamide, rifapentine, bedaquiline, linezolid
Skin discolouration	Nil	Clofazimine
Peripheral neuropathy	Peripheral neuropathy	Isoniazid, linezolid
Haematological toxicity	Agranulocytosis, Haematopoietic cytopenias	Linezolid, rifampicin
Ophthalmic toxicity	Optic nerve disorders	Ethambutol, linezolid

4.9 Microbiology variables

4.9.1 Sputum sample collection status

Sputum sample collection status is defined separately from the individual test results because the permutations of *no sputum collected* represent a standardised part of the classification of test results (non-collection may in some circumstances be imputed as a negative test result).

Sputum sample collection status will be classified at the following timepoints:

- Baseline and all scheduled visits listed in the protocol at which sputum collection is mandated (including two separate protocol-mandated collections at week 96, which will be separately classified).
- Additional end-of-treatment sample (this will usually be identical to a scheduled visit sample).
- Additional relapse sample(s) (these may be performed separately from scheduled visit weeks)

For all these timepoints, the sputum sample collection status will be classified as follows:

- Sputum sample collected
- No sputum produced
    - Sputum collection attempted, unable to produce
        - Participant has clinical disease recovery*
        - Participant does not have clinical disease recovery
        - Clinical disease recovery not assessed
    - Sputum collection not attempted, *met* omission criteria **
- Missed sputum collection
    - Sputum collection not attempted, *did not meet* omission criteria**
    - Visit not attended – missed visit; telephone visit; withdrawn; lost to follow-up; deceased

Notes on classification

* Clinical disease recovery defined as absence of any of the 6 TB-related symptoms during the previous 7 days on the symptom screen recorded at the visit where the sputum sample was due (i.e. a symptom score of 0; section 4.7.1).

** Met omission criteria requires all of the following criteria to be met:

- >6m after completion of last TB treatment course AND
- no productive cough or symptoms suggestive of relapse (taken as equivalent to absence of the 6 TB symptoms in the visit checklist) AND

- sputum attempts on 3 previous visits were classified as *attempted, unable to produce* or *not attempted, met omission criteria*

/

Re-classification (imputation) of no sputum produced as negative
The following two categories of no sputum produced may be re-classified (imputed) as negative in some smear and culture analyses (section 4.9.2 and 4.9.3 below):

- Attempted, no sputum produced, clinical disease recovery
- Not attempted, met omission criteria

4.9.2 Allocating sputum samples to visits

The results of all sputum samples collected (or sputum collection status where the attempt to collect was made, or a collection was not attempted at a visit where it was mandated) will be allocated to a visit week according to pre-specified visit windows (section 4.4). In addition to their allocation to specific visit weeks, the results of sputum samples will also be allocated to critical timepoints, within the windows specified below and under the various analyses and definitions.

Allocation to scheduled study visits

Each sputum result will be allocated to a single scheduled study visit week according to the windows specified in section 4.3

Allocation to critical timepoints

Each sputum result that falls within the window for a critical timepoint will also be allocated to that critical timepoint as follows:

- Baseline: window between screening (included) and baseline + 1 day.
- End of treatment (any treatment, defined in section 4.6.4): window 7 days before the end of treatment day to 7 days after the end of treatment day (inclusive); although in the analysis of treatment failure (section 4.9.10) eligible culture results will be restricted to a window of 5 days before and after the end of treatment.
- End of treatment (strict randomised, defined in section 4.6.4); as for above, with the same window but applied to the end day for the strict randomised regimen.
- Relapse episode: from first positive culture to start of treatment or two negative cultures terminating episode (section 4.9.11)
- Post week 96 re-treatment follow-up, final visit: window -14 days to + 14 days.

4.9.3 Interpreting multiple samples at a single visit / critical timepoint

In most cases, a single sample is mandated at a study visit / critical timepoint and determining the overall result for a specific test done at that visit is straightforward.

However, in some cases, there may be several results at a particular scheduled visit or at a critical timepoint. The approach to assigning a value for the visit or critical timepoint will depend on the needs of the specific analysis, as described in the derivation of microbiology parameters below, or in the analyses sections that use those parameters.

The general approach outlined below will be used to assign a value for that test result based on the "highest" result obtained (on any sample in the window) in the following hierarchical order:

- Positive (for sputum culture this includes positive, contaminated)
- Negative
- Negative imputed (for specific analyses only, selected samples reclassified from no sputum produced category)
- Contaminated /Other (applies to sputum culture only)
- No result (≥1 sample obtained but did not yield one of the above results]
- No sputum produced [at least one visit done, but no samples obtained within window]
- Missed sputum collection

Where the test provides a measure of disease burden, assign the value that indicates the highest disease burden on that particular test (i.e. highest smear grade, lowest cycle threshold and lowest time-to-positivity).

This approach will be used for study implementation analyses, described in section 9.4.3 and 9.5.2

Maintaining separate, independent results within a window

A number of analyses will treat the results of several samples within a specific visit or window independently because the timing makes a difference to interpretation e.g. time to culture conversion, end-of-treatent culture status, time to treatment failure/relapse, and time to unfavourbale outcome where the timing of samples may be of critical importance. These are described under the relevant sections.

4.9.4 Sputum smear status and grade

Sputum smear status

For all scheduled visits or critical timepoints at which a sputum smear test is mandated, the smear status will be classified as follows:

Smear status	Definition
Positive	Positive result of any grade (including "scanty").<br>Smear grade subcategories may also be presented for some analyses
Negative (observed)	Result from actual sputum sample

No result	Sputum sample not processed or test results not available
Negative (imputed)	Result imputed when no sputum production in patient that has recovered*
Not mandated	Sputum sample not processed for the test because not mandated
No sputum produced	As in section 4.9.1, above
Missed sputum collection	As in section 4.9.1, above

*Reclassification of "No sputum produced, clinical disease recovery/met omission criteria" as negative (imputed) will be done in selected analyses.

Where multiple smear results are available within the visit window, interpretation will follow the rules set out in section 4.9.3 above.

Baseline smear status
Baseline sputum smear will be the smear performed on the baseline study day. If no smear was performed on the baseline study day, then the test result obtained closest to the baseline day will be used, within the window from screening to baseline + 1 day. A second classification, termed *expanded baseline sputum smear* will also be used, defined as the maximum grade of all smear results obtained between screening and baseline + 1 day.

Smear grade
For positive smear results, the grade is recorded as categorical data as Scanty, Grade 1, Grade 2, and Grade 3.

4.9.5 Sputum smear conversion and reversion

Sputum smear conversion to negative will be defined as two consecutive negative smear results without an intervening positive. The algorithm will follow that used for sputum culture conversion (section 4.9.9) with the analysis interval used for the analysis of the implemenatation of the strategy (i.e. from baseline to 16 weeks). The additional definitions for sensitivity analyses (described for culture conversion) are not required.

Sputum smear reversion to positive will follow the definition of treatment failure/relapse (section 4.9.10 and 4.9.11) and will be defined as two consecutive positive sputum smear results obtained at or after the end of treatment, with the second (confirmatory) positive smear being done on a separate day from the first sample and at least 2 weeks after the end-of-treatment day and within 16 weeks of the first positive sample.

These definitions are used for the strategy implementation analysis

4.9.6 Sputum GeneXpert status and cycle threshold

General principles and classification

GeneXpert testing is performed using two separate types of assays – Xpert MTB/RIF (hereafter referred to as Xpert) and Ultra. The tests record the signal from rpoB probes (5 probes for Xpert MTB/RIF, 4 probes for Ultra) and, in the case of Ultra, from additional IS6110 or IS1081 probes. The manufacturer's algorithm for reporting the test as positive requires at least 2 rpoB probes to be detected for Xpert or for Ultra; or, in the case of Ultra only, if there is ≤ 1 rpoB probe positive but the additional IS6110 or IS1081 probe is positive, this is regarded as showing a low level of infection (termed "trace" by the assay).

For all visits or critical timepoints at which a sputum test is mandated for GeneXpert, the status will be classified as follows:

**GeneXpert status**	**Definition**
Positive	Positive result of any level (including "trace" if Ultra).<br>Results by CT categories may also be presented for some analyses (High, Medium, Low, Very Low, Trace)
Negative (observed)	Result from actual sputum sample
No result	Sputum sample not processed or test results not available
Negative (imputed)	Result imputed when no sputum production in patient that has recovered*
Not mandated	Sputum sample not processed for the test because not mandated
No sputum produced	As in section 4.9.1, above
Missed sputum collection	As in section 4.9.1, above

*Reclassification of "No sputum produced, clinical disease recovery" as negative (imputed) may be done in selected analyses:

- Week 8 GeneXpert status
- End of treatment GeneXpert status
- Relapse assessment GeneXpert status

Where multiple GeneXpert results are available within the visit window, interpretation will follow the rules set out in section 4.9.3 above.

The determination of the absolute result - positive or negative – follows the manufacturer's algorithm. This is used for trial eligibility and for assessments using the disease activity clinical management criteria and disease activity research criteria for the primary outcome.

Cycle threshold results

In addition to using the absolute GeneXpert test results (positive or negative) trial analyses will also utilise the quantitative data available from the cycle threshold (C_T) results.

The minimum cyle threshold (C_T) from the rpoB probes (but not the IS6110 or IS1081 probe) will be recorded for each test. For avoidance of doubt, this may include tests that have been reported as negative according to the manufacturers algorithm but that have a single rpoB probe positive.

The minimum C_T results will be used to classify the result from Xpert into 4 categories (<16, 16-22, >22-28, > 28) based on standard cut-offs reported by the manufacturer used to approximate disease burden (called high, medium, low and very low respectively)(ref Blakemore, AJRCCM, 2011:184 (9): 1097-84).

C_T results from Ultra will be classified into the same categories using the following equivalance table:

**Disease burden**	**Ct Xpert**	**Ct Ultra**
High	<16	15-18.9
Medium	16 – 22	19-24.9
Low	>22-28	25-28.9
Very low	>28	29-40

For analyses based on categorical data, the C_T results from Xpert and Ultra tests will be combined (using the 4 test-specific categorical thresholds as above). For the main analysis based on continuous data, C_T values from Ultra will be converted to the equivalent C_T results using a conversion formula derived from published comparative data (Chakravorty, J Am Soc Microbiol 2017). Additional sensitivity analyses may be performed with the results of the two tests separately (without conversion factor for the results of Ultra).

For statistical analyses, when TB is not detected by any of the rpoB probes (which would be reported as a negative test for Xpert, and either a negative or trace test for Ultra), a CT value of 40 (the highest value used to identify TB in the Xpert assay) will be imputed (ref Shenai S, PloSOne, 2016: 11(8); e0160062).

<u>CT results in windows</u>

See sections 4.9.2 and 4.9.3.

The baseline GeneXpert result will be from the test done at screening. A second definition will be based on the lowest C_T from all tests performed between screening and baseline +1 day (section 4.2), including tests that have just 1 rpoB probe positive.

The GeneXpert week 8 test result will be the lowest C_T from the test performed within the week 8 visit window from Week 7 to 9 (Day 49 – Day 63 inclusive).

The End of Treatment (EOT) test result will be the lowest C_T within the window of 7 days before or after stopping treatment, inclusive (4.9.3).

The relapse test result will be the lowest C_T within the relapse episode (4.9.3).

Baseline GeneXpert status

Baseline GeneXpert status will be the result of the test done at screening. If no test was done at screening, then the test result obtained closest to the baseline day will be used, within the window from -28 days to +1 day around baseline. A second classification, *expanded baseline GeneXpert* will also be used, based on the lowest CT from all tests performed within the window for baseline GeneXpert (section 4.2).

4.9.7 Sputum GeneXpert conversion and reversion

GeneXpert conversion will be defined as a negative result at end of treatment (or at week 8 if test not done at end of treatment) in a patient that had a positive GeneXpert at baseline (using the expanded baseline definition).

GeneXpert reversion to positive will be defined as at least one positive GeneXpert test result obtained at least 2 weeks after the end of treatment (designated as confirmed if a repeat test done within 16 weeks of the first; not required by the protocol; or unconfirmed if only one test) in a patient that had a negative GeneXpert test at end of treatment (or at week 8 if test not done at end of treatment).

4.9.8 Sputum Culture Status and Time to Positivity

For all visits or critical timepoints at which a sputum culture test is mandated, the culture status will be classified as follows:

Culture status	Definition
Positive	TTP<42, MPT64 positive, BAP negative<br>TTP result, categorised or absolute, may be used for some analyses
Positive, contaminated	TTP <42, MPT64 positive, BAP positive *
Contaminated only	TTP < 42, MPT64 negative, BAP positive or negative *
Other	TTP< 42, MPT64 not done, BAP positive or negative *
Negative (observed)	TTP ≥ 42
No result	Sputum sample not processed or test results not available
Negative (imputed)	Result imputed when no sputum production in patient that has recovered**
Not mandated	Sputum sample not processed for the test because not mandated
No sputum produced	As in section 4.9.1, above
Missed sputum collection	As in section 4.9.1, above

* Where TTP < 42 days but BAP has not been done, the BAP result will be assumed to be positive for the purposes of assigning a category. This does not affect the determination of the primary outcomes for the strategy analysis or regimen analysis or the analysis of regimen bacterical activity by time to culture conversion (TTCC), but it means that the value of TTP is treated as not evaluable for the analysis of regimen bactericidal activity by change in TTP model (section 12.8).

** Reclassification of "No sputum produced, clinical disease recovery" as negative (imputed) will be done in selected analyses.

Where multiple smear results are available within the visit window, interpretation will follow the rules set out in section 4.9.3 above.

Summary table of classification of MGIT culture results for efficacy analyses based on sputum culture results *

**TTP**	**MPT64**	**BAP**	**TB culture classification**	**TB culture status (for TTCC analysis)**	**TTP (for change TTP analysis)**
<42	+	+	Positive, contaminated	Positive	Impute TTP as < 42
<42	+	-	Positive	Positive	Use TTP as measured
<42	+	Not done	Positive, contaminated	Positive	Impute TTP as < 42
<42	-	+	Contaminated only	Not evaluable	Not evaluable
<42	-	-	Contaminated only	Not evaluable	Not evaluable
<42	-	Not done	Contaminated only	Not evaluable	Not evaluable
<42	Not done	+	Other	Not evaluable	Not evaluable
<42	Not done	-	Other	Not evaluable	Not evaluable
<42	Not done	Not done	Other	Not evaluable	Not evaluable
>=42	Not relevant	Not relevant	Negative	Negative	Impute TTP as ⩾ 42

* This is based on sputum samples that are collected and processed for culture.
For some analyses, an additional category of TB culture status negative (imputed) will be used - TTCC and change in TTP (both as sensitivity analyses)

4.9.9 Sputum culture conversion

General definition of culture conversion

Culture conversion is defined as attaining two consecutive negative sputum culture results without an intervening positive culture (i.e. ignoring all culture results classified as not evaluable) after baseline (i.e. not including baseline as one of the two) until the end of a specified analysis period (see below).

The date of culture conversion is the date of the first of the two consecutive negative cultures.

(i) Culture conversion for comparing bactericidal activity of the regimens

General definition above; specific parameter definitions below; analyses described in section 12.

In addition to the general parameters above, the following specific parameters are used to classify patients for culture conversion in this analysis:

Sputum classification

Sputum classification at each time point will use categories listed in section 4.9.4.
The following sputum results at individual visits will be classified as not evaluable:

- Sputum sample produced, classified as "contaminated only" or "other" (see table in 4.9.4)
- No sputum produced (defined in section 4.9.1)
- Missed sputum collection (defined in 4.9.1)

Analysis period

This is the period from baseline to the *earliest* of the following:

- The date of switch to standard treatment (for patients allocated to receive a boosted regimen, who switch for toxicity or other reasons; with the exception of ≤ 7 days of standard treatment followed by re-introduction of boosted regimen; and with the exception of doses of standard treatment given in Arms B and C following cessation of linezolid after at least 42 doses that count as qualifying days of boosted treatment [see section 4.6.4]).
- The date of addition of any anti-TB drugs not specified in the randomly-allocated regimen (with the exception of addition of a fluoroquinolone for documented or suspected drug resistance in the SMS arm).
- The date of the 7th missed day of treatment, in patients who have 7 missed days (consecutive or non-consecutive) of allocated treatment during the first 56 days following randomisation.
- The designated end of follow-up for that analysis, which is 8 weeks.

Censoring

If culture conversion is not achieved during the analysis period, follow-up will be censored at the date of the last evaluable sample obtained during the analysis period. Where this last evaluable sample is positive the outcome will be classified as censored at that time. Where this last evaluable sample is negative, the outcome will be classified as culture conversion at that time.

Sensitivity analyses

Two additional sensitivity analyses will be performed for the purposes of comparing bactericidal activity between regimens:

- Where culture conversion is not achieved during the analysis period and where the last evaluable sample is negative the outcome will be classified as censored at the time of the last evaluable sample [yields more conservative estimate of time to culture conversion compared with the main analysis].
- For any protocol-mandated visit where the culture status is classified as *no sputum produced, associated with clinical disease recovery* (defined in 4.9.1; *no sputum produced, met omission criteria* does not apply to patients on treatment), the sputum culture status will be reclassified as negative and analysed identically to an observed negative sample at those visits. For avoidance of doubt, if this creates the scenario where the last evaluable sample is re-classified as negative, and culture conversion has not otherwise been achieved during the analysis period, the outcome will be classified as culture conversion at the time of the last evaluable sample (as for the main analysis with the last evaluable sample being an observed negative)[yields less conservative estimate of time to culture conversion compared with the main analysis]

(ii) Analysis parameters for analysis of *implementation and performance of the strategy*

General definition above; specific parameter definitions below; analyses described in section 9.5.2.

Sputum classification

- As for bactericidal activity analysis (above), but using for the main analysis the imputation method specified as a sensitivity analysis for the bactericidal activity comparison (above) i.e. that for any protocol-mandated visit where the culture status is classified as *no sputum produced, associated with clinical disease recovery* (defined in 4.9.1; *no sputum produced, met omission criteria* does not apply to patients on treatment), the sputum culture status will be reclassified as negative and analysed identically to an observed negative sample at those visits.

Analysis period

This is the period from baseline to the 16 weeks (none of the above conditions for the analysis period of bactericidal activity apply because they are mostly encompassed by the selection of patients in the category of “complete boosted”, specified for this analysis; see section 9.5.2). The

later end date of 16 weeks – later than that used for the analysis of bactericidal activity of regimens- is to allow time for late culture conversion following cessation of the boosted regimen.

Censoring
As for main approach for regimen bactericidal activity comparison (above)

Sensitivity analysis
An additional analysis will be done using an analysis period ending with the day of end of boosted treatment.

4.9.10 Treatment failure analysis criteria

Treatment failure refers to:

- Failure at cessation of the initial course of treatment (strict randomised regimen) OR
- Failure at switch to standard treatment following the completion of the initial minimum period of boosted treatment.

Treatment failure is an outcome measure in the regimen analysis but not in the strategy analysis, although it will be presented in the analysis of the implementation of the strategy. The respective definitions are provided below.

<u>(i) Treatment failure at cessation of initial course of treatment</u>

The following *treatment failure analysis criteria* must be met:

- End-of-treatment sputum culture* positive**

  AND

- Second, consecutive (confirmatory) sputum culture positive taken more than 2 weeks after the end-of-treatment day and within 16-weeks from the end-of-treatment sputum culture positive***

  AND

- WGS of the isolate from the baseline sample and confirmatory sample, if available, do not indicate a different strain ****

Notes

* End of treatment sputum culture

The end of treatment sputum culture is the sample taken at the end of the initial treatment course (*strict randomised regimen*; defined in section 4.6.4); in the TMS arms this is limited to patients that do not switch from boosted treatment to standard treatment (i.e. are classified as *complete boosted only*; separate definition for those who switch is given below).

To be eligible, the end of treatment culture must be:

- Taken within a window of -5 days and + 5 days (inclusive) from the end of treatment day (as defined above)
- Taken after at least 54 qualifying days of boosted treatment (in TMS arms) or 154 qualifying days of treatment in the SMS arm.
- If there is more than one end of treatment culture meeting these criteria, then the end of treatment culture result will be taken as the latest evaluable result within the window (evaluable meaning positive, positive contaminated, or negative).

** Positive means positive only or positive, contaminated (section 4.9.8)

*** Confirmatory sample

  - A positive sample collected on the day of restarting treatment, but no later, is allowed as the confirmatory sample (provided meets the other specified criteria).
  - Consecutive means no more than 1 intervening (observed) negative culture result between the end-of-treatment sputum culture positive and the subsequent sputum culture positive. Intervening results that are not evaluable will be ignored (no imputation of negative result for *no sputum, no symptoms* will be done for this analysis).

**** Whole genome sequencing: when a strain obtained at or after the end of treatment differs by ≤ 12 SNPs from baseline, or where WGS is not performed or the results are inconclusive, this will count as treatment failure. When the only strain(s) differ by > 12 SNPs from baseline then this will be considered as reinfection (multiple samples with the same WGS that differs from baseline) or possible sample processing error (if single sample with WGS that differs from baseline) as described in 4.9.12. When WGS shows >12 SNP difference, *confirmed* treatment failure will be counted as unassessable in the analysis of unfavourable outcome and *unconfirmed* treatment failure will be ignored in the analyses of unfavourable outcome (as described in sections 4.12 and 4.13).

Time of treatment failure, definition of episode duration and number of cultures

The date of treatment failure (and start of treatment failure episode) will be the date of the positive end-of-treatment sputum culture.

The end of the treatment failure episode will be the date of the start of re-treatment; or, if treatment is not re-started, the date of the first of two consecutive negative cultures that are considered to terminate the failure episode (may be imputed if *no sputum, no symptoms*); or if treatment is not restarted and no two consecutive negative cultures obtained, the date of the last study visit.

The number of positive sputum cultures during the treatment failure episode is the total number of positive sputum samples (obtained on separate days) during the treatment failure episode (includes first and last samples).

Censoring

Patients on TMS arms who do not take 54 qualifying days of the boosted regimen prior to treatment cessation (54 dose minimum rather than 56 is to allow margin for miscalculation, as described in section 4.6.7) will be censored for the outcome of treatment failure (at cessation) on the day that the last dose of the boosted regimen was taken.

Patients on SMS arm that do not complete the course of standard treatment (defined as 154 doses, section 4.6.7) will be classified as censored for the outcome of treatment failure (at cessation) on the day of the last dose of the standard regimen was taken.

<u>(ii) Treatment failure at switch to standard treatment</u>

For patients that complete the boosted regimen (≥ 54 qualifying days) but instead of ceasing treatmemt met switch to standard treatment, treatment failure (switch) will be classified using a similar approach to treatment failure (cessation) in the following way:

- End of treatment sputum culture positive must be taken at or before (within a window of 5 days before) the end of treatment day for the boosted regimen, but whilst on the boosted regimen and not after switch.
- Taken after at least 54 qualifying days of boosted treatment
- If there is more than one end of treatment culture meeting these criteria, then the end of treatment culture result will be the latest evaluable result within the window (evaluable meaning positive, positive contaminated, or negative).
- No confirmatory sputum culture is required

Where the WGS done on the end of treatment culture differs by ≤ 12 SNPs from baseline, or where WGS is not performed or the results are inconclusive, this will count as treatment failure and as unfavourable in the analysis of regimen efficacy. When the WGS on the end of treatment culture differs by > 12 SNPs from baseline then this will be considered as possible sample processing error and not count as treatment failure (switch) in the analysis of treatment failure and will also be ignored in the analyses of unfavourable outcome (as described in sections 4.12 and 4.13).

Time of treatment failure (switch)

The date of treatment failure (switch) will be the date of the positive end-of-treatment sputum culture.

Censoring

Patients on TMS arms who do not take 54 qualifying days of the boosted regimen prior to treatment switch will be censored for the outcome of treatment failure (at cessation) on the day that the last dose of the boosted regimen was taken.

<u>Treatment failure after re-treatment courses</u>

Positive cultures at or after completion of a re-treatment course will be defined using similar criteria (based on those for SMS arm, above) but will be identified and reported separately as individual cases of treatment failure (after re-treatment).

4.9.11 Relapse

Relapse, described here, refers to relapse following the initial treatment course. Positive cultures following completion of a re-treatment course will be defined using similar criteria but will be identified and reported separately as “relapse (after re-treatment)”.

Relapse is an outcome measure in the regimen analysis and refers to recurrence of infection following cessation of treatment.

Relapse is not an outcome measure in the strategy analysis but will be presented in the analysis of the implementation of the strategy. The respective definitions are provided below.

All of the following *relapse analysis criteria* must be met:

- Participant does not meet the criteria for confirmed treatment failure

  AND

- Two consecutive positive sputum cultures are obtained after the end of treatment *

  AND

- Second (confirmatory) sputum culture positive done on a separate day from the first sample and within 16 weeks from the first positive sample **

  AND

- WGS of the isolate from the baseline sample and relapse sample(s) does not indicate a different strain ***

Notes

* Positive means positive only or positive, contaminated (section 4.9.8). Consecutive means not more than 1 intervening (observed) negative result between two positive sputum cultures. Intervening results that are not evaluable will be ignored (no imputation of negative result for *no sputum, no symptoms* will be done for this analysis).

To be eligible to be considered as relapse, the patient must have completed treatment with at least 54 qualifying days of boosted treatment (in TMS arms) or 154 qualifying days of treatment in the SMS arm.

** When there is a single sputum culture positive after the end of treatment and there is no subsequent positive or negative culture to confirm or negate the relapse prior to re-starting

treatment (samples collected on the day of restarting treatment are allowed), the outcome will be classified as *relapse (unconfirmed)* at the time of the single positive sputum culture.

When there is there is a single sputum culture positive after the end of treatment and no subsequent positive or negative culture within the 16-week period (if the patient does not restart treatment during this time), the initial single positive culture will be ignored. A subsequent positive culture will be considered afresh as a potential new relapse episode.

*** Whole genome sequencing: as described in 4.9.12, when a strain obtained at or after the end of treatment differs by no more than 12 SNPs from baseline, or where WGS is not performed or the results are inconclusive, it will be assumed that the sequences are the same (i.e. counts as relapse). When the only strain (s) differ by more than 12 SNPs from the baseline then the relapse episode will considered as reinfection (if multiple samples with the same WGS that differs from baseline) or possible sample processing error (if single sample with WGS that differs from baseline). In the case of *confirmed* relapse, this will be counted as unassessable in the analysis of unfavourable outcome; in the case of *unconfirmed* relapse this will be ignored in the analyses of unfavourable outcome (as described in sections 4.12 and 4.13

<u>Time of relapse, definition of episode duration and number of cultures</u>

The date of relapse (and start of relapse episode) will be the date of the first of the two consecutive positive sputum cultures that serve to meet the diagnostic criteria (confirmed or unconfirmed).

The end of the relapse episode will be the date of the start of re-treatment; or, if treatment is not re-started, the date of the first of two consecutive negative cultures that are considered to terminate the relapse episode (may be imputed if attempted but no sputum available and no symptoms); or if treatment is not restarted and no two consecutive negative cultures have been obtained, the date of the last study visit.

<u>Censoring</u>

For the definition used for *regimen analysis:*

Exactly as done for the definition of treatment failure (above), patients on the TMS arms who do not take 54 doses of the boosted regimen prior to treatment cessation or switch to standard treatment will be classified as censored for the outcome of relapse on the day that the last dose of the boosted regimen was taken.

When at least 54 doses of boosted treatment have been taken but treatment is extended with standard treatment instead of stopping treatment, then the patient will be censored for the outcome of relapse on the day of the last dose of the boosted regimen (note difference from analysis for treatment failure where they may be classified as treatment failure (unconfirmed) on the last day of the boosted regimen).

Exactly as done for the definition of treatment failure (above), patients in the SMS arm that do not complete the course of standard treatment (defined as 154 doses, section 4.6.7) will be classified as censored for the outcome of relapse on the day of the last dose of the standard regimen was taken.

Patients in either arm that do not meet the definition of relapse (confirmed or unconfirmed) will be classified as censored for the outcome of relapse on the day of their last in-person visit or on the day that treatment is restarted (whicher occurs earlier).

4.9.12 Interpretation of positive culture results by whole genome sequencing

Whole genome sequencing (WGS) is performed on all isolates from positive baseline cultures (or at first available positive culture during initial treatment, if the baseline culture is negative) and all isolates from positive cultures obtained at or after week 8 of the trial. Additional sequencing may be performed on selected isolates obtained between baseline and week 8 during the initial course of treatment with the primary purpose of investigating differential drug resistance evolution between arms (see below).

Sequences that differ by more than 12 Single Nucleotide Polymorphisms (SNPs) will be considered as different strains (Walker et al, Lancet Respir Med 2014). These may represent reinfection or may indicate a sample processing error. Sequences that differ by more than 12 SNPs may be examined more closely by phylogenetic analysis and for the possibility of mixed infection. Supportive descriptive data may be provided but the classification rules for analysis will remain the same.

For analysis of treatment failure (end of treatment), treatment failure (switch) and relapse episodes, when a strain obtained at or after the end of treatment differs by no more than 12 SNPs from baseline, or where sequencing of samples is not performed or the results are inconclusive, it will be assumed that the sequences are the same (i.e. the case is treatment failure [end of treatment, or switch] or relapse, not re-infection). When the only strain (or strains) with WGS obtained in a treatment failure or relapse episode all differ by more than 12 SNPs from the baseline then any associated putative treatment failure or relapse episode will considered as reinfection (if multiple samples with the same WGS that differs from baseline) or possible sample processing error (if single sample with WGS that differs from baseline). In the case of *confirmed* treatment failure or relapse, this will be counted as *unassessable* in the analyses; in the case of *unconfirmed* treatment failure or relapse or in the case of treatment failure (switch), these will be ignored in the analyses (as described in sections 4.12 and 4.13).

For analysis of unsatisfactory or unfavourable outcome at week 96 based on ongoing disease activity research criteria at week 96, where a classification of definite or probable TB is made but the WGS strain(s) all differ by more than 12 SNPs from baseline, the event will be reclassified as reinfection or sample error and be counted as *unassessable* in the analysis. Where there is an isolated positive culture result at week 96 with no other evidence for ongoing disease activity and where the strain differs by more than 12 SNPs from baseline this will be *ignored* in the assessment of the primary outcome.

For analysis of sterilising bactericidal activity of regimens using outcomes of time to culture conversion and time to positivity, and for analyses of disease burden over time as part of strategy implementation analyses, where any strain obtained at or after week 8 differs by more than 12 SNPs from baseline this will be assumed to represent a contaminant will be ignored (treated as a non-evaluable sputum result) in these analyses.

Relapses after re-treatment courses

Positive cultures at or after completion of a re-treatment course will be defined using similar criteria (based on those for SMS arm, above) but will be identified and reported separately as individual cases of relapse (after re-treatment).

4.9.13 Drug resistance by MGIT

Drug resistance will be determined using the results of MGIT-based phenotypic drug susceptibility testing, with established cut-offs. Susceptibility testing to streptomycin is performed as part of routine testing but is not included in study analyses or reports.

“Confirmed drug resistance” at a particular trial time point requires two independent samples taken at separate times that show phenotypic resistance to the same drug by MGIT testing.

“Unconfirmed drug resistance" is based on a single sample that shows phenotypic resistance, where there is no other isolate available from a separate sample to confirm the resistance (a second sample that shows susceptibility would refute the case of drug resistance).

Additional drug susceptibility information is available from MIC testing, molecular testing and whole genome sequencing done during the trial on selected samples. All unconfirmed cases of drug resistance on phenotypic testing will be reviewed in context with any other results from molecular tests or Whole Genome Sequencing by the trial microbiologist to arriave at a final verdict. Where these test results are discrepant with the MGIT direct susceptibility tests they may be used to reject a case of “unconfirmed” drug resistance on MGIT testing (see below); but they will not over-rule a case of MGIT phenotypic resistance that had been “confirmed” on two separate samples.

Baseline drug susceptibility

Where there is no isolate available for drug susceptibility testing at baseline, the susceptibility profile of the first available isolate will be used to infer the baseline susceptibility profile. If the first available isolate tested for susceptibility is taken at or before week 2, this will be used to infer susceptibility or resistance at baseline. If the first available isolate is after week 2, drugs to which the isolate is shown to be susceptible will be inferred as susceptible at baseline; drugs that are shown to be resistant after week 2 will be designated as unknown susceptibility at baseline.

Where there are no MGIT drug susceptibility test results available from the patient at any follow-up time point, the patient will be assumed to have rifampicin-susceptible TB based on the original GeneXpert result obtained at screening.

Acquired drug resistance

Cases of drug resistance at follow-up will be classified as "confirmed" or "unconfirmed" based on the criteria above.

A case of "confirmed" acquired drug resistance will require the following evidence:

(i) Confirmed resistance to a drug at follow-up on two isolates; criteria as above.
(ii) Confirmed susceptibility to the same drug shown by MGIT testing on two separate isolates taken prior to the first of the resistant isolates.
(iii) Exposure to the relevant drug (or another drug known to cause cross-resistance) during the trial and preceding the first observation of drug resistance.

Where there is only one isolate showing drug resistance and/or one isolate showing susceptibility prior to the isolate with resistance, or where there is no known relevant drug exposure, the acquired drug resistance will be designated as "unconfirmed". Results from molecular tests and whole genome sequencing maybe used to reject a case of acquired drug resistance by DST where they show that resistance mutations explaining the phenotypic acquired drug resistance are present at baseline; or where they show that the follow-up resistant strain is not related to the earlier susceptible strain. All possible cases of acquired drug resistance are reviewed by a microbiologist for expert determination.

Drug resistance will be evaluated to the week 96 trial visit (and in a sensitivity analysis to the completion of the final trial visit).

4.9.14 Drug resistance by MIC plate testing

Additional data will be generated from plate MIC testing in a subset of participants.

The MIC data will be used to corroborate DST data obtained from MGIT and discrepancies resolved after review by an expert microbiologist. Plate MIC testing may be used to evaluate emergence of sub-threshold resistance to specific drugs. These will be described in a separate analysis plan.

4.9.15 Drug resistance by whole genome sequencing

As in 4.9.12 above, whole genome sequencing is performed on all isolates from positive baseline cultures (or up to week 2, if the baseline culture is negative) and all isolates from positive cultures obtained at or after week 8 of the trial. Additional sequencing may be performed on selected

isolates obtained between baseline and week 8 during the initial course of treatment to investigate differential drug evolution between arms. Recognized drug-associated resistance mutations will be identified by TB Profiler.

The WGS drug-resistance data will be used to corroborate DST data obtained from MGIT and discrepancies resolved after review by an expert microbiologist. WGS may be used to evaluate emergence of specific resistance mutations and to predict sub-threshold MIC changes. These will be described in a separate analysis plan.

4.10 Baseline Risk Stratum

The *baseline risk stratum* will follow the definition used in the protocol for stratified randomisation, based on smear, CXR cavities and HIV status determined at screening:

- Lower risk: screening smear negative and screening CXR cavities ≤ 4cm and HIV negative.
- Intermediate risk: screening smear positive (≤ 2+) and screening CXR cavities ≤ 4cm and HIV negative.
- Higher risk: screening smear positive (3+) or cavities > 4cm or HIV positive

In addition to being used for stratified randomisation, the *baseline risk stratum* will be used for description of baseline patient characteristics; for adjustment for baseline risk in the primary analysis of strategy and regimen efficacy; and for subgroup analyses. Any discrepancy between the baseline risk stratum reported by the site for the purposes of randomisation and the actual *baseline risk stratum* derived from the screening results (i.e. an error in the randomisation process) will be summarised in a table in the study report; all analyses will use the actual baseline risk stratum (derived from screening results).

Two additional definitions of baseline risk stratum may also be used for subgroup analyses:

Expanded baseline risk stratum: this will be based on the values of the expanded baseline smear and expanded baseline CXR classification as described in section 4.2. The expanded baseline risk stratum will be used for an alternative subgroup analysis of the primary outcome for strategy and regimen efficacy only if the proportion in the medium and high-risk categories (total combined) increases by more than an absolute of 10% from the definition used above.

Very low risk stratum: smear negative participants (in standard or expanded baseline dataset, depending on which is used) in the lower-risk category of the baseline risk stratum will be further sub-divided into:

- Those who have no CXR cavitation; allocated to *very low risk* stratum
- Those in whom cavitation is present (with maximum cavity size of ≤ 4cm) allocated to *low-risk* stratum

This will be used for subgroup analyses of strategy and regimen efficacy.

4.11 Disease activity criteria

There are four sets of overlapping criteria relevant to the assessment of disease activity and the primary outcomes used for trial analyses:

- Treatment failure criteria: described in section 4.9.10
- Relapse criteria: described in section 4.9.11
- Disease activity clinical management criteria: described in section 4.11.1
- Disease activity research criteria: described in section 4.11.2

The two sets of disease activity criteria overlap substantially but differ in important ways reflecting their purpose.

The *disease activity clinical management criteria* are determined in real-time at visits where relapse is suspected by the clinician. They are used for real-time clinical management decisions where culture results may not be available (and are therefore not required for the criteria to be met) and where a clinical decision therefore needs to give increased importance to more readily available indicators: symptoms, smear, GeneXpert or CXR. A subset of criteria, based on symptoms and smear only, are determined at weeks 8, 10 and 12 and are used for the real-time clinical management decision of whether to continue the treatment beyond those time points due to inadequate response.

The *disease activity research criteria* are research criteria used for the primary outcome definition for the strategy and regimen analysis at week 96 and are necessarily more rigorous than the *disease activity clinical management criteria*. They require a minimum duration of 7 days for symptoms to ensure that transient symptoms of short duration do not have overdue influence on the outcome (whereas no minimum duration is specified for symptoms used in the *disease activity clinical management criteria*). The *disease activity research criteria* also require at least one positive culture for the microbiological component of the criteria to be met (whereas the *disease activity clinical management criteria* do not, as stated above).

4.11.1 Disease activity clinical management criteria

These criteria form the basis of the real-time clinical management decisions made during the trial; in particular the decision that a relapse has occurred and the need to re-start treatment.

They go further than the monitoring approach for the treatment strategy, that is based on symptoms and smear only; they also incorporate the results of sputum cultures (routinely tested), GeneXpert and CXR (triggered by suspected relapse). Sputum cultures are not available in a programme setting and information may be incomplete at the time the decision is made, given the time required to perform the culture. Thus, these criteria neither reflect the situation in which the monitoring strategy might be used in the field (where decisions would need to be made without culture information) nor criteria that are definitive for research purposes.

The criteria are evaluated by the site clinician in real-time and, where relapse is suspected, are recorded on the relapse assessment form; with the components of the diagnostic criteria recorded on visit CRFs and laboratory test result forms.

Use in analyses:

Disease activity clinical management criteria are used in the following ways in the analyses:

For primary outcome at week 96 (strategy and regimen analyses)

- To determine ongoing requirement for TB treatment at week 96 in a patient that started treatment at or after week 72 but stopped it before week 96: criteria used to determine whether patient had evidence of disease activity *at the time of start of treatment* (impacts on the classification as unsatisfactory versus unassessable).
- To determine whether a patient that died prior to week 96 had evidence of ongoing TB disease activity *at their last visit* (impacts, with other criteria, section 4.12.1, on the classification of the death as unsatisfactory vesus unassessable).
- To determine whether a patient that did not attend in person for the week 96 visit had evidence of ongoing TB disease activity *at their last visit* (impacts, with other criteria, section 4.12, on the classification of the patient as satisfactory, unassessable, or unsatisfactory on the primary outcome).

For strategy implementation analysis

- To determine whether decisions to re-treat patients followed the trial criteria (i.e. were consistent with the disease activity clinical management criteria) and to estimate the lag time between meeting criteria and restart of treatment (section 9.5.5).

For the various analyses, the criteria are evaluated at fixed time points:

- At the time that treatment was re-started (or a decision to re-start treatment was made and treatment prescribed).
- At the last study visit for patients who died; or were not seen at week 96

Evaluation of the criteria in the analyses:

The evaluation of the criteria will take into account information from the study visit performed at or immediately preceding the required assessment timepoint; and will include any information from any relapse assessment or additional tests performed within 90 days prior to or 7 days after the assessment timepoint.

The criteria are described below:

A. Clinical Disease Progression

New, recurrent or increased severity of one or more standard TB-related symptoms (cough, one or more episodes of haemoptysis, fever, pleuritic chest pain, weight loss, night sweats) or physical signs compared with the end of the last course of TB treatment (or period after the end

of treatment if further improvement occurred subsequently) without alternative explanation(s) considered more likely in the opinion of the managing clinician.

B. CXR Progression

Presence of abnormalities that are compatible with active TB disease (cavitation, infiltrates, consolidation) with clear evidence of progression compared with CXR at end of the last course of treatment (or period after the end of treatment if further improvement occurred subsequently) without alternative explanation(s) considered more likely in the opinion of the managing clinician.

C. Microbiological persistence / progression
Sputum sample taken at or after the end of treatment is:

Smear positive OR

GeneXpert positive (if >24w after end of treatment; or if end of treatment test was negative) OR

Culture positive

D. Confirmed positive sputum culture

Sputum culture positive on two consecutive samples taken on separate days with at least one taken ≥ 4 weeks after end of treatment**.**

<u>Meeting criteria</u>

Patients will be considered to have met the criteria for clinical disease activity if at least 2 out of criteria A or B or C are satisfied (presumptive clinical disease activity), or if criterion D is satisfied (confirmed clinical disease activity).

Time that criteria were met: this will be estimated from the date of the specific assessments done that led to the criteria first being satisfied. For a culture result, the date will be adjusted for the lag time in receiving the result (TTP plus an additional 7 days for confirmation).

<u>Time to clinical disease progression</u>
This is the time from the end of last course of treatment to the time that criterion A is met. The date of clinical disease progression will be derived from the date of the visit at which the relapse assessment was conducted, minus the symptom duration reported at that time. In the case where the relapse assessment was conducted after the patient had started treatment (e.g. where treatment was started outside the study site) the date of clinical disease progression will be the date at which treatment was re-started. Time to clinical disease progression is used in strategy implementation analysis, utility of monitoring (section 9.5.4, and 9.5.5).

4.11.2 Disease activity research criteria

These criteria are used to determine the *ongoing disease activity* component of the primary outcome at week 96.

The criteria are assessed based on the clinical status at the definitive week 96 visit and the tests performed at that visit. The definitive week 96 visit is the first in-person visit within the protocol-mandated visit window; or if there is no visit within the protocol-mandated visit window, then use the first in-person visit within the analysis visit window (definitions in section 4.4).

Where the clinical status at the week 96 visit or the results of tests performed at the week 96 visit indicate a possibility of relapse and where a formal relapse assessment is then performed, the results of any assessments (clinical, imaging, microbiology) performed as part of the relapse assessment will be considered in the evaluation of the disease activity at week 96, provided that they were done within the week 96 analysis window.

The disease activity research criteria are classified as *met* if:

- At least 2 out of criteria A or B or C are satisfied
  OR
- Criterion D is satisfied.

The strength of evidence for ongoing disease activity is further classified as:

- Definitive: Criterion D is satisfied (with or without other criteria)
- Presumed: Criterion C is satisfied with at least one of A or B satisfied
- Possible: Criterion A and B satisfied only

Criteria:

A. Clinical evidence:

New, recurrent, or increased severity of one or more standard TB-related symptoms (cough, one or more episodes of haemoptysis, fever, pleuritic chest pain, weight loss, night sweats) for a duration of at least 7 days, or of physical signs that are suggestive of ongoing TB disease activity/ relapse, compared with the end of the last course of TB treatment (or period after the end of treatment if further improvement occurred subsequently) without alternative explanation(s) considered more likely in the opinion of the managing clinician.

B. CXR evidence

CXR taken within week 96 analysis window that has abnormalities that are compatible with active TB disease (cavitation, infiltrates, consolidation) and clear evidence of progression compared with CXR at the end of the last course of TB treatment (or period after the end of treatment if further improvement occurred subsequently) without alternative explanation(s) considered more likely in the opinion of the managing clinician.

C. Microbiological evidence

Observed sputum culture positive for *Mycobacterium tuberculosis* on 1 sample taken within the week 96 analysis window.

D. Microbiological evidence

2 positive cultures: Sputum culture positive for *Mycobacterium tuberculosis* on 2 samples taken on separate days with at least one taken within the week 96 analysis window [if only one positive sample in the week 96 window, examine the week 84 window for confirmatory sample]

OR

One positive culture plus other: Sputum culture positive on 1 sample taken within the week 96 analysis window AND a second sample taken on a separate day within the week 96 analysis window that is either:

Smear positive (only if taken > 12 weeks after the end of the last course of treatment) and/or

GeneXpert positive (only if taken > 24 weeks after the end of treatment or if end of treatment result was negative).

Extrapulmonary TB disease activity

The above criteria apply to evidence of ongoing pulmonary TB disease activity. The disease activity research criteria may also be met at week 96 if the patient fulfils equivalent criteria that indicate ongoing TB disease activity at another site i.e. non-pulmonary symptoms or signs, evidence of abnormalities on another imaging test that are compatible with active TB disease with evidence of progression from an earlier comparable imaging test (if no previous test available for determining progression, the abnormalities should be of sufficient severity to explain the symptoms), and microbiological evidence (presumptive or definitive) based on samples obtained from another site. However, the evidence from A, B and C (or the two samples positive for D) should relate to the same disease site.

Disease activity modified by whole genome sequencing

Whole genome sequencing (WGS) will be performed, where possible, in all new episodes of TB disease activity diagnosed throughout the trial and used to interpret whether the disease activity represents relapse of reinfection (or, in some cases, a sample processing issue), as described in 4.9.11 and 4.9.12 and as set out in sections 4.12 and 4.13 below.

4.12 Definition of unsatisfactory clinical outcome at week 96 (primary outcome for the strategy analysis

Unsatisfactory clinical outcome is the primary outcome for the strategy analysis, used to compare the efficacy of the TMS arms with the SMS arm.

Unsatisfactory clinical outcome at week 96 is a composite outcome chosen to represent a pragmatic view of the outcome of the management strategies from the patient and programme perspective.

This composite outcome is derived from a classification on the following 3 components:

- Ongoing requirement for TB treatment at week 96
- Ongoing TB disease activity at week 96
- Death prior to week 96

Each of these components will be classified as unsatisfactory or unassessable at week 96.

If any of the 3 components are classified as *unsatisfactory* the overall clinical outcome will be classified as *unsatisfactory*.

If none of the components are classified as unsatisfactory but one or more of the components are classified as *unassessable,* then the overall clinical outcome will be classified as *unassessable*.

If none of three components are classified as unsatisfactory or unassessable then the overall clinical outcome will be classified as *satisfactory*.

4.12.1 Classification of clinical outcome for patients who attend during the week 96 analysis window or are known to have died

As noted in section 4.4, for the purposes of the analysis of the primary outcome, the week 96 analysis window is from weeks 90 to 108 weeks from randomization (Day 630 to Day 756 inclusive). If there are multiple in-person visits within the window, the visit closest to week 96 (Day 672) will be taken as the definitive visit date. Where there are two visits within the window that are equidistant from Day 672, the second visit will be taken as the definitive visit (with the results of any assessments not done at the second visit carried forward from the first visit).

Where there is no in-person clinic visit during the week 96 analysis window, a home visit done by site staff will also count, provided that the patient was seen and assessed in person. In such cases, those assessments gathered at a home visit performed during the analysis window will be taken as the definitive data for week 96; this may include the result from a CXR performed at an imaging facility (not the usual site imaging facility); and the results of any sputum samples collected at home and sent for processing at the *site* laboratory. The results obtained from sputum samples sent for processing at a *non-study lab* will not be formally used in the study analyses.

All microbiology tests conducted within the analysis window (and done in the designated site lab) will be taken into account in the assessment of the primary outcome.

Classify as follows:

1) <u>Ongoing requirement for TB treatment at week 96</u>

Classify as unsatisfactory if the following are met:

- Is taking any combination of TB drugs when seen during the week 96 analysis window. [For avoidance of doubt, this requires simply that the patient is on treatment at week 96; there is no requirement to have met the diagnostic criteria at the start of the treatment course].

OR

- Has been prescribed a course a course of treatment for a new episode of active TB (meeting the disease activity clinical management criteria, defined in section 4.11.1 above) starting within 6 months (24 weeks) prior to the scheduled week 96 visit date (i.e. after week 72, day 504) but has stopped the drugs before the week 96 visit. If has been prescribed treatment but did not meet disease activity clinical management criteria at the start of treatment (or it is not known whether met those criteria at the start of treatment) classify as unassessable on this outcome.

2) Ongoing TB disease activity at week 96

Classified by disease activity research criteria (as in section 4.11.2, above), with some modifications based on WGS results and the presence of negative cultures. These are summarised in the text below and the table in section 4.13.3.

If only criteria A and B are met (not C or D), the classification of unsatisfactory outcome will be changed to unassessable if there is ≥ 1 negative sputum sample (observed) in the week 96 analysis window.

If only criterion C is met (not A, B or D), disease activity research criteria are not met. This special case - isolated positive sputum culture – will result in a classification of unassessable, unless EITHER WGS at week 96 shows a different strain from baseline OR WGS is inconclusive or not done and there is a second culture within the week 96 analysis window at which an observed negative value is obtained (not imputed): in both of these scenarios the isolated positive culture will be ignored.

3) Death during or prior to week 96 analysis visit window

All deaths during or prior to the close of the week 96 analysis window will be classified as an unsatisfactory outcome, with the following exception:

If the patient had a study visit at or after week 48; was not on treatment and had no evidence of ongoing TB activity when last seen (in study clinic or home visit; ongoing TB activity assessed by *disease activity clinical management criteria*); and the cause of death is known to be unrelated to TB or the drugs used to treat it (e.g. road traffic accident; cause of death will be taken to be unrelated to TB or drugs based on the SAE form indicating “unrelated” to both) the patient will be classified as unassessable on this outcome.

4.12.2 Classification of clinical outcome for patients not attending during the week 96 analysis window

If the patient did not attend in person during the week 96 window (and was not seen for a home visit, as described 4.4 and 4.12.1, above), information gathered at a telephone visit with the patient may be used to make an evaluation. If there is more than one patient telephone visit within the window follow the above rules for deciding which to take as the definitive visit. If there is no in-person patient visit or patient telephone visit within the week 96 analysis window (to week 108, see section 4.4), information gathered from friend/relative telephone visits within the week 96 analysis window will be considered in the final outcome.

Deaths will only be counted in the primary outcome analysis if the date of death occurs before the close of the week 96 analysis window. Any report of patient being alive at week 96 will only be considered if there is a report of a friend/relative seeing the patient in person on a date on or after the opening of the week 96 analysis window (i.e. from week 90, see section 4.4). Any report of a patient being on treatment or having symptoms will only be considered if the friend/relative reports seeing the patient in person on a date within the week 96 analysis window (considered to be within the first day of the first month to the last day of the last month covered by the analysis window, if the friend/relative can only estimate the date to the nearest month). If there is more than one friend/relative telephone visit, take the visit that reports the date of seeing the patient in person that is closest to the scheduled week 96 visit. If the dates are equidistant, or dates are not certain (but considered likely to be within months of the visit window), take the worst assessment on each of the TB symptoms and treatment questions to classify outcome. If no visit within window, the information will be regarded as missing.

Classification will be based on the following rules.

If the patient:

(a) Completed their last prescribed course of treatment (minimum 54 days of the boosted regimen, or 154 qualifying treatment days if on or switched to standard regimen; classified as in section 4.6.7) AND

(b) Had at least one study visit at (on the day of cessation) or after completing the course of treatment AND

(c) Did not meet the criteria for ongoing requirement for treatment or ongoing TB disease activity when last seen (assessed by disease activity clinical management criteria, section 4.11.1; evidence of CXR progression omitted unless CXR available at the visit when last seen); AND

(d) The last two sputum cultures obtained at study visits, taken at or following the completion of treatment ("at" defined as no more than 7 days prior to cessation of treatment) were negative (observed; or imputed from inability to produce sputum, with clinical disease recovery or by meeting criteria for sputum omission, see section 4.9.9) AND

(e) EITHER

The patient can be contacted at week 96 and confirms that they are not currently taking TB treatment (or have been prescribed TB treatment that they should be taking – information on intent to prescribe at last attended visit takes precedence over information provided by the patient), AND has no current pulmonary symptoms meeting the criteria of clinical evidence of TB (as listed in disease activity clinical management criteria), discounting symptoms clearly attributable to other causes)

OR

the patient cannot be contacted but a designated friend / relative verifies that they have personally seen the patient (within the week 96 visit analysis window) and that the patient was alive, neither reported taking TB treatment nor that they had symptoms meeting the criteria of clinical evidence of pulmonary TB (above) nor were observed to have symptoms meeting such criteria.

If (a) to (e) all true, classify as satisfactory clinical outcome.

If (a) to (d) all true, but there is no information available from patient or friend/relative to make a judgement on (e) in the absence of the patient having been contacted, classify as unassessable.

If (a) to (e) are not all true, classify as unsatisfactory.

Where additional information is available for a patient at week 96 (such as smears of CXR done at local clinics or the site clinic) but the patient is not seen in person (at clinic or home visit), the patient will be evaluated under the criteria in this section. The additional information will not alter the assessment of the outcome but may be reported as supporting information for the verdict derived from the above decision rules.

4.12.3 Blinded review of outcomes

In complex cases, the components of the primary outcome (clinical information, CXR evidence, microbiological evidence, including whole genome sequencing) at week 96 will be reviewed, blinded to treatment group, by reviewers with clinical, imaging, microbiological and sequencing expertise to ensure that outcomes have been correctly classified according to the algorithm.

4.13 Definition of unfavourable outcome (primary outcome for the regimen analysis)

This is the primary outcome of the regimen analysis and will also be used for biomarker analyses.

The algorithm for the definition of unfavourable outcome is shown in the table below (with the outcomes for the strategy analysis for comparison). The main differences between the primary outcome for regimen analysis (unfavourable outcome) and the strategy analysis (unsatisfactory outcome) are in events that occur before week 96:

- Variations in initial treatment from the strict randomised regimen specified in the protocol: these count as unassessable (and are censored in time-to-event analyses) or as unfavourable outcome in the regimen analysis, whereas in the strategy analysis they are ignored.
- Treatment failure/relapse and restarting treatment: these count as unfavourable outcome or unassessable (and are censored) in the regimen analysis, whereas in the strategy analysis they are ignored (unless the patient is on treatment or there is ongoing disease activity at week 96).

Phase 3 trials sometimes count switches due to toxicity as unfavourable outcome. This is reasonable when making an overall assessment of a drug prior to licensing, but it partially confounds the estimate of efficacy. In the regimen analysis of this trial, designed to provide a preliminary assessment of regimen (sterilising) efficacy, treatment switches are primarily counted as unassessable (and handled by censoring in time-to-event analyses) unless there is clear evidence of treatment failure preceding the switch. Toxicity is assessed separately by recording adverse events.

The following lists events that lead to a classification of unassessable (and censoring in the time-to-event outcome) or unfavourable outcome. If the patient does not meet the criteria for unassessable or unfavourable outcome then they are classified as favourable outcome. The patient is classified by the first event that occurs (in time, not in the order of the list below, although the listed is arranged by the approximate time at which the events would be expected to occur).

<u>Criteria for classification as unassessable outcome</u>

- [TMS arm only] Took less than a total of 54 qualifying treatment days of the (strictly-allocated) boosted regimen (reduced by 2 days from the protocol-mandated 56 days i.e. 8 weeks, to allow for prescribing or dispensing miscalculation, or patient-initiated early termination; qualifying treatment days defined in section 4.6.1). This is equivalent to the definition in the per-protocol population used for the strategy analysis (section 6.6).
- [SMS arm only] Took less than a total of 154 qualifying treatment days of the standard regimen (reduced by 14 days from the protocol-mandated 168 days i.e. 24 weeks to allow for prescribing or dispensing miscalculation of the standard regimen or patient-initiated early termination; includes any pre-baseline standard treatment; qualifying treatment days defined in section 4.6.1).
- [Both arms] Took less than 49 qualifying treatment days (of boosted treatment or standard treatment, as allocated by randomisation) in the first 56 days after randomisation of the trial (i.e. had more than 7 missed days; additional degrees of non-adherence – 3 days and 14 days – will be done as sensitivity analyses, section 11.6.2)
- [TMS arm only] Switch from boosted to standard treatment during initial treatment course (for toxicity or other reasons) <u>unless</u>:
    - The switch was temporary, for no more than 7 daily doses of standard treatment, and the boosted regimen was subsequently resumed (in which case ignore) OR

  - Treatment failure at switch: switch occurred after taking ≥ 54 qualifying days of the boosted regimen and the last sputum culture prior to switch was positive (taken after ≥ 54 qualifying days of boosted regimen; AND at or before the last day of the boosted regimen; AND and ≤5 days before switch; (single positive culture adequate; confirmation not required) AND WGS same strain, inconclusive or not done (if WGS shows different strain, classify as unassessable)
- [Both arms] A new TB drug is added to the assigned initial regimen, unless the addition is for no more than 7 days (in which case ignore) or the addition is a fluoroquinolone in the SMS arm for documented baseline drug resistance (also ignore).
- Treatment failure on stopping initial treatment *confirmed* (treatment failure analysis criteria met), but WGS shows different strain from baseline
- Relapse after initial treatment at or before week 96 (relapse analysis criteria met; *confirmed*); but WGS shows different strain from baseline
- Re-start of treatment for any reason before week 96 unless there was evidence of treatment failure or relapse prior to restarting treatment (in which case evaluate against the these criteria, above and below) .
- Ongoing TB disease activity at week 96 (*definitive or presumed*; by disease activity research criteria – i.e. criteria [D] or [C+A and/or B] are met); WGS shows only different strain(s) from baseline – none show same strain as baseline.
- Ongoing TB disease activity at week 96 (*possible*; by disease activity research criteria – i.e. criteria A and B only) with ≥ 1 negative sputum culture result (observed, not imputed) at week 96.
- Single positive sputum culture in week 96 analysis window", no other disease activity criteria (A, B or D) met and WGS same strain, inconclusive or not done (unless WGS shows different strain from baseline in which case ignore; OR ≥1 sputum culture is observed negative AND WGS is inconclusive or not done in which case ignore).
- Death before close of week 96 analysis window with last study visit after week 48, patient was well, not on treatment and had no evidence of ongoing TB activity at the last visit (*disease activity clinical management criteria*), and the cause of death is known to be unrelated to TB or TB drugs
- Withdrawal, lost to follow up or missed visit at week 96 and met the criteria [(a) to (d) true, and (e) did not have information available] listed above (4.12.2)

Criteria for classification as unfavourable outcome

- [TMS arm only] Switch from boosted to standard treatment during initial treatment course (for toxicity or other reasons) and:
  - Treatment failure at switch: switch occurred after taking ≥ 54 qualifying days of the boosted regimen and the last sputum culture prior to switch was positive (taken after ≥ 54 qualifying days of boosted regimen; AND at or before the last day of the boosted regimen; AND and ≤5 days before switch; (single positive culture adequate; confirmation not required) AND WGS same strain, inconclusive or not done)

- Treatment failure by treatment failure analysis criteria (*confirmed or unconfirmed*; WGS same strain as baseline, inconclusive or not done)
- Relapse at / before week 96 by relapse analysis criteria (*confirmed or unconfirmed*; WGS same strain as baseline, inconclusive or not done)
- Ongoing TB disease activity at week 96 (*definitive* or *presumed*, by *disease activity research criteria*; WGS same strain, inconclusive, or not done)
- Ongoing TB disease activity at week 96 (*possible* by disease activity research criteria; i.e. criteria A and B only; no negative sputum samples (observed) at week 96)
- Died before close of week 96 analysis window; <u>unless</u> last study visit was after week 48, patient was well, not on treatment and had no evidence of ongoing TB activity at the last visit (*disease activity clinical management criteria*), and the cause of death is known to be unrelated to TB or TB drugs (in which case classify as unassessable)
- Withdrawal, lost to follow up or missed visit at week 96 (<u>unless</u> met the disease activity research criteria 4.12.2, above, with (a) to (d) true and any response on [e], in which case will classify as unassessable or ignore).

<u>Criteria for classification as favourable outcome</u>

- None of the above criteria for unfavourable or unassessable are met

<u>Time to unfavourable outcome</u>
For the purposes of a secondary analysis of unfavourable outcome using a time-to-event approach, each participant will be allocated an outcome of either censored (for unassessable) or unfavourable at the time when the first outcome-defining event occurs (as shown in the table below). Participants who reach week 96 without experiencing one of the outcome-defining events (and that are therefore classified as “favourable” on the binary approach) will be allocated an outcome of censored at week 96.

Table: Summary of algorithm for classification of unfavourable and unsatisfactory Outcomes

**EVENT**	**Unfavourable Outcome** Regimen analysis (time of outcome)	**Unsatisfactory Outcome** Strategy analysis
Initial treatment insufficient / non-adherent		
TMS arms only:<br>Took less than 54 qualifying days of boosted regimen in total*	Unassessable (at last qualifying day of boosted regimen)	Ignore
SMS arm only:<br>Took less than 154 qualifying days of standard regimen in total**	Unassessable (at last qualifying day of standard regimen)	Ignore
TMS arms and SMS arm:<br>Took less than 49 qualifying days of boosted or standard regimen (as allocated by randomisation) during the first 56 days of the trial***	Unassessable (at 7th missed dose)	Ignore
Initial treatment switch		
TMS arms only:<br>Switched to standard regimen<br><br>Exception 1:<br>≤ 7 days of standard treatment followed by reintroduction of boosted regimen<br><br>Exception 2: Treatment failure at switch<br>Took ≥ 54 qualifying days of boosted regimen; latest culture positive (taken at ≥ 54 qualifying days of boosted regimen; AND at or before the last day of the boosted regimen; AND at ≤5 days before switch (single positive culture adequate; confirmation not required); AND WGS same strain, inconclusive or not done (if WGS shows different strain this exception doesn't apply, so classified as unassessable)	Unassessable (at last qualifying day of boosted regimen)<br><br>Exception 1: Ignore (do not censor)<br><br>Exception 2: Unfavourable (at switch) ****	Ignore<br><br>Ignore<br><br>Ignore<br><br>Ignore
TMS arms and SMS arm:<br>Added new TB drugs to the assigned initial regimen<br><br>Exception 1:<br>Temporary addition (≤ 7 days of new drug)<br><br>Exception 2:<br>Addition of fluoroquinolone in the SMS arm for documented baseline drug resistance.	Unassessable (at start of new drug)<br><br>Exception 1: Ignore (do not censor)<br><br>Exception 2: Ignore (do not censor)	Ignore<br><br>Ignore<br><br>Ignore

Treatment failure on stopping initial treatment		
Treatment failure *confirmed* or *unconfirmed* by *Treatment failure analysis criteria*<br>WGS same strain as baseline, inconclusive or not done<br><br>Exception 1:<br>Treatment failure *confirmed*<br>WGS shows different strain from baseline<br><br>Exception 2:<br>Treatment failure *unconfirmed*<br>WGS shows different strain from baseline	Unfavourable (at end-of-treatment culture) ****<br><br>Exception 1:<br>Unassessable (at end-of-treatment culture)<br><br>Exception 2: Ignore (do not censor)	Ignore
Relapse after initial treatment		
Relapse *confirmed* or *unconfirmed* by *relapse analysis criteria*<br>WGS same strain as baseline, inconclusive or not done<br><br>Exception 1:<br>Relapse *confirmed*<br>WGS shows different strain from baseline<br><br>Exception 2:<br>Relapse *unconfirmed*<br>WGS shows different strain from baseline	Unfavourable (at first positive culture in relapse episode) ****<br><br>Exception 1:<br>Unassessable (at first positive culture in relapse episode)<br><br>Exception 2: Ignore (do not censor)	Ignore
Re-treatment		
Re-started treatment before week 96 (any reason apart from treatment failure/relapse/reinfection meeting criteria above)	Unassessable (at restart of treatment)	Ignore
Ongoing requirement for TB treatment at week 96		
Started treatment at or after week 72 but stopped treatment before week 96<br><br>Exception:<br>Did not meet *disease activity clinical management criteria* when started treatment	Ignore [covered by criteria above]<br><br>Exception: Ignore [covered by criteria above]	Unsatisfactory<br><br>Exception: Unassessable
On treatment at week 96	Ignore [covered by criteria above]	Unsatisfactory
Ongoing TB disease activity at week 96		
Assessed by *disease activity research criteria* at week 96: *Definitive* (D) or *Presumed* (C + A and/or B) disease activity [WGS same strain, inconclusive or not done]<br><br>Exception:<br>WGS shows different strain from baseline	Unfavourable (at week 96)<br><br>Exception:<br>Unassessable (at week 96)	Unsatisfactory<br><br>Exception: Unassessable
Assessed by *disease activity research criteria* at week 96: *Possible* (A+B only) disease activity	Unfavourbale (at week 96)*****	Unsatisfactory

Exception:<br>At least one sputum culture (observed) negative at week 96	Exception:<br>Unassessable	Exception<br>Unassessable
Single positive culture at week 96		
Assessed by *disease activity research criteria* at week 96:<br>Criterion C met (A, B and D all not met)<br>WGS same strain; or WGS inconclusive or not done<br><br>Exception 1:<br>WGS shows different strain from baseline<br><br>Exception 2:<br>WGS inconclusive or not done <u>and</u> at least one other sputum culture (observed) negative at week 96	Unassessable (at week 96)******<br><br><br>Exception 1: Ignore (do not censor)<br><br>Exception 2: Ignore (do not censor)	Unassessable<br><br><br>Exception 1: Ignore<br><br>Exception 2: Ignore
Death at or prior to week 96		
Death before close of week 96 analysis window<br><br><br>Exception<br>Last study visit was after week 48<br>AND<br>well, not on treatment, no evidence ongoing TB activity at last study visit [assessed by *disease activity clinical management criteria*]<br>AND<br>cause of death known to be unrelated to TB or TB drugs	Unfavourable (at day of death)<br><br><br>Exception<br>Unassessable (at day of death)	Unsatisfactory<br><br><br>Exception<br>Unassessable
Did not attend week 96 visit (and not known to have died)		
Withdrawal, lost to follow up, missed week 96 visit<br><br>Exception 1:<br>(a) Completed last prescribed course of treatment (minimum 54 days of boosted regimen, or 154 qualifying treatment days if on or switched to standard regimen)<br>AND<br>(b) Had at least 1 study visit at or after completing last course of treatment<br>AND<br>(c) Did not meet the criteria for ongoing requirement for treatment [addressed by (a), above] or ongoing TB disease activity when last seen [assessed by *disease activity clinical management criteria*; evidence of CXR progression omitted unless CXR available at the visit when last seen]<br>AND<br>(d) Last 2 visits (at or following end of treatment) had negative sputum cultures (observed or imputed based on clinical recovery)<br>AND<br>(e) EITHER patient can be contacted at week 96 and confirms not on treatment and no TB symptoms OR friend/relative can be contacted at week 96 confirming	Unfavourable (at last study visit)<br><br>Exception 1: Ignore (do not censor)	Unsatisfactory<br><br>Exception 1: Ignore

seen alive in previous 2 months, did not report TB treatment nor symptoms (or observed to have symptoms)<br>(a) to (e) all true<br>Exception 2:<br>(a) to (d) all true, no information available to judge (e)	Exception 2:<br>Unassessable<br>(at last study visit)	Exception 2:<br>Unassessable

Notes

* Censoring for more than 56 days of boosted regimen will be explored in sensitivity analyses of regimen efficacy (section 11.6.2)

**Duration of standard regimen includes period of standard treatment taken before randomisation

*** Different durations of non-adherence (>3 days missed; > 14 days missed) will be explored in sensitivity analyses of regimen efficacy (section 11.6.2)

**** Classification of treatment failure at switch, treatment failure at end of treatment *unconfirmed* and relapse unconfirmed – all with WGS same strain, inconclusive or not done – as unassessable will be explore in a sensitivity analysis of regimen efficacy (section 11.6.2)

*****Classification of possible disease activity as unassessable will be explored in a sensitivity analysis of regimen efficacy (section 11.6.2)

******Classification of isolated culture positive as unfavourable will be explored in a sensitivity analysis of regimen efficacy (section 11.6.2)

4.14 Patient-centred variables

4.14.1 Acceptability of the strategy

Variables for assessment of acceptability are derived from the patient acceptability questionnaire that is administered at weeks 48 and 96. This comprises separate assessments of difficulty, anxiety, motivation and overall perception.

<u>Evaluation of difficulty and anxiety domains</u>

A response will be designated as complete on the difficulty domain and anxiety domain if all questions for the domain are answered. If neither the week 48 nor the week 96 questionnaire has a complete response for that domain, the patient will be designated as not evaluable for that domain.

For patients with an evaluable response on each of the difficulty and anxiety domains, a binary classification of acceptability will be created. The intervention will be designated as being acceptable to the patient if, for all the complete responses on that domain (i.e. obtained at week

48, week 96 or both), all questions are answered "none", "a little" or "some" (i.e. no question answered as "a lot").

For patients with an evaluable response, a summary score for difficulty and anxiety will be obtained by allocating a score of 0,1,2,3 to each of the responses on each question in the domain, summing the scores to get a total score (averaged if evaluable complete responses are obtained at both week 48 and 96 on that domain), and then converting the score (x10/8 for difficulty; x10/12 for anxiety) to a scale out of 10.

Evaluation of motivation

A patient will be evaluable for motivation if there is a response on this question at week 48 (week 96 will not be considered). There is no binary classification of acceptability for this domain. For patients with an evaluable response, a motivation summary score will be calculated as described for anxiety and difficulty above, based on the week 48 questionnaire only.

Evaluation of overall perception

This is obtained from the response to the question on which treatment option the patient would recommend to a friend. The response at week 96 will be used. Where there is no response available at week 96 the response at week 48 will be used. Answers to this question will be used directly in the analysis without further derivation of variables.

4.14.2 Time off work or study due to illness or treatment

The sum of all reported days of work or school missed due to illness from randomisation up to week 96.

4.14.3 Quality of life (MOS-HIV)

Patient responses on the MOS-HIV quality of life questionnaire will be converted to scores on 11 subscales (see Shim et al. 2018, Health and Quality of Life Outcomes 2018; 16:92 for a list of items under each subscale), ranging from 0 to 100, with higher scores indicating better health. The scores are then synthesised into a physical health summary score (PHS) and a mental health summary score (MHS) using factor score coefficients (Table 2 in Revicki et al, Reliability and validity of physical and mental health summary scores from the Medical Outcomes Study HIV Health Survey. Medical Care 1998 Feb;36(2):126-37).

4.14.4 Health status (EQ-5D)

The scores on the 5 levels of the EQ5D will be converted to an overall index score using EQ-5D-5L country-specific value sets. The value set for Indonesia is available[21] and valuations of EQ-5D-5L have been completed for other study countries. [Fredrick DP,et al. The Indonesian EQ-5D-5L value set. PharmacoEconomics 2017; 35:1153-1165]. In case a value set is not available at the time of data analysis, a value set will be used that is obtained from a population that most closely approximates that country.

4.14.5 Respiratory disability (MRC dyspnoea scale)

Respiratory disability will be defined as a score of ≥ Grade 3 on the MRC dyspnoea scale, based on patient self-report of breathlessness.

An alternative assessment of respiratory disability will be based on spirometry measurement (see section 4.6.3)

4.15 Definitions related to transmission risk

Household transmission risk scores

Definitions

Participant with transmission risk is defined as:

- confirmed relapse (section 4.9.11)
- at least one smear at least grade 1+ during the transmission risk period (defined below).

Transmission risk period (for participant with transmission risk, defined as above) defined as:

- Start: date of the first positive smear (≥ grade 1+) obtained after the start of the relapse episode
- End: start of re-treatment or date of last positive smear (≥ grade 1+) before end of trial follow-up, if treatment is not re-started.

Exposed household contact defined as person living in the same household as participant at any time during the transmission risk period (defined as above), obtained from the household contact form (F4) that was completed at the time of suspected relapse (or imputed from the previous and/or subsequent household contact forms, if none completed at the time of suspected relapse).

New exposed housold contact: exposed household contact (defined as above), not living in same household as participant at time of trial enrollment (contacts at trial entry deemed to have been previously exposed with limited additional risk of transmission)

Calculation of scores

Household contact transmission risk score (new contacts)*: the sum of new exposed household contacts

Household contact transmission risk score (all contacts): the sum of all exposed household contacts

Adjusted household contact transmission risk score (new contacts)*: the sum of the adjusted risk for each new household contact. Risk adjusted as follows (adjustments made are approximate, based on risks identified for contact tracing, generally based on low-quality evidence):

Base risk score is 1 per household contact

- Smear status of participant with transmission risk (highest smear grade during transmission risk period, defined as above): smear 1+, no adjustment; smear 2+, multiple by 2; smear 3+, multiply by 3
- Presence of cough in participant with transmission risk (any report of cough on relapse assessment form or on visit symptom review during transmission risk period): no cough, multiply by 0.5; cough: no adjustment
- Degree of contact: sleep in same room, multiply risk by 2; does not sleep in same room but spends ⩾ 4 hours a day in the same room, no adjustment; spends less than 4 hours per day in same room, multiply by 0.5

Adjusted household contact transmission risk score (all contacts): as above, but scored for all contacts

If trial participant has a second confirmed relapse after completion of 6 months' treatment for the first relapse episode, the exposure associated with the second episode will also be estimated as above. Where the contact has exposure they will be counted only once in the (unadjusted) score; for the adjusted score they will be counted according to the higher of the risk estimates calculated for the two episodes.

* Main scores used for evaluating strategy are the household contact transmission risk score (new contacts), unadjusted and adjusted

<u>Overall community transmission risk score</u>

Definitions
Smear positive transmission risk period : defined as above
Maximum smear grade: highest smear grade obtained during the transmission risk period.

Calculation of score

Overall community transmission risk score: obtained as the product of the smear positive transmission risk period (in months) and the smear grade

4.16 Definitions related to health economics evaluation

The definitions and sources of the healthcare resource utilisation parameters and costs will be set out in a separate health economics analysis plan.

4.17 Derivation of socioeconomic status

Two of the main components of overall socioeconomic status assessment will be used as independent indicators of socioeconomic status:

- Years of education: categorised as none /primary (0-7), secondary (8-13), tertiary (≥14)
- Employment: categorized as working, studying, not working

Education and employment will be presented at baseline to characterise the patient population for assessment of comparability of randomised groups and for assessing external generalisability. Education is included as a potential subgroup for analyses for the primary strategy outcome. Education and employment will be included as potential predictive factors for outcomes in the strategy implementation analysis.

Data on education and employment and other parameters collected in the socioeconomic status battery will be transformed into both continuous and binary classifications of socioeconomic status; overall living standards will be categorised into high, medium and low.

5 SAMPLE SIZE CALCULATION

We estimate that the proportion of patients meeting the primary endpoint of unsatisfactory outcome in the control arm will be 10%.[13] The sample size estimate is based on a non-inferiority margin of 12% of absolute difference in proportions, meaning no greater than 22% of patients with unsatisfactory outcome in a boosted arm. The justification for the margin of 12% for evaluating new regimens is given in the Section 10.3 of the trial protocol (page 76).

We also estimate that 10% will be classified as unassessable (and therefore will be excluded from the primary analysis) based on estimates from the REMoxTB, RIFAQUIN and OFLOTUB trials (although these trials used a broader definition of unassessable).

We assume that:

(i) 2 boosted arms will be included in the final analysis

(ii) the proportion of patients with an unsatisfactory outcome in each of these arms will also be 10% (i.e. the same as the standard-of-care arm).

With these estimates and assumptions, with 87% power to conclude non-inferiority at a 1.25% one-sided significance level (reduced from the standard 2.5% to allow for the two final comparisons) the required sample size for the control arm and each of the boosted arms at the final analysis will be 180 patients per arm. With four intervention arms and one control arm, the maximum sample size will be 900.

6 ANALYSIS POPULATIONS

6.1 Intention-To-Treat (ITT) Population

The ITT population is defined as all randomised patients with the exclusion of:

- Patients considered to be randomised in error i.e. patient was ineligible for study participation based on the eligibility criteria; the eligibility error was identified on the day of randomisation and before the patient received any TB drugs (standard or non-standard boosted-regimen drugs); and with no trial follow-up arranged after randomisation.

The ITT population will be used for the analysis of safety of the TRUNCATE-TB management strategy, including reported adverse events as well as secondary safety outcomes of respiratory disability, acquired drug resistance and community transmission risk.

6.2 Intention-To-Treat Exposed (ITT Exposed) Population

The ITT exposed population is defined as the ITT population, with the additional exclusion of:

- Patients who did not receive at least one dose of the randomly-allocated study regimen.

The ITT exposed population will be used for the analysis of regimen safety, including reported adverse events as well as secondary safety outcomes of respiratory disability and acquired drug resistance.

6.3 Modified Intention-To-Treat (mITT) Population

The mITT population is defined as the ITT population, with the additional exclusion of:

- Patients with isolates taken at baseline (up to week 2, if no isolate at baseline) that show resistance (confirmed or unconfirmed) to rifampicin (see section 4.9.13)

The mITT population will be used for the main analyses of all primary and secondary efficacy ouctomes for the evaluation of the TRUNCATE-TB management strategy.

6.4 Modified Intention-To-Treat Exposed (mITT Exposed) Population

The mITT exposed population is defined as the mITT population, with the additional exclusion of:

- Patients who did not receive at least one dose of the randomly-allocated study regimen.

The mITT exposed population will be used for the primary analysis of regimen efficacy (unfavourable outcome at week 96); secondary analysis of regimen efficacy (time to unfavourable outcome); the primary analysis of regimen bactericidal activity (time to culture

conversion); secondary analyses of regimen bactericidal activity (culture status at week 8 and change in Time-to-Positivity); and analyses of biomarkers relating to unfavourable outcome.

6.5 Modified Intention-To-Treat, other populations

mITT assessable population

This is defined as the mITT population, with the additional exclusion of:

- Patients allocated an outcome of "non-assessable" in the algorithm for determining unsatisfactory clinical outcome at week 96.

This population will be used for a sensitivity analysis of the primary outcome in the strategy analysis.

mITT exposed assessable population

This is defined as the mITT exposed population, with the additional exclusion of:

- Patients allocated an outcome of "non-assessable" in the algorithm for determining unfavourable outcome at week 96

This population will be used for a sensitivity analysis of the primary outcome for the evaluation of regimen efficacy (unfavourable outcome at week 96) and for biomarker analyses or predictors of unfavourable outcome.

mITT exposed culture positive population

This is defined as the mITT exposed population, with the additional exclusion of:

- Patients who did not have at least one positive MGIT culture at any time (including prior to baseline) up to week 2.

This population will be used for a sensitivity analysis of the primary outcome for the evaluation of regimen efficacy (unfavourable outcome at week 96); and a sensitivity analysis of the primary outcome of the evaluation of regimen bactericidal activity (time to culture conversion)

mITT exposed, fully susceptible population

This is defined as the mITT exposed population, with the additional exclusion of:

- Patients with known drug resistance detected at baseline or anytime up to week 2 (see definitions in section 4.9.13) to any drug allocated by randomization. If no evidence to the contrary, assume full susceptibility at baseline.

This population will be used for a sensitivity analysis of the primary outcome for the evaluation of regimen efficacy (unfavourable outcome at week 96)

mITT exposed, culture positive, fully susceptible population

This combines the above two populations and will be used for a single sensitivity analysis of the main outcomes (time to culture conversion and change in time to positivity) of regimen bactericidal activity.

6.6 Per-Protocol Population

This is intended, broadly, to capture the population of patients who took the boosted regimen (or the initial standard regimen course if randomised to the SMS arm) for the minimum duration recommended in the protocol; with adequate adherence; and who did not add or substitute other TB drugs.

For the regimen analysis, the algorithm for assessing outcome on relapse-free cure already selects out (and classifies as unassessable, or unfavourable) those participants that had inadequate duration of treatment, or who added drugs to the regimen, or who were non-adherent during the initial course of treatment (section 4.13), obviating the need for a per-protocol population.

The per-protocol population is defined for use in the strategy analysis only, as a sensitivity analysis of the primary outcome at week 96. The per-protocol population is derived from the mITT population with the additional exclusion of:

- Patients randomised to a TMS arm who took less than a total of 54 qualifying treatment days of the (strictly-allocated) boosted regimen (reduced by 2 days from the protocol-mandated 56, 8 weeks, to allow for prescribing or dispensing miscalculation, or patient-initiated early termination; qualifying treatment days defined in section 4.6.1).

- Patients randomised to the SMS arm who took less than a total of 154 qualifying treatment days of the standard regimen (reduced by 14 days from the protocol-mandated 168, 24 weeks, to allow for prescribing or dispensing miscalculation of the standard regimen or patient-initiated early termination; includes any pre-baseline standard treatment; qualifying treatment days defined in section 4.6.1).

- Patients randomised to TMS or SMS arms who took less than 49 qualifying treatment days in first 56 days after randomisation (88% adherence).

- Patients randomised to TMS arms that switched to the standard treatment regimen after taking ≥ 54 days of the boosted regimen; unless temporary switch for ≤ 7 days and reintroduced boosted regimen; or unless the switch was for the purposes of treatment extension for persistent clinical disease assessed at week 12 that met criteria for treatment extension (TB symptoms and positive smear).

- Patients randomised to a TMS arm who added any anti-TB drugs to the boosted regimen during the initial treatment course.

- Patients randomised to the SMS arm who added any anti-TB drugs to the standard regimen in the initial treatment course with the exception of addition of a fluoroquinolone (for suspected or documented drug resistance).

Summary of analysis populations

Outcome	Outcome hierarchy	Analysis population
**Strategy efficacy analysis**		
Unsatisfactory outcome, W96	Primary, main	mITT
	Primary, sensitivity	mITT, assessable
	Primary, sensitivity	Per-protocol
	Primary, subgroup analysis	mITT
Acceptability, treatment days, adherence, default, time off work, quality of life	Secondary	mITT
**Strategy safety analysis**		
Adverse events, death, respiratory disability, acquired drug resistance, community transmission risk	Secondary	ITT
**Strategy implementation**		
Unsatisfactory outcome, W96	Other	mITT
**Strategy, health economics**		
Cost effectiveness	Other	mITT
**Regimen sterilising efficacy**		
Unfavourable outcome, W96	Primary, main	mITT, exposed
	Primary, sensitivity	mITT, exposed, assessable
	Primary, sensitivity	mITT, exposed, culture positive
	Primary, sensitivity	mITT, exposed, fully susceptible
	Primary, subgroup analysis	mITT, exposed
Time to unfavourable outcome	Secondary	mITT, exposed
**Regimen bactericidal activity**		
Time to culture conversion, culture conversion status at week 8, change in TTP	Secondary, main	mITT, exposed
	Secondary, sensitivity	mITT, exposed, culture positive, fully susceptible
**Regimen safety**		
Adverse events, death, acquired drug resistance	Secondary	ITT, exposed

7 Statistical analysis general principles

7.1 Sequence of analyses

TRUNCATE-TB addresses a unique strategic approach to TB treatment. In addition to the innovation in the design and definition of novel endpoints, the analyses also differ from those used in standard TB trials comparing efficacy of drug regimens. The experimental paradigm provides opportunities for numerous secondary analyses that have the potential to make a broad contribution across the field of TB research. The analyses are described in some detail below but may be developed further in supplementary analysis plans where needed.

For ease of reference, this sequence of analyses below is arranged in the order in which they are intended to be conducted and published. Additional analyses, such as pharmacokinetics analyses and biomarker analyses, will be done in parallel with the main analyses. The analyses are treated as discrete, with definition of trial populations, treatment and other key parameters described for each to maximise clarity, although there may be overlap.

The analyses are organised as follows:

- Efficacy and safety of the management strategy
- Implementation and performance of the management strategy
- Health economics of the management strategy
- Efficacy and safety of the boosted regimens
- Bactericidal activity of the boosted regimens
- Routine clinical biomarkers for predicting outcomes
- Novel biomarkers for predicting outcomes
- Pharmacokinetics
- Pharmacokinetic-pharmacodynamic relationships
- Others (including drug resistance development)

Plans for analyses of biomarkers and pharmacokinetics will be developed as separate appendices to this plan.

7.2 General approach to analysis

Continuous variables will be summarised using descriptive statistics, i.e., either mean and standard deviation; or median, range (minimum and maximum), and interquartile range (IQR – presented as lower quartile and upper quartile).

Categorical variables will be summarised by frequencies and proportions. Unless otherwise indicated, proportions in tables will be column percentages, using number of participants in the analysis group, i.e., the column, as the denominator. Percentages will be rounded to one decimal place and, therefore may not always add up to 100.

All p-values are two-sided. A p-value <0.05 is considered statistically significant unless there are specified adjustment for multiplicity. Except for specified primary and secondary outcomes, confidence intervals rather than P values will be reported when multiple testing has been performed.

All confidence intervals (CI) are at 95% level unless otherwise indicated.

All statistical analyses will be carried out using SAS version 9.4 or higher (SAS Institute, North Carolina, USA), unless otherwise indicated.

8 Analysis of the efficacy and safety of the management strategy

8.1 General

The primary outcome, unsatisfactory clinical outcome, is a composite outcome assessed at week 96 using decision rules described in section 4.12. The rate of unsatisfactory outcome in the TMS arms will be assessed for non-inferiority against the rate in the SMS arm. In addition, the various advantages and disadvantages of the TMS will be explored in analyses of secondary outcomes.

It is important to note that the decision on the appropriateness and utility of the TMS will depend as much on consideration of key secondary outcomes as it will on meeting a pre-specified non-inferiority threshold for the primary outcome.

Data analysis of the primary outcome and secondary outcomes will control for site and baseline risk stratum (lower, intermediate, higher). Sites recruiting fewer than 30 participants will be pooled geographically with the next smallest site(s) within a country or, if fewer than a total of 30 participants within a country, with the next smallest site within another country. Controlling for site may be omitted if data anlaysis results become unstable with adjustment for site.

A Bonferroni correction for multiplicity will be applied in the pairwise-comparisons of the primary outcome between the boosted regimens that continue through to the final analysis and the control arm. Apart from this correction on the primary outcome, no formal adjustment for multiple testing will be made for comparisons on secondary or other outcomes. However, all significance tests will be interpreted in the context of the number of comparisons performed. The study report will include a statement of the number of signficance tests performed for subgroup analyses of the primary outcome, for secondary outcomes and for other outcomes along with a statement of the number of statistically-significant results that would be expected to occur by chance alone, following the approach recommended by Wang et al (NEJM 2007).

Summary statistics for each outcome variable will be presented by treatment arm, for all treatment arms. However, unless specified otherwise below, only the *complete* TMS arms will be compared formally (with statistical testing) with the SMS arm.

For secondary outcomes that are expressed as proportions, Cochran Mantel Haenszel $\chi 2$ test controlling for study site and baseline risk stratum will be used to compare arms. Fisher’s exact test will be used for comparison of proportions with few events (defined as more than 20% of the expected counts are less than 5 in the contingency table). Continuous data will be compared between groups by t-tests or ANOVA, or non-parametric tests, as appropriate. Relative risks will be adjusted for study site and baseline risk stratum; they will be provided with a 95% CI. P vaues of less than 0.05 will be taken to indicate a significant difference.

8.2 Analysis populations and treatment arms

8.2.1 Analysis populations

Analysis populations are described in Section 6.

Primary outcome
The primary analysis of the efficacy of the management strategy will be done on the mITT population.

Although it is traditional for equal importance to be given to analyses based on mITT and PP populations in non-inferiority trials, in a strategy trial such as this, where treatment switches and re-treatment form an essential part of the strategy, a PP analysis is of less relevance. The PP population will therefore be used only in a sensitivity analysis of the primary outcome.

The mITT assessable population will also be used for a sensitivity analysis of the primary outcome.

Secondary outcomes

Analyses of the secondary outcomes that relate to efficacy or patient experience will be done mainly on the mITT population.

Analyses of secondary safety outcomes that relate to safety (adverse events, respiratory disability) will be done using the ITT population.

8.2.2 Analysis treatment arms

Primary outcome

The primary outcome will be analysed on all the *complete* arms (defined in section 4.5), with individual pairwise comparisons with the SMS arm.

Exploratory analyses will be done with the *complete* arms combined in a single analysis set for comparison with the SMS arm.

Estimates of the primary outcome will also be given for the *partial* TMS arms but no inferences on non-inferiority will be made.

Secondary outcomes relating to efficacy or patient experience
These will be analysed on complete arms only.

Secondary outcomes relating to safety

Safety outcomes will be analysed primarily on the *complete* arms (defined in section 4.5), with individual pairwise comparisons with the SMS arm.

Safety outcomes will be reported separately for individual *partial* arms as descriptive data, but no statistical comparisons will be made with the safety in the SMS arm.

8.3 Enrollment and follow-up

The numbers of patients screened, randomized (with reasons for not randomising), treated, withdrawn, lost-to-follow-up, and analysed will be summarised in a CONSORT flow chart.

The number of participants randomised in error and immediately withdrawn and other eligibility violations will be summarized in a line listing.

Visit attendance will be summarised for the trial population in the following categories:
1) All 16 scheduled in person follow-up visits (baseline excluded) from week 1 to week 96 (inclusive), counting only 1 visit per scheduled visit time (n/N, % of total expected visits for all trial patients)
2) Week 96 visit only (counting only 1 visit, even if several within window) (n/N, %)
3) All 11 additional scheduled telephone visits
4) All 27 scheduled follow-up visits (in-person and telephone)

For each of these visit categories, the number of visits will be presented in the following sub-categories:
- Visits attended in person (including home visits)*
- Visits attended by telephone
- Visits attended, either in person or by telephone

Where there are both in-person and telephone visits within a visit window, the visit will be counted as an in-person visit.

* Home visit defined as site staff physically seeing patient at home or another location outside the clinic to perform in person a minimum of symptom assessment, medication assessment and relapse assessment. Home visits will be included in total of visits attended, but the number of total visits that comprise home visits will also be specified.

For all 4 categories, these will be presented by:
- Randomised arm
- Total population

For category (1) [all scheduled visits from week 1 to week 96], the total visits (and subcategory of in person and telephone) will also be presented by:
- Study site

8.4 Demographics and Baseline Characteristics

Demographics and baseline characteristics will be summarised for the mITT population with columns for each randomised arm (complete and partial) and a total column.

Definitions and derivation of baseline characteristics given in Chapter 4.

Descriptive statistics will be provided for the following baseline characteristics:

<u>Demographic and medical history</u>

- Sex
- Age: median (IQR); range; categories [< 35, 35-50, >50]
- Study country (determined by the site at which participant was enrolled)
- Socioeconomic status (employment): categories [working (total, and 3 subcategories: full time, part time, occasionally); full-time student; not working (total and 3 subcategories: ill health, lack of employment, retirement)]
- Socioeconomic status (education), total years of education: categories [0-7, 8-13, ≥ 14]
- Smoking status: categories [current, past, never]
- Alcohol consumption, units per week: categories [none, 1-7, 8-14, > 14]
- History of Diabetes (at study entry or diagnosed within 1 week of study entry)
- History of Chronic Obstructive Pulmonary Disease (at study entry)
- HIV co-infection

<u>TB disease</u>

- TB symptoms at baseline, present during previous 7 days: categories [fever, night sweats, cough, haemoptysis, pleuritic chest pain, progressive weight loss; any symptom, no symptom]
- MRC Dyspnoea Scale grade at baseline: median (IQR); categories [1-5]
- Body weight at baseline: median (IQR); range
- Body mass index: median (IQR); range; categories [< 17, 17 to <18.5, 18.5-25, > 25]

<u>Previous TB treatment</u>

- Pre-baseline treatment, days: median, IQR, range (defined in section 4.6.4)

<u>General Investigations</u>

- Baseline creatinine clearance (calculated using Cockroft-Gault): mean (SD), range, categories [<60 ml/min; ≥60 ml/min]
- Baseline haemoglobin: mean (SD), range, categories [< 9g/dl; ≥9g/dl]
- Baseline QTcF (from screening ECG, or baseline ECG if no ECG at screening): mean (SD)

<u>Imaging</u>

Baseline CXR (definition in section 4.2)

- Days before baseline: median, IQR, range
- Bilateral disease (Y/N)
- Pleural effusion: categories [none, blunting, <25%, 25-50%,>50%]
- Proportion of total lung affected (%): median, IQR, range; categories [<20%, 20-50%, >50%]
- Cavitation: categories [absent; present largest cavity ≤ 4cm; present largest cavity > 4cm]
- Size of largest cavity: median (in those with cavities), IQR; categories [0, 1, 2, 3, 4, > 4]

Expanded baseline CXR (definition in section 4.2)
Parameters as above (except omit days before baseline)

<u>Microbiology test results</u>

Baseline sputum smear (definition in section 4.2)

- Sputum smear grade: categories [no test; negative, scanty, 1+, 2+, 3+]

Expanded baseline sputum smear (definition in section 4.2)

- Sputum smear grade: categories [no test; negative, scanty, 1+, 2+, 3+]

Baseline GeneXpert (definition in section 4.2)

- Test type (No test, Standard vs Ultra); n, %
- Days before baseline: median, IQR, range
- CT: median (IQR), categories [high, medium, low, very low] (section 4.9.6)

Expanded baseline GeneXpert (definition in section 4.2)

- CT: median (IQR), categories [high, medium, low, very low] (section 4.9.6)

Baseline MGIT (definitions in section 4.2)

- MGIT culture status: categories [negative, TB positive, TB positive and contaminated, contaminated only, other positive/uncertain, no result available, no sample obtained]
- MGIT culture positive status (any culture to week 2): categories [TB positive; TB positive and contaminated]
- Time to positivity (TTP): median (IQR); categories [<7 days, 7-14 days, > 14 days]

Baseline drug susceptibility test (DST)

- DST results from baseline MGIT culture: n (%)
- DST results from basaeline to week 2: n (%)
- Rifampicin resistance: n / N tested (%)
- Isoniazid resistance: n /N tested (%)
- Pyrazinamide resistance: n /N tested (%)
- Ethambutol resistance: n/N tested (%)
- Levofloxacin resistance: n/N tested (%)

<u>Aggregate relapse risk category</u>

- Risk stratum at baseline (categorized as lower, intermediate, higher as defined in the protocol) [based on observed smear, CXR result recorded at baseline]: n, %
- Very low risk stratum (section 4.10)
- Mismatch with randomised risk stratum: n, %
- Expanded risk stratum at baseline (defined in section 4.2 and 4.10): n, %

<u>Health status (EQ5D) and Quality of Life</u>

- Health status score (EQ-5D), average of 5 questions: mean (SD), range
- Health status score (EQ-5D), distribution by category: number (%) with score ≥ 3 (at least moderate impaired health status) in each category (Walking, washing/dressing, usual activities, pain/discomfort, anxiety/depression)

8.5 Exposure and Adherence to Study Medications

Participants who do not receive allocated treatment will be reported in the CONSORT diagram.

8.5.1 Trial-wide regimen changes

If any systematic dose changes were made during the trial, the numbers of patients and proportion will be reported in each affected trial arm:

- Started at original dose and stopped boosted treatment without changing to modified dose
- Started original dose and changed to modified dose before completion of boosted treatment
- Started modified dose

For the second group, the median, range, IQR for number of days of treatment taken at the original dose prior to switch will be reported (section 4.6.2).

8.5.2 Number of treatment courses

See definitions in section 4.6.3

The following will be presented for each of the TMS arms (*complete* and *partial* arms) and for the SMS arm:

- Number of TB treatment courses given (categorical 1, 2, ≥ 3): n (patients), %
- Time to second treatment course (from randomisation, days): median, range, IQR
- Time to second treatment course (from end day of initial course, days): median, range, IQR
- Time to third treatment course (from randomisation, days): median, range, IQR
- Time to third treatment course (from end of treatment of previous course, days): median, range, IQR

8.5.3 Treatment course duration and daily doses taken

Definitions in 4.6.4 and 4.6.5.

The following will be presented for each of the SMS and TMS (complete and partial) arms:

1) Pre-baseline treatment
   - Duration, days: median, range, IQR
   - Daily doses taken (qualifying), days: median, range, IQR
2) Initial treatment course (strict randomised regimen)
   - Duration, days: median, range, IQR
   - Daily doses taken (qualifying), days: median, range, IQR
3) Initial treatment course (any regimen)
   - Duration, days: median, range, IQR
   - Daily doses taken (qualifying), days: median, range, IQR
4) Total initial treatment course (pre-baseline plus post-baseline, any regimen)
   - Duration, days: median, range, IQR
   - Daily doses taken (qualifying), days: median, range, IQR
5) Total re-treatment courses (any regimen)
   - Duration, days: median, range, IQR
   - Daily doses taken (qualifying), days: median, range, IQR
6) Total treatment courses (post-baseline; any regimen) – the sum of all treatment courses given post-baseline to the week 96 visit:
   - Duration, days: median, range, IQR *
   - Daily doses taken (qualifying), days: median, range, IQR
   - Daily doses taken (qualifying; and missed, partial), days: median, range, IQR *

*Total treatment duration (the duration of all treatment courses post-baseline to week 96, as above) is a secondary outcome for evaluating the management strategy from the patient and programme perspective. Treatment duration is preferred as the burden of treatment is likely more closely related to the period when treatment is expected to be taken rather than the number of days on which treatment was actually taken or provided.

*Total treatment daily doses taken (the number of days that counted as qualifying days) in all treatment courses post-baseline to week 96 is an alternative secondary outcome for evaluating the management strategy from the patient and programme perspective. This is similar to total treatment duration but does not count missed days or periods of treatment interruption. This is also a measure of burden on patient and programme.

8.5.4 Treatment completion and default

Definitions in 4.6.7

<u>Treatment completion and default categories, initial treatment course</u>

The number and proportion of patients in each of the treatment completion categories and the aggregate categories (completed treatment; defaulted treatment; defaulted treatment [including pre-baseline doses in the total]) as defined in 4.6.7 will be presented for each of the TMS (complete and partial) and SMS arms for the initial treatment course.

<u>All treatment default categories</u>

The number and proportion of patients with treatment default in each arm will be presented for the following categories:

- Treatment default during the first 8 weeks*
  Defined as treatment cessation within 56 days following baseline (complete cessation for >56 days, or until re-start of treatment for relapse)
  *This is a secondary outcome for analysis of strategy from the programme perspective

- Treatment default before completion of the initial treatment course (as defined in 4.6.7 above)
  This is an alternative definition used for the analysis of strategy

- Treatment default during any treatment course
  This is an alternative definition used for the analysis of strategy

Treatment default during the first 8 weeks will be compared between TMS (complete) and SMS arm by appropriate test.

8.5.5 Adherence

Definitions in section 4.6.10

Adherence will be presented for each of the SMS and TMS (complete and partial) arms during the following two periods:

1) Initial treatment course (any regimen) to week 8
    - Number of qualifying treatment days: n, median, range, IQR
    - Proportion of qualifying treatment days, %: median, range, IQR
    - Number of patients in each of the following categories of missed days:
        - < 7 missed days, n (%)
        - 7-13 missed days, n (%)
        - >14 missed days, n (%)

2) Total treatment courses (post-baseline, any regimen) to week 96
    - Number of qualifying treatment days: n, median, range, IQR [also required in 8.5.3 above]

- Proportion of qualifying treatment days, %: median, range, IQR

These are secondary outcomes from the programme perspective (with adherence in the first 8 weeks being the main outcome; adherence over the total treatment course is a supplementary outcome).

The proportion of qualifying treatment days (%) will be compared pairwise between each *complete* TMS arm and the SMS arm by suitable test (see section 8.1).

8.5.6 Treatment observation

Definitions in section 4.6.11.

For each arm and for the study population as a whole, the number and proportion of patients for which each method was recorded as the main method of observation in the initial period of post-baseline treatment; and the number and proportion of patients for which it was used as the main method of observation at any time during the initial treatment course will be presented as descriptive data.

8.6 Analysis of the primary outcome

8.6.1 Main analysis of the primary outcome

The number and proportion (within the mITT population) of participants in each TMS arm and the SMS arm classified as having satisfactory, unsatisfactory and unassessable outcome (definitions in Sections 4.12.1 and 4.12.2) will be presented.

The number of participants (%) meeting the component definitions of unsatisfactory outcome and unassessable outcome will be listed in the following summary categories (precise definitions in 4.12.1 and 4.12.2):

Unsatisfactory outcome

- On TB treatment at week 96
- Ongoing TB disease activity at week 96 (definitive, presumed, possible; listed separately)
- Death by week 96 (unless unrelated to TB)
- Did not attend week 96 visit and lacks evidence of good outcome at last attended visit and/or has evidence of bad outcome on telephone assessment or friend/relative assessment at week 96

Unassessable outcome

- Death by week 96, cause unrelated to TB
- Single positive sputum culture at week 96

- Did not attend week 96 visit, has evidence of good outcome at last attended visit but lacks information from telephone visit (patient or friend/relative) to assess disease activity at week 96

Each of the disease activity research criteria (section 4.11.2); and the combined criteria for possible (A and B only) and presumed (C with either A or B or both) will also be presented without statistical comparison between arms.

For the primary endpoint comparison, the difference in proportion of participants with an unsatisfactory outcome and a two-sided 97.5% confidence interval for that difference will be calculated by a generalized linear model with binomial distribution and identity link function, comparing each *complete* TMS arm with the SMS arm. Included in the model will be randomisation stratification factors (site and baseline risk stratum; strata may be combined dependent on their size). Alternative methods will be used in the event of non-convergence of the model.

The use of a two-sided 97.5% confidence interval for the primary outcome in assessment of non-inferiority is based on a Bonferroni correction for multiplicity, on the assumption that two *complete* TMS arms would continue through to the final analysis. If fewer or more than two *complete* TMS arms continue through to the final analysis, the correction and confidence interval will be adjusted accordingly.

A *complete* TMS arm will be declared to be non-inferior to the SMS arm if the upper limit of the two-sided 97.5% confidence interval for the difference between the two arms in the proportion of patients with unsatisfactory outcome (*complete* TMS arm minus SMS arm) is less than 12% (justification of non-inferiority margin in Section 10.3 of the trial protocol).

The primary comparison for non-inferiority will also be presented as the Bayesian probability that the between-group difference in the percentage of participants who would attain unsatisfactory outcome status (*complete* TMS arm minus SMS arm) would be less than 12% (additional thresholds may be presented in exploratory analyses, see below). The approach will follow that of Laptook et al (2017) and use a flat uninformative prior distribution.

8.6.2 Sensitivity analyses of the primary outcome

The following sensitivity analyses of the primary outcome will be performed, comparing the proportion of patients with unsatisfactory outcome in each *complete* TMS arm versus SMS arm only:

<u>Sensitivity analysis 1</u>
mITT assessable population (unassessable patients excluded; defined in section 6.5)

<u>Sensitivity analysis 2</u>

Per-protocol population (patients with inadequate initial treatment excluded; defined in section 6.6)

<u>Sensitivity analysis 3</u>
mITT population, unsatisfactory outcome restricted to definite and presumed TB according to disease activity research criteria; possible TB (section 4.11.2) re-classified as unassessable.

8.6.3 Exploratory analyses of primary outcome on all arms

The main analysis of primary outcome in the mITT population (done with individual complete TMS arms) will also be done for the following arms/combination of arms:

- All *complete* TMS arms combined, with numbers, proportions, 95% confidence interval and component breakdown as described above. The combined TMS arm will be compared in a single pairwise comparison with the SMS arm, using the same corrected confidence interval for the difference (97.5% two sided) used for multiple group comparisons in the main analysis (appropriate in the context of multiple analyses). Non-inferioirty will be formally assessed using the same boundary (12%) as for the individual *complete* arms.

- The individual *partial* TMS arms, with descriptive statistics as above. The estimated difference for a pairwise comparison with the SMS arm will be presented using the same confidence interval used in the primary analysis above (ie. 97.5% two-sided, if two *complete* arms go through to final analysis), but this is presented for descriptive purposes and non-inferiority will not be formally assessed.

For patients that extend follow-up beyond week 96 to complete follow-up to 48 weeks post-re-treatment (at a post week 96 re-treatment follow-up final visit), the primary outcome will be re-evaluated for these patients using the same algorithm used for week 96. The outcome at week 96 will be replaced by the revised outcome for these patients and the overall outcomes for the group recalculated.

8.6.4 Subgroup analyses of the primary outcome

The proportion of participants with unsatisfactory outcome on the primary analysis will be compared pairwise between each of the *complete* TMS arms and the SMS arm in a number of pre-specified subgroups, under the same population and definitions as the main analysis. Subgroup analyses will be performed with adjustment for site and baseline risk strata (standard definition) to reflect the randomisation strata (and the adjusted approach used for the main analysis).

The subgroups have been selected based on specific factors that might affect the primary outcome of the strategy (through increasing late relapses or through higher mortality during

relapse) such as markers of high disease burden at baseline, presence of underlying co-morbid disease at baseline, and standard demographic and socioeconomic factors.

The subgroups and subcategories that will be considered for analysis are:

- Sex
- Age (<35, 35-50, > 50)
- Country (of study site)
- Education (none/primary; secondary; tertiary)
- Smoking status (never, current/past)
- HIV co-infection
- History of diabetes (defined in section 4.7.6)
- History of Chronic Obstructive Pulmonary Disease
- Malnutrition (body mass index <18.5)
- Cavitation on baseline CXR (expanded definition; none, largest cavity ≤ 4cm, > 4cm)
- Proportion of total lung affected on CXR (expanded definition; <20, 20-50, >50%)
- Smear status at baseline (expanded definition; negative, scanty/1+, 2+, 3+)
- Baseline risk stratum using the standard or expanded definitions (section 4.10)
- Isoniazid resistance at baseline
- MRC Dyspnoea Scale score at baseline (1, ≥ 2)

A particular subgroup analysis will not be performed, unless of special interest, if there are less than 30 participants in a subgroup category within each of the two arms (TMS arm and SMS arm) that are being compared. In such cases, thresholds may be modified or subgroup categories may be consolidated to achieve the minimum number of participants, or the subgroup analysis will be omitted.

The subgroup analysis may be repeated for the alternative outcome classifications and for the per-protocol population as described under the sensitivity analyses for the primary outcome, above.

Additional exploratory subgroup analyses may be added later in the trial to include additional biomarkers as these are identified or as measurements become available.

8.7 Analysis of Secondary Outcomes from the patient perspective - patient-centred outcomes and measured respiratory disability

Analyses of secondary outcomes from the patient perspective that relate to efficacy will be done in the mITT population (6.2.1). Analyses of secondary outcomes from the patient perspective that relate to safety (e.g. respiratory disability) will be done in the ITT population (section 6.1).

Only the *complete* TMS arms will be formally compared with the SMS arms; no formal statistical comparisons will be made between the *partial* TMS arms and the SMS arm.

The main analysis of secondary outcomes will be done on data collected at or up to the in-person week 96 visits.

For patients not attending the week 96 visit:

- Outcomes that are continuously assessed - total days on TB treatment, time off work/study, adverse events, adherence, acquired drug resistance and community transmission risk - will be determined from data collected until the last visit attended in-person prior to week 96.
- Outcomes that are assessed by a specific evaluation done at week 96 - acceptability, quality of life, respiratory disability – will be determined from the results of the same evaluation done at week 48 (or at the time of withdrawal, if the patient withdrew after week 48 and the evaluation was perfomed at the time of withdrawal). If the evaluation was not performed at or after week 48, the outcome will be classified as missing.

An additional analysis of secondary outcomes will be performed including any relevant data from the post week 96 retreatment follow up (done in patients who re-start treatment later in follow up and require continued follow-up after week 96 to complete 48 weeks of post retreatment follow-up; see section 4.3). For this analysis, week 96 secondary outcomes will be revised to include any relevant data collected at in-person visits from the patients undergoing post week 96 retreatment follow-up that is obtained up until closure of the trial site.

8.7.1 Acceptability of the strategy

Definitions in section 4.14.1

The frequency and proportion of patients selecting each response to the individual questions or subquestions on the patient acceptability questionnaire will be summarized separately at week 48 and 96 for each arm.

The proportion of patients for whom the intervention is designated as acceptable in the difficulty and anxiety domains (defined in section 4.14.1) will be presented for each arm, together with the proportion with a non-evaluable response.

Summary statistics will be presented for the difficulty summary score and anxiety summary score (combined across weeks 48 and 96; as described in section 4.14.1) for each arm; no formal comparisons between arms.

Summary statistics will be presented for the motivation summary score (at week 48 only; derived as described in section 4.14.1) for each arm; no formal comparisons between arms.

The proportion of participants recommending each treatment option will be presented (at week 96 only) for those randomised to a TMS arm, with responses (2 months, 6 months, no preference/unsure); no formal comparisons between arms.

It is recognized that no single quantitative measurement can capture the complex contruct of patient acceptability. The primary evaluation of acceptability of the strategy will be the proportion of participants in each of the complete TMS arms that stated that they would recommend the 2-month treatment strategy to a friend or expressed no preference or were unsure. Other descriptive measures listed above will be used to understand the strength of the support (or otherwise) expressed for the TRUNCATE-TB management strategy. Additional measures such as rates of loss-to-follow-up and withdrawal may also provide an indication of acceptability of the strategy to patients and will be considered in the overall assessment of acceptability.

Measures of acceptability derived for participants randomised to the SMS may be useful for interpreting findings in the TMS arms, but no formal statistical comparisons will be made between arms.

8.7.2 Total days on TB drug treatment

Definitions in section 4.6.5 and 4.6.6.

Comprehensive descriptive data on the duration of treatment courses and number of treatment daily doses will be presented as background for the treatment regimen (section 8.5.3).

Total days on TB drug treatment will be presented in two ways – by the total duration of the treatment courses (including interruptions and complete missed doses; excluding periods of default) in a patient; and by the median number of daily treatment doses taken over the whole trial duration (excludes interruptions and complete missed doses) in each patient.

The median values for each of these parameters will be compared pairwise between the TMS (complete) arms and SMS arm.

8.7.3 Time off work or study due to illness/treatment

The total days missed from work or from study or from both (as defined in section 4.12) from randomisation until the week 96 visit will be presented as mean, median, IQR, minimum and maximum for each treatment arm. TMS (complete) and SMS Arms will be compared by appropriate statistical methods (section 8.1).

8.7.4 Quality of life

Derivation of summary scores in 4.14.3

The median score for each domain and for the PHS and MHS summary score will be provided for each group at week 96. No formal statistical comparison will be made.

A general linear mixed model will be used for pairwise comparison of the TMS (complete) and SMS arms in the summary PHS and MHS quality of life indexes from baseline to week 96.

8.7.5 Health Status

The mean score (SD) and the proportion of participants with a score of ≥ 3 in each of the 5 domains at week 96 will be tabulated.

A general linear mixed model will be used to compare the EQ-5D overall index score of the treatment arms over the follow-up period from baseline (excluded) to week 96.

8.7.6 Respiratory disability

MRC dyspnoea scale

The number and proportion of patients with respiratory disability by the MRC dyspnoea scale (a score of ≥ 3, as defined in section 4.12) at week 96 will be presented by treatment arm. The number of patients in each score category on the MRC dyspnoea scale at week 96 (1-5) and the number and proportion with *new* respiratory disability at week 96 (defined as section 4.12) will also be shown as descriptive data (not for statistical comparison). The proportion with respiratory disability at week 96 will be compared by appropriate statistical methods (section 8.1).

Spirometry

The proportion of spirometry assessments at weeks 8, 48 and 96 that are classified in each of the Grades (A to E) of the ATS/ERS system will be reported for the whole study population as an indicator of overall quality of the spirometry assessments.

Descriptive statistics will be presented for FEV1% (including category of impairment), FEV1, FVC, and FEV1/FVC ratio at each visit. Change in FEV1%, absolute FEV1, FVC and FEV1/FVC ratio from week 8 to weeks 48 and 96 (all as described in section 4.7.5) will also be shown as descriptive data (not for statistical comparison).

The number and proportion of patients with severe respiratory disability by spirometry (FEV1 < 50% predicted, derived as described in section 4.7.5) at week 96 will be presented by treatment arm. Arms will be compared by appropriate statistical methods (section 8.1).

The number and proportion with new respiratory disability (defined in section 4.7.5) will also be shown (not for statistical comparison).

If the absolute proportion with severe respiratory disability is higher by >5% in one or more TMS Arms compared to the SMS arm at week 96, further exploratory analyses may be performed including:

- An analysis limited to results of spirometry assessments that meet ATS/ELS criteria of Grade A-C only.
- An analysis examining the change in spirometry parameters (FEV1, FVC and FEV1/FVC ratio) using a linear mixed effects model with repeated measurements. The model will include change from week 8 as a response variable, site, treatment arm, disease burden at baseline (smear grade and cavities).

8.8 Analysis of secondary outcomes from the patient perspective – clinical adverse events

Incident AEs and SAEs occurring from randomisation to week 96 will be included in the strategy analysis

Analysis of adverse events will be done in the ITT population (section 6.1). Safety outcomes will be compared between individual *complete* TMS Arms and the SMS arm. Descriptive safety data will be presented for the individual *partial* TMS Arms, but no formal statistical comparison will be made with the safety in the SMS arm.

“Incident” AE and “related” AEs and counting rules are defined in section 4.8.4 and 4.8.5.

<u>Tables of reported adverse events, general approach</u>

Tables will show number and proportion (%) of patients who experienced one or more events in each category and the total number of events in each category in each treatment arm (in columns).

For each category a patient will only be counted once, even if there are multiple occurrences of the same category of event in the same patient.

The standard international order of SOCs will be used for all tables, with events listed by system organ class (SOC) and then subcategorised by preferred term (PT).

<u>Overall summary table</u>

A summary table will be presented including the following categories of AEs:
- AE (all, any grade)
- AE of grade 3 or 4 severity (all)
- AE of grade 3 or 4 severity related to TB medication
- AE of grade 3 or 4 severity related to TB disease
- SAE (all, including death)
- SAE related to TB medication

- SAE related to TB disease
- Death (all)
- Death related to TB medication
- Death related to TB disease

The main presentation of AE results will be without consolidation of TB symptoms as an aggregated event and with the grade of TB relapse as allocated by the site based on social and functional status; and with component symptoms and their grades presented separately (as in 4.8.6). The AE results will also be presented as a sensitivity analysis using the *TB relapse aggregated event* classification (as described in 4.8.6), with TB symptoms consolidated into a single relapse event, and the event graded using the highest grade of the component symptoms.

<u>Tables and listings of specific AE types</u>

(i) Grade 3 or 4 AEs (all) presented as a table with rows for total AEs, and each SOC and PT subcategory.

(ii) Grade 3 or 4 AEs considered related to TB drugs presented as a line listing for each arm, with the SOC, PT and grade.

(iii) Grade 3 or 4 AEs considered related to TB disease presented as a line listing for each arm, with the SOC, PT and grade.

(iv) SAEs (all) presented as a table with rows for total SAEs and SAE category (fatal*, life-threatening etc).

(v) SAEs considered related to TB drugs presented as a line listing for each arm, with the SOC and PT.

(vi) SAEs considered related to TB disease presented as a line listing for each arm, with the SOC and PT.

*Death after close of week 96 analysis window is not included in category of “fatal” SAE.

<u>Statistical comparisons</u>

Safety outcomes in each *complete* TMS arm will be compared pairwise with the SMS arm for the following secondary safety outcomes (methods described in 8.1):

- Proportion of patients with at least one AE of grade 3 or 4 severity (including death)
- Proportion of patients with at least one SAE (all, including death)
- Proportion of patients who died (prior to closure of week 96 analysis window)

All other adverse event types will be presented as descriptive data only and no formal statistical comparison between arms will be made.

8.9 Analysis of secondary outcomes from the programme perspective

Analyses of secondary outcomes from the programme perspective that relate to programme implementation efficacy will be done in the mITT population. Analyses of secondary outcomes from the programme perspective that relate to programme implementation safety (acquired drug resistance and community transmission risk) will be done in the ITT population (section 6.1).

Results will be reported for all TMS arms (*complete* and *partial*) and SMS arm, but formal statistical comparisons will be made only between the *complete* TMS arms and the SMS arm.

8.9.1 Adherence to TB medication

Adherence (number and proportion of qualifying daily doses taken; i.e. course duration minus daily doses missed) during the first 8 weeks (56 days) from baseline and for the total treatment course (initial and any retreatment courses) from baseline until week 96 will be presented as mean, median, IQR, minimum and maximum for each treatment arm.

The proportion of participants missing more than 14 days of treatment (strict randomised regimen or standard regimen if switched) within the first 56 days will be compared by appropriate statistical methods (section 8.1; see also 8.5.5 above). A supplementary comparison of adherence over the total treatment course will also be performed.

8.9.2 Treatment default

The number and proportion of patients in each arm who defaulted treatment (defined in section 4.5.6) in the first 8 weeks will be presented for each treatment arm. Arms will be compared by appropriate statistical methods (section 8.1). These are described in 8.5.4 above. Numbers and proportions with default during in the initial treatment course will also be presented.

8.9.3 Acquired drug resistance

The proportion of patients who have confirmed acquired drug resistance (defined as in section 4.9.3) during the trial (to week 96) will be presented for each treatment arm, with a line listing of the drugs affected.

The proportion will also be presented for an expanded definition including unconfirmed cases of drug resistance. An additional analysis may include the follow-up period extending to the final trial visit in those who had retreatment and required extended follow-up after week 96. Complete TMS and SMS arms will be compared by appropriate statistical methods (section 8.1).

8.9.4 Community transmission risk

The new household contact transmission risk score (simple and adjusted scores), total household contact transmission risk score (simple and adjusted scores), and the overall community transmission risk score will be presented in a table for each treatment arm (scores derived as described in section 4.15). They will be presented in two ways:

As a median (IQR) score for all participants that have experienced relapse in an arm, with the number of participants with relapse also indicated (the score range is likely to include 0 for those participants who experienced relapse but with no associated transmission risk); and in 3 categories (0, >0 – 5, > 5); thresholds may be adjusted depending on distribution of data).

Data will also be presented as a mean score for all participants in each arm to provide an overall indication of the risk of the strategy.

Community transmission risk outcomes will be compared between individual complete TMS Arms and the SMS arm by appropriate statistical methods (section 8.1). Community transmission risk will be presented for the individual partial TMS Arms, but no formal statistical comparison will be made with the SMS arm.

8.10 Analysis of other outcomes from the patient and programme perspective

8.10.1 Body weight

Change in absolute body weight from baseline over time will be shown for all treatment arms in a figure.

Descriptive statistics will be presented for absolute and % change in body weight from baseline to week 96 and compared pairwise between all complete TMS arms and the SMS arm. Percentage change from baseline to week 96 will also be presented in the following categories: proportion with weight stability (≤5% change), weight increase (>5%, > 10%) and decrease (>5%, >10%).

Given the potential differences in extent of recovery between arms during the initial treatment phase and the impact of relapses during follow-up, change in body weight from baseline to week 96 will also be evaluated using linear mixed effect models with repeated measurements.

The model will include:

- change from baseline as a response variable
- treatment, visit and randomisation stratification factors as fixed effects:
    - trial site – specifically by site in this case, rather than country, as body weight measurements are likely to be affected by the site-specific weighing scale used; any trial site that has less than 30 participants enrolled will be aggregated with

the next smallest trial site or sites within the same country to ensure a minimum sample size of 30 participants per site (or aggregate of sites); any country that has less than 30 participants in aggregate will be combined with the next smallest country to ensure a country sample size of at least 50 participants (see section 8.1)
    - risk stratum
- baseline as continuous covariate
- treatment by visit as interaction term
- patient as random effect
- visit will be included as a repeated effect in the model

An unstructured matrix will be used to model the variance-covariance structure within patient. If this model fails to converge, other variance-covariance structures - heterogeneous TEOPLITZ (TEOP), heterogeneous autoregressive of order one (ARH (1)), autoregressive of order one (AR(1)) or compound symmetry (CS) structures will be considered in the specified order to model the correlation between time points from the same patient.

The difference between LS-means of two treatment arms (standard treatment pairwise versus each boosted regimen), corresponding 95% confidence interval and p-value will be reported.

8.10.2 BMI

Descriptive statistics will be presented for absolute change in BMI from baseline to week 96; this will be compared pairwise between all *complete* TMS arms and the SMS arm.

A stacked area chart of the proportion of participants in each of the following BMI categories at week 96 will be presented for each of the TMS arms and the SMS arm: < 17, 17 to <18.5, 18.5-25, > 25 to <30, > 30.

If > 30% of participants in the SMS arm have BMI <18.5 at baseline, a Kaplan-Meier analysis of time to recovery of ideal body weight (BMI ≥ 18.5) will be performed in this subgroup that is underweight at baseline.

9 Analysis of the implementation of the management strategy

9.1 General

The strategy is a complex intervention. Following the identification of a patient with a diagnosis of tuberculosis, and a decision to treat, the following specific components of the intervention need to be considered. Options for each component (and those specified for this protocol) are:

1. Identification of patients suited for the strategy (trial eligibility criteria)
2. Initial treatment (boosted regimen, with option for switch or extension)
3. Initial treatment, decision to stop (minimum duration and symptom/smear criteria) …………………………………………………………………………………
4. Monitoring (symptoms, smear; [and cultures]) ………………………………………………………………………………..…
5. Re-treatment, decision to start (disease activity clinical management criteria)
6. Re-treatment (standard treatment, modified by resistance testing).
7. Re-treatment, decision to stop (24 weeks' fixed duration) ………………………………………………………………………………….
8. Monitoring (symptoms, smear; [and cultures])
   etc.

The selection of components of the strategy used for this trial was based on inference from existing trial data and rational deduction about what was feasible and likely to be needed for the strategy to be successful. Alternative approaches may further optimise the outcomes of the intervention. The overall success of the TRUNCATE-TB management strategy is evaluated in the main safety and efficacy analysis described in section 8. In that analysis, the strategy is analysed in its entirety without detailed examination of the performance of the components or their impact on overall outcome.

The aim of the implementation analyses described in this section is to evaluate the components of the complex intervention to determine:

- Feasibility and acceptability: assessed from adherence to protocol-specified strategy parameters and reasons given for non-adherence (obtained from specific CRF questions; or protocol deviation reports to the extent available). This may be assessed from the patient perspective or programme (site implementation) perspective or both.

- Utility: this will be determined by the relationship between the components of the intervention and intermediate outcome measures, principally microbiological outcomes (treatment failure/relapse; new resistance) and/or clinical disease to determine whether the component of the intervention is operating as expected.

We will then explore the independent relationship between various treatment implementation parameters with intermediate outcomes; and the relationship between initial treatment duration,

relapse and other variables with the primary strategy outcome and key secondary outcomes in a multivariable regression model.

The information gained in these analyses may allow a better understanding of how components of the strategy contribute to outcomes (primary and secondary outcomes from the strategy; as well as resource utilisation for health economic analyses). This may help understand the overall success or failure of the strategy tested in the main analysis and how it might be usefully modified for future implementation or study.

9.2 Analysis population and treatment arms

Two analysis populations will be used, depending on the analysis question.

- mITT population - used for some descriptive analyses of implementation of the initial regimen that assess adherence to the initial boosted regimen course parameters.
- mITT complete boosted cases population – used for most analyses of implementation. This population only includes those patients in TMS arms classified, based on their initial course of treatment, as “complete boosted” i.e. who took at least 54 days of boosted regimen and who stopped without continuing with standard treatment (as defined in section 4.6.7); as well as the patients in the SMS (used as a comparison for some analyses). This provides the cleanest population for analysing the effect of the other parts of the treatment strategy i.e. the post-treatment monitoring, assessment of relapse, and re-treatment. As this population excludes those participants in the TMS arms who switched for toxicity or who extend for persistent clinical disease, it may lead to biased estimates of efficacy. Hence the analyses will avoid drawing conclusions on regimen efficacy or making comparisons between TMS arms, or between TMS and SMS arms.

Initial descriptive statistics will be presented for individual TMS arms and an informal assessment of overall implementation parameters for the initial treatment regimen (in particular the proportion of patients that complete boosted treatment, or switch treatment, or relapse) will be made to determine whether these are broadly similar between arms (qualitative assessment, no formal testing for heterogeneity). If broadly similar then the results will be presented with all TMS arms combined; with supportive analyses stratified by arm on a few key parameters to demonstrate consistency.

Where there are major systematic differences in implementation between any of the individual TMS arms that may affect interpretation and conclusions, the approach will depend on whether these affect the complete arms or partial arms. If they affect one or more partial TMS arms only, then the affected arms will be omitted from the combined analysis and will be presented as sensitivity analyses. Where these differences are between complete TMS arms then the analysis will be done for all arms separately. For ease of interpretation, the same arrangement of TMS arms (individual or combined) will be used throughout all analysis.

9.3 Enrollment and follow-up

The CONSORT diagram used for the strategy analysis will be used, with the following changes:

- Removal of numbers for the per-protocol analysis
- Removal of the SMS arm
- Addition of regimen characteristics for the initial treatment in each arm (categorical definitions in section 4.6.7):
    - Complete boosted only
    - Boosted and standard for 24 weeks
    - Other
    - No treatment

For the complete boosted group (for each boosted regimen) only:

- Completed follow-up to 96 weeks
- Died
- Withdrawn
- Lost to follow-up

9.4 Demographics and Baseline Characteristics

Demographics and baseline characteristics will be summarised for the mITT population (as defined for the strategy analyses, see section 6.3). This is the same as the information presented for the main analysis in section 8.

The following site characteristics will also be summarised:

- Location: standalone TB clinic, clinic as part of specialist respiratory medicine hospital, clinic as part of general hospital
- Relationship with NTP: under NTP / independent of NTP
- Smear testing routine at site: Y/N
- Site has DOT clinic implementing daily (> 5 days per week) direct observation: Y/N

9.5 Overall evaluation of strategy implementation

Done in the mITT population (all). An overall description of the strategy as implemented in the trial will be presented for the following 3 parameters to provide a framework for interpretation of the components:

- treatment burden
- symptom burden
- microbiological disease burden.

9.5.1 Overall treatment burden over 96 weeks

Done in the mITT population (all). This will be shown as a stacked area chart, based on snapshot data of participants known to be taking treatment at each scheduled study visit from randomisation through to week 96:

Categories:
- Off TB treatment, under-follow up (in person or telephone visit within the previous 6 months)
- On TB treatment, boosted regimen
- On TB treatment, standard regimen
- No data - died, withdrawn or lost-to-follow-up - no visit within previous 6 months

Categorisation at each study visit will be based on the treatment prescribed (non-adherence will be ignored, except for treatment default).

The chart will be shown for all TMS arms combined and the SMS arm will be shown for illustration as a separate panel.

9.5.2 Overall symptom burden over 96 weeks

Done in the mITT population (all). Details in section 4.7.1.

Proportion of patients with each category of symptom score (0,1,2,3,4,5,6) at the 28 scheduled timepoints from baseline to week 96 will be shown as a stacked area chart, with an additional category for not assessed/missed visit. The chart will be shown for all TMS arms combined and the SMS arm will be shown for illustration as a separate panel.

Proportion of patients with each of the 6 individual TB symptoms present at scheduled visits from baseline to week 96 will be presented in a single figure with a line showing the prevalence of each individual TB symptom. The chart will be shown for all TMS arms combined and the SMS arm will be shown for illustration as a separate panel.

9.5.3 Overall microbiological disease activity over 96 weeks

Done in the mITT population (all). Disease activity over time to week 96, with snapshot classification of proportion of participants over time in sputum culture categories at each scheduled visit where sputum collection is mandated (as in section 4.9.8):

Proportion with sputum production and culture result at each scheduled visit at which a culture is mandated, presented as area chart (for all scheduled follow-up time points) with classification as in section 4.9.3:

Categories:
- Culture negative

- No sputum produced [attempted, unable to produce with clinical disease recovery; or met omission criteria will be imputed as negative in sensitivity analysis]
- Culture positive (positive and positive contaminated are combined)
- Culture Contaminated / Other
- Test not performed / no result
- No sputum produced [but symptoms present, or no data on symptoms; or did not meet omission criteria]
- Missed sputum collection (telephone visit / died / missed visit / lost-to-follow up)

A supplementary analysis will present the data in the same way with the subcategories of [No sputum produced, clinical disease recovery and No sputum produced, met omission criteria] reclassified as culture negative (section 4.9.1)

The chart will be shown for all TMS arms combined and the SMS arm will be shown for illustration as a separate panel.

9.6 Analyses of feasibility and utility of each of the strategy components

9.6.1 Identification of patients suitable for the strategy

Feasibility
Adherence to the pre-specified selection criteria of patients will be evaluated in the main strategy analysis.

Utility

For eligibility, some information on utility of criteria in predicting outcomes will be obtained from subgroup analyses.

These are not considered further in the implementation analysis in this section.

9.6.2 Initial treatment

The sterilising activity and bactericidal activity of the boosted regimens will be analysed as described in sections 11 and 12. The optimal drug dose will be analysed in the PK analyses. The analyses here provide additional information on implementation and clinical efficacy of the boosted regimens.

This analysis will use both the mITT population (all) and the mITT complete boosted cases population.

Feasibility

Done in mITT population (all):

- Proportion of patients in each classification category for the initial treatment course (categories as in section 4.6.7), n (%)

Done in mITT complete boosted cases group only:

- Duration of initial treatment: days, median, IQR, range
- Number of daily doses of initial treatment: days, median, IQR, range
- Main method of treatment supervision used (categories defined in section 4.6.11), n (%)
- Adherence to boosted regimen (Day 1 to Day 56), defined in section 4.6.10
    - Proportion of missed days:, %: median, range
    - Number of missed days, in each of the following categories:
        - < 7 missed days, n (%)
        - 7-13 missed days, n (%)
        - ≥14 missed days, n (%)
    - Proportion with ≥ 4 consecutive days missed

Utility

(i) Symptom resolution (see definition in section 4.7.1):

Done in mITT complete boosted cases only. Derivation of data in 4.7.1.

TB symptom prevalence
Symptom prevalence data will be censored at the symptom assessment result done at the last visit prior to or at cessation of treatment.

This is a cross-sectional (snapshot) assessment at each study visit during initial treatment. The number and proportion of patients with each symptom and the total symptom score (numerical categories) will be presented for the following timepoints, censored at end of treatment: baseline, weeks 4, 8, 10, 12.

Proportion of patients with each category of symptom score will be shown as a stacked area chart, with an additional category for not assessed/missed visit. The chart will be shown for all TMS arms combined and the SMS arm will be shown for illustration as a separate panel.

Both in person and telephone visits will be considered within each visit window. For details of approach see 4.7.1.

Time to symptom clearance

Done in mITT complete boosted cases population only. Symptom clearance is a time-to-event measure (defined in section 4.7.1).

The analysis period will extend to 16 weeks from randomisation to allow for a prolonged period of boosted treatment (up to 12 weeks) and also for ongoing recovery (and symptom resolution) that may occur after the end of the boosted treatment course.

For the main analysis, symptom clearance is taken as absence of all 6 TB symptoms (i.e. symptom score of 0). The analysis may be repeated separately for the symptom of cough; and for any other individual symptoms that are present in >40% of patients at baseline.

Time from baseline to symptom clearance will be shown as a Kaplan-Meier curve with median (IQR) time to symptom clearance.

<u>(ii) Smear resolution</u>

Done in complete boosted cases only

Smear prevalence
This is a cross-sectional (snapshot) assessment at each study visit. The number and proportion of patients with the following smear results categories will be presented at key scheduled visits (baseline, weeks 4, 8, 10, 12; and the end of treatment visit itself) censored at the end of treatment:

- Smear positive 3+
- Smear positive 2+
- Smear positive 1+/scanty
- Smear negative
- No sputum produced – [and no symptoms/met omission criteria-imputed negative, section 4.9.1]
- No sputum produced – no clinical disease recovery [non-evaluable]
- Test not performed / no result
- Missed sputum collection

For this analysis, the subcategory of [No sputum produced, clinical disease recovery] will be reclassified as smear negative (section 4.9.1)

Time to smear clearance

Done in complete boosted cases only. Time from randomisation to smear conversion to negative (defined in section 4.9.5), median (IQR) shown as Kaplan-Meier.

For this analysis, the subcategory of [No sputum produced, clinical disease recovery] will be reclassified as smear negative (section 4.9.1)

The analysis period will extend to 16 weeks from randomisation to allow for a prolonged period of boosted treatment (up to 12 weeks) and also for late clearance of smear that may occur after the end of the boosted treatment course (as for symptoms above, this cannot be affected by smear reversion).

A sensitivity analysis will be done using an analysis with the follow-up period ending with the day of end of boosted treatment (section 4.9.9).

(iii) Culture resolution

Done in complete boosted cases only

Prevalence of positive culture
Cross-sectional (snapshot) assessment at each study visit. The number and proportion of patients with the following culture results categories will be presented at key scheduled visits (baseline, weeks 4, 8, 10, 12; and the end of treatment visit itself) censored at the end of treatment:

Categories:
- Culture positive (Culture positive and contaminated combined)
- Culture negative
- Culture Contaminated / Other
- Test not performed / no result
- No sputum produced – clinical disease recovery/met omission criteria [may consider negative, section 4.9.1]
- No sputum produced – no clinical disease recovery/did not meet omission criteria [non-evaluable]
- Missed sputum collection (telephone visit / died / missed visit / lost-to-follow up)

The analysis will be performed for TMS arms combined and a separate panel for the SMS arm shown for illustration (no formal statistical comparison).

Time to culture conversion

Done in complete boosted cases only. Definitions of culture conversion for this analysis are given in section 4.9.9.

The analysis period will extend to 16 weeks from randomisation to allow for:
- a prolonged period of boosted treatment (up to 12 weeks) due to interruption/non-adherence and/or extension due to persistent clinical disease
- late culture conversion after the end of the boosted treatment course.

A sensitivity analysis will be done using an analysis with the follow-up period ending with the day of end of boosted treatment (section 4.9.9).

For this analysis, the subcategory of [No sputum produced, clinical disease recovery] will be reclassified as culture negative (section 4.9.1)

Time from baseline to culture conversion will be shown as a Kaplan-Meier curve and the median (IQR) time to culture conversion and the cumulative percentage with culture conversion by week 16 will be presented.

The same parameters will be presented for the sensitivity analysis in which the analysis period ends with the day of end of boosted treatment (16 weeks for the SMS arm used for comparison i.e. the same SMS parameters as the analysis above.

(iv) Overall clinical and microbiological status at end of treatment

Done in complete boosted cases only

Status at end of treatment visit (snapshot): n (%)

- Had positive symptoms alone
- Had positive smear alone
- Had positive culture alone
- Had both symptoms and positive smear
- Had both symptoms and positive culture
- Had positive symptoms, smear and culture
- Had no positive symptoms, smear, or culture

(v) New drug resistance during initial treatment

Done in the complete boosted population.

Classify as:

- New drug resistance detected during treatment
- New drug resistance detected after treatment (before next treatment course started)
- No new drug resistance – confirmed (at least one positive culture at or after end of treatment that shows no new drug resistance; no new resistance detected in any culture)
- No new drug resistance – assumed (no positive culture at or after end of treatment, or positive culture but susceptibility testing not done)

9.6.3 Initial treatment course, stop decision

In order for the strategy to have impact, a moderate to high proportion of patients enrolled in the strategy need to have short treatment. The strategy could be undermined if large numbers of patients switch or extend treatment to 6 months (or longer) for unnecessary reasons.

Feasibility

This analysis will be done in the mITT population

- Reasons for switch or extension with standard treatment (and therefore continuation to 6 months):
  - categories as in Form B8/B10/B12: n (%)

- Reasons for extension of boosted regimen alone beyond 56 doses: proportion that meet each criterion whilst still on treatment, at any point up to week 12:
  - Persistent symptoms consistent with active TB at any of week 8, 10 or 12
  - Positive smear at week 8, 10 or 12
  - Persistent symptoms and positive smear (at the same visit) at week 8, 10 or 12
  - Positive culture at week 8, 10 or 12

Utility
This will be shown for the mITT population and, separately, for the complete boosted cases only.

- Premature cessation of initial treatment: this is defined as cases of microbiological treatment failure or relapse. These will be presented as a table by duration of the initial treatment (categorised – see categories below).

- Excess initial treatment (maximum): this is the difference between the end day of the initial course of treatment minus the time of culture conversion, presented as:
  - Mean, median, IQR
  - Number (%) in each category of excess treatment:
    >0 to <4 weeks, > 4 to ≤ 8 weeks, >8 to ≤ 12 weeks, >12 weeks

Premature cessation and excess initial treatment (maximum) will be presented for the following categories of duration of initial treatment (daily doses):

<8 weeks, ≥8 to ≤ 12 weeks, >12 to ≤ 16 weeks, >16 to ≤ 20 weeks, >20 to ≤ 24 weeks, >24 weeks

9.6.4 Monitoring

The monitoring strategy comprises a surveillance component (regular monitoring of symptoms and smear) and an evaluation component (relapse assessment, triggered by a change in symptoms or smear).

The analysis of utility will focus on symptom and smear monitoring but will also examine weight monitoring.

Cultures were done for research purposes, but as results were returned to the managing site *de facto* they may have impacted the monitoring strategy. The analysis will also identify the number of cases of relapse where real-time culture results indicated relapse earlier than was suspected

by symptoms and smear monitoring - to assess the robustness of the monitoring strategy without cultures.

All of the analyses below are done in the complete boosted cases only

Surveillance component

Symptom monitoring

Feasibility

Proportion of the scheduled timepoints for symptom assessment from end of treatment to start of re-treatment, final trial visit, or week 96 visit (whichever is earlier) at which symptom assessment was done:

- In person: % of scheduled visits per patient (ignore protocol-mandated mode of visit)
- By telephone: % of scheduled visits per patient (ignore protocol-mandated mode of visit)
- No assessment: % of scheduled visits per patient

Interval between assessments, months: Mean (SD)

Maximum interval between assessments (categorised as ≤ 2m, > 2m to ≤ 4m, > 4m to ≤ 6m, > 6m): n,%

Proportion of follow-up time for which no assessment was done in the previous 4m

Symptom assessment will be classified as "done" if at least 1 box in the symptom assessment (for any of the 6 TB-symptoms) has been checked as "yes" or "no" (section 4.7.2).

Utility

(i) Association between symptom profile at end of treatment and outcome

- Symptom profile (at end of initial treatment course) in patients without confirmed treatment failure or relapse (later)
- Symptom profile (at end of initial treatment) in patients with treatment failure
- Symptom profile (at end of initial treatment and and post treatment) in patients with subsequent relapse

Proportion with each of the individual symptoms and with each category of total symptom score presented for each of the 3 outcome groups.

(ii) Time from end of treatment to symptom recurrence (defined in section 4.7.1); median (IQR); shown as Kaplan Meier.

(iii) Time from end of treatment to clinical disease progression (component A of disease activity clinical management criteria, described in 4.11.1): median (IQR); shown as Kaplan Meier; compared with (ii).

(iv) Assessing potential of symptoms to detect relapse:

Population: Patients without confirmed treatment failure (or presumed treatment failure where treatment was re-started); TMS arms only; complete boosted cases only

Method: case control

- Cases: patients with culture-confirmed relapse
- Controls: patients without culture-confirmed relapse who did not restart treatment for any reason, matched to cases by treatment arm and study site (extend to country for remaining cases that are unable to match at site); additional matching factors (in priority order) where possible to match more than 3 at site: baseline relapse risk stratum, age (above/below 50), sex. Up to 3 matched cases per control, provided that matching criteria met for treatment arm and study site/country.

Period for data evaluation:

- Start: 12 months prior to date of confirmed relapse (cultures) - for controls: 12 months prior to the date of confirmed relapse in the case with which the control is matched; or 4 weeks after end date of first course of treatment – whichever is later.
- End: date of restart of treatment in cases (for controls: restart of treatment date in the case with which the control is matched)

Descriptive comparison:

Overall symptom score for cases and controls at each of the following time points prior to and following confirmed relapse (cultures): n (%)
-6 to <-3m; -3m to <-1m; -1m to +1m; >1m to <3m; 3m-6m; > 6m

The predictive potential of symptoms may be explored in further, separate biomarker analyses.

[The analytical comparison will be developed further in the section on biomarker development].

Smear monitoring
Same patient population and selection criteria
Similar descriptive and analytical approach used for symptoms, as above

Weight monitoring

Dynamics of weight change from end of treatment – similar approach to above

At key visits: patients classified as

- weight increase vs end of treatment (>5%, > 10%)
- weight decrease vs end of treatment (>5%, >10%)
- weight stable vs end of treatment (≤5% change)

Employ similar approach to symptoms (above) to look at predictive potential for relapse

Impact of culture monitoring

Proportion of culture-confirmed relapse cases where earliest date of diagnosis of culture positive relapse precedes date of symptom recurrence or smear reversion to positive.

Culture positive (single culture): date of culture-positive relapse diagnosis: date the positive culture was collected + TTP (day) result for that culture + 3 days to allow for report.

Culture positive (confirmed on second consecutive culture): date of culture positive relapse diagnosis, as above but date of collection and TTP + 3 days is related to the second culture.

Evaluation component

<u>Feasibility of relapse assessment</u>

Population: complete boosted patients only, with culture-confirmed relapse

Relapse episode:
- Start of episode: date of first positive culture in patients with culture-confirmed relapse
- End of episode: start date of treatment

Timing relates to start and end of relapse episode.

Report the following for each type of assessment:

Number of assessments performed during the relapse episode, timing, number of assessments indicating disease progression, timing of first assessment documenting progression on that assessment

- Symptom assessment
- CXR assessment
- Smear
- GeneXpert
- Culture
- Documented relapse assessment

<u>Utility of relapse assessment</u>

The sensitivity and specificity of the tests and the disease activity clinical management criteria for detecting culture confirmed relapse (and non-relapse) will be further explored in separate biomarker analyses.

9.6.5 Retreatment, decision to start

These analyses will be done in the complete boosted cases only

Feasibility

For cases that restarted treatment, the proportion that had met disease activity clinical management criteria (and components thereof i.e. proportion that met criterion A, B, C, D) at time they started re-treatment will be presented

Utility

(i) Time between detection of relapse (or treatment failure) by disease activity clinical management criteria (section 4.11.1) and start of re-treatment in those with relapse, days: median IQR and mean.

Reasons for delay in re-starting (delay in clinician prescription or delay in patient start after prescription - times for both)

(ii) Disease severity at time of restart of treatment

n, % with the following (at time closest to the start of retreatment):

- Symptoms (6 TB symptoms; any symptoms; no symptoms)
- Smear positive (criteria – by smear grade)
- Culture positive
- Drug resistance (new)
- CXR progression since EOT film (Y/N)
- CXR % lung involvement
- MRC breathlessness scale (score ≥ 3)
- Maximum grade of relapse on DAIDS grading scale, based on interference with usual social and functional activities (reported on AE form; section 4.7.2)
- Maximum severity of TB-relapse related adverse events based on the most severe grading of any symptom (DAIDS grading scale) reported during the relapse episode
- Did the relapse result in hospitalisation for treatment (SAE) (Y/N)?
- Location of TB at relapse: pulmonary, extrapulmonary, both

(iii) number that re-started treatment without meeting clinical disease activity management criteria, and reasons if known

9.6.6 Re-treatment

These analyses will be done in the complete boosted cases only

Feasibility
Descriptive data for treatment parameters:

- Proportion given standard treatment
- Duration, adherence, completion versus default as for main strategy analysis and for initial treatment regimen (above)

Utility

- Resolution of symptoms: as for initial treatment regimen (above).
- Resolution of morbidity at end of treatment: as for initial treatment regimen (above).
- Stable culture conversion
- Absence of new drug resistance mutations: as for initial treatment regimen (above)

9.6.7 Retreatment, decision to stop

In order for the strategy to succeed, retreatment must be successful. Although it may not be necessary to have a full 6 months of retreatment, the strategy could be undermined if large numbers of patients discontinue the re-treatment course too soon, leading to repeat replapse episodes.

Feasibility
There were no formal cessation criteria defined for the re-treatment course hence feasibility of implementation protocol stopping criteria cannot be assessed.

Utility:
These analyses will be done in the complete boosted cases only. The same approach will be used as for the initial treatment course

Premature cessation of re-treatment: defined as cases of microbiological treatment failure or relapse. These will be presented as a table by duration of the re-treatment

Excess re-treatment: this is defined as the difference between the end day of the re-treatment course minus the time of culture conversion. This will be presented as continuous data (mean, median, IQR) as well as the number (%) in each category of excess treatment (see below).

The categories for excess re-treatment are: [>0 to <4 weeks],[> 4 to ≤ 8 weeks],[>8 to ≤ 12 weeks] and [>12 weeks].

Outcome
Percentages below are for outcomes in those initiating a second treatment course:

- Not completed re-treatment course by week 96, n (%)

- Completed re-treatment course by week 96, period of observation from end of second course of treatment to week 96: median, IQR, range; categories <24, ≥ 24 -48, > 48 weeks
- Completed treatment course by week 96, had subsequent treatment failure, n (%)
- Completed treatment course by week 96, had subsequent relapse before week 96
- Completed treatment course by week 96, had subsequent relapse after week 96 (will only be known from the close of trial telephone visit, 4.6.9)

9.7 Relationship between site characteristics, patient factors and implementation parameters

Associations between the following site and patient characteristics and implementation parameters will be explored by multivariable linear or logistic regression models, as appropriate to the nature of the implementation parameter outcome. Predictive factors will be classified as binary or ternary for ease of evaluation.

The analysis of implementation parameters will be done in the mITT population because looking at intermediate outcomes relevant to implementation of the strategy (so need poor and good implementation):

<u>Site factors</u>
As listed in 9.3 above:
- Clinic location / type: standalone clinic vs clinic in hospital
- Relationship with NTP: under NTP vs independent of NTP
- Smear testing routine at site: Y/N
- Site has DOT clinic implementing daily (> 5 days per week) direct observation: Y/N

<u>Patient factors</u>
- Age (<50, ≥ 50)
- Sex
- Socioeconomic status (working/student; not working)
- Socioeconomic status (education): total years of education: 0-13, ≥ 14
- Sputum smear grade at baseline (negative, scanty/1+, 2+/3+)
- CXR proportion of total lung affected (%): <20%, 20-50%, >50%
- Cavitation (absent; present)
- Risk stratum at baseline (categorized as lower, intermediate/higher)

<u>Key implementation factors (outcomes)</u>

Treatment
- Directly observed therapy by healthcare worker (in person or video) for first 8 weeks (Y/N)
- Missed < 14 doses of treatment in first 8 weeks (Y/N)
- Took boosted regimen ≥ 54 doses, stopped without switch/extension to standard treatment (Y/N)

Monitoring

- At least 1 interval of > 4 months (between week 12 and week 96) with no symptom assessment (Y/N)
- At least 1 interval of > 4 months (between week 12 and week 96) with no sputum sample attempted

9.8 Relationship between initial treatment, relapses and strategy outcomes

This main analysis will be done in the complete boosted cases

In simple terms the strategy represents a trade off between the benefits of shorter treatment against the negative consequences arising from relapses. Any analysis attempting to dissect these two aspects will be complicated by the competing risks from the higher rate of relapse accompanying short treatment; as well as a number of biases including selection bias of those patients who choose (or are prescribed) treatment extensions. Nevertheless, it is still likely to be informative for optimising the outcomes from the strategy to attempt to examine the impact of the two dimensions most likely to impact strategy outcomes.

The relationship between these two categories and the primary and secondary outcomes will be examined in linear or logistic regression models appropriate for the nature of the outcome variable. The initial model will include these 2 variables, as well as site, treatment group, age, sex, baseline risk stratum and any other baseline factors (without a strong likelihood of collinearity) that are found to have P < 0.3 on initial univariable analysis. A backwards elimination approach will be used to remove variables stepwise from the multivariable model that have P > 0.05 (with the exception of the variables of initial treatment duration and relapse that will remain in the model irrespective of P values).

An interaction term will also be introduced into the model for the interaction between treatment duration (boosted 8 weeks, 8-10 weeks, 10 to 12 weeks – equivalent daly doses) and relapse (yes/no), and stratum-specific odds ratios calculated.

The predictor variables to be considered for the model may include:

- Sex
- Age (<50, ≥ 50)
- Clinic site
- Socioeconomic status (working/student; not working)
- Socioeconomic status (education): total years of education: 0-13, ≥ 14
- Baseline risk stratum (lower, versus intermediate / higher)
- Overall TB severity grade at screening (DAIDS Grade 1 vs 2-4)
- Sputum smear grade at baseline (negative, scanty/1+, 2+/3+)
- CXR proportion of total lung affected (%): <20%, 20-50%, >50%
- Treatment arm

- Boosted duration 8 weeks vs >8 to 12 weeks
- Directly observed therapy by healthcare worker (in person or video) for first 8 weeks (Y/N)
- At least 1 interval of > 4 months (between week 12 and week 96) with no symptom assessment (Y/N)
- At least 1 interval of > 4 months (between week 12 and week 96) with no sputum sample attempted
- Relapse: defined as re-start of treatment for presumed or confirmed relapse; vs no restart of treatment (patients who cannot be cleanly classified on this variable – who restart treatment for some other reasons will be excluded from the model)

Several models will be developed for key primary and secondary outomes at week 96, that may include the following outcomes:

- Unsatisfactory clinical outcome (primary outcome)
- Patient acceptability (overall recommendation to friend Y/N)
- Death
- Severe respiratory disability
- Acquired drug resistance
- Late relapse (>6 months after end of initial treatment course)

Models will not be performed if < 20 outcomes.

10 Analysis of the health economics of the management strategy

An outline summary of the planned health economics analysis is given below. A detailed analysis plan will be presented separately.

10.1 Analysis population and treatment arms

The mITT population will be used for the health economics analysis, consistent with main efficacy analyses of the strategy.

Due to differences in the cost of regimens, the monitoring approach required, and potentially the incidence of severe AEs and SAEs, the main analysis will be done separately for the TMS arms. The main analysis will compare the individual *complete* TMS arms, compared pairwise with the SMS arm.

Similar to the approach used in the strategy implementation analysis, an informal assessment of overall implementation parameters (treatment and monitoring) and strategy outcomes will be made to determine whether these are broadly similar between arms (qualitative assessment, no formal testing for heterogeneity). Where implementation and outcome are broadly similar across all the TMS arms, a secondary analysis will be presented with all TMS arms combined; with supportive analyses stratified by arm on a few key parameters to demonstrate consistency.

10.2 Analysis general

The health economic analysis for the study will consider the patient, the programme, the health system (direct medical costs) as well as the overall societal cost (inclusive of indirect medical costs) over the period of the trial.

Costs will cover the use of TB medication, laboratory tests, hospital, primary care and community health services. Routinely available estimates of unit costs that best reflect long run marginal opportunity cost will be attached to resource use to obtain a cost per patient over the period of follow-up to the week 96 visit in the initial analysis. An additional analysis may incorporate data up to the final trial visit (where this occurs after week 96 in patients who commence retreatment after week 48).

For the within-trial analysis, the differential cost of the management strategies will be related to their differential outcomes in terms of the primary outcome. The relative cost-effectiveness of the alternative management strategies (explored and developed in the analysis of implementation of the management strategy, section 9) will then be assessed using standard decision rules and a full stochastic analysis will be undertaken.

A cost-utility analysis will also be conducted using the EQ-5D data collected at each visit. Assessment of cost-utility will be based on differential mean costs and QALYs between the TMS arms and the SMS arm. If the more effective management is also the more costly, an incremental cost-effectiveness ratio (ICER) will be calculated as the ratio of differential mean costs and

differential mean QALYs, which gives the cost per additional QALY gained. To assess value for money, estimated ICERs will be compared with appropriate cost-effectiveness thresholds published for the individual countries, or typical thresholds used for assessing benefits of TB treatment in resource-limited settings.

Further scenario analysis will be conducted, including the analysis of a maximum “break-even” rate of relapse which offsets the net present value of the intervention benefits.

Depending on trial findings, the within-trial analysis will be augmented by extrapolation beyond the trial follow-up to predict the implications of any difference in clinical endpoints in the trial for subsequent quality-adjusted survival duration and long-term resource costs. This will inform the question of whether any differences in management costs between the treatment arms are offset by reduction in other treatment costs or health improvements in the long-term.

11 Analysis of efficacy and safety of boosted regimens

11.1 General

Although TRUNCATE-TB is designed primarily to answer the strategy question, the follow-up data collected after cessation of the boosted regimens provides a unique opportunity to assess the sterilizing efficacy and safety of several novel regimens of two months' duration.

This information may be useful as a supplement the evaluation of the performance of the strategy and contribute to revision of the strategy for future implementation or trials. It may also provide information relevant to assessing the overall feasibility of achieving a regimen of 2 months duration that has a sufficiently high cure rate to be acceptable for use in programmes. The configuration of the regimens (several of which differ by a single drug) may also allow inferences about the relative sterilising efficacy of drugs.

The target sample size of 180 for each arm in the trial is substantially larger than that typically used to assess bactericidal activity in Phase 2b trials (measured by the rate of sputum culture conversion, described in section 12). However, it is generally smaller than that typically used to assess definitive sterilising efficacy of regimens tested in Phase 3 trials. The primary outcome parameter for assessing regimen efficacy will be a classification of "unfavourable" on the outcome of relapse-free cure. Unfavourable outcome is often used (variably defined) to assess sterilising activity of TB drug regimens in Phase 3 trials.

The analysis approach for determining unfavourable outcome for the regimen analysis is similar to that used for satisfactory outcome on the management strategy - assigning an overall outcome based on the status at the end of 96 weeks' follow-up. However, in the regimen analysis, events occurring during early follow-up such as changes in initial treatment, occurrence of treatment failure or relapse, and initiation of re-treatment influence the outcome - whereas these are ignored in the analysis of the strategy primary outcome. The differences between the outcomes are summarised in section 4.13.

Phase 3 trials have used unfavourable outcome on a measure of relapse-free cure over periods ranging from 12 to 24 months. In this trial we chose 96 weeks to align with the primary outcome for the strategy and to allow for the possibility that there might be a higher proportion of late relapses than seen in earlier studies given the different nature of the treatment regimens.

The main analysis will present the outcome as the proportion experiencing unfavourable outcome rather than time to the outcome as this is easier to interpret as a measure of the overall treatment efficacy relative to existing treatments. A secondary analysis using a time-to-event approach for unfavourable outcome will also be done and may provide more power for differentiating between the individual boosted regimens.

Given the relatively modest sample size for each of the groups, the trial may not have sufficient power to make a definitive comparison between the two-month boosted regimens and the 6-month regimen using a typical margin of non-inferiority used for a Phase 3 trial. In order to do

so with this sample size, we would need to hypothesise that the boosted regimens at 2-month duration had equivalent or superior sterilising efficacy to the 6-month regimen; whereas the underlying premise of this trial is the opposite – that the markedly shorter regimens would achieve a lower rate of sterilising efficacy (but that any adverse consequences of that could be mitigated by the monitoring and re-treatment strategy). Thus, it is not possible to construct a plausible *a priori* hypothesis of non-inferiority (or indeed superiority) for evaluating the 2-month boosted regimens against the standard of care that could be tested at a conventional, pre-specified, level of statistical significance.

Instead, the approach taken for this exploratory analysis is to present the efficacy comparison in the form of a Bayesian probability that the difference in the proportion of unfavourable outcome in a boosted regimen compared to the standard treatment regimen is no greater than 12%. The 12% margin was used in the STAND trial for non-inferiority of the test regimen versus control and is justified, especially given the more radical reduction in treatment duration being tested (2 months versus 4 months in STAND). The analysis approach will provide an estimate of the probability that this goal could be met with the boosted regimen(s) evaluated in the trial, which would be informative for determining whether a two-month regimen is a realistic goal for TB treatment. The analysis will also seek to explore this further in subgroups to identify those in which non-inferiority with a 2-month regimen might already be achievable with a high degree of likelihood. In the (unlikely) event that Bayesian probability exceeds 95% that the difference between a boosted regimen and the standard regimen lies within the pre-specified margin, then we will declare the boosted regimen to be non-inferior. Given the sample size limitations, the efficacy comparison against the standard regimen will only be done for the boosted regimens in *complete* TMS arms.

In addition to the comparison of the boosted regimens with the standard regimen, the probability that the absolute rate of unfavourable response is no worse than 20% will be calculated using Bayesian probability for each of the TMS arms (*complete* and *partial*) and the SMS arm.

The frequency of adverse events (incident adverse events censored 30 days after discontinuing a boosted regimen) in the *complete* TMS arms will be compared with the standard treatment regimen (in the SMS arm) using standard tests for comparing proportions (below). The frequency of adverse events occurring on the boosted regimens in the *partial* TMS arms will be presented as descriptive data but without formal statistical comparison to the standard regimen.

Data analysis of the primary outcome and secondary outcomes will control for site and baseline risk stratum (lower, intermediate, higher). Sites recruiting fewer than 30 subjects will be pooled geographically (e.g., within country). Controlling for site may be omitted if data anlaysis results become unstable with adjustment for site.

Given the exploratory nature of the analysis and the presentation as a descriptive probability, no formal adjustment for multiplicity will be done. However, significance tests will be interpreted

in the context of the number of comparisons performed. The study report will include a statement of the number of signficiance tests performed for subgroup analyses of the primary outcome, for secondary outcomes and for other outcomes along with a statement of the number of statistically-significant results that would be expected to occur by chance alone, following the approach recommended by Wang et al (NEJM 2007).

Summary statistics will be presented by treatment arm. For secondary outcomes that are expressed as proportions, Cochran Mantel Haenszel $\chi 2$ test controlling for study site and baseline risk stratum will be used to compare arms, with a P value less than 0.05 taken to indicate a significant difference. Common relative risk adjusted for site and baseline risk stratum will be provided with its 95% CI. Fisher's exact test will be used for comparison of proportions with few events (defined as more than 20% of the expected counts are less than 5 in the contingency table).

Analyses comparing trial regimens on outcomes related to bactericidal activity are described separately in section 12 below.

11.2 Study population and treatment arms

Analysis populations

The following study populations will be used for the analyses of regimen efficacy and safety.

The mITT, exposed population (defined in section 6.4) will be used for the main analysis, sensitivity analyses and subgroup analyses of the primary outcome.

The mITT exposed, assessable population; mITT exposed, culture positive population (defined in section 6.5) and mITT exposed, fully susceptible population (defined in section 6.5) will be used for sensitivity analyses of the primary outcome.

There is no per-protocol population defined for the regimen analysis because the decision criteria used for classifying outcome on relapse-free cure already include several major criteria reflecting adherence to protocol. As a result, the mITT assessable population therefore resembles many of the usual aspects of a per-protocol population. To evaluate further the effect of non-adherence on the primary regimen outcome, additional sensitivity analyses will be performed in which the analysis population will exclude only patients with more severe non-adherence (> 14 days missed over the first 56 days) or exclude patients even with modest levels of non-adherence (> 3 days missed over the first 56 days).

Secondary outcomes that relate to regimen safety (adverse events) will be analysed using the *ITT exposed* population.

Analysis treatment arms

Analyses will be done on all TMS arms (*complete* and *partial*). However, for the *partial* arms results will be interpreted with caution due to the wider confidence intervals with smaller number of patients; and also the potential for bias introduced by early termination.

11.3 Enrollment and follow-up

This is as for the strategy analysis (section 8.3), except that the numbers in the CONSORT diagram will be shown for the mITT exposed population (which may differ in a small number of cases from the mITT population, used for the strategy analysis, that does not require exposure to the allocated trial regimen). The numbers in the assessable population and the reasons for exclusion will be shown in the table presenting the main trial results (below).

11.4 Demographics and Baseline Characteristics

Demographics and baseline characteristics will be summarised for the mITT exposed population (as defined for the strategy analyses, see section 8.4), with columns for each randomised arm and a total column. Numbers will differ slightly from the baseline characteristics of the strategy analysis (mITT population) due to the additional exclusion of participants who do not have exposure to the allocated trial regimen.

11.5 Exposure and Adherence to Study Medications

Participants who do not receive allocated treatment will be reported in the CONSORT diagram.

<u>Trial-wide regimen changes</u>
If any systematic dose changes were made during the trial, the numbers of patients starting at the original and modified doses will be reported; and the number of patients in the original dose group who switched to the modified dose (as a result of the study-wide recommendation) will be reported, with the median number of days of treatment taken at the original dose prior to switch (section 4.6.2).

<u>Treatment duration and daily doses</u>

The following will be presented for each of the treatment arms (all TMS arms and SMS arm):

1) Initial treatment course duration (pre-randomisation)
    - Duration, days: median, range
    - Number of daily doses taken, n: median, range
2) Initial treatment course duration (post-randomisation, strict randomised regimen)
    - Duration, days: median, range
    - Number of daily doses taken, n: median, range

The number and proportion of participants in each TMS arm that switched from the boosted regimen to standard treatment prior to completion of the initial regimen or who extended

treatment with standard treatment immediately following the initial regimen, and the median time to introduction of standard treatment will be presented for each of the arms.

Adherence

The following will be presented for each of the treatment arms:

Adherence during the initial strict randomised treatment course from baseline to cessation or switch from boosted treatment:

%: median, range
Number of missed days, in each of the following categories:
< 7 missed days, n (%)
7-13 missed days, n (%)
>14 missed days, n (%)

Proportion with ≥ 4 consecutive days missed

Treatment observation

The main method of treatment observation during the initial treatment course will be summarized across the study population, n, % by category (section 4.6.11)

11.6 Efficacy of the trial regimens

11.6.1 Main analysis of the primary outcome

The number and proportion of participants in each *complete* TMS arm and the SMS arm in the mITT exposed population who are classified as having unfavourable, favourable and unassessable outcome (definitions in Sections 4.13) will be provided, along with 95% Bayesian credible interval for those proportions.

The number of participants meeting the following component definitions of unfavourable and unassessable outcome will be listed in the following summary categories, without formal group comparison (summary listing of categories below; precise definitions given in 4.13). Participants that meet more than one criterion will be classified under the first criterion that is met during study follow-up.

Unfavourable outcome

- Treatment failure at switch to standard treatment
- Treatment failure at end of treatment (confirmed, unconfirmed; listed separately)
- Relapse (confirmed, unconfirmed; listed separately)
- Ongoing disease activity at week 96 (definitive, presumed, possible; listed separately)
- Death by week 96 by possible TB related cause

- Did not attend week 96 and lacks evidence of good outcome at last attended visit and/or has evidence of bad outcome on telephone assessment or friend/relative assessment at week 96

Unassessable outcome

- Did not complete initial treatment
- Missed > 7 days treatment in first 56 days
- Switched boosted to standard > 7 days, and without positive culture before switch
- Added new drugs (> 7 days)
- Treatment failure on cessation confirmed, different WGS
- Relapse confirmed, different WGS
- Restarted treatment for reason other than treatment failure or relapse
- Ongoing disease activity at week 96, definite or presumed, different WGS
- Ongoing disease activity at week 96, possible, at least one culture negative
- Single positive sputum culture at week 96
- Death by week 96 (cause unrelated to TB)
- Did not attend week 96 visit and had evidence of good outcome at last attended visit but lacks information from telephone visit (patient or friend/relative) to assess disease activity at week 96

The difference in proportion of participants with an unfavourable outcome between the boosted regimen in each *complete* TMS arm and the standard regimen in the SMS arm, will be presented as the Bayesian probability that the between-group difference in the percentage of participants who would attain unfavourable outcome status would be no worse than 12%. The approach will follow that of Laptook et al (2017) and use a flat uninformative prior distribution. A boosted regimen will be declared to be non-inferior to the standard regimen if the probability that the difference is no worse than 12% is at least 95% (justification for 12% non-inferiority margin given in section 12.1 above).

In addition to the comparison of the boosted regimens with the standard regimen, the probability that the absolute rate of unfavourable response is no worse than 20% will be calculated using Bayesian probability for each of the TMS arms (*complete* and *partial*) and the SMS arm.

11.6.2 Sensitivity analyses of the primary outcome

The following sensitivity analyses of the primary outcome for regimen efficacy will be performed, presenting the Bayesian probability that the difference in proportion of patients with unfavourable outcome in each *complete* TMS arm versus SMS arm is no more than 12%:

<u>Sensitivity analysis 1</u>

mITT exposed assessable population (unassessable patients excluded; section 6.4)

Sensitivity analysis 2
mITT exposed culture positive (section 6.5)

Sensitivity analysis 3
mITT exposed, fully susceptible (section 6.5).

Sensitivity analysis 4
mITT exposed, as per algorithm but if boosted regimen continues for more than 58 qualifying daily doses (increased from 56 to allow for error) count as unassessable (censor follow-up at 58 qualifying days of boosted treatment). (The aim of this sensitivity analysis is to assess efficacy of a strict 8 weeks' boosted treatment).

Sensitivity analysis 5
mITT exposed, as per algorithm but if more than 3 days missed in first 56 days, count as unassessable (censor follow-up on 3rd missed day).

Sensitivity analysis 6
mITT exposed, as per algorithm but if more than 14 days missed in first 56 days, count as unassessable (censor follow-up on 14th missed day).

Sensitivity analysis 7
mITT exposed, as per algorithm but with treatment failure at switch, treatment failure at end of treatment (*unconfirmed)* and relapse (*unconfirmed)* – all with WGS shows same strain, inconclusive or not done - classified as unassessable (censor follow-up at week 96).

Sensitivity analysis 8
mITT exposed, as per algorithm but if possible disease activity (i.e. criteria A and B met only) and no negative sputum sample (to refute disease activity), count as unassessable (censor follow-up at week 96).

Sensitivity analysis 9
mITT exposed, as per algorithm but if isolated positive culture at week 96 (no other ongoing disease activity research criteria met; and WGS shows same strain, inconclusive or not done; and there is no other negative sputum sample in the week 96 analysis window), count as unfavourable.

11.6.3 Possible relapses in post week-96 follow-up period

In addition to the above sensitivity analyses, the number of additional patients in each TMS arm that had been treated with a boosted regimen only (no switch to standard treatment) and had not had relapse or restarted treatment at or before week 96, who were subsequently started on TB treatment for possible relapse between week 96 and the close of trial visit will be

reported for each regimen (information obtained from the close of trial telephone visit); along with the additional total follow-up time post week 96 for that group as an indication (imprecise) of the possibility of late relapses in each regimen (4.6.9). This is regarded as supportive information for any conclusions drawn, not a formal sensitivity analysis.

11.6.4 Subgroup analyses of the primary outcome

The proportion of participants with unfavourable outcome on the primary analysis will be compared pairwise between each of the *complete* TMS arms and the SMS arm in a number of pre-specified subgroups, using the same population and definitions as the main analysis.

The subgroups have been selected based on baseline factors that might affect the primary outcome of the boosted regimen such as markers of higher disease burden (possibly increasing the frequency of treatment failure or relapse); or underlying disease (malnutrition, diabetes, older age, smoking-induced lung damage, chronic obstructive pulmonary disease) that might increase mortality associated with relapse.

The subgroups and subcategories that will be considered for analysis are:

- Sex
- Age (<35, 35-50, > 50)
- Country (of study site)
- Smoking status (never, current/past)
- HIV co-infection
- History of diabetes (defined in 4.7.6)
- History of Chronic Obstructive Pulmonary Disease
- Malnutrition (body mass index <18.5)
- Expanded CXR, extend of cavitation at baseline (absent, largest cavity ≤ 4cm, > 4cm)
- Expanded CXR, extent of disease at baseline (proportion total lung affected, <20, 20-50, >50%)
- Expanded GeneXpert cycle threshold [high, medium, low, very low]
- Expanded smear status at baseline (negative, scanty/1+, 2+, 3+)
- Baseline risk stratum using the standard or expanded baseline definition (decision criteria in section 4.10)
- Isoniazid resistance at baseline

If there are less than 30 participants in a subgroup category within either of the two arms (TMS arm and SMS arm) that are being compared, that subgroup analysis will not be performed, unless of special interest. In such cases, thresholds may be adjusted or subgroup categories consolidated to achieve the minimum number of participants; or the subgroup analysis will be omitted.

If a systematic dose change is made during the trial, an additional subgroup analysis will be performed with participants classified into two groups depending on the starting dose of the drug.

Additional exploratory subgroup analyses may be added later in the trial to include additional biomarkers as these are identified or as measurements become available.

11.6.5 Analysis of time-to-unfavourable outcome

In addition to the main analysis of unfavourable outcome as a binary variable, an analysis of time to unfavourable outcome will be performed for the main analysis using the classification and approach to censoring described in section 4.13. The analysis will be done in the mITT exposed population.

Time to unfavourable outcome for each treatment arm will be analysed using Kaplan-Meier methodology, and survival curves and median survival time (with 95% confidence interval) will be provided for all treatment arms. Pairwise comparison between each boosted regimen (limited to *complete* TMS arms) and the standard regimen will be performed using the log-rank test.

A hazard ratio (with 95% confidence interval and p-value) comparing time to unfavorable outcome with the boosted regimen in each of the complete TMS arms with the standard regimen in the SMS arm will be estimated using a Cox proportional hazards regression model, with adjustment for site (aggregated by country if sample size is smaller than 10 for at least one site) and baseline risk stratum (defined in section 4.10). The proportional-hazards assumption will be assessed using graphical methods.

A sensitivity analysis will be performed on the median time to unfavourable outcome and Hazard Ratio for time to unfavourable outcome without adjustment for site and other randomisation stratification factors.

11.6.6 Analysis of time to treatment failure or relapse

In addition to the main analysis of time to unfavourable outcome, an analysis of time to treatment failure/relapse (defined using criteria and censoring rules in section 4.9.10 and 4.9.11) will be performed using an identical approach to comparisons as that for time to unfavourbale outcome (above). The outcome classification and censoring rules overlap substantially with those used in the time to unfavourbale outcome analysis, except that the outcomes for treatment failure/relapse are purely microbiological, driven exclusively by culture results and without attributing additional unfavourable outcomes on the basis of clinical events.

11.6.7 Analysis of Chest X-ray change

Lesion penetration is critical for sterilising efficacy and it is known that penetration of TB lung varies by individual drugs. We will compare the change in CXR proportion of total lung affected from baseline to week 8 in the mITT exposed population in the *complete* TMS arms pairwise against the SMS arm by appropriate statistical test.

11.7 Safety of the trial regimens

11.7.1 General overview

Analysis of the safety of the TRUNCATE-TB regimens will be done in the ITT exposed population (section 6.2).

Safety outcomes will be compared between individual *complete* TMS Arms and the SMS arm during the initial treatment period only. Descriptive safety data will be presented for the individual *partial* TMS Arms, but no formal statistical comparison will be made with the standard regimen in the SMS arm.

<u>Censoring of adverse events</u>

For assessing the safety of the boosted regimens, incident adverse events (new events or progression of existing events to a higher grade, section 4.8) will only be counted if the date of onset (or progression) of the event occurs during the initial period of treatment with the strict randomised regimen (defined by first and last qualifying treatment day; interruptions and missed days are ignored, section 4.6.4) or within 30 days following the end of the strict randomised regimen (defined by the last qualifying day, section 4.6.4; these additional 30 days may include days on the standard regimen in patients that switch or extend with standard treatment).

Similarly, for assessing the safety of the standard regimen, incident adverse events in the SMS arm will only be counted they occur whilst the patient is on treatment with the standard regimen or within 30 days following the last qualifying day of the standard regimen. For this analysis the standard regimen may include a fluoroquinolone in place of one of the standard drugs or in addition to the standard drugs (for resistance or toxicity reasons) as described in section 4.6.1.

<u>Tables of reported adverse events, general approach</u>

Tables will show number (and %) of patients who experienced one or more events during the initial treatment period with the strict randomised regimen (plus 30 days) in each category and the total number of events in each category; by treatment arm (in columns). For each category a patient will only be counted once, even if there are multiple occurrences of the same type/category of event in the same patient.

The standard international order of medDRA SOCs will be used for all tables, with events listed by system organ class (SOC) and then subcategorised by preferred term (PT).

<u>Summary tables of reported adverse events</u>

A summary table will be presented including the following categories of events (general):
- AE (all, any grade)

- AE of grade 3 or 4 severity (all)
- AE of grade 3 or 4 severity related to TB medication
- AE (any grade) causing permanent dose reduction or cessation of any prescribed TB drug
- SAE (all, including death)
- SAE related to TB medication
- Death (all)
- Death related to TB medication

<u>Tables and listings of specific AE types</u>

(i) AE of Grade 3 or 4 severity (all) presented as a *table* with rows for total AEs, and each SOC and PT subcategory.

(ii) AE of Grade 3 or 4 severity considered related to TB medication presented as a *line listing* for each arm, with the SOC, PT and grade.

(iii) AE (any grade) causing permanent dose reduction or cessation of any prescribed TB drug, presented as a *line listing* for each arm with the SOC, PT and grade, date of event, and date of all TB drugs interrupted or ceased within 7 days prior to the start date of the AE and 14 days after the end date of the AE.

(iv) SAEs (all) presented as a *table* with rows for total SAEs and by SAE category (fatal, hospitalization, life-threatening, etc).

(v) SAEs considered related to TB drugs presented as a *line listing* for each arm, with the SOC and PT.

(vii) Drug toxicities of special interest (all; derived from SMQs), presented as a *table* with rows for each toxicity (defined in section 4.8)

<u>Statistical comparisons of reported AEs</u>

Safety outcomes on the boosted regimen in each *complete* TMS arm will be compared pairwise with the standard regimen in the SMS arm on the following secondary safety outcomes using the $\chi 2$ test (or Fisher's exact test if fewer than 5 events in any one group):

- Proportion of patients with at least one AE of grade 3 or 4 severity
- Proportion of patients with at least one SAE (all, including death)

All other adverse event types will be presented as descriptive data only and no formal statistical comparison between arms will be made.

<u>Changes in measured laboratory parameters</u>

Changes in measured laboratory parameters that may indicate systematic organ effects will be evaluated by comparing change from baseline to week 8 in all treatment arms using linear mixed effect models with repeated measurements.

The model will include:

- change from baseline as a response variable
- treatment, visit and randomisation stratification factors (trial site and risk stratum) as fixed effects; any trial site that has less than 10 participants enrolled will be aggregated with the next smallest trial site or sites within the same country to ensure a minimum sample size of 30 participants per site (or aggregate of sites); any country that has less than 30 participants in aggregate will be combined with the next smallest country to ensure a country sample size of at least 50 participants
- baseline as continuous covariate
- treatment by visit as interaction term
- patient as random effect
- visit will be included as a repeated effect in the model

An unstructured matrix will be used to model the variance-covariance structure within patient. If this model fails to converge, other variance-covariance structures - heterogeneous TEOPLITZ (TEOP), heterogeneous autoregressive of order one (ARH (1)), autoregressive of order one (AR(1)) or compound symmetry (CS) structures will be considered in the specified order to model the correlation between time points from the same patient.

The difference between LS-means of two treatment arms (standard treatment pairwise versus each boosted regimen), corresponding 95% confidence interval and p-value will be reported for each visit.

The following measured/derived laboratory parameters will be evaluated:

- Haemoglobin
- Absolute neutrophil count
- Platelets
- ALT
- Alkaline phosphatase
- Bilirubin

Mean absolute values of the above parameters will be shown in line graphs from baseline to week 12.

11.7.2 QTc Prolongation

The following will be tabulated by treatment arm for the initial treatment period. Definitions and handling of data are described in 4.7.2:

- Maximum QTcF, ms
- Proportion of patients with QTcF ≥500ms (confirmed): n, %
- Proportion of patients with QTcF ≥500 ms (unconfirmed): n, %
- Proportion of patients with QTcF increase ≥ 60 ms: n, %

If more than 20 participants in an arm have QTc ≥500 (unconfirmed), the median time to first QTc ≥ 500ms will be calculated.

11.7.3 Liver toxicity

The following will be tabulated by treatment arm for the initial treatment period (+30 days).

- Proportion of patients with maximum ALT ≥ 5 times upper limit of normal: n, %
- Proportion of patients with maximum ALT ≥ 10 times upper limit of normal: n, %
- Proportion of patients with maximum bilirubin > 2 times upper limit of normal, n (%)
- Proportion of patients with elevation of ALT ≥ 3 times upper limit of normal and total bilirubin > 2 times upper limit of normal in same episode, n (%)*

*Line listing, by treatment arm, of cases meeting this criterion with dates and results of all LFTs, details of all drugs taken (TB and non-TB drugs) with start and stop dates until end of episode.

12 Analysis of bactericidal activity of the regimens

12.1 Endpoints for analysis of the bactericidal activity of the regimens

Time to sputum culture conversion within 8 weeks from randomization*

Sputum culture conversion status at week 8

Change in time to positivity in sputum culture from baseline to week 8

12.2 Study population and treatment arms

The following study populations will be used for analyses of bactericidal activity of the regimens:
- mITT, exposed (defined in section 6.4)
- mITT, exposed, culture positive, fully susceptible (defined in section 6.5)

Analyses will be done on all randomised arms (including partial arms that did not meet the enrolment target for the TRUNCATE-TB Management Strategy).

Follow-up will be censored in patients who switch from boosted to standard regimen (censor on the last day that a dose of the boosted regimen was taken; ignore if take ≤ 7 days of standard regimen before return to boosted regimen) or in patients who took less than 49 qualifying days of boosted regimen during the first 56 days (censored at 7th missed dose); if both conditions met, the earliest date will be used for censoring.

12.3 Enrollment and follow-up

As for the regimen analysis, section 11.3 above except that the numbers in the CONSORT diagram will reflect follow-up only to the end of the analysis period (week 8).

12.4 Demographics and Baseline Characteristics

Demographics and baseline characteristics will be summarised for the mITT exposed population, as per the regimen efficacy analysis (section 11.4), with columns for each randomised arm and a total column.

12.5 Exposure and Adherence to Study Medications

Participants who do not receive allocated treatment will be reported in the CONSORT diagram.

Trial-wide regimen changes

If any systematic dose changes were made during the trial, the numbers of patients starting at the original and modified doses will be reported; and the number of patients in the original dose group who switched to the modified dose (as a result of the study-wide recommendation) will be reported, with the median number of days of treatment taken at the original dose prior to switch (section 4.6.2).

Treatment duration and daily doses

The following will be presented for each of the treatment arms (complete and partial arms):

Initial treatment course duration (pre-randomisation)

- Duration, days: median, range
- Number of daily doses taken, n: median, range

Initial treatment course duration (post-randomisation, strict randomised regimen; censored at week 8)

- Duration, days: median, range
- Number of daily doses taken, n: median, range

Number and proportion of participants in each arm that switched to standard treatment prior to week 8

Adherence

The following will be presented for each of the treatment arms:
Adherence during the initial strict randomised treatment course from randomisation to week 8 or cessation of boosted treatment:

%: median, range
Number of missed days, in each of the following categories:
< 7 missed days, n (%)
7-13 missed days, n (%)
>14 missed days, n (%)

Treatment observation
The main method of treatment observation during the initial treatment course will be summarized across the study population, n, % by category (section 4.6.11)
**Planned Tables: 7.5.1 (exposure), 7.5.2 (adherence),

12.6 Time to culture conversion by week 8

The main analysis will be done in the mITT, exposed population.

Analysis will be done in all arms.

The derivation of the time to culture conversion in individual patients is described in section 4.9.9. For this analysis, the designated end of the follow-up period is week 8.

In the main analysis, participants who do not meet the definition of culture conversion during the analysis period but have their first negative culture at the time of their last evaluable sputum culture during the analysis period will be classified as achieving culture conversion at that time.

Time-to-culture conversion for each treatment will be analysed using Kaplan-Meier methodology. The Kaplan-Meier survival curves and median survival time (with 95% confidence interval) will be provided for each treatment arm. Pairwise comparison between each boosted regimen and the standard regimen will be performed using the log-rank test.

A hazard ratio (with 95% confidence interval and p-value) comparing time to culture conversion in each boosted regimen with the standard regimen will be estimated using a Cox proportional hazards regression model, with adjustment for site (aggregated by country if sample size is smaller than 10 for at least one site) and baseline risk stratum (defined in section 4.10). The proportional-hazards assumption will be tested using Schoenfeld residuals, with a threshold of $p<0{\cdot}05$ indicating evidence of non-proportionality.

The following sensitivity analyses will be performed on the median time to culture conversion and Hazarad Ratio for time to culture conversion:

- Classifying as censored rather than culture conversion in the case where the last evaluable sample is negative (described above)
- Classifying no sputum produced associated with clinical disease recovery, or no sputum produced, met omission criteria samples as negative
- Without adjustment for site and other randomisation stratification factors
- Using the mITT, exposed, culture positive population (defined in section 6.5)
- Using the mITT, exposed, culture positive, fully susceptible population (defined in section 6.5)

12.7 Sputum culture conversion status at week 8

The analysis will be done in the mITT population. Analysis will be done in all arms.

The derivation of the time to culture conversion in individual patients is described in section 4.9.9. For this analysis, the designated end of the follow-up period is week 8.

The cumulative proportion with sputum culture conversion by week 8 in each arm will be estimated from the Kaplan-Meier curve of time to culture conversion (obtained as above). A 95% CI for the proportion will be provided for each treatment group.

12.8 Change in Time to Positivity (TTP)

The analysis will be done in the mITT, exposed population. Analysis will be done in all arms.

Analysis will use all evaluable TTP results as defined in section 4.9.8 obtained within the analysis period for each patient. The *analysis period* for each patient is defined (in the same way as for the calculation of time to culture conversion, section 4.9.9) as the time from baseline to the earliest of the following:

- The date of switch to standard treatment (for patients allocated to receive a boosted regimen, who switch for toxicity or other reasons).
- The designated end of follow-up, which for this analysis is week 8

The rate of change in $\log_{10}$TTP will be estimated using a robust Bayesian non-linear mixed effects regression model as follows (Burger 2018). This model, specifically designed for TTP data obtained from EBA studies, can handle missing data due to contamination, participant withdrawal, or negative cultures. The model accounts for correlations between the random intercepts and slopes over time. Study sites may enter into the model as a random effect.

$$log_{10}(y_{ijk}) = \alpha_{ij} + \beta_{1ij}t_{ijk} + \beta_{2ij}\gamma_{ij}log\left(\frac{e^{\frac{t_{ijk}-\kappa_{ij}}{\gamma_{ij}}} + e^{-\frac{t_{ijk}-\kappa_{ij}}{\gamma_{ij}}}}{e^{\frac{\kappa_{ij}}{\gamma_{ij}}} + e^{-\frac{\kappa_{ij}}{\gamma_{ij}}}}\right) + \varepsilon_{ijk},$$

where y_{ijk} is the TTP value for patient $j = 1, \ldots, N_i$ in treatment arm $i = R, C$ at time point k and $t_{ijk} \geqslant 0$ is the corresponding measurement time; α_{ij} are the random intercepts, β_{1ij} and β_{2ij} the 2 random slopes, κ_{ij} the inflection point, γ_{ij} the "smoothness" parameters, and ε_{ijk} are the residuals.

In this model, samples that are culture negative will be entered as a censored value of $\geqslant$ 42 days; samples that are "TB positive and contaminated" will be entered as a censored value of < 42 days; samples that are 'contaminated only' or 'other', and no samples due to patients unable to produce sputum or missed visit will be entered as a missing value.

The rate of change in $\log_{10}$ TTP from time t_1 to t_2 in treatment arm i is calculated as follows

$$\frac{f_i(t_2) - f_i(t_1)}{t_2 - t_1}$$

where $f_i(t) = \alpha_i + \beta_{1i}t + \beta_{2i}\gamma_i log\left(\frac{e^{\frac{t-\kappa_i}{\gamma_i}} + e^{-\frac{t-\kappa_i}{\gamma_i}}}{e^{\frac{\kappa_i}{\gamma_i}} + e^{-\frac{\kappa_i}{\gamma_i}}}\right)$, and α_i, β_{1i}, β_{2i}, κ_i, γ_i is the mean of random effects α_{ij}, β_{1ij}, β_{2ij}, κ_{ij}, γ_{ij}, respectively. Based on the fitted model, the rate of change in $\log_{10}$TTP from baseline to week 8 and its 95% Bayesian credible interval (BCI) will be provided for each treatment arm.

Rates of change in TTP will be compared pairwise between the boosted regimen arms and the control arm.

A line plot of posterior estimate of mean $\log_{10}$ (TTP) will be produced by treatment arm, with shaded area indicating the 95% BCI.
